# Dissecting the bacterial type VI secretion system by a genome wide *in silico *analysis: what can be learned from available microbial genomic resources?

**DOI:** 10.1186/1471-2164-10-104

**Published:** 2009-03-12

**Authors:** Frédéric Boyer, Gwennaële Fichant, Jérémie Berthod, Yves Vandenbrouck, Ina Attree

**Affiliations:** 1 CEA, iRTSV, Laboratoire Biologie, Informatique et Mathématiques, F-38054 Grenoble, France; 2 Université de Toulouse, UPS, Laboratoire de Microbiologie et Génétique Moléculaires, F-31000 Toulouse, France; 3 Centre National de la Recherche Scientifique, LMGM, F-31000 Toulouse, France; 4 CEA, iRTSV, Laboratoire Biochimie et Biophysique des Systèmes Intégrés, F-38054 Grenoble, France; 5 UMR5092, Centre National de la Recherche Scientifique/CEA/Université Joseph Fourier, F-38000 Grenoble, France

## Abstract

**Background:**

The availability of hundreds of bacterial genomes allowed a comparative genomic study of the Type VI Secretion System (T6SS), recently discovered as being involved in pathogenesis. By combining comparative and phylogenetic approaches using more than 500 prokaryotic genomes, we characterized the global T6SS genetic structure in terms of conservation, evolution and genomic organization.

**Results:**

This genome wide analysis allowed the identification of a set of 13 proteins constituting the T6SS protein core and a set of conserved accessory proteins. 176 T6SS loci (encompassing 92 different bacteria) were identified and their comparison revealed that T6SS-encoded genes have a specific conserved genetic organization. Phylogenetic reconstruction based on the core genes showed that lateral transfer of the T6SS is probably its major way of dissemination among pathogenic and non-pathogenic bacteria. Furthermore, the sequence analysis of the VgrG proteins, proposed to be exported in a T6SS-dependent way, confirmed that some C-terminal regions possess domains showing similarities with adhesins or proteins with enzymatic functions.

**Conclusion:**

The core of T6SS is composed of 13 proteins, conserved in both pathogenic and non-pathogenic bacteria. Subclasses of T6SS differ in regulatory and accessory protein content suggesting that T6SS has evolved to adapt to various microenvironments and specialized functions. Based on these results, new functional hypotheses concerning the assembly and function of T6SS proteins are proposed.

## Background

Complex interactions between Gram-negative bacteria and their environment are facilitated by numerous surface-attached and exported macromolecules, some of which represent bacterial toxins and effectors. In order to cross two bacterial membranes, those molecules are transported by nanomachineries, called secretion systems, which may be more or less complex in terms of their composition and regulation. Up to recently, five distinct secretion systems have been identified in Gram-negative bacteria [1,2]. In 2006, two groups presented evidences on the existence of a novel secretion system in *Vibrio cholerae *[3] and *Pseudomonas aeruginosa *[4], and named it the Type VI Secretion System (T6SS).

Both systems export the Hcp (Haemolysin-Coregulated Protein) and presumably a class of proteins named Vgr (Val-Gly Repeats), whose exact function is still speculative. In *V. cholera*e, three Vgrs (VgrG1-3) are encoded in the genome, and are exported in a T6SS-dependent way. All N-terminal domains of Vgr proteins show strong homology with bacteriophage T4 proteins gp27 and gp5, which are constituents of phage baseplate [5], and are able to co-associate [6]. On the contrary, the C-terminal domains are Vgr-specific and some of them seem to carry an "activity" function, as illustrated by *V. cholerae *VgrG1 which can cross-link cellular actin [6].

The genes encoding T6SS have been reported a few years ago as being present in different bacterial species, although it was not clear at that time whether those genes act together or are important in bacteria-host interactions [7]. Recent reports demonstrated the importance of T6SS in pathogenesis of several bacterial species. *Burkholderia mallei *uses T6SS to proliferate in macrophages and an Hcp-related protein is produced *in vivo *during infection of model animals [8]. The fish pathogen *Edwardsiella tarda *has an active T6SS [9]. Hcp1 of *P. aeruginosa *is actively secreted by clinical isolates and cystic fibrosis patients develop antibodies against Hcp, demonstrating that the system is active during infection [4]. In addition to Vgr and Hcp proteins, the actual hallmark of this novel system is the presence of an AAA+ Clp-like ATPase and of two additional genes *icmF *and *dotU*, encoding homologs of T4SS stabilising proteins [10].

As in the case of the majority of virulence factors, the expression of T6SS is tightly controlled either at transcriptional or post-transcriptional level. One of the four T6SS encoded in the genome of *B. mallei*, found to be required for virulence in the hamster model of infection, is under the control of the VirAG two-component system and an AraC-type regulator [8]. The expression of the T6SS of *Burkholderia cenocepacia *and *P. aeruginosa *is regulated by a similar sensor kinase containing seven transmembrane domains and belonging to the 7TMR-DISMED (7 TransMembrane Receptors with Divers Intracellular Signalling Modules) protein family [11]. Finally, the activity of T6SS of *P. aeruginosa *is regulated by a Ser/Thr kinase and phosphatase able to act on the FHA (Fork Head Associated)-containing component of the machinery [12].

All together, these findings provide strong evidence that the T6SS is important for bacterial pathogenesis. However, as a new secretion system, little information is available about the structural and the genomic organization of the T6SS apparatus considering the vast amount of microbial genomic data available. The first *in silico *study, carried out in 2003, reported the presence of 27 homologs of *icmF *gene within 16 bacterial genomes belonging to the Gram-negative Proteobacteria division [13]. In that study, the phylogenetic analysis performed on three proteins (IcmF, ClpV1 and DotU) from the IAHP (IcmF Associated Homologous Proteins) cluster suggested that some bacteria acquired this cluster by lateral transfer. This was further confirmed through a phylogenetic analysis of two other conserved protein sequences (IglA/B) [14]. Recently, a similar study based on ortholog search and focused on functional annotation [15] detected T6SS in 42 pathogenic proteobacteria. However, the approach that was employed could not detect if there are more than one T6SS per genome. Thus, in order to investigate thoroughly T6SS regarding its phylogenetic distribution, gene content, organization and evolution we undertook a large-scale genome screening approach by designing an *in silico *strategy using more than 500 available bacterial genomes. Finally, some specific topics are discussed, such as the VgrG protein family and their potential role in host-pathogen interaction.

## Results and Discussion

### Conserved T6SS primary core building and genome wide scanning

**Figure 1:**
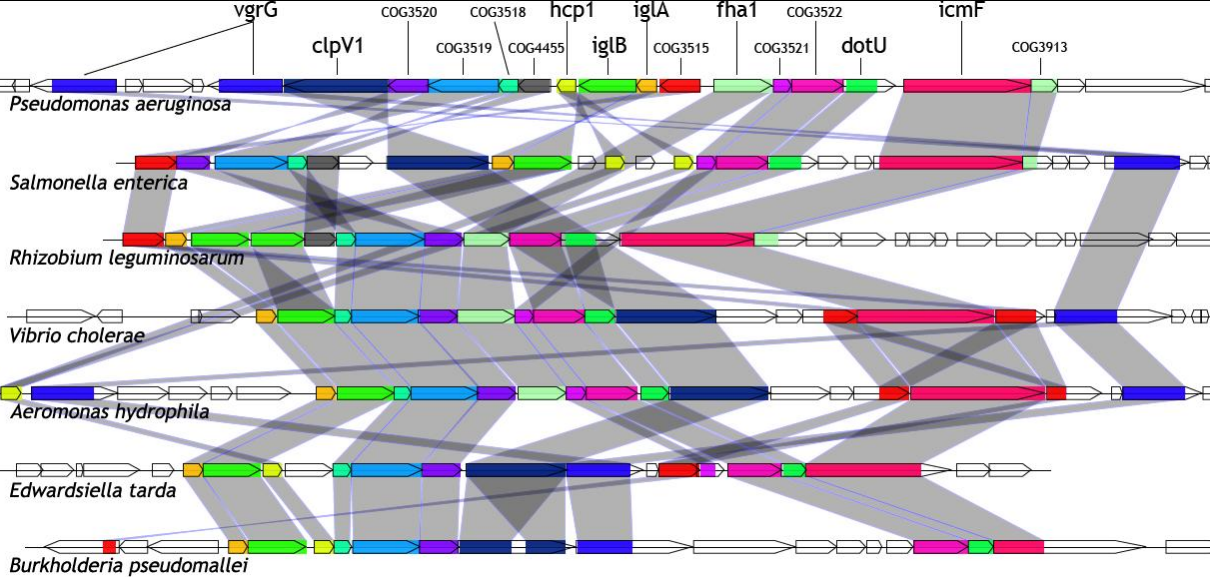
**Genomic organization of the characterized T6SS loci**. Genes are represented as arrows. Translated sequences of the most conserved proteins were aligned to the COG sequences and the hits are represented as coloured boxes. A unique colour is assigned to highly conserved COGs. *Burkholderia mallei *is closely related to *B. pseudomallei*, only this latter one is depicted.

First, a list of the 16 conserved genes encoded in T6SS gene clusters demonstrated to be functional in virulence have been manually established (Table 1 and Figure 1). They are characterized by 16 different COGs (Cluster of Orthologous Groups of proteins, defined in [16]). Thus, each COG (listed in Table 1) was used as a bait to retrieve potential T6SS encoding regions from the 506 complete genomic sequences of the Genome Reviews Repository (September 2007, see Materials and methods §1). The retrieved genomic regions are referred later in the text as *T6SS encoding regions *or *T6SS loci*. We were able to identify 176 different loci from 92 different bacteria.

**Table 1:** Conserved T6SS homologs

COG id	Gene name	Pseudomonas aeruginosa	Vibrio cholerae	Rhizobium leguminosarum	Salmonella enterica	Edwardsiella tarda	Burkholderia pseudomallei	Burkholderia mallei	Aeromonas hydrophila
		AE004091_GR^a^	AE003853_GR^b^	AF361470^c^	AJ320483^d^	AY424360^e^	BX571966_GR^f^	CP000011_GR^g^	CP000462_GR^h^
COG3913		PA0076	-	impM	sciT	-	-	-	-
COG3523*	icmf	PA0077	VC_A0120	impL	sciS	evpO	BPSS1511	BMAA0729.1 (tssM)	AHA_1845
COG3455*	ompA/motB/dotU	PA0078	VC_A0115	impK	sciP	evpN	BPSS1510	BMAA0731 (tssL)	AHA_1840
COG3522*		PA0079	VC_A0114	impJ	sciO	evpM	BPSS1509	BMAA0732 (tssK)	AHA_1839
COG3521*		PA0080	VC_A0113	-	sciN	evpL	BPSS1508	BMAA0733 (tssJ)	AHA_1838
COG3456	fha1	PA0081	VC_A0112	impI	-	-	-	-	AHA_1837
COG3515*		PA0082	VC_A0119, VC_A0121	impA	sciA	evpK	BPSS1493	BMAA0747 (bimE)	AHA_1844, AHA_1846
COG3516*	iglA	PA0083	VC_A0107	impB	sciH	evpA	BPSS1496	BMAA0744 (tssA)	AHA_1832
COG3517*	iglB	PA0084	VC_A0108	impC, impD	sciI	evpB	BPSS1497	BMAA0743 (tssB)	AHA_1833
COG3157*	hcp1	PA0085	-	-	sciK, sciM	evpC	BPSS1498	BMAA0742	AHA_1826
COG4455		PA0086	-	impE	sciE	-	-	-	-
COG3518*		PA0087	VC_A0109	impF	sciD	evpE	BPSS1499	BMAA0741 (tssC)	AHA_1834
COG3519*		PA0088	VC_A0110	impG	sciC	evpF	BPSS1500	BMAA0740 (tssD)	AHA_1835
COG3520*		PA0089	VC_A0111	impH	sciB	evpG	BPSS1501	BMAA0739 (tssE)	AHA_1836
COG0542*	clpV1	PA0090	VC_A0116	-	sciG	evpH	BPSS1502	BMAA0738	AHA_1841
COG3501*	vgrG	PA0091, PA0095	VC_A0123	-	vrgS	evpI	BPSS1503	BMAA0737	AHA_1827, AHA_1848
localisation for complete chromosome		83380..122266	115142..141407				2036034..2064669	735641..771918	1990515..2021714
Reference		[4]	[3]	[7]	[49]	[9]	[50]	[8]	[28]

Conserved T6SS homologs together with gene and COG names, encoded in experimentally studied T6SS. ^a^*Pseudomonas aeruginosa *(strain LMG 12228/ATCC 15692/PRS 101/1C/PAO1); ^b^*Vibrio cholerae *(serovar O1, strain ATCC 39315/El Tor Inaba N16961); ^c^*Rhizobium leguminosarum *bv. trifolii imp gene locus, complete sequence; ^d^*Salmonella enterica subsp. enterica serovar Typhimurium *DNA for centisome 7 genomic island; ^e^*Edwardsiella tarda *strain PPD130/91 putative transporting ATPase and hypothetical proteins genes, complete cds; type VI secretion system gene cluster; ^f^*Burkholderia pseudomallei *(strain K96243); ^g^*Burkholderia mallei *(strain ATCC 23344); ^h^*Aeromonas hydrophila *(subsp. hydrophila, strain ATCC 7966/NCIB 9240). Not included in tab: *Escherichia coli EC042 *(no annotated sequence published) [51], *Francisella tularensis tularensis *(see Result and Discussion) (AJ749949_GR 717996..738073) [33]

**Figure 2:**
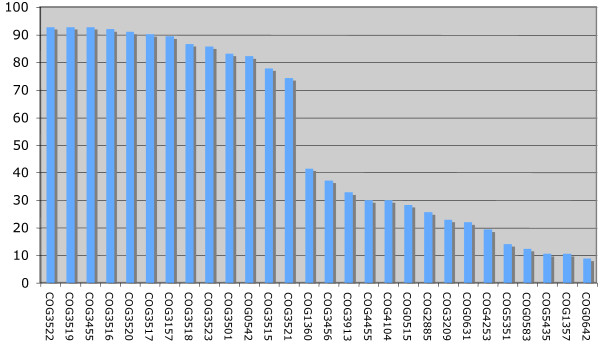
**T6SS genetic component frequencies**. Frequencies were computed on the basis of all identified T6SS gene clusters except clusters encoded from closely related bacterial strains (in this case, only the clusters encoded in one strain has been taken into account).

Each selected locus contained at least five genes predicted to encode proteins showing significant similarities (e-value ≤ 10^-6^) with the list of the 16 bait COGs. In order to estimate the degree of conservation of each COG in T6SS, we counted the number of loci where the COG was detected. A group of 13 COGs with frequency higher than 70% is clearly identified (Figure 2, these 13 COGs are marked with an asterisk in Table 1). All of these COGs were previously identified in the eight experimentally characterized T6SS, except the COG3157 which corresponds to the Hcp1 protein that is absent in the *V. cholerae *locus, albeit located elsewhere within its genome.

To estimate the ability of these 13 COGs to specifically predict T6SS, we investigated their phylogenetic distribution. Eleven COGs are found only in organisms where at least one T6SS locus has been predicted. Therefore, they appear specific to this secretion system. Among the two remaining COGs, COG3501 shows a clear correlation with T6SS associated organisms but is also present in other groups of bacteria including bacteroides (anaerobic gram-negative bacteria). On the contrary, COG0542 ('ATPases with chaperone activity') is clearly not specific to T6SS and picks up different sub-families of bacterial ATPases. A previous phylogenetic tree constructed by Schlieker *et al*., which includes homologs of ClpB and ClpV ATPases, discriminates clearly the two subfamilies [17]. However, the COG0542 is not specific enough to perform this discrimination.

### T6SS taxonomic distribution

The taxonomic distribution of T6SS was deduced from the phyla associated to the different bacteria harbouring at least one T6SS gene cluster. All but two of the bacterial species (*Rhodopirellula baltica *and *Solibacter usitatus*) belong to Proteobacteria, in agreement with results published recently [14]. It is noteworthy, that among the 13 epsilon proteobacteria genomes, only *Helicobacter hepaticus *presents a unique predicted T6SS. In addition, this cluster seems to have been acquired by horizontal gene transfer (see below section 4). Therefore, it appears that T6SS is mainly found in Proteobacteria, except in the epsilon sub-group. The rarity of T6SS in the epsilon sub-group may be due to specific traits of this later group as it branches at the root of the proteobacterial phylogenetic tree [18].

### Description and functional association of most conserved T6SS components

Among the 13 most conserved proteins, IcmF and DotU (COG3523 and COG3455) have firstly been studied in the context of T4SS secretion and have been shown to be necessary for efficient T4SS-dependent secretion. IcmF homologs are predicted to have three transmembrane helices hence most probably associated with bacterial membranes. Both proteins have been shown to be required for T4SS apparatus stabilization in *Legionella pneumophilla *[10].

The ClpV homologs (COG0542) belong to a sub type of ATPAse AAA+ family, being specific to T6SS gene clusters [17]. This group lacks the disaggregating function found in the ClpB type, but it is still able to take part into translocation processes. Therefore, it has been proposed that ClpV proteins provide energy to the protein secretion process [17]. Indeed, ClpV is essential for a T6SS function [19].

In *Francisella*, IglA and IglB (COG3516 and COG3517) are cytoplasmic proteins which interact directly and are required for intracellular growth in macrophages [20]. The authors propose that the IglAB complex plays a role of chaperone-like proteins involved in secretion pathway of virulence factors. Indeed, homologous proteins in Vibrio, named VipA et VipB, form complexes and interact with ClpV [19].

VgrG and Hcp proteins, both identified as conserved in T6SS loci, have been found in culture supernatant in T6SS dependent manner, in various organisms. However, it is still an ongoing debate whether these two proteins are constituents of the T6SS apparatus or are involved in translocation into host cell or are toxic *per se*. Besides, Pukatzki and co-workers hypothesized that three VgrG proteins assemble into a membrane-penetrating device whose structure is analogous to the bacteriophage T4 tail spike complex. Indeed, VgrGs seem to be a fusion of phage tail proteins gp27 and gp5 [6]. Interestingly, another protein (COG3518), present in most of the analyzed loci, shows weak similarities to a second bacteriophage T4 protein (PFAM: 'GPW/gp25 family protein', putative lysozyme). The quaternary structure of the bacteriophage T4 baseplate suggests that gp25 and gp27 are in close proximity in the baseplate and may physically interact [5]. However, in *Edwardsiella tarda*, yeast two-hybrid experiments do not detect any interaction between EvpI (VgrG homolog) and EvpE (COG3518). Although, the *ΔevpE *mutant shows defect in the secretion of EvpC (Hcp homolog), EvpE itself does not appear to be secreted [9].

**Figure 3:**
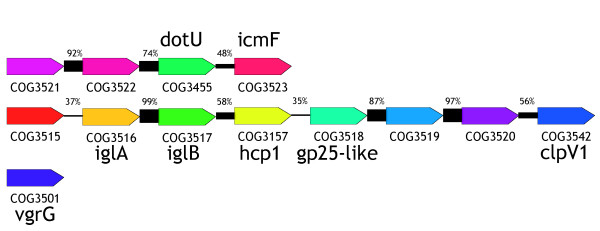
**T6SS consensual genomic organization**. COGs are depicted as arrows. The colour scheme is as on Figure 1. When the order and the transcriptional orientation of two genes are conserved, the two corresponding COGs are linked. The more the genomic organization is conserved, the bolder the connectors. The percentages correspond to the link conservation frequencies. They were computed on the basis of all identified T6SS gene clusters except clusters encoded from closely related bacterial strains.

To further characterize functional links that may exist between T6SS components, we decided to focus on the conserved genomic organization, as it often suggests co-translation and/or protein-protein interactions [21]. The genomic organization among T6SS loci was investigated by computing the number of loci in which every pair of COGs could potentially be co-transcribed (i.e. two COGs are encoded in tandem and co-oriented). As shown on Figure 3, it clearly appears that T6SS gene clusters are strongly biased in their organization suggesting that these 13 proteins are part of a small-scale network of physical and functional interactions. From this analysis, three groups of conserved organization can be clearly delineated:

• COG3516 (IglA), COG3517 (IglB) and COG3157 (Hcp). As mentioned previously, the complex IglAB may play a role of chaperone-like proteins involved in secretion pathway.

• COG3521 ('Putative prokaryote lipoprotein'), COG3522 (unknown function), COG3455 (DotU homologs) and COG3523 (IcmF homologs). Sequence analyses performed on those proteins reveal: i) a predicted signal peptide in 86% of the sequences associated to COG3521, ii) one predicted transmembrane helix but no signal peptide in 87% of the DotU sequences, iii) three transmembrane helices in 63% of the IcmF sequences (84% have at least one predicted transmembrane helix) and iv) no signal peptide or transmembrane segment in all COG3522 sequences. The results obtained on DotU and IcmF proteins are in accordance with experiments conducted in the context of T4SS. Moreover, recent experiments in *E. coli *have confirmed the lipoprotein function of SciN, the protein of the T6SS locus retrieved by the COG3521 [22].

• COG3518 (gp25-like), COG3519 (unknown function) and COG3520 (unknown function). Most of the sequences associated to these COGs have neither a predicted signal peptide nor transmembrane helix, suggesting that they might be cytoplasmic proteins.

### T6SS phylogenetic analysis and profiles

**Figure 4:**
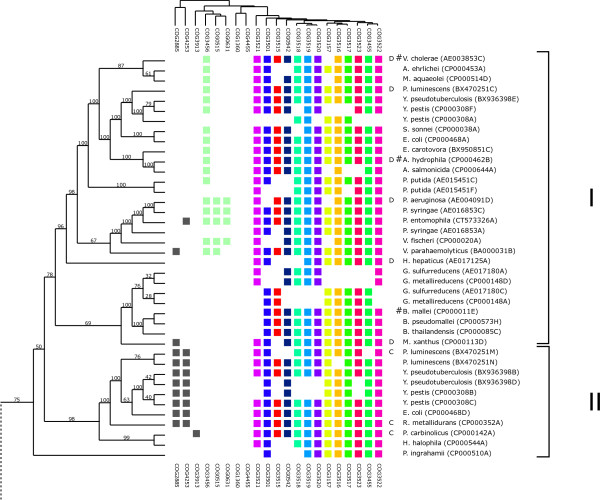
**Relationship between phylogeny and T6SS gene content**. Rows represent T6SS loci and columns represent protein functional classes (based on COG assignment). The tree on the left is the consensus phylogenetic tree (super-tree, see Materials and methods §5, manually rooted) whereas the upper dendrogram represents a hierarchical clustering of the phylogenetic profiles. Core T6SS conserved proteins are depicted on the right columns with the same color code as in Figures 1 and 3. Presence and absence of conserved accessory proteins (grey and light green) highlights the presence of sub-groups numbered from I to V. Letters in front of bacteria names correspond to the four groups proposed by Bingle and co-workers [14], '#' marks functional T6SS loci depicted in Table 1 and included in this figure. The two major sub-trees have been split and displayed separately for clarity. Light blue and light green highlighted groups are examples of close T6SS loci associated to bacteria with similar ecological niche. These two groups are associated to marine bacteria (sub-group V, blue) and plant associated bacteria (sub-group IV, green).

**Figure 5:**
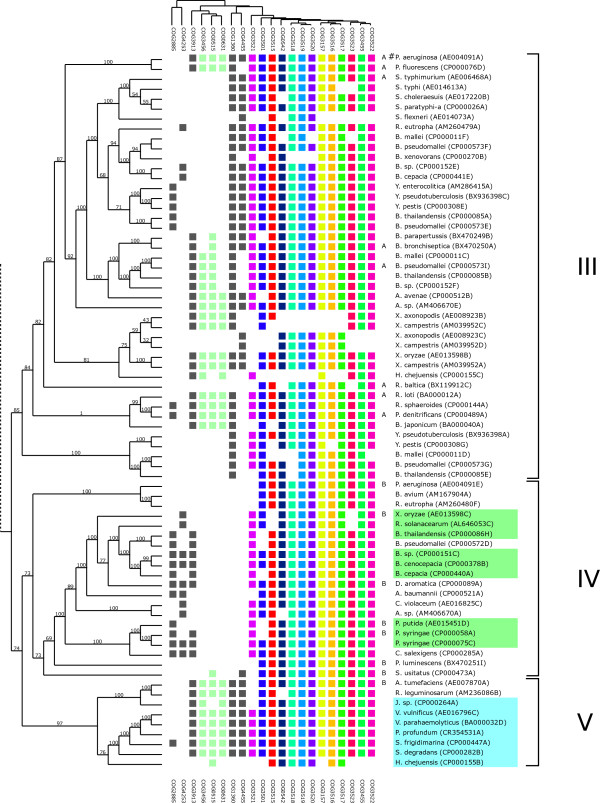
**Relationship between phylogeny and T6SS gene content**. Rows represent T6SS loci and columns represent protein functional classes (based on COG assignment). The tree on the left is the consensus phylogenetic tree (super-tree, see Materials and methods §5, manually rooted) whereas the upper dendrogram represents a hierarchical clustering of the phylogenetic profiles. Core T6SS conserved proteins are depicted on the right columns with the same color code as in Figures 1 and 3. Presence and absence of conserved accessory proteins (grey and light green) highlights the presence of sub-groups numbered from I to V. Letters in front of bacteria names correspond to the four groups proposed by Bingle and co-workers [14], '#' marks functional T6SS loci depicted in Table 1 and included in this figure. The two major sub-trees have been split and displayed separately for clarity. Light blue and light green highlighted groups are examples of close T6SS loci associated to bacteria with similar ecological niche. These two groups are associated to marine bacteria (sub-group V, blue) and plant associated bacteria (sub-group IV, green).

In order to identify sub-groups of T6SS, we performed a phylogenetic analysis on the T6SS encoding loci. A tree was built on sequences of each protein family found at the different loci. Then, the trees were compared in order to decide whether or not they can be considered as the result of a single evolutionary scenario, *i.e*., if the core genes we identified co-evolved and have been transmitted (by horizontal transfer or not) as a single functional unit. The similarities between the trees were computed using ***TOPD/FMTS ***[23] with the split distance (see Materials and methods §5). A hierarchical classification was applied on these distances. It suggests that the set of trees is homogenous as it is not possible to clearly identify different groups of trees in the resulting dendrogram [see Additional file 1]. Nevertheless, among the 13 trees, the ones associated to Hcp (COG3157), VgrG (COG3501), COG3521 (lipoprotein) and another protein of unknown function (COG3522) show the greatest distances to the other trees. As these four trees correspond to proteins either secreted (VgrG and Hcp) or exposed to the cell surface (COG3521), their relative differences may be explained by a higher rate of mutation probably due to their direct interaction with environment or host cells. We thus decided to discard them for further computations. Using the weighted MRP method [24], we combined these trees to reconstruct a super-tree that would reflect a consensual scenario of T6SS evolution (Figure 4 &5). Besides being acknowledge to give accurate results [25], the weighted MRP method also offers the possibility to integrate in a unique tree all loci, even if they share only a few common genes.

In the super-tree, each leaf corresponds to one of the 176 T6SS loci. First, the clusters of loci obtained on the tree were analyzed in terms of COG contents. We have defined five sub-groups (Figure 4 &5) according to the tree topology and the presence or absence of conserved accessory proteins. The four T6SS groups proposed by Bingle and co-workers (based on concatenated sequences of IglA and IglB for only 37 loci) have been placed on the super-tree [14]. The loci corresponding to these four groups belong to four different clusters on the tree. Therefore, the two classification obtained through different approaches are in agreement. However, we demonstrated here that it extends to the 13 core genes that can then be considered as one single functional unit. The analysis of our resulting tree can also give more insights regarding T6SS evolution. From the tree topology and the gene content of each locus, we can infer that some T6SS have been split into two different loci after their acquisition by the bacteria. Indeed, both loci are clustered on the tree and their gene contents are complementary, forming a complete T6SS [*Geobacter sulfurreducens *(AE017180A-AE017180C) and *metallireducens *(CP000148A-CP000148D), *Xanthomonas axonopodis *(AE008923B-AE008923C) and *campestris *(AM0399952C-AM039952D), *Yersinia pestis *(CP000308F-CP000308A)]. As the two split loci found in *Hahella chejuensis *localized distantly on the tree and belong to two different sub-groups (III and V), they should have been acquired through two independent events followed by gene lost. On the other hand, leaf clustering can also reveal recent duplications of T6SS gene clusters. We can detect two such events, one in *Pseudomonas putida *(AE015451C-AE015451F) and the other in the common ancestor of *Yersinia pestis *and *Yersinia pseudotuberculosis *(CP000308B-CP000308C and BX936398B-BX936398D). In both cases, gene lost in one of the duplicated cluster is observed. The tree also highlights a large fraction of organisms that possess multiple T6SS gene clusters (T6SS copy numbers are listed in a supplementary table [see Additional file 2]). Some of these clusters are either incomplete or with different gene contents. The most striking examples come from the pathogenic bacteria *Yersinia pestis *and *Burkholderia pseudomallei *that encode up to six T6SS gene clusters. The distant location on the tree of the different loci suggests that these duplication events are not recent. Given that these copies are ancient, belong to different sub-groups, and have been fixed, we can make the hypothesis that they may have specialized into specific functions.

Furthermore, a strong correlation between the loci gene content and some subtrees can be observed. COG0515, COG0631, COG1360, COG3456, COG3913 and COG4455 cluster together in different sub-trees. Interestingly, three of these COGs correspond to proteins involved in the post-translational regulation mechanism of the T6SS in *Pseudomonas aeruginosa *[12]: PpkA (a Thr/Ser kinase, COG0515), PppA (a phosphatase, COG0631), and Fha1 (a protein containing a fork-head motif, COG3456). Except in sub-group I, the correlation is even more pronounced between COG3913 and Fha1 (COG3456), which may indicate a functional role of COG3913 in the PpkA/PppA/Fha regulatory pathway. The association between COG1360 ('MotB, flagellar motor protein') and Fha1 (COG3456) has already been reported in a gene co-occurrence/gene neighbourhood study [26]. However, in our case, this association is not always strictly observed.

The sub-groups II and IV can be characterized by the co-occurrence of two COGs: COG2885 ('Outer membrane protein and related peptidoglycan-associated (lipo)proteins') and COG4253 ('uncharacterized protein conserved'). As the domain encoded by COG4253 appears fused with the N-terminal domain of a sub-group of VgrG proteins (see below), the observed correlation could reflect a possible interaction between the COG2885 protein and VgrG through the COG4253 domain. A putative role of this interaction could be the modulation of the VgrG function (secretion or translocation and virulence).

To further explore the specificities of sub-trees in terms of sequence conservation, we compared our tree with a clustering obtained on the proteins by applying the ***TribeMCL ***strategy [27] which is based only on sequence similarities (see Materials and methods §4). This alternative approach is mainly useful to correlate some of the sub-trees with the presence of specific COGs: COG3456 (Fha1), COG3515, COG3518, COG3520, COG3521. Moreover, another important feature revealed by the ***TribeMCL ***clustering is the definition of a small sequence cluster whose members are mainly associated to COG2204 ('Response regulator containing CheY-like receiver, AAA-type ATPase, and DNA-binding domains') [see Additional file 3]. Loci belonging to that cluster are mainly found in the previously defined sub-group I (Figure 4) that contains the *V. cholera *locus whose regulation does not depend of the PpkA/PppA/Fha mechanism described for *P. aeruginosa *[12]. The COG2204 member of the *V. cholerae *locus corresponds to the sigma 54 activator [3], which is conserved in *Aeromonas hydrophila *and has been shown to be necessary for T6SS expression in that organism[28].

All together, these observations suggest that i) the genes whose products are involved in the assembly of the putative secretion apparatus are well conserved through evolution, and ii) the bacteria evolved different regulatory components for the expression of those genes probably for specific bacterial adaptation to its host and/or niche.

Regarding the deep branches of our tree, no common topology can be found with the bacterial taxonomy, some sub-trees appearing more correlated with ecological niches, as illustrated for marine bacteria (Figure 5, group V), plant pathogens/symbionts (Figure 5, group IV) or animal pathogens. In addition, many observations favour the hypothesis that T6SS may be acquired by horizontal transfer as they belong to genomic islands gained by such a mechanism. This has been reported for: i) *Helicobacter hepaticus *where the T6SS gene cluster belongs to the large HHGI1 genomic island that has been shown to be required for the induction of liver disease [29], ii) *Photobacterium profundum *where the genes coding for T6SS are found in region chr1.3 which is probably horizontally acquired and absent from strain 3TCK of *P. profundum *[30], iii) the uropathogenic *Escherichia coli *O6 strain UPEC/O6:H1/ATCC 700928/CFT073, where one of the two T6SS gene clusters is encoded in the genomic island PAI-CFT073-metV [31], and iv) the enterohaemorrhagic *E. coli *O157:H7 where it is located in the genomic island OI#7 [32] whereas no T6SS footprint was found in the non pathogenic *E. coli *K12.

### Is FPI a true T6SS?

In two recent papers, Nano and coworkers [20,33] argued that the FPI (*Francisella *pathogenicity island) locus might encode a T6SS. IglA and IglB were shown to interact, and IglA was found as crucial for intracellular growth of *Francisella*. The same authors also identified an FPI encoded protein that presents similarity to members of the DotU protein family and another FPI encoded protein with a part of the conserved domain associated with the IcmF protein family. Intriguingly, the phylogenetic analysis of Bingle and coworkers [14], based exclusively on IglA and IglB sequences, showed that *Francisella *sequences clustered together in a deep branch of the tree, and our approach did not identify any T6SS locus in *Francisella*. In order to investigate this discrepancy, each gene product of the FPI was used as query with the ***psiblast ***program against the NCBI non-redundant protein databank. For each protein, functional domain detection was achieved through an ***rpsblast ***search [34] against the COG database. The complete results are summarized in a supplementary Table [see Additional file 4]. Ten out of the 18 proteins show no similarity with any sequence of the databank, except with FPI encoded proteins from other strains of *Francisella*. Among the remaining proteins, only three exhibit significant sequence similarities to T6SS core genes: IglA, IglB and PigF, an homolog of MotB/OmpA/DotU. We also confirmed that the PdpB sequence presents a similarity in its C-terminal region with the C-terminal fragment of the COG3523 (IcmF). We were unable to find any protein showing significant similarity with the T6SS dependent secreted protein, neither Hcp nor Vgr in all available *Francisella *genomes.

These results suggest that the FPI would not encode a T6SS similar to the recently described T6SS but may rather encode another system implied in bacterial virulence that shares some components with both T6SS and T4SS. Ongoing functional and structural studies on orphan proteins encoded in the FPI (like the recently resolved structure of IglC [35]), and the determination of the macromolecular complex encoded by this island should help to better classify and understand this putative secretion system.

### Focus on VgrG proteins

In addition to secreted Hcp whose function is still unclear, T6SS export a class of VgrG proteins. VgrG proteins have been proposed to bind/puncture target eukaryotic cells in order to deliver effector domains encoded within their C-terminal regions [6]. To further examine this hypothesis, we looked for conserved features and/or domains in the C-terminal regions of the VgrG proteins identified in all the T6SS loci reported above.

Full length T6SS encoded VgrG proteins were selected according to the ***rpsblast ***analysis performed previously (see Materials and methods §1): only proteins showing an alignment covering at least 80% of the COG3501 PSSM with an E-value ≤ 10^-6 ^were retained. We ended up with a set of 180 proteins with length ranging from 529 to 1207 amino acids. As previously reported and suggested by their nomenclature Val-Gly-Repeats [36], the VgrG proteins were already known for containing repeats in their C-terminal part. Systematic search for short length amino acid tandem repeats in C-terminal regions identified 31 VgrG sequences having such repeats [see Additional file 5]. Their sequences were compared both at the DNA and amino acid levels. When reported on the super-tree built on the T6SS loci, these proteins belongs to two distinct groups, except for two of them (one from *Pseudomonas aeruginosa *and one from *Bordetella bronchiseptica*) that remained un-clustered [see Additional file 6]. The first group is characterized by VgrG sequences whose repeats are highly conserved both at the protein and DNA levels, implying that they are recent. The second group corresponds to VgrG repeats that are no more detectable at the DNA level, but with a period and some amino acids strongly conserved at specific positions, suggesting that a selective pressure is acting in order to keep these repeated motifs functional. The VgrG sequences belonging to the two un-clustered loci present the same characteristics than the ones of the second group. All the species harbouring such VgrG proteins are animal pathogens. It strongly suggests that these repeats may be involved in virulence.

In the 149 remaining proteins, an ***rpsblast ***search was performed on their C-terminal regions and hits with an E-value ≤ 10^-3 ^were retained. Similarity with the COG3409 ('Putative peptidoglycan-binding domain-containing protein'), already reported for VC_A0123 of *V. Cholera *[6], was not found in other VgrG proteins. On the other hand, we found significant similarities between the COG4253 ('unknown function') and the C-terminal regions of fifty VgrG proteins, all issued from beta or gamma Proteobacteria. Among them, the two sequences from the two *Burkholderia cenocepacia *available strains present a partial additional hit in their C-terminal part with the COG3846 (TrbL/VirB6), known to be a component of the T4SS. The C-terminal regions of the remaining 98 VgrG proteins do not contain any identified conserved domain, but share, for some of them, sequence similarities. Therefore, it appears that the VgrG proteins found in T6SS loci are mainly characterized by the presence, in their C-terminal part, of either repeats or COG4253 functional domain. Indeed, more than one third of the VgrG proteins possess one of these two features. These two domains have been predicted by Pukatzki and coworkers as being structurally similar to putative adhesive molecules fibronectin-, YidB-, YadA- or tropomyosin-like [6]. A detailed manual sequence comparison of eight VgrG proteins listed in their publication showed that four of them contained either repeats or COG4253 at their C-terminal ends.

In conclusion, as VgrG proteins are suspected to be in contact with host cells, these C-terminal domains (repeats and COG4253) may be involved in host cell adhesion or virulence. However, their role in secretion, translocation or virulence needs to be experimentally documented.

## Conclusion

Our screening approach has revealed the presence of complete T6SS gene clusters in more than 80 bacterial genomes. Despite the increasing number of sequenced genomes, this secretion system appears to be confined to Proteobacteria. We have clearly identified a core of 13 genes highly conserved and specific of T6SS. Through a phylogenetic reconstruction of the T6SS loci and the analysis of phylogenetic profiles, subtypes of T6SS have been defined. They appear to have evolved different regulatory mechanisms necessary for bacterial adaptation to specific environments and/or hosts.

The comparative genomic approach highlights a highly conserved genomic organization of three groups of genes among the 13 core T6SS genes, and suggests that the proteins they encode may physically interact or form functionally important complexes. The first group (COG3521, COG3522, COG3455 and COG3523) might constitute the membrane component of the basal secretion apparatus. The second group, composed of COG3516 (IglA), COG3517 (IglB) and COG3157 (Hcp), may direct a more efficient processing of unfolded proteins – in particular unfolded and unassembled Hcp proteins – by the chaperon-like proteins IglA-IglB. Indeed, the last group contains three proteins (COG3518, COG3519 and COG3520) of unknown function that have been proposed to be part of the secretion apparatus [9]. One of its members (COG3518) has similarities with the phage gp25 protein. It has been suggested that gp25 and gp27 are in close proximity in the baseplate and may physically interact [4]. As the N-terminal domain of VgrG is similar to gp27, both domains may also interact in T6SS.

Among the 13 core proteins, the secreted VgrG proteins are suspected to carry effector domains at their C-terminal end. We found that VgrG proteins are mainly characterized by the presence of repeats or of the functional domain COG4253 of unknown function that have been suggested to promotes adhesion to host cell [6].

One striking feature of this secretion system is that multiple copies of almost complete loci are found in about one third of the genomes harbouring such a system. Our phylogenic tree reveals that they do not result from recent duplication events and have been conserved through evolution, suggesting a sustained and constrained mechanism that favoured this trend. On one hand, the high number of T6SS gene clusters found in various pathogenic bacteria (*Burkholderia*, *Yersinia*, *Pseudomonas*) may be explained by a specialization of the secretion system by means of regulatory mechanisms and targets. On the other hand, the presence of T6SS clusters in non-pathogenic bacteria suggests that T6SS involvement is not limited to virulence, but that it may also be implicated in functions such as host/symbiont communication, exchange and cell-cell communication. For instance, in *Acinetobacter baylyi *ADP1, the knock-out of T6SS genes display a strong phenotype on colony growth depending on various carbon sources, suggesting a putative role of T6SS in a cascade of events leading, when not functional, to membrane or cell adhesion disorders [37].

No consensual model for the assembly of the Type VI Secretion machinery has emerged yet ([38,39]). These divergences emphasize the need for further experiments to get a better characterization of each T6SS components as well as defining the protein complexes building up the system. Our results bring new functional hypothesis that could be tested experimentally and enrich our knowledge on this poorly understood secretion system.

## Methods

### Building T6SS protein coding gene core

Protein sequences from genomic fragments listed in Table 1 were extracted and aligned against the Position Scoring Specific Matrices (PSSMs) of the COG section [16] from the Conserved Domain Database (CDD) [40]. Alignments were performed with the ***rpsblast ***program and each of them was manually checked to assess their consistency.

The genomic fragments and their annotations were either downloaded from the EMBL nucleotide databank (for *Rhizobium leguminosarum*, *Edwardsiella tarda *and *Salmonella enterica*) or extracted from the Genome Reviews files when the complete genome was available (*Pseudomonas aeruginosa*, *Vibrio cholerae*, *Burkholderia pseudomallei*, *Burkholderia mallei *and *Aeromonas hydrophila*). File identifiers and genomic locations are reported in Table 1.

### Genome wide mapping of T6SS

Annotated genomes were downloaded from the Genome Reviews [41] ftp site (, September 2007, 461 bacterial genomes, 40 archaeal genomes and 5 eukaryotic genomes for a total of 976 replicons). Protein sequences from all complete annotated genomes were aligned with ***rpsblast ***against the COGs of the CDD. Only proteins showing an alignment covering at least 80% of the COG PSSM with an E-value ≤ 10^-6 ^were retained. To avoid any errors in COG assignments, we discarded all hits that overlap another hit with a better E-value on more than 50% of its length. To delineate genomic localization of genes encoding T6SS components, we clustered contiguous protein coding genes showing similarity with the COGs listed in Table 1. Two genes were considered as contiguous if they are separated by less than five non-related T6SS genes. Clusters containing at least five genes encoding different putative T6SS proteins (different COGs) were conserved. A threshold of five genes was chosen, as it allows to recover incomplete T6SS without selecting the icmf/dotU region involved in T4SS in *Legionella pneumophila *(for a description of type IV-B secretion system see [42]). These first selected genomic regions were then extended by taking into account five kilo-bases on each side in order to detect putative conserved genes not yet known to be associated with T6SS (description of all identified loci are available as supplementary material [see Additional file 7]). All selected proteins were tested for the presence of a signal peptide and transmembrane helices with ***SignalP ***[43] and ***TMHMM ***[44] respectively.

### T6SS core gene frequencies and taxonomic distribution

To compute the COG frequencies, all T6SS regions were assigned the same weight, *i.e*., a COG present in two different T6SS loci encoded within the same replicon is counted twice. On the other hand, if a COG was encoded by more than one gene in a same T6SS locus (in case of gene duplication for example), it was just counted once (present/absent). In order to evaluate the taxonomic distribution, we used the phylogenetic classification provided within the genome review files. To avoid any bias due to redundancy caused by sequencing of different strains of the same species, only one strain harbouring at least one predicted T6SS was kept as reference. (strains containing T6SS loci but not considered in frequency computations are listed in a supplementary table [see Additional file 8]).

### Phylogenetic profiles

Phylogenetic profiles (PP) were elaborated by two approaches.

a. In the first approach, the presence of proteins similar to each previously selected COG is searched in all the genomes under study. For each COG, a boolean vector is constructed whose size is equal to the number of T6SS loci identified previously. Values are either '1' (presence of at least one occurrence of this COG in the locus) or '0' (absence of this COG in the locus). This vector defines a first type of phylogenetic profile.

b. The second approach relies on the ***TribeMCL ***strategy [27]. The procedure starts with all-against-all ***blastp ***comparisons of a set of proteins of interest. An E-value cut off of 10^-6 ^was chosen for retaining similarities. Then, protein relationships are converted into a graph in which the nodes represent protein sequences, and the weighted edges represent their relationships. The weights are computed as the -log_10_(E-value) of the ***blastp ***results for each pair of sequences. The graph is further processed by a graph-clustering step called Markov Clustering (MCL algorithm). An important parameter in the MCL algorithm is the inflation value, regulating the cluster granularity. We set the inflation value at 2. Each protein cluster is transformed into a boolean vector whose length is equal to the number of T6SS loci identified previously. For each locus that encodes a protein belonging to the cluster, the vector value receives '1' (presence), otherwise it is assigned to '0' (abscence). This vector corresponds to a second type of phylogenetic profile.

For both sets of profiles, distances between two PPs were computed using the Jaccard distance. Mean linkage hierarchical clustering was applied to the distance matrix to identify clusters of PPs that follow the same locus distribution. Complete results are available as supplementary material [see Additional file 3].

### Phylogenetic tree construction

For each of the 13 most conserved COG groups, one maximum likelihood tree with 100 bootstrap replicates was built. Sequences were multi-aligned with ***Muscle ***[45] and trees were computed with ***PhyML ***[46] using the WAG amino acid substitution model of evolution [47] and four categories of substitution rates. Similarities between trees were computed using ***TOPD/FMTS ***with the split distance (a low split distance value is synonymous of a high number of common branches between the two trees) [48]. Mean linkage hierarchical clustering was applied on these distances in order to check the homogeneity of trees. The super-tree was built by applying the Weighted Matrix Representation with Parsimony (W-MRP) method [24]. The W-MRP method creates a matrix whose characters refer to the topologies of the sources trees. The rows of the matrix correspond to the leaves (taxa) of input trees and each column represents one internal branch in one source tree, so the number of columns of the matrix is equal to the total number of internal branches across all the source trees. The method works by examining each internal branch of each input tree, and assigning a '1' to any taxa contained within the clade defined by that internal branch. A '0' is assigned to any taxon present in the source tree but not included in the clade, and a '?' is assigned to any taxon absent from the source tree. To consider gene duplications, '?' is allocated to any taxon whose genes are found on both sides of the internal branch. For matrix computation, only internal branches with a boostrap support higher than 50 has been considered. To enhance the contribution, in the super-tree construction, of the highly supported branches of source trees, the columns are weighted as follow: each internal branch is represented by a number *p *of columns, with *p *equal to 1 when the boostrap value is 50 and *p *is incremented by 1 for each increase of 10 of the boostrap value. To recover the consensus tree, the Maximum Parsimony method has been applied with 100 boostrap replicates (***phylip ***package, ).

## Authors' contributions

FB, GF, YV and IA conceived the study and designed the experiments. FB and JB implemented the algorithms and performed the experiments. FB, GF, IA and YV drafted the manuscript. All authors read and approved the final manuscript.

## Supplementary Material

Additional file 1 **Mean linkage hierarchical clustering of the 13 trees obtained with the most conserved COG groups. **Result of the mean linkage hierarchical clustering applied to the split distances computed with ***TOPD/FMTS ***[23] on the 13 trees obtained with the most conserved COG groups. Red colours indicate value higher than the mean. Blue colours reveal value lower than the mean. Click here for file

Additional file 2 **List of bacterial species containing multiple T6SS gene clusters.** Table containing the numbers of T6SS gene clusters predicted in species encoding more than one T6SS. Click here for file

Additional file 3 **Relationship between phylogeny and T6SS gene content.** Rows represent T6SS loci and columns represent protein functional classes (based on COG assignment and ***TribeMCL ***protein clusters). The tree on the left is the consensus phylogenetic tree (super-tree, see Materials and Methods §5, manually rooted) whereas the two upper dendrograms represent a hierarchical clustering of the two sets of phylogenetic profiles. Core T6SS conserved proteins are depicted with the same color code as in Figure 4. Letters in front of bacteria names correspond to the four groups proposed by Bingle and co-workers [14], '#' marks functional T6SS loci depicted in Table 1 and included in this figure. Click here for file

Additional file 4 **Sequence similarities for for the FPI encoded proteins. **Details of sequence similarities detected for the FPI encoded proteins against the NCBI non-redundant databank and the Conserved Domain Database. Click here for file

Additional file 5 **Repeats identified in VgrG C-terminal regions. **Proteic sequences of the VgrG proteins and the repeats identified in their C-terminal regions. Click here for file

Additional file 6 **Relationship between the inferred phylogeny for the T6SS loci and their VgrG content. **Each row represents a T6SS locus. Boxes represent VgrG proteins. Inside the boxes, each rectangle indicates a COG hit. Colours indicate the nature of the COG hit: COG3501 (purple), COG4253 (blue) or other COGs (green). VgrG that contain C-terminal repeats are striped. Note that VgrG of *Aeromonas Samonicida *(CP000644A) has not been included as it is disrupted by an insertion element. Click here for file

Additional file 7 **Detailed description of all identified T6SS gene clusters.** Archive containing the detailed description of each identified T6SS locus as an HTML file. Click here for file

Additional file 8 **Bacterial strains not taken into account for the computations of genes frequencies and gene neighbourhood frequencies.** List of bacterial strains not taken into account for the computations of genes frequencies and gene neighbourhood frequencies. Click here for file
