# Supplementary figures and images for: Dissecting the bacterial type VI secretion system by a genome wide in silico analysis: what can be learned from available microbial genomic resources?
Source: BMC Genomics. 2009 Mar 12;10:104. doi: 10.1186/1471-2164-10-104 (PMC2660368; doi:10.1186/1471-2164-10-104)

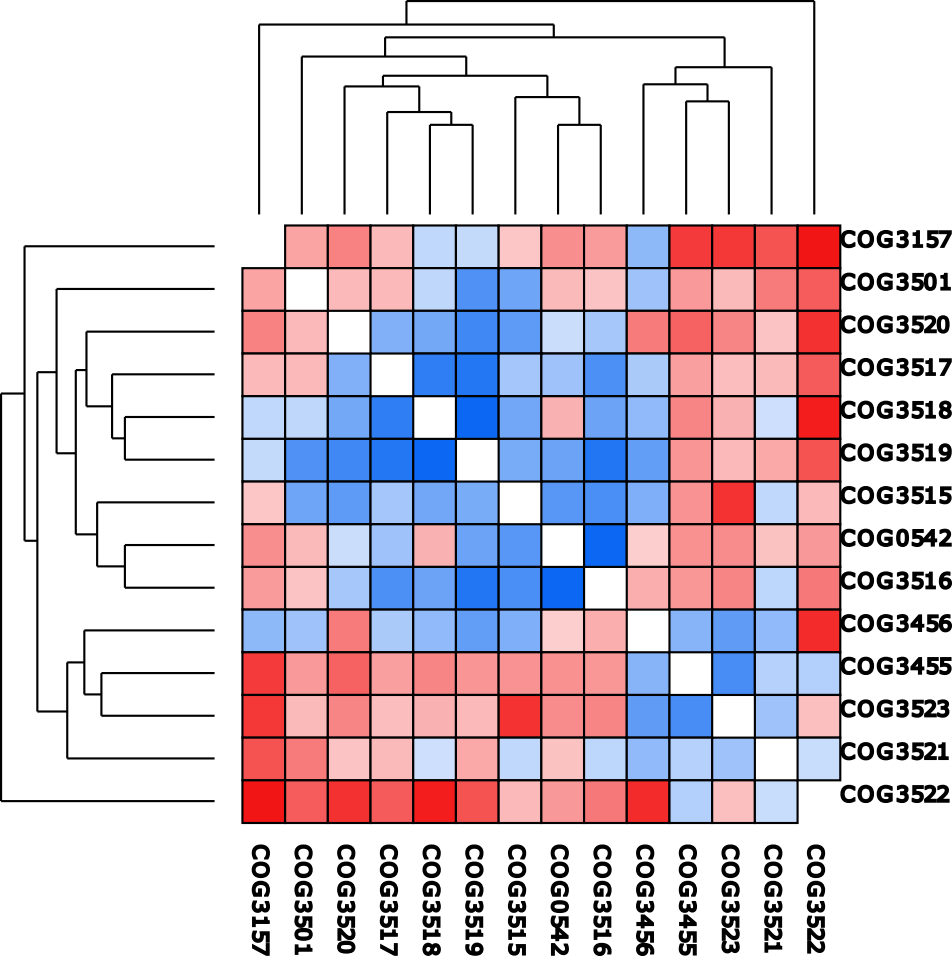

Supplement: Additional file 1 — Mean linkage hierarchical clustering of the 13 trees obtained with the most conserved COG groups. Result of the mean linkage hierarchical clustering applied to the split distances computed with TOPD/FMTS [23] on the 13 trees obtained with the most conserved COG groups. Red colours indicate value higher than the mean. Blue colours reveal value lower than the mean. [file 1471-2164-10-104-S1.pdf]

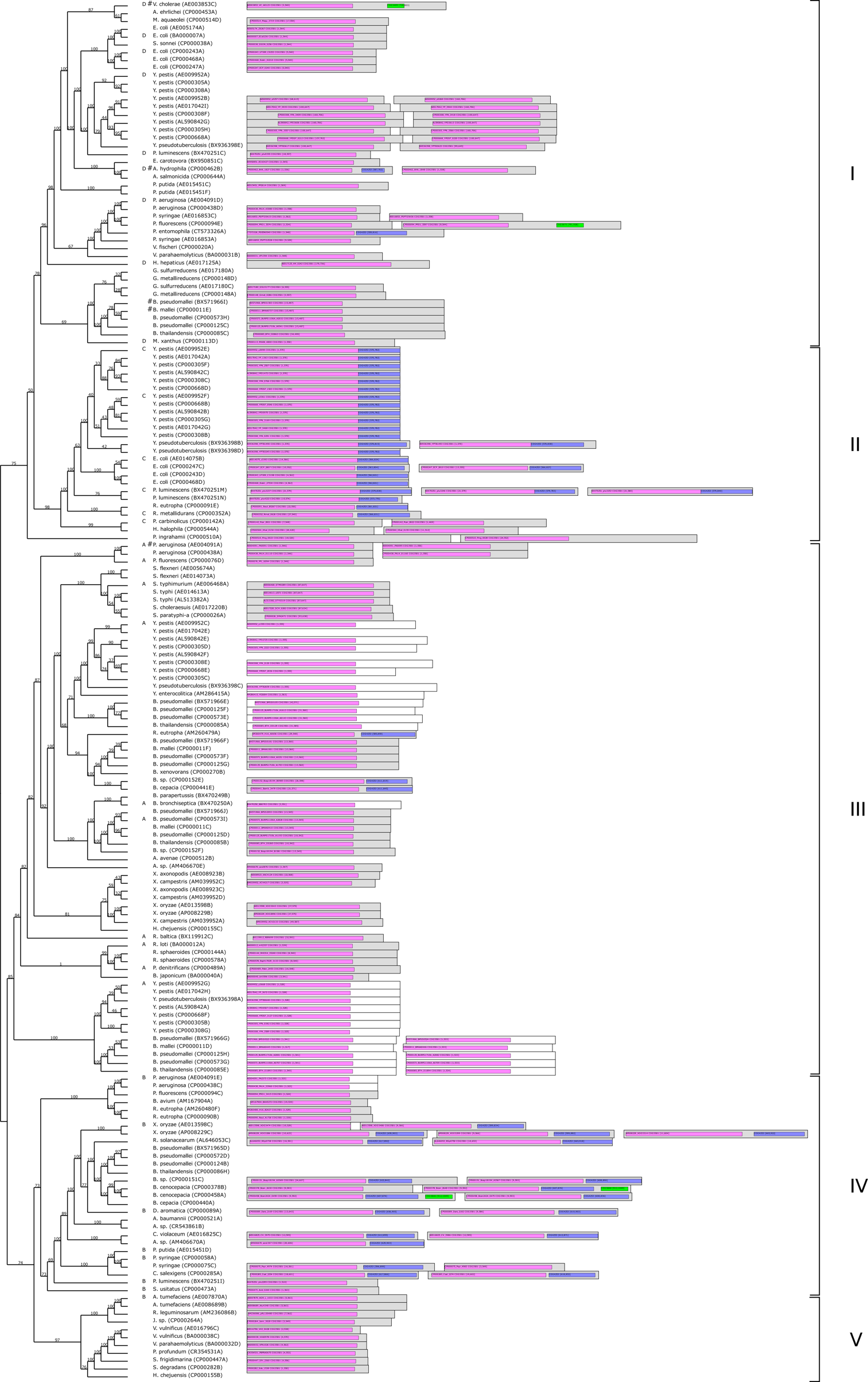

Supplement: Additional file 6 — Relationship between the inferred phylogeny for the T6SS loci and their VgrG content. Each row represents a T6SS locus. Boxes represent VgrG proteins. Inside the boxes, each rectangle indicates a COG hit. Colours indicate the nature of the COG hit: COG3501 (purple), COG4253 (blue) or other COGs (green). VgrG that contain C-terminal repeats are striped. Note that VgrG of Aeromonas Samonicida (CP000644A) has not been included as it is disrupted by an insertion element. [file 1471-2164-10-104-S6.pdf]
