# Supplementary material for: Dissecting the bacterial type VI secretion system by a genome wide in silico analysis: what can be learned from available microbial genomic resources?
Source: BMC Genomics. 2009 Mar 12;10:104. doi: 10.1186/1471-2164-10-104 (PMC2660368; doi:10.1186/1471-2164-10-104)
Supplement: Additional file 2 — List of bacterial species containing multiple T6SS gene clusters. Table containing the numbers of T6SS gene clusters predicted in species encoding more than one T6SS. [file 1471-2164-10-104-S2.doc]

| Yersinia pestis (biovar Antiqua Nepal516, strain Nepal516) | 6* |
| --- | --- |
| Yersinia pestis (biovar Mediaevalis, strain KIM5) | 6 |
| Yersinia pestis (biovar Orientalis, strain CO-92) | 6 |
| Yersinia pestis (biovar Antiqua Antiqua, strain Antiqua) | 6 |
| Burkholderia pseudomallei (strain 1710b) | 6 |
| Burkholderia pseudomallei (strain 1106a) | 6 |
| Burkholderia pseudomallei (strain K96243) | 6 |
| Yersinia pestis (biovar Mediaevalis, strain 91001) | 5 |
| Yersinia pseudotuberculosis (serovar I, strain IP32953) | 5 |
| Yersinia pestis (strain Pestoides F) | 5 |
| Burkholderia thailandensis (strain E264 / ATCC 700388 / DSM 13276 / CIP | 5 |
| Burkholderia mallei (strain ATCC 23344) | 4 |
| Photorhabdus luminescens laumondii (strain TT01) | 4 |
| Burkholderia sp. (strain ATCC 17760 / NCIB 9086 / R18194 / 383) / 383) | 3 |
| Pseudomonas aeruginosa (strain LMG 12228 / ATCC 15692 / PRS 101 / 1C / | 3 |
| Pseudomonas putida (strain KT2440) | 3 |
| Pseudomonas aeruginosa (strain UCBPP-PA14) | 3 |
| Xanthomonas campestris (pathovar vesicatoria, strain 85-10) | 2* |
| Xanthomonas oryzae oryzae (strain KXO85 / KACC10331) | 2 |
| Xanthomonas oryzae (pathovar oryzae, strain MAFF 311018) | 2 |
| Burkholderia cepacia (strain ATCC 53795 / AMMD) | 2 |
| Ralstonia eutropha (strain ATCC 17699 / H16 / DSM 428 / Stanier 337) | 2 |
| Ralstonia eutropha (strain JMP134) | 2 |
| Pseudomonas syringae tomato (strain DC3000) | 2 |
| Pseudomonas fluorescens (strain PfO-1) | 2 |
| Vibrio parahaemolyticus (serovar O3:K6, strain RIMD 2210633) | 2 |
| Escherichia coli O1:K1 / APEC | 2 |
| Escherichia coli (strain UTI89 / UPEC) | 2 |
| Escherichia coli O6:K15:H31 (strain 536 / UPEC) | 2 |
| Azoarcus sp. (strain BH72) | 2 |

* one copy split into two loci
