# Supplementary material for: Dissecting the bacterial type VI secretion system by a genome wide in silico analysis: what can be learned from available microbial genomic resources?
Source: BMC Genomics. 2009 Mar 12;10:104. doi: 10.1186/1471-2164-10-104 (PMC2660368; doi:10.1186/1471-2164-10-104)
Supplement: Additional file 4 — Sequence similarities for for the FPI encoded proteins. Details of sequence similarities detected for the FPI encoded proteins against the NCBI non-redundant databank and the Conserved Domain Database. [file 1471-2164-10-104-S4.doc]

| Gene locus tag | Gene name | Domains | Psi-Blast |
| --- | --- | --- | --- |
| FTT1699 | pdpA | - | - |
| FFT1700 | pdpB | Cter-Cter COG3523 (IcmF) (E-value = 2e-06) | - |
| FTT1701 |  |  |  |
|  | pigA | - | - |
| FTT1702 | pigB | - | Low similarity (E-value = 0.049) to Rhs element Vgr protein |
| FTT1703 | pigC | - | - |
| FTT1704 | pigD | - | Similar to putative uncharacterized proteins in many proteobacteria including *Pseudomonas aeruginosa* (PA2375 HSI-III encoded, E-value = 5e-34) |
| FTT1705 | pigE | - | - |
| FFT1706 | pigF | Partial COG3455 (ompA/motB/dotU) | Similar to T6SS encoded ompA/motB/dotU proteins |
| FFT1707 | pigG | - | - |
| FFT1708 | pigH | - | - |
| FFT1709 | pdpC | - | - |
| FFT1710 | pigI | - | - |
| FFT1711c | iglD | - | Low similarity (E-value = 0.090) to a *Pseudoalteromonas tunicata* D2 protein associated to COG3522 |
| FFT1712c | iglC | - | - |
| FFT1713c | iglB | COG3517 (iglB) | Similar to iglB SST6 encoded proteins |
| FFT1714c | iglA | COG3516 (iglA) | Similar to iglA SST6 encoded proteins |
| FFT1715c | pdpD | - | - |
| FFT1718c |  | Nter-COG3415, Cter-Partial Nter COG3335 (Both Transposase and inactivated derivatives), | Similar to a large number of transposases |
