# Supplementary material for: Dissecting the bacterial type VI secretion system by a genome wide in silico analysis: what can be learned from available microbial genomic resources?
Source: BMC Genomics. 2009 Mar 12;10:104. doi: 10.1186/1471-2164-10-104 (PMC2660368; doi:10.1186/1471-2164-10-104)
Supplement: Additional file 5 — Repeats identified in VgrG C-terminal regions. Proteic sequences of the VgrG proteins and the repeats identified in their C-terminal regions. [file 1471-2164-10-104-S5.pdf]

CP000011_BMAA0446 True 762 CP000011D CP000011_GR []
ORG Burkholderia mallei (strain ATCC 23344)
>CP000011_BMAA0446
MRLIELRSPLLDPDAVALSFVVHENLSQEPSYQLDLLSHDSNLDFDALLG
STLSADIDLGEGDIRTFNTHVFGGYDTGQMSGQYTYTLELRSWLSFLAEN
RNSRIFQDLSVPQIVEQVFQGHQRNGYRFELEGTYEPREYCVQFQETDLN
FVKRLLEDEGIYFWVEHEPDRHVVVISDTQRFEDLPLPNDTLEYLPDGEE
SRAIQGREGVQRLQRTRRIKSNNVALRDFDYHAPSKQLDSDAQVEQQSLG
GIPLEYYDYAAGYRDPEQGERLARLRLEAIQADAHALGGEANARALAVGR
AFTLVGHPALSRNRRYYVTNSELTFIQDGPDSTSQGRNVAVKFRALADDQ
PFRPLLVTKRPRVPGIQSATVVGPEMSEVHTDKLGRIRVHFHWDRYKTTE
ADASCWIRVTQAWAGKGWGVLAMPRVGQEVIVVYVDGDLDRPLATGIVYN
GENPTPYDLPKDIRYTGLVTRSIKRAGGIPNASQLTFDDQHGAERVMIHA
ERDLQQTVERNSSTSIAQDLNLSV
KGTSTSV
VGISVSF
TGISVSY
TGLSVSF
TGVSARF
TGVSTSF
TGVSTSF
TGVSTSF
TGVDTSF
TGVSTGF
KGVDTSF
TGVATSM
VGVSTSI
TGSSNSV
TGVSNSM
TGISSSW
KDVSMST
TGQSESI
TGVSLSY
TGTSNSM
TGTSTSV
TGTSTSI
TGTSMSN
TGSSTSI
TGTSMST
TGSSVST
TGSSMSA
TGSSVGT
TGSSVST
TGSKMSV
TGFSFSY
TGASYED
VGVDLKK
LGMQTKN

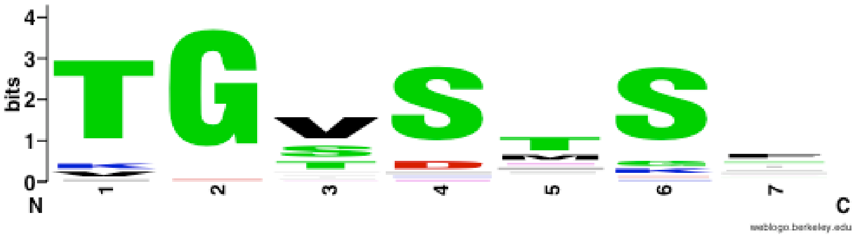

CP000438_PA14_33960 True 668 CP000438C CP000438_GR []
ORG Pseudomonas aeruginosa (strain UCBPP-PA14)
>CP000438_PA14_33960
MPRPTDSNTSLSLTASALAALFPERLAGEERLNALPGWELLGYSASPLAL
DGAIATHLTATLHNDADQRPLDGLVAEIRQLPGDASAERYQVLLRPWLWW
LTLASNNRVFQNLATSDIVTQVFDQHGFSDYQLQLSGSYQPREYCVQYGE
SDLAFVSRLLEEDGIFWFFTHAAGKHTLVLADSNDAFPPIPNGPQVAYLG
QGIGVRELQGVRSAQYSLQAVSGTYSATDYEFTTPSTSLYSQAEAVSGAA
GVYQHPGGYTAKAQGDSLTKQRIDGLRSQETRLIGESDCRWLVPGHWFTL
SGHDDDSLNIDWVLTSVTHDASHAHYRNRFEAIPKATAYRPARVTPKPRM
HTQTALVVGKAGEEIWTDQYGRIKIQFPWDRDGKNDETSSCWVRVVLPWS
GKGFGMQFVPRIGQEVIVTFIDGDPDRPLVTGCVYNGDNALPYALPDNQT
QSGIKTNSSKGGGGFNELRFEDKKDAEEVFLQAQKDLNVNVLNDSTASIG
HDETLTVQNARTRTVKEGDETVTLEKGKRSVTIQTGSDSLDVKDTRTVTV
GADQTHSTGGNYSHKVSGDFELTVD
GNLTIKVS
GTLALQSG
GSLTLKSD
ADLAAQAG
TSLTSKAG
TSLTNQAG
TSLTNKAG
TSLTNDAG
VSLTNKAG
AEQTVDGG
GMLTIKGG
LVKVN

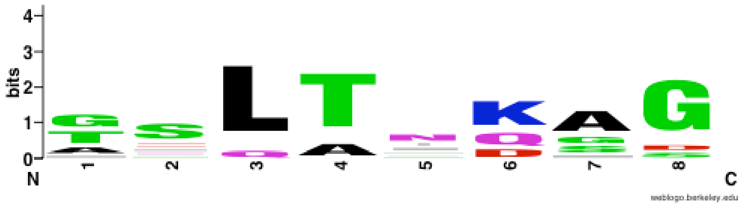

CP000011_BMAA0445 True 748 CP000011D CP000011_GR []
ORG Burkholderia mallei (strain ATCC 23344)
>CP000011_BMAA0445
MRLIELRSPLLDPDAVALSFVVHESLSQEPSYQLDLLSHDPDLDFDALFG
STLSADIDLGEGDIRTFNTHVFGGYDTGQMSGQYTYTLELRSWLSFLAEN
RNSRIFQNMSVPQIVEQVFQGHQRNGYRFELEGTYEPREYCVQFQETDLN
FVKRLLEDEGIYFWVEHEPDRHVVVISDTQRFEDLPLPNDTLEYLPDGEE
SRAIQGREGVQRLQRTRRIKSNNVALRDFDYHAPSNKLDSDAQQVSPPNL
EGIPLEYYDYAAGYREPEQGERLARLRLEAIQAESHTLVGEANARALATG
RAFTLIGHPALGRNRRYYVTNSELTFIQDGPDSTSQGRNVAVKFRALADD
QPFRPLLTTPRPEVPGIQSATVVGPEMSEVHTDKLGRIRVHFHWDRYKTT
EADASCWIRVSQAWAGKGWGVIAMPRVGQEVLVTYVDGDLDRPLVTGIVY
NGENPTPYDLPKDIRYTGLVSRSIKRAGGYQNASQITFDDQRGAERVMIH
AERDMQQTVE
RNSSTSV
VGISISF
TGISVSY
TGLSVSF
TGVSASF
TGVSTSF
TGVSTSF
TGVSTSF
TGVDTGF
KGVSTSF
TGVDTSF
KGVSTSF
TGVSTSL
TGSSNSV
TGVSNSM
TGISSSW
TDVSMST
TGQSQSI
TGVSLSY
TGTSNSM
TGTSTSV
TGTSTSI
TGTSMSN
TGSSTSI
TGTSMST
TGSSTSV
TGSSVST
TDSSVST
TGSSVST
TGSSVST
TGFSFSY
TGVSYSD
TGIDLKK
VGMQVKS

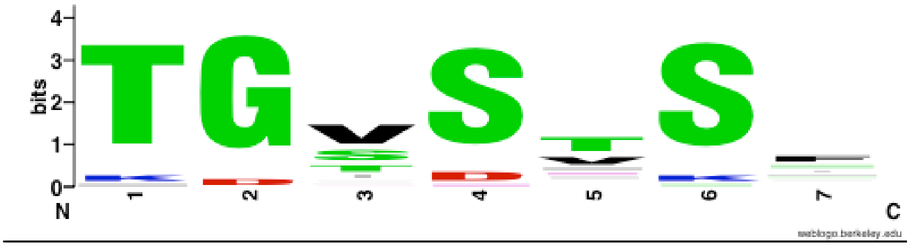

CP000573_BURPS1106A_A0143 True 896 CP000573E CP000573_GR
[]
ORG Burkholderia pseudomallei (strain 1106a)
>CP000573_BURPS1106A_A0143
MPNHFSNGRTNQSRTVVIRSGAMPRLLGQPALEFLSLRGEEHLGKLYTYE
LLLRTPDDFHVPLATSANLDLKAMIGTEMTVCIQLDGIGTGAQGGVGAGA
REISGLVVKAGFLRCEGRYNVYRIELRPWLWLATLTSDYKIFQDKSVVEI
IDTVLHDYPYPVEKRLDIDKYSVAGESARNEPRAFQVQYGETDFDFVQRL
MEEWGIYWFFEHSDNKHRLVLCDHIGGHRKAPSEAYHEIAHHPEGGKIDI
EYINYFSTDEALRPGRVVIDDFDFTRPLASLVTSNHQPRETNWGEGELFE
WPGDYTDSKHGDLISRVRMEERRATGSRAYGRGNVRGLACGHTFVLSKHK
HDGANREYLVIESALMLTEVADETGSGYRYECDNELVVQPSNEVFRMPRE
TPKPTTSGPQSAIVVGPPGHEVWTDEFGRVKIRFLWDRYARNDATDSCWV
RVSQAWAGVNFGGIYIPRIGQEVIVGFMNGDPDRPLILGSLYNTITPPPW
DLPGDATKSGFKSKSITGGRENYNGIRFEDKLGAEEFHMQAEKDMNRLTK
NDESHTVGANFSIGVGLTHTRAVGAMFSSIVGGAASYAVGGAESTMIGGA
YALNVGGAHAVAVGGASSVSVGGAYARNVGGAYALTVGGVLSIVCGASSI
TMTACGSIKIVGKNIRIIGSDEVVVQGAPLQLNPGDSDCGGGGGGGGGGG
AIPPIPLPSFFLDITKPILPPPPPPPTEVPPD
PTPTPTPTPTPTPTPTPTPTPTPTPTPTPTPTPTPTPTPTPTPTPTPTPT
PTPTPTPTPTPTPTPTPTPTPTPTPTPTPTPTPTPTPTPTPTPTPTPTPT
PTPTPTPTPTPTPTPTPTPTPTPTPTPTPTPTPTPTPTPTPTPTPTPTPT
PTPTPTPTPT
SSEI

CP000573_BURPS1106A_A0707 True 763 CP000573G CP000573_GR
[]
ORG Burkholderia pseudomallei (strain 1106a)
>CP000573_BURPS1106A_A0707
MRLIELRSPLLDPDAVALSFVVHENLSQEPSYQLDLLSHDPDLDFDALLG
STLSADIDLGEGDIRTFNTHVFGGYDTGQMSGQYTYTLELRSWLSFLAEN
RNSRIFQNMSVPQIVEQVFQGHQRNGYRFELEGTYEPREYCVQFQETDLN
FVKRLLEDEGIYFWVEHEPDRHVVVISDTQRFEDLPLPNDTLEYLPDGEE
SRAIQGREGVQRLQRTRRIKSNNVALRDFDYHAPSNKLDSDAQQVSPPNL
EGIPLEYYDYAAGYREPEQGERLARLRLEAIQAESHTLVGEANARALATG
RAFTLIGHPALGRNRRYYVTNSELTFIQDGPDSTSQGRNVAVKFRALVDD
QPFRPLLTTPRPEVPGIQSATVVGPEMSEVHTDKLGRIRVHFHWDRYKTT
EADASCWIRVSQAWAGKGWGVIAMPRVGQEVLVTYVDGDLDRPLVTGIVY
NGENPTPYDLPKDIRYTGLVSRSIKRAGGYQNASQITFDDQRGAERVMIH
AERDMQQTVERNSSTSIAQDLNLSVKGTSTSVVG
ISISFTG
ISVSYTG
LSVSFTG
VSASFTG
VSTSFTG
VSTSFTG
VSTSFTG
VDTGFKG
VSTSFTG
VDTSFKG
VSTSFTG
VSTSLTG
SSNSVTG
VSNSMTG
ISSSWTD
VSMSTTG
QSQSITG
VSLSYTG
TSNSMTG
TSTSVTG
TSTSITG
TSMSNTG
SSTSITG
TSMSTTG
SSTSVTG
SSVSTTG
SSVSTTG
SSVSTTG
SSVSTTG
FSFSYTG
VSYSDTG
IDLKKVGMQVKS

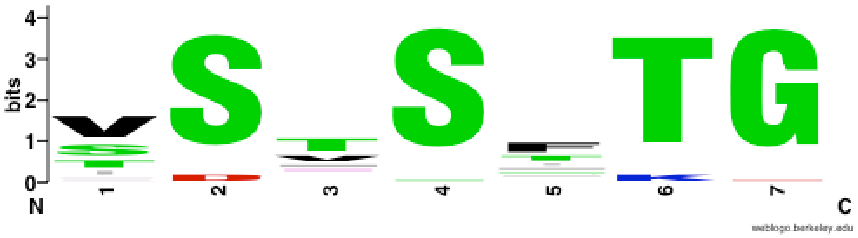

CP000085_BTH_II1894 True 763 CP000085E CP000085_GR []
ORG Burkholderia thailandensis (strain E264 / ATCC 700388 / DSM 13276 / CIP
>CP000085_BTH_II1894
MRLIELRSPLLDPDAVALSFVVHENLSQEPSYQLDLLSHDPDLDFDALLG
STLSADIDLGGGDIRTFNTHVFGGHDTGQMSGQYTYTLELRSWLSFLAEN
RNSRIFQNMSVPQIVEQVFQGHQRNGYRFELEGTYEPREYCVQFQETDLN
FVKRLLEDEGIYFWVEHEPDRHVVVISDTQRFEDLPLPNETLEYLPDGEE
SRAIQGREGVQRLQRTRRIKASNVALRDFDYHAPSNKLDSDAQLVSPPNL
EGIPLEYYDYAAGYREPEQGERLARLRLEAIQADSHMLVGEANARALATG
RAFTLIGHPALGRNRRYYVTNSELTFIQDGPDSTSQGRNVAVKFRALADD
QPYRPLLTTPRPEVPGIQSATVVGPEMSEVHTDKLGRIRVHFHWDRYKTT
EADASCWIRVSQAWAGKGWGVIAMPRVGQEVLITYVDGDLDRPLVTGIVY
NGENPTPYDLPKDIRYTGLVSRSIKRAGGYQNASQITFDDQRGAERVMIH
AERDMQQTVERNSSTSIAQDLNLSV
KGTATSV
VGISVSF
TGISVSY
TGLSVSF
TGVSASF
TGVSTSF
TGVSTSF
TGVSTSF
TGVSTSF
IGVSTSF
TGVDTGF
KGVSTAM
IGVSTSV
VGSSNSV
TGVSNSM
TGISSSW
TDVSMST
TGQSQSM
TGVSLSY
TGTSNSM
TGTSTSV
TGTSTSI
TGTSMSN
TGSSTSI
TGTSMST
TGSSTSI
TGSSVST
TGSSVST
TGSSVST
TGSSVST
TGFSFSY
TGASYSD
VGIDLKKVGMQVKS

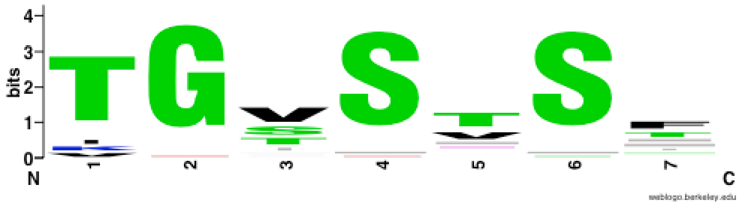

CP000125_BURPS1710b_A2082 True 762 CP000125H CP000125_GR
[]
ORG Burkholderia pseudomallei (strain 1710b)
>CP000125_BURPS1710b_A2082
MRLIELRSPLLDPDAVALSFVVHENLSQEPSYQLDLLSHDSNLDFDALLG
STLSADIDLGEGDIRTFNTHVFGGYDTGQMSGQYTYTLELRSWLSFLAEN
RNSRIFQDLSVPQIVEQVFQGHQRNGYRFELEGTYEPREYCVQFQETDLN
FVKRLLEDEGIYFWVEHEPDRHVVVISDTQRFEDLPLPNDTLEYLPDGEE
SRAIQGREGVQRLQRTRRIKSNNVALRDFDYHAPSKQLDSDAQVEQQSLG
GIPLEYYDYAAGYRDPEQGERLARLRLEAIQADAHALGGEANARALAVGR
AFTLVGHPALSRNRRYYVTNSELTFIQDGPDSTSQGRNVAVKFRALADDQ
PFRPLLVTKRPRVPGIQSATVVGPEMSEVHTDKLGRIRVHFHWDRYKTTE
ADASCWIRVTQAWAGKGWGVLAMPRVGQEVIVVYVDGDLDRPLATGIVYN
GENPTPYDLPKDIRYTGLVTRSIKRAGGIPNASQLTFDDQHGAERVMIHA
ERDLQQTVERNSSTSIAQDLNLSV
KGTSTSV
VGISVSF
TGISVSY
TGLSVSF
TGVSARF
TGVSTSF
TGVSTSF
TGVSTSF
TGVDTSF
TGVSTGF
KGVDTSF
TGVATSM
VGVSTSI
TGSSNSV
TGVSNSM
TGISSSW
KDVSMST
TGQSESI
TGVSLSY
TGTSNSM
TGTSTSV
TGTSTSI
TGTSMSN
TGSSTSI
TGTSMST
TGSSVST
TGSSMSA
TGSSVGT
TGSSVST
TGSKMSV
TGFSFSY
TGASYED
VGVDLKKLGMQTKN

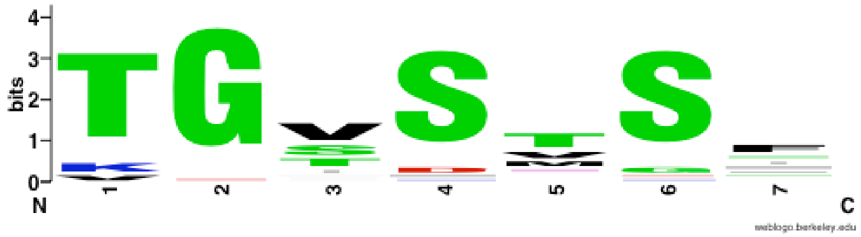

CP000085_BTH_II1893 True 762 CP000085E CP000085_GR []
ORG Burkholderia thailandensis (strain E264 / ATCC 700388 / DSM 13276 / CIP
>CP000085_BTH_II1893
MRLIELRSPLLDPDVVALSFVVHENLSQEPSYQLDLLSRDPNLDFDELLG
STLSADIDLGGGDIRTFNTHVFGGHDTGQMSGQYTYTLELRSWLSFLAEN
RNSRIFQDLSVPQIVEQVFQGHQRNGYRFELEGTYEPREYCVQFQETDLN
FVKRLLEDEGIYFWVEHEPDRHVVVISDTQRFEDLPLPNETLEYLPDGEE
SRAIQGREGVQRLQRTRRIKASNVALRDFDYHAPSKQLDSDAQIEQQSLG
GIPLEYYDYAAGYRDPEQGERLARLRLEAIQADAHALGGEANARALAVGR
AFTLVGHPALSRNRRYYVTNSELTFIQDGPDSTSQGRNVAVKFRALADDR
PYRPLLVTKRPRVPGIQSATVVGPEMSEVHTDKLGRIRVHFHWDRYKTTE
ADASCWIRVTQAWAGKGWGVLAMPRVGQEVIVVYVDGDLDRPLATGIVYN
GENPTPYDLPKDIRYTGLVTRSIKRAGGIPNASQLTFDDQHGAERVMIHA
ERDLQQTVERNSSTSIAQDLNLSVNGTSTSVIGIKVSFTGISVSYTGLSV
SFTGVSASFTGVSTSFTGVSTSFTGVSTSFTGVTTGFTGVSTSFVGVDTS
FTGISTGFVGVSTSI
TGSKNSV
TGVSNSM
TGISSSW
TDVSMST
TGQSQSI
TGVSLSY
TGTSNSM
TGTSTSV
TGTSTSI
TGTSMSN
TGSSTSI
TGTSMST
TGSSVGT
TGSSMSA
TGSSVST
TGSSVST
TGSSMSV
TGFSFSY
IGASYSD
VGIDLKKLGMQTKN

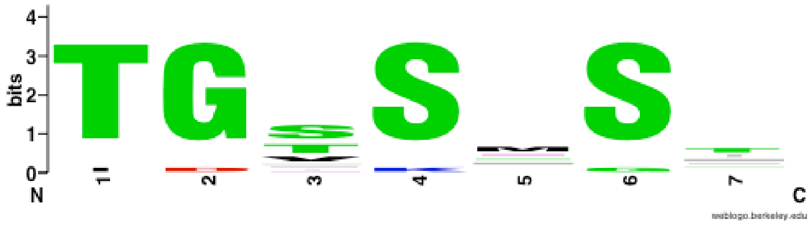

AL590842_YPO2725 True 921 AL590842E AL590842_GR []
ORG Yersinia pestis (biovar Orientalis, strain CO-92)
>AL590842_YPO2725
MSRTINLSSPAMPYLLGEPALVLSKLEGEEAFSTLYSYTITAKTPANPLI
PWQAASNVDLKSLIGKEMTIEMELDGNGLGDVRSVGKGTREITGLVERAR
YIGRDANQAIFEITIKPWFYLANLTSDFKIYQNKNVVDIIEEVFSDYNFP
FEKRLATTYPILDFQVQYGETDFNFLQRLMEEWGIYWFFEHQDHKQKLIL
VDHVGAHKRSFSTAYHAIEYLSDEPKAGEEYITQFQTQETLTSGTWVTND
YDFTKSRADILAMDSKPRKTSFNDLEIYHWPGDYDQPDIGEHLSRVRIEE
RGALGSRAVGSGQLRGIVCGANFELKGFPVDKANREYMIISSRLTVAEVD
QLSGGEDAFSCESSFTVQPTTKIYRHPQVTPKPKTNGPQNAIIVGPPGEE
IWTDEYGRVKVRFVWDRYGTNSESDSCWLRVSQAWAGNSFGGIYIPRIGQ
EVIVDCINGDPDRPMVMGSLYNNVTRPPWDLPANATQSGMVSRTVGGGLT
NYNGVRFEDKSGLEQYWEQAERDMSRLTKNNETQIIGADSVLNVGANRSE
VVGANYNQDVLGTSMLAIGAASMLQVGLARSVVVGGAHSLNVVLANA
TNVGGANM
TNVGGFNG
LAVGGAHQ
TAVGGAAT
LAAGGPIA
IGAGGELI

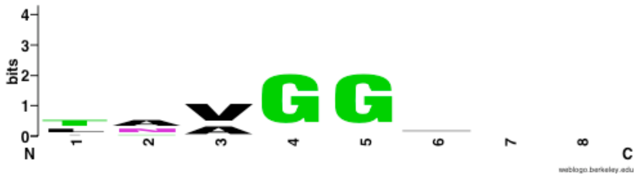

LSGDIVKIVGKSKVIIQGGEVHINSDDCCGRKTGNGGGAFGAFASLAGLL
PLAGAIALPLPLPLLPIV
VPPVTS
PPDPTV
PPDPTL
PPDPTL
PPDPTL
PPETTA
PPETTA
PPETTA
PPETTA
PPETTA
PPETTA
PPETTA
PPETTA
PPETTA
PPETTA
PPETTA
PPETTA
PPETTA
PPETTA
PPETTA
PPETTA
PPETTA
PPETTA
PPETTA
PPETTA
PPETTA
PPETTA

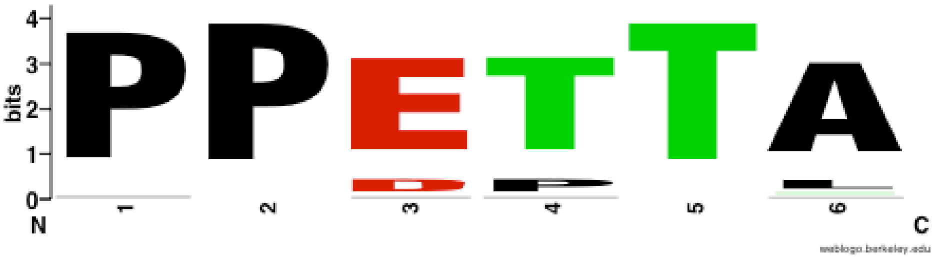

PPETTA
PPETTA
PPETTA
PPETTA
PPEPTRTPPGTQTPPP

CP000668_YPDSF_2036 True 759 CP000668E CP000668_GR []
ORG Yersinia pestis (strain Pestoides F)
>CP000668_YPDSF_2036
MSRTINLSSPAMPYLLGEPALVLSKLEGEEAFSTLYSYTITAKTPANPLI
PWQAASNVDLKSLIGKEMTIEMELDGNGLGDVRSVGKGTREITGLVERAR
YIGRDANQAIFEITIKPWFYLANLTSDFKIYQNKNVVDIIEEVFSDYNFP
FEKRLATTYPILDFQVQYGETDFNFLQRLMEEWGIYWFFEHQDHKQKLIL
VDHVGAHKRSFSTAYHAIEYLSDEPKAGEEYITQFQTQETLTSGTWVTND
YDFTKSRADILAMDSKPRKTSFNDLEIYHWPGDYDQPDIGEHLSRVRIEE
RGALGSRAVGSGQLRGIVCGANFELKGFPVDKANREYMIISSRLTVAEVD
QLSGGEDAFSCESSFTVQPTTKIYRHPQVTPKPKTNGPQNAIIVGPPGEE
IWTDEYGRVKVRFVWDRYGTNSESDSCWLRVSQAWAGNSFGGIYIPRIGQ
EVIVDCINGDPDRPMVMGSLYNNVTRPPWDLPANATQSGMVSRTVGGGLT
NYNGVRFEDKSGLEQYWEQAERDMSRLTKNNETQIIGADSVLNVGANRSE
VVGANYNQDVLGTSMLAI
GAASMLQV
GLARSVVV
GGAHSLNV
VLANATNV
GGANMTNV
GGFNGLAV
GGAHQTAV
GGSATLAA
GGPIAIGA
GGELILSG

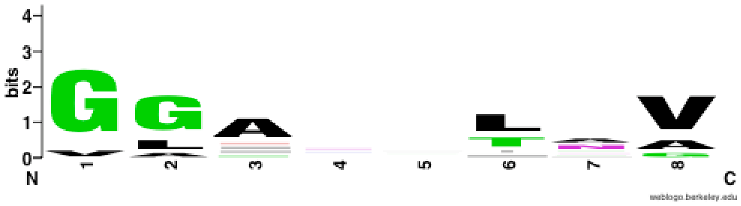

DIVKIVGKSKVIIQGGEVHINSDDCCGRKTGNGGGAFGAFASLAGLLPLAGA
IALPLPLPLLPIVVPPVTS
PPDPTV
PPDPTL
PPDPTL
PPDPTL
PPEPTR

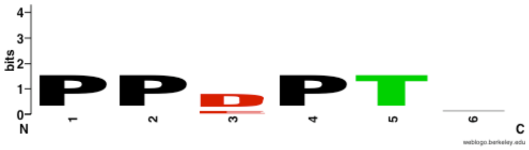

TPPGTQTPPP

CP000125_BURPS1710b_A1613 True 858 CP000125F CP000125_GR
[]
ORG Burkholderia pseudomallei (strain 1710b)
>CP000125_BURPS1710b_A1613
MPNHFSNGRTNQSRTVVIRSGAMPRLLGQPALEFLSLRGEEHLGKLYTYE
LLLRTPDDFHVPLATSANLDLKAMIGTEMTVCIQLDGIGTGAQGGVGAGA
REISGLVVKAGFLRCEGRYNVYRIELRPWLWLATLTSDYKIFQDKSVVEI
IDTVLHDYPYPVEKRLDIDKYSVAGESARNEPRAFQVQYGETDFDFVQRL
MEEWGIYWFFEHSDNKHRLVLCDHIGGHRKAPSEAYHEIAHHPEGGKIDI
EYINYFSTDEALRPGRVVIDDFDFTRPLASLVTSNHQPRETNWGEGELFE
WPGDYTDSKHGDLISRVRMEERRATGSRAYGRGNVRGLACGHTFVLSKHK
HDGANREYLVIESALMLTEVADETGSGYRYECDNELVVQPSNEVFRMPRE
TPKPTTSGPQSAIVVGPPGHEVWTDEFGRVKIRFLWDRYARNDATDSCWV
RVSQAWAGVNFGGIYIPRIGQEVIVGFMNGDPDRPLILGSLYNTITPPPW
DLPGDATKSGFKSKSITGGRENYNGIRFEDKLGAEEFHMQAEKDMNRLTK
NDESHTVGANFSIGVGLTHTRAVGAMFSSIVGGAASYAVGGAESTMIGGA
YALNVGGAHAVAVGGASSVSVGGAYARNVGGAYALTVGGVLSIVCGASSI
TMTACGSIKIVGKNIRIIGSDEVVVQGAPLQLNPGDSDCGGGGGGGGGGG
AIPPIPLPSFFLDITKPILPPPPPPPTEVPPDPTPTPTPTPTPTPTPTPT
PTPTPTPTPTPTPTPTPTPTPTPTPTPTPTPTPTPTPTPTPTPTPTPTPT
PTPTPTPTPTPTPTPTPTPTPTPTPTPTPTPTPTPTPTPTPTPTPTPTPT
PTPTSSEI

CP000308_YPA_2120 True 948 CP000308E CP000308_GR []
ORG Yersinia pestis (biovar Antiqua Antiqua, strain Antiqua)
>CP000308_YPA_2120
MSRTINLSSPAMPYLLGEPALVLSKLEGEEAFSTLYSYTITAKTPANPLI
PWQAASNVDLKSLIGKEMTIEMELDGNGLGDVRSVGKGTREITGLVERAR
YIGRDANQAIFEITIKPWFYLANLTSDFKIYQNKNVVDIIEEVFSDYNFP
FEKRLATTYPILDFQVQYGETDFNFLQRLMEEWGIYWFFEHQDHKQKLIL
VDHVGAHKRSFSTAYHAIEYLSDEPKAGEEYITQFQTQETLTSGTWVTND
YDFTKSRADILAMDSKPRKTSFNDLEIYHWPGDYDQPDIGEHLSRVRIEE
RGALGSRAVGSGQLRGIVCGANFELKGFPVDKANREYMIISSRLTVAEVD
QLSGGEDAFSCESSFTVQPTTKIYRHPQVTPKPKTNGPQNAIIVGPPGEE
IWTDEYGRVKVRFVWDRYGTNSESDSCWLRVSQAWAGNSFGGIYIPRIGQ
EVIVDCINGDPDRPMVMGSLYNNVTRPPWDLPANATQSGMVSRTVGGGLT
NYNGVRFEDKSGLEQYWEQAERDMSRLTKNNETQIIGADSVLNVGANRSE
VVGANYNQDVLGTSMLAIGAASMLQVGLARSVVVGGAHSLNVVLANATNV
GGANMTNVGGFNGLAVGGAHQTAVGGAATLAAGGPIAIGAGGELILSGDI
VKIVGKSKVIIQGGEVHINSDDCCGRKTGNGGGAFGAFASLAGLLPLAGA
IALPLPLPLLPIVVPPVTSPPDPTVPPDPTLPPDPTLPPDPTLPPDPTL
PPETTA
PPETTA
PPRNHG
TAGNHG
TARNNG
TARDNG
TAGNHG
TAGNNG
TARDNG
TARDNG
TARNNG
TAGNNG
TAGNNG
TAGNNG
TAGNNG
TAGNHG
TARDNG
TARDNG
TAGNNG
TARNNG
TAGNNG
TAGNNG
TAGNNG
TAGNNG
TARNNG
TAGNNG
TARNNG
TAGNNG
TARNNG
TARNNGTAGTDANTARDADATALIL

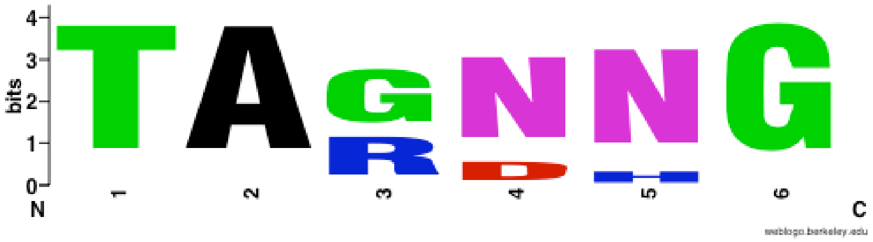

AE009952_y1555 True 861 AE009952C AE009952_GR []
ORG Yersinia pestis (biovar Mediaevalis, strain KIM5)
>AE009952_y1555
MSRTINLSSPAMPYLLGEPALVLSKLEGEEAFSTLYSYTITAKTPANPLI
PWQAASNVDLKSLIGKEMTIEMELDGNGLGDVRSVGKGTREITGLVERAR
YIGRDANQAIFEITIKPWFYLANLTSDFKIYQNKNVVDIIEEVFSDYNFP
FEKRLATTYPILDFQVQYGETDFNFLQRLMEEWGIYWFFEHQDHKQKLIL
VDHVGAHKRSFSTAYHAIEYLSDEPKAGEEYITQFQTQETLTSGTWVTND
YDFTKSRADILAMDSKPRKTSFNDLEIYHWPGDYDQPDIGEHLSRVRIEE
RGALGSRAVGSGQLRGIVCGANFELKGFPVDKANREYMIISSRLTVAEVD
QLSGGEDAFSCESSFTVQPTTKIYRHPQVTPKPKTNGPQNAIIVGPPGEE
IWTDEYGRVKVRFVWDRYGTNSESDSCWLRVSQAWAGNSFGGIYIPRIGQ
EVIVDCINGDPDRPMVMGSLYNNVTRPPWDLPANATQSGMVSRTVGGGLT
NYNGVRFEDKSGLEQYWEQAERDMSRLTKNNETQIIGADSVLNVGANRSE
VVGANYNQDVLGTSMLAIGAASMLQVGLARSVVVGGAHSLNVVLANATNV
GGANMTNVGGFNGLAVGGAHQTAVGGAATLAAGGPIAIGAGGELILSGDI
VKIVGKSKVIIQGGEVHINSDDCCGRKTGNGGGAFGAFASLAGLLPLAGA
IALPLPLPLLPIVVPPVTS
PPDPTV
PPDPTL
PPDPTL
PPDPTL
PPDPTL
PPETTA
PPETTA
PPETTA
PPETTA
PPETTA
PPETTA
PPETTA
PPETTA
PPETTA
PPETTA
PPETTA
PPETTA
PPETTA
PPETTA
PPETTA
PPETTA
PPEPTRTPPGTQTPPP

BX571966_BPSS0524 True 762 BX571966G BX571966_GR []
ORG Burkholderia pseudomallei (strain K96243)
>BX571966_BPSS0524
MRLIELRSPLLDPDAVALSFVVHENLSQEPSYQLDLLSHDSNLDFDALLG
STLSADIDLGEGDIRTFNTHVFGGYDTGQMSGQYTYTLELRSWLSFLAEN
RNSRIFQDLSVPQIVEQVFQGHQRNGYRFELEGTYEPREYCVQFQETDLN
FVKRLLEDEGIYFWVEHEPDRHVVVISDTQRFEDLPLPNDTLEYLPDGEE
SRAIQGREGVQRLQRTRRIKSNNVALRDFDYHAPSKQLDSDAQVEQQSLG
GIPLEYYDYAAGYRDPEQGERLARLRLEAIQADAHALGGEANARALAVGR
AFTLVGHPALSRNRRYYVTNSELTFIQDGPDSTSQGRNVAVKFRALADDQ
PFRPLLVTKRPRVPGIQSATVVGPEMSEVHTDKLGRIRVHFHWDRYKTTE
ADASCWIRVTQAWAGKGWGVLAMPRVGQEVIVVYVDGDLDRPLATGIVYN
GENPTPYDLPKDIRYTGLVTRSIKRAGGIPNASQLTFDDQHGAERVMIHA
ERDLQQTVERNSSTSIAQDLNLSV
KGTSTSV
VGISVSF
TGISVSY
TGLSVSF
TGVSARF
TGVSTSF
TGVSTSF
TGVSTSF
TGVDTSF
TGVSTGF
KGVDTSF
TGVATSM
VGVSTSI
TGSSNSV
TGVSNSM
TGISSSW
KDVSMST
TGQSESI
TGVSLSY
TGTSNSM
TGTSTSV
TGTSTSI
TGTSMSN
TGSSTSI
TGTSMST
TGSSVST
TGSSMSA
TGSSVGT
TGSSVST
TGSKMSV
TGFSFSY
TGASYED
VGVDLKKLGMQTKN

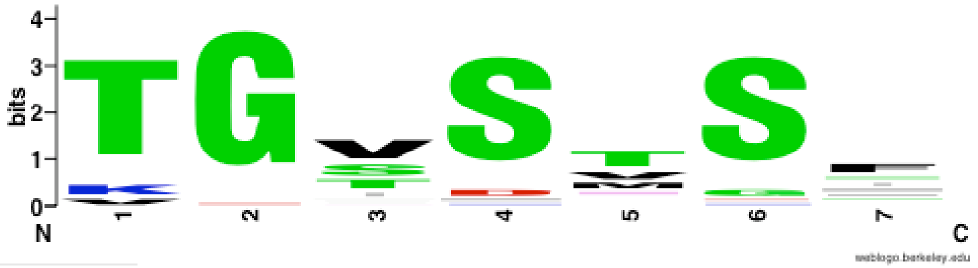

BX571966_BPSS0523 True 763 BX571966G BX571966_GR []
ORG Burkholderia pseudomallei (strain K96243)
>BX571966_BPSS0523
MRLIELRSPLLDPDAVALSFVVHENLSQEPSYQLDLLSHDPDLDFDALLG
STLSADIDLGEGDIRTFNTHVFGGYDTGQMSGQYTYTLELRSWLSFLAEN
RNSRIFQNMSVPQIVEQVFQGHQRNGYRFELEGTYEPREYCVQFQETDLN
FVKRLLEDEGIYFWVEHEPDRHVVVISDTQRFEDLPLPNDTLEYLPDGEE
SRAIQGREGVQRLQRTRRIKSNNVALRDFDYHAPSNKLDSDAQQVSPPNL
EGIPLEYYDYAAGYREPEQGERLARLRLEAIQAESHTLVGEANARALATG
RAFTLIGHPALGRNRRYYVTNSELTFIQDGPDSTSQGRNVAVKFRALADD
QPFRPLLTTPRPEVPGIQSATVVGPEMSEVHTDKLGRIRVHFHWDRYKTT
EADASCWIRVSQAWAGKGWGVIAMPRVGQEVLVTYVDGDLDRPLVTGIVY
NGENPTPYDLPKDIRYTGLVSRSIKRAGGYQNASQITFDDQRGAERVMIH
AERDMQQTVERNSSTSIAQDLNLSV
KGTSTSV
VGISISF
TGISVSY
TGLSVSF
TGVSASF
TGLSTSF
TGVSTSF
TGVSTSF
TGVDTSF
KGVSTSF
TGVDTSF
KGVSTSF
TGVSTSL
TGSSNSV
TGVSNSM
TGISSSW
TDVSMST
TGQSQSI
TGVSLSY
TGTSNSM
TGTSTSV
TGTSTSI
TGTSMSN
TGSSTSI
TGTSMST
TGSSTSV
TGSSVST
TGSSVST
TGSSVST
TGSSVST
TGFSFSY
TGVSYSD
TGIDLKKVGMQVKS

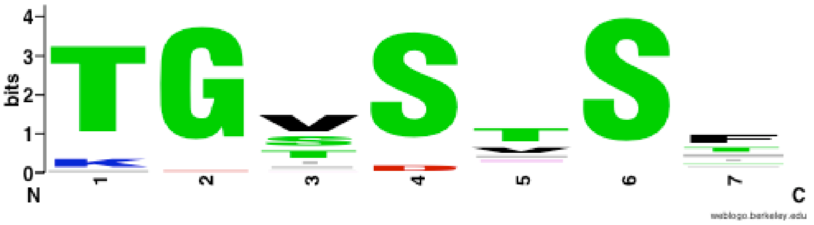

BX936398_YPTB2659 True 970 BX936398C BX936398_GR []
ORG Yersinia pseudotuberculosis (serovar I, strain IP32953)
>BX936398_YPTB2659
MSRTINLSSPAMPYLLGEPALVLSKLEGEEAFSTLYSYTITAKTPANPLI
PWQAASNVDLKSLIGKEMTIEMELDGNGLGDVRSVGKGTREITGLVERAR
YIGRDANQAIFEITIKPWFYLANLTSDFKIYQNKNVVDIIEEVFSDYNFP
FEKRLATTYPILDFQVQYGETDFNFLQRLMEEWGIYWFFEHQDHKQKLIL
VDHVGAHKRSFSTAYHAIEYLSDEPKAGEEYITQFQTQETLTSGTWVTND
YDFTKSRADILAMDSKPRKTSFNDLEIYHWPGDYDQPDIGEHLSRVRIEE
RGALGSRAVGSGQLRGIVCGANFELKGFPVDKANREYMIISSRLTVAEVD
QLSGGEDAFSCESSFTVQPTTKIYRHPQVTPKPKTNGPQNAIIVGPPGEE
IWTDEYGRVKVRFVWDRYGTNSESDSCWLRVSQAWAGNSFGGIYIPRIGQ
EVIVDCINGDPDRPMVMGSLYNNVTRPPWDLPANATQSGMVSRTVGGGLT
NYNGVRFEDKSGLEQYWEQAERDMSRLTKNNETQIIGADSVLNVGANRSE
VVGANYNQDVLGTSMLAIGAASMLQVGLARSVVVGGAHSLNVVLANATNV
GGANMTNVGGFNGLAVGGAHQTAVGGAATLAAGGPIAIGAGGELILSGDI
VKIVGKSKVIIQGGEVHINSDDCCGRKTGNGGGAFGAFASLAGLLPLAGA
IALPLPLPLLPIVVPPVTS
PPDPTV
PPDPTL
PPDPTL
PPDPTL
PPETTA
PPETTA
PPETTA
PPETTA
PPETTA
PPETTA
PPETTA
PPETTA
PPETTA
PPETTA
PPETTA
PPETTA
PPETTA
PPETTA
PPETTA
PPETTA
PPETTA
PPETTA
PPETTA
PPETTA
PPETTA

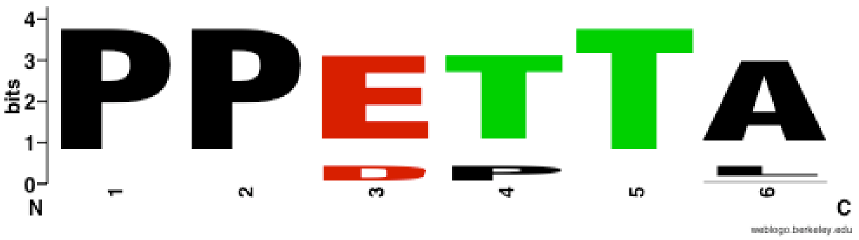

PRRKQR
HRQKQR
HRQKQR
HRQKQR
HRQRQR
HRRKQR
HRQRQR
HRQRQR

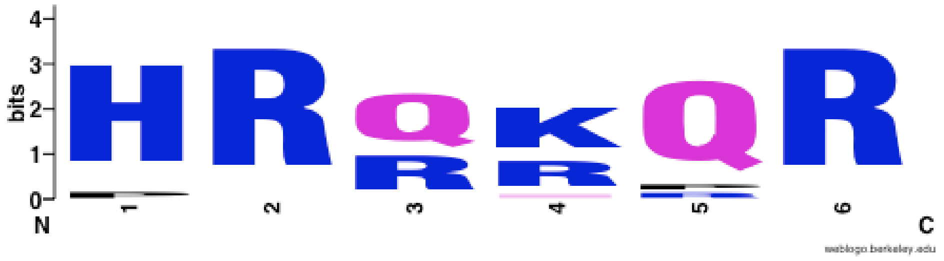

HRRKQR
HRRKQR
HRRKPR
HRQRQR
HRQRQR
HRRNRR
EHRQGRRRHRLDFIDKR

AE017042_YP_2441 False 777 AE017042C AE017042_GR []
ORG Yersinia pestis (biovar Mediaevalis, strain 91001)
>AE017042_YP_2441
MSRTINLSSPAMPYLLGEPALVLSKLEGEEAFSTLYSYTITAKTPANPLI
PWQAASNVDLKSLIGKEMTIEMELDGNGLGDVRSVGKGTREITGLVERAR
YIGRDANQAIFEITIKPWFYLANLTSDFKIYQNKNVVDIIEEVFSDYNFP
FEKRLATTYPILDFQVQYGETDFNFLQRLMEEWGIYWFFEHQDHKQKLIL
VDHVGAHKRSFSTAYHAIEYLSDEPKAGEEYITQFQTQETLTSGTWVTND
YDFTKSRADILAMDSKPRKTSFNDLEIYHWPGDYDQPDIGEHLSRVRIEE
RGALGSRAVGSGQLRGIVCGANFELKGFPVDKANREYMIISSRLTVAEVD
QLSGGEDAFSCESSFTVQPTTKIYRHPQVTPKPKTNGPQNAIIVGPPGEE
IWTDEYGRVKVRFVWDRYGTNSESDSCWLRVSQAWAGNSFGGIYIPRIGQ
EVIVDCINGDPDRPMVMGSLYNNVTRPPWDLPANATQSGMVSRTVGGGLT
NYNGVRFEDKSGLEQYWEQAERDMSRLTKNNETQII
GADSVLNV
GANRSEVV
GANYNQDV
LGTSMLAI
GAASMLQV
GLARSVVV
GGAHSLNV
VLANATNV
GGANMTNV
GGFNGLAV
GGAHQTAV
GGAATLAA
GGPIAIGA
GGELILSG

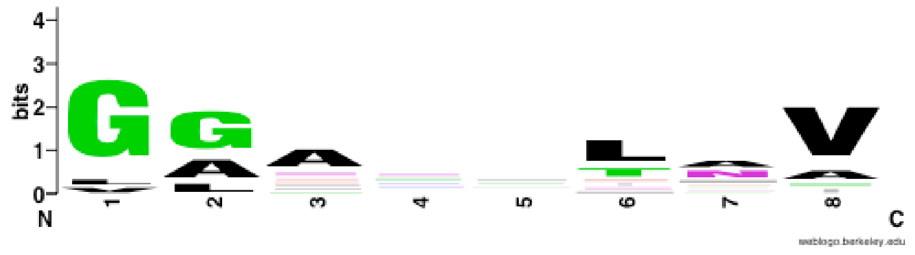

DIVKIVGKSKVIIQGGEVHINSDDCCGRKTGNGGGAFGAFASLAGLLPLA
GAIALPLPLPLLPIV
VPPVTS
PPDPTV
PPDPTL
PPDPTL
PPDPTL
PPDPTL
PPETTA
PPETTA
PPEPTR

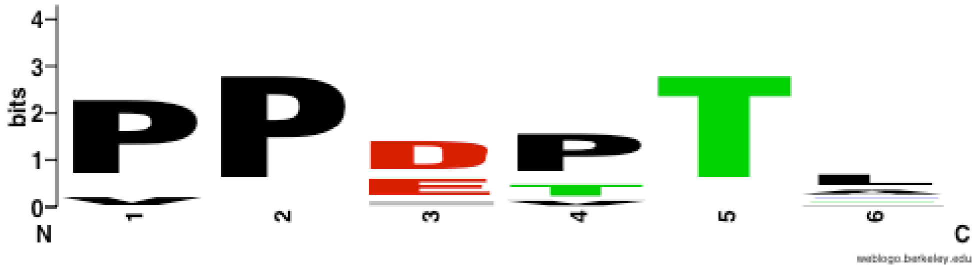

TPPGTQTPPP

AE004091_PA2373 True 668 AE004091E AE004091_GR []
ORG Pseudomonas aeruginosa (strain LMG 12228 / ATCC 15692 / PRS 101 / 1C /
>AE004091_PA2373
MPRPTDTNTTLSLSASTLGDLYPQTLSGGEALNELGSLTLGGYSATALTL
SNAVATHLTATLHNDADQRPLDGLVAEIRQLPGDASAERYQVLLRPWLWW
LTLASNNRVFQNLATSDIVTQVFDQHGFSDYQLQLSGSYQPREYCVQYGE
SDLAFVSRLLEEDGIFWFFTHAAGKHTLVLADSNDAFPPIPNGPQVAYLG
QGIGVRELQGVRSAQYSLQAVSGTYSATDYEFTTPGTSLYSQAEAVSGAA
GVYQHPGGYTAKAQGDSLTKQRIDGLRSQETRLIGESDCRWLVPGHWFTL
SGHDDDSLNIDWVLTSVTHDASHAHYRNRFEAIPKATAYRPARVTPKPRM
HTQTALVVGKAGEEIWTDQYGRIKIQFPWDRDGKNDETSSCWVRVVLPWS
GKGFGMQFVPRIGQEVIVTFIDGDPDRPLVTGCVYNGDNALPYALPDNQT
QSGIKTNSSKGGGGFNELRFEDKKDAEEVFLQAQKDLNVNVLNDSTASIG
HDETLTVQNARTRTVKEGDETVTLEKGKRTVTIQTGSDSLDVK
DTRTVTVG
ADQTHSTG
GNYSHKVS
GNFELTVD
GNLTIKVS
GTLALQSG
GSLTLKSD
ADLAAQAG
TSLTSKAG
TSLTNQAG
TSLTNKAG
TSLTNDAG
VSLTNKAG
AEQTVDGG
GMLTIKGG
LVKVN

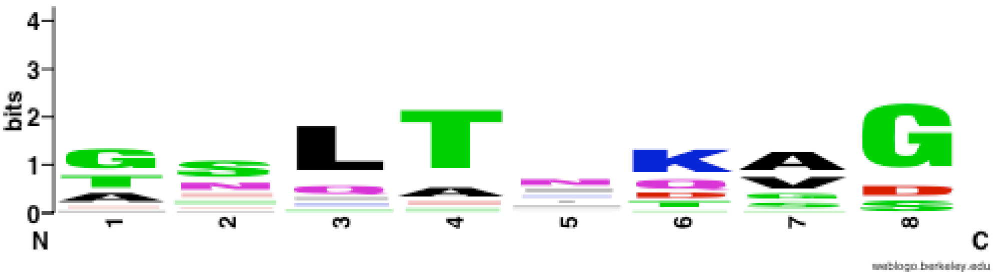

BX571966_BPSS0105 True 899 BX571966E BX571966_GR []
ORG Burkholderia pseudomallei (strain K96243)
>BX571966_BPSS0105
MFLGKWKREDCMPNHFSNGRTNQSRTVVIRSGAMPRLLGQPALEFLSLRG
EEHLGKLYTYELLLRTPDDFHVPLATSANLDLKAMIGTEMTVCIQLDGIG
TGAQGGVGAGAREISGLVVKAGFLRCEGRYNVYRIELRPWLWLATLTSDY
KIFQDKSVVEIIDTVLHDYPYPVEKRLDIDKYSVAGESARNEPRAFQVQY
GETDFDFVQRLMEEWGIYWFFEHSDNKHRLVLCDHIGGHRKAPSEAYHEI
AHHPEGGKIDIEYINYFSTDEALRPGRVVIDDFDFTRPLASLVTSNHQPR
ETNWGEGELFEWPGDYTDSKHGDLISRVRMEERRATGSRAYGRGNVRGLA
CGHTFVLSKHKHDGANREYLVIESALMLTEVADETGSGYRYECDNELVVQ
PSNEVFRMPRETPKPTTSGPQSAIVVGPPGHEVWTDEFGRVKIRFLWDRY
ARNDATDSCWVRVSQAWAGVNFGGIYIPRIGQEVIVGFMNGDPDRPLILG
SLYNTITPPPWDLPGDATKSGFKSKSITGGRENYNGIRFEDKLGAEEFHM
QAEKDMNRLTKNDESHTVGANFSIGVGLTHTRAVGAMFSSIVGGAASYAV
GGAESTMIGGAYALNVGGAHAVAVGGASSVSVGGAYARNVGGAYALTVGG
VLSIVCGASSITMTACGSIKLVGKNIRIIGSDEVVVQGAPLQLNPGDSDC
GGGGGGGGGGGAIPPIPLPSFFLDITKPILPPPPPPPTEVPPDPTPTPTP
TPTPTPTPTPTPTPTPTPTPTPTPTPTPTPTPTPTPTPTPTPTPTPTPTP
TPTPTPTPTPTPTPTPTPTPTPMPTPTPTPTPTPTPTPTPTPTPTPTPTP
TPTPTPTPTPTPTPTPTPTPTPTPTPTPTPTPTPTPTPTPTPTPTSSEI

CP000125_BURPS1710b_A2081 True 763 CP000125H CP000125_GR
[]
ORG Burkholderia pseudomallei (strain 1710b)
>CP000125_BURPS1710b_A2081
MRLIELRSPLLDPDAVALSFVVHESLSQEPSYQLDLLSHDPDLDFDALLG
STLSADIDLGEGDIRTFNTHVFGGYDTGQMSGQYTYTLELRSWLSFLAEN
RNSRIFQNMSVPQIVEQVFQGHQRNGYRFELEGTYEPREYCVQFQETDLN
FVKRLLEDEGIYFWVEHEPDRHVVVISDTQRFEDLPLPNDTLEYLPDGEE
SRAIQGREGVQRLQRTRRIKSNNVALRDFDYHAPSNKLDSDAQQVSPPNL
EGIPLEYYDYAAGYREPEQGERLARLRLEAIQAESHTLVGEANARALATG
RAFTLIGHPALGRNRRYYVTNSELTFIQDGPDSTSQGRNVAVKFRALADD
QPFRPLLTTPRPEVPGIQSATVVGPEMSEVHTDKLGRIRVHFHWDRYKTT
EADASCWIRVSQAWAGKGWGVIAMPRVGQEVLVTYVDGDLDRPLVTGIVY
NGENPTPYDLPKDIRYTGLVSRSIKRAGGYQNASQITFDDQRGAERVMIH
AERDMQQTVERNSSTSIAQDLNLSV
KGTSTSV
VGISISF
TGISVSY
TGLSVSF
TGVSASF
TGLSTSF
TGVSTSF
TGVSTSF
TGVDTSF
KGVSTSF
TGVDTSF
KGVSTSF
TGVSTSL
TGSSNSV
TGVSNSM
TGISSSW
TDVSMST
TGQSQSI
TGVSLSY
TGTSNSM
TGTSTSV
TGTSTSI
TGTSMSN
TGSSTSI
TGTSMST
TGSSTSV
TGSSVST
TGSSVST
TGSSVST
TGSSVST
TGFSFSY
TGVSYSD

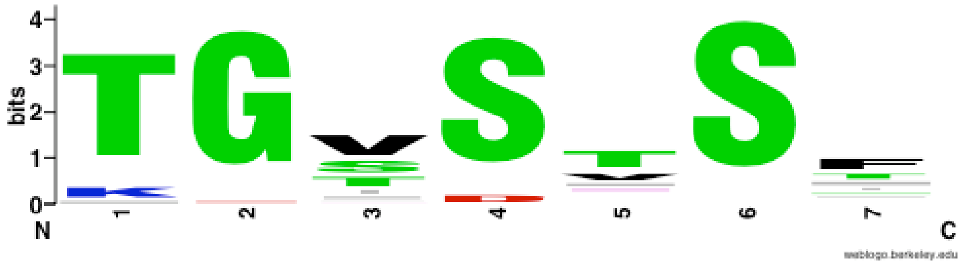

TGIDLKKVGMQVKS

CP000573_BURPS1106A_A0709 True 762 CP000573G CP000573_GR
[]
ORG Burkholderia pseudomallei (strain 1106a)
>CP000573_BURPS1106A_A0709
MRLIELRSPLLDPDAVALSFVVHENLSQEPSYQLDLLSHDSNLDFDALLG
STLSADIDLGEGDIRTFNTHVFGGYDTGQMSGQYTYTLELRSWLSFLAEN
RNSRIFQDLSVPQIVEQVFQGHQRNGYRFELEGTYEPREYCVQFQETDLN
FVKRLLEDEGIYFWVEHEPDRHVVVISDTQRFEDLPLPNDTLEYLPDGEE
SRAIQGREGVQRLQRTRRIKSNNVALRDFDYHAPSKQLDSDAQVEQQSLG
GIPLEYYDYAAGYRDPEQGERLARLRLEAIQADAHALGGEANARALAVGR
AFTLVGHPALSRNRRYYVTNSELTFIQDGPDSTSQGRNVAVKFRALADDQ
PFRPLLVTKRPRVPGIQSATVVGPEMSEVHTDKLGRIRVHFHWDRYKTTE
ADASCWIRVTQAWAGKGWGVLAMPRVGQEVIVVYVDGDLDRPLATGIVYN
GENPTPYDLPKDIRYTGLVTRSIKRAGGIPNASQLTFDDQHGAERVMIHA
ERDLQQTVERNSSTSIAQDLNLSV
KGTSTSV
VGISVSF
TGISVSY
TGLSVSF
TGVSARF
TGVSTSF
TGVSTSF
TGVSTSF
TGVDTSF
TGVSTGF
KGVDTSF
TGVATSM
VGVSTSI
TGSSNSV
TGVSNSM
TGISSSW
KDVSMST
TGQSESI
TGVSLSY
TGTSNSM
TGTSTSV
TGTSTSI
TGTSMSN
TGSSTSI
TGTSMST
TGSSVST
TGSSMSA
TGSSVGT
TGSSVST
TGSKMSV
TGFSFSY
TGASYED
VGVDLKKLGMQTKN

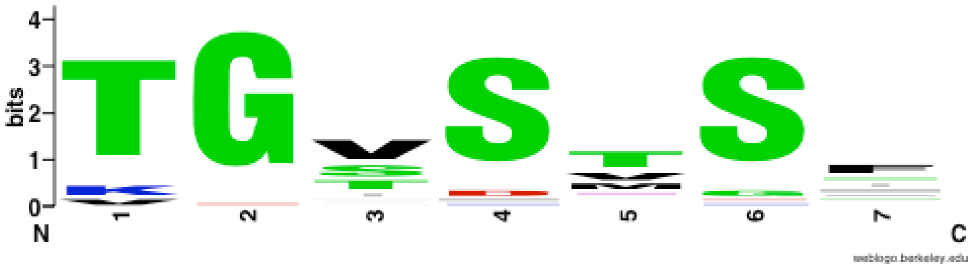

AM286415_YE2684 True 904 AM286415A AM286415_GR []
ORG Yersinia enterocolitica (serovar O:8 / biotype 1B, strain 8081)
>AM286415_YE2684
MSRTIKLSSPAMPYLLGEPALVLSKLEGEEAFSTLYSYTITAKTPANPSI
PWQTASNVDLKALIGKEMTIEMELDGNGLDYVKGVGKGTREISGLVEKAR
FIGRDANQAMFEIVLRPWFYLTDLTSDFKIFQNKNVVDIIDEVLADYNFP
FEKRLATTYPILDFQVQYGETDFNFLQRLMEEWGIYWFFEHDDHKQKLIL
VDHVGAHKRYFSEAYHTIEYLSDEPKAGEEYITQFQTQETLTTGRWVYND
YDFTKSRADILTMDSKPRKTSFSDLEIYQWPGDYDQPDIGEHLARVRIEE
RGALGSRAVGCGEVRGIVCGANFELQGFPVDKANREYMVISSRLSVSEVA
QLSRGDEFHYETTFTVQPTTKIYRHPQLTPKPKTSGPQNAIVVGPPGEEV
WTDEYGRVKVRFVWDRYGKNAESDSCWLRVSQAWAGNSFGGIYIPRIGQE
VLVDCINGDPDRPLVSGSLYNNVTRPPWDLPANATQSGMVSRTIGGGLTN
YNGVRFEDKPGLEQYWEQAERDMARLTKNDETQVIGANADLSVGANRSVL
VGANSTSTVLGASAQSVALASSIQVGLARSVVVGATHALNVALANATNVG
GANLTNVGGYNALSVGGAHQVAAGGAATLVAGGAVAIGAGGELVLSADTI
KIVGKKKVIIQGGEIHLNSDDCCGGGGGGAGGGLAALSSLAGLKLLGGGI
GLPLPLPPLPIPPIIPPIPPIDPPEPTAETPEPTIDPPEPTTET
PEPTVT
PEPTVT
PEPTVT
PEPTVT
PEPTVT
PEPTVT
PEPTVT
PEPTVT
PEPTVT
PEPTVT
PEPTDT
PEPTDT
PEPTDT
PEPTDT
PEPTDT
PEPTDT
PEPTDT
PEPTDT
PEPTDT
PEPTDT
PEPTVT
PEPTVT
PEPTDT
PEPTEP
PETPEP
TPTDEQ
TPPP

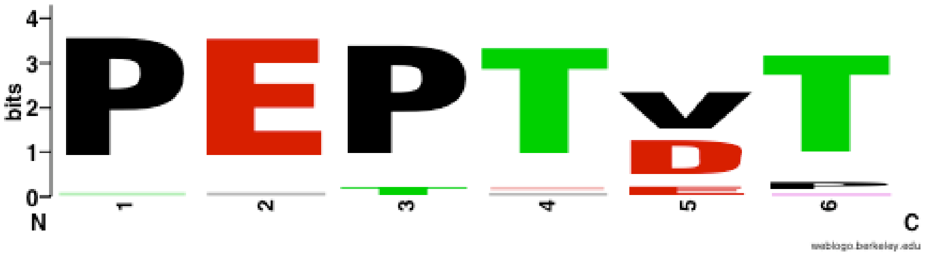

CP000305_YPN_2222 True 867 CP000305D CP000305_GR []
ORG Yersinia pestis (biovar Antiqua Nepal516, strain Nepal516)
>CP000305_YPN_2222
MSRTINLSSPAMPYLLGEPALVLSKLEGEEAFSTLYSYTITAKTPANPLI
PWQAASNVDLKSLIGKEMTIEMELDGNGLGDVRSVGKGTREITGLVERAR
YIGRDANQAIFEITIKPWFYLANLTSDFKIYQNKNVVDIIEEVFSDYNFP
FEKRLATTYPILDFQVQYGETDFNFLQRLMEEWGIYWFFEHQDHKQKLIL
VDHVGAHKRSFSTAYHAIEYLSDEPKAGEEYITQFQTQETLTSGTWVTND
YDFTKSRADILAMDSKPRKTSFNDLEIYHWPGDYDQPDIGEHLSRVRIEE
RGALGSRAVGSGQLRGIVCGANFELKGFPVDKANREYMIISSRLTVAEVD
QLSGGEDAFSCESSFTVQPTTKIYRHPQVTPKPKTNGPQNAIIVGPPGEE
IWTDEYGRVKVRFVWDRYGTNSESDSCWLRVSQAWAGNSFGGIYIPRIGQ
EVIVDCINGDPDRPMVMGSLYNNVTRPPWDLPANATQSGMVSRTVGGGLT
NYNGVRFEDKSGLEQYWEQAERDMSRLTKNNETQIIGADSVLNVGANRSE
VVGANYNQDVLGTSMLAIGAASMLQVGLARSVVVGGAHSLNVVLANATNV
GGANMTNVGGFNGLAVGGAHQTAVGGAATLAAGGPIAIGAGGELILSGDI
VKIVGKSKVIIQGGEVHINSDDCCGRKTGNGGGAFGAFASLAGLLPLAGA
IALPLPLPLLPIVVPPVTS
PPDPTV
PPDPTL
PPDPTL
PPDPTL
PPDPTL
PPETTA
PPETTA
PPETTA
PPETTA
PPETTA
PPETTA
PPETTA
PPETTA
PPETTA
PPETTA
PPETTA
PPETTA
PPETTA
PPETTA
PPETTA
PPETTA
PPETTA
PPEPTR
TPPGTQTPPP

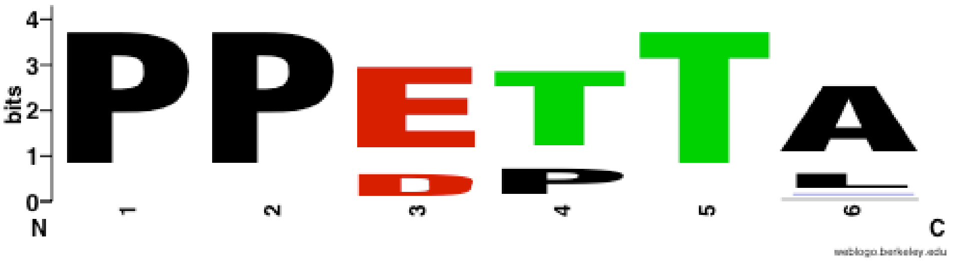

CP000085_BTH_II0129 True 872 CP000085A CP000085_GR []
ORG Burkholderia thailandensis (strain E264 / ATCC 700388 / DSM 13276 / CIP
>CP000085_BTH_II0129
MPNHFSNGRTNQSRTVVIRSGAMPRLLGQPALKFLSLRGEEHLGKLYTYE
LLLRTPDDFHVPLATSANLDLKAMIGTEMTVGIQLDGIGTGAQGGVGAGA
REISGLVVKAGFLRSEGRYNVYRIELRPWLWLATLTSDYKIFQDKSVVEI
IDTVLHDYPYPVEKRLDIDKYSVAGESARNEPRAFQVQYGETDFDFVQRL
MEEWGIYWFFEHSDNKHRLVLCDHIGGHRKAPSEAYHEIAHHPEGGKIDI
EYINYFSTDEALRPGRVVIDDFDFTRPLASLVTSNHQPRETNWGEGELFE
WPGDYTDSKHGDLISRVRMEERRATGSRAYGRGNVRGLACGHTFVLAKHK
HDDANREYLVIESALMLTEVADETGSGYRYECDNELVVQPSNEVFRMPRE
TPKPTTNGPQSAIVVGPPGHEVWTDEFGRVKIRFLWDRYARNDATDSCWV
RVSQAWAGVNFGGIYIPRIGQEVIVGFMNGDPDRPLILGSLYNTITPPPW
DLPGDATKSGFKSKSITGGRENYNGIRFEDKLGAEEFHMQAEKDMNRLTK
NDESHTIGANYSIGVGITHTRAVGAMFSSIVGGAASYAVGGAESTMIGGA
YALNVGGAHAVAVGGASSVSVGGAYARNVGGAYALTVGGVLSIVCGASSI
TMTACGSIKIVGKNIRIIGSDEVVVQGAPLQLNPGDSDCGGGGGGGGGGG
AIPPIPLPSFFLDITKPILPPPPPPPTEVPPEPTPTPEPTPTPEPTPTPT
PTPTPTPTPTPTPTPTPTPTPTPTPTPTPTPTPTPTPTPTPTPTPTPTPT
PTPTPTPTPTPTPTPTPTPTPTPTPTPTPTPTPTPTPTPTPTPTPTPTPT
PTPTPTPTPTPTPTPTPTSSEI

BX936398_YPTB0648 True 782 BX936398A BX936398_GR
[('COG3889', 637, 735)]
ORG Yersinia pseudotuberculosis (serovar I, strain IP32953)
>BX936398_YPTB0648
MQLIEIENEVLSGQDAFPLSFKTQEKVSGNPSYQLVFQVAEADLDLVSLL
GEIIKVRIELPDSAGYRTFFTYVIAGADEGQRQDKFVYSLELSTWTWFLM
QNRNCRIFQDLNIIDIIEQVFSKYNFADYRFDIVGNYRLREYCVQFAETD
FDFVNRLMEDEGVWYYFEHNEDKHTLVMTDQQQFPVLEGHYAELSFLPDS
EEMRAIREGIQRIQRSQRIHSSEIVLRDFDFLNPRNTLQTHIEESRQHLQ
GVPLEWYDYAAGYTDPQHGESIARLRLEAIQSNGQLLSGESNATGLVPGR
SFALVQHPDNNRNRGFKLISCDYSFVQDGPDSASQGRNVACKFKALNDDV
VYRPQCVTPPPKVPGVQSATVVGARESEVHTDKFARIRVHFHWDRYKTTE
DDSSCWIRVVQAWAGKGWGVLAMPRVGQEVLVNYVDGDLDRPMVTGIVYN
GENPPPYRLPDHINYSGFVSRSLRFGQPQHASQLTFDDNRGNERIMLHAE
RDLQRTVERNSATAVGQDKYDTVERTATEWINNHISYKDFSFSV
TGMSVSA
TGISVST
TGTSLSV
TGMSTSV
TGVSVGF
TLIDTSF
TGVSTSF
TGVGTSF
TGASNSL
TGVSNSM
TGCSSSF
TGTSNSM
TGSSHSM
TGMSTSI
TGHSMSQ
TGSSSSI
TGDSTSF
TGSSVSS
TGSSVST
TGVSTST
TGSSTST
TGCSVST
TGSSTST
TGNSVSM
TGNSTST
TGCSIST
TGSSIGT
VGSSIST
TGSSVST
TGSSIST
TGLSVSY
TGAQYSD

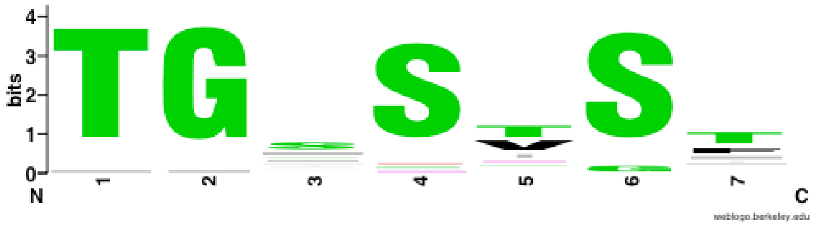

VGVDLKTVGMQSKN

AE017042_YP_3672 True 782 AE017042H AE017042_GR
[('COG3889', 637, 735)]
ORG Yersinia pestis (biovar Mediaevalis, strain 91001)
>AE017042_YP_3672
MQLIEIENEVLSGQDAFPLSFKTQEKVSGNPSYQLVFQVAEADLDLVSLL
GEIIKVRIELPDSAGYRTFFTYVIAGADEGQRQDKFVYSLELSTWTWFLM
QNRNCRIFQDLNIIDIIEQVFSKYNFADYRFDIVGNYRLREYCVQFAETD
FDFVNRLMEDEGVWYYFEHNEDKHTLVMTDQQQFPVLEGHYAELSFLPDS
EEMRAIREGIQRIQRSQRIHSSEIVLRDFDFLNPRNTLQTHIEESRQHLQ
GVPLEWYDYAAGYTDPQHGESIARLRLEAIQSNGQLLSGESNATGLVPGR
SFALVQHPDNNRNRGFKLISCDYSFVQDGPDSASQGRNVACKFKALNDDV
VYRPQCVTPPPKVPGVQSATVVGARESEVHTDKFARIRVHFHWDRYKTTE
DDSSCWIRVVQAWAGKGWGVLAMPRVGQEVLVNYVDGDLDRPMVTGIVYN
GENPPPYRLPDHINYSGFVSRSLRFGQPQHASQLTFDDNRGNERIMLHAE
RDLQRTVERNSATAVGQDKYDTVERTATEWINNHISYKDFSFSV
TGMSVSA
TGISVST
TGTSLSV
TGMSTSV
TGVSVGF
TLIGTSF
TGVSTSF
TGVGTSF
TGASNSL
TGVSNSM
TGCSSSF
TGTSNSM
TGSSHSM
TGMSTSI
TGHSMSQ
TGSSSSI
TGDSTSF
TGSSVSS
TGSSVST
TGVSTST
TGSSTST
TGCSVST
TGSSTST
TGNSVSM
TGNSTST
TGCSIST
TGSSIGT
VGSSIST
TGSSVST
TGSSIST
TGLSVSY
TGAQYSD

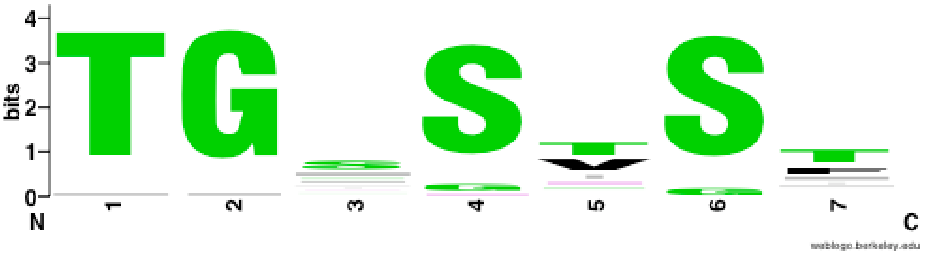

VGVDLKTVGMQSKN

AE009952_y3668 True 782 AE009952G AE009952_GR
[('COG3889', 637, 735)]
ORG Yersinia pestis (biovar Mediaevalis, strain KIM5)
>AE009952_y3668
MQLIEIENEVLSGQDAFPLSFKTQEKVSGNPSYQLVFQVAEADLDLVSLL
GEIIKVRIELPDSAGYRTFFTYVIAGADEGQRQDKFVYSLELSTWTWFLM
QNRNCRIFQDLNIIDIIEQVFSKYNFADYRFDIVGNYRLREYCVQFAETD
FDFVNRLMEDEGVWYYFEHNEDKHTLVMTDQQQFPVLEGHYAELSFLPDS
EEMRAIREGIQRIQRSQRIHSSEIVLRDFDFLNPRNTLQTHIEESRQHLQ
GVPLEWYDYAAGYTDPQHGESIARLRLEAIQSNGQLLSGESNATGLVPGR
SFALVQHPDNNRNRGFKLISCDYSFVQDGPDSASQGRNVACKFKALNDDV
VYRPQCVTPPPKVPGVQSATVVGARESEVHTDKFARIRVHFHWDRYKTTE
DDSSCWIRVVQAWAGKGWGVLAMPRVGQEVLVNYVDGDLDRPMVTGIVYN
GENPPPYRLPDHINYSGFVSRSLRFGQPQHASQLTFDDNRGNERIMLHAE
RDLQRTVERNSATAVGQDKYDTVERTATEWINNHISYKDFSFSV
TGMSVSA
TGISVST
TGTSLSV
TGMSTSV
TGVSVGF
TLIGTSF
TGVSTSF
TGVGTSF
TGASNSL
TGVSNSM
TGCSSSF
TGTSNSM
TGSSHSM
TGMSTSI
TGHSMSQ
TGSSSSI
TGDSTSF
TGSSVSS
TGSSVST
TGVSTST
TGSSTST
TGCSVST
TGSSTST
TGNSVSM
TGNSTST
TGCSIST
TGSSIGT
VGSSIST
TGSSVST
TGSSIST
TGLSVSY
TGAQYSD

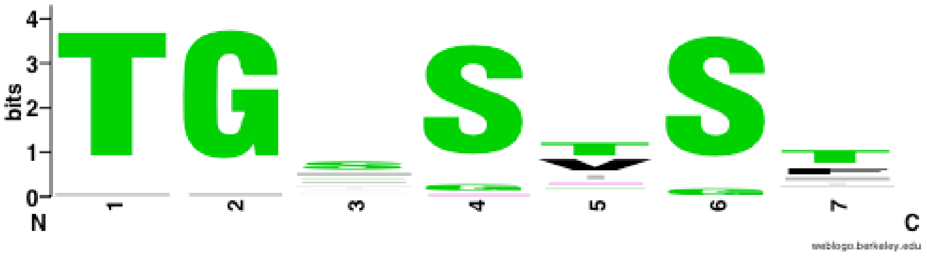

VGVDLKTVGMQSKN

AL590842_YPO0507 True 782 AL590842A AL590842_GR
[('COG3889', 637, 735)]
ORG Yersinia pestis (biovar Orientalis, strain CO-92)
>AL590842_YPO0507
MQLIEIENEVLSGQDAFPLSFKTQEKVSGNPSYQLVFQVAEADLDLVSLL
GEIIKVRIELPDSAGYRTFFTYVIAGADEGQRQDKFVYSLELSTWTWFLM
QNRNCRIFQDLNIIDIIEQVFSKYNFADYRFDIVGNYRLREYCVQFAETD
FDFVNRLMEDEGVWYYFEHNEDKHTLVMTDQQQFPVLEGHYAELSFLPDS
EEMRAIREGIQRIQRSQRIHSSEIVLRDFDFLNPRNTLQTHIEESRQHLQ
GVPLEWYDYAAGYTDPQHGESIARLRLEAIQSNGQLLSGESNATGLVPGR
SFALVQHPDNNRNRGFKLISCDYSFVQDGPDSASQGRNVACKFKALNDDV
VYRPQCVTPPPKVPGVQSATVVGARESEVHTDKFARIRVHFHWDRYKTTE
DDSSCWIRVVQAWAGKGWGVLAMPRVGQEVLVNYVDGDLDRPMVTGIVYN
GENPPPYRLPDHINYSGFVSRSLRFGQPQHASQLTFDDNRGNERIMLHAE
RDLQRTVERNSATAVGQDKYDTVERTATEWINNHISYKDFSFSV
TGMSVSA
TGISVST
TGTSLSV
TGMSTSV
TGVSVGF
TLIGTSF
TGVSTSF
TGVGTSF
TGASNSL
TGVSNSM
TGCSSSF
TGTSNSM
TGSSHSM
TGMSTSI
TGHSMSQ
TGSSSSI
TGDSTSF
TGSSVSS
TGSSVST
TGVSTST
TGSSTST
TGCSVST
TGSSTST
TGNSVSM
TGNSTST
TGCSIST
TGSSIGT
VGSSIST
TGSSVST
TGSSIST
TGLSVSY
TGAQYSD

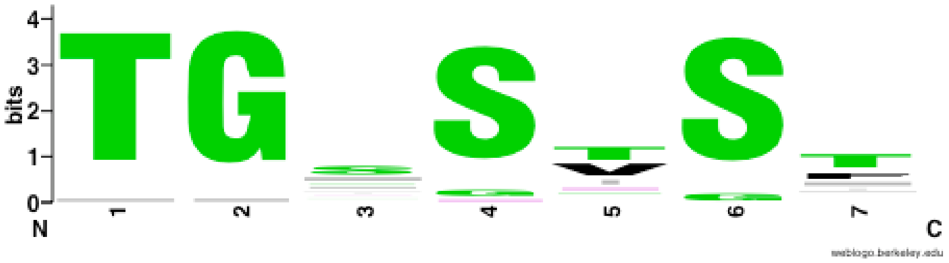

VGVDLKTVGMQSKN

CP000668_YPDSF_3127 True 782 CP000668F CP000668_GR [('COG3889', 637, 735)]
ORG Yersinia pestis (strain Pestoides F)
>CP000668_YPDSF_3127
MQLIEIENEVLSGQDAFPLSFKTQEKVSGNPSYQLVFQVAEADLDLVSLL
GEIIKVRIELPDSAGYRTFFTYVIAGADEGQRQDKFVYSLELSTWTWFLM
QNRNCRIFQDLNIIDIIEQVFSKYNFADYRFDIVGNYRLREYCVQFAETD
FDFVNRLMEDEGVWYYFEHNEDKHTLVMTDQQQFPVLEGHYAELSFLPDS
EEMRAIREGIQRIQRSQRIHSSEIVLRDFDFLNPRNTLQTHIEESRQHLQ
GVPLEWYDYAAGYTDPQHGESIARLRLEAIQSNGQLLSGESNATGLVPGR
SFALVQHPDNNRNRGFKLISCDYSFVQDGPDSASQGRNVACKFKALNDDV
VYRPQCVTPPPKVPGVQSATVVGARESEVHTDKFARIRVHFHWDRYKTTE
DDSSCWIRVVQAWAGKGWGVLAMPRVGQEVLVNYVDGDLDRPMVTGIVYN
GENPPPYRLPDHINYSGFVSRSLRFGQPQHASQLTFDDNRGNERIMLHAE
RDLQRTVERNSATAVGQDKYDTVERTATEWINNHISYKDFSFS
VTGMSVS
ATGISVS
TTGTSLS
VTGMSTS
VTGVSVG
FTLIGTS
FTGVSTS
FTGVGTS
FTGASNS
LTGVSNS
MTGCSSS
FTGTSNS
MTGSSHS
MTGMSTS
ITGHSMS
QTGSSSS
ITGDSTS
FTGSSVS
STGSSVS
TTGVSTS
TTGSSTS
TTGCSVS
TTGSSTS
TTGNSVS
MTGNSTS
TTGCSIS
TTGSSIG
TVGSSIS
TTGSSVS
TTGSSIS
TTGLSVS
YTGAQYS
DVGVDLKTVGMQSKN

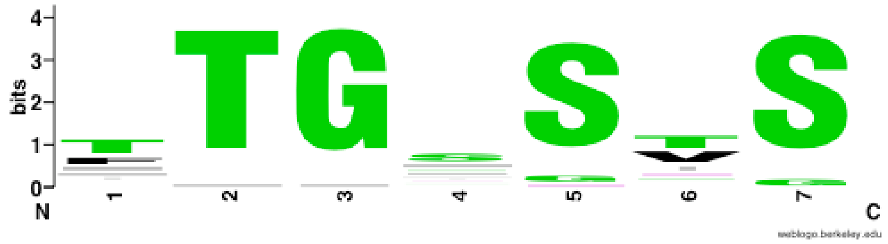

CP000305_YPN_0382 True 782 CP000305B CP000305_GR
[('COG3889', 637, 735)]
ORG Yersinia pestis (biovar Antiqua Nepal516, strain Nepal516)
>CP000305_YPN_0382
MQLIEIENEVLSGQDAFPLSFKTQEKVSGNPSYQLVFQVAEADLDLVSLL
GEIIKVRIELPDSAGYRTFFTYVIAGADEGQRQDKFVYSLELSTWTWFLM
QNRNCRIFQDLNIIDIIEQVFSKYNFADYRFDIVGNYRLREYCVQFAETD
FDFVNRLMEDEGVWYYFEHNEDKHTLVMTDQQQFPVLEGHYAELSFLPDS
EEMRAIREGIQRIQRSQRIHSSEIVLRDFDFLNPRNTLQTHIEESRQHLQ
GVPLEWYDYAAGYTDPQHGESIARLRLEAIQSNGQLLSGESNATGLVPGR
SFALVQHPDNNRNRGFKLISCDYSFVQDGPDSASQGRNVACKFKALNDDV
VYRPQCVTPPPKVPGVQSATVVGARESEVHTDKFARIRVHFHWDRYKTTE
DDSSCWIRVVQAWAGKGWGVLAMPRVGQEVLVNYVDGDLDRPMVTGIVYN
GENPPPYRLPDHINYSGFVSRSLRFGQPQHASQLTFDDNRGNERIMLHAE
RDLQRTVERNSATAVGQDKYDTVERTATEWINNHISYKDFSFSV
TGMSVSA
TGISVST
TGTSLSV
TGMSTSV
TGVSVGF
TLIGTSF
TGVSTSF
TGVGTSF
TGASNSL
TGVSNSM
TGCSSSF
TGTSNSM
TGSSHSM
TGMSTSI
TGHSMSQ
TGSSSSI
TGDSTSF
TGSSVSS
TGSSVST
TGVSTST
TGSSTST
TGCSVST
TGSSTST
TGNSVSM
TGNSTST
TGCSIST
TGSSIGT
VGSSIST
TGSSVST
TGSSIST
TGLSVSY
TGAQYSD
VGVDLKTVGMQSKN

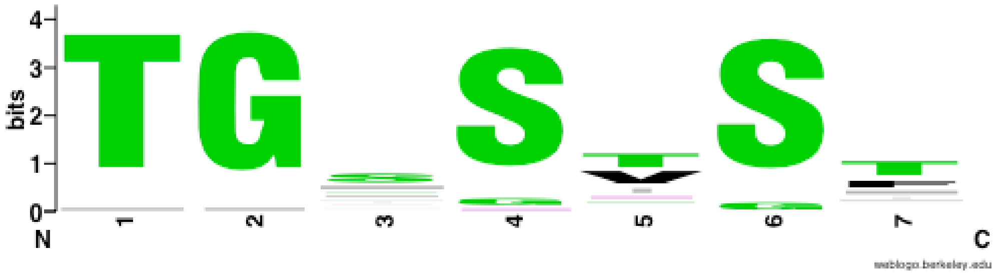

CP000308_YPA_3589 True 779 CP000308G CP000308_GR
[('COG3889', 634, 732)]
ORG Yersinia pestis (biovar Antiqua Antiqua, strain Antiqua)
>CP000308_YPA_3589
MQLIEIENEVLSGQDAFPLSFKTQEKVSGNPSYQLVFQVAEADLDLVSLL
GEIIKVRIELPDSAGYRTFFTYVIAGADEGQRQDKFVYSLELSTWTWFLM
QNRNCRIFQDLNIIEQVFSKYNFADYRFDIVGNYRLREYCVQFAETDFDF
VNRLMEDEGVWYYFEHNEDKHTLVMTDQQQFPVLEGHYAELSFLPDSEEM
RAIREGIQRIQRSQRIHSSEIVLRDFDFLNPRNTLQTHIEESRQHLQGVP
LEWYDYAAGYTDPQHGESIARLRLEAIQSNGQLLSGESNATGLVPGRSFA
LVQHPDNNRNRGFKLISCDYSFVQDGPDSASQGRNVACKFKALNDDVVYR
PQCVTPPPKVPGVQSATVVGARESEVHTDKFARIRVHFHWDRYKTTEDDS
SCWIRVVQAWAGKGWGVLAMPRVGQEVLVNYVDGDLDRPMVTGIVYNGEN
PPPYRLPDHINYSGFVSRSLRFGQPQHASQLTFDDNRGNERIMLHAERDL
QRTVERNSATAVGQDKYDTVERTATEWINNHISYKDFSFSV
TGMSVSA
TGISVST
TGTSLSV
TGMSTSV
TGVSVGF
TLIGTSF
TGVSTSF
TGVGTSF
TGASNSL
TGVSNSM
TGCSSSF
TGTSNSM
TGSSHSM
TGMSTSI
TGHSMSQ
TGSSSSI
TGDSTSF
TGSSVSS
TGSSVST
TGVSTST
TGSSTST
TGCSVST
TGSSTST
TGNSVSM
TGNSTST
TGCSIST
TGSSIGT
VGSSIST
TGSSVST
TGSSIST
TGLSVSY
TGAQYSD

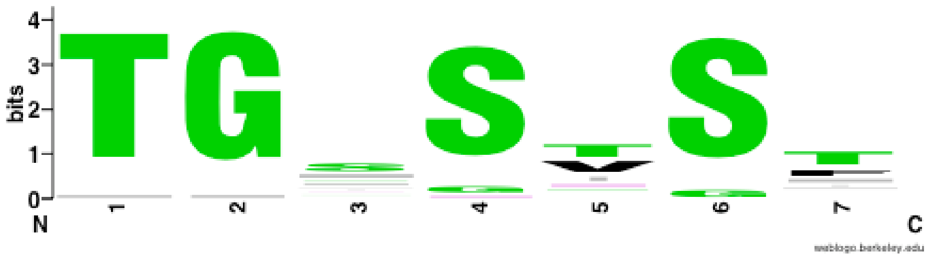

VGVDLKTVGMQSKN

BX470250_BB0793 True 787 BX470250A BX470250_GR
[('COG3889', 534, 667)]
ORG Bordetella bronchiseptica (strain NCTC 13252 / RB50 / ATCC BAA-588)
>BX470250_BB0793
METILAMAERIVRALTPLPPQALQFRSMHGHEGLSALYEFEVDLLAATHT
LELKSLLGKPVSLEIETGGAPRYLSGQATRCALVGREGDSARQYVYRVTM
RPWLWYLTQTSDSKIFQQMSVVDVLRQVLADYPFPVEYRLAGSYRRWEYC
VQYQETDFAFVSRLMEHEGIYYWFRHESGRHTLVLTDDITQHDECPGAAQ
LPYYGPDRATVPQEQYVSQWQVAEEITPDGFATVDYDFKKPAASLDAQSS
NPGAYEPGGLQVYEWLGGYTEPDQGERYSRIRLEALQAHGESVTGACNVR
AFAPGYLFTLRNHPRPAENRQYLIAQAHYRIQEGGYASGAEDAVFDIDFR
VLPATVPFRVARATPVPRTHGPQTATVVGQAGEEIWTDEYGRVKVHFHWD
RYGKKNENSSCWVRVSSPWAGGGFGGIQLPRVGDEVIVDFIGGYPDRPIV
IGRVYNASNMPPWDLPGNATQSGFLSRSKNGDRGTANALMFEDSAGAERI
WLHAERDMDCEVEANESHTVDGNRTTLIGGNDTLTVRGTRTTTIDGLDTE
TFNAGATRTV
TGEVSETT
TGNETRTF
NGDVTETV
NGVETRTV
NGDWKETI
TGEMTETR
TGDETRTV
TGAVTETI
TGDVTQTI
TGAVTQTQ
TGAHDITI
TGDQTSSI
TGAVSHTV
TGAYTQTV

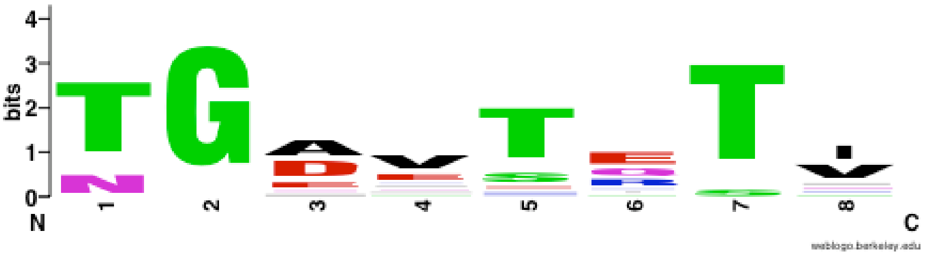

TDPVTVNANTSMTVVTPSWTVSSASQQAFWTANTLRGTPARLTVVGAAAD
FWGVRQQVYGGINSQWSTVKIDLAAFKNGSNGFESGQAGAQIKAIGAQIK
TGGAAVISRVINLFT

CP000011_BMAA1901 True 775 CP000011F CP000011_GR []
ORG Burkholderia mallei (strain ATCC 23344)
>CP000011_BMAA1901
MPNFSAARTVTVSGPAVPTTPVGEPALELSAIRGDETLSEIYSYTLDCLT
PPDPLLAHERAANLDLKAMIGKALTVTVQLEGMGSFVPGMPGMAGAANIG
AGAREISGIVTGARFEGQLNRQCRYRLTMRPWIYLADLRSDYRIFQNRSV
DEIVDEVLNAYSYSYDKRLSGRYPKLGYQVQYGETDFAFIQRLMQEHGIY
WFFEHTNQVHRMVLVDHLGAHKPVESAAYRTLRYYPPGHKIDAEYIDRFS
TEEHIRPGRWATGDFDFEKPNADLGVENALPRDTAHNGLERYEWPGDYTA
REHGEHLARVRMEQTRARGERASGGGNVRDIVCGTTFALAGHPHASANRE
YLVIGATFEATETVGASGPGAYRIDTSFVAQPATTAFRPPRTVRKPRTRG
PQTAIVTGPRGQDIWTDQYGRVKLKFHWDRSLVDDQNSSCWVRVSYAWSG
NNYGGVNIPRVGSEVVVDFEHGDPDRPLVTGQVYNALHMPPWPLPENATQ
SGFMTRSPAGGGENANMLRFEDKAGEEQVKLHAERNYDVSVERDATTAVG
RKHLTLVGVDLMPVMSTRSPLERLIDLLRTGGAASPAASGQPAAASRQPS
GQQQQHQQQHQQHQRQHQPYQQLKQQRQMQRAGGWQALAANVAKTLQEIV
AVLTDPFVTASVSTVQGASTSVVFGDAKGVHVGDTVKITSGDAHSEVNGT
AS
SKDVNSV
SLKGTST
SLTGVST
SETGTNI
VTTGLTV
STTGASV
STTGFSA
TETLVQVAKKTQECEFIKGIKISM

CP000573_BURPS1106A_A0251 True 775 CP000573F CP000573_GR
[]
ORG Burkholderia pseudomallei (strain 1106a)
>CP000573_BURPS1106A_A0251
MPNFSAARTVTVSGPAVPTTPVGEPALELSAIRGDETLSEIYSYTLDCLT
PPDPLLAHERAANLDLKAMIGKALTVTVQLEGMGSFVPGMPGMAGAANIG
AGAREISGIVTGARFEGQLNRQCRYRLTMRPWIYLADLRSDYRIFQNRSV
DEIVDEVLNAYSYSYDKRLSGRYPKLGYQVQYGETDFAFIQRLMQEHGIY
WFFEHTNQVHRMVLVDHLGAHKPVESAAYRTLRYYPPGHKIDAEYIDRFS
TEEHIRPGRWATGDFDFEKPNADLGVENALPRDTAHNGLERYEWPGDYTA
REHGEHLARVRMEQTRARGERASGGGNVRDIVCGTTFALAGHPHASANRE
YLVIGATFEATETGGASGPGAYRIDTSFVAQPATTAFRPPRTVRKPRTRG
PQTAIVTGPRGQDIWTDQYGRVKLKFHWDRSLVDDQNSSCWVRVSYAWSG
NNYGGVNIPRVGSEVVVDFEHGDPDRPLVTGQVYNALHMPPWPLPENATQ
SGFMTRSPAGGGENANMLRFEDKAGEEQVKLHAERNYDVSVERDATTAVG
RKHLTLVGVDLMPVMSTRSPLERLIDLLRTGGAASPAASGQPAAASRQPS
GQQQQHQQQHQQHQRQHQPYQQLKQQRQMQRAGGWQALAANVAKTLQEIV
AVLTDPFVTASVSTVQGASTSVVFGDAKGVHVGDTVKITSGDAHSEVNGT
AS
SKDVNSV
SLKGTST
SLTGVST
SETGTNI
VTTGLTV

STTGASV
STTGFSA
TETLVQVAKKTQECEFIKGIKISM
