# Supplementary material for: Dissecting the bacterial type VI secretion system by a genome wide in silico analysis: what can be learned from available microbial genomic resources?
Source: BMC Genomics. 2009 Mar 12;10:104. doi: 10.1186/1471-2164-10-104 (PMC2660368; doi:10.1186/1471-2164-10-104)
Supplement: Additional file 7 — Detailed description of all identified T6SS gene clusters. Archive containing the detailed description of each identified T6SS locus as an HTML file. [file 1471-2164-10-104-S7.tgz › LociHTML/HTML/AE003853C.html]

Locus AE003853C on Vibrio cholerae (serovar O1, strain ATCC 39315 / El Tor Inaba N16961) chromosome 2, complete sequence.

import namespace="svg" implementation="#AdobeSVG"?


# Locus AE003853C

# List of CDS in T6SS locus AE003853C

|  |  |  |  |  |  |  |  |  |
| --- | --- | --- | --- | --- | --- | --- | --- | --- |
| Name | from | to | direct | COG | e-value | COG cover | COG hit start | COG hit end |
| AE003853\_VC\_A0103 | 110871 | 112649 | True | COG0659 | 5e-111 | 96.0 | 1 | 534 |
| AE003853\_VC\_A0104 | 112691 | 113356 | False | COG0564 | 8e-56 | 78.0 | 62 | 289 |
| AE003853\_VC\_A0105 | 115142 | 115426 | True | COG4104 | 1e-11 | 98.0 | 1 | 97 |
| AE003853\_VC\_A0106 | 115341 | 116414 | True | - | - | - | - | - |
| AE003853\_VC\_A0107 | 116846 | 117352 | True | COG3516 | 9e-48 | 97.0 | 5 | 169 |
| AE003853\_VC\_A0108 | 117393 | 118871 | True | COG3517 | 0.0 | 99.0 | 1 | 493 |
| AE003853\_VC\_A0109 | 118874 | 119311 | True | COG3518 | 8e-33 | 98.0 | 1 | 154 |
| AE003853\_VC\_A0110 | 119317 | 121086 | True | COG3519 | 0.0 | 99.0 | 3 | 621 |
| AE003853\_VC\_A0111 | 121050 | 122066 | True | COG3520 | 4e-101 | 98.0 | 3 | 332 |
| AE003853\_VC\_A0112 | 122069 | 123556 | True | COG3456 | 1e-120 | 100.0 | 1 | 430 |
| AE003853\_VC\_A0113 | 123559 | 124035 | True | COG3521 | 1e-47 | 96.0 | 6 | 158 |
| AE003853\_VC\_A0114 | 124042 | 125376 | True | COG3522 | 2e-166 | 100.0 | 1 | 446 |
| AE003853\_VC\_A0115 | 125379 | 126152 | True | COG3455 | 5e-83 | 98.0 | 1 | 259 |
| AE003853\_VC\_A0116 | 126178 | 128787 | True | COG0542 | 0.0 | 100.0 | 1 | 786 |
| AE003853\_VC\_A0117 | 128790 | 130382 | True | COG3829 | 3e-94 | 63.0 | 207 | 559 |
| AE003853\_VC\_A0118 | 130352 | 131035 | True | - | - | - | - | - |
| AE003853\_VC\_A0119 | 131045 | 132454 | True | COG3515 | 2e-41 | 81.0 | 1 | 283 |
| AE003853\_VC\_A0120 | 132470 | 136015 | True | COG3523 | 0.0 | 100.0 | 1 | 1188 |
| AE003853\_VC\_A0121 | 136063 | 137328 | True | COG3515 | 9e-51 | 98.0 | 6 | 346 |
| AE003853\_VC\_A0122 | 137379 | 137621 | True | - | - | - | - | - |
| AE003853\_VC\_A0123 | 137581 | 140634 | True | COG3501 | 6e-172 | 96.0 | 11 | 539 |
| AE003853\_VC\_A0124 | 140631 | 140999 | True | - | - | - | - | - |
| AE003853\_VC\_A0125 | 141147 | 141407 | False | - | - | - | - | - |
| AE003853\_VC\_A0126 | 141382 | 141591 | True | - | - | - | - | - |
| AE003853\_VC\_A0127 | 141640 | 142110 | True | COG1869 | 4e-50 | 100.0 | 1 | 135 |
| AE003853\_VC\_A0128 | 142137 | 143639 | True | COG1129 | 0.0 | 99.0 | 4 | 499 |
| AE003853\_VC\_A0129 | 143636 | 144634 | True | COG1172 | 6e-74 | 95.0 | 7 | 307 |
| AE003853\_VC\_A0130 | 144693 | 145577 | True | COG1879 | 2e-50 | 93.0 | 8 | 309 |
