# Supplementary material for: Dissecting the bacterial type VI secretion system by a genome wide in silico analysis: what can be learned from available microbial genomic resources?
Source: BMC Genomics. 2009 Mar 12;10:104. doi: 10.1186/1471-2164-10-104 (PMC2660368; doi:10.1186/1471-2164-10-104)
Supplement: Additional file 7 — Detailed description of all identified T6SS gene clusters. Archive containing the detailed description of each identified T6SS locus as an HTML file. [file 1471-2164-10-104-S7.tgz › LociHTML/HTML/AE004091A.html]

Locus AE004091A on Pseudomonas aeruginosa (strain LMG 12228 / ATCC 15692 / PRS 101 / 1C / PAO1) chromosome, complete sequence.

import namespace="svg" implementation="#AdobeSVG"?


# Locus AE004091A

# List of CDS in T6SS locus AE004091A

|  |  |  |  |  |  |  |  |  |
| --- | --- | --- | --- | --- | --- | --- | --- | --- |
| Name | from | to | direct | COG | e-value | COG cover | COG hit start | COG hit end |
| AE004091\_PA0072 | 85085 | 86284 | False | COG4591 | 6e-10 | 99.0 | 3 | 408 |
| AE004091\_PA0073 | 86284 | 87003 | False | COG1136 | 3e-51 | 92.0 | 1 | 209 |
| AE004091\_PA0074 | 87000 | 90098 | False | COG0515 | 8e-48 | 96.0 | 1 | 369 |
| AE004091\_PA0075 | 90106 | 90834 | False | COG0631 | 5e-56 | 95.0 | 1 | 250 |
| AE004091\_PA0076 | 90844 | 91524 | False | COG3913 | 2e-77 | 98.0 | 5 | 227 |
| AE004091\_PA0077 | 91521 | 94826 | False | COG3523 | 0.0 | 93.0 | 73 | 1187 |
| AE004091\_PA0078 | 95048 | 96397 | False | COG3455 | 8e-80 | 100.0 | 1 | 262 |
| AE004091\_PA0078 | 95048 | 96397 | False | COG1360 | 3e-32 | 58.0 | 103 | 244 |
| AE004091\_PA0079 | 96404 | 97738 | False | COG3522 | 1e-163 | 100.0 | 1 | 446 |
| AE004091\_PA0080 | 97754 | 98218 | False | COG3521 | 2e-48 | 96.0 | 6 | 158 |
| AE004091\_PA0081 | 98263 | 99756 | False | COG3456 | 1e-103 | 100.0 | 1 | 430 |
| AE004091\_PA0082 | 100124 | 101158 | True | COG3515 | 3e-44 | 99.0 | 1 | 345 |
| AE004091\_PA0083 | 101247 | 101765 | True | COG3516 | 2e-58 | 99.0 | 2 | 169 |
| AE004091\_PA0084 | 101778 | 103274 | True | COG3517 | 0.0 | 100.0 | 1 | 495 |
| AE004091\_PA0085 | 103350 | 103838 | True | COG3157 | 5e-40 | 100.0 | 1 | 162 |
| AE004091\_PA0086 | 104006 | 104851 | True | COG4455 | 3e-94 | 100.0 | 1 | 273 |
| AE004091\_PA0087 | 104853 | 105362 | True | COG3518 | 1e-34 | 100.0 | 1 | 157 |
| AE004091\_PA0088 | 105359 | 107218 | True | COG3519 | 0.0 | 100.0 | 1 | 621 |
| AE004091\_PA0089 | 107182 | 108228 | True | COG3520 | 7e-102 | 99.0 | 2 | 335 |
| AE004091\_PA0090 | 108221 | 110929 | True | COG0542 | 0.0 | 100.0 | 1 | 786 |
| AE004091\_PA0091 | 110976 | 112907 | True | COG3501 | 0.0 | 98.0 | 7 | 549 |
| AE004091\_PA0092 | 113022 | 113306 | False | - | - | - | - | - |
| AE004091\_PA0093 | 113303 | 114595 | False | - | - | - | - | - |
| AE004091\_PA0094 | 114611 | 115045 | False | COG5435 | 1e-44 | 99.0 | 1 | 146 |
| AE004091\_PA0095 | 115299 | 117524 | True | COG3501 | 0.0 | 99.0 | 4 | 550 |
| AE004091\_PA0096 | 117552 | 118001 | True | - | - | - | - | - |
| AE004091\_PA0097 | 117931 | 119130 | True | COG5351 | 5e-157 | 100.0 | 1 | 367 |
| AE004091\_PA0098 | 119127 | 120164 | True | COG0304 | 8e-36 | 83.0 | 34 | 377 |
| AE004091\_PA0099 | 120164 | 121324 | True | - | - | - | - | - |
| AE004091\_PA0100 | 121346 | 122266 | True | - | - | - | - | - |
| AE004091\_PA0101 | 122248 | 123495 | True | - | - | - | - | - |
