# Supplementary material for: Dissecting the bacterial type VI secretion system by a genome wide in silico analysis: what can be learned from available microbial genomic resources?
Source: BMC Genomics. 2009 Mar 12;10:104. doi: 10.1186/1471-2164-10-104 (PMC2660368; doi:10.1186/1471-2164-10-104)
Supplement: Additional file 7 — Detailed description of all identified T6SS gene clusters. Archive containing the detailed description of each identified T6SS locus as an HTML file. [file 1471-2164-10-104-S7.tgz › LociHTML/HTML/AE004091D.html]

Locus AE004091D on Pseudomonas aeruginosa (strain LMG 12228 / ATCC 15692 / PRS 101 / 1C / PAO1) chromosome, complete sequence.

import namespace="svg" implementation="#AdobeSVG"?


# Locus AE004091D

# List of CDS in T6SS locus AE004091D

|  |  |  |  |  |  |  |  |  |
| --- | --- | --- | --- | --- | --- | --- | --- | --- |
| Name | from | to | direct | COG | e-value | COG cover | COG hit start | COG hit end |
| AE004091\_PA1651 | 1797958 | 1799154 | False | COG3135 | 2e-121 | 98.0 | 3 | 399 |
| AE004091\_PA1652 | 1799385 | 1800020 | True | - | - | - | - | - |
| AE004091\_PA1653 | 1800071 | 1800547 | False | COG1846 | 2e-08 | 79.0 | 14 | 113 |
| AE004091\_PA1654 | 1800629 | 1801795 | True | COG1167 | 1e-93 | 85.0 | 66 | 459 |
| AE004091\_PA1655 | 1801851 | 1802453 | True | COG0625 | 3e-38 | 93.0 | 1 | 198 |
| AE004091\_PA1656 | 1803626 | 1805182 | True | COG3515 | 5e-38 | 100.0 | 1 | 346 |
| AE004091\_PA1657 | 1805218 | 1805724 | True | COG3516 | 3e-46 | 97.0 | 5 | 169 |
| AE004091\_PA1658 | 1805753 | 1807228 | True | COG3517 | 0.0 | 99.0 | 1 | 493 |
| AE004091\_PA1659 | 1807241 | 1807648 | True | COG3518 | 5e-29 | 89.0 | 14 | 154 |
| AE004091\_PA1660 | 1808193 | 1809773 | True | COG3519 | 1e-149 | 88.0 | 75 | 621 |
| AE004091\_PA1661 | 1809737 | 1810744 | True | COG3520 | 1e-88 | 99.0 | 1 | 333 |
| AE004091\_PA1662 | 1810751 | 1813384 | True | COG0542 | 0.0 | 97.0 | 2 | 766 |
| AE004091\_PA1663 | 1813395 | 1814906 | True | COG3604 | 8e-153 | 96.0 | 21 | 550 |
| AE004091\_PA1664 | 1814995 | 1815135 | True | - | - | - | - | - |
| AE004091\_PA1665 | 1815153 | 1816346 | True | COG3456 | 4e-92 | 100.0 | 1 | 430 |
| AE004091\_PA1666 | 1816352 | 1816858 | True | COG3521 | 2e-42 | 97.0 | 5 | 159 |
| AE004091\_PA1667 | 1816855 | 1818186 | True | COG3522 | 5e-153 | 99.0 | 2 | 446 |
| AE004091\_PA1668 | 1818189 | 1819058 | True | COG3455 | 3e-76 | 100.0 | 1 | 262 |
| AE004091\_PA1669 | 1819074 | 1822601 | True | COG3523 | 0.0 | 99.0 | 7 | 1187 |
| AE004091\_PA1670 | 1822601 | 1823329 | True | COG0631 | 4e-59 | 95.0 | 2 | 250 |
| AE004091\_PA1671 | 1823326 | 1824315 | True | COG0515 | 2e-36 | 73.0 | 2 | 284 |
| AE004091\_PA1672 | 1824343 | 1824723 | False | COG3324 | 6e-30 | 95.0 | 7 | 127 |
| AE004091\_PA1673 | 1824969 | 1825430 | True | COG2703 | 6e-34 | 100.0 | 1 | 144 |
| AE004091\_PA1674 | 1825495 | 1826040 | False | COG0302 | 2e-78 | 92.0 | 14 | 193 |
| AE004091\_PA1675 | 1826118 | 1826675 | False | COG2840 | 3e-48 | 96.0 | 7 | 184 |
| AE004091\_PA1676 | 1826732 | 1827052 | False | - | - | - | - | - |
| AE004091\_PA1677 | 1827225 | 1827821 | False | COG1335 | 3e-20 | 98.0 | 5 | 205 |
