# Supplementary material for: Dissecting the bacterial type VI secretion system by a genome wide in silico analysis: what can be learned from available microbial genomic resources?
Source: BMC Genomics. 2009 Mar 12;10:104. doi: 10.1186/1471-2164-10-104 (PMC2660368; doi:10.1186/1471-2164-10-104)
Supplement: Additional file 7 — Detailed description of all identified T6SS gene clusters. Archive containing the detailed description of each identified T6SS locus as an HTML file. [file 1471-2164-10-104-S7.tgz › LociHTML/HTML/AE004091E.html]

Locus AE004091E on Pseudomonas aeruginosa (strain LMG 12228 / ATCC 15692 / PRS 101 / 1C / PAO1) chromosome, complete sequence.

import namespace="svg" implementation="#AdobeSVG"?


# Locus AE004091E

# List of CDS in T6SS locus AE004091E

|  |  |  |  |  |  |  |  |  |
| --- | --- | --- | --- | --- | --- | --- | --- | --- |
| Name | from | to | direct | COG | e-value | COG cover | COG hit start | COG hit end |
| AE004091\_PA2353 | 2599893 | 2601047 | False | COG1485 | 8e-116 | 98.0 | 1 | 363 |
| AE004091\_PA2354 | 2601219 | 2602349 | False | COG3829 | 3e-96 | 53.0 | 250 | 547 |
| AE004091\_PA2355 | 2602346 | 2603530 | False | COG1960 | 8e-38 | 100.0 | 1 | 393 |
| AE004091\_PA2356 | 2603560 | 2604705 | False | COG2141 | 1e-55 | 98.0 | 1 | 330 |
| AE004091\_PA2357 | 2604715 | 2605275 | False | COG0431 | 6e-26 | 95.0 | 2 | 176 |
| AE004091\_PA2358 | 2605435 | 2605824 | False | - | - | - | - | - |
| AE004091\_PA2359 | 2605937 | 2607022 | False | COG1221 | 2e-95 | 82.0 | 66 | 399 |
| AE004091\_PA2360 | 2607132 | 2608232 | False | COG3515 | 2e-48 | 100.0 | 1 | 346 |
| AE004091\_PA2361 | 2608229 | 2612044 | False | COG3523 | 0.0 | 99.0 | 2 | 1187 |
| AE004091\_PA2362 | 2612041 | 2612799 | False | COG3455 | 3e-51 | 95.0 | 13 | 262 |
| AE004091\_PA2363 | 2612817 | 2614148 | False | COG3522 | 4e-106 | 100.0 | 1 | 446 |
| AE004091\_PA2364 | 2614208 | 2614684 | False | - | - | - | - | - |
| AE004091\_PA2365 | 2614893 | 2615438 | True | COG3516 | 3e-53 | 100.0 | 1 | 169 |
| AE004091\_PA2366 | 2615461 | 2616945 | True | COG3517 | 0.0 | 99.0 | 2 | 495 |
| AE004091\_PA2367 | 2617019 | 2617516 | True | COG3157 | 2e-39 | 99.0 | 2 | 162 |
| AE004091\_PA2368 | 2617529 | 2617954 | True | COG3518 | 5e-26 | 95.0 | 8 | 157 |
| AE004091\_PA2369 | 2617938 | 2619731 | True | COG3519 | 3e-180 | 100.0 | 1 | 621 |
| AE004091\_PA2370 | 2619695 | 2620711 | True | COG3520 | 3e-79 | 100.0 | 1 | 335 |
| AE004091\_PA2371 | 2620713 | 2623262 | True | COG0542 | 0.0 | 96.0 | 1 | 761 |
| AE004091\_PA2372 | 2623284 | 2623856 | True | - | - | - | - | - |
| AE004091\_PA2373 | 2624204 | 2626210 | True | COG3501 | 3e-158 | 98.0 | 6 | 544 |
| AE004091\_PA2374 | 2626221 | 2626757 | True | COG2849 | 3e-31 | 70.0 | 70 | 230 |
| AE004091\_PA2375 | 2626780 | 2627175 | False | - | - | - | - | - |
| AE004091\_PA2376 | 2627452 | 2628093 | True | COG2197 | 3e-39 | 98.0 | 2 | 208 |
| AE004091\_PA2377 | 2628225 | 2629499 | True | COG1840 | 4e-27 | 95.0 | 1 | 286 |
| AE004091\_PA2378 | 2629916 | 2632231 | False | COG1529 | 3e-99 | 99.0 | 3 | 730 |
