# Supplementary material for: Dissecting the bacterial type VI secretion system by a genome wide in silico analysis: what can be learned from available microbial genomic resources?
Source: BMC Genomics. 2009 Mar 12;10:104. doi: 10.1186/1471-2164-10-104 (PMC2660368; doi:10.1186/1471-2164-10-104)
Supplement: Additional file 7 — Detailed description of all identified T6SS gene clusters. Archive containing the detailed description of each identified T6SS locus as an HTML file. [file 1471-2164-10-104-S7.tgz › LociHTML/HTML/AE005174A.html]

Locus AE005174A on Escherichia coli (strain EDL933 / ATCC 700927 / O157:H7 / EHEC) chromosome, complete sequence.

import namespace="svg" implementation="#AdobeSVG"?


# Locus AE005174A

# List of CDS in T6SS locus AE005174A

|  |  |  |  |  |  |  |  |  |
| --- | --- | --- | --- | --- | --- | --- | --- | --- |
| Name | from | to | direct | COG | e-value | COG cover | COG hit start | COG hit end |
| AE005174\_Z0237 | 238349 | 239089 | True | COG2226 | 1e-07 | 26.0 | 106 | 168 |
| AE005174\_Z0239 | 239086 | 239553 | False | COG0328 | 1e-55 | 99.0 | 2 | 154 |
| AE005174\_Z0241 | 239618 | 240349 | True | COG0847 | 5e-51 | 95.0 | 8 | 240 |
| AE005174\_Z0243 | 240887 | 241687 | True | - | - | - | - | - |
| AE005174\_Z0244 | 241872 | 242168 | False | - | - | - | - | - |
| AE005174\_Z0245 | 242165 | 242620 | False | - | - | - | - | - |
| AE005174\_Z0246 | 242617 | 243213 | False | - | - | - | - | - |
| AE005174\_Z0247 | 243292 | 243513 | False | - | - | - | - | - |
| AE005174\_Z0248 | 243534 | 244013 | False | COG3157 | 4e-43 | 98.0 | 1 | 159 |
| AE005174\_Z0249 | 243979 | 245478 | False | COG3515 | 3e-60 | 96.0 | 12 | 346 |
| AE005174\_Z0250 | 245399 | 248833 | False | COG3523 | 0.0 | 98.0 | 15 | 1188 |
| AE005174\_Z0251 | 248970 | 249764 | False | COG3515 | 3e-36 | 76.0 | 19 | 284 |
| AE005174\_Z0252 | 249761 | 250381 | False | - | - | - | - | - |
| AE005174\_Z0253 | 250386 | 251129 | False | - | - | - | - | - |
| AE005174\_Z0254 | 251126 | 253897 | False | COG0542 | 1e-122 | 53.0 | 1 | 423 |
| AE005174\_Z0254 | 251126 | 253897 | False | COG0542 | 7e-98 | 46.0 | 422 | 786 |
| AE005174\_Z0255 | 253906 | 254667 | False | COG3455 | 8e-83 | 97.0 | 6 | 260 |
| AE005174\_Z0256 | 254672 | 256003 | False | COG3522 | 6e-164 | 100.0 | 1 | 446 |
| AE005174\_Z0257 | 256006 | 256530 | False | COG3521 | 5e-40 | 100.0 | 1 | 159 |
| AE005174\_Z0258 | 256527 | 257828 | False | COG3456 | 4e-120 | 100.0 | 1 | 430 |
| AE005174\_Z0259 | 257832 | 258914 | False | COG3520 | 5e-104 | 99.0 | 1 | 332 |
| AE005174\_Z0260 | 258878 | 260728 | False | COG3519 | 0.0 | 99.0 | 2 | 621 |
| AE005174\_Z0261 | 260732 | 261145 | False | COG3518 | 2e-26 | 96.0 | 3 | 154 |
| AE005174\_Z0262 | 261236 | 262627 | False | COG3517 | 0.0 | 93.0 | 1 | 465 |
| AE005174\_Z0263 | 262678 | 262902 | False | - | - | - | - | - |
| AE005174\_Z0264 | 262937 | 263437 | False | COG3516 | 2e-49 | 98.0 | 2 | 167 |
| AE005174\_Z0265 | 263864 | 264007 | True | - | - | - | - | - |
| AE005174\_Z0266 | 264134 | 264652 | True | COG3157 | 9e-52 | 98.0 | 1 | 160 |
| AE005174\_Z0267 | 264862 | 267003 | True | COG3501 | 9e-175 | 98.0 | 8 | 550 |
| AE005174\_Z0268 | 267079 | 271293 | True | COG3209 | 2e-107 | 98.0 | 1 | 786 |
| AE005174\_Z0268 | 267079 | 271293 | True | COG3209 | 3e-19 | 56.0 | 12 | 463 |
| AE005174\_Z0269 | 271296 | 271907 | True | - | - | - | - | - |
