# Supplementary material for: Dissecting the bacterial type VI secretion system by a genome wide in silico analysis: what can be learned from available microbial genomic resources?
Source: BMC Genomics. 2009 Mar 12;10:104. doi: 10.1186/1471-2164-10-104 (PMC2660368; doi:10.1186/1471-2164-10-104)
Supplement: Additional file 7 — Detailed description of all identified T6SS gene clusters. Archive containing the detailed description of each identified T6SS locus as an HTML file. [file 1471-2164-10-104-S7.tgz › LociHTML/HTML/AE005674A.html]

Locus AE005674A on Shigella flexneri (serovar 2a, strain 301) chromosome, complete sequence.

import namespace="svg" implementation="#AdobeSVG"?


# Locus AE005674A

# List of CDS in T6SS locus AE005674A

|  |  |  |  |  |  |  |  |  |
| --- | --- | --- | --- | --- | --- | --- | --- | --- |
| Name | from | to | direct | COG | e-value | COG cover | COG hit start | COG hit end |
| AE005674\_SF0196 | 224843 | 225463 | True | COG2226 | 7e-18 | 44.0 | 59 | 164 |
| AE005674\_SF0197 | 225511 | 226857 | False | COG1388 | 8e-09 | 95.0 | 1 | 119 |
| AE005674\_SF0197 | 225511 | 226857 | False | COG0741 | 7e-12 | 93.0 | 1 | 278 |
| AE005674\_SF0198 | 226929 | 227684 | False | COG0491 | 1e-26 | 92.0 | 19 | 252 |
| AE005674\_SF0199 | 227715 | 228440 | True | COG2226 | 7e-08 | 26.0 | 106 | 168 |
| AE005674\_SF0201 | 228437 | 228904 | False | COG0328 | 1e-55 | 99.0 | 2 | 154 |
| AE005674\_SF0200 | 228969 | 229700 | True | COG0847 | 4e-51 | 95.0 | 8 | 240 |
| AE005674\_SF0202 | 230040 | 231086 | False | COG3515 | 6e-32 | 96.0 | 7 | 340 |
| AE005674\_SF0203 | 231354 | 231659 | False | COG3520 | 9e-21 | 28.0 | 13 | 109 |
| AE005674\_SF0204 | 231656 | 233539 | False | COG3519 | 0.0 | 100.0 | 1 | 621 |
| AE005674\_SF0205 | 233555 | 234049 | False | COG3518 | 3e-31 | 95.0 | 5 | 154 |
| AE005674\_SF0206 | 234244 | 234876 | False | COG4455 | 6e-68 | 75.0 | 2 | 208 |
| AE005674\_SF0207 | 234863 | 235240 | False | - | - | - | - | - |
| AE005674\_SF0208 | 235478 | 235777 | False | - | - | - | - | - |
| AE005674\_SF0209 | 235830 | 236240 | True | COG2963 | 7e-10 | 99.0 | 1 | 115 |
| AE005674\_SF0210 | 236198 | 237103 | True | COG2801 | 3e-12 | 98.0 | 3 | 230 |
| AE005674\_SF0211 | 237160 | 237447 | False | - | - | - | - | - |
| AE005674\_SF0212 | 237461 | 237982 | False | COG2801 | 3e-17 | 64.0 | 82 | 231 |
| AE005674\_SF0213 | 237951 | 238301 | False | - | - | - | - | - |
| AE005674\_SF0214 | 238328 | 238666 | False | COG2963 | 4e-11 | 86.0 | 2 | 101 |
| AE005674\_SF0215 | 238841 | 239743 | True | COG5565 | 5e-10 | 55.0 | 25 | 68 |
| AE005674\_SF0216 | 239744 | 241909 | True | - | - | - | - | - |
