# Supplementary material for: Dissecting the bacterial type VI secretion system by a genome wide in silico analysis: what can be learned from available microbial genomic resources?
Source: BMC Genomics. 2009 Mar 12;10:104. doi: 10.1186/1471-2164-10-104 (PMC2660368; doi:10.1186/1471-2164-10-104)
Supplement: Additional file 7 — Detailed description of all identified T6SS gene clusters. Archive containing the detailed description of each identified T6SS locus as an HTML file. [file 1471-2164-10-104-S7.tgz › LociHTML/HTML/AE006468A.html]

Locus AE006468A on Salmonella typhimurium (strain ATCC 700720 / SGSC1412 / LT2) chromosome, complete sequence.

import namespace="svg" implementation="#AdobeSVG"?


# Locus AE006468A

# List of CDS in T6SS locus AE006468A

|  |  |  |  |  |  |  |  |  |
| --- | --- | --- | --- | --- | --- | --- | --- | --- |
| Name | from | to | direct | COG | e-value | COG cover | COG hit start | COG hit end |
| AE006468\_STM0259 | 299097 | 299867 | True | COG2226 | 5e-22 | 61.0 | 18 | 164 |
| AE006468\_STM0260 | 299923 | 301290 | False | COG1388 | 1e-07 | 95.0 | 1 | 119 |
| AE006468\_STM0260 | 299923 | 301290 | False | COG1388 | 9e-09 | 93.0 | 1 | 116 |
| AE006468\_STM0260 | 299923 | 301290 | False | COG0741 | 4e-13 | 95.0 | 1 | 284 |
| AE006468\_STM0261 | 301362 | 302117 | False | COG0491 | 9e-26 | 88.0 | 19 | 241 |
| AE006468\_STM0262 | 302152 | 302874 | True | COG2226 | 2e-07 | 42.0 | 81 | 181 |
| AE006468\_STM0263 | 302871 | 303338 | False | COG0328 | 1e-54 | 99.0 | 2 | 154 |
| AE006468\_STM0264 | 303402 | 304133 | True | COG0847 | 3e-51 | 95.0 | 8 | 240 |
| AE006468\_STM0266 | 304665 | 305720 | False | COG3515 | 1e-44 | 97.0 | 3 | 340 |
| AE006468\_STM0267 | 305731 | 306726 | False | COG3520 | 9e-97 | 97.0 | 11 | 335 |
| AE006468\_STM0268 | 306723 | 308606 | False | COG3519 | 0.0 | 100.0 | 1 | 621 |
| AE006468\_STM0269 | 308622 | 309116 | False | COG3518 | 7e-36 | 98.0 | 1 | 154 |
| AE006468\_STM0270 | 309113 | 309937 | False | COG4455 | 2e-109 | 100.0 | 1 | 273 |
| AE006468\_STM0271 | 309924 | 310826 | False | - | - | - | - | - |
| AE006468\_STM0272 | 311194 | 313833 | True | COG0542 | 0.0 | 99.0 | 1 | 781 |
| AE006468\_STM0273 | 313933 | 314475 | True | COG3516 | 2e-57 | 99.0 | 2 | 169 |
| AE006468\_STM0274 | 314499 | 316007 | True | COG3517 | 0.0 | 100.0 | 1 | 495 |
| AE006468\_STM0274.1 | 316070 | 316180 | True | - | - | - | - | - |
| AE006468\_STM0275 | 316307 | 316639 | True | - | - | - | - | - |
| AE006468\_STM0276 | 316893 | 317378 | True | COG3157 | 3e-44 | 100.0 | 1 | 162 |
| AE006468\_STM0277 | 317683 | 318168 | True | - | - | - | - | - |
| AE006468\_STM0278 | 318153 | 318536 | True | - | - | - | - | - |
| AE006468\_STM0279 | 318679 | 319164 | True | COG3157 | 3e-43 | 100.0 | 1 | 162 |
| AE006468\_STM0280 | 319231 | 319767 | True | COG3521 | 4e-43 | 98.0 | 3 | 159 |
| AE006468\_STM0281 | 319771 | 321114 | True | COG3522 | 1e-153 | 100.0 | 1 | 446 |
| AE006468\_STM0282 | 321111 | 322415 | True | COG3455 | 6e-72 | 99.0 | 1 | 261 |
| AE006468\_STM0282 | 321111 | 322415 | True | COG1360 | 6e-32 | 58.0 | 101 | 242 |
| AE006468\_STM0283 | 322420 | 323193 | True | - | - | - | - | - |
| AE006468\_STM0284 | 323396 | 323827 | True | - | - | - | - | - |
| AE006468\_STM0285 | 323861 | 327730 | True | COG3523 | 0.0 | 99.0 | 2 | 1188 |
| AE006468\_STM0286 | 327730 | 328518 | True | - | - | - | - | - |
| AE006468\_STM0287 | 328515 | 328931 | True | COG4893 | 5e-50 | 100.0 | 1 | 123 |
| AE006468\_STM0288 | 328955 | 329476 | True | - | - | - | - | - |
| AE006468\_STM0289 | 329873 | 332062 | True | COG3501 | 6e-163 | 100.0 | 1 | 550 |
| AE006468\_STM0290 | 332086 | 332532 | True | COG5435 | 1e-42 | 98.0 | 1 | 145 |
| AE006468\_STM0291 | 332551 | 336645 | True | COG3209 | 4e-72 | 96.0 | 1 | 769 |
