# Supplementary material for: Dissecting the bacterial type VI secretion system by a genome wide in silico analysis: what can be learned from available microbial genomic resources?
Source: BMC Genomics. 2009 Mar 12;10:104. doi: 10.1186/1471-2164-10-104 (PMC2660368; doi:10.1186/1471-2164-10-104)
Supplement: Additional file 7 — Detailed description of all identified T6SS gene clusters. Archive containing the detailed description of each identified T6SS locus as an HTML file. [file 1471-2164-10-104-S7.tgz › LociHTML/HTML/AE007870A.html]

Locus AE007870A on Agrobacterium tumefaciens (strain C58 / ATCC 33970, sub\_strain Cereon) chromosome linear, complete sequence.

import namespace="svg" implementation="#AdobeSVG"?


# Locus AE007870A

# List of CDS in T6SS locus AE007870A

|  |  |  |  |  |  |  |  |  |
| --- | --- | --- | --- | --- | --- | --- | --- | --- |
| Name | from | to | direct | COG | e-value | COG cover | COG hit start | COG hit end |
| AE007870\_AGR\_L\_1027 | 585922 | 587868 | True | COG5001 | 0.0 | 99.0 | 1 | 661 |
| AE007870\_AGR\_L\_1028 | 588007 | 588312 | False | COG4104 | 5e-16 | 96.0 | 3 | 97 |
| AE007870\_AGR\_L\_1029 | 588325 | 588999 | False | COG5620 | 7e-12 | 93.0 | 14 | 200 |
| AE007870\_AGR\_L\_1030 | 589027 | 589863 | False | - | - | - | - | - |
| AE007870\_AGR\_L\_1031 | 589860 | 590822 | False | - | - | - | - | - |
| AE007870\_AGR\_L\_1033 | 590819 | 593269 | False | COG3501 | 3e-155 | 100.0 | 1 | 550 |
| AE007870\_AGR\_L\_1035 | 593284 | 593796 | False | - | - | - | - | - |
| AE007870\_AGR\_L\_1036 | 593796 | 594245 | False | - | - | - | - | - |
| AE007870\_AGR\_L\_1037 | 594292 | 594768 | False | COG3157 | 4e-31 | 98.0 | 3 | 162 |
| AE007870\_AGR\_L\_1041 | 594826 | 597420 | False | COG0542 | 0.0 | 97.0 | 18 | 786 |
| AE007870\_AGR\_L\_1042 | 597725 | 598780 | True | COG3515 | 2e-52 | 100.0 | 1 | 346 |
| AE007870\_AGR\_L\_1045 | 598819 | 599355 | True | COG3516 | 1e-55 | 100.0 | 1 | 169 |
| AE007870\_AGR\_L\_1047 | 599449 | 600930 | True | COG3517 | 0.0 | 99.0 | 1 | 494 |
| AE007870\_AGR\_L\_1048 | 600943 | 602379 | True | COG3517 | 3e-170 | 94.0 | 26 | 495 |
| AE007870\_AGR\_L\_1050 | 602376 | 603200 | True | COG4455 | 2e-100 | 99.0 | 2 | 273 |
| AE007870\_AGR\_L\_1052 | 603148 | 603702 | True | COG3518 | 7e-33 | 100.0 | 1 | 157 |
| AE007870\_AGR\_L\_1054 | 603695 | 605476 | True | COG3519 | 6e-180 | 100.0 | 1 | 621 |
| AE007870\_AGR\_L\_1055 | 605486 | 606490 | True | COG3520 | 4e-79 | 100.0 | 1 | 335 |
| AE007870\_AGR\_L\_1057 | 606501 | 607700 | True | COG3456 | 5e-99 | 98.0 | 3 | 426 |
| AE007870\_AGR\_L\_1059 | 607690 | 609030 | True | COG3522 | 2e-161 | 100.0 | 1 | 446 |
| AE007870\_AGR\_L\_1060 | 609012 | 610532 | True | COG3455 | 1e-72 | 98.0 | 5 | 262 |
| AE007870\_AGR\_L\_1060 | 609012 | 610532 | True | COG1360 | 1e-31 | 88.0 | 30 | 244 |
| AE007870\_AGR\_L\_1062 | 610529 | 614008 | True | COG3523 | 0.0 | 99.0 | 1 | 1187 |
| AE007870\_AGR\_L\_1064 | 613851 | 615506 | True | COG0631 | 7e-38 | 97.0 | 1 | 255 |
| AE007870\_AGR\_L\_1064 | 613851 | 615506 | True | COG3913 | 2e-72 | 99.0 | 1 | 226 |
| AE007870\_AGR\_L\_1065 | 615481 | 616398 | True | COG0515 | 6e-15 | 47.0 | 24 | 205 |
| AE007870\_AGR\_L\_1067 | 616407 | 617309 | False | COG1596 | 2e-34 | 79.0 | 51 | 239 |
| AE007870\_AGR\_L\_1070 | 617688 | 618197 | False | - | - | - | - | - |
| AE007870\_AGR\_L\_1071 | 618240 | 619790 | True | COG2814 | 1e-11 | 80.0 | 63 | 378 |
| AE007870\_AGR\_L\_1073 | 619876 | 620688 | False | COG1028 | 8e-39 | 100.0 | 1 | 251 |
