# Supplementary material for: Dissecting the bacterial type VI secretion system by a genome wide in silico analysis: what can be learned from available microbial genomic resources?
Source: BMC Genomics. 2009 Mar 12;10:104. doi: 10.1186/1471-2164-10-104 (PMC2660368; doi:10.1186/1471-2164-10-104)
Supplement: Additional file 7 — Detailed description of all identified T6SS gene clusters. Archive containing the detailed description of each identified T6SS locus as an HTML file. [file 1471-2164-10-104-S7.tgz › LociHTML/HTML/AE008689B.html]

Locus AE008689B on Agrobacterium tumefaciens (strain C58 / ATCC 33970, sub\_strain Dupont) chromosome linear, complete sequence.

import namespace="svg" implementation="#AdobeSVG"?


# Locus AE008689B

# List of CDS in T6SS locus AE008689B

|  |  |  |  |  |  |  |  |  |
| --- | --- | --- | --- | --- | --- | --- | --- | --- |
| Name | from | to | direct | COG | e-value | COG cover | COG hit start | COG hit end |
| AE008689\_Atu4325 | 1454155 | 1454880 | True | COG1028 | 6e-37 | 100.0 | 1 | 251 |
| AE008689\_Atu4326 | 1454966 | 1456516 | False | COG2814 | 1e-11 | 80.0 | 63 | 378 |
| AE008689\_Atu4327 | 1456646 | 1457068 | True | - | - | - | - | - |
| AE008689\_Atu4328 | 1457185 | 1457385 | False | - | - | - | - | - |
| AE008689\_Atu4329 | 1457447 | 1458349 | True | COG1596 | 2e-34 | 79.0 | 51 | 239 |
| AE008689\_Atu4330 | 1458358 | 1459170 | False | COG0515 | 3e-15 | 47.0 | 24 | 205 |
| AE008689\_Atu4331 | 1459250 | 1460665 | False | COG3913 | 4e-72 | 99.0 | 1 | 226 |
| AE008689\_Atu4331 | 1459250 | 1460665 | False | COG0631 | 1e-37 | 97.0 | 1 | 255 |
| AE008689\_Atu4332 | 1460748 | 1464227 | False | COG3523 | 0.0 | 99.0 | 1 | 1187 |
| AE008689\_Atu4333 | 1464224 | 1465729 | False | COG3455 | 1e-72 | 98.0 | 5 | 262 |
| AE008689\_Atu4333 | 1464224 | 1465729 | False | COG1360 | 1e-31 | 88.0 | 30 | 244 |
| AE008689\_Atu4334 | 1465726 | 1467066 | False | COG3522 | 2e-161 | 100.0 | 1 | 446 |
| AE008689\_Atu4335 | 1467056 | 1468255 | False | COG3456 | 5e-99 | 98.0 | 3 | 426 |
| AE008689\_Atu4336 | 1468266 | 1469270 | False | COG3520 | 4e-79 | 100.0 | 1 | 335 |
| AE008689\_Atu4337 | 1469280 | 1471061 | False | COG3519 | 6e-180 | 100.0 | 1 | 621 |
| AE008689\_Atu4338 | 1471054 | 1471563 | False | COG3518 | 7e-32 | 100.0 | 1 | 157 |
| AE008689\_Atu4339 | 1471556 | 1472140 | False | COG4455 | 6e-67 | 70.0 | 82 | 273 |
| AE008689\_Atu4340 | 1472377 | 1473813 | False | COG3517 | 3e-170 | 94.0 | 26 | 495 |
| AE008689\_Atu4341 | 1473826 | 1475307 | False | COG3517 | 0.0 | 99.0 | 1 | 494 |
| AE008689\_Atu4342 | 1475401 | 1475937 | False | COG3516 | 1e-55 | 100.0 | 1 | 169 |
| AE008689\_Atu4343 | 1475976 | 1477031 | False | COG3515 | 2e-52 | 100.0 | 1 | 346 |
| AE008689\_Atu4344 | 1477252 | 1479930 | True | COG0542 | 0.0 | 99.0 | 2 | 786 |
| AE008689\_Atu4345 | 1479988 | 1480464 | True | COG3157 | 4e-31 | 98.0 | 3 | 162 |
| AE008689\_Atu4346 | 1480511 | 1480960 | True | - | - | - | - | - |
| AE008689\_Atu4347 | 1480972 | 1481472 | True | - | - | - | - | - |
| AE008689\_Atu4348 | 1481487 | 1483937 | True | COG3501 | 3e-155 | 100.0 | 1 | 550 |
| AE008689\_Atu4349 | 1483940 | 1484896 | True | - | - | - | - | - |
| AE008689\_Atu4350 | 1484893 | 1485729 | True | - | - | - | - | - |
| AE008689\_Atu4351 | 1485805 | 1486431 | True | COG5620 | 8e-12 | 83.0 | 35 | 200 |
| AE008689\_Atu4352 | 1486444 | 1486749 | True | COG4104 | 5e-16 | 96.0 | 3 | 97 |
| AE008689\_Atu4353 | 1486888 | 1488726 | False | COG5001 | 0.0 | 94.0 | 37 | 661 |
