# Supplementary material for: Dissecting the bacterial type VI secretion system by a genome wide in silico analysis: what can be learned from available microbial genomic resources?
Source: BMC Genomics. 2009 Mar 12;10:104. doi: 10.1186/1471-2164-10-104 (PMC2660368; doi:10.1186/1471-2164-10-104)
Supplement: Additional file 7 — Detailed description of all identified T6SS gene clusters. Archive containing the detailed description of each identified T6SS locus as an HTML file. [file 1471-2164-10-104-S7.tgz › LociHTML/HTML/AE008923B.html]

Locus AE008923B on Xanthomonas axonopodis citri (strain 306) chromosome, complete sequence.

import namespace="svg" implementation="#AdobeSVG"?


# Locus AE008923B

# List of CDS in T6SS locus AE008923B

|  |  |  |  |  |  |  |  |  |
| --- | --- | --- | --- | --- | --- | --- | --- | --- |
| Name | from | to | direct | COG | e-value | COG cover | COG hit start | COG hit end |
| AE008923\_XAC4109 | 4813368 | 4814267 | True | COG0408 | 5e-131 | 99.0 | 4 | 303 |
| AE008923\_4815123..4817927 | 4815123 | 4817927 | False | COG0749 | 0.0 | 100.0 | 1 | 593 |
| AE008923\_4815123..4817927 | 4815123 | 4817927 | False | COG0258 | 3e-72 | 95.0 | 10 | 306 |
| AE008923\_XAC4111 | 4818004 | 4818414 | True | - | - | - | - | - |
| AE008923\_XAC4112 | 4818638 | 4819681 | False | COG3515 | 5e-22 | 97.0 | 7 | 342 |
| AE008923\_4819731..4826966 | 4819731 | 4826966 | False | - | - | - | - | - |
| AE008923\_4826870..4828552 | 4826870 | 4828552 | False | COG2831 | 3e-55 | 92.0 | 44 | 554 |
| AE008923\_XAC4115 | 4828543 | 4829004 | False | - | - | - | - | - |
| AE008923\_XAC4116 | 4829001 | 4832708 | False | COG0515 | 4e-37 | 57.0 | 2 | 222 |
| AE008923\_XAC4116 | 4829001 | 4832708 | False | COG1262 | 1e-11 | 61.0 | 86 | 277 |
| AE008923\_4832713..4833429 | 4832713 | 4833429 | False | COG0631 | 3e-38 | 94.0 | 6 | 253 |
| AE008923\_XAC4118 | 4833465 | 4833995 | False | COG3913 | 2e-16 | 71.0 | 8 | 170 |
| AE008923\_XAC4119 | 4833998 | 4837528 | False | COG3523 | 0.0 | 99.0 | 5 | 1183 |
| AE008923\_XAC4120 | 4837525 | 4838880 | False | COG3455 | 5e-50 | 99.0 | 2 | 262 |
| AE008923\_XAC4120 | 4837525 | 4838880 | False | COG1360 | 3e-21 | 57.0 | 103 | 243 |
| AE008923\_XAC4121 | 4838787 | 4840121 | False | COG3522 | 5e-130 | 99.0 | 2 | 446 |
| AE008923\_XAC4122 | 4840701 | 4842017 | False | COG3456 | 1e-40 | 99.0 | 3 | 428 |
| AE008923\_XAC4123 | 4842304 | 4842852 | False | - | - | - | - | - |
| AE008923\_XAC4124 | 4842849 | 4844885 | False | COG3501 | 1e-115 | 98.0 | 9 | 549 |
| AE008923\_XAC4125 | 4844967 | 4846298 | False | COG4976 | 5e-46 | 94.0 | 18 | 287 |
| AE008923\_XAC4125 | 4844967 | 4846298 | False | COG3063 | 6e-09 | 64.0 | 47 | 207 |
| AE008923\_XAC4126 | 4846534 | 4846929 | False | COG4104 | 5e-13 | 98.0 | 1 | 97 |
| AE008923\_4846892..4849189 | 4846892 | 4849189 | False | COG0515 | 1e-37 | 72.0 | 2 | 279 |
| AE008923\_4849390..4849929 | 4849390 | 4849929 | False | COG1595 | 5e-08 | 86.0 | 18 | 175 |
