# Supplementary material for: Dissecting the bacterial type VI secretion system by a genome wide in silico analysis: what can be learned from available microbial genomic resources?
Source: BMC Genomics. 2009 Mar 12;10:104. doi: 10.1186/1471-2164-10-104 (PMC2660368; doi:10.1186/1471-2164-10-104)
Supplement: Additional file 7 — Detailed description of all identified T6SS gene clusters. Archive containing the detailed description of each identified T6SS locus as an HTML file. [file 1471-2164-10-104-S7.tgz › LociHTML/HTML/AE008923C.html]

Locus AE008923C on Xanthomonas axonopodis citri (strain 306) chromosome, complete sequence.

import namespace="svg" implementation="#AdobeSVG"?


# Locus AE008923C

# List of CDS in T6SS locus AE008923C

|  |  |  |  |  |  |  |  |  |
| --- | --- | --- | --- | --- | --- | --- | --- | --- |
| Name | from | to | direct | COG | e-value | COG cover | COG hit start | COG hit end |
| AE008923\_XAC4136 | 4859777 | 4861771 | False | - | - | - | - | - |
| AE008923\_XAC4137 | 4862893 | 4864107 | True | - | - | - | - | - |
| AE008923\_XAC4138 | 4864112 | 4864522 | True | - | - | - | - | - |
| AE008923\_XAC4139 | 4864528 | 4865544 | False | - | - | - | - | - |
| AE008923\_4865531..4868311 | 4865531 | 4868311 | False | COG0542 | 9e-127 | 66.0 | 1 | 521 |
| AE008923\_4865531..4868311 | 4865531 | 4868311 | False | COG0542 | 3e-104 | 43.0 | 434 | 775 |
| AE008923\_XAC4141 | 4868367 | 4869407 | False | COG3520 | 8e-64 | 97.0 | 6 | 330 |
| AE008923\_XAC4142 | 4869371 | 4871254 | False | COG3519 | 3e-179 | 100.0 | 1 | 621 |
| AE008923\_XAC4143 | 4871259 | 4871762 | False | COG3518 | 3e-20 | 100.0 | 1 | 157 |
| AE008923\_XAC4144 | 4871768 | 4872607 | False | COG4455 | 5e-56 | 95.0 | 6 | 267 |
| AE008923\_XAC4145 | 4872761 | 4873264 | False | COG3157 | 4e-22 | 93.0 | 1 | 152 |
| AE008923\_XAC4146 | 4873345 | 4874838 | False | COG3517 | 0.0 | 99.0 | 4 | 494 |
| AE008923\_XAC4147 | 4874842 | 4875351 | False | COG3516 | 4e-54 | 100.0 | 1 | 169 |
| AE008923\_4875665..4876336 | 4875665 | 4876336 | True | COG4977 | 4e-10 | 38.0 | 200 | 326 |
| AE008923\_XAC4149 | 4876433 | 4876879 | False | COG0589 | 7e-16 | 96.0 | 4 | 151 |
| AE008923\_4877137..4877715 | 4877137 | 4877715 | False | COG0110 | 5e-29 | 93.0 | 6 | 182 |
| AE008923\_4878411..4880603 | 4878411 | 4880603 | False | COG0210 | 2e-174 | 99.0 | 1 | 654 |
