# Supplementary material for: Dissecting the bacterial type VI secretion system by a genome wide in silico analysis: what can be learned from available microbial genomic resources?
Source: BMC Genomics. 2009 Mar 12;10:104. doi: 10.1186/1471-2164-10-104 (PMC2660368; doi:10.1186/1471-2164-10-104)
Supplement: Additional file 7 — Detailed description of all identified T6SS gene clusters. Archive containing the detailed description of each identified T6SS locus as an HTML file. [file 1471-2164-10-104-S7.tgz › LociHTML/HTML/AE009952A.html]

Locus AE009952A on Yersinia pestis (biovar Mediaevalis, strain KIM5) chromosome, complete sequence.

import namespace="svg" implementation="#AdobeSVG"?


# Locus AE009952A

# List of CDS in T6SS locus AE009952A

|  |  |  |  |  |  |  |  |  |
| --- | --- | --- | --- | --- | --- | --- | --- | --- |
| Name | from | to | direct | COG | e-value | COG cover | COG hit start | COG hit end |
| AE009952\_y0030 | 44888 | 45250 | True | - | - | - | - | - |
| AE009952\_y0029 | 44998 | 45234 | True | - | - | - | - | - |
| AE009952\_y0031 | 45238 | 46347 | True | COG3839 | 9e-125 | 100.0 | 1 | 338 |
| AE009952\_y0032 | 46418 | 47689 | True | COG4580 | 4e-150 | 99.0 | 2 | 429 |
| AE009952\_y0033 | 47912 | 48841 | True | - | - | - | - | - |
| AE009952\_y0034 | 49078 | 49236 | False | - | - | - | - | - |
| AE009952\_y0035 | 49240 | 49590 | True | - | - | - | - | - |
| AE009952\_y0036 | 49742 | 50260 | False | COG3157 | 1e-50 | 98.0 | 1 | 160 |
| AE009952\_y0037 | 50781 | 51281 | True | COG3516 | 7e-50 | 98.0 | 1 | 167 |
| AE009952\_y0038 | 51349 | 52830 | True | COG3517 | 0.0 | 99.0 | 1 | 493 |
| AE009952\_y0039 | 52837 | 53277 | True | COG3518 | 9e-35 | 100.0 | 1 | 157 |
| AE009952\_y0040 | 53277 | 54503 | True | COG3519 | 6e-95 | 59.0 | 3 | 374 |
| AE009952\_y0041 | 54393 | 54650 | False | - | - | - | - | - |
| AE009952\_y0042 | 54689 | 55471 | False | COG1484 | 2e-64 | 100.0 | 1 | 254 |
| AE009952\_y0043 | 55468 | 56490 | False | COG4584 | 2e-58 | 100.0 | 1 | 278 |
| AE009952\_y0044 | 56560 | 56985 | True | COG1943 | 2e-28 | 87.0 | 15 | 133 |
| AE009952\_y0045 | 57193 | 57432 | False | COG3074 | 1e-14 | 100.0 | 1 | 79 |
| AE009952\_y0046 | 58060 | 58908 | True | COG0580 | 1e-57 | 99.0 | 2 | 241 |
| AE009952\_y0047 | 59085 | 60608 | True | COG0554 | 0.0 | 100.0 | 1 | 499 |
