# Supplementary material for: Dissecting the bacterial type VI secretion system by a genome wide in silico analysis: what can be learned from available microbial genomic resources?
Source: BMC Genomics. 2009 Mar 12;10:104. doi: 10.1186/1471-2164-10-104 (PMC2660368; doi:10.1186/1471-2164-10-104)
Supplement: Additional file 7 — Detailed description of all identified T6SS gene clusters. Archive containing the detailed description of each identified T6SS locus as an HTML file. [file 1471-2164-10-104-S7.tgz › LociHTML/HTML/AE009952B.html]

Locus AE009952B on Yersinia pestis (biovar Mediaevalis, strain KIM5) chromosome, complete sequence.

import namespace="svg" implementation="#AdobeSVG"?


# Locus AE009952B

# List of CDS in T6SS locus AE009952B

|  |  |  |  |  |  |  |  |  |
| --- | --- | --- | --- | --- | --- | --- | --- | --- |
| Name | from | to | direct | COG | e-value | COG cover | COG hit start | COG hit end |
| AE009952\_y0255 | 269719 | 274263 | False | COG3209 | 2e-63 | 99.0 | 1 | 795 |
| AE009952\_y0255 | 269719 | 274263 | False | COG4104 | 4e-10 | 73.0 | 25 | 96 |
| AE009952\_y0256 | 274302 | 274724 | False | COG5435 | 6e-45 | 97.0 | 3 | 145 |
| AE009952\_y0257 | 274727 | 276829 | False | COG3501 | 0.0 | 99.0 | 1 | 549 |
| AE009952\_y0258 | 276826 | 276924 | False | - | - | - | - | - |
| AE009952\_y0259 | 276946 | 277122 | False | - | - | - | - | - |
| AE009952\_y0260 | 277204 | 277692 | True | - | - | - | - | - |
| AE009952\_y0261 | 277774 | 277998 | True | - | - | - | - | - |
| AE009952\_y0262 | 277977 | 278174 | False | - | - | - | - | - |
| AE009952\_y0263 | 278074 | 278265 | True | - | - | - | - | - |
| AE009952\_y0264 | 278315 | 278800 | False | - | - | - | - | - |
| AE009952\_y0265 | 278802 | 280172 | False | COG3209 | 8e-32 | 59.0 | 326 | 795 |
| AE009952\_y0266 | 280200 | 283091 | False | COG3209 | 1e-61 | 99.0 | 2 | 794 |
| AE009952\_y0267 | 283057 | 283515 | False | COG5435 | 5e-48 | 100.0 | 1 | 147 |
| AE009952\_y0268 | 283521 | 285923 | False | COG3501 | 0.0 | 99.0 | 1 | 547 |
| AE009952\_y0270 | 287229 | 287591 | True | - | - | - | - | - |
| AE009952\_y0272 | 290946 | 292352 | False | COG3515 | 1e-36 | 82.0 | 1 | 285 |
| AE009952\_y0273 | 292340 | 293035 | False | - | - | - | - | - |
| AE009952\_y0274 | 293023 | 293820 | False | - | - | - | - | - |
| AE009952\_y0275 | 293817 | 296420 | False | COG0542 | 0.0 | 99.0 | 1 | 784 |
| AE009952\_y0276 | 296431 | 297198 | False | COG3455 | 2e-86 | 98.0 | 4 | 260 |
| AE009952\_y0277 | 297198 | 298544 | False | COG3522 | 4e-167 | 100.0 | 1 | 446 |
| AE009952\_y0278 | 298547 | 299092 | False | COG3521 | 2e-39 | 100.0 | 1 | 159 |
| AE009952\_y0279 | 299092 | 300408 | False | COG3456 | 9e-124 | 100.0 | 1 | 430 |
| AE009952\_y0280 | 300534 | 301631 | False | COG3520 | 1e-105 | 99.0 | 1 | 332 |
| AE009952\_y0281 | 301586 | 302458 | False | COG3519 | 3e-60 | 39.0 | 379 | 621 |
| AE009952\_y0282 | 302218 | 302895 | True | COG2963 | 5e-13 | 95.0 | 6 | 116 |
| AE009952\_y0283 | 302949 | 303569 | True | COG2801 | 6e-09 | 64.0 | 16 | 164 |
| AE009952\_y0284 | 303596 | 304618 | True | COG4584 | 2e-58 | 100.0 | 1 | 278 |
| AE009952\_y0285 | 304615 | 305397 | True | COG1484 | 2e-64 | 100.0 | 1 | 254 |
| AE009952\_y0286 | 305910 | 308024 | False | COG2274 | 1e-170 | 96.0 | 1 | 682 |
