# Supplementary material for: Dissecting the bacterial type VI secretion system by a genome wide in silico analysis: what can be learned from available microbial genomic resources?
Source: BMC Genomics. 2009 Mar 12;10:104. doi: 10.1186/1471-2164-10-104 (PMC2660368; doi:10.1186/1471-2164-10-104)
Supplement: Additional file 7 — Detailed description of all identified T6SS gene clusters. Archive containing the detailed description of each identified T6SS locus as an HTML file. [file 1471-2164-10-104-S7.tgz › LociHTML/HTML/AE009952C.html]

Locus AE009952C on Yersinia pestis (biovar Mediaevalis, strain KIM5) chromosome, complete sequence.

import namespace="svg" implementation="#AdobeSVG"?


# Locus AE009952C

# List of CDS in T6SS locus AE009952C

|  |  |  |  |  |  |  |  |  |
| --- | --- | --- | --- | --- | --- | --- | --- | --- |
| Name | from | to | direct | COG | e-value | COG cover | COG hit start | COG hit end |
| AE009952\_y1529 | 1692646 | 1693128 | False | COG5435 | 7e-43 | 100.0 | 1 | 147 |
| AE009952\_y1530 | 1693125 | 1694807 | False | COG2885 | 1e-27 | 55.0 | 86 | 190 |
| AE009952\_y1531 | 1694808 | 1695560 | False | - | - | - | - | - |
| AE009952\_y1532 | 1695506 | 1696843 | False | - | - | - | - | - |
| AE009952\_y1533 | 1696883 | 1697914 | False | - | - | - | - | - |
| AE009952\_y1534 | 1697921 | 1698991 | False | COG3515 | 1e-40 | 90.0 | 29 | 341 |
| AE009952\_y1536 | 1699201 | 1700262 | False | COG3520 | 2e-97 | 100.0 | 1 | 335 |
| AE009952\_y1535 | 1699474 | 1699692 | True | - | - | - | - | - |
| AE009952\_y1537 | 1700226 | 1702106 | False | COG3519 | 0.0 | 100.0 | 1 | 621 |
| AE009952\_y1538 | 1702814 | 1705564 | True | COG0542 | 0.0 | 98.0 | 1 | 777 |
| AE009952\_y1539 | 1705756 | 1706310 | True | COG3539 | 2e-14 | 100.0 | 1 | 184 |
| AE009952\_y1540 | 1706307 | 1706588 | False | - | - | - | - | - |
| AE009952\_y1541 | 1706449 | 1707228 | True | COG3121 | 6e-59 | 99.0 | 1 | 234 |
| AE009952\_y1542 | 1708928 | 1710136 | False | COG3328 | 2e-112 | 98.0 | 1 | 375 |
| AE009952\_y1544 | 1711336 | 1711944 | True | COG3539 | 4e-16 | 91.0 | 16 | 184 |
| AE009952\_y1545 | 1712028 | 1712573 | True | COG3516 | 3e-59 | 99.0 | 2 | 169 |
| AE009952\_y1546 | 1712576 | 1714099 | True | COG3517 | 0.0 | 100.0 | 1 | 495 |
| AE009952\_y1547 | 1714341 | 1714910 | True | COG3157 | 4e-42 | 100.0 | 1 | 162 |
| AE009952\_y1548 | 1715124 | 1715723 | True | COG3521 | 2e-39 | 100.0 | 1 | 159 |
| AE009952\_y1549 | 1715727 | 1717076 | True | COG3522 | 3e-158 | 99.0 | 2 | 446 |
| AE009952\_y1550 | 1717755 | 1718777 | True | COG4584 | 2e-58 | 100.0 | 1 | 278 |
| AE009952\_y1551 | 1718774 | 1719556 | True | COG1484 | 2e-64 | 100.0 | 1 | 254 |
| AE009952\_y1553 | 1720666 | 1724244 | True | COG3523 | 0.0 | 97.0 | 30 | 1185 |
| AE009952\_y1555 | 1724538 | 1727123 | True | COG3501 | 7e-150 | 97.0 | 6 | 539 |
| AE009952\_y1554 | 1726535 | 1727032 | False | COG2171 | 2e-07 | 51.0 | 104 | 243 |
| AE009952\_y1556 | 1726636 | 1727175 | False | - | - | - | - | - |
| AE009952\_y1557 | 1727222 | 1727482 | True | - | - | - | - | - |
| AE009952\_y1559 | 1727482 | 1728330 | True | - | - | - | - | - |
| AE009952\_y1558 | 1727575 | 1727763 | False | - | - | - | - | - |
| AE009952\_y1560 | 1728350 | 1729156 | True | COG4455 | 9e-108 | 100.0 | 1 | 273 |
| AE009952\_y1561 | 1729285 | 1729764 | True | COG3518 | 4e-35 | 99.0 | 1 | 156 |
| AE009952\_y1562 | 1729771 | 1730136 | True | - | - | - | - | - |
| AE009952\_y1563 | 1730211 | 1730552 | False | COG2824 | 2e-46 | 100.0 | 1 | 112 |
| AE009952\_y1564 | 1730928 | 1733027 | False | COG4907 | 4e-08 | 30.0 | 412 | 594 |
| AE009952\_y1565 | 1732972 | 1733523 | False | COG1704 | 3e-55 | 99.0 | 2 | 185 |
| AE009952\_y1566 | 1734091 | 1734597 | True | - | - | - | - | - |
