# Supplementary material for: Dissecting the bacterial type VI secretion system by a genome wide in silico analysis: what can be learned from available microbial genomic resources?
Source: BMC Genomics. 2009 Mar 12;10:104. doi: 10.1186/1471-2164-10-104 (PMC2660368; doi:10.1186/1471-2164-10-104)
Supplement: Additional file 7 — Detailed description of all identified T6SS gene clusters. Archive containing the detailed description of each identified T6SS locus as an HTML file. [file 1471-2164-10-104-S7.tgz › LociHTML/HTML/AE009952E.html]

Locus AE009952E on Yersinia pestis (biovar Mediaevalis, strain KIM5) chromosome, complete sequence.

import namespace="svg" implementation="#AdobeSVG"?


# Locus AE009952E

# List of CDS in T6SS locus AE009952E

|  |  |  |  |  |  |  |  |  |
| --- | --- | --- | --- | --- | --- | --- | --- | --- |
| Name | from | to | direct | COG | e-value | COG cover | COG hit start | COG hit end |
| AE009952\_y2675 | 2952460 | 2953668 | True | COG3328 | 2e-112 | 98.0 | 1 | 375 |
| AE009952\_y2676 | 2953712 | 2954365 | False | - | - | - | - | - |
| AE009952\_y2677 | 2954593 | 2956356 | False | COG0488 | 8e-172 | 99.0 | 1 | 528 |
| AE009952\_y2678 | 2956871 | 2957494 | True | - | - | - | - | - |
| AE009952\_y2679 | 2957706 | 2959073 | False | COG3515 | 9e-41 | 96.0 | 13 | 346 |
| AE009952\_y2680 | 2959098 | 2959367 | False | COG3518 | 2e-11 | 52.0 | 75 | 157 |
| AE009952\_y2681 | 2959360 | 2959551 | False | COG3518 | 1e-07 | 39.0 | 3 | 64 |
| AE009952\_y2682 | 2959551 | 2960132 | False | COG3521 | 3e-35 | 98.0 | 1 | 157 |
| AE009952\_y2683 | 2960107 | 2961192 | False | COG3520 | 3e-85 | 97.0 | 1 | 328 |
| AE009952\_y2684 | 2961156 | 2962919 | False | COG3519 | 0.0 | 100.0 | 1 | 621 |
| AE009952\_y2685 | 2963140 | 2963610 | False | - | - | - | - | - |
| AE009952\_y2686 | 2963610 | 2964668 | False | - | - | - | - | - |
| AE009952\_y2687 | 2964686 | 2966287 | False | COG3515 | 8e-42 | 100.0 | 1 | 346 |
| AE009952\_y2688 | 2966331 | 2969753 | False | COG3523 | 0.0 | 100.0 | 1 | 1188 |
| AE009952\_y2689 | 2969750 | 2970982 | False | - | - | - | - | - |
| AE009952\_y2690 | 2972094 | 2972414 | True | - | - | - | - | - |
| AE009952\_y2691 | 2972545 | 2972682 | True | - | - | - | - | - |
| AE009952\_y2692 | 2973147 | 2973617 | False | - | - | - | - | - |
| AE009952\_y2694 | 2973790 | 2975952 | False | - | - | - | - | - |
| AE009952\_y2693 | 2973921 | 2974181 | False | - | - | - | - | - |
| AE009952\_y2695 | 2975989 | 2976249 | False | COG4253 | 2e-25 | 30.0 | 144 | 229 |
| AE009952\_y2696 | 2976403 | 2977176 | False | - | - | - | - | - |
| AE009952\_y2697 | 2977173 | 2979473 | False | - | - | - | - | - |
| AE009952\_y2698 | 2979489 | 2981837 | False | COG3501 | 1e-105 | 99.0 | 1 | 549 |
| AE009952\_y2698 | 2979489 | 2981837 | False | COG4253 | 3e-67 | 82.0 | 2 | 229 |
| AE009952\_y2699 | 2981840 | 2984479 | False | COG0542 | 0.0 | 99.0 | 1 | 784 |
| AE009952\_y2700 | 2984867 | 2985358 | False | COG3157 | 2e-40 | 98.0 | 1 | 160 |
| AE009952\_y2701 | 2984900 | 2985385 | True | - | - | - | - | - |
| AE009952\_y2702 | 2985362 | 2987098 | False | COG2885 | 8e-27 | 94.0 | 12 | 190 |
| AE009952\_y2703 | 2987098 | 2987784 | False | COG3455 | 2e-48 | 91.0 | 21 | 260 |
| AE009952\_y2704 | 2987781 | 2989133 | False | COG3522 | 6e-133 | 99.0 | 2 | 446 |
| AE009952\_y2705 | 2989145 | 2990689 | False | COG3517 | 0.0 | 100.0 | 1 | 495 |
| AE009952\_y2706 | 2990732 | 2991235 | False | COG3516 | 3e-49 | 100.0 | 1 | 169 |
| AE009952\_y2707 | 2992221 | 2993069 | False | COG0331 | 2e-74 | 93.0 | 2 | 290 |
| AE009952\_y2708 | 2993454 | 2993942 | False | - | - | - | - | - |
| AE009952\_y2709 | 2993981 | 2994769 | False | COG1028 | 4e-25 | 99.0 | 2 | 250 |
| AE009952\_y2710 | 2994772 | 2995548 | False | COG1024 | 5e-37 | 93.0 | 1 | 240 |
| AE009952\_y2711 | 2995526 | 2996296 | False | COG1024 | 7e-29 | 87.0 | 29 | 252 |
