# Supplementary material for: Dissecting the bacterial type VI secretion system by a genome wide in silico analysis: what can be learned from available microbial genomic resources?
Source: BMC Genomics. 2009 Mar 12;10:104. doi: 10.1186/1471-2164-10-104 (PMC2660368; doi:10.1186/1471-2164-10-104)
Supplement: Additional file 7 — Detailed description of all identified T6SS gene clusters. Archive containing the detailed description of each identified T6SS locus as an HTML file. [file 1471-2164-10-104-S7.tgz › LociHTML/HTML/AE009952F.html]

Locus AE009952F on Yersinia pestis (biovar Mediaevalis, strain KIM5) chromosome, complete sequence.

import namespace="svg" implementation="#AdobeSVG"?


# Locus AE009952F

# List of CDS in T6SS locus AE009952F

|  |  |  |  |  |  |  |  |  |
| --- | --- | --- | --- | --- | --- | --- | --- | --- |
| Name | from | to | direct | COG | e-value | COG cover | COG hit start | COG hit end |
| AE009952\_y3353 | 3695454 | 3697751 | False | COG3179 | 6e-09 | 98.0 | 4 | 206 |
| AE009952\_y3354 | 3697767 | 3698402 | False | COG4253 | 3e-62 | 81.0 | 4 | 229 |
| AE009952\_y3356 | 3698934 | 3699269 | False | COG1662 | 8e-26 | 95.0 | 6 | 121 |
| AE009952\_y3355 | 3699010 | 3699267 | False | COG1662 | 2e-09 | 39.0 | 41 | 88 |
| AE009952\_y3357 | 3699323 | 3699445 | False | COG3677 | 1e-06 | 31.0 | 78 | 117 |
| AE009952\_y3358 | 3699479 | 3699634 | True | - | - | - | - | - |
| AE009952\_y3359 | 3700005 | 3700394 | False | - | - | - | - | - |
| AE009952\_y3360 | 3700505 | 3702727 | False | - | - | - | - | - |
| AE009952\_y3361 | 3702742 | 3705090 | False | COG3501 | 9e-110 | 99.0 | 1 | 549 |
| AE009952\_y3361 | 3702742 | 3705090 | False | COG4253 | 6e-66 | 82.0 | 2 | 229 |
| AE009952\_y3362 | 3705087 | 3707735 | False | COG0542 | 0.0 | 100.0 | 1 | 786 |
| AE009952\_y3363 | 3708153 | 3708644 | False | COG3157 | 2e-40 | 98.0 | 1 | 160 |
| AE009952\_y3364 | 3708186 | 3708671 | True | - | - | - | - | - |
| AE009952\_y3365 | 3708648 | 3710384 | False | COG2885 | 8e-27 | 94.0 | 12 | 190 |
| AE009952\_y3366 | 3710384 | 3711070 | False | COG3455 | 2e-48 | 91.0 | 21 | 260 |
| AE009952\_y3367 | 3711067 | 3712419 | False | COG3522 | 2e-132 | 99.0 | 2 | 446 |
| AE009952\_y3368 | 3712431 | 3713975 | False | COG3517 | 0.0 | 100.0 | 1 | 495 |
| AE009952\_y3369 | 3714018 | 3714521 | False | COG3516 | 3e-49 | 100.0 | 1 | 169 |
| AE009952\_y3370 | 3715918 | 3716289 | False | - | - | - | - | - |
| AE009952\_y3371 | 3716794 | 3717189 | False | - | - | - | - | - |
| AE009952\_y3372 | 3717402 | 3717497 | False | - | - | - | - | - |
| AE009952\_y3373 | 3717640 | 3718290 | False | - | - | - | - | - |
| AE009952\_y3374 | 3719465 | 3720115 | True | COG3916 | 3e-64 | 100.0 | 1 | 209 |
