# Supplementary material for: Dissecting the bacterial type VI secretion system by a genome wide in silico analysis: what can be learned from available microbial genomic resources?
Source: BMC Genomics. 2009 Mar 12;10:104. doi: 10.1186/1471-2164-10-104 (PMC2660368; doi:10.1186/1471-2164-10-104)
Supplement: Additional file 7 — Detailed description of all identified T6SS gene clusters. Archive containing the detailed description of each identified T6SS locus as an HTML file. [file 1471-2164-10-104-S7.tgz › LociHTML/HTML/AE009952G.html]

Locus AE009952G on Yersinia pestis (biovar Mediaevalis, strain KIM5) chromosome, complete sequence.

import namespace="svg" implementation="#AdobeSVG"?


# Locus AE009952G

# List of CDS in T6SS locus AE009952G

|  |  |  |  |  |  |  |  |  |
| --- | --- | --- | --- | --- | --- | --- | --- | --- |
| Name | from | to | direct | COG | e-value | COG cover | COG hit start | COG hit end |
| AE009952\_y3655 | 4055920 | 4058289 | True | COG0417 | 0.0 | 98.0 | 5 | 788 |
| AE009952\_y3656 | 4058750 | 4061656 | True | COG0553 | 2e-72 | 98.0 | 8 | 861 |
| AE009952\_y3657 | 4061912 | 4062271 | False | - | - | - | - | - |
| AE009952\_y3658 | 4062287 | 4065781 | False | COG3523 | 0.0 | 98.0 | 12 | 1187 |
| AE009952\_y3659 | 4065790 | 4067427 | False | COG3455 | 2e-72 | 100.0 | 1 | 262 |
| AE009952\_y3659 | 4065790 | 4067427 | False | COG1360 | 2e-27 | 56.0 | 108 | 244 |
| AE009952\_y3660 | 4067397 | 4068770 | False | COG3522 | 2e-141 | 100.0 | 1 | 446 |
| AE009952\_y3661 | 4068872 | 4069246 | False | COG3521 | 5e-27 | 78.0 | 34 | 158 |
| AE009952\_y3662 | 4069356 | 4069721 | False | - | - | - | - | - |
| AE009952\_y3663 | 4069727 | 4070344 | False | - | - | - | - | - |
| AE009952\_y3664 | 4070337 | 4071440 | False | COG1357 | 1e-18 | 99.0 | 3 | 238 |
| AE009952\_y3665 | 4071466 | 4073685 | False | COG1357 | 4e-15 | 83.0 | 18 | 215 |
| AE009952\_y3665 | 4071466 | 4073685 | False | COG5351 | 1e-07 | 50.0 | 68 | 253 |
| AE009952\_y3668 | 4073698 | 4076046 | False | COG3501 | 5e-154 | 95.0 | 10 | 533 |
| AE009952\_y3666 | 4073783 | 4074103 | True | - | - | - | - | - |
| AE009952\_y3667 | 4074107 | 4074439 | True | - | - | - | - | - |
| AE009952\_y3669 | 4076150 | 4078753 | False | COG0542 | 0.0 | 99.0 | 1 | 783 |
| AE009952\_y3670 | 4078756 | 4079778 | False | COG3520 | 3e-84 | 99.0 | 3 | 334 |
| AE009952\_y3671 | 4079732 | 4081576 | False | COG3519 | 0.0 | 99.0 | 1 | 617 |
| AE009952\_y3672 | 4081609 | 4082052 | False | COG3518 | 8e-31 | 95.0 | 4 | 153 |
| AE009952\_y3673 | 4082126 | 4082644 | False | COG3157 | 7e-36 | 100.0 | 1 | 162 |
| AE009952\_y3674 | 4082807 | 4084318 | False | COG3517 | 0.0 | 100.0 | 1 | 495 |
| AE009952\_y3675 | 4084318 | 4084878 | False | COG3516 | 1e-57 | 99.0 | 2 | 169 |
| AE009952\_y3676 | 4084889 | 4085902 | False | COG3515 | 4e-55 | 100.0 | 1 | 346 |
| AE009952\_y3677 | 4086232 | 4086975 | True | COG5419 | 2e-48 | 100.0 | 1 | 160 |
| AE009952\_y3678 | 4088198 | 4088818 | True | COG0564 | 1e-59 | 72.0 | 80 | 289 |
| AE009952\_y3679 | 4089116 | 4089949 | False | COG1076 | 6e-35 | 100.0 | 1 | 174 |
| AE009952\_y3680 | 4090134 | 4092476 | True | COG1452 | 0.0 | 100.0 | 1 | 784 |
