# Supplementary material for: Dissecting the bacterial type VI secretion system by a genome wide in silico analysis: what can be learned from available microbial genomic resources?
Source: BMC Genomics. 2009 Mar 12;10:104. doi: 10.1186/1471-2164-10-104 (PMC2660368; doi:10.1186/1471-2164-10-104)
Supplement: Additional file 7 — Detailed description of all identified T6SS gene clusters. Archive containing the detailed description of each identified T6SS locus as an HTML file. [file 1471-2164-10-104-S7.tgz › LociHTML/HTML/AE013598B.html]

Locus AE013598B on Xanthomonas oryzae oryzae (strain KXO85 / KACC10331) chromosome, complete sequence.

import namespace="svg" implementation="#AdobeSVG"?


# Locus AE013598B

# List of CDS in T6SS locus AE013598B

|  |  |  |  |  |  |  |  |  |
| --- | --- | --- | --- | --- | --- | --- | --- | --- |
| Name | from | to | direct | COG | e-value | COG cover | COG hit start | COG hit end |
| AE013598\_XOO3030 | 3253076 | 3253855 | True | COG0745 | 2e-47 | 98.0 | 2 | 227 |
| AE013598\_XOO3031 | 3253852 | 3254844 | True | COG1858 | 1e-48 | 94.0 | 20 | 362 |
| AE013598\_XOO3032 | 3254841 | 3257300 | True | COG0642 | 5e-28 | 88.0 | 38 | 336 |
| AE013598\_XOO3033 | 3257414 | 3258394 | True | COG0583 | 2e-16 | 99.0 | 2 | 297 |
| AE013598\_XOO3034 | 3258391 | 3259431 | True | COG3515 | 9e-15 | 96.0 | 7 | 341 |
| AE013598\_XOO4863 | 3259604 | 3259930 | False | - | - | - | - | - |
| AE013598\_XOO3035 | 3259927 | 3262830 | False | COG0515 | 4e-35 | 72.0 | 2 | 278 |
| AE013598\_XOO3035 | 3259927 | 3262830 | False | COG1262 | 5e-09 | 61.0 | 86 | 277 |
| AE013598\_XOO3036 | 3262827 | 3263549 | False | COG0631 | 2e-35 | 94.0 | 4 | 251 |
| AE013598\_XOO3037 | 3263546 | 3264199 | False | COG3913 | 1e-13 | 46.0 | 1 | 106 |
| AE013598\_XOO3038 | 3264190 | 3267648 | False | COG3523 | 0.0 | 99.0 | 3 | 1184 |
| AE013598\_XOO3039 | 3267652 | 3268968 | False | COG3455 | 2e-48 | 96.0 | 7 | 259 |
| AE013598\_XOO3039 | 3267652 | 3268968 | False | COG1360 | 4e-20 | 56.0 | 108 | 244 |
| AE013598\_XOO3040 | 3268970 | 3271741 | False | COG3456 | 1e-22 | 75.0 | 1 | 325 |
| AE013598\_XOO3040 | 3268970 | 3271741 | False | COG3522 | 8e-129 | 100.0 | 1 | 446 |
| AE013598\_XOO3042 | 3271738 | 3272289 | False | - | - | - | - | - |
| AE013598\_XOO3043 | 3272286 | 3274334 | False | COG3501 | 9e-127 | 96.0 | 10 | 539 |
| AE013598\_XOO3044 | 3275021 | 3275272 | False | - | - | - | - | - |
| AE013598\_XOO3045 | 3276579 | 3279377 | False | COG0542 | 0.0 | 99.0 | 1 | 783 |
| AE013598\_XOO3046 | 3279317 | 3280357 | False | COG3520 | 2e-72 | 99.0 | 1 | 332 |
| AE013598\_XOO3047 | 3280291 | 3282168 | False | COG3519 | 4e-170 | 100.0 | 1 | 621 |
| AE013598\_XOO3048 | 3282172 | 3282675 | False | COG3518 | 1e-21 | 98.0 | 1 | 154 |
| AE013598\_XOO3049 | 3282663 | 3283496 | False | COG4455 | 7e-49 | 94.0 | 9 | 265 |
| AE013598\_XOO3050 | 3283532 | 3284035 | False | COG3157 | 2e-22 | 93.0 | 1 | 151 |
| AE013598\_XOO3051 | 3284135 | 3285706 | False | COG3517 | 0.0 | 100.0 | 1 | 495 |
| AE013598\_XOO3052 | 3285642 | 3286337 | False | COG3516 | 5e-55 | 97.0 | 2 | 166 |
| AE013598\_XOO3053 | 3286670 | 3287311 | False | - | - | - | - | - |
| AE013598\_XOO3905 | 3287507 | 3288475 | False | COG3039 | 3e-22 | 99.0 | 1 | 229 |
| AE013598\_XOO3055 | 3288537 | 3289121 | False | - | - | - | - | - |
| AE013598\_XOO3056 | 3288624 | 3289262 | True | - | - | - | - | - |
| AE013598\_XOO3057 | 3289124 | 3289912 | False | - | - | - | - | - |
| AE013598\_XOO3058 | 3289259 | 3290107 | True | COG3464 | 2e-12 | 42.0 | 209 | 378 |
| AE013598\_XOO4864 | 3289981 | 3290874 | False | - | - | - | - | - |
| AE013598\_XOO3060 | 3290771 | 3291826 | False | - | - | - | - | - |
