# Supplementary material for: Dissecting the bacterial type VI secretion system by a genome wide in silico analysis: what can be learned from available microbial genomic resources?
Source: BMC Genomics. 2009 Mar 12;10:104. doi: 10.1186/1471-2164-10-104 (PMC2660368; doi:10.1186/1471-2164-10-104)
Supplement: Additional file 7 — Detailed description of all identified T6SS gene clusters. Archive containing the detailed description of each identified T6SS locus as an HTML file. [file 1471-2164-10-104-S7.tgz › LociHTML/HTML/AE013598C.html]

Locus AE013598C on Xanthomonas oryzae oryzae (strain KXO85 / KACC10331) chromosome, complete sequence.

import namespace="svg" implementation="#AdobeSVG"?


# Locus AE013598C

# List of CDS in T6SS locus AE013598C

|  |  |  |  |  |  |  |  |  |
| --- | --- | --- | --- | --- | --- | --- | --- | --- |
| Name | from | to | direct | COG | e-value | COG cover | COG hit start | COG hit end |
| AE013598\_XOO3462 | 3702677 | 3703903 | True | COG4823 | 9e-42 | 96.0 | 2 | 291 |
| AE013598\_XOO3463 | 3703950 | 3705494 | True | COG0286 | 2e-65 | 99.0 | 2 | 489 |
| AE013598\_XOO3464 | 3705472 | 3706866 | True | COG0732 | 5e-23 | 84.0 | 60 | 391 |
| AE013598\_XOO3465 | 3706900 | 3707208 | False | - | - | - | - | - |
| AE013598\_XOO4880 | 3707215 | 3707478 | False | - | - | - | - | - |
| AE013598\_XOO3466 | 3708207 | 3708962 | True | COG3501 | 5e-33 | 28.0 | 2 | 160 |
| AE013598\_XOO4881 | 3708821 | 3708991 | True | - | - | - | - | - |
| AE013598\_XOO3467 | 3709256 | 3710278 | False | - | - | - | - | - |
| AE013598\_XOO3468 | 3710288 | 3713155 | False | - | - | - | - | - |
| AE013598\_XOO3469 | 3713174 | 3714079 | False | - | - | - | - | - |
| AE013598\_XOO3470 | 3714021 | 3715082 | False | COG4253 | 2e-18 | 79.0 | 1 | 221 |
| AE013598\_XOO3471 | 3714076 | 3716028 | False | COG3501 | 3e-31 | 39.0 | 265 | 483 |
| AE013598\_XOO3472 | 3716033 | 3718045 | False | - | - | - | - | - |
| AE013598\_XOO4882 | 3719245 | 3720123 | False | - | - | - | - | - |
| AE013598\_XOO3473 | 3720130 | 3721437 | False | COG4253 | 7e-20 | 86.0 | 1 | 240 |
| AE013598\_XOO3474 | 3721305 | 3722894 | False | COG3501 | 1e-94 | 82.0 | 1 | 454 |
| AE013598\_XOO4883 | 3722987 | 3723340 | False | - | - | - | - | - |
| AE013598\_XOO3475 | 3723371 | 3726100 | False | COG0542 | 0.0 | 98.0 | 1 | 778 |
| AE013598\_XOO3476 | 3726186 | 3727277 | False | COG3520 | 3e-63 | 98.0 | 1 | 331 |
| AE013598\_XOO3477 | 3727241 | 3729076 | False | COG3519 | 1e-151 | 99.0 | 2 | 620 |
| AE013598\_XOO3478 | 3729079 | 3729567 | False | COG3518 | 1e-28 | 96.0 | 4 | 155 |
| AE013598\_XOO3479 | 3729715 | 3730212 | False | COG3157 | 2e-23 | 89.0 | 6 | 150 |
| AE013598\_XOO3480 | 3730354 | 3731850 | False | COG3517 | 0.0 | 99.0 | 1 | 491 |
| AE013598\_XOO3481 | 3731854 | 3732354 | False | COG3516 | 9e-47 | 94.0 | 2 | 160 |
| AE013598\_XOO3482 | 3732401 | 3733015 | False | - | - | - | - | - |
| AE013598\_XOO3483 | 3733231 | 3733884 | True | COG3521 | 2e-18 | 86.0 | 1 | 137 |
| AE013598\_XOO3484 | 3733943 | 3735370 | True | COG3522 | 3e-104 | 99.0 | 2 | 446 |
| AE013598\_XOO3485 | 3735367 | 3736158 | True | COG3455 | 2e-41 | 93.0 | 13 | 258 |
| AE013598\_XOO3486 | 3736169 | 3738706 | True | COG4253 | 7e-31 | 79.0 | 1 | 221 |
| AE013598\_XOO3486 | 3736169 | 3738706 | True | COG3501 | 2e-93 | 96.0 | 2 | 530 |
| AE013598\_XOO3487 | 3738731 | 3741625 | True | COG3179 | 2e-12 | 76.0 | 44 | 201 |
| AE013598\_XOO3488 | 3742460 | 3743893 | True | COG4253 | 2e-33 | 82.0 | 1 | 230 |
| AE013598\_XOO3488 | 3742460 | 3743893 | True | COG3501 | 3e-27 | 32.0 | 354 | 530 |
| AE013598\_XOO3489 | 3743897 | 3746152 | True | - | - | - | - | - |
| AE013598\_XOO3490 | 3746714 | 3748975 | True | COG4253 | 1e-33 | 94.0 | 1 | 263 |
| AE013598\_XOO3490 | 3746714 | 3748975 | True | COG3501 | 5e-48 | 63.0 | 184 | 530 |
| AE013598\_XOO4884 | 3748975 | 3750519 | True | - | - | - | - | - |
| AE013598\_XOO3491 | 3751166 | 3752389 | True | - | - | - | - | - |
| AE013598\_XOO3492 | 3752416 | 3752955 | True | - | - | - | - | - |
| AE013598\_XOO4885 | 3753076 | 3753369 | True | - | - | - | - | - |
| AE013598\_XOO4886 | 3754099 | 3754446 | True | - | - | - | - | - |
| AE013598\_XOO3493 | 3754541 | 3755431 | True | COG3522 | 2e-69 | 65.0 | 154 | 446 |
| AE013598\_XOO3494 | 3755428 | 3756219 | True | COG3455 | 7e-42 | 93.0 | 13 | 258 |
| AE013598\_XOO3495 | 3756230 | 3757396 | True | COG3501 | 2e-62 | 68.0 | 2 | 375 |
| AE013598\_XOO3496 | 3757451 | 3758563 | True | COG3501 | 4e-18 | 24.0 | 396 | 530 |
| AE013598\_XOO3497 | 3757737 | 3758570 | True | COG4253 | 1e-19 | 47.0 | 27 | 157 |
| AE013598\_XOO3498 | 3758605 | 3758994 | False | COG4253 | 1e-13 | 40.0 | 127 | 239 |
| AE013598\_XOO3499 | 3758975 | 3761257 | False | - | - | - | - | - |
| AE013598\_XOO3500 | 3761206 | 3761802 | False | - | - | - | - | - |
| AE013598\_XOO3501 | 3761795 | 3762670 | False | - | - | - | - | - |
| AE013598\_XOO3502 | 3762616 | 3765474 | False | COG4253 | 9e-14 | 54.0 | 90 | 240 |
| AE013598\_XOO3503 | 3763083 | 3764609 | False | COG3501 | 2e-39 | 51.0 | 268 | 550 |
| AE013598\_XOO3504 | 3764610 | 3765293 | False | COG4682 | 1e-06 | 49.0 | 44 | 106 |
| AE013598\_XOO3504 | 3764610 | 3765293 | False | COG3501 | 3e-19 | 22.0 | 124 | 246 |
| AE013598\_XOO3505 | 3765389 | 3766435 | True | COG1262 | 2e-25 | 67.0 | 43 | 254 |
| AE013598\_XOO3506 | 3766489 | 3769554 | False | - | - | - | - | - |
| AE013598\_XOO3507 | 3769541 | 3770086 | False | COG4253 | 3e-16 | 52.0 | 122 | 266 |
| AE013598\_XOO3508 | 3770168 | 3772000 | False | - | - | - | - | - |
