# Supplementary material for: Dissecting the bacterial type VI secretion system by a genome wide in silico analysis: what can be learned from available microbial genomic resources?
Source: BMC Genomics. 2009 Mar 12;10:104. doi: 10.1186/1471-2164-10-104 (PMC2660368; doi:10.1186/1471-2164-10-104)
Supplement: Additional file 7 — Detailed description of all identified T6SS gene clusters. Archive containing the detailed description of each identified T6SS locus as an HTML file. [file 1471-2164-10-104-S7.tgz › LociHTML/HTML/AE014073A.html]

Locus AE014073A on Shigella flexneri (serovar 2a, strain 2457T / ATCC 700930) chromosome, complete sequence.

import namespace="svg" implementation="#AdobeSVG"?


# Locus AE014073A

# List of CDS in T6SS locus AE014073A

|  |  |  |  |  |  |  |  |  |
| --- | --- | --- | --- | --- | --- | --- | --- | --- |
| Name | from | to | direct | COG | e-value | COG cover | COG hit start | COG hit end |
| AE014073\_S0204 | 223972 | 224592 | True | COG2226 | 7e-18 | 44.0 | 59 | 164 |
| AE014073\_S0205 | 224640 | 225986 | False | COG1388 | 8e-09 | 95.0 | 1 | 119 |
| AE014073\_S0205 | 224640 | 225986 | False | COG0741 | 7e-12 | 93.0 | 1 | 278 |
| AE014073\_S0206 | 226058 | 226813 | False | COG0491 | 1e-26 | 92.0 | 19 | 252 |
| AE014073\_S0207 | 226844 | 227569 | True | COG2226 | 7e-08 | 26.0 | 106 | 168 |
| AE014073\_S0208 | 227566 | 228033 | False | COG0328 | 1e-55 | 99.0 | 2 | 154 |
| AE014073\_S0209 | 228098 | 228829 | True | COG0847 | 4e-51 | 95.0 | 8 | 240 |
| AE014073\_S0211 | 229169 | 230215 | False | COG3515 | 6e-32 | 96.0 | 7 | 340 |
| AE014073\_S0212 | 230226 | 230339 | False | - | - | - | - | - |
| AE014073\_S0213 | 230483 | 230788 | False | COG3520 | 9e-21 | 28.0 | 13 | 109 |
| AE014073\_S0214 | 230785 | 232668 | False | COG3519 | 0.0 | 100.0 | 1 | 621 |
| AE014073\_S0215 | 232684 | 233178 | False | COG3518 | 3e-31 | 95.0 | 5 | 154 |
| AE014073\_S0217 | 233373 | 234005 | False | COG4455 | 6e-68 | 75.0 | 2 | 208 |
| AE014073\_S0218 | 233992 | 234369 | False | - | - | - | - | - |
| AE014073\_S0219 | 234607 | 234906 | False | - | - | - | - | - |
| AE014073\_S0220 | 234959 | 235369 | True | COG2963 | 1e-09 | 99.0 | 1 | 115 |
| AE014073\_S0221 | 235327 | 236232 | True | COG2801 | 2e-12 | 98.0 | 3 | 230 |
| AE014073\_S0223 | 236654 | 236956 | True | COG2963 | 7e-09 | 85.0 | 3 | 101 |
| AE014073\_S0224 | 236983 | 237822 | True | COG2801 | 2e-21 | 94.0 | 12 | 231 |
| AE014073\_S0227 | 238088 | 238294 | False | - | - | - | - | - |
| AE014073\_S0229 | 238511 | 238780 | False | COG3772 | 4e-22 | 51.0 | 74 | 152 |
