# Supplementary material for: Dissecting the bacterial type VI secretion system by a genome wide in silico analysis: what can be learned from available microbial genomic resources?
Source: BMC Genomics. 2009 Mar 12;10:104. doi: 10.1186/1471-2164-10-104 (PMC2660368; doi:10.1186/1471-2164-10-104)
Supplement: Additional file 7 — Detailed description of all identified T6SS gene clusters. Archive containing the detailed description of each identified T6SS locus as an HTML file. [file 1471-2164-10-104-S7.tgz › LociHTML/HTML/AE014075B.html]

Locus AE014075B on Escherichia coli O6 (strain UPEC / O6:H1 / ATCC 700928 / CFT073) chromosome, complete sequence.

import namespace="svg" implementation="#AdobeSVG"?


# Locus AE014075B

# List of CDS in T6SS locus AE014075B

|  |  |  |  |  |  |  |  |  |
| --- | --- | --- | --- | --- | --- | --- | --- | --- |
| Name | from | to | direct | COG | e-value | COG cover | COG hit start | COG hit end |
| AE014075\_c3379 | 3217751 | 3218044 | True | - | - | - | - | - |
| AE014075\_c3380 | 3217784 | 3218011 | False | - | - | - | - | - |
| AE014075\_c3381 | 3218167 | 3219408 | True | COG0520 | 8e-125 | 100.0 | 1 | 405 |
| AE014075\_c3382 | 3219408 | 3219851 | True | COG2166 | 3e-45 | 97.0 | 1 | 141 |
| AE014075\_c3383 | 3219902 | 3220708 | False | COG1179 | 6e-113 | 99.0 | 1 | 262 |
| AE014075\_c3384 | 3220785 | 3222218 | False | COG2821 | 3e-130 | 100.0 | 1 | 373 |
| AE014075\_c3385 | 3223002 | 3223523 | True | COG3516 | 2e-39 | 96.0 | 6 | 168 |
| AE014075\_c3386 | 3223576 | 3225120 | True | COG3517 | 0.0 | 100.0 | 1 | 495 |
| AE014075\_c3387 | 3225140 | 3226477 | True | COG3522 | 3e-94 | 99.0 | 2 | 446 |
| AE014075\_c3388 | 3226474 | 3227139 | True | COG3455 | 3e-28 | 80.0 | 43 | 254 |
| AE014075\_c3389 | 3227152 | 3228804 | True | COG2885 | 9e-27 | 83.0 | 33 | 190 |
| AE014075\_c3390 | 3228841 | 3229443 | False | - | - | - | - | - |
| AE014075\_c3391 | 3228862 | 3229353 | True | COG3157 | 3e-39 | 96.0 | 1 | 157 |
| AE014075\_c3392 | 3229545 | 3232181 | True | COG0542 | 0.0 | 99.0 | 1 | 781 |
| AE014075\_c3393 | 3232181 | 3234655 | True | COG4253 | 1e-38 | 99.0 | 2 | 277 |
| AE014075\_c3393 | 3232181 | 3234655 | True | COG3501 | 2e-86 | 97.0 | 13 | 549 |
| AE014075\_c3394 | 3234670 | 3235494 | True | - | - | - | - | - |
| AE014075\_c3395 | 3235491 | 3237467 | True | - | - | - | - | - |
| AE014075\_c3396 | 3237477 | 3237749 | True | COG4104 | 3e-08 | 90.0 | 9 | 97 |
| AE014075\_c3397 | 3237749 | 3239182 | True | - | - | - | - | - |
| AE014075\_c3398 | 3239189 | 3242527 | True | COG3523 | 5e-99 | 99.0 | 5 | 1188 |
| AE014075\_c3399 | 3242493 | 3244130 | True | COG3515 | 3e-18 | 86.0 | 9 | 309 |
| AE014075\_c3400 | 3244161 | 3246965 | True | COG3519 | 1e-134 | 99.0 | 1 | 616 |
| AE014075\_c3400 | 3244161 | 3246965 | True | COG3520 | 8e-59 | 95.0 | 14 | 334 |
| AE014075\_c3401 | 3246946 | 3247482 | True | COG3521 | 2e-15 | 86.0 | 8 | 145 |
| AE014075\_c3402 | 3247486 | 3247914 | True | COG3518 | 2e-13 | 91.0 | 7 | 150 |
| AE014075\_c3403 | 3247914 | 3249290 | True | COG3515 | 1e-11 | 46.0 | 32 | 192 |
| AE014075\_c3404 | 3248464 | 3248616 | False | - | - | - | - | - |
| AE014075\_c3405 | 3249590 | 3250546 | False | COG0111 | 3e-61 | 82.0 | 48 | 313 |
| AE014075\_c3406 | 3250609 | 3251205 | False | COG0794 | 1e-48 | 95.0 | 1 | 193 |
| AE014075\_c3407 | 3251208 | 3252383 | False | COG1168 | 2e-101 | 99.0 | 4 | 388 |
| AE014075\_c3408 | 3252383 | 3253963 | False | COG1263 | 2e-42 | 100.0 | 1 | 393 |
| AE014075\_c3408 | 3252383 | 3253963 | False | COG1264 | 1e-16 | 89.0 | 2 | 80 |
