# Supplementary material for: Dissecting the bacterial type VI secretion system by a genome wide in silico analysis: what can be learned from available microbial genomic resources?
Source: BMC Genomics. 2009 Mar 12;10:104. doi: 10.1186/1471-2164-10-104 (PMC2660368; doi:10.1186/1471-2164-10-104)
Supplement: Additional file 7 — Detailed description of all identified T6SS gene clusters. Archive containing the detailed description of each identified T6SS locus as an HTML file. [file 1471-2164-10-104-S7.tgz › LociHTML/HTML/AE014613A.html]

Locus AE014613A on Salmonella typhi (strain ATCC 700931 / Ty2) chromosome, complete sequence.

import namespace="svg" implementation="#AdobeSVG"?


# Locus AE014613A

# List of CDS in T6SS locus AE014613A

|  |  |  |  |  |  |  |  |  |
| --- | --- | --- | --- | --- | --- | --- | --- | --- |
| Name | from | to | direct | COG | e-value | COG cover | COG hit start | COG hit end |
| AE014613\_t2568 | 2645055 | 2645732 | False | - | - | - | - | - |
| AE014613\_t2569 | 2645735 | 2649799 | False | COG3209 | 3e-69 | 83.0 | 1 | 661 |
| AE014613\_t2570 | 2649818 | 2650264 | False | COG5435 | 1e-42 | 98.0 | 1 | 145 |
| AE014613\_t2571 | 2650288 | 2652477 | False | COG3501 | 9e-163 | 100.0 | 1 | 550 |
| AE014613\_t2572 | 2652840 | 2652947 | False | - | - | - | - | - |
| AE014613\_t2573 | 2652955 | 2653392 | False | - | - | - | - | - |
| AE014613\_t2574 | 2653419 | 2653835 | False | COG4893 | 3e-49 | 100.0 | 1 | 123 |
| AE014613\_t2575 | 2653963 | 2654424 | False | - | - | - | - | - |
| AE014613\_t2576 | 2654409 | 2654879 | False | - | - | - | - | - |
| AE014613\_t2577 | 2654922 | 2655689 | False | - | - | - | - | - |
| AE014613\_t2579 | 2659787 | 2660281 | False | - | - | - | - | - |
| AE014613\_t2580 | 2660313 | 2661062 | False | - | - | - | - | - |
| AE014613\_t2581 | 2661067 | 2662368 | False | COG3455 | 6e-72 | 99.0 | 1 | 261 |
| AE014613\_t2581 | 2661067 | 2662368 | False | COG1360 | 6e-32 | 58.0 | 101 | 242 |
| AE014613\_t2582 | 2662365 | 2663708 | False | COG3522 | 1e-153 | 100.0 | 1 | 446 |
| AE014613\_t2583 | 2663712 | 2664248 | False | COG3521 | 8e-43 | 97.0 | 3 | 157 |
| AE014613\_t2584 | 2664315 | 2664800 | False | COG3157 | 3e-44 | 100.0 | 1 | 162 |
| AE014613\_t2585 | 2665037 | 2665462 | False | - | - | - | - | - |
| AE014613\_t2586 | 2665434 | 2665835 | False | - | - | - | - | - |
| AE014613\_t2588 | 2667420 | 2667962 | False | COG3516 | 9e-58 | 99.0 | 2 | 169 |
| AE014613\_t2589 | 2668026 | 2668316 | False | - | - | - | - | - |
| AE014613\_t2590 | 2668319 | 2668417 | False | - | - | - | - | - |
| AE014613\_t2591 | 2668402 | 2671065 | False | COG0542 | 0.0 | 99.0 | 1 | 781 |
| AE014613\_t2592 | 2671353 | 2671424 | True | - | - | - | - | - |
| AE014613\_t2593 | 2671433 | 2672335 | True | - | - | - | - | - |
| AE014613\_t2594 | 2672322 | 2673146 | True | COG4455 | 2e-108 | 100.0 | 1 | 273 |
| AE014613\_t2595 | 2673143 | 2673637 | True | COG3518 | 5e-35 | 98.0 | 1 | 154 |
| AE014613\_t2597 | 2673653 | 2675536 | True | COG3519 | 0.0 | 100.0 | 1 | 621 |
| AE014613\_t2596 | 2675533 | 2676528 | True | COG3520 | 7e-95 | 97.0 | 11 | 335 |
| AE014613\_t2598 | 2676539 | 2676961 | True | COG3515 | 1e-19 | 39.0 | 3 | 138 |
| AE014613\_t2599 | 2676961 | 2677593 | True | COG3515 | 3e-18 | 56.0 | 145 | 340 |
| AE014613\_t2601 | 2678123 | 2678854 | False | COG0847 | 3e-51 | 95.0 | 8 | 240 |
| AE014613\_t2602 | 2678918 | 2679385 | True | COG0328 | 1e-54 | 99.0 | 2 | 154 |
| AE014613\_t2603 | 2679382 | 2680104 | False | COG2226 | 2e-07 | 36.0 | 81 | 168 |
| AE014613\_t2604 | 2680139 | 2680894 | True | COG0491 | 2e-25 | 88.0 | 19 | 241 |
| AE014613\_t2605 | 2680966 | 2682333 | True | COG1388 | 1e-07 | 95.0 | 1 | 119 |
| AE014613\_t2605 | 2680966 | 2682333 | True | COG1388 | 9e-09 | 93.0 | 1 | 116 |
| AE014613\_t2605 | 2680966 | 2682333 | True | COG0741 | 4e-13 | 95.0 | 1 | 284 |
| AE014613\_t2606 | 2682389 | 2683159 | False | COG2226 | 8e-23 | 61.0 | 18 | 164 |
