# Supplementary material for: Dissecting the bacterial type VI secretion system by a genome wide in silico analysis: what can be learned from available microbial genomic resources?
Source: BMC Genomics. 2009 Mar 12;10:104. doi: 10.1186/1471-2164-10-104 (PMC2660368; doi:10.1186/1471-2164-10-104)
Supplement: Additional file 7 — Detailed description of all identified T6SS gene clusters. Archive containing the detailed description of each identified T6SS locus as an HTML file. [file 1471-2164-10-104-S7.tgz › LociHTML/HTML/AE015451C.html]

Locus AE015451C on Pseudomonas putida (strain KT2440) chromosome, complete sequence.

import namespace="svg" implementation="#AdobeSVG"?


# Locus AE015451C

# List of CDS in T6SS locus AE015451C

|  |  |  |  |  |  |  |  |  |
| --- | --- | --- | --- | --- | --- | --- | --- | --- |
| Name | from | to | direct | COG | e-value | COG cover | COG hit start | COG hit end |
| AE015451\_PP2610 | 2984253 | 2984510 | False | COG4104 | 7e-08 | 76.0 | 16 | 90 |
| AE015451\_PP2611 | 2984507 | 2985466 | False | - | - | - | - | - |
| AE015451\_PP2612 | 2985405 | 2988395 | False | - | - | - | - | - |
| AE015451\_PP2613 | 2988388 | 2989338 | False | - | - | - | - | - |
| AE015451\_PP2614 | 2989308 | 2991476 | False | COG3501 | 1e-139 | 99.0 | 6 | 550 |
| AE015451\_PP2615 | 2991551 | 2992066 | False | COG3157 | 7e-44 | 97.0 | 1 | 158 |
| AE015451\_PP2616 | 2992200 | 2993069 | False | COG3455 | 2e-51 | 95.0 | 11 | 259 |
| AE015451\_PP2617 | 2993071 | 2994408 | False | COG3522 | 6e-132 | 100.0 | 1 | 446 |
| AE015451\_PP2618 | 2994464 | 2995261 | False | COG3521 | 2e-15 | 69.0 | 49 | 158 |
| AE015451\_PP2619 | 2995258 | 2995827 | False | COG3456 | 3e-18 | 33.0 | 2 | 144 |
| AE015451\_PP2620 | 2995824 | 2996840 | False | COG3520 | 7e-61 | 88.0 | 21 | 316 |
| AE015451\_PP2621 | 2996804 | 2998570 | False | COG3519 | 3e-143 | 99.0 | 3 | 621 |
| AE015451\_PP2622 | 2998574 | 2998984 | False | COG3518 | 7e-12 | 93.0 | 8 | 154 |
| AE015451\_PP2623 | 2998981 | 3000471 | False | COG3517 | 0.0 | 98.0 | 3 | 491 |
| AE015451\_PP2624 | 3000489 | 3000992 | False | COG3516 | 5e-39 | 99.0 | 2 | 169 |
| AE015451\_PP2625 | 3001274 | 3001969 | True | - | - | - | - | - |
| AE015451\_PP2626 | 3001957 | 3003420 | True | - | - | - | - | - |
| AE015451\_PP2627 | 3003417 | 3007037 | True | COG3523 | 0.0 | 98.0 | 11 | 1184 |
| AE015451\_PP2628 | 3007115 | 3009091 | False | COG1132 | 8e-119 | 97.0 | 15 | 567 |
| AE015451\_PP2629 | 3009133 | 3009903 | True | - | - | - | - | - |
| AE015451\_PP2630 | 3009908 | 3010762 | True | - | - | - | - | - |
| AE015451\_PP2631 | 3010759 | 3010944 | True | - | - | - | - | - |
| AE015451\_PP2632 | 3010937 | 3012550 | True | - | - | - | - | - |
