# Supplementary material for: Dissecting the bacterial type VI secretion system by a genome wide in silico analysis: what can be learned from available microbial genomic resources?
Source: BMC Genomics. 2009 Mar 12;10:104. doi: 10.1186/1471-2164-10-104 (PMC2660368; doi:10.1186/1471-2164-10-104)
Supplement: Additional file 7 — Detailed description of all identified T6SS gene clusters. Archive containing the detailed description of each identified T6SS locus as an HTML file. [file 1471-2164-10-104-S7.tgz › LociHTML/HTML/AE015451D.html]

Locus AE015451D on Pseudomonas putida (strain KT2440) chromosome, complete sequence.

import namespace="svg" implementation="#AdobeSVG"?


# Locus AE015451D

# List of CDS in T6SS locus AE015451D

|  |  |  |  |  |  |  |  |  |
| --- | --- | --- | --- | --- | --- | --- | --- | --- |
| Name | from | to | direct | COG | e-value | COG cover | COG hit start | COG hit end |
| AE015451\_PP3084 | 3470069 | 3472528 | False | COG1629 | 7e-34 | 96.0 | 28 | 768 |
| AE015451\_PP3085 | 3472636 | 3473601 | False | COG3712 | 4e-57 | 96.0 | 10 | 321 |
| AE015451\_PP3086 | 3473598 | 3474104 | False | COG1595 | 4e-23 | 85.0 | 17 | 171 |
| AE015451\_PP3087 | 3474193 | 3476709 | False | COG0178 | 0.0 | 88.0 | 109 | 933 |
| AE015451\_PP3088 | 3476877 | 3477962 | False | COG3515 | 1e-17 | 90.0 | 19 | 331 |
| AE015451\_PP3089 | 3478058 | 3478600 | False | COG3157 | 3e-26 | 98.0 | 1 | 160 |
| AE015451\_PP3090 | 3478720 | 3481215 | False | COG3523 | 3e-90 | 50.0 | 9 | 602 |
| AE015451\_PP3090 | 3478720 | 3481215 | False | COG2885 | 7e-19 | 65.0 | 66 | 189 |
| AE015451\_PP3091 | 3482117 | 3485920 | False | COG3523 | 8e-76 | 47.0 | 5 | 565 |
| AE015451\_PP3091 | 3482117 | 3485920 | False | COG3523 | 1e-48 | 51.0 | 552 | 1166 |
| AE015451\_PP3092 | 3485951 | 3486667 | False | COG3455 | 1e-28 | 83.0 | 39 | 258 |
| AE015451\_PP3093 | 3486664 | 3488007 | False | COG3522 | 2e-93 | 99.0 | 1 | 444 |
| AE015451\_PP3094 | 3488004 | 3488726 | False | COG3521 | 6e-16 | 95.0 | 5 | 156 |
| AE015451\_PP3095 | 3488751 | 3491387 | False | COG0542 | 0.0 | 97.0 | 1 | 766 |
| AE015451\_PP3096 | 3491345 | 3492415 | False | COG3520 | 2e-68 | 96.0 | 1 | 323 |
| AE015451\_PP3097 | 3492379 | 3494199 | False | COG3519 | 2e-146 | 100.0 | 1 | 621 |
| AE015451\_PP3098 | 3494199 | 3494681 | False | COG3518 | 2e-19 | 98.0 | 1 | 154 |
| AE015451\_PP3099 | 3494708 | 3496210 | False | COG3517 | 0.0 | 99.0 | 1 | 492 |
| AE015451\_PP3100 | 3496225 | 3496800 | False | COG3516 | 5e-52 | 95.0 | 2 | 162 |
| AE015451\_PP3101 | 3498998 | 3499780 | True | COG1397 | 4e-22 | 74.0 | 74 | 308 |
