# Supplementary material for: Dissecting the bacterial type VI secretion system by a genome wide in silico analysis: what can be learned from available microbial genomic resources?
Source: BMC Genomics. 2009 Mar 12;10:104. doi: 10.1186/1471-2164-10-104 (PMC2660368; doi:10.1186/1471-2164-10-104)
Supplement: Additional file 7 — Detailed description of all identified T6SS gene clusters. Archive containing the detailed description of each identified T6SS locus as an HTML file. [file 1471-2164-10-104-S7.tgz › LociHTML/HTML/AE015451F.html]

Locus AE015451F on Pseudomonas putida (strain KT2440) chromosome, complete sequence.

import namespace="svg" implementation="#AdobeSVG"?


# Locus AE015451F

# List of CDS in T6SS locus AE015451F

|  |  |  |  |  |  |  |  |  |
| --- | --- | --- | --- | --- | --- | --- | --- | --- |
| Name | from | to | direct | COG | e-value | COG cover | COG hit start | COG hit end |
| AE015451\_PP4066 | 4590927 | 4591742 | True | COG1024 | 2e-42 | 100.0 | 1 | 257 |
| AE015451\_PP4067 | 4591739 | 4593691 | True | COG4770 | 0.0 | 99.0 | 2 | 645 |
| AE015451\_PP4068 | 4593990 | 4594694 | False | COG1974 | 2e-14 | 95.0 | 6 | 196 |
| AE015451\_PP4069 | 4594782 | 4595081 | True | - | - | - | - | - |
| AE015451\_PP4070 | 4595530 | 4596045 | True | - | - | - | - | - |
| AE015451\_PP4071 | 4596179 | 4599805 | False | COG3523 | 0.0 | 98.0 | 11 | 1184 |
| AE015451\_PP4072 | 4599802 | 4601268 | False | - | - | - | - | - |
| AE015451\_PP4073 | 4601256 | 4601888 | False | - | - | - | - | - |
| AE015451\_PP4074 | 4602163 | 4602666 | True | COG3516 | 5e-37 | 94.0 | 9 | 168 |
| AE015451\_PP4076 | 4604168 | 4604578 | True | COG3518 | 9e-11 | 93.0 | 8 | 154 |
| AE015451\_PP4077 | 4604582 | 4606348 | True | COG3519 | 2e-142 | 99.0 | 3 | 621 |
| AE015451\_PP4078 | 4606312 | 4607328 | True | COG3520 | 2e-62 | 94.0 | 15 | 331 |
| AE015451\_PP4079 | 4607325 | 4608122 | True | COG3521 | 1e-16 | 86.0 | 21 | 158 |
| AE015451\_PP4080 | 4608172 | 4609509 | True | COG3522 | 9e-130 | 100.0 | 1 | 446 |
| AE015451\_PP4081 | 4609454 | 4610386 | True | COG3455 | 5e-51 | 96.0 | 7 | 259 |
| AE015451\_PP4082 | 4610520 | 4611035 | True | COG3157 | 1e-43 | 97.0 | 1 | 158 |
| AE015451\_PP4084 | 4613142 | 4613573 | True | - | - | - | - | - |
| AE015451\_PP4085 | 4613627 | 4618219 | True | COG3209 | 4e-29 | 85.0 | 2 | 680 |
| AE015451\_PP4085 | 4613627 | 4618219 | True | COG4104 | 1e-06 | 43.0 | 45 | 87 |
