# Supplementary material for: Dissecting the bacterial type VI secretion system by a genome wide in silico analysis: what can be learned from available microbial genomic resources?
Source: BMC Genomics. 2009 Mar 12;10:104. doi: 10.1186/1471-2164-10-104 (PMC2660368; doi:10.1186/1471-2164-10-104)
Supplement: Additional file 7 — Detailed description of all identified T6SS gene clusters. Archive containing the detailed description of each identified T6SS locus as an HTML file. [file 1471-2164-10-104-S7.tgz › LociHTML/HTML/AE016796C.html]

Locus AE016796C on Vibrio vulnificus (strain CMCP6) chromosome 2, complete sequence.

import namespace="svg" implementation="#AdobeSVG"?


# Locus AE016796C

# List of CDS in T6SS locus AE016796C

|  |  |  |  |  |  |  |  |  |
| --- | --- | --- | --- | --- | --- | --- | --- | --- |
| Name | from | to | direct | COG | e-value | COG cover | COG hit start | COG hit end |
| AE016796\_VV2\_0425 | 454915 | 459348 | False | COG3209 | 5e-36 | 88.0 | 1 | 708 |
| AE016796\_VV2\_0426 | 459349 | 460257 | False | - | - | - | - | - |
| AE016796\_VV2\_0427 | 460258 | 460548 | False | COG4104 | 1e-13 | 82.0 | 18 | 98 |
| AE016796\_VV2\_0428 | 460558 | 462297 | False | COG3501 | 5e-102 | 93.0 | 37 | 549 |
| AE016796\_VV2\_0429 | 462448 | 462927 | False | COG3157 | 1e-14 | 92.0 | 1 | 150 |
| AE016796\_VV2\_0430 | 463143 | 465707 | False | COG0542 | 0.0 | 99.0 | 1 | 784 |
| AE016796\_VV2\_0431 | 465721 | 466689 | False | COG3520 | 2e-35 | 90.0 | 16 | 317 |
| AE016796\_VV2\_0432 | 466686 | 468512 | False | COG3519 | 9e-100 | 100.0 | 1 | 621 |
| AE016796\_VV2\_0433 | 468509 | 468982 | False | COG3518 | 6e-16 | 95.0 | 6 | 155 |
| AE016796\_VV2\_0434 | 468979 | 469782 | False | COG4455 | 8e-31 | 91.0 | 12 | 261 |
| AE016796\_VV2\_0435 | 469793 | 471286 | False | COG3517 | 5e-121 | 86.0 | 67 | 493 |
| AE016796\_VV2\_0436 | 471331 | 472824 | False | COG3517 | 0.0 | 98.0 | 6 | 492 |
| AE016796\_VV2\_0437 | 472836 | 473348 | False | COG3516 | 6e-48 | 97.0 | 1 | 165 |
| AE016796\_VV2\_0438 | 473365 | 474498 | False | COG3515 | 8e-16 | 97.0 | 8 | 344 |
| AE016796\_VV2\_0439 | 474498 | 475331 | False | COG0631 | 8e-51 | 95.0 | 7 | 256 |
| AE016796\_VV2\_0440 | 475303 | 475998 | False | COG3913 | 4e-13 | 96.0 | 1 | 218 |
| AE016796\_VV2\_0441 | 475980 | 479495 | False | COG3523 | 0.0 | 99.0 | 5 | 1187 |
| AE016796\_VV2\_0442 | 479482 | 480810 | False | COG1360 | 1e-25 | 86.0 | 34 | 244 |
| AE016796\_VV2\_0442 | 479482 | 480810 | False | COG3455 | 1e-45 | 85.0 | 39 | 261 |
| AE016796\_VV2\_0443 | 480819 | 482105 | False | COG3522 | 2e-101 | 97.0 | 13 | 446 |
| AE016796\_VV2\_0444 | 482165 | 482620 | False | COG3521 | 9e-26 | 81.0 | 16 | 145 |
| AE016796\_VV2\_0445 | 482621 | 483745 | False | COG3456 | 4e-42 | 93.0 | 31 | 430 |
| AE016796\_VV2\_0446 | 484385 | 486322 | True | COG0515 | 3e-26 | 96.0 | 2 | 372 |
| AE016796\_VV2\_0447 | 486386 | 487003 | False | COG0625 | 3e-29 | 95.0 | 1 | 201 |
| AE016796\_VV2\_0448 | 487104 | 487691 | True | COG1309 | 2e-07 | 91.0 | 6 | 188 |
| AE016796\_VV2\_0449 | 487688 | 488086 | True | COG1661 | 7e-20 | 95.0 | 7 | 141 |
| AE016796\_VV2\_0450 | 488090 | 488353 | False | COG3530 | 2e-23 | 97.0 | 1 | 69 |
| AE016796\_VV2\_0451 | 488353 | 488583 | False | - | - | - | - | - |
| AE016796\_VV2\_0452 | 488711 | 489055 | False | - | - | - | - | - |
