# Supplementary material for: Dissecting the bacterial type VI secretion system by a genome wide in silico analysis: what can be learned from available microbial genomic resources?
Source: BMC Genomics. 2009 Mar 12;10:104. doi: 10.1186/1471-2164-10-104 (PMC2660368; doi:10.1186/1471-2164-10-104)
Supplement: Additional file 7 — Detailed description of all identified T6SS gene clusters. Archive containing the detailed description of each identified T6SS locus as an HTML file. [file 1471-2164-10-104-S7.tgz › LociHTML/HTML/AE016825C.html]

Locus AE016825C on Chromobacterium violaceum (strain IFO 12614 / ATCC 12472 / DSM 30191 / NCIB 9131 / JCM 1249) chromosome, complete sequence.

import namespace="svg" implementation="#AdobeSVG"?


# Locus AE016825C

# List of CDS in T6SS locus AE016825C

|  |  |  |  |  |  |  |  |  |
| --- | --- | --- | --- | --- | --- | --- | --- | --- |
| Name | from | to | direct | COG | e-value | COG cover | COG hit start | COG hit end |
| AE016825\_CV\_3958 | 4278258 | 4279397 | False | COG0142 | 6e-25 | 97.0 | 6 | 319 |
| AE016825\_CV\_3959 | 4279442 | 4281430 | False | COG0367 | 4e-83 | 95.0 | 1 | 517 |
| AE016825\_CV\_3960 | 4281435 | 4282250 | False | COG1608 | 6e-30 | 98.0 | 3 | 250 |
| AE016825\_CV\_3961 | 4282261 | 4282986 | False | COG1891 | 2e-69 | 93.0 | 1 | 219 |
| AE016825\_CV\_3962 | 4283454 | 4283612 | True | - | - | - | - | - |
| AE016825\_CV\_3963 | 4283686 | 4284771 | False | COG3515 | 1e-22 | 97.0 | 7 | 342 |
| AE016825\_CV\_3964 | 4285738 | 4286475 | False | - | - | - | - | - |
| AE016825\_CV\_3965 | 4286490 | 4289117 | False | COG0542 | 0.0 | 99.0 | 1 | 782 |
| AE016825\_CV\_3966 | 4289166 | 4289894 | False | - | - | - | - | - |
| AE016825\_CV\_3967 | 4289891 | 4290934 | False | COG3520 | 5e-52 | 93.0 | 15 | 326 |
| AE016825\_CV\_3968 | 4290915 | 4291214 | False | - | - | - | - | - |
| AE016825\_CV\_3969 | 4291222 | 4293081 | False | COG3519 | 1e-144 | 100.0 | 1 | 621 |
| AE016825\_CV\_3970 | 4293147 | 4293836 | False | - | - | - | - | - |
| AE016825\_CV\_3971 | 4293888 | 4295963 | False | - | - | - | - | - |
| AE016825\_CV\_3972 | 4295951 | 4296499 | False | - | - | - | - | - |
| AE016825\_CV\_3973 | 4296545 | 4297087 | False | - | - | - | - | - |
| AE016825\_CV\_3974 | 4297084 | 4297857 | False | - | - | - | - | - |
| AE016825\_CV\_3975 | 4297872 | 4300490 | False | COG4253 | 1e-40 | 79.0 | 1 | 221 |
| AE016825\_CV\_3975 | 4297872 | 4300490 | False | COG3501 | 2e-118 | 96.0 | 4 | 531 |
| AE016825\_CV\_3976 | 4300599 | 4301036 | False | - | - | - | - | - |
| AE016825\_CV\_3977 | 4301111 | 4301608 | False | COG3157 | 2e-33 | 100.0 | 1 | 162 |
| AE016825\_CV\_3978 | 4301678 | 4303156 | False | COG3517 | 0.0 | 99.0 | 4 | 495 |
| AE016825\_CV\_3979 | 4303153 | 4303659 | False | COG3516 | 2e-50 | 97.0 | 2 | 166 |
| AE016825\_CV\_3980 | 4304734 | 4308516 | False | COG3523 | 3e-91 | 46.0 | 16 | 565 |
| AE016825\_CV\_3980 | 4304734 | 4308516 | False | COG3523 | 3e-59 | 52.0 | 565 | 1186 |
| AE016825\_CV\_3981 | 4308545 | 4309402 | False | - | - | - | - | - |
| AE016825\_CV\_3982 | 4309554 | 4310102 | True | COG3521 | 2e-15 | 89.0 | 5 | 146 |
| AE016825\_CV\_3983 | 4310149 | 4311495 | True | COG3522 | 7e-82 | 99.0 | 1 | 444 |
| AE016825\_CV\_3984 | 4311503 | 4312204 | True | COG3455 | 1e-32 | 80.0 | 37 | 248 |
| AE016825\_CV\_3985 | 4312201 | 4312680 | True | COG3518 | 7e-21 | 93.0 | 8 | 154 |
| AE016825\_CV\_3986 | 4312701 | 4315319 | True | COG4253 | 4e-41 | 85.0 | 1 | 237 |
| AE016825\_CV\_3986 | 4312701 | 4315319 | True | COG3501 | 1e-121 | 96.0 | 4 | 531 |
| AE016825\_CV\_3987 | 4315330 | 4316205 | True | - | - | - | - | - |
| AE016825\_CV\_3988 | 4316209 | 4316565 | True | - | - | - | - | - |
| AE016825\_CV\_3989 | 4316633 | 4317145 | True | - | - | - | - | - |
| AE016825\_CV\_3990 | 4317223 | 4318794 | True | - | - | - | - | - |
| AE016825\_CV\_3991 | 4318820 | 4320046 | True | - | - | - | - | - |
| AE016825\_CV\_3992 | 4320113 | 4321021 | False | COG0109 | 1e-80 | 92.0 | 16 | 296 |
