# Supplementary material for: Dissecting the bacterial type VI secretion system by a genome wide in silico analysis: what can be learned from available microbial genomic resources?
Source: BMC Genomics. 2009 Mar 12;10:104. doi: 10.1186/1471-2164-10-104 (PMC2660368; doi:10.1186/1471-2164-10-104)
Supplement: Additional file 7 — Detailed description of all identified T6SS gene clusters. Archive containing the detailed description of each identified T6SS locus as an HTML file. [file 1471-2164-10-104-S7.tgz › LociHTML/HTML/AE016853A.html]

Locus AE016853A on Pseudomonas syringae tomato (strain DC3000) chromosome, complete sequence.

import namespace="svg" implementation="#AdobeSVG"?


# Locus AE016853A

# List of CDS in T6SS locus AE016853A

|  |  |  |  |  |  |  |  |  |
| --- | --- | --- | --- | --- | --- | --- | --- | --- |
| Name | from | to | direct | COG | e-value | COG cover | COG hit start | COG hit end |
| AE016853\_PSPTO2534 | 2796816 | 2797496 | False | - | - | - | - | - |
| AE016853\_PSPTO2535 | 2797493 | 2800933 | False | - | - | - | - | - |
| AE016853\_PSPTO2536 | 2800881 | 2801324 | False | - | - | - | - | - |
| AE016853\_PSPTO2537 | 2801296 | 2801763 | False | - | - | - | - | - |
| AE016853\_PSPTO2538 | 2801756 | 2803798 | False | COG3501 | 3e-145 | 94.0 | 12 | 531 |
| AE016853\_PSPTO2539 | 2803874 | 2804389 | False | COG3157 | 7e-29 | 96.0 | 1 | 156 |
| AE016853\_PSPTO2541 | 2806011 | 2806988 | False | COG3039 | 2e-24 | 99.0 | 1 | 229 |
| AE016853\_PSPTO2543 | 2807665 | 2808165 | True | COG3516 | 4e-39 | 97.0 | 5 | 169 |
| AE016853\_PSPTO2544 | 2808195 | 2809670 | True | COG3517 | 0.0 | 98.0 | 6 | 493 |
| AE016853\_PSPTO2545 | 2809686 | 2810093 | True | COG3518 | 4e-15 | 82.0 | 14 | 143 |
| AE016853\_PSPTO2546 | 2810191 | 2811978 | True | COG3519 | 4e-147 | 99.0 | 3 | 621 |
| AE016853\_PSPTO2547 | 2811942 | 2812949 | True | COG3520 | 7e-75 | 97.0 | 1 | 328 |
| AE016853\_PSPTO2548 | 2812959 | 2815562 | True | COG0542 | 0.0 | 96.0 | 2 | 761 |
| AE016853\_PSPTO2549 | 2815564 | 2817093 | True | COG3604 | 9e-113 | 95.0 | 19 | 543 |
| AE016853\_PSPTO2550 | 2817143 | 2817283 | True | - | - | - | - | - |
| AE016853\_PSPTO2551 | 2817337 | 2817828 | True | COG3521 | 3e-30 | 88.0 | 1 | 140 |
| AE016853\_PSPTO2552 | 2817858 | 2819156 | True | COG3522 | 1e-122 | 97.0 | 13 | 446 |
| AE016853\_PSPTO2553 | 2819156 | 2820028 | True | COG3455 | 1e-57 | 92.0 | 16 | 257 |
| AE016853\_PSPTO2554 | 2820042 | 2823548 | True | COG3523 | 0.0 | 99.0 | 8 | 1187 |
| AE016853\_PSPTO\_2555 | 2823826 | 2824659 | True | COG3638 | 1e-86 | 94.0 | 2 | 246 |
| AE016853\_PSPTO2556 | 2824697 | 2825707 | True | COG3221 | 3e-66 | 97.0 | 1 | 291 |
| AE016853\_PSPTO2557 | 2825735 | 2826523 | True | COG3639 | 9e-65 | 92.0 | 21 | 282 |
| AE016853\_PSPTO2558 | 2826562 | 2827281 | True | COG2188 | 7e-42 | 98.0 | 2 | 234 |
| AE016853\_PSPTO2559 | 2827296 | 2827760 | True | COG3624 | 6e-41 | 100.0 | 1 | 151 |
| AE016853\_PSPTO2560 | 2827760 | 2828356 | True | COG3625 | 2e-50 | 99.0 | 1 | 195 |
| AE016853\_PSPTO2561 | 2828356 | 2829435 | True | COG3626 | 2e-163 | 97.0 | 1 | 358 |
