# Supplementary material for: Dissecting the bacterial type VI secretion system by a genome wide in silico analysis: what can be learned from available microbial genomic resources?
Source: BMC Genomics. 2009 Mar 12;10:104. doi: 10.1186/1471-2164-10-104 (PMC2660368; doi:10.1186/1471-2164-10-104)
Supplement: Additional file 7 — Detailed description of all identified T6SS gene clusters. Archive containing the detailed description of each identified T6SS locus as an HTML file. [file 1471-2164-10-104-S7.tgz › LociHTML/HTML/AE016853C.html]

Locus AE016853C on Pseudomonas syringae tomato (strain DC3000) chromosome, complete sequence.

import namespace="svg" implementation="#AdobeSVG"?


# Locus AE016853C

# List of CDS in T6SS locus AE016853C

|  |  |  |  |  |  |  |  |  |
| --- | --- | --- | --- | --- | --- | --- | --- | --- |
| Name | from | to | direct | COG | e-value | COG cover | COG hit start | COG hit end |
| AE016853\_PSPTO5413 | 6149242 | 6151620 | False | - | - | - | - | - |
| AE016853\_PSPTO5414 | 6152531 | 6153547 | False | COG0790 | 1e-08 | 52.0 | 93 | 245 |
| AE016853\_PSPTO5415 | 6153553 | 6155589 | False | COG3501 | 4e-166 | 99.0 | 5 | 550 |
| AE016853\_PSPTO5416 | 6155611 | 6156606 | False | COG0515 | 1e-31 | 71.0 | 2 | 277 |
| AE016853\_PSPTO5417 | 6156615 | 6157343 | False | COG0631 | 1e-54 | 93.0 | 6 | 250 |
| AE016853\_PSPTO5418 | 6157343 | 6160921 | False | COG3523 | 0.0 | 99.0 | 7 | 1187 |
| AE016853\_PSPTO5419 | 6160884 | 6161759 | False | COG3455 | 2e-68 | 97.0 | 7 | 262 |
| AE016853\_PSPTO5420 | 6161765 | 6163096 | False | COG3522 | 1e-146 | 99.0 | 2 | 446 |
| AE016853\_PSPTO5421 | 6163099 | 6163599 | False | COG3521 | 2e-36 | 95.0 | 8 | 159 |
| AE016853\_PSPTO5422 | 6163605 | 6164801 | False | COG3456 | 5e-72 | 100.0 | 1 | 430 |
| AE016853\_PSPTO5423 | 6164819 | 6164983 | False | - | - | - | - | - |
| AE016853\_PSPTO5424 | 6165047 | 6166564 | False | COG3604 | 1e-130 | 94.0 | 30 | 550 |
| AE016853\_PSPTO5425 | 6166575 | 6169223 | False | COG0542 | 4e-123 | 57.0 | 2 | 453 |
| AE016853\_PSPTO5425 | 6166575 | 6169223 | False | COG0542 | 4e-99 | 50.0 | 367 | 766 |
| AE016853\_PSPTO5426 | 6169237 | 6170244 | False | COG3520 | 2e-82 | 99.0 | 1 | 333 |
| AE016853\_PSPTO5427 | 6170208 | 6171761 | False | COG3519 | 4e-134 | 86.0 | 84 | 621 |
| AE016853\_PSPTO5428 | 6173232 | 6174209 | False | COG3039 | 2e-24 | 99.0 | 1 | 229 |
| AE016853\_PSPTO5429 | 6175026 | 6175142 | True | - | - | - | - | - |
| AE016853\_PSPTO5430 | 6175762 | 6176286 | False | COG4104 | 6e-12 | 96.0 | 4 | 98 |
| AE016853\_PSPTO5431 | 6176297 | 6176704 | False | COG3518 | 1e-24 | 89.0 | 14 | 154 |
| AE016853\_PSPTO5432 | 6176718 | 6178196 | False | COG3517 | 0.0 | 99.0 | 1 | 493 |
| AE016853\_PSPTO5433 | 6178225 | 6178731 | False | COG3516 | 2e-42 | 97.0 | 5 | 169 |
| AE016853\_PSPTO5434 | 6178764 | 6180320 | False | COG3515 | 3e-22 | 100.0 | 1 | 346 |
| AE016853\_PSPTO5435 | 6181106 | 6181624 | True | COG3157 | 3e-46 | 97.0 | 1 | 158 |
| AE016853\_PSPTO5436 | 6181756 | 6183798 | True | COG3501 | 7e-173 | 99.0 | 5 | 550 |
| AE016853\_PSPTO5437 | 6183842 | 6184525 | True | - | - | - | - | - |
| AE016853\_PSPTO5438 | 6184525 | 6189243 | True | COG3209 | 9e-41 | 82.0 | 6 | 660 |
