# Supplementary material for: Dissecting the bacterial type VI secretion system by a genome wide in silico analysis: what can be learned from available microbial genomic resources?
Source: BMC Genomics. 2009 Mar 12;10:104. doi: 10.1186/1471-2164-10-104 (PMC2660368; doi:10.1186/1471-2164-10-104)
Supplement: Additional file 7 — Detailed description of all identified T6SS gene clusters. Archive containing the detailed description of each identified T6SS locus as an HTML file. [file 1471-2164-10-104-S7.tgz › LociHTML/HTML/AE017042A.html]

Locus AE017042A on Yersinia pestis (biovar Mediaevalis, strain 91001) chromosome, complete sequence.

import namespace="svg" implementation="#AdobeSVG"?


# Locus AE017042A

# List of CDS in T6SS locus AE017042A

|  |  |  |  |  |  |  |  |  |
| --- | --- | --- | --- | --- | --- | --- | --- | --- |
| Name | from | to | direct | COG | e-value | COG cover | COG hit start | COG hit end |
| AE017042\_YP\_1350 | 1480241 | 1481011 | True | COG1024 | 7e-29 | 87.0 | 29 | 252 |
| AE017042\_YP\_1351 | 1480989 | 1481765 | True | COG1024 | 5e-37 | 93.0 | 1 | 240 |
| AE017042\_YP\_1352 | 1481768 | 1482556 | True | COG1028 | 4e-25 | 99.0 | 2 | 250 |
| AE017042\_YP\_1353 | 1482595 | 1483083 | True | - | - | - | - | - |
| AE017042\_YP\_1354 | 1483121 | 1483369 | True | - | - | - | - | - |
| AE017042\_YP\_1355 | 1483468 | 1484316 | True | COG0331 | 2e-74 | 93.0 | 2 | 290 |
| AE017042\_YP\_1356 | 1485302 | 1485805 | True | COG3516 | 1e-48 | 100.0 | 1 | 169 |
| AE017042\_YP\_1357 | 1485848 | 1487392 | True | COG3517 | 0.0 | 100.0 | 1 | 495 |
| AE017042\_YP\_1358 | 1487404 | 1488756 | True | COG3522 | 6e-133 | 99.0 | 2 | 446 |
| AE017042\_YP\_1359 | 1488753 | 1489439 | True | COG3455 | 2e-48 | 91.0 | 21 | 260 |
| AE017042\_YP\_1360 | 1489439 | 1491175 | True | COG2885 | 5e-27 | 94.0 | 12 | 190 |
| AE017042\_YP\_1361 | 1491179 | 1491670 | True | COG3157 | 2e-40 | 98.0 | 1 | 160 |
| AE017042\_YP\_1362 | 1492058 | 1494700 | True | COG0542 | 0.0 | 99.0 | 1 | 784 |
| AE017042\_YP\_1363 | 1494703 | 1497051 | True | COG3501 | 1e-105 | 99.0 | 1 | 549 |
| AE017042\_YP\_1363 | 1494703 | 1497051 | True | COG4253 | 6e-67 | 82.0 | 2 | 229 |
| AE017042\_YP\_1364 | 1497067 | 1499367 | True | - | - | - | - | - |
| AE017042\_YP\_1365 | 1499364 | 1500137 | True | - | - | - | - | - |
| AE017042\_YP\_1366 | 1500291 | 1500551 | True | COG4253 | 2e-25 | 30.0 | 144 | 229 |
| AE017042\_YP\_1367 | 1500567 | 1502750 | True | - | - | - | - | - |
| AE017042\_YP\_1368 | 1502923 | 1503393 | True | - | - | - | - | - |
| AE017042\_YP1369 | 1504126 | 1504446 | False | - | - | - | - | - |
| AE017042\_YP\_1370 | 1505669 | 1506790 | True | - | - | - | - | - |
| AE017042\_YP\_1371 | 1506787 | 1510209 | True | COG3523 | 0.0 | 100.0 | 1 | 1188 |
| AE017042\_YP\_1373 | 1511844 | 1512929 | True | - | - | - | - | - |
| AE017042\_YP\_1374 | 1512929 | 1513399 | True | - | - | - | - | - |
| AE017042\_YP\_1375 | 1513620 | 1515383 | True | COG3519 | 0.0 | 100.0 | 1 | 621 |
| AE017042\_YP\_1376 | 1515347 | 1516432 | True | COG3520 | 3e-85 | 97.0 | 1 | 328 |
| AE017042\_YP\_1377 | 1516407 | 1516988 | True | COG3521 | 3e-35 | 98.0 | 1 | 157 |
| AE017042\_YP\_1378 | 1516988 | 1517440 | True | COG3518 | 1e-27 | 98.0 | 3 | 157 |
| AE017042\_YP\_1379 | 1517465 | 1518832 | True | COG3515 | 9e-41 | 96.0 | 13 | 346 |
| AE017042\_YP\_1380 | 1519044 | 1519667 | False | - | - | - | - | - |
| AE017042\_YP\_1381 | 1520182 | 1521945 | True | COG0488 | 8e-172 | 99.0 | 1 | 528 |
| AE017042\_YP\_1382 | 1522173 | 1522817 | True | - | - | - | - | - |
| AE017042\_YP\_1383 | 1522839 | 1524047 | False | COG3328 | 2e-112 | 98.0 | 1 | 375 |
