# Supplementary material for: Dissecting the bacterial type VI secretion system by a genome wide in silico analysis: what can be learned from available microbial genomic resources?
Source: BMC Genomics. 2009 Mar 12;10:104. doi: 10.1186/1471-2164-10-104 (PMC2660368; doi:10.1186/1471-2164-10-104)
Supplement: Additional file 7 — Detailed description of all identified T6SS gene clusters. Archive containing the detailed description of each identified T6SS locus as an HTML file. [file 1471-2164-10-104-S7.tgz › LociHTML/HTML/AE017042E.html]

Locus AE017042E on Yersinia pestis (biovar Mediaevalis, strain 91001) chromosome, complete sequence.

import namespace="svg" implementation="#AdobeSVG"?


# Locus AE017042E

# List of CDS in T6SS locus AE017042E

|  |  |  |  |  |  |  |  |  |
| --- | --- | --- | --- | --- | --- | --- | --- | --- |
| Name | from | to | direct | COG | e-value | COG cover | COG hit start | COG hit end |
| AE017042\_YP\_2752 | 3054348 | 3058832 | True | COG3468 | 8e-14 | 95.0 | 30 | 592 |
| AE017042\_YP\_2752 | 3054348 | 3058832 | True | COG3468 | 5e-09 | 58.0 | 233 | 578 |
| AE017042\_YP\_2754 | 3060117 | 3061325 | False | COG3328 | 2e-112 | 98.0 | 1 | 375 |
| AE017042\_YP\_2756 | 3062525 | 3063133 | True | COG3539 | 4e-16 | 91.0 | 16 | 184 |
| AE017042\_YP\_2757 | 3063217 | 3063762 | True | COG3516 | 3e-59 | 99.0 | 2 | 169 |
| AE017042\_YP\_2758 | 3063765 | 3065288 | True | COG3517 | 0.0 | 100.0 | 1 | 495 |
| AE017042\_YP\_2759 | 3065530 | 3066099 | True | COG3157 | 4e-42 | 100.0 | 1 | 162 |
| AE017042\_YP\_2760 | 3066313 | 3066912 | True | COG3521 | 2e-39 | 100.0 | 1 | 159 |
| AE017042\_YP\_2761 | 3066916 | 3068265 | True | COG3522 | 3e-158 | 99.0 | 2 | 446 |
| AE017042\_YP\_2763 | 3068944 | 3069966 | True | COG4584 | 2e-58 | 100.0 | 1 | 278 |
| AE017042\_YP\_2764 | 3069963 | 3070745 | True | COG1484 | 2e-64 | 100.0 | 1 | 254 |
| AE017042\_YP\_2766 | 3071341 | 3072852 | True | COG1953 | 4e-132 | 97.0 | 8 | 493 |
| AE017042\_YP\_2767 | 3073098 | 3073862 | False | COG0847 | 6e-50 | 96.0 | 8 | 242 |
