# Supplementary material for: Dissecting the bacterial type VI secretion system by a genome wide in silico analysis: what can be learned from available microbial genomic resources?
Source: BMC Genomics. 2009 Mar 12;10:104. doi: 10.1186/1471-2164-10-104 (PMC2660368; doi:10.1186/1471-2164-10-104)
Supplement: Additional file 7 — Detailed description of all identified T6SS gene clusters. Archive containing the detailed description of each identified T6SS locus as an HTML file. [file 1471-2164-10-104-S7.tgz › LociHTML/HTML/AE017042G.html]

Locus AE017042G on Yersinia pestis (biovar Mediaevalis, strain 91001) chromosome, complete sequence.

import namespace="svg" implementation="#AdobeSVG"?


# Locus AE017042G

# List of CDS in T6SS locus AE017042G

|  |  |  |  |  |  |  |  |  |
| --- | --- | --- | --- | --- | --- | --- | --- | --- |
| Name | from | to | direct | COG | e-value | COG cover | COG hit start | COG hit end |
| AE017042\_YP\_3455 | 3927235 | 3927885 | False | COG3916 | 3e-64 | 100.0 | 1 | 209 |
| AE017042\_YP\_3456 | 3929055 | 3929705 | True | - | - | - | - | - |
| AE017042\_YP\_3457 | 3930156 | 3930551 | True | - | - | - | - | - |
| AE017042\_YP\_3458 | 3930613 | 3930882 | True | COG3677 | 2e-18 | 68.0 | 26 | 114 |
| AE017042\_YP\_3460 | 3931056 | 3931427 | True | - | - | - | - | - |
| AE017042\_YP\_3461 | 3932824 | 3933327 | True | COG3516 | 1e-48 | 100.0 | 1 | 169 |
| AE017042\_YP\_3462 | 3933370 | 3934920 | True | COG3517 | 0.0 | 100.0 | 1 | 495 |
| AE017042\_YP\_3463 | 3934932 | 3936284 | True | COG3522 | 4e-132 | 99.0 | 2 | 446 |
| AE017042\_YP\_3464 | 3936281 | 3936967 | True | COG3455 | 2e-48 | 91.0 | 21 | 260 |
| AE017042\_YP\_3465 | 3936967 | 3938703 | True | COG2885 | 8e-27 | 94.0 | 12 | 190 |
| AE017042\_YP\_3466 | 3938707 | 3939198 | True | COG3157 | 2e-40 | 98.0 | 1 | 160 |
| AE017042\_YP\_3467 | 3939616 | 3942264 | True | COG0542 | 0.0 | 100.0 | 1 | 786 |
| AE017042\_YP\_3468 | 3942261 | 3944609 | True | COG3501 | 3e-109 | 99.0 | 1 | 549 |
| AE017042\_YP\_3468 | 3942261 | 3944609 | True | COG4253 | 3e-66 | 82.0 | 2 | 229 |
| AE017042\_YP\_3469 | 3944624 | 3946846 | True | - | - | - | - | - |
| AE017042\_YP\_3470 | 3946847 | 3947347 | True | - | - | - | - | - |
| AE017042\_YP\_3471 | 3947531 | 3947725 | True | - | - | - | - | - |
| AE017042\_YP\_3472 | 3947751 | 3948029 | True | COG3677 | 3e-21 | 71.0 | 26 | 117 |
| AE017042\_YP\_3474 | 3948950 | 3949585 | True | COG4253 | 3e-62 | 81.0 | 4 | 229 |
| AE017042\_YP\_3475 | 3949601 | 3951898 | True | COG3179 | 6e-09 | 98.0 | 4 | 206 |
