# Supplementary material for: Dissecting the bacterial type VI secretion system by a genome wide in silico analysis: what can be learned from available microbial genomic resources?
Source: BMC Genomics. 2009 Mar 12;10:104. doi: 10.1186/1471-2164-10-104 (PMC2660368; doi:10.1186/1471-2164-10-104)
Supplement: Additional file 7 — Detailed description of all identified T6SS gene clusters. Archive containing the detailed description of each identified T6SS locus as an HTML file. [file 1471-2164-10-104-S7.tgz › LociHTML/HTML/AE017042H.html]

Locus AE017042H on Yersinia pestis (biovar Mediaevalis, strain 91001) chromosome, complete sequence.

import namespace="svg" implementation="#AdobeSVG"?


# Locus AE017042H

# List of CDS in T6SS locus AE017042H

|  |  |  |  |  |  |  |  |  |
| --- | --- | --- | --- | --- | --- | --- | --- | --- |
| Name | from | to | direct | COG | e-value | COG cover | COG hit start | COG hit end |
| AE017042\_YP\_3661 | 4163085 | 4165454 | True | COG0417 | 0.0 | 98.0 | 5 | 788 |
| AE017042\_YP\_3662 | 4165915 | 4168821 | True | COG0553 | 2e-72 | 98.0 | 8 | 861 |
| AE017042\_YP\_3663 | 4169077 | 4169436 | False | - | - | - | - | - |
| AE017042\_YP\_3664 | 4169452 | 4172946 | False | COG3523 | 0.0 | 98.0 | 12 | 1187 |
| AE017042\_YP\_3665 | 4172955 | 4174592 | False | COG3455 | 2e-72 | 100.0 | 1 | 262 |
| AE017042\_YP\_3665 | 4172955 | 4174592 | False | COG1360 | 2e-27 | 56.0 | 108 | 244 |
| AE017042\_YP\_3666 | 4174562 | 4175935 | False | COG3522 | 2e-141 | 100.0 | 1 | 446 |
| AE017042\_YP\_3667 | 4176019 | 4176510 | False | COG3521 | 1e-34 | 98.0 | 3 | 158 |
| AE017042\_YP\_3668 | 4176503 | 4176868 | False | - | - | - | - | - |
| AE017042\_YP\_3669 | 4176874 | 4177491 | False | - | - | - | - | - |
| AE017042\_YP\_3670 | 4177484 | 4178587 | False | COG1357 | 1e-18 | 99.0 | 3 | 238 |
| AE017042\_YP\_3671 | 4178613 | 4180832 | False | COG1357 | 4e-15 | 83.0 | 18 | 215 |
| AE017042\_YP\_3671 | 4178613 | 4180832 | False | COG5351 | 1e-07 | 50.0 | 68 | 253 |
| AE017042\_YP\_3672 | 4180845 | 4183193 | False | COG3501 | 5e-154 | 95.0 | 10 | 533 |
| AE017042\_YP\_3673 | 4183297 | 4185900 | False | COG0542 | 0.0 | 99.0 | 1 | 783 |
| AE017042\_YP\_3674 | 4185903 | 4186925 | False | COG3520 | 3e-84 | 99.0 | 3 | 334 |
| AE017042\_YP\_3675 | 4186879 | 4188723 | False | COG3519 | 0.0 | 99.0 | 1 | 617 |
| AE017042\_YP\_3676 | 4188756 | 4189199 | False | COG3518 | 8e-31 | 95.0 | 4 | 153 |
| AE017042\_YP\_3677 | 4189273 | 4189791 | False | COG3157 | 7e-36 | 100.0 | 1 | 162 |
| AE017042\_YP\_3678 | 4189954 | 4191465 | False | COG3517 | 0.0 | 100.0 | 1 | 495 |
| AE017042\_YP\_3679 | 4191465 | 4192025 | False | COG3516 | 1e-57 | 99.0 | 2 | 169 |
| AE017042\_YP\_3680 | 4192036 | 4193049 | False | COG3515 | 4e-55 | 100.0 | 1 | 346 |
| AE017042\_YP\_3681 | 4193379 | 4194122 | True | COG5419 | 2e-48 | 100.0 | 1 | 160 |
| AE017042\_YP\_3682 | 4195345 | 4195965 | True | COG0564 | 1e-59 | 72.0 | 80 | 289 |
| AE017042\_YP\_3683 | 4196263 | 4197096 | False | COG1076 | 6e-35 | 100.0 | 1 | 174 |
| AE017042\_YP\_3684 | 4197281 | 4199623 | True | COG1452 | 0.0 | 100.0 | 1 | 784 |
