# Supplementary material for: Dissecting the bacterial type VI secretion system by a genome wide in silico analysis: what can be learned from available microbial genomic resources?
Source: BMC Genomics. 2009 Mar 12;10:104. doi: 10.1186/1471-2164-10-104 (PMC2660368; doi:10.1186/1471-2164-10-104)
Supplement: Additional file 7 — Detailed description of all identified T6SS gene clusters. Archive containing the detailed description of each identified T6SS locus as an HTML file. [file 1471-2164-10-104-S7.tgz › LociHTML/HTML/AE017042I.html]

Locus AE017042I on Yersinia pestis (biovar Mediaevalis, strain 91001) chromosome, complete sequence.

import namespace="svg" implementation="#AdobeSVG"?


# Locus AE017042I

# List of CDS in T6SS locus AE017042I

|  |  |  |  |  |  |  |  |  |
| --- | --- | --- | --- | --- | --- | --- | --- | --- |
| Name | from | to | direct | COG | e-value | COG cover | COG hit start | COG hit end |
| AE017042\_YP\_3933 | 4472528 | 4477069 | False | COG3209 | 9e-64 | 99.0 | 1 | 795 |
| AE017042\_YP\_3933 | 4472528 | 4477069 | False | COG4104 | 3e-10 | 73.0 | 25 | 96 |
| AE017042\_YP\_3934 | 4477108 | 4477530 | False | COG5435 | 6e-45 | 97.0 | 3 | 145 |
| AE017042\_YP\_3935 | 4477533 | 4479731 | False | COG3501 | 0.0 | 99.0 | 1 | 549 |
| AE017042\_YP\_3936 | 4479753 | 4479929 | False | - | - | - | - | - |
| AE017042\_YP\_3937 | 4480011 | 4480499 | True | - | - | - | - | - |
| AE017042\_YP\_3938 | 4480581 | 4480805 | True | - | - | - | - | - |
| AE017042\_YP\_3939 | 4480881 | 4481072 | True | - | - | - | - | - |
| AE017042\_YP\_3940 | 4481122 | 4481607 | False | - | - | - | - | - |
| AE017042\_YP\_3941 | 4481609 | 4482979 | False | COG3209 | 8e-32 | 59.0 | 326 | 795 |
| AE017042\_YP\_3942 | 4483007 | 4485898 | False | COG3209 | 1e-61 | 99.0 | 2 | 794 |
| AE017042\_YP\_3943 | 4485864 | 4486322 | False | COG5435 | 5e-48 | 100.0 | 1 | 147 |
| AE017042\_YP\_3944 | 4486328 | 4488730 | False | COG3501 | 0.0 | 99.0 | 1 | 547 |
| AE017042\_YP\_3945 | 4488752 | 4489285 | False | COG3515 | 6e-19 | 50.0 | 13 | 188 |
| AE017042\_YP\_3946 | 4489282 | 4490064 | False | COG3515 | 3e-44 | 78.0 | 12 | 284 |
| AE017042\_YP\_3947 | 4490189 | 4493722 | False | COG3523 | 0.0 | 100.0 | 1 | 1188 |
| AE017042\_YP\_3948 | 4493754 | 4495160 | False | COG3515 | 7e-37 | 82.0 | 1 | 285 |
| AE017042\_YP\_3949 | 4495148 | 4495843 | False | - | - | - | - | - |
| AE017042\_YP\_3950 | 4495831 | 4496628 | False | - | - | - | - | - |
| AE017042\_YP\_3951 | 4496625 | 4499228 | False | COG0542 | 0.0 | 99.0 | 1 | 784 |
| AE017042\_YP\_3952 | 4499239 | 4500006 | False | COG3455 | 2e-86 | 98.0 | 4 | 260 |
| AE017042\_YP\_3953 | 4500006 | 4501352 | False | COG3522 | 1e-167 | 100.0 | 1 | 446 |
| AE017042\_YP\_3954 | 4501355 | 4501900 | False | COG3521 | 2e-39 | 100.0 | 1 | 159 |
| AE017042\_YP\_3955 | 4501900 | 4503216 | False | COG3456 | 9e-124 | 100.0 | 1 | 430 |
| AE017042\_YP\_3956 | 4503342 | 4504439 | False | COG3520 | 1e-105 | 99.0 | 1 | 332 |
| AE017042\_YP\_3958 | 4505167 | 4505703 | True | COG2963 | 5e-12 | 95.0 | 6 | 116 |
| AE017042\_YP\_3959 | 4505757 | 4506542 | True | COG2801 | 1e-17 | 92.0 | 16 | 230 |
| AE017042\_YP\_3960 | 4507015 | 4508244 | True | COG2814 | 7e-11 | 85.0 | 9 | 344 |
| AE017042\_YP\_3961 | 4508690 | 4508875 | True | - | - | - | - | - |
| AE017042\_YP\_3962 | 4508948 | 4509535 | True | - | - | - | - | - |
