# Supplementary material for: Dissecting the bacterial type VI secretion system by a genome wide in silico analysis: what can be learned from available microbial genomic resources?
Source: BMC Genomics. 2009 Mar 12;10:104. doi: 10.1186/1471-2164-10-104 (PMC2660368; doi:10.1186/1471-2164-10-104)
Supplement: Additional file 7 — Detailed description of all identified T6SS gene clusters. Archive containing the detailed description of each identified T6SS locus as an HTML file. [file 1471-2164-10-104-S7.tgz › LociHTML/HTML/AE017125A.html]

Locus AE017125A on Helicobacter hepaticus (strain 3B1 / ATCC 51449) chromosome, complete sequence.

import namespace="svg" implementation="#AdobeSVG"?


# Locus AE017125A

# List of CDS in T6SS locus AE017125A

|  |  |  |  |  |  |  |  |  |
| --- | --- | --- | --- | --- | --- | --- | --- | --- |
| Name | from | to | direct | COG | e-value | COG cover | COG hit start | COG hit end |
| AE017125\_HH\_0236 | 226607 | 227245 | False | - | - | - | - | - |
| AE017125\_HH\_0237 | 227242 | 228036 | False | - | - | - | - | - |
| AE017125\_HH\_0238 | 228126 | 228986 | False | COG0338 | 2e-29 | 98.0 | 4 | 272 |
| AE017125\_HH\_0239 | 228983 | 229768 | False | - | - | - | - | - |
| AE017125\_HH\_0240 | 229864 | 230250 | False | - | - | - | - | - |
| AE017125\_HH\_0241 | 230306 | 231583 | False | - | - | - | - | - |
| AE017125\_HH\_0242 | 231618 | 234416 | False | COG3501 | 2e-42 | 81.0 | 92 | 537 |
| AE017125\_HH\_0243 | 234543 | 235052 | False | COG3157 | 7e-40 | 95.0 | 1 | 155 |
| AE017125\_HH\_0244 | 235149 | 236144 | False | COG3520 | 1e-28 | 69.0 | 82 | 313 |
| AE017125\_HH\_0245 | 236141 | 237856 | False | COG3519 | 5e-72 | 95.0 | 8 | 600 |
| AE017125\_HH\_0246 | 237926 | 238315 | False | - | - | - | - | - |
| AE017125\_HH\_0247 | 238319 | 239791 | False | COG3517 | 9e-171 | 97.0 | 9 | 493 |
| AE017125\_HH\_0248 | 239839 | 240336 | False | COG3516 | 3e-38 | 93.0 | 12 | 169 |
| AE017125\_HH\_0249 | 240524 | 241060 | True | COG3521 | 2e-11 | 79.0 | 8 | 133 |
| AE017125\_HH\_0250 | 241053 | 242432 | True | COG3522 | 6e-65 | 99.0 | 1 | 444 |
| AE017125\_HH\_0251 | 242444 | 243232 | True | COG3455 | 5e-20 | 82.0 | 39 | 254 |
| AE017125\_HH\_0252 | 243237 | 246791 | True | COG3523 | 2e-83 | 98.0 | 9 | 1184 |
| AE017125\_HH\_0253 | 246801 | 247781 | True | - | - | - | - | - |
| AE017125\_HH\_0254 | 247872 | 249140 | False | - | - | - | - | - |
| AE017125\_HH\_0255 | 249168 | 253067 | False | - | - | - | - | - |
