# Supplementary material for: Dissecting the bacterial type VI secretion system by a genome wide in silico analysis: what can be learned from available microbial genomic resources?
Source: BMC Genomics. 2009 Mar 12;10:104. doi: 10.1186/1471-2164-10-104 (PMC2660368; doi:10.1186/1471-2164-10-104)
Supplement: Additional file 7 — Detailed description of all identified T6SS gene clusters. Archive containing the detailed description of each identified T6SS locus as an HTML file. [file 1471-2164-10-104-S7.tgz › LociHTML/HTML/AE017180A.html]

Locus AE017180A on Geobacter sulfurreducens (strain ATCC 51573 / DSM 12127 / PCA) chromosome, complete sequence.

import namespace="svg" implementation="#AdobeSVG"?


# Locus AE017180A

# List of CDS in T6SS locus AE017180A

|  |  |  |  |  |  |  |  |  |
| --- | --- | --- | --- | --- | --- | --- | --- | --- |
| Name | from | to | direct | COG | e-value | COG cover | COG hit start | COG hit end |
| AE017180\_GSU0421 | 451662 | 452648 | True | COG1868 | 1e-61 | 97.0 | 1 | 324 |
| AE017180\_GSU0422 | 452645 | 452950 | True | COG1886 | 4e-18 | 61.0 | 53 | 136 |
| AE017180\_GSU0423 | 453450 | 454217 | True | COG1338 | 2e-74 | 99.0 | 2 | 247 |
| AE017180\_GSU0424 | 454230 | 454499 | True | COG1987 | 2e-17 | 100.0 | 1 | 89 |
| AE017180\_GSU0425 | 454550 | 455344 | True | COG1684 | 1e-32 | 95.0 | 12 | 258 |
| AE017180\_GSU0426 | 455349 | 456407 | True | COG1377 | 6e-98 | 96.0 | 2 | 351 |
| AE017180\_GSU0427 | 456466 | 456936 | False | - | - | - | - | - |
| AE017180\_GSU0428 | 457223 | 457777 | True | COG3521 | 2e-07 | 95.0 | 3 | 154 |
| AE017180\_GSU0429 | 457774 | 459165 | True | COG3522 | 1e-50 | 97.0 | 9 | 444 |
| AE017180\_GSU0430 | 459169 | 459576 | True | COG3518 | 1e-15 | 91.0 | 13 | 155 |
| AE017180\_GSU0431 | 459605 | 461338 | True | COG3519 | 1e-113 | 99.0 | 7 | 621 |
| AE017180\_GSU0432 | 461302 | 462294 | True | COG3520 | 1e-53 | 93.0 | 8 | 321 |
| AE017180\_GSU0433 | 462307 | 464934 | True | COG0542 | 0.0 | 98.0 | 1 | 774 |
| AE017180\_GSU0434 | 464944 | 465948 | False | COG0482 | 1e-29 | 93.0 | 2 | 335 |
| AE017180\_GSU0435 | 466122 | 467771 | True | COG2804 | 3e-39 | 98.0 | 2 | 493 |
| AE017180\_GSU0436 | 467785 | 468855 | True | COG2805 | 4e-135 | 98.0 | 5 | 352 |
| AE017180\_GSU0437 | 469239 | 470654 | True | COG0043 | 3e-115 | 99.0 | 1 | 475 |
