# Supplementary material for: Dissecting the bacterial type VI secretion system by a genome wide in silico analysis: what can be learned from available microbial genomic resources?
Source: BMC Genomics. 2009 Mar 12;10:104. doi: 10.1186/1471-2164-10-104 (PMC2660368; doi:10.1186/1471-2164-10-104)
Supplement: Additional file 7 — Detailed description of all identified T6SS gene clusters. Archive containing the detailed description of each identified T6SS locus as an HTML file. [file 1471-2164-10-104-S7.tgz › LociHTML/HTML/AE017180C.html]

Locus AE017180C on Geobacter sulfurreducens (strain ATCC 51573 / DSM 12127 / PCA) chromosome, complete sequence.

import namespace="svg" implementation="#AdobeSVG"?


# Locus AE017180C

# List of CDS in T6SS locus AE017180C

|  |  |  |  |  |  |  |  |  |
| --- | --- | --- | --- | --- | --- | --- | --- | --- |
| Name | from | to | direct | COG | e-value | COG cover | COG hit start | COG hit end |
| AE017180\_GSU3157 | 3464329 | 3465129 | True | COG0596 | 5e-26 | 99.0 | 3 | 282 |
| AE017180\_GSU3158 | 3465190 | 3466116 | False | COG0031 | 3e-103 | 99.0 | 4 | 300 |
| AE017180\_GSU3159 | 3466266 | 3466820 | True | COG1051 | 3e-07 | 75.0 | 11 | 119 |
| AE017180\_GSU3160 | 3466826 | 3467578 | True | COG0564 | 2e-49 | 73.0 | 78 | 288 |
| AE017180\_GSU3161 | 3467663 | 3468151 | True | COG0225 | 2e-61 | 89.0 | 4 | 159 |
| AE017180\_GSU3162 | 3468247 | 3469293 | True | COG2008 | 3e-100 | 100.0 | 1 | 342 |
| AE017180\_GSU3163 | 3469290 | 3469436 | True | - | - | - | - | - |
| AE017180\_GSU3164 | 3469452 | 3469658 | True | - | - | - | - | - |
| AE017180\_GSU3165 | 3469894 | 3470688 | True | COG3455 | 1e-17 | 72.0 | 66 | 256 |
| AE017180\_GSU3166 | 3470702 | 3474154 | True | COG3523 | 3e-63 | 47.0 | 15 | 581 |
| AE017180\_GSU3167 | 3474201 | 3476570 | True | COG3515 | 7e-13 | 76.0 | 9 | 271 |
| AE017180\_GSU3168 | 3476577 | 3477770 | True | COG0304 | 8e-13 | 86.0 | 4 | 361 |
| AE017180\_GSU3169 | 3477770 | 3478438 | True | - | - | - | - | - |
| AE017180\_GSU3170 | 3478435 | 3479463 | True | - | - | - | - | - |
| AE017180\_GSU3171 | 3479479 | 3481098 | True | - | - | - | - | - |
| AE017180\_GSU3172 | 3481200 | 3481685 | True | COG3516 | 3e-35 | 92.0 | 7 | 163 |
| AE017180\_GSU3173 | 3481696 | 3483180 | True | COG3517 | 0.0 | 99.0 | 1 | 493 |
| AE017180\_GSU3174 | 3483262 | 3483747 | True | COG3157 | 2e-33 | 97.0 | 1 | 158 |
| AE017180\_GSU3175 | 3483866 | 3484321 | True | - | - | - | - | - |
| AE017180\_GSU3176 | 3484376 | 3485293 | True | - | - | - | - | - |
| AE017180\_GSU3177 | 3485350 | 3487443 | True | COG3501 | 1e-136 | 99.0 | 1 | 549 |
| AE017180\_GSU3178 | 3487475 | 3487750 | True | - | - | - | - | - |
| AE017180\_GSU3179 | 3487734 | 3487979 | True | - | - | - | - | - |
| AE017180\_GSU3180 | 3488000 | 3489253 | True | - | - | - | - | - |
| AE017180\_GSU3181 | 3489250 | 3490293 | True | - | - | - | - | - |
| AE017180\_GSU3182 | 3490313 | 3490693 | True | - | - | - | - | - |
| AE017180\_GSU3183 | 3490745 | 3491122 | True | - | - | - | - | - |
| AE017180\_GSU3184 | 3491320 | 3493002 | True | - | - | - | - | - |
