# Supplementary material for: Dissecting the bacterial type VI secretion system by a genome wide in silico analysis: what can be learned from available microbial genomic resources?
Source: BMC Genomics. 2009 Mar 12;10:104. doi: 10.1186/1471-2164-10-104 (PMC2660368; doi:10.1186/1471-2164-10-104)
Supplement: Additional file 7 — Detailed description of all identified T6SS gene clusters. Archive containing the detailed description of each identified T6SS locus as an HTML file. [file 1471-2164-10-104-S7.tgz › LociHTML/HTML/AE017220B.html]

Locus AE017220B on Salmonella choleraesuis (strain SC-B67) chromosome, complete sequence.

import namespace="svg" implementation="#AdobeSVG"?


# Locus AE017220B

# List of CDS in T6SS locus AE017220B

|  |  |  |  |  |  |  |  |  |
| --- | --- | --- | --- | --- | --- | --- | --- | --- |
| Name | from | to | direct | COG | e-value | COG cover | COG hit start | COG hit end |
| AE017220\_SCH\_0255 | 295006 | 295776 | True | COG2226 | 8e-23 | 61.0 | 18 | 164 |
| AE017220\_SCH\_0256 | 295832 | 297199 | False | COG1388 | 1e-07 | 95.0 | 1 | 119 |
| AE017220\_SCH\_0256 | 295832 | 297199 | False | COG1388 | 1e-08 | 93.0 | 1 | 116 |
| AE017220\_SCH\_0256 | 295832 | 297199 | False | COG0741 | 4e-13 | 95.0 | 1 | 284 |
| AE017220\_SCH\_0257 | 297271 | 298026 | False | COG0491 | 2e-25 | 88.0 | 19 | 241 |
| AE017220\_SCH\_0258 | 298061 | 298783 | True | COG2226 | 2e-07 | 42.0 | 81 | 181 |
| AE017220\_SCH\_0259 | 298780 | 299604 | False | COG0328 | 2e-57 | 99.0 | 2 | 154 |
| AE017220\_SCH\_0260 | 299302 | 300042 | True | COG0847 | 1e-51 | 96.0 | 6 | 240 |
| AE017220\_SCH\_0261 | 300574 | 301629 | False | COG3515 | 1e-43 | 97.0 | 3 | 340 |
| AE017220\_SCH\_0262 | 301640 | 302635 | False | COG3520 | 1e-95 | 97.0 | 11 | 335 |
| AE017220\_SCH\_0263 | 302632 | 304515 | False | COG3519 | 0.0 | 100.0 | 1 | 621 |
| AE017220\_SCH\_0264 | 304531 | 305025 | False | COG3518 | 7e-36 | 98.0 | 1 | 154 |
| AE017220\_SCH\_0265 | 305022 | 305771 | False | COG4455 | 6e-101 | 91.0 | 23 | 273 |
| AE017220\_SCH\_0266 | 305833 | 306735 | False | - | - | - | - | - |
| AE017220\_SCH\_0267 | 306744 | 306815 | False | - | - | - | - | - |
| AE017220\_SCH\_0268 | 309841 | 310383 | True | COG3516 | 1e-57 | 99.0 | 2 | 169 |
| AE017220\_SCH\_0269 | 310407 | 311915 | True | COG3517 | 0.0 | 100.0 | 1 | 495 |
| AE017220\_SCH\_0270 | 311982 | 312368 | True | - | - | - | - | - |
| AE017220\_SCH\_0271 | 312381 | 312827 | True | - | - | - | - | - |
| AE017220\_SCH\_0272 | 313079 | 313564 | True | COG3157 | 2e-43 | 100.0 | 1 | 162 |
| AE017220\_SCH\_0273 | 313631 | 314167 | True | COG3521 | 1e-42 | 97.0 | 3 | 157 |
| AE017220\_SCH\_0274 | 314171 | 315514 | True | COG3522 | 1e-153 | 100.0 | 1 | 446 |
| AE017220\_SCH\_0275 | 315511 | 316815 | True | COG3455 | 6e-72 | 99.0 | 1 | 261 |
| AE017220\_SCH\_0275 | 315511 | 316815 | True | COG1360 | 2e-31 | 58.0 | 101 | 242 |
| AE017220\_SCH\_0276 | 316820 | 317593 | True | - | - | - | - | - |
| AE017220\_SCH\_0277 | 317783 | 318226 | True | - | - | - | - | - |
| AE017220\_SCH\_0278 | 318260 | 322129 | True | COG3523 | 0.0 | 99.0 | 2 | 1188 |
| AE017220\_SCH\_0279 | 322129 | 322917 | True | - | - | - | - | - |
| AE017220\_SCH\_0280 | 322914 | 323330 | True | COG4893 | 3e-49 | 100.0 | 1 | 123 |
| AE017220\_SCH\_0281 | 323354 | 323875 | True | - | - | - | - | - |
| AE017220\_SCH\_0282 | 324271 | 326505 | True | COG3501 | 4e-161 | 97.0 | 1 | 538 |
| AE017220\_SCH\_0283 | 326517 | 327023 | True | - | - | - | - | - |
| AE017220\_SCH\_0284 | 327039 | 331820 | True | COG3209 | 2e-42 | 82.0 | 2 | 660 |
| AE017220\_SCH\_0284 | 327039 | 331820 | True | COG3209 | 5e-09 | 44.0 | 305 | 658 |
