# Supplementary material for: Dissecting the bacterial type VI secretion system by a genome wide in silico analysis: what can be learned from available microbial genomic resources?
Source: BMC Genomics. 2009 Mar 12;10:104. doi: 10.1186/1471-2164-10-104 (PMC2660368; doi:10.1186/1471-2164-10-104)
Supplement: Additional file 7 — Detailed description of all identified T6SS gene clusters. Archive containing the detailed description of each identified T6SS locus as an HTML file. [file 1471-2164-10-104-S7.tgz › LociHTML/HTML/AL513382A.html]

Locus AL513382A on Salmonella typhi (strain CT18) chromosome, complete sequence.

import namespace="svg" implementation="#AdobeSVG"?


# Locus AL513382A

# List of CDS in T6SS locus AL513382A

|  |  |  |  |  |  |  |  |  |
| --- | --- | --- | --- | --- | --- | --- | --- | --- |
| Name | from | to | direct | COG | e-value | COG cover | COG hit start | COG hit end |
| AL513382\_STY0280 | 296922 | 297692 | True | COG2226 | 8e-23 | 61.0 | 18 | 164 |
| AL513382\_STY0281 | 297748 | 299115 | False | COG1388 | 1e-07 | 95.0 | 1 | 119 |
| AL513382\_STY0281 | 297748 | 299115 | False | COG1388 | 9e-09 | 93.0 | 1 | 116 |
| AL513382\_STY0281 | 297748 | 299115 | False | COG0741 | 4e-13 | 95.0 | 1 | 284 |
| AL513382\_STY0282 | 299187 | 299942 | False | COG0491 | 2e-25 | 88.0 | 19 | 241 |
| AL513382\_STY0283 | 299977 | 300699 | True | COG2226 | 2e-07 | 36.0 | 81 | 168 |
| AL513382\_STY0284 | 300696 | 301163 | False | COG0328 | 1e-54 | 99.0 | 2 | 154 |
| AL513382\_STY0285 | 301227 | 301958 | True | COG0847 | 3e-51 | 95.0 | 8 | 240 |
| AL513382\_STY0286 | 302488 | 303120 | False | COG3515 | 3e-18 | 56.0 | 145 | 340 |
| AL513382\_STY0287 | 303120 | 303542 | False | COG3515 | 1e-19 | 39.0 | 3 | 138 |
| AL513382\_STY0288 | 303553 | 304548 | False | COG3520 | 7e-95 | 97.0 | 11 | 335 |
| AL513382\_STY0289 | 304545 | 306428 | False | COG3519 | 0.0 | 100.0 | 1 | 621 |
| AL513382\_STY0290 | 306444 | 306938 | False | COG3518 | 5e-35 | 98.0 | 1 | 154 |
| AL513382\_STY0291 | 306935 | 307759 | False | COG4455 | 2e-108 | 100.0 | 1 | 273 |
| AL513382\_STY0292 | 307746 | 308648 | False | - | - | - | - | - |
| AL513382\_STY0293 | 308657 | 308728 | False | - | - | - | - | - |
| AL513382\_STY0294 | 309016 | 311679 | True | COG0542 | 0.0 | 99.0 | 1 | 781 |
| AL513382\_STY0295 | 311664 | 311762 | True | - | - | - | - | - |
| AL513382\_STY0296 | 311765 | 312055 | True | - | - | - | - | - |
| AL513382\_STY0297 | 312119 | 312661 | True | COG3516 | 9e-58 | 99.0 | 2 | 169 |
| AL513382\_STY0300 | 314246 | 314647 | True | - | - | - | - | - |
| AL513382\_STY0301 | 314619 | 315044 | True | - | - | - | - | - |
| AL513382\_STY0302 | 315281 | 315766 | True | COG3157 | 3e-44 | 100.0 | 1 | 162 |
| AL513382\_STY0303 | 315833 | 316369 | True | COG3521 | 8e-43 | 97.0 | 3 | 157 |
| AL513382\_STY0304 | 316373 | 317716 | True | COG3522 | 3e-153 | 100.0 | 1 | 446 |
| AL513382\_STY0305 | 317713 | 319014 | True | COG3455 | 6e-72 | 99.0 | 1 | 261 |
| AL513382\_STY0305 | 317713 | 319014 | True | COG1360 | 6e-32 | 58.0 | 101 | 242 |
| AL513382\_STY0306 | 319019 | 319768 | True | - | - | - | - | - |
| AL513382\_STY0307 | 319800 | 320294 | True | - | - | - | - | - |
| AL513382\_STY0310 | 324392 | 325159 | True | - | - | - | - | - |
| AL513382\_STY0311 | 325202 | 325672 | True | COG1705 | 2e-07 | 67.0 | 45 | 179 |
| AL513382\_STY0312 | 325657 | 326130 | True | - | - | - | - | - |
| AL513382\_STY0313 | 326234 | 326704 | True | - | - | - | - | - |
| AL513382\_STY0314 | 326689 | 327150 | True | - | - | - | - | - |
| AL513382\_STY0316 | 327278 | 327694 | True | COG4893 | 3e-49 | 100.0 | 1 | 123 |
| AL513382\_STY0317 | 327721 | 328158 | True | - | - | - | - | - |
| AL513382\_STY0318 | 328166 | 328273 | True | - | - | - | - | - |
| AL513382\_STY0319 | 328636 | 330825 | True | COG3501 | 9e-163 | 100.0 | 1 | 550 |
| AL513382\_STY0320 | 330849 | 331295 | True | COG5435 | 1e-42 | 98.0 | 1 | 145 |
| AL513382\_STY0321 | 331314 | 335378 | True | COG3209 | 3e-69 | 83.0 | 1 | 661 |
| AL513382\_STY0322 | 335381 | 336058 | True | - | - | - | - | - |
