# Supplementary material for: Dissecting the bacterial type VI secretion system by a genome wide in silico analysis: what can be learned from available microbial genomic resources?
Source: BMC Genomics. 2009 Mar 12;10:104. doi: 10.1186/1471-2164-10-104 (PMC2660368; doi:10.1186/1471-2164-10-104)
Supplement: Additional file 7 — Detailed description of all identified T6SS gene clusters. Archive containing the detailed description of each identified T6SS locus as an HTML file. [file 1471-2164-10-104-S7.tgz › LociHTML/HTML/AL590842A.html]

Locus AL590842A on Yersinia pestis (biovar Orientalis, strain CO-92) chromosome, complete sequence.

import namespace="svg" implementation="#AdobeSVG"?


# Locus AL590842A

# List of CDS in T6SS locus AL590842A

|  |  |  |  |  |  |  |  |  |
| --- | --- | --- | --- | --- | --- | --- | --- | --- |
| Name | from | to | direct | COG | e-value | COG cover | COG hit start | COG hit end |
| AL590842\_YPO0495 | 526127 | 528469 | False | COG1452 | 0.0 | 100.0 | 1 | 784 |
| AL590842\_YPO0496 | 528654 | 529487 | True | COG1076 | 6e-35 | 100.0 | 1 | 174 |
| AL590842\_YPO0497 | 529785 | 530405 | False | COG0564 | 1e-59 | 72.0 | 80 | 289 |
| AL590842\_YPO0498 | 531628 | 532371 | False | COG5419 | 2e-48 | 100.0 | 1 | 160 |
| AL590842\_YPO0499 | 532701 | 533714 | True | COG3515 | 4e-55 | 100.0 | 1 | 346 |
| AL590842\_YPO0500 | 533725 | 534285 | True | COG3516 | 1e-57 | 99.0 | 2 | 169 |
| AL590842\_YPO0501 | 534285 | 535796 | True | COG3517 | 0.0 | 100.0 | 1 | 495 |
| AL590842\_YPO0502 | 535959 | 536477 | True | COG3157 | 7e-36 | 100.0 | 1 | 162 |
| AL590842\_YPO0503 | 536551 | 536994 | True | COG3518 | 8e-31 | 95.0 | 4 | 153 |
| AL590842\_YPO0504 | 537027 | 538871 | True | COG3519 | 0.0 | 99.0 | 1 | 617 |
| AL590842\_YPO0505 | 538864 | 539847 | True | COG3520 | 2e-83 | 98.0 | 4 | 334 |
| AL590842\_YPO0506 | 539850 | 542453 | True | COG0542 | 0.0 | 99.0 | 1 | 783 |
| AL590842\_YPO0507 | 542557 | 544905 | True | COG3501 | 5e-154 | 95.0 | 10 | 533 |
| AL590842\_YPO0508 | 544918 | 545490 | True | - | - | - | - | - |
| AL590842\_YPO0509 | 545515 | 547137 | True | COG1357 | 8e-14 | 83.0 | 18 | 215 |
| AL590842\_YPO0510 | 547163 | 548266 | True | COG1357 | 1e-18 | 99.0 | 3 | 238 |
| AL590842\_YPO0511 | 548259 | 548876 | True | - | - | - | - | - |
| AL590842\_YPO0511.1 | 548882 | 549247 | True | - | - | - | - | - |
| AL590842\_YPO0512 | 549240 | 549731 | True | COG3521 | 1e-34 | 98.0 | 3 | 158 |
| AL590842\_YPO0513 | 549851 | 551206 | True | COG3522 | 2e-141 | 100.0 | 1 | 446 |
| AL590842\_YPO0514 | 551203 | 552813 | True | COG3455 | 1e-72 | 100.0 | 1 | 262 |
| AL590842\_YPO0514 | 551203 | 552813 | True | COG1360 | 2e-27 | 56.0 | 108 | 244 |
| AL590842\_YPO0515 | 552863 | 556315 | True | COG3523 | 0.0 | 98.0 | 18 | 1187 |
| AL590842\_YPO0516 | 556337 | 556690 | True | - | - | - | - | - |
| AL590842\_YPO0517 | 556946 | 559852 | False | COG0553 | 2e-72 | 98.0 | 8 | 861 |
| AL590842\_YPO0518 | 560313 | 562682 | False | COG0417 | 0.0 | 98.0 | 5 | 788 |
