# Supplementary material for: Dissecting the bacterial type VI secretion system by a genome wide in silico analysis: what can be learned from available microbial genomic resources?
Source: BMC Genomics. 2009 Mar 12;10:104. doi: 10.1186/1471-2164-10-104 (PMC2660368; doi:10.1186/1471-2164-10-104)
Supplement: Additional file 7 — Detailed description of all identified T6SS gene clusters. Archive containing the detailed description of each identified T6SS locus as an HTML file. [file 1471-2164-10-104-S7.tgz › LociHTML/HTML/AL590842B.html]

Locus AL590842B on Yersinia pestis (biovar Orientalis, strain CO-92) chromosome, complete sequence.

import namespace="svg" implementation="#AdobeSVG"?


# Locus AL590842B

# List of CDS in T6SS locus AL590842B

|  |  |  |  |  |  |  |  |  |
| --- | --- | --- | --- | --- | --- | --- | --- | --- |
| Name | from | to | direct | COG | e-value | COG cover | COG hit start | COG hit end |
| AL590842\_YPO0966 | 1067376 | 1069673 | False | COG3179 | 6e-09 | 98.0 | 4 | 206 |
| AL590842\_YPO0967 | 1069689 | 1070324 | False | COG4253 | 3e-62 | 81.0 | 4 | 229 |
| AL590842\_YPO0967.1 | 1070859 | 1071191 | False | COG1662 | 8e-26 | 95.0 | 6 | 121 |
| AL590842\_YPO0968 | 1071245 | 1071520 | False | COG3677 | 3e-21 | 71.0 | 26 | 117 |
| AL590842\_YPO0968.1 | 1071907 | 1072287 | False | - | - | - | - | - |
| AL590842\_YPO0969 | 1072398 | 1074620 | False | - | - | - | - | - |
| AL590842\_YPO0970 | 1074635 | 1076983 | False | COG3501 | 9e-110 | 99.0 | 1 | 549 |
| AL590842\_YPO0970 | 1074635 | 1076983 | False | COG4253 | 6e-66 | 82.0 | 2 | 229 |
| AL590842\_YPO0971 | 1076980 | 1079628 | False | COG0542 | 0.0 | 100.0 | 1 | 786 |
| AL590842\_YPO0973 | 1080046 | 1080537 | False | COG3157 | 2e-40 | 98.0 | 1 | 160 |
| AL590842\_YPO0974 | 1080541 | 1082277 | False | COG2885 | 8e-27 | 94.0 | 12 | 190 |
| AL590842\_YPO0975 | 1082277 | 1082963 | False | COG3455 | 2e-48 | 91.0 | 21 | 260 |
| AL590842\_YPO0976 | 1082960 | 1084312 | False | COG3522 | 6e-133 | 99.0 | 2 | 446 |
| AL590842\_YPO0977 | 1084324 | 1085868 | False | COG3517 | 0.0 | 100.0 | 1 | 495 |
| AL590842\_YPO0978 | 1085911 | 1086411 | False | COG3516 | 6e-49 | 99.0 | 2 | 169 |
| AL590842\_YPO0980 | 1087811 | 1088155 | False | - | - | - | - | - |
| AL590842\_YPO0980.1 | 1088261 | 1088320 | False | - | - | - | - | - |
| AL590842\_YPO0981 | 1088356 | 1088619 | False | COG3677 | 2e-18 | 68.0 | 26 | 114 |
| AL590842\_YPO0982 | 1088687 | 1089082 | False | - | - | - | - | - |
| AL590842\_YPO0983 | 1089533 | 1090183 | False | - | - | - | - | - |
| AL590842\_YPO0984 | 1091363 | 1092013 | True | COG3916 | 3e-64 | 100.0 | 1 | 209 |
