# Supplementary material for: Dissecting the bacterial type VI secretion system by a genome wide in silico analysis: what can be learned from available microbial genomic resources?
Source: BMC Genomics. 2009 Mar 12;10:104. doi: 10.1186/1471-2164-10-104 (PMC2660368; doi:10.1186/1471-2164-10-104)
Supplement: Additional file 7 — Detailed description of all identified T6SS gene clusters. Archive containing the detailed description of each identified T6SS locus as an HTML file. [file 1471-2164-10-104-S7.tgz › LociHTML/HTML/AL590842C.html]

Locus AL590842C on Yersinia pestis (biovar Orientalis, strain CO-92) chromosome, complete sequence.

import namespace="svg" implementation="#AdobeSVG"?


# Locus AL590842C

# List of CDS in T6SS locus AL590842C

|  |  |  |  |  |  |  |  |  |
| --- | --- | --- | --- | --- | --- | --- | --- | --- |
| Name | from | to | direct | COG | e-value | COG cover | COG hit start | COG hit end |
| AL590842\_YPO1458 | 1651381 | 1652100 | True | COG1024 | 6e-29 | 87.0 | 29 | 252 |
| AL590842\_YPO1459 | 1652093 | 1652854 | True | COG1024 | 3e-37 | 93.0 | 1 | 240 |
| AL590842\_YPO1460 | 1652857 | 1653645 | True | COG1028 | 4e-25 | 99.0 | 2 | 250 |
| AL590842\_YPO1461 | 1653684 | 1654172 | True | - | - | - | - | - |
| AL590842\_YPO1462 | 1654210 | 1654458 | True | - | - | - | - | - |
| AL590842\_YPO1463 | 1654557 | 1655405 | True | COG0331 | 2e-74 | 93.0 | 2 | 290 |
| AL590842\_YPO1464 | 1655562 | 1655729 | True | - | - | - | - | - |
| AL590842\_YPO1465 | 1656394 | 1656894 | True | COG3516 | 2e-48 | 99.0 | 2 | 169 |
| AL590842\_YPO1466 | 1656937 | 1658487 | True | COG3517 | 0.0 | 100.0 | 1 | 495 |
| AL590842\_YPO1467 | 1658499 | 1659851 | True | COG3522 | 4e-132 | 99.0 | 2 | 446 |
| AL590842\_YPO1468 | 1659848 | 1660534 | True | COG3455 | 2e-48 | 91.0 | 21 | 260 |
| AL590842\_YPO1469 | 1660534 | 1662270 | True | COG2885 | 5e-27 | 94.0 | 12 | 190 |
| AL590842\_YPO1470 | 1662274 | 1662765 | True | COG3157 | 2e-40 | 98.0 | 1 | 160 |
| AL590842\_YPO1471 | 1663153 | 1665795 | True | COG0542 | 0.0 | 99.0 | 1 | 784 |
| AL590842\_YPO1472 | 1665798 | 1668146 | True | COG3501 | 1e-105 | 99.0 | 1 | 549 |
| AL590842\_YPO1472 | 1665798 | 1668146 | True | COG4253 | 6e-67 | 82.0 | 2 | 229 |
| AL590842\_YPO1473 | 1668162 | 1670462 | True | - | - | - | - | - |
| AL590842\_YPO1474 | 1670459 | 1671232 | True | - | - | - | - | - |
| AL590842\_YPO1475 | 1671386 | 1671646 | True | COG4253 | 2e-25 | 30.0 | 144 | 229 |
| AL590842\_YPO1476 | 1671662 | 1673845 | True | - | - | - | - | - |
| AL590842\_YPO1477 | 1674018 | 1674488 | True | - | - | - | - | - |
| AL590842\_YPO1481 | 1676764 | 1677885 | True | - | - | - | - | - |
| AL590842\_YPO1482 | 1677882 | 1681304 | True | COG3523 | 0.0 | 100.0 | 1 | 1188 |
| AL590842\_YPO1483 | 1681348 | 1682949 | True | COG3515 | 8e-42 | 100.0 | 1 | 346 |
| AL590842\_YPO1484 | 1682940 | 1684025 | True | - | - | - | - | - |
| AL590842\_YPO1484.1 | 1684025 | 1684495 | True | - | - | - | - | - |
| AL590842\_YPO1485 | 1684716 | 1686479 | True | COG3519 | 0.0 | 100.0 | 1 | 621 |
| AL590842\_YPO1486 | 1686443 | 1687528 | True | COG3520 | 3e-85 | 97.0 | 1 | 328 |
| AL590842\_YPO1487 | 1687596 | 1688084 | True | COG3521 | 9e-36 | 98.0 | 1 | 157 |
| AL590842\_YPO1488 | 1688084 | 1688536 | True | COG3518 | 1e-27 | 98.0 | 3 | 157 |
| AL590842\_YPO1489 | 1688561 | 1689928 | True | COG3515 | 9e-41 | 96.0 | 13 | 346 |
| AL590842\_YPO1490 | 1690140 | 1690763 | False | - | - | - | - | - |
| AL590842\_YPO1491 | 1691446 | 1693041 | True | COG0488 | 2e-170 | 99.0 | 3 | 528 |
| AL590842\_YPO1492 | 1693269 | 1693922 | True | - | - | - | - | - |
| AL590842\_YPO1493 | 1693966 | 1695174 | False | COG3328 | 2e-112 | 98.0 | 1 | 375 |
