# Supplementary material for: Dissecting the bacterial type VI secretion system by a genome wide in silico analysis: what can be learned from available microbial genomic resources?
Source: BMC Genomics. 2009 Mar 12;10:104. doi: 10.1186/1471-2164-10-104 (PMC2660368; doi:10.1186/1471-2164-10-104)
Supplement: Additional file 7 — Detailed description of all identified T6SS gene clusters. Archive containing the detailed description of each identified T6SS locus as an HTML file. [file 1471-2164-10-104-S7.tgz › LociHTML/HTML/AL590842E.html]

Locus AL590842E on Yersinia pestis (biovar Orientalis, strain CO-92) chromosome, complete sequence.

import namespace="svg" implementation="#AdobeSVG"?


# Locus AL590842E

# List of CDS in T6SS locus AL590842E

|  |  |  |  |  |  |  |  |  |
| --- | --- | --- | --- | --- | --- | --- | --- | --- |
| Name | from | to | direct | COG | e-value | COG cover | COG hit start | COG hit end |
| AL590842\_YPO2715 | 3042058 | 3042912 | False | - | - | - | - | - |
| AL590842\_YPO2716 | 3043412 | 3045211 | True | COG0481 | 0.0 | 99.0 | 6 | 603 |
| AL590842\_YPO2717 | 3045221 | 3046219 | True | COG0681 | 6e-15 | 57.0 | 8 | 103 |
| AL590842\_YPO2718 | 3046571 | 3047251 | True | COG0571 | 1e-77 | 97.0 | 3 | 230 |
| AL590842\_YPO2719 | 3047248 | 3048159 | True | COG1159 | 4e-122 | 99.0 | 2 | 298 |
| AL590842\_YPO2720 | 3048169 | 3048894 | True | COG1381 | 7e-56 | 96.0 | 8 | 250 |
| AL590842\_YPO2721 | 3049021 | 3050043 | True | COG4584 | 2e-58 | 100.0 | 1 | 278 |
| AL590842\_YPO2722 | 3050040 | 3050822 | True | COG1484 | 2e-64 | 100.0 | 1 | 254 |
| AL590842\_YPO2723 | 3050889 | 3051674 | True | COG1360 | 1e-31 | 59.0 | 95 | 240 |
| AL590842\_YPO2723 | 3050889 | 3051674 | True | COG3455 | 3e-30 | 42.0 | 151 | 262 |
| AL590842\_YPO2724 | 3051683 | 3055510 | True | COG3523 | 0.0 | 99.0 | 3 | 1185 |
| AL590842\_YPO2725 | 3055804 | 3058569 | True | COG3501 | 7e-149 | 97.0 | 6 | 539 |
| AL590842\_YPO2726 | 3058928 | 3059776 | True | - | - | - | - | - |
| AL590842\_YPO2727 | 3059796 | 3060602 | True | COG4455 | 9e-108 | 100.0 | 1 | 273 |
| AL590842\_YPO2728 | 3060699 | 3061178 | True | COG3518 | 4e-35 | 99.0 | 1 | 156 |
| AL590842\_YPO2729 | 3061203 | 3061550 | True | - | - | - | - | - |
| AL590842\_YPO2730 | 3061607 | 3061948 | False | COG2824 | 2e-46 | 100.0 | 1 | 112 |
| AL590842\_YPO2731 | 3062324 | 3064363 | False | COG4907 | 4e-08 | 30.0 | 412 | 594 |
| AL590842\_YPO2732 | 3064368 | 3064910 | False | COG1704 | 3e-55 | 99.0 | 2 | 185 |
| AL590842\_YPO2733 | 3065526 | 3065993 | True | - | - | - | - | - |
