# Supplementary material for: Dissecting the bacterial type VI secretion system by a genome wide in silico analysis: what can be learned from available microbial genomic resources?
Source: BMC Genomics. 2009 Mar 12;10:104. doi: 10.1186/1471-2164-10-104 (PMC2660368; doi:10.1186/1471-2164-10-104)
Supplement: Additional file 7 — Detailed description of all identified T6SS gene clusters. Archive containing the detailed description of each identified T6SS locus as an HTML file. [file 1471-2164-10-104-S7.tgz › LociHTML/HTML/AL590842F.html]

Locus AL590842F on Yersinia pestis (biovar Orientalis, strain CO-92) chromosome, complete sequence.

import namespace="svg" implementation="#AdobeSVG"?


# Locus AL590842F

# List of CDS in T6SS locus AL590842F

|  |  |  |  |  |  |  |  |  |
| --- | --- | --- | --- | --- | --- | --- | --- | --- |
| Name | from | to | direct | COG | e-value | COG cover | COG hit start | COG hit end |
| AL590842\_YPO2927 | 3272776 | 3273036 | True | - | - | - | - | - |
| AL590842\_YPO2928 | 3273192 | 3273650 | False | COG1943 | 4e-33 | 97.0 | 1 | 133 |
| AL590842\_YPO2929 | 3273852 | 3274232 | False | COG0736 | 7e-40 | 98.0 | 1 | 125 |
| AL590842\_YPO2930 | 3274232 | 3274963 | False | COG0854 | 2e-99 | 100.0 | 1 | 243 |
| AL590842\_YPO2931 | 3275153 | 3275935 | False | COG1484 | 2e-64 | 100.0 | 1 | 254 |
| AL590842\_YPO2932 | 3275932 | 3276954 | False | COG4584 | 2e-58 | 100.0 | 1 | 278 |
| AL590842\_YPO2934 | 3277633 | 3278982 | False | COG3522 | 3e-158 | 99.0 | 2 | 446 |
| AL590842\_YPO2935 | 3278986 | 3279546 | False | COG3521 | 4e-39 | 100.0 | 1 | 159 |
| AL590842\_YPO2937 | 3279799 | 3280284 | False | COG3157 | 7e-42 | 100.0 | 1 | 162 |
| AL590842\_YPO2938 | 3280610 | 3282112 | False | COG3517 | 0.0 | 100.0 | 1 | 495 |
| AL590842\_YPO2939 | 3282136 | 3282660 | False | COG3516 | 8e-59 | 99.0 | 2 | 169 |
| AL590842\_YPO2940 | 3282765 | 3283319 | False | COG3539 | 5e-16 | 99.0 | 2 | 184 |
| AL590842\_YPO2942 | 3284573 | 3285781 | True | COG3328 | 2e-112 | 98.0 | 1 | 375 |
| AL590842\_YPO2944 | 3287481 | 3288260 | False | COG3121 | 6e-59 | 99.0 | 1 | 234 |
| AL590842\_YPO2945 | 3288399 | 3288944 | False | COG3539 | 1e-14 | 100.0 | 1 | 184 |
| AL590842\_YPO2946 | 3289145 | 3291820 | False | COG0542 | 0.0 | 98.0 | 1 | 777 |
| AL590842\_YPO2947 | 3292603 | 3294483 | True | COG3519 | 0.0 | 100.0 | 1 | 621 |
| AL590842\_YPO2948 | 3294483 | 3295508 | True | COG3520 | 1e-97 | 100.0 | 1 | 335 |
| AL590842\_YPO2949 | 3295607 | 3296788 | True | COG3515 | 1e-49 | 98.0 | 1 | 341 |
| AL590842\_YPO2950 | 3296795 | 3297826 | True | - | - | - | - | - |
| AL590842\_YPO2951 | 3297866 | 3299203 | True | - | - | - | - | - |
| AL590842\_YPO2952 | 3299200 | 3299883 | True | - | - | - | - | - |
| AL590842\_YPO2953 | 3299884 | 3301566 | True | COG2885 | 1e-27 | 55.0 | 86 | 190 |
| AL590842\_YPO2954 | 3301563 | 3302045 | True | COG5435 | 7e-43 | 100.0 | 1 | 147 |
