# Supplementary material for: Dissecting the bacterial type VI secretion system by a genome wide in silico analysis: what can be learned from available microbial genomic resources?
Source: BMC Genomics. 2009 Mar 12;10:104. doi: 10.1186/1471-2164-10-104 (PMC2660368; doi:10.1186/1471-2164-10-104)
Supplement: Additional file 7 — Detailed description of all identified T6SS gene clusters. Archive containing the detailed description of each identified T6SS locus as an HTML file. [file 1471-2164-10-104-S7.tgz › LociHTML/HTML/AL590842G.html]

Locus AL590842G on Yersinia pestis (biovar Orientalis, strain CO-92) chromosome, complete sequence.

import namespace="svg" implementation="#AdobeSVG"?


# Locus AL590842G

# List of CDS in T6SS locus AL590842G

|  |  |  |  |  |  |  |  |  |
| --- | --- | --- | --- | --- | --- | --- | --- | --- |
| Name | from | to | direct | COG | e-value | COG cover | COG hit start | COG hit end |
| AL590842\_YPO3588 | 3996574 | 3997509 | True | COG0540 | 5e-115 | 100.0 | 1 | 316 |
| AL590842\_YPO3589 | 3997521 | 3997985 | True | COG1781 | 9e-57 | 100.0 | 1 | 153 |
| AL590842\_YPO3590 | 3998123 | 3998509 | True | COG0251 | 7e-33 | 97.0 | 3 | 129 |
| AL590842\_YPO3591 | 3998850 | 3999872 | True | COG4584 | 2e-58 | 100.0 | 1 | 278 |
| AL590842\_YPO3592 | 3999869 | 4000651 | True | COG1484 | 2e-64 | 100.0 | 1 | 254 |
| AL590842\_YPO3592.1 | 4000721 | 4000924 | False | - | - | - | - | - |
| AL590842\_YPO3594 | 4001721 | 4002770 | True | COG3520 | 2e-105 | 99.0 | 1 | 332 |
| AL590842\_YPO3595 | 4002896 | 4004212 | True | COG3456 | 9e-124 | 100.0 | 1 | 430 |
| AL590842\_YPO3596 | 4004212 | 4004757 | True | COG3521 | 2e-39 | 100.0 | 1 | 159 |
| AL590842\_YPO3597 | 4004760 | 4006106 | True | COG3522 | 1e-167 | 100.0 | 1 | 446 |
| AL590842\_YPO3598 | 4006106 | 4006873 | True | COG3455 | 2e-86 | 98.0 | 4 | 260 |
| AL590842\_YPO3599 | 4006884 | 4009487 | True | COG0542 | 0.0 | 99.0 | 1 | 784 |
| AL590842\_YPO3600 | 4009484 | 4010281 | True | - | - | - | - | - |
| AL590842\_YPO3601 | 4010278 | 4010964 | True | - | - | - | - | - |
| AL590842\_YPO3602 | 4010961 | 4012358 | True | COG3515 | 2e-36 | 82.0 | 1 | 285 |
| AL590842\_YPO3603 | 4012390 | 4015923 | True | COG3523 | 0.0 | 100.0 | 1 | 1188 |
| AL590842\_YPO3604 | 4016048 | 4016830 | True | COG3515 | 3e-44 | 78.0 | 12 | 284 |
| AL590842\_YPO3605 | 4016827 | 4017360 | True | COG3515 | 6e-19 | 50.0 | 13 | 188 |
| AL590842\_YPO3606 | 4017382 | 4019784 | True | COG3501 | 0.0 | 99.0 | 1 | 547 |
| AL590842\_YPO3607 | 4019790 | 4020248 | True | COG5435 | 5e-48 | 100.0 | 1 | 147 |
| AL590842\_YPO3608 | 4020241 | 4023105 | True | COG3209 | 1e-61 | 99.0 | 2 | 794 |
| AL590842\_YPO3609 | 4023133 | 4024503 | True | COG3209 | 8e-32 | 59.0 | 326 | 795 |
| AL590842\_YPO3610 | 4024505 | 4024990 | True | - | - | - | - | - |
| AL590842\_YPO3611 | 4025040 | 4025231 | False | - | - | - | - | - |
| AL590842\_YPO3612 | 4025613 | 4026020 | False | - | - | - | - | - |
| AL590842\_YPO3613 | 4026381 | 4028579 | True | COG3501 | 0.0 | 99.0 | 1 | 549 |
| AL590842\_YPO3614 | 4028582 | 4029004 | True | COG5435 | 6e-45 | 97.0 | 3 | 145 |
| AL590842\_YPO3615 | 4029049 | 4033587 | True | COG3209 | 3e-63 | 99.0 | 1 | 795 |
| AL590842\_YPO3615 | 4029049 | 4033587 | True | COG4104 | 4e-10 | 73.0 | 25 | 96 |
