# Supplementary material for: Dissecting the bacterial type VI secretion system by a genome wide in silico analysis: what can be learned from available microbial genomic resources?
Source: BMC Genomics. 2009 Mar 12;10:104. doi: 10.1186/1471-2164-10-104 (PMC2660368; doi:10.1186/1471-2164-10-104)
Supplement: Additional file 7 — Detailed description of all identified T6SS gene clusters. Archive containing the detailed description of each identified T6SS locus as an HTML file. [file 1471-2164-10-104-S7.tgz › LociHTML/HTML/AL646053C.html]

Locus AL646053C on Ralstonia solanacearum (strain GMI1000) plasmid megaplasmid Rsp, complete sequence.

import namespace="svg" implementation="#AdobeSVG"?


# Locus AL646053C

# List of CDS in T6SS locus AL646053C

|  |  |  |  |  |  |  |  |  |
| --- | --- | --- | --- | --- | --- | --- | --- | --- |
| Name | from | to | direct | COG | e-value | COG cover | COG hit start | COG hit end |
| AL646053\_RSp0732 | 919454 | 921946 | True | - | - | - | - | - |
| AL646053\_RSp0733 | 922097 | 923044 | False | - | - | - | - | - |
| AL646053\_RSp0734 | 923063 | 923980 | False | - | - | - | - | - |
| AL646053\_RSp0735 | 924024 | 925265 | False | - | - | - | - | - |
| AL646053\_RSp0736 | 925361 | 925795 | False | - | - | - | - | - |
| AL646053\_RSp0737 | 925792 | 926493 | False | - | - | - | - | - |
| AL646053\_RSp0738 | 926494 | 929190 | False | COG4253 | 1e-67 | 99.0 | 1 | 277 |
| AL646053\_RSp0738 | 926494 | 929190 | False | COG3501 | 2e-128 | 98.0 | 2 | 543 |
| AL646053\_RSp0739 | 929249 | 930040 | False | COG3455 | 7e-63 | 96.0 | 10 | 262 |
| AL646053\_RSp0740 | 930037 | 931383 | False | COG3522 | 7e-136 | 100.0 | 1 | 446 |
| AL646053\_RSp0741 | 931427 | 932008 | False | COG3521 | 4e-33 | 98.0 | 4 | 159 |
| AL646053\_RSp0742 | 932376 | 933023 | True | - | - | - | - | - |
| AL646053\_RSp0743 | 933060 | 933572 | True | COG3516 | 4e-52 | 98.0 | 2 | 167 |
| AL646053\_RSp0744 | 933565 | 935055 | True | COG3517 | 0.0 | 100.0 | 1 | 495 |
| AL646053\_RSp0745 | 935144 | 935647 | True | COG3157 | 8e-38 | 99.0 | 2 | 162 |
| AL646053\_RSp0746 | 935708 | 936181 | True | COG3518 | 2e-39 | 98.0 | 4 | 157 |
| AL646053\_RSp0747 | 936244 | 938094 | True | COG3519 | 0.0 | 99.0 | 2 | 621 |
| AL646053\_RSp0748 | 938058 | 939149 | True | COG3520 | 6e-84 | 100.0 | 1 | 335 |
| AL646053\_RSp0749 | 939182 | 941899 | True | COG0542 | 0.0 | 98.0 | 1 | 776 |
| AL646053\_RSp0750 | 941920 | 944676 | True | COG4253 | 1e-50 | 85.0 | 1 | 239 |
| AL646053\_RSp0750 | 941920 | 944676 | True | COG3501 | 3e-124 | 99.0 | 1 | 549 |
| AL646053\_RSp0751 | 944694 | 945827 | True | - | - | - | - | - |
| AL646053\_RSp0752 | 945805 | 948582 | True | - | - | - | - | - |
| AL646053\_RSp0753 | 948595 | 949701 | True | - | - | - | - | - |
