# Supplementary material for: Dissecting the bacterial type VI secretion system by a genome wide in silico analysis: what can be learned from available microbial genomic resources?
Source: BMC Genomics. 2009 Mar 12;10:104. doi: 10.1186/1471-2164-10-104 (PMC2660368; doi:10.1186/1471-2164-10-104)
Supplement: Additional file 7 — Detailed description of all identified T6SS gene clusters. Archive containing the detailed description of each identified T6SS locus as an HTML file. [file 1471-2164-10-104-S7.tgz › LociHTML/HTML/AM039952A.html]

Locus AM039952A on Xanthomonas campestris (pathovar vesicatoria, strain 85-10) chromosome, complete sequence.

import namespace="svg" implementation="#AdobeSVG"?


# Locus AM039952A

# List of CDS in T6SS locus AM039952A

|  |  |  |  |  |  |  |  |  |
| --- | --- | --- | --- | --- | --- | --- | --- | --- |
| Name | from | to | direct | COG | e-value | COG cover | COG hit start | COG hit end |
| AM039952\_XCV2115 | 2426147 | 2427460 | True | COG2133 | 1e-71 | 99.0 | 2 | 397 |
| AM039952\_XCV2116 | 2427704 | 2428738 | True | COG0673 | 2e-42 | 95.0 | 4 | 329 |
| AM039952\_XCV2117 | 2429057 | 2429281 | False | - | - | - | - | - |
| AM039952\_XCV2118 | 2429665 | 2430627 | False | - | - | - | - | - |
| AM039952\_XCV2119 | 2430754 | 2431170 | False | - | - | - | - | - |
| AM039952\_XCV2120 | 2431311 | 2431817 | True | COG3516 | 1e-50 | 97.0 | 2 | 165 |
| AM039952\_XCV2121 | 2431753 | 2433327 | True | COG3517 | 0.0 | 100.0 | 1 | 495 |
| AM039952\_XCV2122 | 2433427 | 2433930 | True | COG3157 | 5e-22 | 93.0 | 1 | 151 |
| AM039952\_XCV2123 | 2433957 | 2434796 | True | COG4455 | 2e-48 | 94.0 | 9 | 265 |
| AM039952\_XCV2124 | 2434784 | 2435287 | True | COG3518 | 6e-21 | 99.0 | 1 | 156 |
| AM039952\_XCV2125 | 2435290 | 2437167 | True | COG3519 | 2e-173 | 100.0 | 1 | 621 |
| AM039952\_XCV2126 | 2437131 | 2438141 | True | COG3520 | 2e-72 | 99.0 | 1 | 333 |
| AM039952\_XCV2127 | 2438174 | 2440912 | True | COG0542 | 3e-127 | 64.0 | 1 | 505 |
| AM039952\_XCV2127 | 2438174 | 2440912 | True | COG0542 | 6e-103 | 42.0 | 434 | 767 |
| AM039952\_XCV2128 | 2440984 | 2441373 | True | - | - | - | - | - |
| AM039952\_XCV2129 | 2441565 | 2442113 | True | - | - | - | - | - |
| AM039952\_XCV2130 | 2443138 | 2443392 | True | - | - | - | - | - |
| AM039952\_XCV2131 | 2443377 | 2443856 | True | - | - | - | - | - |
| AM039952\_XCV2132 | 2443853 | 2444422 | True | - | - | - | - | - |
| AM039952\_XCV2133 | 2444452 | 2446536 | True | COG3501 | 2e-127 | 96.0 | 10 | 539 |
| AM039952\_XCV2134 | 2446545 | 2447084 | True | - | - | - | - | - |
| AM039952\_XCV2135 | 2447081 | 2448466 | True | COG3456 | 3e-39 | 99.0 | 1 | 429 |
| AM039952\_XCV2136 | 2448463 | 2449800 | True | COG3522 | 5e-132 | 100.0 | 1 | 446 |
| AM039952\_XCV2137 | 2449802 | 2451118 | True | COG3455 | 1e-47 | 90.0 | 22 | 259 |
| AM039952\_XCV2137 | 2449802 | 2451118 | True | COG1360 | 4e-21 | 50.0 | 123 | 244 |
| AM039952\_XCV2138 | 2451122 | 2454580 | True | COG3523 | 0.0 | 99.0 | 3 | 1184 |
| AM039952\_XCV2139 | 2454577 | 2455230 | True | COG3913 | 2e-13 | 43.0 | 1 | 98 |
| AM039952\_XCV2140 | 2455227 | 2455949 | True | COG0631 | 2e-37 | 94.0 | 4 | 251 |
| AM039952\_XCV2141 | 2455946 | 2458849 | True | COG0515 | 2e-35 | 58.0 | 2 | 225 |
| AM039952\_XCV2141 | 2455946 | 2458849 | True | COG1262 | 4e-09 | 61.0 | 86 | 277 |
| AM039952\_XCV2142 | 2458846 | 2459172 | True | - | - | - | - | - |
| AM039952\_XCV2143 | 2459341 | 2460369 | False | COG3515 | 3e-17 | 98.0 | 2 | 341 |
| AM039952\_XCV2144 | 2460378 | 2461349 | False | COG0583 | 6e-17 | 99.0 | 2 | 297 |
| AM039952\_XCV2145 | 2461463 | 2463919 | False | COG0642 | 2e-27 | 87.0 | 38 | 330 |
| AM039952\_XCV2146 | 2463916 | 2464908 | False | COG1858 | 2e-50 | 96.0 | 10 | 362 |
| AM039952\_XCV2147 | 2464905 | 2465684 | False | COG0745 | 2e-48 | 99.0 | 2 | 228 |
