# Supplementary material for: Dissecting the bacterial type VI secretion system by a genome wide in silico analysis: what can be learned from available microbial genomic resources?
Source: BMC Genomics. 2009 Mar 12;10:104. doi: 10.1186/1471-2164-10-104 (PMC2660368; doi:10.1186/1471-2164-10-104)
Supplement: Additional file 7 — Detailed description of all identified T6SS gene clusters. Archive containing the detailed description of each identified T6SS locus as an HTML file. [file 1471-2164-10-104-S7.tgz › LociHTML/HTML/AM039952C.html]

Locus AM039952C on Xanthomonas campestris (pathovar vesicatoria, strain 85-10) chromosome, complete sequence.

import namespace="svg" implementation="#AdobeSVG"?


# Locus AM039952C

# List of CDS in T6SS locus AM039952C

|  |  |  |  |  |  |  |  |  |
| --- | --- | --- | --- | --- | --- | --- | --- | --- |
| Name | from | to | direct | COG | e-value | COG cover | COG hit start | COG hit end |
| AM039952\_XCV4204 | 4823183 | 4824865 | False | COG2831 | 4e-55 | 92.0 | 44 | 554 |
| AM039952\_XCV4205 | 4824856 | 4825317 | False | - | - | - | - | - |
| AM039952\_XCV4206 | 4825314 | 4829009 | False | COG0515 | 3e-37 | 57.0 | 2 | 222 |
| AM039952\_XCV4206 | 4825314 | 4829009 | False | COG1262 | 1e-11 | 61.0 | 86 | 277 |
| AM039952\_XCV4207 | 4829014 | 4829730 | False | COG0631 | 4e-37 | 95.0 | 4 | 253 |
| AM039952\_XCV4208 | 4829727 | 4830311 | False | COG3913 | 2e-15 | 59.0 | 8 | 143 |
| AM039952\_XCV4209 | 4830299 | 4833823 | False | COG3523 | 0.0 | 99.0 | 5 | 1183 |
| AM039952\_XCV4210 | 4833826 | 4835082 | False | COG3455 | 2e-51 | 97.0 | 7 | 262 |
| AM039952\_XCV4210 | 4833826 | 4835082 | False | COG1360 | 2e-22 | 57.0 | 103 | 243 |
| AM039952\_XCV4211 | 4835085 | 4836419 | False | COG3522 | 1e-130 | 99.0 | 2 | 446 |
| AM039952\_XCV4212 | 4836922 | 4837812 | False | COG0667 | 4e-54 | 97.0 | 5 | 312 |
| AM039952\_XCV4213 | 4838041 | 4838943 | True | COG0583 | 4e-35 | 96.0 | 3 | 288 |
| AM039952\_XCV4214 | 4839002 | 4840333 | False | COG3456 | 6e-39 | 99.0 | 3 | 428 |
| AM039952\_XCV4215 | 4840409 | 4840642 | False | - | - | - | - | - |
| AM039952\_XCV4216 | 4840653 | 4841201 | False | - | - | - | - | - |
| AM039952\_XCV4217 | 4841198 | 4843165 | False | COG3501 | 3e-116 | 96.0 | 11 | 539 |
| AM039952\_XCV4218 | 4843315 | 4844646 | False | COG4976 | 3e-46 | 94.0 | 18 | 287 |
| AM039952\_XCV4218 | 4843315 | 4844646 | False | COG3063 | 1e-08 | 61.0 | 47 | 200 |
| AM039952\_XCV4219 | 4844882 | 4845196 | False | COG4104 | 9e-11 | 98.0 | 1 | 97 |
| AM039952\_XCV4220 | 4845240 | 4847741 | False | COG0515 | 9e-38 | 72.0 | 2 | 279 |
| AM039952\_XCV4221 | 4847738 | 4848304 | False | COG1595 | 6e-08 | 86.0 | 18 | 175 |
