# Supplementary material for: Dissecting the bacterial type VI secretion system by a genome wide in silico analysis: what can be learned from available microbial genomic resources?
Source: BMC Genomics. 2009 Mar 12;10:104. doi: 10.1186/1471-2164-10-104 (PMC2660368; doi:10.1186/1471-2164-10-104)
Supplement: Additional file 7 — Detailed description of all identified T6SS gene clusters. Archive containing the detailed description of each identified T6SS locus as an HTML file. [file 1471-2164-10-104-S7.tgz › LociHTML/HTML/AM039952D.html]

Locus AM039952D on Xanthomonas campestris (pathovar vesicatoria, strain 85-10) chromosome, complete sequence.

import namespace="svg" implementation="#AdobeSVG"?


# Locus AM039952D

# List of CDS in T6SS locus AM039952D

|  |  |  |  |  |  |  |  |  |
| --- | --- | --- | --- | --- | --- | --- | --- | --- |
| Name | from | to | direct | COG | e-value | COG cover | COG hit start | COG hit end |
| AM039952\_XCV4228 | 4857766 | 4858425 | False | - | - | - | - | - |
| AM039952\_XCV4229 | 4858477 | 4858866 | False | - | - | - | - | - |
| AM039952\_XCV4230 | 4858983 | 4859192 | False | - | - | - | - | - |
| AM039952\_XCV4231 | 4859397 | 4859939 | False | - | - | - | - | - |
| AM039952\_XCV4232 | 4860021 | 4860329 | False | - | - | - | - | - |
| AM039952\_XCV4233 | 4861394 | 4861891 | False | - | - | - | - | - |
| AM039952\_XCV4234 | 4862063 | 4862329 | True | - | - | - | - | - |
| AM039952\_XCV4235 | 4862362 | 4863195 | True | COG2801 | 1e-12 | 94.0 | 12 | 230 |
| AM039952\_XCV4236 | 4863335 | 4866097 | False | COG0542 | 1e-125 | 64.0 | 1 | 505 |
| AM039952\_XCV4236 | 4863335 | 4866097 | False | COG0542 | 7e-104 | 49.0 | 394 | 783 |
| AM039952\_XCV4237 | 4866154 | 4867194 | False | COG3520 | 3e-64 | 95.0 | 2 | 321 |
| AM039952\_XCV4238 | 4867158 | 4869068 | False | COG3519 | 3e-179 | 100.0 | 1 | 621 |
| AM039952\_XCV4239 | 4869046 | 4869549 | False | COG3518 | 6e-20 | 97.0 | 1 | 153 |
| AM039952\_XCV4240 | 4869555 | 4870349 | False | COG4455 | 4e-54 | 96.0 | 6 | 268 |
| AM039952\_XCV4241 | 4870553 | 4871056 | False | COG3157 | 3e-22 | 93.0 | 1 | 152 |
| AM039952\_XCV4242 | 4871139 | 4872632 | False | COG3517 | 0.0 | 99.0 | 4 | 494 |
| AM039952\_XCV4243 | 4872637 | 4873146 | False | COG3516 | 9e-54 | 100.0 | 1 | 169 |
| AM039952\_XCV4244 | 4873456 | 4874127 | True | COG4977 | 2e-10 | 33.0 | 217 | 326 |
| AM039952\_XCV4245 | 4874224 | 4874670 | False | COG0589 | 2e-16 | 96.0 | 4 | 151 |
| AM039952\_XCV4246 | 4874921 | 4875544 | False | COG0110 | 1e-29 | 94.0 | 6 | 184 |
| AM039952\_XCV4247 | 4875802 | 4876164 | True | - | - | - | - | - |
| AM039952\_XCV4248 | 4876347 | 4878518 | False | COG0210 | 1e-174 | 99.0 | 1 | 654 |
