# Supplementary material for: Dissecting the bacterial type VI secretion system by a genome wide in silico analysis: what can be learned from available microbial genomic resources?
Source: BMC Genomics. 2009 Mar 12;10:104. doi: 10.1186/1471-2164-10-104 (PMC2660368; doi:10.1186/1471-2164-10-104)
Supplement: Additional file 7 — Detailed description of all identified T6SS gene clusters. Archive containing the detailed description of each identified T6SS locus as an HTML file. [file 1471-2164-10-104-S7.tgz › LociHTML/HTML/AM167904A.html]

Locus AM167904A on Bordetella avium (strain 197N) chromosome, complete sequence.

import namespace="svg" implementation="#AdobeSVG"?


# Locus AM167904A

# List of CDS in T6SS locus AM167904A

|  |  |  |  |  |  |  |  |  |
| --- | --- | --- | --- | --- | --- | --- | --- | --- |
| Name | from | to | direct | COG | e-value | COG cover | COG hit start | COG hit end |
| AM167904\_BAV0261 | 268341 | 268616 | False | - | - | - | - | - |
| AM167904\_BAV0262 | 268628 | 270964 | False | COG0446 | 3e-46 | 81.0 | 1 | 338 |
| AM167904\_BAV0262 | 268628 | 270964 | False | COG1752 | 2e-25 | 77.0 | 14 | 249 |
| AM167904\_BAV0263 | 271008 | 271793 | False | COG1028 | 8e-37 | 99.0 | 2 | 250 |
| AM167904\_BAV0264 | 271841 | 272581 | False | COG4689 | 6e-114 | 99.0 | 1 | 246 |
| AM167904\_BAV0265 | 273187 | 273738 | True | COG3516 | 4e-43 | 99.0 | 2 | 169 |
| AM167904\_BAV0266 | 273790 | 275283 | True | COG3517 | 0.0 | 99.0 | 1 | 494 |
| AM167904\_BAV0267 | 275344 | 275820 | True | COG3157 | 1e-19 | 98.0 | 3 | 162 |
| AM167904\_BAV0268 | 275829 | 276233 | True | COG3518 | 4e-13 | 88.0 | 8 | 146 |
| AM167904\_BAV0269 | 276250 | 277992 | True | COG3519 | 2e-98 | 98.0 | 3 | 616 |
| AM167904\_BAV0270 | 277989 | 278903 | True | COG3520 | 6e-35 | 91.0 | 15 | 320 |
| AM167904\_BAV0271 | 278906 | 281524 | True | COG0542 | 0.0 | 96.0 | 1 | 761 |
| AM167904\_BAV0272 | 281517 | 283367 | True | COG3501 | 2e-115 | 96.0 | 23 | 550 |
| AM167904\_BAV0273 | 283367 | 283996 | True | COG2849 | 7e-16 | 56.0 | 101 | 230 |
| AM167904\_BAV0274 | 283993 | 284379 | True | - | - | - | - | - |
| AM167904\_BAV0275 | 284383 | 284535 | True | - | - | - | - | - |
| AM167904\_BAV0276 | 284535 | 285467 | True | COG0834 | 2e-15 | 80.0 | 27 | 246 |
| AM167904\_BAV0277 | 285511 | 286755 | True | COG1301 | 6e-35 | 91.0 | 12 | 392 |
| AM167904\_BAV0278 | 286850 | 288160 | True | COG1301 | 2e-22 | 94.0 | 21 | 414 |
| AM167904\_BAV0279 | 288157 | 288609 | True | - | - | - | - | - |
| AM167904\_BAV0280 | 288651 | 289940 | True | COG3522 | 7e-30 | 70.0 | 5 | 317 |
| AM167904\_BAV0281 | 289937 | 290680 | True | COG3455 | 3e-14 | 77.0 | 50 | 251 |
| AM167904\_BAV0282 | 290714 | 294478 | True | COG3523 | 4e-17 | 37.0 | 49 | 497 |
| AM167904\_BAV0283 | 294475 | 295488 | True | COG3515 | 1e-17 | 97.0 | 7 | 342 |
| AM167904\_BAV0284 | 295613 | 296482 | True | - | - | - | - | - |
| AM167904\_BAV0285 | 296612 | 298021 | False | COG0154 | 3e-112 | 99.0 | 1 | 474 |
| AM167904\_BAV0286 | 298069 | 299295 | False | COG0683 | 4e-39 | 98.0 | 3 | 363 |
| AM167904\_BAV0287 | 299415 | 300341 | True | COG0583 | 1e-27 | 99.0 | 1 | 296 |
| AM167904\_BAV0288 | 300438 | 301427 | True | COG4177 | 1e-31 | 100.0 | 1 | 314 |
