# Supplementary material for: Dissecting the bacterial type VI secretion system by a genome wide in silico analysis: what can be learned from available microbial genomic resources?
Source: BMC Genomics. 2009 Mar 12;10:104. doi: 10.1186/1471-2164-10-104 (PMC2660368; doi:10.1186/1471-2164-10-104)
Supplement: Additional file 7 — Detailed description of all identified T6SS gene clusters. Archive containing the detailed description of each identified T6SS locus as an HTML file. [file 1471-2164-10-104-S7.tgz › LociHTML/HTML/AM236086B.html]

Locus AM236086B on Rhizobium leguminosarum bv. viciae (strain 3841) plasmid pRL12, complete sequence.

import namespace="svg" implementation="#AdobeSVG"?


# Locus AM236086B

# List of CDS in T6SS locus AM236086B

|  |  |  |  |  |  |  |  |  |
| --- | --- | --- | --- | --- | --- | --- | --- | --- |
| Name | from | to | direct | COG | e-value | COG cover | COG hit start | COG hit end |
| AM236086\_pRL120456 | 491368 | 492300 | False | - | - | - | - | - |
| AM236086\_pRL120457 | 492421 | 493320 | True | COG0583 | 2e-40 | 98.0 | 2 | 293 |
| AM236086\_pRL120458 | 493557 | 493889 | False | - | - | - | - | - |
| AM236086\_pRL120459 | 493892 | 494557 | False | COG3652 | 2e-28 | 95.0 | 8 | 170 |
| AM236086\_pRL120460 | 494565 | 494912 | False | COG3189 | 3e-36 | 95.0 | 3 | 114 |
| AM236086\_pRL120461 | 495175 | 496314 | True | COG1596 | 2e-32 | 99.0 | 2 | 239 |
| AM236086\_pRL120462 | 496311 | 497225 | False | COG0515 | 8e-23 | 53.0 | 1 | 206 |
| AM236086\_pRL120463 | 497215 | 498588 | False | COG3913 | 3e-36 | 94.0 | 1 | 214 |
| AM236086\_pRL120463 | 497215 | 498588 | False | COG0631 | 3e-30 | 94.0 | 5 | 252 |
| AM236086\_pRL120464 | 498599 | 502075 | False | COG3523 | 0.0 | 99.0 | 7 | 1187 |
| AM236086\_pRL120465 | 502072 | 503604 | False | COG3455 | 1e-59 | 96.0 | 8 | 260 |
| AM236086\_pRL120465 | 502072 | 503604 | False | COG1360 | 5e-32 | 90.0 | 23 | 244 |
| AM236086\_pRL120466 | 503601 | 504941 | False | COG3522 | 6e-147 | 100.0 | 1 | 446 |
| AM236086\_pRL120467 | 504931 | 506130 | False | COG3456 | 2e-25 | 87.0 | 1 | 378 |
| AM236086\_pRL120468 | 506142 | 507143 | False | COG3520 | 3e-57 | 97.0 | 1 | 325 |
| AM236086\_pRL120469 | 507164 | 508945 | False | COG3519 | 7e-150 | 100.0 | 1 | 621 |
| AM236086\_pRL120470 | 508938 | 509447 | False | COG3518 | 2e-24 | 94.0 | 7 | 155 |
| AM236086\_pRL120471 | 509440 | 510270 | False | COG4455 | 6e-62 | 95.0 | 2 | 263 |
| AM236086\_pRL120472 | 510267 | 511655 | False | COG3517 | 4e-144 | 85.0 | 68 | 489 |
| AM236086\_pRL120473 | 511731 | 513212 | False | COG3517 | 0.0 | 99.0 | 1 | 494 |
| AM236086\_pRL120474 | 513343 | 513888 | False | COG3516 | 6e-51 | 100.0 | 1 | 169 |
| AM236086\_pRL120475 | 513925 | 514917 | False | COG3515 | 3e-29 | 92.0 | 26 | 346 |
| AM236086\_pRL120477 | 517603 | 518079 | True | COG3157 | 9e-29 | 98.0 | 3 | 162 |
| AM236086\_pRL120478 | 518229 | 519305 | True | - | - | - | - | - |
| AM236086\_pRL120479 | 519351 | 520244 | True | - | - | - | - | - |
| AM236086\_pRL120480 | 520276 | 522540 | True | COG3501 | 4e-148 | 99.0 | 2 | 550 |
| AM236086\_pRL120481 | 522554 | 523663 | True | COG5351 | 3e-100 | 99.0 | 1 | 366 |
| AM236086\_pRL120482 | 523660 | 525162 | True | - | - | - | - | - |
| AM236086\_pRL120483 | 525159 | 526274 | True | - | - | - | - | - |
| AM236086\_pRL120484 | 526530 | 527630 | True | - | - | - | - | - |
