# Supplementary material for: Dissecting the bacterial type VI secretion system by a genome wide in silico analysis: what can be learned from available microbial genomic resources?
Source: BMC Genomics. 2009 Mar 12;10:104. doi: 10.1186/1471-2164-10-104 (PMC2660368; doi:10.1186/1471-2164-10-104)
Supplement: Additional file 7 — Detailed description of all identified T6SS gene clusters. Archive containing the detailed description of each identified T6SS locus as an HTML file. [file 1471-2164-10-104-S7.tgz › LociHTML/HTML/AM260479A.html]

Locus AM260479A on Ralstonia eutropha (strain ATCC 17699 / H16 / DSM 428 / Stanier 337) chromosome 1, complete sequence.

import namespace="svg" implementation="#AdobeSVG"?


# Locus AM260479A

# List of CDS in T6SS locus AM260479A

|  |  |  |  |  |  |  |  |  |
| --- | --- | --- | --- | --- | --- | --- | --- | --- |
| Name | from | to | direct | COG | e-value | COG cover | COG hit start | COG hit end |
| AM260479\_H16\_A0641 | 679719 | 681281 | True | COG1894 | 2e-160 | 98.0 | 1 | 416 |
| AM260479\_H16\_A0642 | 681317 | 684196 | True | COG3383 | 0.0 | 98.0 | 2 | 964 |
| AM260479\_H16\_A0643 | 684212 | 685078 | True | COG1526 | 1e-73 | 90.0 | 24 | 264 |
| AM260479\_H16\_A0644 | 685075 | 685299 | True | - | - | - | - | - |
| AM260479\_H16\_A0645 | 685318 | 686364 | False | COG3515 | 5e-25 | 95.0 | 7 | 338 |
| AM260479\_H16\_A0646 | 686367 | 687407 | False | COG3520 | 1e-77 | 100.0 | 1 | 335 |
| AM260479\_H16\_A0647 | 687410 | 689320 | False | COG3519 | 0.0 | 99.0 | 1 | 618 |
| AM260479\_H16\_A0648 | 689295 | 689816 | False | COG3518 | 1e-18 | 94.0 | 6 | 154 |
| AM260479\_H16\_A0649 | 689809 | 690645 | False | COG4455 | 2e-65 | 95.0 | 9 | 268 |
| AM260479\_H16\_A0650 | 690635 | 691237 | False | - | - | - | - | - |
| AM260479\_H16\_A0651 | 691692 | 694406 | True | COG0542 | 0.0 | 96.0 | 1 | 760 |
| AM260479\_H16\_A0652 | 694446 | 694979 | True | COG3516 | 7e-58 | 100.0 | 1 | 169 |
| AM260479\_H16\_A0653 | 695029 | 696522 | True | COG3517 | 0.0 | 99.0 | 4 | 495 |
| AM260479\_H16\_A0654 | 696651 | 697136 | True | COG3157 | 1e-38 | 100.0 | 1 | 162 |
| AM260479\_H16\_A0655 | 697450 | 698544 | True | - | - | - | - | - |
| AM260479\_H16\_A0656 | 698568 | 701162 | True | COG4253 | 7e-51 | 99.0 | 1 | 276 |
| AM260479\_H16\_A0656 | 698568 | 701162 | True | COG3501 | 2e-118 | 91.0 | 24 | 526 |
| AM260479\_H16\_A0657 | 701196 | 703544 | True | - | - | - | - | - |
| AM260479\_H16\_A0658 | 703636 | 705096 | True | - | - | - | - | - |
| AM260479\_H16\_A0659 | 705214 | 706671 | True | - | - | - | - | - |
| AM260479\_H16\_A0660 | 707107 | 707613 | True | COG3521 | 5e-31 | 85.0 | 8 | 143 |
| AM260479\_H16\_A0661 | 707618 | 708961 | True | COG3522 | 1e-135 | 100.0 | 1 | 446 |
| AM260479\_H16\_A0662 | 708958 | 710235 | True | COG3455 | 1e-53 | 85.0 | 38 | 261 |
| AM260479\_H16\_A0662 | 708958 | 710235 | True | COG1360 | 1e-25 | 57.0 | 103 | 242 |
| AM260479\_H16\_A0663 | 710248 | 714069 | True | COG3523 | 0.0 | 99.0 | 2 | 1186 |
| AM260479\_H16\_A0664 | 714048 | 715145 | True | - | - | - | - | - |
| AM260479\_H16\_A0665 | 714828 | 715523 | False | COG5009 | 2e-75 | 31.0 | 1 | 252 |
| AM260479\_H16\_A0666 | 715582 | 716631 | False | COG2055 | 2e-72 | 99.0 | 4 | 349 |
| AM260479\_H16\_A0667 | 716645 | 718186 | False | COG0471 | 4e-38 | 98.0 | 6 | 461 |
| AM260479\_H16\_A0668 | 718621 | 718866 | True | - | - | - | - | - |
| AM260479\_H16\_A0669 | 718917 | 719738 | True | COG2365 | 4e-20 | 93.0 | 8 | 240 |
