# Supplementary material for: Dissecting the bacterial type VI secretion system by a genome wide in silico analysis: what can be learned from available microbial genomic resources?
Source: BMC Genomics. 2009 Mar 12;10:104. doi: 10.1186/1471-2164-10-104 (PMC2660368; doi:10.1186/1471-2164-10-104)
Supplement: Additional file 7 — Detailed description of all identified T6SS gene clusters. Archive containing the detailed description of each identified T6SS locus as an HTML file. [file 1471-2164-10-104-S7.tgz › LociHTML/HTML/AM260480F.html]

Locus AM260480F on Ralstonia eutropha (strain ATCC 17699 / H16 / DSM 428 / Stanier 337) chromosome 2, complete sequence.

import namespace="svg" implementation="#AdobeSVG"?


# Locus AM260480F

# List of CDS in T6SS locus AM260480F

|  |  |  |  |  |  |  |  |  |
| --- | --- | --- | --- | --- | --- | --- | --- | --- |
| Name | from | to | direct | COG | e-value | COG cover | COG hit start | COG hit end |
| AM260480\_H16\_B2408 | 2719224 | 2720498 | False | COG0845 | 1e-16 | 86.0 | 26 | 348 |
| AM260480\_H16\_B2409 | 2720515 | 2721786 | False | COG1538 | 1e-23 | 92.0 | 24 | 444 |
| AM260480\_H16\_B2410 | 2721884 | 2722258 | False | - | - | - | - | - |
| AM260480\_H16\_B2411 | 2722544 | 2722888 | True | - | - | - | - | - |
| AM260480\_H16\_B2412 | 2722941 | 2723147 | False | - | - | - | - | - |
| AM260480\_H16\_B2413 | 2723190 | 2723783 | False | COG1595 | 2e-18 | 86.0 | 19 | 176 |
| AM260480\_H16\_B2414 | 2723870 | 2725033 | False | - | - | - | - | - |
| AM260480\_H16\_B2415 | 2725094 | 2726179 | False | COG3515 | 8e-25 | 98.0 | 1 | 342 |
| AM260480\_H16\_B2416 | 2726176 | 2730294 | False | COG3523 | 6e-29 | 37.0 | 49 | 495 |
| AM260480\_H16\_B2417 | 2730332 | 2731084 | False | COG3455 | 6e-17 | 79.0 | 39 | 247 |
| AM260480\_H16\_B2418 | 2731111 | 2732439 | False | COG3522 | 1e-35 | 70.0 | 1 | 316 |
| AM260480\_H16\_B2419 | 2732485 | 2732988 | False | - | - | - | - | - |
| AM260480\_H16\_B2420 | 2733018 | 2734472 | False | COG1301 | 2e-27 | 92.0 | 12 | 395 |
| AM260480\_H16\_B2421 | 2734469 | 2735191 | False | COG1794 | 4e-35 | 99.0 | 1 | 229 |
| AM260480\_H16\_B2422 | 2735203 | 2736537 | False | COG1301 | 7e-40 | 93.0 | 10 | 395 |
| AM260480\_H16\_B2423 | 2736534 | 2737283 | False | COG0834 | 2e-18 | 81.0 | 24 | 248 |
| AM260480\_H16\_B2424 | 2737527 | 2737787 | False | - | - | - | - | - |
| AM260480\_H16\_B2425 | 2737805 | 2738191 | False | - | - | - | - | - |
| AM260480\_H16\_B2426 | 2738194 | 2738856 | False | COG2849 | 3e-18 | 83.0 | 39 | 229 |
| AM260480\_H16\_B2427 | 2738915 | 2740816 | False | COG3501 | 2e-123 | 98.0 | 1 | 542 |
| AM260480\_H16\_B2428 | 2740820 | 2743540 | False | COG0542 | 0.0 | 96.0 | 1 | 762 |
| AM260480\_H16\_B2429 | 2743552 | 2744598 | False | COG3520 | 1e-42 | 100.0 | 1 | 335 |
| AM260480\_H16\_B2430 | 2744553 | 2746328 | False | COG3519 | 1e-117 | 99.0 | 2 | 621 |
| AM260480\_H16\_B2431 | 2746321 | 2746776 | False | COG3518 | 2e-12 | 82.0 | 14 | 143 |
| AM260480\_H16\_B2432 | 2746789 | 2747313 | False | COG3157 | 4e-15 | 82.0 | 29 | 161 |
| AM260480\_H16\_B2433 | 2747374 | 2748885 | False | COG3517 | 0.0 | 99.0 | 3 | 495 |
| AM260480\_H16\_B2434 | 2748949 | 2749485 | False | COG3516 | 5e-44 | 99.0 | 2 | 169 |
| AM260480\_H16\_B2435 | 2750276 | 2750995 | True | COG2197 | 1e-33 | 86.0 | 29 | 210 |
| AM260480\_H16\_B2436 | 2751281 | 2751637 | True | - | - | - | - | - |
| AM260480\_H16\_B2437 | 2751899 | 2752792 | True | COG0583 | 3e-30 | 96.0 | 6 | 291 |
| AM260480\_H16\_B2438 | 2752996 | 2754228 | True | COG1804 | 3e-94 | 97.0 | 7 | 394 |
| AM260480\_H16\_B2439 | 2754334 | 2755311 | True | COG3181 | 2e-63 | 99.0 | 1 | 316 |
