# Supplementary material for: Dissecting the bacterial type VI secretion system by a genome wide in silico analysis: what can be learned from available microbial genomic resources?
Source: BMC Genomics. 2009 Mar 12;10:104. doi: 10.1186/1471-2164-10-104 (PMC2660368; doi:10.1186/1471-2164-10-104)
Supplement: Additional file 7 — Detailed description of all identified T6SS gene clusters. Archive containing the detailed description of each identified T6SS locus as an HTML file. [file 1471-2164-10-104-S7.tgz › LociHTML/HTML/AM286415A.html]

Locus AM286415A on Yersinia enterocolitica (serovar O:8 / biotype 1B, strain 8081) chromosome, complete sequence.

import namespace="svg" implementation="#AdobeSVG"?


# Locus AM286415A

# List of CDS in T6SS locus AM286415A

|  |  |  |  |  |  |  |  |  |
| --- | --- | --- | --- | --- | --- | --- | --- | --- |
| Name | from | to | direct | COG | e-value | COG cover | COG hit start | COG hit end |
| AM286415\_YE2678 | 2903500 | 2905104 | False | COG1263 | 9e-10 | 74.0 | 85 | 375 |
| AM286415\_YE2679 | 2905488 | 2906885 | False | COG0534 | 2e-66 | 96.0 | 15 | 453 |
| AM286415\_YE2680 | 2907123 | 2908487 | True | COG0137 | 4e-133 | 100.0 | 1 | 403 |
| AM286415\_YE2681 | 2908619 | 2909098 | False | COG3518 | 1e-30 | 99.0 | 1 | 156 |
| AM286415\_YE2682 | 2909098 | 2909925 | False | COG4455 | 9e-85 | 97.0 | 1 | 265 |
| AM286415\_YE2683 | 2909951 | 2910793 | False | - | - | - | - | - |
| AM286415\_YE2684 | 2911171 | 2913885 | False | COG3501 | 7e-146 | 98.0 | 6 | 548 |
| AM286415\_YE2684 | 2911171 | 2913885 | False | COG0810 | 1e-07 | 59.0 | 9 | 152 |
| AM286415\_YE2685 | 2914034 | 2917867 | False | COG3523 | 0.0 | 99.0 | 3 | 1187 |
| AM286415\_YE2686 | 2917876 | 2919252 | False | COG3455 | 5e-51 | 95.0 | 11 | 260 |
| AM286415\_YE2686 | 2917876 | 2919252 | False | COG1360 | 6e-30 | 79.0 | 48 | 240 |
| AM286415\_YE2687 | 2919249 | 2920598 | False | COG3522 | 2e-154 | 99.0 | 2 | 446 |
| AM286415\_YE2688 | 2920602 | 2921153 | False | COG3521 | 9e-33 | 99.0 | 2 | 159 |
| AM286415\_YE2689 | 2921360 | 2921845 | False | COG3157 | 4e-40 | 100.0 | 1 | 162 |
| AM286415\_YE2690 | 2922177 | 2923604 | False | COG3517 | 0.0 | 94.0 | 27 | 495 |
| AM286415\_YE2691 | 2923697 | 2924221 | False | COG3516 | 6e-57 | 99.0 | 2 | 169 |
| AM286415\_YE2692 | 2924326 | 2924928 | False | COG3539 | 3e-14 | 91.0 | 16 | 184 |
| AM286415\_YE2693 | 2924913 | 2927588 | False | COG3188 | 1e-172 | 97.0 | 8 | 817 |
| AM286415\_YE2694 | 2927692 | 2928489 | False | COG3121 | 6e-57 | 99.0 | 1 | 234 |
| AM286415\_YE2695 | 2928597 | 2929157 | False | COG3539 | 3e-11 | 98.0 | 3 | 184 |
| AM286415\_YE2696 | 2929324 | 2931996 | False | COG0542 | 0.0 | 99.0 | 1 | 782 |
| AM286415\_YE2697 | 2932716 | 2934596 | True | COG3519 | 0.0 | 100.0 | 1 | 621 |
| AM286415\_YE2698 | 2934596 | 2935621 | True | COG3520 | 2e-87 | 100.0 | 1 | 335 |
| AM286415\_YE2699 | 2935696 | 2936859 | True | COG3515 | 3e-31 | 96.0 | 7 | 341 |
| AM286415\_YE2700 | 2936866 | 2937870 | True | - | - | - | - | - |
| AM286415\_YE2701 | 2937909 | 2939246 | True | - | - | - | - | - |
| AM286415\_YE2702 | 2939243 | 2939890 | True | - | - | - | - | - |
| AM286415\_YE2703 | 2939891 | 2941561 | True | COG2885 | 1e-23 | 54.0 | 87 | 190 |
| AM286415\_YE2704 | 2941568 | 2942053 | True | COG5435 | 3e-32 | 98.0 | 2 | 146 |
