# Supplementary material for: Dissecting the bacterial type VI secretion system by a genome wide in silico analysis: what can be learned from available microbial genomic resources?
Source: BMC Genomics. 2009 Mar 12;10:104. doi: 10.1186/1471-2164-10-104 (PMC2660368; doi:10.1186/1471-2164-10-104)
Supplement: Additional file 7 — Detailed description of all identified T6SS gene clusters. Archive containing the detailed description of each identified T6SS locus as an HTML file. [file 1471-2164-10-104-S7.tgz › LociHTML/HTML/AM406670A.html]

Locus AM406670A on Azoarcus sp. (strain BH72) chromosome, complete sequence.

import namespace="svg" implementation="#AdobeSVG"?


# Locus AM406670A

# List of CDS in T6SS locus AM406670A

|  |  |  |  |  |  |  |  |  |
| --- | --- | --- | --- | --- | --- | --- | --- | --- |
| Name | from | to | direct | COG | e-value | COG cover | COG hit start | COG hit end |
| AM406670\_azo1294 | 1406776 | 1410699 | True | COG3164 | 5e-154 | 99.0 | 3 | 1261 |
| AM406670\_azo1295 | 1410696 | 1411553 | True | COG0388 | 1e-51 | 95.0 | 2 | 262 |
| AM406670\_azo1296 | 1411624 | 1413069 | True | COG0312 | 5e-118 | 99.0 | 2 | 454 |
| AM406670\_azo1297 | 1413077 | 1413520 | False | COG3518 | 9e-15 | 92.0 | 8 | 153 |
| AM406670\_azo1298 | 1413533 | 1414231 | False | COG3455 | 2e-31 | 89.0 | 21 | 254 |
| AM406670\_azo1299 | 1414245 | 1415591 | False | COG3522 | 5e-80 | 99.0 | 1 | 444 |
| AM406670\_azo1300 | 1415603 | 1416217 | False | COG3521 | 4e-13 | 84.0 | 5 | 138 |
| AM406670\_azo1301 | 1416388 | 1417059 | True | - | - | - | - | - |
| AM406670\_azo1302 | 1417056 | 1420748 | True | COG3523 | 5e-102 | 98.0 | 16 | 1188 |
| AM406670\_azo1303 | 1420836 | 1421360 | True | COG3516 | 2e-47 | 97.0 | 2 | 166 |
| AM406670\_azo1304 | 1421344 | 1422837 | True | COG3517 | 0.0 | 99.0 | 3 | 494 |
| AM406670\_azo1305 | 1422881 | 1423378 | True | COG3157 | 6e-33 | 100.0 | 1 | 162 |
| AM406670\_azo1306 | 1423378 | 1423854 | True | - | - | - | - | - |
| AM406670\_azo1307 | 1423911 | 1426628 | True | COG4253 | 1e-37 | 99.0 | 1 | 276 |
| AM406670\_azo1307 | 1423911 | 1426628 | True | COG3501 | 1e-100 | 98.0 | 5 | 548 |
| AM406670\_azo1308 | 1426635 | 1428473 | True | COG3519 | 2e-145 | 100.0 | 1 | 621 |
| AM406670\_azo1309 | 1428437 | 1429453 | True | COG3520 | 2e-55 | 98.0 | 1 | 330 |
| AM406670\_azo1310 | 1429450 | 1430565 | False | COG3515 | 1e-19 | 99.0 | 4 | 346 |
| AM406670\_azo1311 | 1430572 | 1430835 | False | COG4104 | 3e-17 | 83.0 | 14 | 95 |
| AM406670\_azo1312 | 1431039 | 1432133 | True | COG2265 | 2e-64 | 80.0 | 84 | 432 |
| AM406670\_azo1313 | 1432168 | 1432506 | True | COG2824 | 3e-44 | 100.0 | 1 | 112 |
| AM406670\_azo1314 | 1432522 | 1432893 | False | - | - | - | - | - |
| AM406670\_azo1315 | 1432952 | 1433749 | False | COG0390 | 7e-49 | 98.0 | 1 | 252 |
| AM406670\_azo1316 | 1433746 | 1434351 | False | COG4619 | 1e-29 | 97.0 | 1 | 217 |
| AM406670\_azo1317 | 1434635 | 1435387 | True | - | - | - | - | - |
| AM406670\_azo1318 | 1435529 | 1436443 | False | COG4395 | 8e-18 | 99.0 | 3 | 281 |
