# Supplementary material for: Dissecting the bacterial type VI secretion system by a genome wide in silico analysis: what can be learned from available microbial genomic resources?
Source: BMC Genomics. 2009 Mar 12;10:104. doi: 10.1186/1471-2164-10-104 (PMC2660368; doi:10.1186/1471-2164-10-104)
Supplement: Additional file 7 — Detailed description of all identified T6SS gene clusters. Archive containing the detailed description of each identified T6SS locus as an HTML file. [file 1471-2164-10-104-S7.tgz › LociHTML/HTML/AM406670E.html]

Locus AM406670E on Azoarcus sp. (strain BH72) chromosome, complete sequence.

import namespace="svg" implementation="#AdobeSVG"?


# Locus AM406670E

# List of CDS in T6SS locus AM406670E

|  |  |  |  |  |  |  |  |  |
| --- | --- | --- | --- | --- | --- | --- | --- | --- |
| Name | from | to | direct | COG | e-value | COG cover | COG hit start | COG hit end |
| AM406670\_azo3869 | 4242264 | 4242509 | True | COG0508 | 3e-08 | 15.0 | 15 | 78 |
| AM406670\_azo3870 | 4242513 | 4243226 | True | COG0508 | 3e-52 | 59.0 | 159 | 400 |
| AM406670\_azo3871 | 4243226 | 4243996 | True | COG0095 | 1e-08 | 78.0 | 32 | 225 |
| AM406670\_azo3872 | 4244062 | 4244643 | True | - | - | - | - | - |
| AM406670\_azo3873 | 4244733 | 4246307 | True | COG2303 | 8e-52 | 97.0 | 10 | 536 |
| AM406670\_azo3874 | 4246369 | 4246983 | True | - | - | - | - | - |
| AM406670\_azo3875 | 4247215 | 4249182 | True | COG3284 | 5e-160 | 99.0 | 1 | 605 |
| AM406670\_azo3876 | 4249321 | 4251393 | True | COG3501 | 4e-163 | 98.0 | 8 | 550 |
| AM406670\_azo3877 | 4251398 | 4252432 | True | COG5351 | 8e-50 | 92.0 | 1 | 341 |
| AM406670\_azo3878 | 4252437 | 4253474 | True | - | - | - | - | - |
| AM406670\_azo3879 | 4253497 | 4254504 | True | - | - | - | - | - |
| AM406670\_azo3880 | 4254527 | 4255069 | True | - | - | - | - | - |
| AM406670\_azo3881 | 4255159 | 4255536 | True | - | - | - | - | - |
| AM406670\_azo3882 | 4255546 | 4255974 | True | - | - | - | - | - |
| AM406670\_azo3883 | 4255979 | 4257190 | True | - | - | - | - | - |
| AM406670\_azo3884 | 4257210 | 4259045 | True | COG3456 | 2e-15 | 59.0 | 3 | 258 |
| AM406670\_azo3884 | 4257210 | 4259045 | True | COG3456 | 3e-33 | 75.0 | 106 | 430 |
| AM406670\_azo3885 | 4259097 | 4259867 | True | COG0631 | 8e-34 | 97.0 | 5 | 259 |
| AM406670\_azo3886 | 4259941 | 4260594 | True | - | - | - | - | - |
| AM406670\_azo3887 | 4260635 | 4261894 | True | - | - | - | - | - |
| AM406670\_azo3888 | 4261940 | 4263946 | False | COG0515 | 1e-33 | 72.0 | 1 | 277 |
| AM406670\_azo3889 | 4264005 | 4264706 | False | COG3913 | 2e-25 | 90.0 | 8 | 212 |
| AM406670\_azo3890 | 4264709 | 4268281 | False | COG3523 | 0.0 | 99.0 | 4 | 1187 |
| AM406670\_azo3891 | 4268313 | 4269617 | False | COG3455 | 1e-53 | 98.0 | 1 | 258 |
| AM406670\_azo3891 | 4268313 | 4269617 | False | COG1360 | 2e-28 | 65.0 | 82 | 242 |
| AM406670\_azo3892 | 4269682 | 4271016 | False | COG3522 | 1e-125 | 99.0 | 1 | 445 |
| AM406670\_azo3893 | 4271065 | 4271583 | False | COG3521 | 1e-27 | 91.0 | 1 | 145 |
| AM406670\_azo3894 | 4271852 | 4272913 | True | COG3515 | 3e-20 | 100.0 | 1 | 346 |
| AM406670\_azo3895 | 4272962 | 4273471 | True | COG3516 | 9e-58 | 99.0 | 2 | 169 |
| AM406670\_azo3896 | 4273523 | 4275019 | True | COG3517 | 0.0 | 100.0 | 1 | 495 |
| AM406670\_azo3897 | 4275148 | 4275636 | True | COG3157 | 1e-30 | 98.0 | 1 | 160 |
| AM406670\_azo3898 | 4275831 | 4276337 | True | COG3157 | 1e-24 | 90.0 | 4 | 150 |
| AM406670\_azo3899 | 4276353 | 4277174 | True | COG4455 | 4e-69 | 95.0 | 6 | 267 |
| AM406670\_azo3900 | 4277167 | 4277676 | True | COG3518 | 8e-21 | 96.0 | 3 | 154 |
| AM406670\_azo3901 | 4277775 | 4279646 | True | COG3519 | 1e-179 | 100.0 | 1 | 621 |
| AM406670\_azo3902 | 4279622 | 4280665 | True | COG3520 | 2e-79 | 94.0 | 14 | 331 |
| AM406670\_azo3903 | 4280714 | 4283434 | True | COG0542 | 4e-128 | 64.0 | 1 | 506 |
| AM406670\_azo3903 | 4280714 | 4283434 | True | COG0542 | 2e-103 | 51.0 | 385 | 785 |
| AM406670\_azo3904 | 4283535 | 4283924 | True | - | - | - | - | - |
| AM406670\_azo3905 | 4283936 | 4285093 | True | - | - | - | - | - |
| AM406670\_azo3906 | 4285131 | 4286267 | True | - | - | - | - | - |
| AM406670\_azo3907 | 4286286 | 4287635 | True | COG4976 | 3e-46 | 95.0 | 12 | 285 |
| AM406670\_azo3908 | 4287636 | 4289066 | False | COG0515 | 1e-44 | 73.0 | 1 | 284 |
