# Supplementary material for: Dissecting the bacterial type VI secretion system by a genome wide in silico analysis: what can be learned from available microbial genomic resources?
Source: BMC Genomics. 2009 Mar 12;10:104. doi: 10.1186/1471-2164-10-104 (PMC2660368; doi:10.1186/1471-2164-10-104)
Supplement: Additional file 7 — Detailed description of all identified T6SS gene clusters. Archive containing the detailed description of each identified T6SS locus as an HTML file. [file 1471-2164-10-104-S7.tgz › LociHTML/HTML/AP008229B.html]

Locus AP008229B on Xanthomonas oryzae (pathovar oryzae, strain MAFF 311018) chromosome, complete sequence.

import namespace="svg" implementation="#AdobeSVG"?


# Locus AP008229B

# List of CDS in T6SS locus AP008229B

|  |  |  |  |  |  |  |  |  |
| --- | --- | --- | --- | --- | --- | --- | --- | --- |
| Name | from | to | direct | COG | e-value | COG cover | COG hit start | COG hit end |
| AP008229\_XOO2882 | 3252720 | 3253499 | True | COG0745 | 2e-47 | 98.0 | 2 | 227 |
| AP008229\_XOO2883 | 3253496 | 3254488 | True | COG1858 | 7e-49 | 94.0 | 20 | 362 |
| AP008229\_XOO2884 | 3254485 | 3256944 | True | COG0642 | 5e-28 | 88.0 | 38 | 336 |
| AP008229\_XOO2885 | 3257058 | 3258038 | True | COG0583 | 2e-16 | 99.0 | 2 | 297 |
| AP008229\_XOO2886 | 3258047 | 3259075 | True | COG3515 | 1e-14 | 96.0 | 7 | 341 |
| AP008229\_XOO2887 | 3259248 | 3259574 | False | - | - | - | - | - |
| AP008229\_XOO2888 | 3259571 | 3262474 | False | COG0515 | 5e-35 | 72.0 | 2 | 278 |
| AP008229\_XOO2888 | 3259571 | 3262474 | False | COG1262 | 4e-09 | 61.0 | 86 | 277 |
| AP008229\_XOO2889 | 3262471 | 3263193 | False | COG0631 | 2e-35 | 94.0 | 4 | 251 |
| AP008229\_XOO2890 | 3263190 | 3263837 | False | COG3913 | 1e-13 | 46.0 | 1 | 106 |
| AP008229\_XOO2891 | 3263834 | 3267292 | False | COG3523 | 0.0 | 99.0 | 3 | 1184 |
| AP008229\_XOO2892 | 3267296 | 3268612 | False | COG3455 | 3e-49 | 96.0 | 7 | 259 |
| AP008229\_XOO2892 | 3267296 | 3268612 | False | COG1360 | 5e-20 | 56.0 | 108 | 244 |
| AP008229\_XOO2893 | 3268614 | 3269951 | False | COG3522 | 3e-131 | 100.0 | 1 | 446 |
| AP008229\_XOO2894 | 3269948 | 3271339 | False | COG3456 | 2e-39 | 99.0 | 1 | 426 |
| AP008229\_XOO2895 | 3271336 | 3271875 | False | - | - | - | - | - |
| AP008229\_XOO2896 | 3271884 | 3273932 | False | COG3501 | 9e-127 | 96.0 | 10 | 539 |
| AP008229\_XOO2897 | 3274086 | 3274544 | False | - | - | - | - | - |
| AP008229\_XOO2898 | 3274619 | 3274870 | False | - | - | - | - | - |
| AP008229\_XOO2899 | 3276177 | 3278882 | False | COG0542 | 0.0 | 99.0 | 1 | 783 |
| AP008229\_XOO2900 | 3278915 | 3279925 | False | COG3520 | 1e-72 | 99.0 | 1 | 332 |
| AP008229\_XOO2901 | 3279889 | 3281766 | False | COG3519 | 4e-170 | 100.0 | 1 | 621 |
| AP008229\_XOO2902 | 3281770 | 3282273 | False | COG3518 | 1e-21 | 98.0 | 1 | 154 |
| AP008229\_XOO2903 | 3282261 | 3283094 | False | COG4455 | 7e-49 | 94.0 | 9 | 265 |
| AP008229\_XOO2904 | 3283130 | 3283633 | False | COG3157 | 2e-22 | 93.0 | 1 | 151 |
| AP008229\_XOO2905 | 3283733 | 3285304 | False | COG3517 | 0.0 | 100.0 | 1 | 495 |
| AP008229\_XOO2906 | 3285240 | 3285875 | False | COG3516 | 5e-53 | 97.0 | 2 | 166 |
| AP008229\_XOO2907 | 3286268 | 3286909 | False | - | - | - | - | - |
| AP008229\_XOO2908 | 3287105 | 3288073 | False | COG3039 | 3e-22 | 99.0 | 1 | 229 |
| AP008229\_XOO2909 | 3288209 | 3288595 | True | COG5659 | 2e-08 | 30.0 | 10 | 127 |
| AP008229\_XOO2910 | 3288576 | 3289169 | True | COG5659 | 2e-16 | 41.0 | 223 | 383 |
| AP008229\_XOO2911 | 3289340 | 3290575 | True | COG3464 | 4e-28 | 98.0 | 5 | 400 |
| AP008229\_XOO2912 | 3290761 | 3291729 | False | COG3039 | 1e-22 | 99.0 | 1 | 229 |
