# Supplementary material for: Dissecting the bacterial type VI secretion system by a genome wide in silico analysis: what can be learned from available microbial genomic resources?
Source: BMC Genomics. 2009 Mar 12;10:104. doi: 10.1186/1471-2164-10-104 (PMC2660368; doi:10.1186/1471-2164-10-104)
Supplement: Additional file 7 — Detailed description of all identified T6SS gene clusters. Archive containing the detailed description of each identified T6SS locus as an HTML file. [file 1471-2164-10-104-S7.tgz › LociHTML/HTML/AP008229C.html]

Locus AP008229C on Xanthomonas oryzae (pathovar oryzae, strain MAFF 311018) chromosome, complete sequence.

import namespace="svg" implementation="#AdobeSVG"?


# Locus AP008229C

# List of CDS in T6SS locus AP008229C

|  |  |  |  |  |  |  |  |  |
| --- | --- | --- | --- | --- | --- | --- | --- | --- |
| Name | from | to | direct | COG | e-value | COG cover | COG hit start | COG hit end |
| AP008229\_XOO3282 | 3728990 | 3730960 | False | - | - | - | - | - |
| AP008229\_XOO3283 | 3730945 | 3731970 | False | - | - | - | - | - |
| AP008229\_XOO3284 | 3731974 | 3734841 | False | - | - | - | - | - |
| AP008229\_XOO3285 | 3734860 | 3735765 | False | - | - | - | - | - |
| AP008229\_XOO3286 | 3735707 | 3738469 | False | COG4253 | 5e-21 | 79.0 | 1 | 221 |
| AP008229\_XOO3286 | 3735707 | 3738469 | False | COG3501 | 1e-100 | 94.0 | 1 | 522 |
| AP008229\_XOO3287 | 3738563 | 3738916 | False | - | - | - | - | - |
| AP008229\_XOO3288 | 3738947 | 3741676 | False | COG0542 | 0.0 | 98.0 | 1 | 778 |
| AP008229\_XOO3289 | 3741762 | 3742853 | False | COG3520 | 3e-63 | 98.0 | 1 | 331 |
| AP008229\_XOO3290 | 3742817 | 3744652 | False | COG3519 | 5e-151 | 99.0 | 2 | 620 |
| AP008229\_XOO3291 | 3744655 | 3745143 | False | COG3518 | 2e-28 | 96.0 | 4 | 155 |
| AP008229\_XOO3292 | 3745291 | 3745788 | False | COG3157 | 2e-23 | 89.0 | 6 | 150 |
| AP008229\_XOO3293 | 3745930 | 3747426 | False | COG3517 | 0.0 | 99.0 | 1 | 491 |
| AP008229\_XOO3294 | 3747430 | 3747930 | False | COG3516 | 3e-47 | 94.0 | 2 | 160 |
| AP008229\_XOO3295 | 3747977 | 3748591 | False | - | - | - | - | - |
| AP008229\_XOO3296 | 3748853 | 3749461 | True | COG3521 | 4e-18 | 86.0 | 1 | 137 |
| AP008229\_XOO3297 | 3749613 | 3750947 | True | COG3522 | 4e-104 | 98.0 | 6 | 446 |
| AP008229\_XOO3298 | 3750947 | 3751735 | True | COG3455 | 4e-42 | 93.0 | 13 | 258 |
| AP008229\_XOO3299 | 3751746 | 3754463 | True | COG4253 | 3e-36 | 94.0 | 1 | 263 |
| AP008229\_XOO3299 | 3751746 | 3754463 | True | COG3501 | 2e-94 | 96.0 | 2 | 530 |
| AP008229\_XOO3300 | 3755996 | 3757966 | True | COG4253 | 2e-19 | 56.0 | 1 | 158 |
| AP008229\_XOO3300 | 3755996 | 3757966 | True | COG3501 | 1e-63 | 74.0 | 124 | 530 |
| AP008229\_XOO3301 | 3758372 | 3760603 | False | - | - | - | - | - |
| AP008229\_XOO3302 | 3760603 | 3761199 | False | - | - | - | - | - |
| AP008229\_XOO3303 | 3761192 | 3761785 | False | - | - | - | - | - |
| AP008229\_XOO3304 | 3762012 | 3764681 | False | COG4253 | 1e-22 | 86.0 | 1 | 240 |
| AP008229\_XOO3304 | 3762012 | 3764681 | False | COG3501 | 2e-63 | 77.0 | 124 | 550 |
| AP008229\_XOO3305 | 3764867 | 3765829 | True | COG1262 | 2e-25 | 67.0 | 43 | 254 |
| AP008229\_XOO3306 | 3766597 | 3768948 | False | - | - | - | - | - |
| AP008229\_XOO3307 | 3768935 | 3769480 | False | COG4253 | 3e-16 | 52.0 | 122 | 266 |
| AP008229\_XOO3308 | 3769562 | 3770884 | False | - | - | - | - | - |
| AP008229\_XOO3309 | 3771391 | 3771870 | False | - | - | - | - | - |
| AP008229\_XOO3310 | 3771894 | 3772358 | False | - | - | - | - | - |
| AP008229\_XOO3311 | 3772675 | 3773154 | False | - | - | - | - | - |
| AP008229\_XOO3312 | 3773178 | 3773642 | False | - | - | - | - | - |
| AP008229\_XOO3313 | 3773639 | 3774535 | False | - | - | - | - | - |
| AP008229\_XOO3314 | 3774535 | 3777351 | False | COG4253 | 2e-23 | 79.0 | 1 | 221 |
| AP008229\_XOO3314 | 3774535 | 3777351 | False | COG3501 | 5e-90 | 99.0 | 3 | 550 |
| AP008229\_XOO3315 | 3777472 | 3777690 | False | - | - | - | - | - |
| AP008229\_XOO3316 | 3778301 | 3779374 | True | COG3515 | 1e-29 | 100.0 | 1 | 346 |
| AP008229\_XOO3317 | 3779427 | 3780227 | False | COG2885 | 2e-25 | 55.0 | 82 | 187 |
| AP008229\_XOO3318 | 3780230 | 3781213 | False | COG3913 | 3e-22 | 92.0 | 8 | 216 |
| AP008229\_XOO3319 | 3781210 | 3784902 | False | COG3523 | 0.0 | 98.0 | 17 | 1188 |
| AP008229\_XOO3320 | 3784963 | 3786450 | False | - | - | - | - | - |
| AP008229\_XOO3321 | 3786504 | 3786764 | False | COG4104 | 4e-15 | 89.0 | 10 | 97 |
| AP008229\_XOO3322 | 3786874 | 3787752 | False | - | - | - | - | - |
| AP008229\_XOO3323 | 3787952 | 3788692 | True | - | - | - | - | - |
| AP008229\_XOO3324 | 3788973 | 3789509 | True | - | - | - | - | - |
| AP008229\_XOO3325 | 3789709 | 3790911 | False | COG0027 | 3e-164 | 100.0 | 1 | 394 |
