# Supplementary material for: Dissecting the bacterial type VI secretion system by a genome wide in silico analysis: what can be learned from available microbial genomic resources?
Source: BMC Genomics. 2009 Mar 12;10:104. doi: 10.1186/1471-2164-10-104 (PMC2660368; doi:10.1186/1471-2164-10-104)
Supplement: Additional file 7 — Detailed description of all identified T6SS gene clusters. Archive containing the detailed description of each identified T6SS locus as an HTML file. [file 1471-2164-10-104-S7.tgz › LociHTML/HTML/BA000007A.html]

Locus BA000007A on Escherichia coli (strain Sakai / O157:H7 / RIMD 0509952 / EHEC) chromosome, complete sequence.

import namespace="svg" implementation="#AdobeSVG"?


# Locus BA000007A

# List of CDS in T6SS locus BA000007A

|  |  |  |  |  |  |  |  |  |
| --- | --- | --- | --- | --- | --- | --- | --- | --- |
| Name | from | to | direct | COG | e-value | COG cover | COG hit start | COG hit end |
| BA000007\_ECs0209 | 238348 | 239088 | True | COG2226 | 1e-07 | 26.0 | 106 | 168 |
| BA000007\_ECs0210 | 239085 | 239552 | False | COG0328 | 1e-55 | 99.0 | 2 | 154 |
| BA000007\_ECs0211 | 239617 | 240348 | True | COG0847 | 5e-51 | 95.0 | 8 | 240 |
| BA000007\_ECs0212 | 241871 | 242167 | False | - | - | - | - | - |
| BA000007\_ECs0213 | 242164 | 242613 | False | - | - | - | - | - |
| BA000007\_ECs0214 | 242616 | 243263 | False | - | - | - | - | - |
| BA000007\_ECs0215 | 243291 | 243512 | False | - | - | - | - | - |
| BA000007\_ECs0216 | 243533 | 244012 | False | COG3157 | 4e-43 | 98.0 | 1 | 159 |
| BA000007\_ECs0217 | 243978 | 245387 | False | COG3515 | 9e-59 | 96.0 | 12 | 346 |
| BA000007\_ECs0218 | 245398 | 248505 | False | COG3523 | 0.0 | 89.0 | 129 | 1188 |
| BA000007\_ECs0219 | 248762 | 248950 | False | - | - | - | - | - |
| BA000007\_ECs0220 | 248969 | 249763 | False | COG3515 | 3e-36 | 76.0 | 19 | 284 |
| BA000007\_ECs0221 | 249760 | 250380 | False | - | - | - | - | - |
| BA000007\_ECs0222 | 250385 | 251128 | False | - | - | - | - | - |
| BA000007\_ECs0223 | 251125 | 253896 | False | COG0542 | 1e-122 | 53.0 | 1 | 423 |
| BA000007\_ECs0223 | 251125 | 253896 | False | COG0542 | 7e-98 | 46.0 | 422 | 786 |
| BA000007\_ECs0224 | 253905 | 254666 | False | COG3455 | 8e-83 | 97.0 | 6 | 260 |
| BA000007\_ECs0225 | 254671 | 256002 | False | COG3522 | 6e-164 | 100.0 | 1 | 446 |
| BA000007\_ECs0226 | 256005 | 256529 | False | COG3521 | 5e-40 | 100.0 | 1 | 159 |
| BA000007\_ECs0227 | 256526 | 257806 | False | COG3456 | 1e-119 | 100.0 | 1 | 430 |
| BA000007\_ECs0228 | 257831 | 258913 | False | COG3520 | 5e-104 | 99.0 | 1 | 332 |
| BA000007\_ECs0229 | 258877 | 260727 | False | COG3519 | 0.0 | 99.0 | 2 | 621 |
| BA000007\_ECs0230 | 260731 | 261144 | False | COG3518 | 2e-26 | 96.0 | 3 | 154 |
| BA000007\_ECs0231 | 261235 | 262626 | False | COG3517 | 0.0 | 93.0 | 1 | 465 |
| BA000007\_ECs0232 | 262677 | 262901 | False | - | - | - | - | - |
| BA000007\_ECs0233 | 262936 | 263436 | False | COG3516 | 2e-49 | 98.0 | 2 | 167 |
| BA000007\_ECs0234 | 264133 | 264651 | True | COG3157 | 9e-52 | 98.0 | 1 | 160 |
| BA000007\_ECs0235 | 264684 | 264821 | True | - | - | - | - | - |
| BA000007\_ECs0236 | 264861 | 267002 | True | COG3501 | 2e-174 | 98.0 | 8 | 550 |
| BA000007\_ECs0237 | 267078 | 271292 | True | COG3209 | 2e-104 | 98.0 | 1 | 786 |
| BA000007\_ECs0237 | 267078 | 271292 | True | COG3209 | 3e-19 | 56.0 | 12 | 463 |
| BA000007\_ECs0238 | 271295 | 271906 | True | - | - | - | - | - |
| BA000007\_ECs0239 | 271928 | 272110 | True | - | - | - | - | - |
