# Supplementary material for: Dissecting the bacterial type VI secretion system by a genome wide in silico analysis: what can be learned from available microbial genomic resources?
Source: BMC Genomics. 2009 Mar 12;10:104. doi: 10.1186/1471-2164-10-104 (PMC2660368; doi:10.1186/1471-2164-10-104)
Supplement: Additional file 7 — Detailed description of all identified T6SS gene clusters. Archive containing the detailed description of each identified T6SS locus as an HTML file. [file 1471-2164-10-104-S7.tgz › LociHTML/HTML/BA000012A.html]

Locus BA000012A on Rhizobium loti (strain MAFF303099) chromosome, complete sequence.

import namespace="svg" implementation="#AdobeSVG"?


# Locus BA000012A

# List of CDS in T6SS locus BA000012A

|  |  |  |  |  |  |  |  |  |
| --- | --- | --- | --- | --- | --- | --- | --- | --- |
| Name | from | to | direct | COG | e-value | COG cover | COG hit start | COG hit end |
| BA000012\_mlr2327 | 1876685 | 1877644 | True | COG1175 | 1e-62 | 98.0 | 4 | 295 |
| BA000012\_mlr2328 | 1877641 | 1878531 | True | COG0395 | 4e-64 | 100.0 | 1 | 281 |
| BA000012\_mlr2329 | 1878542 | 1879639 | True | COG3839 | 5e-119 | 100.0 | 1 | 338 |
| BA000012\_mlr2330 | 1879666 | 1880829 | True | COG4948 | 4e-76 | 99.0 | 2 | 372 |
| BA000012\_mlr2332 | 1880853 | 1881602 | True | COG0684 | 4e-53 | 100.0 | 1 | 210 |
| BA000012\_mll2333 | 1881611 | 1882051 | False | - | - | - | - | - |
| BA000012\_msr2334 | 1882032 | 1882193 | True | - | - | - | - | - |
| BA000012\_mll2335 | 1882260 | 1885097 | False | COG0542 | 6e-126 | 61.0 | 1 | 480 |
| BA000012\_mll2335 | 1882260 | 1885097 | False | COG0542 | 6e-98 | 49.0 | 396 | 786 |
| BA000012\_mlr2336 | 1885421 | 1886596 | True | COG3515 | 3e-45 | 100.0 | 1 | 346 |
| BA000012\_mlr2337 | 1886602 | 1887138 | True | COG3516 | 4e-49 | 100.0 | 1 | 169 |
| BA000012\_mlr2338 | 1887142 | 1888647 | True | COG3517 | 0.0 | 100.0 | 1 | 495 |
| BA000012\_mlr2339 | 1888697 | 1889179 | True | COG3157 | 2e-35 | 95.0 | 3 | 157 |
| BA000012\_mlr2341 | 1889166 | 1889918 | True | COG3518 | 9e-27 | 100.0 | 1 | 157 |
| BA000012\_mlr2342 | 1889920 | 1891794 | True | COG3519 | 0.0 | 100.0 | 1 | 621 |
| BA000012\_mlr2343 | 1891758 | 1892825 | True | COG3520 | 7e-82 | 99.0 | 2 | 335 |
| BA000012\_mlr2345 | 1892822 | 1894282 | True | COG3456 | 3e-103 | 98.0 | 3 | 424 |
| BA000012\_mlr2346 | 1894286 | 1894738 | True | COG3521 | 3e-40 | 96.0 | 1 | 154 |
| BA000012\_mlr2347 | 1894779 | 1896113 | True | COG3522 | 1e-139 | 100.0 | 1 | 446 |
| BA000012\_mlr2348 | 1896118 | 1897446 | True | COG3455 | 3e-66 | 99.0 | 1 | 261 |
| BA000012\_mlr2348 | 1896118 | 1897446 | True | COG1360 | 3e-23 | 53.0 | 113 | 243 |
| BA000012\_mlr2349 | 1897448 | 1900990 | True | COG3523 | 0.0 | 99.0 | 1 | 1187 |
| BA000012\_mlr2350 | 1900972 | 1901514 | True | COG3913 | 5e-52 | 96.0 | 1 | 219 |
| BA000012\_mlr2351 | 1901514 | 1902617 | True | COG5351 | 5e-164 | 100.0 | 1 | 367 |
| BA000012\_mlr2352 | 1902614 | 1903657 | True | COG0304 | 4e-35 | 90.0 | 34 | 407 |
| BA000012\_mlr2353 | 1903654 | 1904703 | True | - | - | - | - | - |
| BA000012\_mlr2354 | 1904700 | 1905761 | True | - | - | - | - | - |
| BA000012\_mll2355 | 1905788 | 1906126 | False | - | - | - | - | - |
| BA000012\_mll2356 | 1906126 | 1906548 | False | - | - | - | - | - |
| BA000012\_mll2357 | 1906557 | 1908884 | False | COG3501 | 4e-172 | 98.0 | 7 | 546 |
| BA000012\_mlr2358 | 1909132 | 1910211 | True | COG3456 | 3e-91 | 99.0 | 1 | 427 |
| BA000012\_mlr2359 | 1910475 | 1911989 | True | COG3455 | 4e-69 | 100.0 | 1 | 262 |
| BA000012\_mlr2359 | 1910475 | 1911989 | True | COG1360 | 3e-27 | 56.0 | 102 | 240 |
| BA000012\_mlr2360 | 1911989 | 1915519 | True | COG3523 | 0.0 | 99.0 | 8 | 1187 |
| BA000012\_mlr2361 | 1915592 | 1916434 | True | COG0631 | 7e-58 | 99.0 | 2 | 261 |
| BA000012\_mlr2363 | 1916431 | 1919004 | True | COG0515 | 8e-33 | 94.0 | 2 | 365 |
| BA000012\_mlr2364 | 1919017 | 1919517 | True | COG5480 | 9e-54 | 98.0 | 3 | 147 |
| BA000012\_mlr2365 | 1919514 | 1921952 | True | COG0739 | 8e-32 | 41.0 | 143 | 258 |
