# Supplementary material for: Dissecting the bacterial type VI secretion system by a genome wide in silico analysis: what can be learned from available microbial genomic resources?
Source: BMC Genomics. 2009 Mar 12;10:104. doi: 10.1186/1471-2164-10-104 (PMC2660368; doi:10.1186/1471-2164-10-104)
Supplement: Additional file 7 — Detailed description of all identified T6SS gene clusters. Archive containing the detailed description of each identified T6SS locus as an HTML file. [file 1471-2164-10-104-S7.tgz › LociHTML/HTML/BA000031B.html]

Locus BA000031B on Vibrio parahaemolyticus (serovar O3:K6, strain RIMD 2210633) chromosome 1, complete sequence.

import namespace="svg" implementation="#AdobeSVG"?


# Locus BA000031B

# List of CDS in T6SS locus BA000031B

|  |  |  |  |  |  |  |  |  |
| --- | --- | --- | --- | --- | --- | --- | --- | --- |
| Name | from | to | direct | COG | e-value | COG cover | COG hit start | COG hit end |
| BA000031\_VP1390 | 1486402 | 1491144 | True | COG2885 | 1e-12 | 72.0 | 50 | 187 |
| BA000031\_VP1391 | 1491226 | 1492857 | False | COG3604 | 3e-103 | 93.0 | 34 | 546 |
| BA000031\_VP1392 | 1492844 | 1495516 | False | COG0542 | 0.0 | 98.0 | 1 | 778 |
| BA000031\_VP1393 | 1495971 | 1496489 | True | COG3157 | 2e-38 | 98.0 | 1 | 160 |
| BA000031\_VP1394 | 1496562 | 1498640 | True | COG3501 | 1e-141 | 98.0 | 8 | 549 |
| BA000031\_VP1395 | 1498640 | 1499125 | True | - | - | - | - | - |
| BA000031\_VP1396 | 1499195 | 1499968 | True | - | - | - | - | - |
| BA000031\_VP1397 | 1499937 | 1500536 | True | - | - | - | - | - |
| BA000031\_VP1398 | 1500517 | 1501509 | True | COG5351 | 2e-33 | 87.0 | 1 | 322 |
| BA000031\_VP1399 | 1501502 | 1502446 | True | - | - | - | - | - |
| BA000031\_VP1400 | 1502624 | 1504684 | True | COG0515 | 1e-14 | 90.0 | 37 | 383 |
| BA000031\_VP1401 | 1504684 | 1506258 | True | COG3515 | 2e-09 | 57.0 | 7 | 206 |
| BA000031\_VP1402 | 1506285 | 1506791 | True | COG3516 | 4e-41 | 94.0 | 8 | 167 |
| BA000031\_VP1403 | 1506800 | 1508275 | True | COG3517 | 0.0 | 99.0 | 1 | 492 |
| BA000031\_VP1404 | 1508283 | 1508747 | True | COG3518 | 1e-14 | 86.0 | 14 | 149 |
| BA000031\_VP1405 | 1508758 | 1510506 | True | COG3519 | 8e-134 | 99.0 | 7 | 621 |
| BA000031\_VP1406 | 1510470 | 1511501 | True | COG3520 | 2e-63 | 98.0 | 1 | 331 |
| BA000031\_VP1407 | 1511565 | 1512017 | False | COG1522 | 3e-22 | 96.0 | 1 | 149 |
| BA000031\_VP1408 | 1512050 | 1515439 | False | COG3523 | 2e-108 | 98.0 | 12 | 1187 |
| BA000031\_VP1409 | 1515461 | 1516771 | False | COG3515 | 5e-11 | 62.0 | 6 | 221 |
| BA000031\_VP1410 | 1517264 | 1517875 | True | - | - | - | - | - |
| BA000031\_VP1411 | 1517885 | 1519399 | True | COG3456 | 5e-17 | 91.0 | 25 | 419 |
| BA000031\_VP1412 | 1519392 | 1519892 | True | COG3521 | 8e-21 | 88.0 | 6 | 145 |
| BA000031\_VP1413 | 1519904 | 1521229 | True | COG3522 | 2e-121 | 99.0 | 1 | 445 |
| BA000031\_VP1414 | 1521211 | 1522017 | True | COG3455 | 1e-36 | 97.0 | 1 | 256 |
| BA000031\_VP1415 | 1522119 | 1524323 | True | - | - | - | - | - |
| BA000031\_VP1416 | 1524323 | 1524916 | True | - | - | - | - | - |
| BA000031\_VP1417 | 1524886 | 1525467 | True | - | - | - | - | - |
| BA000031\_VP1418 | 1525615 | 1526049 | True | - | - | - | - | - |
| BA000031\_VP1419 | 1526430 | 1526996 | True | - | - | - | - | - |
