# Supplementary material for: Dissecting the bacterial type VI secretion system by a genome wide in silico analysis: what can be learned from available microbial genomic resources?
Source: BMC Genomics. 2009 Mar 12;10:104. doi: 10.1186/1471-2164-10-104 (PMC2660368; doi:10.1186/1471-2164-10-104)
Supplement: Additional file 7 — Detailed description of all identified T6SS gene clusters. Archive containing the detailed description of each identified T6SS locus as an HTML file. [file 1471-2164-10-104-S7.tgz › LociHTML/HTML/BA000032D.html]

Locus BA000032D on Vibrio parahaemolyticus (serovar O3:K6, strain RIMD 2210633) chromosome 2, complete sequence.

import namespace="svg" implementation="#AdobeSVG"?


# Locus BA000032D

# List of CDS in T6SS locus BA000032D

|  |  |  |  |  |  |  |  |  |
| --- | --- | --- | --- | --- | --- | --- | --- | --- |
| Name | from | to | direct | COG | e-value | COG cover | COG hit start | COG hit end |
| BA000032\_VPA1022 | 1074208 | 1077264 | False | COG0419 | 6e-69 | 100.0 | 1 | 908 |
| BA000032\_VPA1023 | 1077274 | 1078407 | False | COG0420 | 5e-51 | 96.0 | 1 | 375 |
| BA000032\_VPA1024 | 1078504 | 1079346 | False | - | - | - | - | - |
| BA000032\_VPA1025 | 1079347 | 1079637 | False | COG4104 | 2e-13 | 96.0 | 4 | 98 |
| BA000032\_VPA1026 | 1079651 | 1081486 | False | COG3501 | 5e-113 | 98.0 | 6 | 548 |
| BA000032\_VPA1027 | 1081544 | 1082023 | False | COG3157 | 9e-15 | 92.0 | 1 | 150 |
| BA000032\_VPA1028 | 1082229 | 1084796 | False | COG0542 | 0.0 | 98.0 | 1 | 776 |
| BA000032\_VPA1029 | 1084807 | 1085772 | False | COG3520 | 4e-38 | 91.0 | 23 | 328 |
| BA000032\_VPA1030 | 1085769 | 1087592 | False | COG3519 | 1e-105 | 100.0 | 1 | 621 |
| BA000032\_VPA1031 | 1087589 | 1088053 | False | COG3518 | 3e-16 | 92.0 | 10 | 155 |
| BA000032\_VPA1032 | 1088062 | 1088859 | False | COG4455 | 6e-36 | 92.0 | 12 | 263 |
| BA000032\_VPA1033 | 1088870 | 1090390 | False | COG3517 | 3e-118 | 96.0 | 17 | 493 |
| BA000032\_VPA1034 | 1090437 | 1091930 | False | COG3517 | 0.0 | 96.0 | 17 | 492 |
| BA000032\_VPA1035 | 1091930 | 1092460 | False | COG3516 | 5e-48 | 100.0 | 1 | 169 |
| BA000032\_VPA1036 | 1092476 | 1093597 | False | COG3515 | 3e-18 | 99.0 | 4 | 346 |
| BA000032\_VPA1037 | 1093594 | 1094382 | False | COG0631 | 1e-51 | 94.0 | 8 | 255 |
| BA000032\_VPA1038 | 1094393 | 1095088 | False | COG3913 | 9e-15 | 95.0 | 1 | 217 |
| BA000032\_VPA1039 | 1095070 | 1098588 | False | COG3523 | 0.0 | 99.0 | 7 | 1187 |
| BA000032\_VPA1040 | 1098604 | 1099905 | False | COG3455 | 7e-43 | 98.0 | 1 | 259 |
| BA000032\_VPA1040 | 1098604 | 1099905 | False | COG1360 | 9e-27 | 62.0 | 86 | 238 |
| BA000032\_VPA1041 | 1099908 | 1101233 | False | COG3522 | 9e-98 | 100.0 | 1 | 446 |
| BA000032\_VPA1042 | 1101255 | 1101710 | False | COG3521 | 2e-31 | 91.0 | 1 | 145 |
| BA000032\_VPA1043 | 1101720 | 1102916 | False | COG3456 | 3e-41 | 99.0 | 1 | 427 |
| BA000032\_VPA1044 | 1103236 | 1105380 | True | COG0515 | 3e-25 | 63.0 | 2 | 243 |
| BA000032\_VPA1045 | 1105492 | 1107027 | True | - | - | - | - | - |
| BA000032\_VPA1046 | 1107024 | 1107365 | True | - | - | - | - | - |
| BA000032\_VPA1047 | 1107457 | 1107588 | False | - | - | - | - | - |
| BA000032\_VPA1048 | 1107620 | 1108114 | False | COG2172 | 7e-08 | 84.0 | 1 | 124 |
