# Supplementary material for: Dissecting the bacterial type VI secretion system by a genome wide in silico analysis: what can be learned from available microbial genomic resources?
Source: BMC Genomics. 2009 Mar 12;10:104. doi: 10.1186/1471-2164-10-104 (PMC2660368; doi:10.1186/1471-2164-10-104)
Supplement: Additional file 7 — Detailed description of all identified T6SS gene clusters. Archive containing the detailed description of each identified T6SS locus as an HTML file. [file 1471-2164-10-104-S7.tgz › LociHTML/HTML/BA000038C.html]

Locus BA000038C on Vibrio vulnificus (strain YJ016) chromosome II, complete sequence.

import namespace="svg" implementation="#AdobeSVG"?


# Locus BA000038C

# List of CDS in T6SS locus BA000038C

|  |  |  |  |  |  |  |  |  |
| --- | --- | --- | --- | --- | --- | --- | --- | --- |
| Name | from | to | direct | COG | e-value | COG cover | COG hit start | COG hit end |
| BA000038\_VVA0976 | 1081034 | 1085530 | False | COG3209 | 6e-38 | 88.0 | 1 | 708 |
| BA000038\_VVA0977 | 1085531 | 1086439 | False | - | - | - | - | - |
| BA000038\_VVA0978 | 1086740 | 1088575 | False | COG3501 | 2e-106 | 98.0 | 6 | 549 |
| BA000038\_VVA0979 | 1088630 | 1089109 | False | COG3157 | 1e-14 | 92.0 | 1 | 150 |
| BA000038\_VVA0980 | 1089325 | 1091889 | False | COG0542 | 0.0 | 99.0 | 1 | 784 |
| BA000038\_VVA0981 | 1091903 | 1092904 | False | COG3520 | 2e-36 | 90.0 | 16 | 317 |
| BA000038\_VVA0982 | 1092868 | 1094694 | False | COG3519 | 9e-100 | 100.0 | 1 | 621 |
| BA000038\_VVA0983 | 1094691 | 1095164 | False | COG3518 | 6e-16 | 95.0 | 6 | 155 |
| BA000038\_VVA0984 | 1095161 | 1095964 | False | COG4455 | 9e-31 | 91.0 | 12 | 261 |
| BA000038\_VVA0985 | 1095975 | 1097582 | False | COG3517 | 2e-120 | 86.0 | 67 | 493 |
| BA000038\_VVA0986 | 1097513 | 1099006 | False | COG3517 | 0.0 | 98.0 | 6 | 492 |
| BA000038\_VVA0987 | 1099018 | 1099530 | False | COG3516 | 6e-48 | 97.0 | 1 | 165 |
| BA000038\_VVA0988 | 1099547 | 1100680 | False | COG3515 | 3e-15 | 97.0 | 8 | 344 |
| BA000038\_VVA0989 | 1100680 | 1101552 | False | COG0631 | 3e-51 | 95.0 | 7 | 256 |
| BA000038\_VVA0990 | 1101485 | 1102180 | False | COG3913 | 3e-13 | 96.0 | 1 | 218 |
| BA000038\_VVA0991 | 1102162 | 1105686 | False | COG3523 | 0.0 | 99.0 | 5 | 1187 |
| BA000038\_VVA0992 | 1105664 | 1106992 | False | COG1360 | 6e-26 | 86.0 | 34 | 244 |
| BA000038\_VVA0992 | 1105664 | 1106992 | False | COG3455 | 5e-45 | 85.0 | 39 | 261 |
| BA000038\_VVA0993 | 1107001 | 1108323 | False | COG3522 | 1e-105 | 100.0 | 1 | 446 |
| BA000038\_VVA0994 | 1108347 | 1108802 | False | COG3521 | 9e-26 | 81.0 | 16 | 145 |
| BA000038\_VVA0995 | 1108803 | 1110107 | False | COG3456 | 3e-45 | 100.0 | 1 | 430 |
| BA000038\_VVA0996 | 1110336 | 1112504 | True | COG0515 | 1e-25 | 96.0 | 2 | 372 |
| BA000038\_VVA0997 | 1112568 | 1113185 | False | COG0625 | 2e-29 | 95.0 | 1 | 201 |
| BA000038\_VVA0998 | 1113286 | 1113873 | True | COG1309 | 2e-07 | 91.0 | 6 | 188 |
| BA000038\_VVA0999 | 1113870 | 1114265 | True | COG1661 | 3e-19 | 95.0 | 7 | 141 |
| BA000038\_VVA1000 | 1114272 | 1114535 | False | COG3530 | 2e-23 | 100.0 | 1 | 71 |
| BA000038\_VVA1001 | 1114568 | 1114873 | True | - | - | - | - | - |
| BA000038\_VVA1002 | 1114893 | 1115237 | False | - | - | - | - | - |
