# Supplementary material for: Dissecting the bacterial type VI secretion system by a genome wide in silico analysis: what can be learned from available microbial genomic resources?
Source: BMC Genomics. 2009 Mar 12;10:104. doi: 10.1186/1471-2164-10-104 (PMC2660368; doi:10.1186/1471-2164-10-104)
Supplement: Additional file 7 — Detailed description of all identified T6SS gene clusters. Archive containing the detailed description of each identified T6SS locus as an HTML file. [file 1471-2164-10-104-S7.tgz › LociHTML/HTML/BA000040A.html]

Locus BA000040A on Bradyrhizobium japonicum (strain USDA 110) chromosome, complete sequence.

import namespace="svg" implementation="#AdobeSVG"?


# Locus BA000040A

# List of CDS in T6SS locus BA000040A

|  |  |  |  |  |  |  |  |  |
| --- | --- | --- | --- | --- | --- | --- | --- | --- |
| Name | from | to | direct | COG | e-value | COG cover | COG hit start | COG hit end |
| BA000040\_bll3583 | 3957252 | 3958016 | False | - | - | - | - | - |
| BA000040\_bll6701 | 3958210 | 3959556 | True | - | - | - | - | - |
| BA000040\_blr3585 | 3959846 | 3960496 | True | - | - | - | - | - |
| BA000040\_bll3586 | 3960586 | 3962187 | False | COG4797 | 3e-13 | 84.0 | 1 | 227 |
| BA000040\_bll3586 | 3960586 | 3962187 | False | COG0220 | 3e-09 | 38.0 | 27 | 114 |
| BA000040\_bll3586 | 3960586 | 3962187 | False | COG4123 | 6e-07 | 70.0 | 40 | 214 |
| BA000040\_bll3587 | 3962178 | 3964817 | False | COG0542 | 0.0 | 99.0 | 1 | 782 |
| BA000040\_bll3588 | 3964915 | 3965979 | False | COG3520 | 3e-60 | 88.0 | 19 | 316 |
| BA000040\_bll3589 | 3965943 | 3966599 | False | COG3519 | 3e-40 | 33.0 | 417 | 621 |
| BA000040\_bll3590 | 3966580 | 3967911 | False | COG3519 | 1e-68 | 66.0 | 1 | 411 |
| BA000040\_bll3591 | 3967913 | 3968461 | False | COG3518 | 7e-10 | 92.0 | 11 | 155 |
| BA000040\_bll3592 | 3968644 | 3969129 | False | COG3157 | 1e-32 | 100.0 | 1 | 162 |
| BA000040\_bll3593 | 3969180 | 3970691 | False | COG3517 | 0.0 | 99.0 | 2 | 494 |
| BA000040\_bll3594 | 3970699 | 3971250 | False | COG3516 | 1e-42 | 98.0 | 3 | 169 |
| BA000040\_bll3595 | 3971366 | 3972706 | False | - | - | - | - | - |
| BA000040\_blr3596 | 3973027 | 3974895 | True | COG3501 | 3e-136 | 96.0 | 5 | 535 |
| BA000040\_blr3597 | 3974901 | 3975464 | True | - | - | - | - | - |
| BA000040\_blr3598 | 3975474 | 3976856 | True | COG3456 | 1e-47 | 99.0 | 1 | 429 |
| BA000040\_blr3599 | 3976893 | 3978242 | True | COG3522 | 2e-107 | 100.0 | 1 | 446 |
| BA000040\_blr3600 | 3978239 | 3979765 | True | COG3455 | 7e-42 | 94.0 | 16 | 262 |
| BA000040\_blr3600 | 3978239 | 3979765 | True | COG1360 | 1e-25 | 59.0 | 99 | 242 |
| BA000040\_blr3601 | 3979809 | 3983345 | True | COG3523 | 0.0 | 98.0 | 19 | 1187 |
| BA000040\_blr3602 | 3983342 | 3984043 | True | COG3913 | 4e-17 | 62.0 | 7 | 149 |
| BA000040\_blr3603 | 3984050 | 3984826 | True | COG0631 | 5e-44 | 95.0 | 1 | 251 |
| BA000040\_blr3604 | 3984823 | 3986631 | True | COG0515 | 1e-34 | 70.0 | 2 | 271 |
| BA000040\_bll3605 | 3986641 | 3987753 | False | - | - | - | - | - |
| BA000040\_bsl3606 | 3987723 | 3988013 | False | - | - | - | - | - |
| BA000040\_blr3607 | 3988164 | 3989885 | True | - | - | - | - | - |
