# Supplementary material for: Dissecting the bacterial type VI secretion system by a genome wide in silico analysis: what can be learned from available microbial genomic resources?
Source: BMC Genomics. 2009 Mar 12;10:104. doi: 10.1186/1471-2164-10-104 (PMC2660368; doi:10.1186/1471-2164-10-104)
Supplement: Additional file 7 — Detailed description of all identified T6SS gene clusters. Archive containing the detailed description of each identified T6SS locus as an HTML file. [file 1471-2164-10-104-S7.tgz › LociHTML/HTML/BX119912C.html]

Locus BX119912C on Rhodopirellula baltica (strain 1) chromosome, complete sequence.

import namespace="svg" implementation="#AdobeSVG"?


# Locus BX119912C

# List of CDS in T6SS locus BX119912C

|  |  |  |  |  |  |  |  |  |
| --- | --- | --- | --- | --- | --- | --- | --- | --- |
| Name | from | to | direct | COG | e-value | COG cover | COG hit start | COG hit end |
| BX119912\_RB9666 | 5209189 | 5209872 | True | - | - | - | - | - |
| BX119912\_RB9668 | 5209887 | 5210069 | False | - | - | - | - | - |
| BX119912\_RB9669 | 5210092 | 5210265 | False | - | - | - | - | - |
| BX119912\_RB9670 | 5210290 | 5211006 | False | - | - | - | - | - |
| BX119912\_RB9673 | 5211126 | 5211281 | True | - | - | - | - | - |
| BX119912\_RB9674 | 5211283 | 5213163 | True | COG2936 | 2e-18 | 85.0 | 51 | 529 |
| BX119912\_RB9676 | 5213215 | 5214558 | False | - | - | - | - | - |
| BX119912\_RB9679 | 5214695 | 5216233 | True | COG3522 | 2e-46 | 96.0 | 2 | 430 |
| BX119912\_RB9684 | 5216230 | 5216877 | True | COG3455 | 2e-13 | 67.0 | 65 | 241 |
| BX119912\_RB9685 | 5216911 | 5218644 | True | - | - | - | - | - |
| BX119912\_RB9687 | 5218795 | 5219868 | True | COG3515 | 5e-18 | 96.0 | 7 | 341 |
| BX119912\_RB9689 | 5219934 | 5220461 | True | COG3516 | 9e-53 | 94.0 | 2 | 161 |
| BX119912\_RB9690 | 5220509 | 5222011 | True | COG3517 | 0.0 | 100.0 | 1 | 495 |
| BX119912\_RB9692 | 5222065 | 5222544 | True | COG3157 | 2e-15 | 98.0 | 4 | 162 |
| BX119912\_RB9693 | 5222556 | 5223110 | True | COG3518 | 5e-10 | 65.0 | 2 | 104 |
| BX119912\_RB9696 | 5223290 | 5225191 | True | COG3519 | 1e-147 | 100.0 | 1 | 621 |
| BX119912\_RB9698 | 5225279 | 5226328 | True | COG3520 | 2e-54 | 90.0 | 15 | 316 |
| BX119912\_RB9699 | 5226252 | 5228345 | True | COG3501 | 7e-129 | 99.0 | 2 | 550 |
| BX119912\_RB9700 | 5228428 | 5229342 | True | - | - | - | - | - |
| BX119912\_RB9702 | 5229342 | 5229632 | True | COG4104 | 2e-08 | 100.0 | 1 | 98 |
| BX119912\_RB9704 | 5229636 | 5234027 | True | - | - | - | - | - |
