# Supplementary material for: Dissecting the bacterial type VI secretion system by a genome wide in silico analysis: what can be learned from available microbial genomic resources?
Source: BMC Genomics. 2009 Mar 12;10:104. doi: 10.1186/1471-2164-10-104 (PMC2660368; doi:10.1186/1471-2164-10-104)
Supplement: Additional file 7 — Detailed description of all identified T6SS gene clusters. Archive containing the detailed description of each identified T6SS locus as an HTML file. [file 1471-2164-10-104-S7.tgz › LociHTML/HTML/BX470249B.html]

Locus BX470249B on Bordetella parapertussis (strain NCTC 13253 / ATCC BAA-587 / 12822) chromosome, complete sequence.

import namespace="svg" implementation="#AdobeSVG"?


# Locus BX470249B

# List of CDS in T6SS locus BX470249B

|  |  |  |  |  |  |  |  |  |
| --- | --- | --- | --- | --- | --- | --- | --- | --- |
| Name | from | to | direct | COG | e-value | COG cover | COG hit start | COG hit end |
| BX470249\_BPP0709 | 748898 | 751447 | True | COG1357 | 4e-17 | 80.0 | 17 | 207 |
| BX470249\_BPP0709 | 748898 | 751447 | True | COG5351 | 3e-22 | 54.0 | 71 | 270 |
| BX470249\_BPP0711 | 752527 | 753432 | True | - | - | - | - | - |
| BX470249\_BPP0712 | 753473 | 754033 | True | - | - | - | - | - |
| BX470249\_BPP0713 | 754606 | 755655 | True | COG3515 | 2e-19 | 97.0 | 3 | 341 |
| BX470249\_BPP0714 | 755698 | 756261 | True | COG3516 | 4e-55 | 98.0 | 3 | 169 |
| BX470249\_BPP0715 | 756283 | 757785 | True | COG3517 | 0.0 | 97.0 | 12 | 495 |
| BX470249\_BPP0716 | 757843 | 758328 | True | COG3157 | 3e-20 | 100.0 | 1 | 162 |
| BX470249\_BPP0717 | 758378 | 759187 | True | COG4455 | 1e-52 | 91.0 | 13 | 263 |
| BX470249\_BPP0718 | 759184 | 759702 | True | COG3518 | 3e-17 | 92.0 | 5 | 150 |
| BX470249\_BPP0719 | 759702 | 761615 | True | COG3519 | 1e-168 | 100.0 | 1 | 621 |
| BX470249\_BPP0720 | 761650 | 762309 | True | - | - | - | - | - |
| BX470249\_BPP0721 | 762312 | 763406 | True | COG3520 | 3e-63 | 94.0 | 12 | 327 |
| BX470249\_BPP0722 | 763403 | 763936 | True | - | - | - | - | - |
| BX470249\_BPP0723 | 763926 | 764303 | True | COG4893 | 3e-15 | 97.0 | 4 | 123 |
| BX470249\_BPP0724 | 764327 | 766900 | True | COG0542 | 0.0 | 99.0 | 1 | 784 |
| BX470249\_BPP0725 | 766999 | 767799 | False | - | - | - | - | - |
| BX470249\_BPP0726 | 767827 | 768222 | False | - | - | - | - | - |
| BX470249\_BPP0727 | 768487 | 769029 | True | COG3521 | 1e-26 | 89.0 | 2 | 144 |
| BX470249\_BPP0728 | 769075 | 770409 | True | COG3522 | 3e-127 | 100.0 | 1 | 446 |
| BX470249\_BPP0729 | 770430 | 771710 | True | COG3455 | 1e-59 | 95.0 | 10 | 260 |
| BX470249\_BPP0729 | 770430 | 771710 | True | COG1360 | 1e-27 | 65.0 | 86 | 244 |
| BX470249\_BPP0730 | 771707 | 775312 | True | COG3523 | 0.0 | 99.0 | 2 | 1186 |
| BX470249\_BPP0731 | 775309 | 776043 | True | COG3913 | 5e-15 | 67.0 | 8 | 160 |
| BX470249\_BPP0732 | 776068 | 777270 | True | COG0515 | 8e-07 | 50.0 | 96 | 289 |
| BX470249\_BPP0734 | 778708 | 780120 | True | COG5361 | 3e-57 | 97.0 | 12 | 458 |
| BX470249\_BPP0735 | 780128 | 783082 | False | COG3468 | 1e-18 | 79.0 | 120 | 592 |
