# Supplementary material for: Dissecting the bacterial type VI secretion system by a genome wide in silico analysis: what can be learned from available microbial genomic resources?
Source: BMC Genomics. 2009 Mar 12;10:104. doi: 10.1186/1471-2164-10-104 (PMC2660368; doi:10.1186/1471-2164-10-104)
Supplement: Additional file 7 — Detailed description of all identified T6SS gene clusters. Archive containing the detailed description of each identified T6SS locus as an HTML file. [file 1471-2164-10-104-S7.tgz › LociHTML/HTML/BX470250A.html]

Locus BX470250A on Bordetella bronchiseptica (strain NCTC 13252 / RB50 / ATCC BAA-588) chromosome, complete sequence.

import namespace="svg" implementation="#AdobeSVG"?


# Locus BX470250A

# List of CDS in T6SS locus BX470250A

|  |  |  |  |  |  |  |  |  |
| --- | --- | --- | --- | --- | --- | --- | --- | --- |
| Name | from | to | direct | COG | e-value | COG cover | COG hit start | COG hit end |
| BX470250\_BB0787 | 825621 | 826130 | False | - | - | - | - | - |
| BX470250\_BB0788 | 826127 | 827497 | False | - | - | - | - | - |
| BX470250\_BB0789 | 827502 | 828008 | False | - | - | - | - | - |
| BX470250\_BB0790 | 828015 | 828809 | False | - | - | - | - | - |
| BX470250\_BB0791 | 828844 | 829884 | False | COG2805 | 1e-140 | 97.0 | 3 | 347 |
| BX470250\_BB0792 | 830131 | 830958 | True | COG1989 | 5e-27 | 89.0 | 9 | 235 |
| BX470250\_BB0793 | 831107 | 833470 | True | COG3501 | 2e-149 | 99.0 | 4 | 550 |
| BX470250\_BB0794 | 833487 | 833900 | True | - | - | - | - | - |
| BX470250\_BB0795 | 833897 | 836521 | True | COG1357 | 5e-21 | 93.0 | 17 | 238 |
| BX470250\_BB0795 | 833897 | 836521 | True | COG5351 | 2e-22 | 67.0 | 71 | 317 |
| BX470250\_BB0796 | 836511 | 837572 | True | COG1357 | 4e-19 | 95.0 | 9 | 236 |
| BX470250\_BB0797 | 837603 | 838511 | True | - | - | - | - | - |
| BX470250\_BB0798 | 838552 | 839112 | True | - | - | - | - | - |
| BX470250\_BB0799 | 839515 | 840564 | True | COG3515 | 2e-19 | 97.0 | 3 | 341 |
| BX470250\_BB0800 | 840607 | 841170 | True | COG3516 | 4e-55 | 98.0 | 3 | 169 |
| BX470250\_BB0801 | 841192 | 842694 | True | COG3517 | 0.0 | 97.0 | 12 | 495 |
| BX470250\_BB0802 | 842770 | 843237 | True | COG3157 | 8e-18 | 96.0 | 7 | 162 |
| BX470250\_BB0803 | 843287 | 844096 | True | COG4455 | 2e-52 | 91.0 | 13 | 263 |
| BX470250\_BB0804 | 844093 | 844611 | True | COG3518 | 3e-17 | 92.0 | 5 | 150 |
| BX470250\_BB0805 | 844611 | 846524 | True | COG3519 | 6e-169 | 100.0 | 1 | 621 |
| BX470250\_BB0806 | 846559 | 847218 | True | - | - | - | - | - |
| BX470250\_BB0807 | 847215 | 848309 | True | COG3520 | 5e-65 | 94.0 | 12 | 327 |
| BX470250\_BB0808 | 848306 | 848839 | True | - | - | - | - | - |
| BX470250\_BB0809 | 848829 | 849206 | True | COG4893 | 3e-15 | 97.0 | 4 | 123 |
| BX470250\_BB0810 | 849230 | 851839 | True | COG0542 | 0.0 | 99.0 | 1 | 784 |
| BX470250\_BB0811 | 851938 | 852738 | False | - | - | - | - | - |
| BX470250\_BB0812 | 852766 | 853161 | False | - | - | - | - | - |
| BX470250\_BB0813 | 853426 | 853968 | True | COG3521 | 5e-26 | 89.0 | 2 | 144 |
| BX470250\_BB0814 | 854014 | 855348 | True | COG3522 | 9e-128 | 100.0 | 1 | 446 |
| BX470250\_BB0815 | 855369 | 856649 | True | COG3455 | 1e-59 | 95.0 | 10 | 260 |
| BX470250\_BB0815 | 855369 | 856649 | True | COG1360 | 1e-27 | 65.0 | 86 | 244 |
| BX470250\_BB0816 | 856646 | 860251 | True | COG3523 | 0.0 | 99.0 | 2 | 1186 |
| BX470250\_BB0817 | 860248 | 860982 | True | COG3913 | 5e-15 | 67.0 | 8 | 160 |
| BX470250\_BB0818 | 861007 | 862185 | True | COG0515 | 6e-07 | 50.0 | 96 | 289 |
| BX470250\_BB0819 | 862283 | 863449 | True | COG0387 | 1e-50 | 96.0 | 12 | 367 |
| BX470250\_BB0820 | 863623 | 865035 | True | COG5361 | 2e-57 | 97.0 | 12 | 458 |
| BX470250\_BB0821 | 865043 | 868006 | False | COG3468 | 2e-19 | 79.0 | 120 | 592 |
