# Supplementary material for: Dissecting the bacterial type VI secretion system by a genome wide in silico analysis: what can be learned from available microbial genomic resources?
Source: BMC Genomics. 2009 Mar 12;10:104. doi: 10.1186/1471-2164-10-104 (PMC2660368; doi:10.1186/1471-2164-10-104)
Supplement: Additional file 7 — Detailed description of all identified T6SS gene clusters. Archive containing the detailed description of each identified T6SS locus as an HTML file. [file 1471-2164-10-104-S7.tgz › LociHTML/HTML/BX470251C.html]

Locus BX470251C on Photorhabdus luminescens laumondii (strain TT01) chromosome, complete sequence.

import namespace="svg" implementation="#AdobeSVG"?


# Locus BX470251C

# List of CDS in T6SS locus BX470251C

|  |  |  |  |  |  |  |  |  |
| --- | --- | --- | --- | --- | --- | --- | --- | --- |
| Name | from | to | direct | COG | e-value | COG cover | COG hit start | COG hit end |
| BX470251\_plu0352 | 372357 | 372677 | False | - | - | - | - | - |
| BX470251\_plu0353 | 372677 | 377092 | False | COG3209 | 1e-31 | 82.0 | 4 | 662 |
| BX470251\_plu0353 | 372677 | 377092 | False | COG4104 | 4e-12 | 89.0 | 10 | 97 |
| BX470251\_plu0354 | 377114 | 377533 | False | COG5435 | 1e-29 | 95.0 | 1 | 140 |
| BX470251\_plu0355 | 377581 | 379476 | False | COG3501 | 6e-170 | 98.0 | 1 | 541 |
| BX470251\_plu0357 | 380265 | 381647 | True | - | - | - | - | - |
| BX470251\_plu0359 | 382431 | 385976 | False | COG3523 | 0.0 | 100.0 | 1 | 1188 |
| BX470251\_plu0360 | 385973 | 387406 | False | COG3515 | 9e-17 | 75.0 | 18 | 279 |
| BX470251\_plu0361 | 387412 | 388059 | False | - | - | - | - | - |
| BX470251\_plu0362 | 388056 | 388856 | False | - | - | - | - | - |
| BX470251\_plu0363 | 388853 | 391498 | False | COG0542 | 0.0 | 99.0 | 1 | 785 |
| BX470251\_plu0364 | 391509 | 392279 | False | COG3455 | 7e-76 | 94.0 | 14 | 260 |
| BX470251\_plu0365 | 392279 | 393631 | False | COG3522 | 4e-144 | 100.0 | 1 | 446 |
| BX470251\_plu0366 | 393634 | 394200 | False | COG3521 | 2e-32 | 93.0 | 11 | 158 |
| BX470251\_plu0367 | 394200 | 395486 | False | COG3456 | 6e-95 | 100.0 | 1 | 430 |
| BX470251\_plu0368 | 395492 | 396472 | False | COG3520 | 3e-87 | 91.0 | 21 | 328 |
| BX470251\_plu0369 | 396508 | 398355 | False | COG3519 | 6e-154 | 99.0 | 3 | 621 |
| BX470251\_plu0370 | 398357 | 398797 | False | COG3518 | 6e-24 | 99.0 | 1 | 156 |
| BX470251\_plu0371 | 398804 | 400282 | False | COG3517 | 0.0 | 99.0 | 1 | 493 |
| BX470251\_plu0372 | 400306 | 400803 | False | COG3516 | 8e-46 | 98.0 | 2 | 167 |
| BX470251\_plu0373 | 401704 | 402222 | True | COG3157 | 8e-51 | 97.0 | 1 | 158 |
| BX470251\_plu0374 | 402861 | 403703 | True | COG2961 | 3e-123 | 100.0 | 1 | 279 |
| BX470251\_plu0375 | 403799 | 405169 | True | COG1249 | 4e-128 | 100.0 | 1 | 454 |
| BX470251\_plu0376 | 405435 | 406523 | True | - | - | - | - | - |
| BX470251\_plu0377 | 406520 | 408172 | True | - | - | - | - | - |
| BX470251\_plu0378 | 408253 | 408771 | False | COG1763 | 1e-44 | 100.0 | 1 | 161 |
| BX470251\_plu0379 | 408768 | 409367 | False | COG0746 | 4e-45 | 98.0 | 1 | 189 |
| BX470251\_plu0380 | 409576 | 410568 | True | COG2334 | 2e-63 | 99.0 | 2 | 331 |
| BX470251\_plu0381 | 410589 | 411212 | True | - | - | - | - | - |
| BX470251\_plu0382 | 411368 | 412444 | False | COG2865 | 7e-38 | 75.0 | 33 | 385 |
| BX470251\_plu0383 | 412510 | 415137 | False | COG1196 | 7e-07 | 33.0 | 743 | 1128 |
