# Supplementary material for: Dissecting the bacterial type VI secretion system by a genome wide in silico analysis: what can be learned from available microbial genomic resources?
Source: BMC Genomics. 2009 Mar 12;10:104. doi: 10.1186/1471-2164-10-104 (PMC2660368; doi:10.1186/1471-2164-10-104)
Supplement: Additional file 7 — Detailed description of all identified T6SS gene clusters. Archive containing the detailed description of each identified T6SS locus as an HTML file. [file 1471-2164-10-104-S7.tgz › LociHTML/HTML/BX470251I.html]

Locus BX470251I on Photorhabdus luminescens laumondii (strain TT01) chromosome, complete sequence.

import namespace="svg" implementation="#AdobeSVG"?


# Locus BX470251I

# List of CDS in T6SS locus BX470251I

|  |  |  |  |  |  |  |  |  |
| --- | --- | --- | --- | --- | --- | --- | --- | --- |
| Name | from | to | direct | COG | e-value | COG cover | COG hit start | COG hit end |
| BX470251\_plu2284 | 2679150 | 2682761 | False | COG0784 | 4e-17 | 92.0 | 4 | 123 |
| BX470251\_plu2284 | 2679150 | 2682761 | False | COG0834 | 5e-21 | 91.0 | 19 | 270 |
| BX470251\_plu2284 | 2679150 | 2682761 | False | COG0642 | 3e-34 | 72.0 | 90 | 331 |
| BX470251\_plu2285 | 2682765 | 2683391 | False | COG2197 | 1e-31 | 98.0 | 2 | 208 |
| BX470251\_plu2286 | 2683422 | 2684681 | False | - | - | - | - | - |
| BX470251\_plu2287 | 2684731 | 2687313 | False | COG0542 | 0.0 | 99.0 | 1 | 781 |
| BX470251\_plu2288 | 2687306 | 2690815 | False | COG3523 | 3e-27 | 31.0 | 9 | 387 |
| BX470251\_plu2289 | 2690831 | 2691451 | False | COG3455 | 6e-14 | 77.0 | 41 | 242 |
| BX470251\_plu2290 | 2691451 | 2692848 | False | COG3522 | 1e-43 | 99.0 | 2 | 445 |
| BX470251\_plu2291 | 2692852 | 2693529 | False | - | - | - | - | - |
| BX470251\_plu2292 | 2693522 | 2694625 | False | COG3515 | 1e-18 | 95.0 | 8 | 338 |
| BX470251\_plu2293 | 2694639 | 2694941 | False | COG4104 | 2e-07 | 95.0 | 4 | 97 |
| BX470251\_plu2294 | 2694938 | 2695534 | False | - | - | - | - | - |
| BX470251\_plu2295 | 2695569 | 2697575 | False | COG3501 | 4e-109 | 94.0 | 4 | 523 |
| BX470251\_plu2296 | 2697600 | 2698610 | False | COG3520 | 2e-32 | 89.0 | 24 | 322 |
| BX470251\_plu2297 | 2698601 | 2700412 | False | COG3519 | 4e-105 | 98.0 | 6 | 617 |
| BX470251\_plu2298 | 2700417 | 2700860 | False | COG3518 | 7e-11 | 68.0 | 8 | 114 |
| BX470251\_plu2299 | 2700912 | 2701406 | False | COG3157 | 9e-20 | 99.0 | 1 | 161 |
| BX470251\_plu2300 | 2701463 | 2702944 | False | COG3517 | 0.0 | 99.0 | 4 | 495 |
| BX470251\_plu2301 | 2702952 | 2703503 | False | COG3516 | 1e-35 | 98.0 | 2 | 168 |
| BX470251\_plu2302 | 2704228 | 2704482 | True | - | - | - | - | - |
| BX470251\_plu2303 | 2704727 | 2705602 | False | - | - | - | - | - |
| BX470251\_plu2305 | 2706247 | 2706519 | True | COG3905 | 5e-11 | 84.0 | 3 | 72 |
| BX470251\_plu2306 | 2706507 | 2706797 | True | COG3668 | 3e-09 | 90.0 | 1 | 89 |
| BX470251\_plu2308 | 2707235 | 2707513 | False | COG3636 | 1e-25 | 89.0 | 7 | 95 |
| BX470251\_plu2309 | 2707510 | 2707812 | False | COG3657 | 8e-32 | 97.0 | 3 | 99 |
