# Supplementary material for: Dissecting the bacterial type VI secretion system by a genome wide in silico analysis: what can be learned from available microbial genomic resources?
Source: BMC Genomics. 2009 Mar 12;10:104. doi: 10.1186/1471-2164-10-104 (PMC2660368; doi:10.1186/1471-2164-10-104)
Supplement: Additional file 7 — Detailed description of all identified T6SS gene clusters. Archive containing the detailed description of each identified T6SS locus as an HTML file. [file 1471-2164-10-104-S7.tgz › LociHTML/HTML/BX470251M.html]

Locus BX470251M on Photorhabdus luminescens laumondii (strain TT01) chromosome, complete sequence.

import namespace="svg" implementation="#AdobeSVG"?


# Locus BX470251M

# List of CDS in T6SS locus BX470251M

|  |  |  |  |  |  |  |  |  |
| --- | --- | --- | --- | --- | --- | --- | --- | --- |
| Name | from | to | direct | COG | e-value | COG cover | COG hit start | COG hit end |
| BX470251\_plu3218 | 3805730 | 3806371 | False | - | - | - | - | - |
| BX470251\_plu3219 | 3807331 | 3808020 | True | - | - | - | - | - |
| BX470251\_plu3220 | 3808060 | 3808746 | True | COG2197 | 2e-11 | 35.0 | 134 | 208 |
| BX470251\_plu3221 | 3809059 | 3809727 | True | COG2197 | 7e-09 | 32.0 | 135 | 202 |
| BX470251\_plu3222 | 3810052 | 3810480 | False | COG3518 | 5e-17 | 91.0 | 9 | 151 |
| BX470251\_plu3223 | 3810485 | 3811024 | False | COG3521 | 7e-18 | 93.0 | 9 | 156 |
| BX470251\_plu3224 | 3811002 | 3812090 | False | COG3520 | 4e-62 | 92.0 | 15 | 323 |
| BX470251\_plu3225 | 3812054 | 3813814 | False | COG3519 | 1e-150 | 100.0 | 1 | 621 |
| BX470251\_plu3226 | 3814341 | 3815324 | False | - | - | - | - | - |
| BX470251\_plu3227 | 3815368 | 3817845 | False | - | - | - | - | - |
| BX470251\_plu3228 | 3817833 | 3818345 | False | - | - | - | - | - |
| BX470251\_plu3229 | 3818397 | 3818909 | False | - | - | - | - | - |
| BX470251\_plu3230 | 3818983 | 3819219 | True | - | - | - | - | - |
| BX470251\_plu3231 | 3819697 | 3823068 | False | COG3523 | 2e-121 | 99.0 | 9 | 1188 |
| BX470251\_plu3232 | 3823065 | 3824144 | False | - | - | - | - | - |
| BX470251\_plu3233 | 3824164 | 3824403 | False | - | - | - | - | - |
| BX470251\_plu3234 | 3824416 | 3824649 | False | - | - | - | - | - |
| BX470251\_plu3235 | 3824737 | 3825843 | False | - | - | - | - | - |
| BX470251\_plu3236 | 3825840 | 3827456 | False | COG1075 | 1e-06 | 16.0 | 105 | 159 |
| BX470251\_plu3237 | 3827457 | 3829982 | False | COG4253 | 5e-41 | 98.0 | 2 | 276 |
| BX470251\_plu3237 | 3827457 | 3829982 | False | COG3501 | 7e-78 | 96.0 | 20 | 549 |
| BX470251\_plu3238 | 3830821 | 3834171 | False | COG3523 | 3e-122 | 90.0 | 116 | 1188 |
| BX470251\_plu3239 | 3834188 | 3835339 | False | - | - | - | - | - |
| BX470251\_plu3240 | 3835336 | 3835593 | False | COG4104 | 2e-10 | 78.0 | 14 | 90 |
| BX470251\_plu3241 | 3835590 | 3838079 | False | - | - | - | - | - |
| BX470251\_plu3242 | 3838067 | 3838579 | False | - | - | - | - | - |
| BX470251\_plu3243 | 3838631 | 3839143 | False | - | - | - | - | - |
| BX470251\_plu3244 | 3839195 | 3839710 | False | - | - | - | - | - |
| BX470251\_plu3245 | 3839720 | 3840502 | False | - | - | - | - | - |
| BX470251\_plu3246 | 3840506 | 3842905 | False | COG4253 | 1e-33 | 79.0 | 2 | 222 |
| BX470251\_plu3246 | 3840506 | 3842905 | False | COG3501 | 5e-78 | 96.0 | 20 | 549 |
| BX470251\_plu3247 | 3843641 | 3847012 | False | COG3523 | 2e-120 | 99.0 | 9 | 1188 |
| BX470251\_plu3248 | 3847009 | 3848127 | False | - | - | - | - | - |
| BX470251\_plu3249 | 3848348 | 3848581 | False | - | - | - | - | - |
| BX470251\_plu3250 | 3848669 | 3849718 | False | - | - | - | - | - |
| BX470251\_plu3251 | 3849897 | 3850988 | False | - | - | - | - | - |
| BX470251\_plu3252 | 3850981 | 3852591 | False | - | - | - | - | - |
| BX470251\_plu3253 | 3852594 | 3855122 | False | COG4253 | 3e-38 | 99.0 | 2 | 278 |
| BX470251\_plu3253 | 3852594 | 3855122 | False | COG3501 | 2e-78 | 96.0 | 20 | 550 |
| BX470251\_plu3254 | 3855299 | 3855790 | False | COG3157 | 7e-35 | 98.0 | 1 | 160 |
| BX470251\_plu3255 | 3855809 | 3857536 | False | COG2885 | 2e-26 | 79.0 | 39 | 189 |
| BX470251\_plu3256 | 3857912 | 3858214 | True | - | - | - | - | - |
| BX470251\_plu3257 | 3858208 | 3858543 | True | COG3436 | 1e-09 | 63.0 | 39 | 138 |
| BX470251\_plu3258 | 3858607 | 3860112 | True | COG3436 | 1e-22 | 97.0 | 5 | 157 |
| BX470251\_plu3260 | 3860551 | 3861900 | False | COG3522 | 1e-99 | 99.0 | 2 | 446 |
| BX470251\_plu3261 | 3861916 | 3863442 | False | COG3517 | 0.0 | 100.0 | 1 | 495 |
| BX470251\_plu3262 | 3863474 | 3863971 | False | COG3516 | 9e-45 | 97.0 | 4 | 167 |
| BX470251\_plu3263 | 3865127 | 3880777 | False | COG1020 | 5e-152 | 99.0 | 1 | 641 |
| BX470251\_plu3263 | 3865127 | 3880777 | False | COG3319 | 2e-20 | 91.0 | 1 | 234 |
| BX470251\_plu3263 | 3865127 | 3880777 | False | COG1020 | 8e-121 | 99.0 | 1 | 641 |
| BX470251\_plu3263 | 3865127 | 3880777 | False | COG1020 | 2e-122 | 99.0 | 1 | 641 |
| BX470251\_plu3263 | 3865127 | 3880777 | False | COG1020 | 8e-123 | 100.0 | 1 | 642 |
| BX470251\_plu3263 | 3865127 | 3880777 | False | COG1020 | 4e-93 | 70.0 | 187 | 641 |
