# Supplementary material for: Dissecting the bacterial type VI secretion system by a genome wide in silico analysis: what can be learned from available microbial genomic resources?
Source: BMC Genomics. 2009 Mar 12;10:104. doi: 10.1186/1471-2164-10-104 (PMC2660368; doi:10.1186/1471-2164-10-104)
Supplement: Additional file 7 — Detailed description of all identified T6SS gene clusters. Archive containing the detailed description of each identified T6SS locus as an HTML file. [file 1471-2164-10-104-S7.tgz › LociHTML/HTML/BX470251N.html]

Locus BX470251N on Photorhabdus luminescens laumondii (strain TT01) chromosome, complete sequence.

import namespace="svg" implementation="#AdobeSVG"?


# Locus BX470251N

# List of CDS in T6SS locus BX470251N

|  |  |  |  |  |  |  |  |  |
| --- | --- | --- | --- | --- | --- | --- | --- | --- |
| Name | from | to | direct | COG | e-value | COG cover | COG hit start | COG hit end |
| BX470251\_plu4192 | 4906404 | 4907219 | False | COG1878 | 4e-23 | 81.0 | 38 | 215 |
| BX470251\_plu4193 | 4907212 | 4907925 | False | COG2091 | 3e-09 | 53.0 | 41 | 159 |
| BX470251\_plu4194 | 4907928 | 4908704 | False | COG1028 | 4e-42 | 98.0 | 5 | 250 |
| BX470251\_plu4195 | 4909679 | 4909996 | False | COG5606 | 2e-11 | 75.0 | 14 | 82 |
| BX470251\_plu4196 | 4910087 | 4910500 | False | COG4679 | 1e-07 | 55.0 | 51 | 114 |
| BX470251\_plu4197 | 4911020 | 4911514 | False | - | - | - | - | - |
| BX470251\_plu4198 | 4911700 | 4913079 | False | COG3515 | 3e-25 | 51.0 | 17 | 194 |
| BX470251\_plu4199 | 4913131 | 4913565 | False | COG3518 | 1e-15 | 92.0 | 4 | 149 |
| BX470251\_plu4200 | 4913569 | 4914105 | False | COG3521 | 4e-18 | 96.0 | 5 | 157 |
| BX470251\_plu4201 | 4914086 | 4915126 | False | COG3520 | 4e-61 | 94.0 | 14 | 331 |
| BX470251\_plu4202 | 4915126 | 4916892 | False | COG3519 | 2e-150 | 100.0 | 1 | 621 |
| BX470251\_plu4203 | 4916971 | 4918569 | False | COG3515 | 2e-20 | 99.0 | 1 | 345 |
| BX470251\_plu4204 | 4918572 | 4918697 | False | - | - | - | - | - |
| BX470251\_plu4205 | 4918735 | 4919478 | False | - | - | - | - | - |
| BX470251\_plu4206 | 4919689 | 4920690 | False | - | - | - | - | - |
| BX470251\_plu4208 | 4921260 | 4922642 | True | - | - | - | - | - |
| BX470251\_plu4210 | 4923248 | 4925854 | False | - | - | - | - | - |
| BX470251\_plu4211 | 4925955 | 4926446 | False | COG3157 | 6e-35 | 98.0 | 1 | 160 |
| BX470251\_plu4212 | 4926419 | 4926589 | False | - | - | - | - | - |
| BX470251\_plu4213 | 4926590 | 4926778 | False | - | - | - | - | - |
| BX470251\_plu4214 | 4927191 | 4930550 | False | COG3523 | 2e-109 | 98.0 | 20 | 1188 |
| BX470251\_plu4215 | 4930543 | 4931718 | False | - | - | - | - | - |
| BX470251\_plu4216 | 4931721 | 4931996 | False | COG4104 | 8e-12 | 91.0 | 9 | 98 |
| BX470251\_plu4217 | 4932024 | 4932692 | False | - | - | - | - | - |
| BX470251\_plu4218 | 4932878 | 4933867 | False | - | - | - | - | - |
| BX470251\_plu4219 | 4933945 | 4934955 | False | - | - | - | - | - |
| BX470251\_plu4220 | 4934952 | 4937552 | False | - | - | - | - | - |
| BX470251\_plu4221 | 4937552 | 4938592 | False | - | - | - | - | - |
| BX470251\_plu4222 | 4938603 | 4940981 | False | COG4253 | 8e-36 | 76.0 | 1 | 213 |
| BX470251\_plu4222 | 4938603 | 4940981 | False | COG3501 | 3e-76 | 99.0 | 4 | 549 |
| BX470251\_plu4223 | 4940978 | 4943659 | False | COG0542 | 0.0 | 99.0 | 1 | 780 |
| BX470251\_plu4224 | 4943908 | 4945584 | False | COG2885 | 1e-25 | 82.0 | 33 | 189 |
| BX470251\_plu4225 | 4945601 | 4946245 | False | COG3455 | 2e-22 | 74.0 | 50 | 245 |
| BX470251\_plu4226 | 4946288 | 4947649 | False | COG3522 | 8e-100 | 100.0 | 1 | 446 |
| BX470251\_plu4227 | 4947666 | 4949198 | False | COG3517 | 0.0 | 99.0 | 2 | 494 |
| BX470251\_plu4228 | 4950105 | 4950545 | False | - | - | - | - | - |
| BX470251\_plu4229 | 4950825 | 4952036 | False | COG2814 | 6e-26 | 87.0 | 17 | 361 |
| BX470251\_plu4230 | 4952453 | 4953391 | True | - | - | - | - | - |
| BX470251\_plu4231 | 4953811 | 4954812 | True | - | - | - | - | - |
