# Supplementary material for: Dissecting the bacterial type VI secretion system by a genome wide in silico analysis: what can be learned from available microbial genomic resources?
Source: BMC Genomics. 2009 Mar 12;10:104. doi: 10.1186/1471-2164-10-104 (PMC2660368; doi:10.1186/1471-2164-10-104)
Supplement: Additional file 7 — Detailed description of all identified T6SS gene clusters. Archive containing the detailed description of each identified T6SS locus as an HTML file. [file 1471-2164-10-104-S7.tgz › LociHTML/HTML/BX571965D.html]

Locus BX571965D on Burkholderia pseudomallei (strain K96243) chromosome 1, complete sequence.

import namespace="svg" implementation="#AdobeSVG"?


# Locus BX571965D

# List of CDS in T6SS locus BX571965D

|  |  |  |  |  |  |  |  |  |
| --- | --- | --- | --- | --- | --- | --- | --- | --- |
| Name | from | to | direct | COG | e-value | COG cover | COG hit start | COG hit end |
| BX571965\_BPSL3093 | 3691032 | 3693293 | True | COG2274 | 0.0 | 98.0 | 1 | 696 |
| BX571965\_BPSL3094 | 3693290 | 3694738 | True | COG1538 | 7e-41 | 87.0 | 45 | 443 |
| BX571965\_BPSL3095 | 3694948 | 3695934 | False | - | - | - | - | - |
| BX571965\_BPSL3096 | 3696333 | 3696911 | True | - | - | - | - | - |
| BX571965\_BPSL3097 | 3697190 | 3701083 | True | COG3523 | 0.0 | 100.0 | 1 | 1188 |
| BX571965\_BPSL3098 | 3701080 | 3702069 | True | COG3913 | 2e-36 | 93.0 | 5 | 216 |
| BX571965\_BPSL3099 | 3702074 | 3703006 | True | COG2885 | 1e-25 | 84.0 | 27 | 186 |
| BX571965\_BPSL3100 | 3703205 | 3704326 | False | COG3515 | 6e-32 | 98.0 | 7 | 346 |
| BX571965\_BPSL3101 | 3704416 | 3707085 | False | COG0542 | 0.0 | 99.0 | 1 | 784 |
| BX571965\_BPSL3102 | 3707119 | 3708219 | False | COG3520 | 8e-61 | 99.0 | 1 | 332 |
| BX571965\_BPSL3103 | 3708183 | 3710009 | False | COG3519 | 3e-144 | 99.0 | 6 | 620 |
| BX571965\_BPSL3104 | 3710101 | 3710613 | False | COG3518 | 4e-34 | 98.0 | 3 | 157 |
| BX571965\_BPSL3105 | 3710641 | 3711144 | False | COG3157 | 2e-34 | 97.0 | 5 | 162 |
| BX571965\_BPSL3106 | 3711217 | 3712707 | False | COG3517 | 0.0 | 99.0 | 2 | 495 |
| BX571965\_BPSL3107 | 3712724 | 3713242 | False | COG3516 | 2e-47 | 99.0 | 2 | 169 |
| BX571965\_BPSL3108 | 3713279 | 3713950 | False | - | - | - | - | - |
| BX571965\_BPSL3109 | 3714325 | 3714939 | True | COG3521 | 9e-27 | 92.0 | 1 | 147 |
| BX571965\_BPSL3110 | 3715048 | 3716394 | True | COG3522 | 3e-112 | 100.0 | 1 | 446 |
| BX571965\_BPSL3111 | 3716391 | 3717176 | True | COG3455 | 3e-47 | 95.0 | 14 | 262 |
| BX571965\_BPSL3114 | 3718434 | 3719234 | True | - | - | - | - | - |
| BX571965\_BPSL3115 | 3719241 | 3720311 | True | COG1396 | 6e-08 | 82.0 | 4 | 102 |
| BX571965\_BPSL3115 | 3719241 | 3720311 | True | COG2856 | 9e-26 | 76.0 | 20 | 183 |
| BX571965\_BPSL3116 | 3720594 | 3720857 | True | - | - | - | - | - |
| BX571965\_BPSL3117 | 3720854 | 3721714 | True | COG2801 | 9e-13 | 91.0 | 20 | 232 |
| BX571965\_BPSL3118 | 3722117 | 3724717 | False | COG0286 | 4e-29 | 68.0 | 34 | 368 |
