# Supplementary material for: Dissecting the bacterial type VI secretion system by a genome wide in silico analysis: what can be learned from available microbial genomic resources?
Source: BMC Genomics. 2009 Mar 12;10:104. doi: 10.1186/1471-2164-10-104 (PMC2660368; doi:10.1186/1471-2164-10-104)
Supplement: Additional file 7 — Detailed description of all identified T6SS gene clusters. Archive containing the detailed description of each identified T6SS locus as an HTML file. [file 1471-2164-10-104-S7.tgz › LociHTML/HTML/BX571966E.html]

Locus BX571966E on Burkholderia pseudomallei (strain K96243) chromosome 2, complete sequence.

import namespace="svg" implementation="#AdobeSVG"?


# Locus BX571966E

# List of CDS in T6SS locus BX571966E

|  |  |  |  |  |  |  |  |  |
| --- | --- | --- | --- | --- | --- | --- | --- | --- |
| Name | from | to | direct | COG | e-value | COG cover | COG hit start | COG hit end |
| BX571966\_BPSS0091 | 110182 | 110727 | True | COG3539 | 4e-15 | 100.0 | 1 | 184 |
| BX571966\_BPSS0092 | 110792 | 111529 | True | COG3121 | 8e-59 | 97.0 | 6 | 234 |
| BX571966\_BPSS0093 | 111753 | 114386 | True | COG3188 | 0.0 | 96.0 | 12 | 818 |
| BX571966\_BPSS0094 | 114379 | 114951 | True | COG3539 | 5e-16 | 96.0 | 6 | 182 |
| BX571966\_BPSS0095 | 115016 | 115672 | True | COG3455 | 2e-10 | 68.0 | 68 | 247 |
| BX571966\_BPSS0096 | 115675 | 117351 | True | COG2885 | 2e-26 | 79.0 | 40 | 190 |
| BX571966\_BPSS0097 | 117729 | 118268 | True | COG3516 | 4e-59 | 99.0 | 2 | 169 |
| BX571966\_BPSS0098 | 118302 | 119801 | True | COG3517 | 0.0 | 100.0 | 1 | 495 |
| BX571966\_BPSS0099 | 120001 | 120483 | True | COG3157 | 2e-35 | 99.0 | 1 | 161 |
| BX571966\_BPSS0100 | 120671 | 121153 | True | COG3521 | 1e-25 | 84.0 | 10 | 143 |
| BX571966\_BPSS0101 | 121159 | 122508 | True | COG3522 | 4e-135 | 99.0 | 1 | 445 |
| BX571966\_BPSS0102 | 122505 | 123806 | True | COG3455 | 9e-49 | 93.0 | 15 | 260 |
| BX571966\_BPSS0102 | 122505 | 123806 | True | COG1360 | 5e-28 | 65.0 | 81 | 241 |
| BX571966\_BPSS0103 | 123821 | 127729 | True | COG3523 | 0.0 | 99.0 | 2 | 1184 |
| BX571966\_BPSS0104 | 127919 | 128488 | True | - | - | - | - | - |
| BX571966\_BPSS0105 | 128553 | 131252 | True | COG3501 | 6e-132 | 90.0 | 24 | 523 |
| BX571966\_BPSS0105 | 128553 | 131252 | True | COG3889 | 3e-14 | 19.0 | 678 | 845 |
| BX571966\_BPSS0108 | 134954 | 136312 | False | - | - | - | - | - |
| BX571966\_BPSS0109 | 136345 | 137373 | False | - | - | - | - | - |
| BX571966\_BPSS0110 | 137428 | 138498 | False | COG3515 | 7e-21 | 97.0 | 1 | 338 |
| BX571966\_BPSS0111 | 138517 | 139566 | False | COG3520 | 1e-81 | 97.0 | 9 | 334 |
| BX571966\_BPSS0112 | 139563 | 141443 | False | COG3519 | 0.0 | 100.0 | 1 | 621 |
| BX571966\_BPSS0113 | 141445 | 141963 | False | COG3518 | 8e-21 | 95.0 | 8 | 157 |
| BX571966\_BPSS0114 | 141950 | 142804 | False | COG4455 | 9e-58 | 95.0 | 12 | 273 |
| BX571966\_BPSS0115 | 142788 | 143843 | False | - | - | - | - | - |
| BX571966\_BPSS0116 | 144273 | 146918 | True | COG0542 | 0.0 | 98.0 | 1 | 775 |
| BX571966\_BPSS0117 | 147309 | 150353 | False | COG2204 | 6e-18 | 34.0 | 6 | 165 |
| BX571966\_BPSS0117 | 147309 | 150353 | False | COG0642 | 7e-41 | 82.0 | 57 | 333 |
| BX571966\_BPSS0118 | 151025 | 151285 | True | - | - | - | - | - |
