# Supplementary material for: Dissecting the bacterial type VI secretion system by a genome wide in silico analysis: what can be learned from available microbial genomic resources?
Source: BMC Genomics. 2009 Mar 12;10:104. doi: 10.1186/1471-2164-10-104 (PMC2660368; doi:10.1186/1471-2164-10-104)
Supplement: Additional file 7 — Detailed description of all identified T6SS gene clusters. Archive containing the detailed description of each identified T6SS locus as an HTML file. [file 1471-2164-10-104-S7.tgz › LociHTML/HTML/BX571966F.html]

Locus BX571966F on Burkholderia pseudomallei (strain K96243) chromosome 2, complete sequence.

import namespace="svg" implementation="#AdobeSVG"?


# Locus BX571966F

# List of CDS in T6SS locus BX571966F

|  |  |  |  |  |  |  |  |  |
| --- | --- | --- | --- | --- | --- | --- | --- | --- |
| Name | from | to | direct | COG | e-value | COG cover | COG hit start | COG hit end |
| BX571966\_BPSS0163 | 215120 | 216988 | True | COG0367 | 4e-105 | 95.0 | 1 | 520 |
| BX571966\_BPSS0164 | 217017 | 217988 | True | COG0604 | 5e-63 | 100.0 | 1 | 326 |
| BX571966\_BPSS0165 | 218043 | 218969 | True | COG3384 | 1e-15 | 78.0 | 31 | 240 |
| BX571966\_BPSS0166 | 219213 | 220175 | False | COG0583 | 3e-33 | 97.0 | 2 | 292 |
| BX571966\_BPSS0167 | 221397 | 225473 | False | COG3523 | 0.0 | 99.0 | 2 | 1184 |
| BX571966\_BPSS0168 | 225502 | 226767 | False | COG3455 | 1e-47 | 94.0 | 15 | 262 |
| BX571966\_BPSS0168 | 225502 | 226767 | False | COG1360 | 1e-26 | 67.0 | 79 | 242 |
| BX571966\_BPSS0169 | 226856 | 228202 | False | COG3522 | 9e-128 | 100.0 | 1 | 446 |
| BX571966\_BPSS0170 | 228224 | 228736 | False | COG3521 | 4e-29 | 93.0 | 8 | 155 |
| BX571966\_BPSS0171 | 228834 | 229319 | False | COG3157 | 4e-37 | 94.0 | 1 | 153 |
| BX571966\_BPSS0172 | 229436 | 230935 | False | COG3517 | 0.0 | 100.0 | 1 | 495 |
| BX571966\_BPSS0173 | 230970 | 231551 | False | COG3516 | 5e-57 | 95.0 | 2 | 163 |
| BX571966\_BPSS0174 | 231587 | 234526 | False | COG0542 | 2e-122 | 57.0 | 1 | 452 |
| BX571966\_BPSS0174 | 231587 | 234526 | False | COG0542 | 4e-101 | 39.0 | 453 | 761 |
| BX571966\_BPSS0175 | 235059 | 235778 | True | - | - | - | - | - |
| BX571966\_BPSS0176 | 235775 | 236740 | True | COG4455 | 2e-51 | 94.0 | 17 | 273 |
| BX571966\_BPSS0177 | 236781 | 237308 | True | COG3518 | 8e-20 | 92.0 | 6 | 151 |
| BX571966\_BPSS0178 | 237339 | 239228 | True | COG3519 | 0.0 | 99.0 | 1 | 619 |
| BX571966\_BPSS0179 | 239240 | 240313 | True | COG3520 | 4e-73 | 98.0 | 7 | 335 |
| BX571966\_BPSS0180 | 240310 | 241455 | True | COG3515 | 7e-19 | 96.0 | 2 | 336 |
| BX571966\_BPSS0181 | 241527 | 243854 | True | COG3501 | 2e-131 | 94.0 | 10 | 531 |
| BX571966\_BPSS0182 | 243886 | 246363 | True | COG1357 | 6e-23 | 93.0 | 1 | 222 |
| BX571966\_BPSS0182 | 243886 | 246363 | True | COG5351 | 1e-21 | 49.0 | 100 | 281 |
| BX571966\_BPSS0183 | 246360 | 247442 | True | COG1357 | 4e-21 | 83.0 | 37 | 234 |
| BX571966\_BPSS0184 | 247624 | 248283 | True | - | - | - | - | - |
| BX571966\_BPSS0185 | 248330 | 248704 | True | - | - | - | - | - |
