# Supplementary material for: Dissecting the bacterial type VI secretion system by a genome wide in silico analysis: what can be learned from available microbial genomic resources?
Source: BMC Genomics. 2009 Mar 12;10:104. doi: 10.1186/1471-2164-10-104 (PMC2660368; doi:10.1186/1471-2164-10-104)
Supplement: Additional file 7 — Detailed description of all identified T6SS gene clusters. Archive containing the detailed description of each identified T6SS locus as an HTML file. [file 1471-2164-10-104-S7.tgz › LociHTML/HTML/BX571966G.html]

Locus BX571966G on Burkholderia pseudomallei (strain K96243) chromosome 2, complete sequence.

import namespace="svg" implementation="#AdobeSVG"?


# Locus BX571966G

# List of CDS in T6SS locus BX571966G

|  |  |  |  |  |  |  |  |  |
| --- | --- | --- | --- | --- | --- | --- | --- | --- |
| Name | from | to | direct | COG | e-value | COG cover | COG hit start | COG hit end |
| BX571966\_BPSS0512 | 697058 | 697831 | False | - | - | - | - | - |
| BX571966\_BPSS0513 | 697828 | 698517 | False | COG0558 | 5e-13 | 96.0 | 2 | 186 |
| BX571966\_BPSS0514 | 698792 | 700396 | True | COG0427 | 9e-161 | 98.0 | 8 | 501 |
| BX571966\_BPSS0515 | 702116 | 703054 | True | COG3515 | 8e-11 | 83.0 | 54 | 343 |
| BX571966\_BPSS0516 | 703088 | 703636 | True | COG3516 | 6e-47 | 97.0 | 5 | 169 |
| BX571966\_BPSS0517 | 703633 | 705144 | True | COG3517 | 0.0 | 99.0 | 1 | 494 |
| BX571966\_BPSS0518 | 705288 | 705815 | True | COG3157 | 2e-27 | 98.0 | 1 | 160 |
| BX571966\_BPSS0519 | 705856 | 706326 | True | - | - | - | - | - |
| BX571966\_BPSS0520 | 706340 | 708202 | True | COG3519 | 2e-87 | 96.0 | 3 | 604 |
| BX571966\_BPSS0521 | 708199 | 709188 | True | COG3520 | 1e-33 | 93.0 | 15 | 328 |
| BX571966\_BPSS0522 | 709191 | 712061 | True | COG0542 | 0.0 | 97.0 | 2 | 766 |
| BX571966\_BPSS0523 | 712052 | 714343 | True | COG3501 | 8e-136 | 97.0 | 10 | 544 |
| BX571966\_BPSS0524 | 714509 | 716797 | True | COG3501 | 3e-135 | 96.0 | 10 | 537 |
| BX571966\_BPSS0525 | 716801 | 719017 | True | COG1357 | 5e-08 | 61.0 | 58 | 204 |
| BX571966\_BPSS0526 | 719014 | 720087 | True | COG1357 | 7e-09 | 66.0 | 32 | 190 |
| BX571966\_BPSS0527 | 720090 | 720806 | True | - | - | - | - | - |
| BX571966\_BPSS0528 | 720849 | 721238 | True | - | - | - | - | - |
| BX571966\_BPSS0529 | 721244 | 721837 | True | COG3521 | 3e-08 | 62.0 | 7 | 106 |
| BX571966\_BPSS0530 | 721834 | 723195 | True | COG3522 | 5e-91 | 98.0 | 7 | 446 |
| BX571966\_BPSS0531 | 723278 | 724936 | True | COG3455 | 2e-25 | 98.0 | 1 | 257 |
| BX571966\_BPSS0531 | 723278 | 724936 | True | COG1360 | 1e-18 | 50.0 | 123 | 244 |
| BX571966\_BPSS0532 | 724933 | 728436 | True | COG3523 | 3e-139 | 98.0 | 17 | 1185 |
| BX571966\_BPSS0533 | 728495 | 728854 | True | - | - | - | - | - |
| BX571966\_BPSS0534 | 728877 | 729302 | True | - | - | - | - | - |
| BX571966\_BPSS0535 | 729527 | 729898 | True | - | - | - | - | - |
| BX571966\_BPSS0536 | 730449 | 731348 | True | - | - | - | - | - |
| BX571966\_BPSS0537 | 731570 | 732889 | True | COG1819 | 2e-39 | 98.0 | 1 | 399 |
| BX571966\_BPSS0538 | 732886 | 734472 | True | COG2814 | 3e-15 | 50.0 | 1 | 198 |
