# Supplementary material for: Dissecting the bacterial type VI secretion system by a genome wide in silico analysis: what can be learned from available microbial genomic resources?
Source: BMC Genomics. 2009 Mar 12;10:104. doi: 10.1186/1471-2164-10-104 (PMC2660368; doi:10.1186/1471-2164-10-104)
Supplement: Additional file 7 — Detailed description of all identified T6SS gene clusters. Archive containing the detailed description of each identified T6SS locus as an HTML file. [file 1471-2164-10-104-S7.tgz › LociHTML/HTML/BX571966I.html]

Locus BX571966I on Burkholderia pseudomallei (strain K96243) chromosome 2, complete sequence.

import namespace="svg" implementation="#AdobeSVG"?


# Locus BX571966I

# List of CDS in T6SS locus BX571966I

|  |  |  |  |  |  |  |  |  |
| --- | --- | --- | --- | --- | --- | --- | --- | --- |
| Name | from | to | direct | COG | e-value | COG cover | COG hit start | COG hit end |
| BX571966\_BPSS1493 | 2036034 | 2037980 | False | COG3515 | 3e-08 | 29.0 | 22 | 124 |
| BX571966\_BPSS1494 | 2037977 | 2038714 | False | COG0745 | 7e-52 | 99.0 | 3 | 229 |
| BX571966\_BPSS1495 | 2038711 | 2040555 | False | COG0642 | 2e-25 | 98.0 | 1 | 330 |
| BX571966\_BPSS1495 | 2038711 | 2040555 | False | COG3447 | 2e-07 | 86.0 | 41 | 306 |
| BX571966\_BPSS1496 | 2040815 | 2041309 | True | COG3516 | 2e-38 | 94.0 | 8 | 166 |
| BX571966\_BPSS1497 | 2041333 | 2042832 | True | COG3517 | 0.0 | 97.0 | 10 | 492 |
| BX571966\_BPSS1498 | 2043052 | 2043561 | True | COG3157 | 2e-12 | 95.0 | 1 | 154 |
| BX571966\_BPSS1499 | 2043554 | 2044015 | True | COG3518 | 9e-10 | 87.0 | 4 | 140 |
| BX571966\_BPSS1500 | 2044052 | 2045794 | True | COG3519 | 5e-102 | 99.0 | 7 | 621 |
| BX571966\_BPSS1501 | 2045782 | 2046804 | True | COG3520 | 2e-44 | 95.0 | 15 | 335 |
| BX571966\_BPSS1502 | 2046791 | 2049829 | True | COG0542 | 4e-124 | 54.0 | 1 | 427 |
| BX571966\_BPSS1502 | 2046791 | 2049829 | True | COG0542 | 6e-101 | 42.0 | 425 | 760 |
| BX571966\_BPSS1503 | 2049856 | 2052879 | True | COG3501 | 2e-82 | 83.0 | 4 | 463 |
| BX571966\_BPSS1504 | 2052905 | 2055547 | True | COG1357 | 3e-21 | 93.0 | 17 | 238 |
| BX571966\_BPSS1504 | 2052905 | 2055547 | True | COG1357 | 4e-09 | 47.0 | 125 | 237 |
| BX571966\_BPSS1504 | 2052905 | 2055547 | True | COG5351 | 7e-07 | 23.0 | 207 | 291 |
| BX571966\_BPSS1505 | 2055565 | 2056629 | True | COG1357 | 7e-16 | 95.0 | 1 | 227 |
| BX571966\_BPSS1505 | 2055565 | 2056629 | True | COG1357 | 8e-09 | 54.0 | 93 | 221 |
| BX571966\_BPSS1506 | 2056626 | 2057378 | True | - | - | - | - | - |
| BX571966\_BPSS1507 | 2057407 | 2057799 | True | - | - | - | - | - |
| BX571966\_BPSS1508 | 2057929 | 2058606 | True | - | - | - | - | - |
| BX571966\_BPSS1509 | 2058639 | 2060036 | True | COG3522 | 2e-42 | 98.0 | 5 | 445 |
| BX571966\_BPSS1510 | 2060051 | 2060698 | True | COG3455 | 2e-19 | 80.0 | 46 | 256 |
| BX571966\_BPSS1511 | 2060710 | 2064669 | True | COG3523 | 2e-44 | 37.0 | 18 | 467 |
| BX571966\_BPSS1512 | 2065185 | 2066609 | True | - | - | - | - | - |
