# Supplementary material for: Dissecting the bacterial type VI secretion system by a genome wide in silico analysis: what can be learned from available microbial genomic resources?
Source: BMC Genomics. 2009 Mar 12;10:104. doi: 10.1186/1471-2164-10-104 (PMC2660368; doi:10.1186/1471-2164-10-104)
Supplement: Additional file 7 — Detailed description of all identified T6SS gene clusters. Archive containing the detailed description of each identified T6SS locus as an HTML file. [file 1471-2164-10-104-S7.tgz › LociHTML/HTML/BX571966J.html]

Locus BX571966J on Burkholderia pseudomallei (strain K96243) chromosome 2, complete sequence.

import namespace="svg" implementation="#AdobeSVG"?


# Locus BX571966J

# List of CDS in T6SS locus BX571966J

|  |  |  |  |  |  |  |  |  |
| --- | --- | --- | --- | --- | --- | --- | --- | --- |
| Name | from | to | direct | COG | e-value | COG cover | COG hit start | COG hit end |
| BX571966\_BPSS2087 | 2825317 | 2826375 | False | COG1609 | 8e-78 | 100.0 | 1 | 333 |
| BX571966\_BPSS2089 | 2827766 | 2828551 | True | - | - | - | - | - |
| BX571966\_BPSS2090 | 2829283 | 2829711 | False | COG3677 | 7e-08 | 83.0 | 22 | 129 |
| BX571966\_BPSS2091 | 2829708 | 2830487 | False | COG3209 | 8e-10 | 18.0 | 2 | 145 |
| BX571966\_BPSS2093 | 2831227 | 2833431 | False | COG3501 | 3e-143 | 96.0 | 23 | 550 |
| BX571966\_BPSS2094 | 2833428 | 2836094 | False | COG0542 | 0.0 | 100.0 | 1 | 786 |
| BX571966\_BPSS2095 | 2836073 | 2837551 | False | COG3520 | 1e-56 | 94.0 | 3 | 318 |
| BX571966\_BPSS2096 | 2837548 | 2839419 | False | COG3519 | 2e-168 | 100.0 | 1 | 621 |
| BX571966\_BPSS2097 | 2839424 | 2839960 | False | COG3518 | 1e-17 | 94.0 | 1 | 149 |
| BX571966\_BPSS2098 | 2839991 | 2840482 | False | COG3157 | 3e-19 | 98.0 | 1 | 159 |
| BX571966\_BPSS2099 | 2840542 | 2842050 | False | COG3517 | 0.0 | 99.0 | 2 | 495 |
| BX571966\_BPSS2100 | 2842043 | 2842621 | False | COG3516 | 2e-53 | 98.0 | 2 | 167 |
| BX571966\_BPSS2101 | 2842684 | 2843763 | False | COG3515 | 8e-20 | 97.0 | 6 | 341 |
| BX571966\_BPSS2102 | 2843816 | 2846431 | False | COG0515 | 8e-31 | 72.0 | 1 | 277 |
| BX571966\_BPSS2103 | 2846642 | 2847574 | False | COG3913 | 3e-12 | 83.0 | 2 | 190 |
| BX571966\_BPSS2104 | 2847580 | 2851209 | False | COG3523 | 0.0 | 99.0 | 7 | 1185 |
| BX571966\_BPSS2105 | 2851212 | 2852528 | False | COG3455 | 2e-59 | 93.0 | 13 | 258 |
| BX571966\_BPSS2105 | 2851212 | 2852528 | False | COG1360 | 1e-27 | 56.0 | 103 | 240 |
| BX571966\_BPSS2106 | 2852544 | 2853932 | False | COG3522 | 2e-127 | 99.0 | 3 | 446 |
| BX571966\_BPSS2107 | 2853944 | 2854462 | False | COG3521 | 1e-25 | 99.0 | 1 | 158 |
| BX571966\_BPSS2108 | 2855102 | 2855503 | True | - | - | - | - | - |
| BX571966\_BPSS2109 | 2855558 | 2856994 | True | COG3456 | 1e-30 | 93.0 | 22 | 425 |
| BX571966\_BPSS2110 | 2857037 | 2858941 | False | - | - | - | - | - |
| BX571966\_BPSS2111 | 2859411 | 2860655 | True | COG0654 | 5e-35 | 87.0 | 2 | 340 |
| BX571966\_BPSS2113 | 2861331 | 2861507 | True | - | - | - | - | - |
| BX571966\_BPSS2114 | 2861989 | 2862693 | False | COG1028 | 2e-28 | 98.0 | 6 | 251 |
