# Supplementary material for: Dissecting the bacterial type VI secretion system by a genome wide in silico analysis: what can be learned from available microbial genomic resources?
Source: BMC Genomics. 2009 Mar 12;10:104. doi: 10.1186/1471-2164-10-104 (PMC2660368; doi:10.1186/1471-2164-10-104)
Supplement: Additional file 7 — Detailed description of all identified T6SS gene clusters. Archive containing the detailed description of each identified T6SS locus as an HTML file. [file 1471-2164-10-104-S7.tgz › LociHTML/HTML/BX936398A.html]

Locus BX936398A on Yersinia pseudotuberculosis (serovar I, strain IP32953) chromosome, complete sequence.

import namespace="svg" implementation="#AdobeSVG"?


# Locus BX936398A

# List of CDS in T6SS locus BX936398A

|  |  |  |  |  |  |  |  |  |
| --- | --- | --- | --- | --- | --- | --- | --- | --- |
| Name | from | to | direct | COG | e-value | COG cover | COG hit start | COG hit end |
| BX936398\_YPTB0636 | 752607 | 754949 | False | COG1452 | 0.0 | 100.0 | 1 | 784 |
| BX936398\_YPTB0637 | 755134 | 755967 | True | COG1076 | 9e-35 | 100.0 | 1 | 174 |
| BX936398\_YPTB0638 | 756284 | 756904 | False | COG0564 | 1e-59 | 72.0 | 80 | 289 |
| BX936398\_YPTB0639 | 758127 | 758870 | False | COG5419 | 2e-48 | 100.0 | 1 | 160 |
| BX936398\_YPTB0640 | 759200 | 760213 | True | COG3515 | 4e-55 | 100.0 | 1 | 346 |
| BX936398\_YPTB0641 | 760224 | 760784 | True | COG3516 | 1e-57 | 99.0 | 2 | 169 |
| BX936398\_YPTB0642 | 760784 | 762295 | True | COG3517 | 0.0 | 100.0 | 1 | 495 |
| BX936398\_YPTB0643 | 762458 | 762976 | True | COG3157 | 7e-36 | 100.0 | 1 | 162 |
| BX936398\_YPTB0644 | 763050 | 763493 | True | COG3518 | 8e-31 | 95.0 | 4 | 153 |
| BX936398\_YPTB0645 | 763526 | 765370 | True | COG3519 | 0.0 | 99.0 | 1 | 617 |
| BX936398\_YPTB0646 | 765363 | 766346 | True | COG3520 | 2e-83 | 98.0 | 4 | 334 |
| BX936398\_YPTB0647 | 766349 | 768952 | True | COG0542 | 0.0 | 99.0 | 1 | 783 |
| BX936398\_YPTB0648 | 769056 | 771404 | True | COG3501 | 5e-154 | 95.0 | 10 | 533 |
| BX936398\_YPTB0649 | 771417 | 773636 | True | COG1357 | 6e-11 | 92.0 | 9 | 227 |
| BX936398\_YPTB0649 | 771417 | 773636 | True | COG1357 | 1e-15 | 83.0 | 18 | 215 |
| BX936398\_YPTB0649 | 771417 | 773636 | True | COG5351 | 1e-07 | 50.0 | 68 | 253 |
| BX936398\_YPTB0650 | 773662 | 774765 | True | COG1357 | 1e-18 | 99.0 | 3 | 238 |
| BX936398\_YPTB0651 | 774758 | 775375 | True | - | - | - | - | - |
| BX936398\_YPTB0652 | 775381 | 775746 | True | - | - | - | - | - |
| BX936398\_YPTB0653 | 775739 | 776230 | True | COG3521 | 3e-34 | 98.0 | 3 | 158 |
| BX936398\_YPTB0654 | 776350 | 777705 | True | COG3522 | 2e-141 | 100.0 | 1 | 446 |
| BX936398\_YPTB0655 | 777702 | 779312 | True | COG3455 | 1e-72 | 100.0 | 1 | 262 |
| BX936398\_YPTB0655 | 777702 | 779312 | True | COG1360 | 2e-27 | 56.0 | 108 | 244 |
| BX936398\_YPTB0656 | 779321 | 782815 | True | COG3523 | 0.0 | 98.0 | 12 | 1187 |
| BX936398\_YPTB0657 | 782837 | 783190 | True | - | - | - | - | - |
| BX936398\_YPTB0658 | 783446 | 786352 | False | COG0553 | 3e-72 | 98.0 | 8 | 861 |
| BX936398\_YPTB0659 | 786813 | 789182 | False | COG0417 | 0.0 | 98.0 | 5 | 788 |
