# Supplementary material for: Dissecting the bacterial type VI secretion system by a genome wide in silico analysis: what can be learned from available microbial genomic resources?
Source: BMC Genomics. 2009 Mar 12;10:104. doi: 10.1186/1471-2164-10-104 (PMC2660368; doi:10.1186/1471-2164-10-104)
Supplement: Additional file 7 — Detailed description of all identified T6SS gene clusters. Archive containing the detailed description of each identified T6SS locus as an HTML file. [file 1471-2164-10-104-S7.tgz › LociHTML/HTML/BX936398B.html]

Locus BX936398B on Yersinia pseudotuberculosis (serovar I, strain IP32953) chromosome, complete sequence.

import namespace="svg" implementation="#AdobeSVG"?


# Locus BX936398B

# List of CDS in T6SS locus BX936398B

|  |  |  |  |  |  |  |  |  |
| --- | --- | --- | --- | --- | --- | --- | --- | --- |
| Name | from | to | direct | COG | e-value | COG cover | COG hit start | COG hit end |
| BX936398\_YPTB1476 | 1770779 | 1771498 | True | COG1024 | 6e-29 | 87.0 | 29 | 252 |
| BX936398\_YPTB1477 | 1771491 | 1772252 | True | COG1024 | 3e-37 | 93.0 | 1 | 240 |
| BX936398\_YPTB1478 | 1772255 | 1773043 | True | COG1028 | 4e-25 | 99.0 | 2 | 250 |
| BX936398\_YPTB1479 | 1773130 | 1773570 | True | - | - | - | - | - |
| BX936398\_YPTB1480 | 1773608 | 1773856 | True | - | - | - | - | - |
| BX936398\_YPTB1481 | 1773955 | 1774803 | True | COG0331 | 2e-74 | 93.0 | 2 | 290 |
| BX936398\_YPTB1482 | 1774960 | 1775127 | True | - | - | - | - | - |
| BX936398\_YPTB1483 | 1775792 | 1776292 | True | COG3516 | 6e-49 | 99.0 | 2 | 169 |
| BX936398\_YPTB1484 | 1776335 | 1777885 | True | COG3517 | 0.0 | 100.0 | 1 | 495 |
| BX936398\_YPTB1485 | 1777897 | 1779249 | True | COG3522 | 5e-132 | 99.0 | 2 | 446 |
| BX936398\_YPTB1486 | 1779246 | 1779932 | True | COG3455 | 5e-48 | 91.0 | 21 | 260 |
| BX936398\_YPTB1487 | 1779932 | 1781668 | True | COG2885 | 5e-27 | 94.0 | 12 | 190 |
| BX936398\_YPTB1488 | 1781672 | 1782163 | True | COG3157 | 2e-40 | 98.0 | 1 | 160 |
| BX936398\_YPTB1489 | 1782551 | 1785199 | True | COG0542 | 0.0 | 100.0 | 1 | 786 |
| BX936398\_YPTB1490 | 1785196 | 1787694 | True | COG3501 | 9e-110 | 99.0 | 1 | 549 |
| BX936398\_YPTB1490 | 1785196 | 1787694 | True | COG4253 | 3e-67 | 93.0 | 2 | 260 |
| BX936398\_YPTB1491 | 1787812 | 1789083 | True | - | - | - | - | - |
| BX936398\_YPTB1492 | 1789090 | 1789482 | True | - | - | - | - | - |
| BX936398\_YPTB1493 | 1789798 | 1792500 | True | COG3501 | 7e-105 | 99.0 | 1 | 549 |
| BX936398\_YPTB1493 | 1789798 | 1792500 | True | COG4253 | 2e-66 | 99.0 | 2 | 277 |
| BX936398\_YPTB1494 | 1792503 | 1792919 | True | - | - | - | - | - |
| BX936398\_YPTB1495 | 1792928 | 1794676 | True | - | - | - | - | - |
| BX936398\_YPTB1496 | 1794681 | 1795193 | True | - | - | - | - | - |
| BX936398\_YPTB1497 | 1796147 | 1797205 | True | - | - | - | - | - |
| BX936398\_YPTB1498 | 1797202 | 1800624 | True | COG3523 | 0.0 | 100.0 | 1 | 1188 |
| BX936398\_YPTB1499 | 1800668 | 1802269 | True | COG3515 | 1e-41 | 100.0 | 1 | 346 |
| BX936398\_YPTB1500 | 1802287 | 1803345 | True | - | - | - | - | - |
| BX936398\_YPTB1501 | 1803345 | 1803815 | True | - | - | - | - | - |
| BX936398\_YPTB1502 | 1804036 | 1805799 | True | COG3519 | 0.0 | 100.0 | 1 | 621 |
| BX936398\_YPTB1503 | 1805763 | 1806848 | True | COG3520 | 3e-85 | 97.0 | 1 | 328 |
| BX936398\_YPTB1504 | 1806724 | 1807425 | True | COG3521 | 1e-35 | 98.0 | 1 | 157 |
| BX936398\_YPTB1505 | 1807425 | 1807877 | True | COG3518 | 4e-27 | 98.0 | 3 | 157 |
| BX936398\_YPTB1506 | 1807902 | 1809269 | True | COG3515 | 8e-38 | 96.0 | 13 | 346 |
| BX936398\_YPTB1507 | 1809481 | 1810104 | False | - | - | - | - | - |
| BX936398\_YPTB1508 | 1810619 | 1812382 | True | COG0488 | 6e-172 | 100.0 | 1 | 530 |
| BX936398\_YPTB1509 | 1812610 | 1813257 | True | - | - | - | - | - |
| BX936398\_YPTB1510 | 1813329 | 1814099 | False | COG0476 | 7e-71 | 100.0 | 1 | 254 |
| BX936398\_YPTB1511 | 1814101 | 1815375 | False | COG0303 | 2e-130 | 100.0 | 1 | 404 |
