# Supplementary material for: Dissecting the bacterial type VI secretion system by a genome wide in silico analysis: what can be learned from available microbial genomic resources?
Source: BMC Genomics. 2009 Mar 12;10:104. doi: 10.1186/1471-2164-10-104 (PMC2660368; doi:10.1186/1471-2164-10-104)
Supplement: Additional file 7 — Detailed description of all identified T6SS gene clusters. Archive containing the detailed description of each identified T6SS locus as an HTML file. [file 1471-2164-10-104-S7.tgz › LociHTML/HTML/BX936398C.html]

Locus BX936398C on Yersinia pseudotuberculosis (serovar I, strain IP32953) chromosome, complete sequence.

import namespace="svg" implementation="#AdobeSVG"?


# Locus BX936398C

# List of CDS in T6SS locus BX936398C

|  |  |  |  |  |  |  |  |  |
| --- | --- | --- | --- | --- | --- | --- | --- | --- |
| Name | from | to | direct | COG | e-value | COG cover | COG hit start | COG hit end |
| BX936398\_YPTB2650 | 3128417 | 3128884 | False | - | - | - | - | - |
| BX936398\_YPTB2651 | 3129501 | 3130043 | True | COG1704 | 3e-55 | 99.0 | 2 | 185 |
| BX936398\_YPTB2652 | 3129988 | 3132087 | True | COG4907 | 2e-08 | 30.0 | 412 | 594 |
| BX936398\_YPTB2653 | 3132463 | 3132804 | True | COG2824 | 2e-46 | 100.0 | 1 | 112 |
| BX936398\_YPTB2654 | 3132879 | 3133244 | False | - | - | - | - | - |
| BX936398\_YPTB2655 | 3133251 | 3133730 | False | COG3518 | 4e-35 | 99.0 | 1 | 156 |
| BX936398\_YPTB2656 | 3133795 | 3134601 | False | COG4455 | 5e-107 | 100.0 | 1 | 273 |
| BX936398\_YPTB2657 | 3134621 | 3135469 | False | - | - | - | - | - |
| BX936398\_YPTB2658 | 3135469 | 3135729 | False | - | - | - | - | - |
| BX936398\_YPTB2659 | 3135809 | 3138721 | False | COG3501 | 6e-142 | 97.0 | 6 | 539 |
| BX936398\_YPTB2659 | 3135809 | 3138721 | False | COG3889 | 1e-06 | 17.0 | 694 | 845 |
| BX936398\_YPTB2660 | 3139015 | 3142842 | False | COG3523 | 0.0 | 99.0 | 3 | 1185 |
| BX936398\_YPTB2661 | 3142851 | 3144227 | False | COG3455 | 4e-53 | 85.0 | 40 | 262 |
| BX936398\_YPTB2661 | 3142851 | 3144227 | False | COG1360 | 6e-32 | 59.0 | 95 | 240 |
| BX936398\_YPTB2662 | 3144224 | 3145573 | False | COG3522 | 3e-158 | 99.0 | 2 | 446 |
| BX936398\_YPTB2663 | 3145577 | 3146137 | False | COG3521 | 4e-39 | 100.0 | 1 | 159 |
| BX936398\_YPTB2664 | 3146390 | 3146959 | False | COG3157 | 3e-42 | 100.0 | 1 | 162 |
| BX936398\_YPTB2665 | 3147208 | 3148710 | False | COG3517 | 0.0 | 100.0 | 1 | 495 |
| BX936398\_YPTB2666 | 3148734 | 3149258 | False | COG3516 | 8e-59 | 99.0 | 2 | 169 |
| BX936398\_YPTB2667 | 3149364 | 3149972 | False | COG3539 | 1e-15 | 97.0 | 6 | 184 |
| BX936398\_YPTB2668 | 3149957 | 3152668 | False | COG3188 | 1e-174 | 99.0 | 7 | 834 |
| BX936398\_YPTB2669 | 3152757 | 3153518 | False | COG3121 | 3e-58 | 94.0 | 12 | 234 |
| BX936398\_YPTB2670 | 3153675 | 3154220 | False | COG3539 | 1e-14 | 100.0 | 1 | 184 |
| BX936398\_YPTB2671 | 3154431 | 3157088 | False | COG0542 | 0.0 | 98.0 | 1 | 777 |
| BX936398\_YPTB2672 | 3157872 | 3159752 | True | COG3519 | 0.0 | 100.0 | 1 | 621 |
| BX936398\_YPTB2673 | 3159752 | 3160777 | True | COG3520 | 6e-98 | 100.0 | 1 | 335 |
| BX936398\_YPTB2674 | 3160876 | 3162069 | True | COG3515 | 2e-48 | 98.0 | 1 | 341 |
| BX936398\_YPTB2675 | 3162076 | 3163107 | True | - | - | - | - | - |
| BX936398\_YPTB2676 | 3163147 | 3164484 | True | - | - | - | - | - |
| BX936398\_YPTB2677 | 3164481 | 3165182 | True | - | - | - | - | - |
| BX936398\_YPTB2678 | 3165183 | 3166865 | True | COG2885 | 3e-27 | 55.0 | 86 | 190 |
| BX936398\_YPTB2679 | 3166862 | 3167344 | True | COG5435 | 7e-43 | 100.0 | 1 | 147 |
