# Supplementary material for: Dissecting the bacterial type VI secretion system by a genome wide in silico analysis: what can be learned from available microbial genomic resources?
Source: BMC Genomics. 2009 Mar 12;10:104. doi: 10.1186/1471-2164-10-104 (PMC2660368; doi:10.1186/1471-2164-10-104)
Supplement: Additional file 7 — Detailed description of all identified T6SS gene clusters. Archive containing the detailed description of each identified T6SS locus as an HTML file. [file 1471-2164-10-104-S7.tgz › LociHTML/HTML/BX936398D.html]

Locus BX936398D on Yersinia pseudotuberculosis (serovar I, strain IP32953) chromosome, complete sequence.

import namespace="svg" implementation="#AdobeSVG"?


# Locus BX936398D

# List of CDS in T6SS locus BX936398D

|  |  |  |  |  |  |  |  |  |
| --- | --- | --- | --- | --- | --- | --- | --- | --- |
| Name | from | to | direct | COG | e-value | COG cover | COG hit start | COG hit end |
| BX936398\_YPTB3238 | 3813445 | 3815742 | False | COG3179 | 7e-09 | 98.0 | 4 | 206 |
| BX936398\_YPTB3242 | 3817314 | 3817589 | False | COG3677 | 6e-22 | 71.0 | 26 | 117 |
| BX936398\_YPTB3243 | 3817986 | 3818486 | False | - | - | - | - | - |
| BX936398\_YPTB3244 | 3818487 | 3820709 | False | - | - | - | - | - |
| BX936398\_YPTB3245 | 3820724 | 3823072 | False | COG3501 | 3e-106 | 99.0 | 1 | 549 |
| BX936398\_YPTB3245 | 3820724 | 3823072 | False | COG4253 | 2e-65 | 82.0 | 2 | 229 |
| BX936398\_YPTB3246 | 3823069 | 3825717 | False | COG0542 | 0.0 | 100.0 | 1 | 786 |
| BX936398\_YPTB3247 | 3826135 | 3826626 | False | COG3157 | 2e-40 | 98.0 | 1 | 160 |
| BX936398\_YPTB3248 | 3826630 | 3828366 | False | COG2885 | 8e-27 | 94.0 | 12 | 190 |
| BX936398\_YPTB3249 | 3828366 | 3829052 | False | COG3455 | 8e-48 | 91.0 | 21 | 260 |
| BX936398\_YPTB3250 | 3829049 | 3830401 | False | COG3522 | 5e-132 | 99.0 | 2 | 446 |
| BX936398\_YPTB3251 | 3830413 | 3831957 | False | COG3517 | 0.0 | 100.0 | 1 | 495 |
| BX936398\_YPTB3252 | 3832006 | 3832506 | False | COG3516 | 6e-49 | 99.0 | 2 | 169 |
| BX936398\_YPTB3255 | 3834445 | 3834708 | False | COG3677 | 1e-18 | 68.0 | 26 | 114 |
| BX936398\_YPTB3256 | 3834776 | 3835171 | False | - | - | - | - | - |
| BX936398\_YPTB3257 | 3835622 | 3836272 | False | - | - | - | - | - |
| BX936398\_YPTB3258 | 3837443 | 3838093 | True | COG3916 | 3e-64 | 100.0 | 1 | 209 |
