# Supplementary material for: Dissecting the bacterial type VI secretion system by a genome wide in silico analysis: what can be learned from available microbial genomic resources?
Source: BMC Genomics. 2009 Mar 12;10:104. doi: 10.1186/1471-2164-10-104 (PMC2660368; doi:10.1186/1471-2164-10-104)
Supplement: Additional file 7 — Detailed description of all identified T6SS gene clusters. Archive containing the detailed description of each identified T6SS locus as an HTML file. [file 1471-2164-10-104-S7.tgz › LociHTML/HTML/BX936398E.html]

Locus BX936398E on Yersinia pseudotuberculosis (serovar I, strain IP32953) chromosome, complete sequence.

import namespace="svg" implementation="#AdobeSVG"?


# Locus BX936398E

# List of CDS in T6SS locus BX936398E

|  |  |  |  |  |  |  |  |  |
| --- | --- | --- | --- | --- | --- | --- | --- | --- |
| Name | from | to | direct | COG | e-value | COG cover | COG hit start | COG hit end |
| BX936398\_YPTB3608 | 4292045 | 4292974 | True | COG0524 | 3e-46 | 94.0 | 16 | 309 |
| BX936398\_YPTB3609 | 4292980 | 4293483 | True | COG1869 | 1e-43 | 97.0 | 1 | 131 |
| BX936398\_YPTB3610 | 4293643 | 4294521 | False | COG2207 | 9e-22 | 86.0 | 18 | 127 |
| BX936398\_YPTB3611 | 4294779 | 4295909 | True | COG0673 | 8e-50 | 99.0 | 2 | 342 |
| BX936398\_YPTB3612 | 4295906 | 4296805 | True | COG1082 | 2e-21 | 98.0 | 2 | 272 |
| BX936398\_YPTB3613 | 4297082 | 4297327 | False | COG3501 | 1e-14 | 11.0 | 226 | 287 |
| BX936398\_YPTB3614 | 4297516 | 4297797 | False | - | - | - | - | - |
| BX936398\_YPTB3615 | 4297794 | 4302356 | False | COG3209 | 4e-56 | 83.0 | 1 | 665 |
| BX936398\_YPTB3615 | 4297794 | 4302356 | False | COG4104 | 3e-10 | 73.0 | 25 | 96 |
| BX936398\_YPTB3616 | 4302401 | 4302823 | False | COG5435 | 6e-45 | 97.0 | 3 | 145 |
| BX936398\_YPTB3617 | 4302826 | 4305024 | False | COG3501 | 0.0 | 99.0 | 1 | 549 |
| BX936398\_YPTB3618 | 4305385 | 4305792 | True | - | - | - | - | - |
| BX936398\_YPTB3619 | 4306095 | 4306448 | True | - | - | - | - | - |
| BX936398\_YPTB3620 | 4306508 | 4307125 | False | - | - | - | - | - |
| BX936398\_YPTB3621 | 4307136 | 4311404 | False | COG3209 | 2e-49 | 91.0 | 1 | 730 |
| BX936398\_YPTB3621 | 4307136 | 4311404 | False | COG3209 | 2e-58 | 99.0 | 2 | 794 |
| BX936398\_YPTB3622 | 4311397 | 4311855 | False | COG5435 | 5e-48 | 100.0 | 1 | 147 |
| BX936398\_YPTB3623 | 4311861 | 4314080 | False | COG3501 | 0.0 | 99.0 | 1 | 547 |
| BX936398\_YPTB3624 | 4314102 | 4315415 | False | COG3515 | 2e-21 | 51.0 | 10 | 188 |
| BX936398\_YPTB3624 | 4314102 | 4315415 | False | COG3515 | 2e-37 | 96.0 | 12 | 344 |
| BX936398\_YPTB3625 | 4315540 | 4319073 | False | COG3523 | 0.0 | 100.0 | 1 | 1188 |
| BX936398\_YPTB3626 | 4319105 | 4320493 | False | COG3515 | 2e-36 | 82.0 | 1 | 285 |
| BX936398\_YPTB3627 | 4320499 | 4321185 | False | - | - | - | - | - |
| BX936398\_YPTB3628 | 4321182 | 4321979 | False | - | - | - | - | - |
| BX936398\_YPTB3629 | 4321976 | 4324579 | False | COG0542 | 0.0 | 99.0 | 1 | 784 |
| BX936398\_YPTB3630 | 4324590 | 4325357 | False | COG3455 | 2e-86 | 98.0 | 4 | 260 |
| BX936398\_YPTB3631 | 4325357 | 4326703 | False | COG3522 | 1e-167 | 100.0 | 1 | 446 |
| BX936398\_YPTB3632 | 4326706 | 4327251 | False | COG3521 | 2e-39 | 100.0 | 1 | 159 |
| BX936398\_YPTB3633 | 4327251 | 4328567 | False | COG3456 | 4e-123 | 100.0 | 1 | 430 |
| BX936398\_YPTB3634 | 4328693 | 4329781 | False | COG3520 | 7e-106 | 99.0 | 1 | 332 |
| BX936398\_YPTB3635 | 4329745 | 4331565 | False | COG3519 | 5e-160 | 99.0 | 3 | 621 |
| BX936398\_YPTB3636 | 4331565 | 4332005 | False | COG3518 | 9e-35 | 100.0 | 1 | 157 |
| BX936398\_YPTB3637 | 4332012 | 4333493 | False | COG3517 | 0.0 | 99.0 | 1 | 493 |
| BX936398\_YPTB3638 | 4333561 | 4334058 | False | COG3516 | 3e-49 | 98.0 | 2 | 167 |
| BX936398\_YPTB3639 | 4334582 | 4335100 | True | COG3157 | 7e-51 | 98.0 | 1 | 160 |
| BX936398\_YPTB3640 | 4335252 | 4335530 | False | - | - | - | - | - |
| BX936398\_YPTB3641 | 4336002 | 4336913 | False | - | - | - | - | - |
| BX936398\_YPTB3642 | 4337154 | 4338425 | False | COG4580 | 4e-150 | 99.0 | 2 | 429 |
| BX936398\_YPTB3643 | 4338496 | 4339605 | False | COG3839 | 9e-125 | 100.0 | 1 | 338 |
| BX936398\_YPTB3644 | 4339609 | 4339845 | False | - | - | - | - | - |
