# Supplementary material for: Dissecting the bacterial type VI secretion system by a genome wide in silico analysis: what can be learned from available microbial genomic resources?
Source: BMC Genomics. 2009 Mar 12;10:104. doi: 10.1186/1471-2164-10-104 (PMC2660368; doi:10.1186/1471-2164-10-104)
Supplement: Additional file 7 — Detailed description of all identified T6SS gene clusters. Archive containing the detailed description of each identified T6SS locus as an HTML file. [file 1471-2164-10-104-S7.tgz › LociHTML/HTML/BX950851C.html]

Locus BX950851C on Erwinia carotovora (subsp. atroseptica, strain ATCC BAA-672 / SCRI 1043) chromosome, complete sequence.

import namespace="svg" implementation="#AdobeSVG"?


# Locus BX950851C

# List of CDS in T6SS locus BX950851C

|  |  |  |  |  |  |  |  |  |
| --- | --- | --- | --- | --- | --- | --- | --- | --- |
| Name | from | to | direct | COG | e-value | COG cover | COG hit start | COG hit end |
| BX950851\_ECA3421 | 3829218 | 3834074 | False | COG3209 | 8e-37 | 83.0 | 1 | 667 |
| BX950851\_ECA3422 | 3834095 | 3835012 | False | - | - | - | - | - |
| BX950851\_ECA3423 | 3835009 | 3835965 | False | - | - | - | - | - |
| BX950851\_ECA3424 | 3836134 | 3836901 | False | - | - | - | - | - |
| BX950851\_ECA3425 | 3836898 | 3837677 | False | - | - | - | - | - |
| BX950851\_ECA3426 | 3837677 | 3839050 | False | COG4104 | 8e-13 | 100.0 | 1 | 98 |
| BX950851\_ECA3427 | 3839060 | 3841096 | False | COG3501 | 2e-152 | 99.0 | 6 | 550 |
| BX950851\_ECA3428 | 3841279 | 3841797 | False | COG3157 | 2e-49 | 98.0 | 1 | 160 |
| BX950851\_ECA3430 | 3842363 | 3843718 | False | COG0790 | 2e-11 | 89.0 | 22 | 282 |
| BX950851\_ECA3430 | 3842363 | 3843718 | False | COG0790 | 2e-08 | 75.0 | 43 | 262 |
| BX950851\_ECA3431 | 3843747 | 3845183 | False | COG3515 | 8e-18 | 47.0 | 19 | 184 |
| BX950851\_ECA3432 | 3845233 | 3848730 | False | COG3523 | 0.0 | 98.0 | 13 | 1188 |
| BX950851\_ECA3433 | 3848755 | 3850173 | False | COG3515 | 6e-15 | 79.0 | 10 | 285 |
| BX950851\_ECA3434 | 3850185 | 3850745 | False | - | - | - | - | - |
| BX950851\_ECA3435 | 3850745 | 3852298 | False | COG2204 | 8e-91 | 72.0 | 121 | 457 |
| BX950851\_ECA3436 | 3852301 | 3854898 | False | COG0542 | 0.0 | 99.0 | 1 | 784 |
| BX950851\_ECA3437 | 3854912 | 3855688 | False | COG3455 | 3e-62 | 94.0 | 13 | 259 |
| BX950851\_ECA3438 | 3855704 | 3857041 | False | COG3522 | 2e-149 | 100.0 | 1 | 446 |
| BX950851\_ECA3439 | 3857044 | 3857565 | False | COG3521 | 3e-31 | 97.0 | 5 | 159 |
| BX950851\_ECA3440 | 3857565 | 3858785 | False | COG3456 | 4e-63 | 99.0 | 3 | 428 |
| BX950851\_ECA3441 | 3858788 | 3859786 | False | COG3520 | 3e-84 | 93.0 | 17 | 328 |
| BX950851\_ECA3442 | 3859750 | 3861516 | False | COG3519 | 8e-145 | 99.0 | 3 | 618 |
| BX950851\_ECA3443 | 3861519 | 3861950 | False | COG3518 | 6e-20 | 92.0 | 7 | 152 |
| BX950851\_ECA3444 | 3861956 | 3863434 | False | COG3517 | 0.0 | 98.0 | 4 | 493 |
| BX950851\_ECA3445 | 3863466 | 3863969 | False | COG3516 | 2e-42 | 94.0 | 9 | 168 |
| BX950851\_ECA3446 | 3865039 | 3866442 | False | COG1538 | 3e-70 | 99.0 | 1 | 453 |
| BX950851\_ECA3447 | 3866439 | 3869582 | False | COG0841 | 0.0 | 99.0 | 2 | 1009 |
