# Supplementary material for: Dissecting the bacterial type VI secretion system by a genome wide in silico analysis: what can be learned from available microbial genomic resources?
Source: BMC Genomics. 2009 Mar 12;10:104. doi: 10.1186/1471-2164-10-104 (PMC2660368; doi:10.1186/1471-2164-10-104)
Supplement: Additional file 7 — Detailed description of all identified T6SS gene clusters. Archive containing the detailed description of each identified T6SS locus as an HTML file. [file 1471-2164-10-104-S7.tgz › LociHTML/HTML/CP000011C.html]

Locus CP000011C on Burkholderia mallei (strain ATCC 23344) chromosome 2, complete sequence.

import namespace="svg" implementation="#AdobeSVG"?


# Locus CP000011C

# List of CDS in T6SS locus CP000011C

|  |  |  |  |  |  |  |  |  |
| --- | --- | --- | --- | --- | --- | --- | --- | --- |
| Name | from | to | direct | COG | e-value | COG cover | COG hit start | COG hit end |
| CP000011\_BMAA0389 | 376517 | 377059 | True | - | - | - | - | - |
| CP000011\_BMAA0390 | 376995 | 377312 | False | - | - | - | - | - |
| CP000011\_BMAA0391 | 377622 | 378944 | False | COG0654 | 4e-36 | 87.0 | 2 | 340 |
| CP000011\_BMAA0392 | 379335 | 381233 | True | - | - | - | - | - |
| CP000011\_BMAA0393 | 381276 | 382712 | False | COG3456 | 2e-31 | 93.0 | 22 | 425 |
| CP000011\_BMAA0394 | 382767 | 383168 | False | - | - | - | - | - |
| CP000011\_BMAA0395 | 383354 | 383638 | True | - | - | - | - | - |
| CP000011\_BMAA0396 | 383787 | 384326 | True | COG3521 | 1e-25 | 99.0 | 1 | 158 |
| CP000011\_BMAA0397 | 384323 | 385726 | True | COG3522 | 2e-127 | 99.0 | 3 | 446 |
| CP000011\_BMAA0398 | 385742 | 387058 | True | COG3455 | 2e-59 | 93.0 | 13 | 258 |
| CP000011\_BMAA0398 | 385742 | 387058 | True | COG1360 | 1e-27 | 56.0 | 103 | 240 |
| CP000011\_BMAA0399 | 387061 | 390690 | True | COG3523 | 0.0 | 99.0 | 7 | 1185 |
| CP000011\_BMAA0400 | 390672 | 391211 | True | COG3913 | 3e-13 | 63.0 | 2 | 146 |
| CP000011\_BMAA0400.1 | 391813 | 394410 | True | COG0515 | 5e-31 | 72.0 | 1 | 277 |
| CP000011\_BMAA0402 | 394463 | 395542 | True | COG3515 | 1e-19 | 97.0 | 6 | 341 |
| CP000011\_BMAA0403 | 395605 | 396183 | True | COG3516 | 2e-52 | 98.0 | 2 | 167 |
| CP000011\_BMAA0404 | 396176 | 397684 | True | COG3517 | 0.0 | 99.0 | 2 | 495 |
| CP000011\_BMAA0405 | 397744 | 398235 | True | COG3157 | 3e-19 | 98.0 | 1 | 159 |
| CP000011\_BMAA0406 | 398266 | 398814 | True | COG3518 | 5e-18 | 94.0 | 1 | 149 |
| CP000011\_BMAA0407 | 398819 | 400690 | True | COG3519 | 2e-168 | 100.0 | 1 | 621 |
| CP000011\_BMAA0408 | 400807 | 402177 | True | COG3520 | 1e-52 | 94.0 | 3 | 318 |
| CP000011\_BMAA0409 | 402156 | 404810 | True | COG0542 | 0.0 | 100.0 | 1 | 786 |
| CP000011\_BMAA0410 | 404807 | 407011 | True | COG3501 | 3e-143 | 96.0 | 23 | 550 |
| CP000011\_BMAA0412 | 407699 | 408727 | True | COG3209 | 2e-14 | 37.0 | 49 | 347 |
| CP000011\_BMAA0413 | 408755 | 409588 | False | COG2801 | 8e-13 | 91.0 | 20 | 232 |
| CP000011\_BMAA0414 | 409612 | 409875 | False | - | - | - | - | - |
| CP000011\_BMAA0415 | 409775 | 410476 | False | - | - | - | - | - |
| CP000011\_BMAA0416 | 410549 | 411529 | False | - | - | - | - | - |
