# Supplementary material for: Dissecting the bacterial type VI secretion system by a genome wide in silico analysis: what can be learned from available microbial genomic resources?
Source: BMC Genomics. 2009 Mar 12;10:104. doi: 10.1186/1471-2164-10-104 (PMC2660368; doi:10.1186/1471-2164-10-104)
Supplement: Additional file 7 — Detailed description of all identified T6SS gene clusters. Archive containing the detailed description of each identified T6SS locus as an HTML file. [file 1471-2164-10-104-S7.tgz › LociHTML/HTML/CP000011D.html]

Locus CP000011D on Burkholderia mallei (strain ATCC 23344) chromosome 2, complete sequence.

import namespace="svg" implementation="#AdobeSVG"?


# Locus CP000011D

# List of CDS in T6SS locus CP000011D

|  |  |  |  |  |  |  |  |  |
| --- | --- | --- | --- | --- | --- | --- | --- | --- |
| Name | from | to | direct | COG | e-value | COG cover | COG hit start | COG hit end |
| CP000011\_BMAA0432 | 429068 | 430114 | True | COG0687 | 9e-51 | 95.0 | 16 | 363 |
| CP000011\_BMAA0433 | 430215 | 431474 | True | COG1176 | 5e-60 | 77.0 | 59 | 279 |
| CP000011\_BMAA0435 | 432426 | 432884 | False | COG4934 | 4e-10 | 12.0 | 425 | 571 |
| CP000011\_BMAA0436 | 432906 | 433739 | False | COG2801 | 8e-13 | 91.0 | 20 | 232 |
| CP000011\_BMAA0437 | 433763 | 434026 | False | - | - | - | - | - |
| CP000011\_BMAA0438 | 434138 | 434563 | True | COG3516 | 3e-31 | 76.0 | 40 | 169 |
| CP000011\_BMAA0439 | 434560 | 436071 | True | COG3517 | 0.0 | 99.0 | 1 | 494 |
| CP000011\_BMAA0440 | 436215 | 436742 | True | COG3157 | 8e-27 | 98.0 | 1 | 160 |
| CP000011\_BMAA0441 | 436822 | 437253 | True | - | - | - | - | - |
| CP000011\_BMAA0442 | 437267 | 439129 | True | COG3519 | 2e-87 | 96.0 | 3 | 604 |
| CP000011\_BMAA0443 | 439126 | 440115 | True | COG3520 | 1e-33 | 93.0 | 15 | 328 |
| CP000011\_BMAA0445 | 442979 | 445225 | True | COG3501 | 3e-134 | 92.0 | 10 | 520 |
| CP000011\_BMAA0446 | 445391 | 447679 | True | COG3501 | 3e-135 | 96.0 | 10 | 537 |
| CP000011\_BMAA0447 | 448268 | 449899 | True | COG1357 | 9e-08 | 61.0 | 58 | 204 |
| CP000011\_BMAA0448 | 449899 | 450969 | True | COG1357 | 8e-09 | 66.0 | 32 | 190 |
| CP000011\_BMAA0449 | 450972 | 451688 | True | - | - | - | - | - |
| CP000011\_BMAA0450 | 451731 | 452120 | True | - | - | - | - | - |
| CP000011\_BMAA0451 | 452126 | 452719 | True | COG3521 | 3e-08 | 62.0 | 7 | 106 |
| CP000011\_BMAA0453 | 454103 | 455818 | True | COG3455 | 2e-24 | 98.0 | 1 | 257 |
| CP000011\_BMAA0453 | 454103 | 455818 | True | COG1360 | 1e-18 | 50.0 | 123 | 244 |
| CP000011\_BMAA0454 | 455875 | 459318 | True | COG3523 | 2e-135 | 97.0 | 31 | 1185 |
| CP000011\_BMAA0455 | 459377 | 459736 | True | - | - | - | - | - |
| CP000011\_BMAA0456 | 459824 | 460327 | True | - | - | - | - | - |
| CP000011\_BMAA0457 | 460387 | 460548 | True | - | - | - | - | - |
| CP000011\_BMAA0458 | 460878 | 461051 | False | - | - | - | - | - |
| CP000011\_BMAA0459 | 461331 | 462230 | True | - | - | - | - | - |
| CP000011\_BMAA0460 | 462452 | 463771 | True | COG1819 | 7e-39 | 98.0 | 1 | 399 |
| CP000011\_BMAA0461 | 463768 | 465354 | True | COG2814 | 4e-15 | 50.0 | 1 | 198 |
