# Supplementary material for: Dissecting the bacterial type VI secretion system by a genome wide in silico analysis: what can be learned from available microbial genomic resources?
Source: BMC Genomics. 2009 Mar 12;10:104. doi: 10.1186/1471-2164-10-104 (PMC2660368; doi:10.1186/1471-2164-10-104)
Supplement: Additional file 7 — Detailed description of all identified T6SS gene clusters. Archive containing the detailed description of each identified T6SS locus as an HTML file. [file 1471-2164-10-104-S7.tgz › LociHTML/HTML/CP000011E.html]

Locus CP000011E on Burkholderia mallei (strain ATCC 23344) chromosome 2, complete sequence.

import namespace="svg" implementation="#AdobeSVG"?


# Locus CP000011E

# List of CDS in T6SS locus CP000011E

|  |  |  |  |  |  |  |  |  |
| --- | --- | --- | --- | --- | --- | --- | --- | --- |
| Name | from | to | direct | COG | e-value | COG cover | COG hit start | COG hit end |
| CP000011\_BMAA0729 | 736735 | 738714 | False | - | - | - | - | - |
| CP000011\_BMAA0729.1 | 738692 | 743269 | False | COG3523 | 2e-43 | 37.0 | 18 | 467 |
| CP000011\_BMAA0731 | 742735 | 743400 | False | COG3455 | 3e-20 | 82.0 | 41 | 256 |
| CP000011\_BMAA0732 | 743397 | 744794 | False | COG3522 | 2e-42 | 98.0 | 5 | 445 |
| CP000011\_BMAA0733 | 744827 | 745624 | False | - | - | - | - | - |
| CP000011\_BMAA0734 | 745634 | 746026 | False | - | - | - | - | - |
| CP000011\_BMAA0735 | 746055 | 746816 | False | - | - | - | - | - |
| CP000011\_BMAA0737 | 750563 | 753586 | False | COG3501 | 1e-82 | 83.0 | 4 | 463 |
| CP000011\_BMAA0738 | 753613 | 756696 | False | COG0542 | 2e-124 | 54.0 | 1 | 427 |
| CP000011\_BMAA0738 | 753613 | 756696 | False | COG0542 | 4e-101 | 42.0 | 425 | 760 |
| CP000011\_BMAA0739 | 756683 | 757705 | False | COG3520 | 1e-44 | 95.0 | 15 | 335 |
| CP000011\_BMAA0740 | 757693 | 759435 | False | COG3519 | 4e-102 | 99.0 | 7 | 621 |
| CP000011\_BMAA0741 | 759472 | 759933 | False | COG3518 | 6e-10 | 87.0 | 4 | 140 |
| CP000011\_BMAA0742 | 759926 | 760435 | False | COG3157 | 1e-12 | 95.0 | 1 | 154 |
| CP000011\_BMAA0743 | 760655 | 762154 | False | COG3517 | 0.0 | 97.0 | 10 | 492 |
| CP000011\_BMAA0744 | 762178 | 762672 | False | COG3516 | 3e-38 | 94.0 | 8 | 166 |
| CP000011\_BMAA0745 | 763148 | 764776 | True | COG0642 | 2e-25 | 98.0 | 1 | 330 |
| CP000011\_BMAA0745 | 763148 | 764776 | True | COG3447 | 6e-07 | 84.0 | 46 | 306 |
| CP000011\_BMAA0746 | 764773 | 765510 | True | COG0745 | 9e-52 | 99.0 | 3 | 229 |
| CP000011\_BMAA0747 | 765507 | 767435 | True | COG3515 | 3e-08 | 78.0 | 22 | 293 |
| CP000011\_BMAA0748 | 767778 | 768155 | False | - | - | - | - | - |
| CP000011\_BMAA0749 | 768235 | 769356 | False | COG5178 | 3e-08 | 1.0 | 5 | 30 |
| CP000011\_BMAA0749 | 768235 | 769356 | False | COG5178 | 2e-08 | 1.0 | 5 | 45 |
| CP000011\_BMAA0749 | 768235 | 769356 | False | COG5295 | 3e-18 | 27.0 | 519 | 715 |
| CP000011\_BMAA0750 | 769385 | 770605 | False | - | - | - | - | - |
| CP000011\_BMAA0751 | 770893 | 771918 | False | COG3023 | 3e-67 | 91.0 | 23 | 257 |
| CP000011\_BMAA0752 | 772063 | 772695 | False | - | - | - | - | - |
