# Supplementary material for: Dissecting the bacterial type VI secretion system by a genome wide in silico analysis: what can be learned from available microbial genomic resources?
Source: BMC Genomics. 2009 Mar 12;10:104. doi: 10.1186/1471-2164-10-104 (PMC2660368; doi:10.1186/1471-2164-10-104)
Supplement: Additional file 7 — Detailed description of all identified T6SS gene clusters. Archive containing the detailed description of each identified T6SS locus as an HTML file. [file 1471-2164-10-104-S7.tgz › LociHTML/HTML/CP000011F.html]

Locus CP000011F on Burkholderia mallei (strain ATCC 23344) chromosome 2, complete sequence.

import namespace="svg" implementation="#AdobeSVG"?


# Locus CP000011F

# List of CDS in T6SS locus CP000011F

|  |  |  |  |  |  |  |  |  |
| --- | --- | --- | --- | --- | --- | --- | --- | --- |
| Name | from | to | direct | COG | e-value | COG cover | COG hit start | COG hit end |
| CP000011\_BMAA1896 | 2076057 | 2076164 | False | - | - | - | - | - |
| CP000011\_BMAA1897 | 2076300 | 2076680 | False | - | - | - | - | - |
| CP000011\_BMAA1898 | 2076721 | 2077380 | False | - | - | - | - | - |
| CP000011\_BMAA1899 | 2077562 | 2078644 | False | COG1357 | 7e-21 | 82.0 | 37 | 233 |
| CP000011\_BMAA1900 | 2078641 | 2081118 | False | COG1357 | 6e-23 | 93.0 | 1 | 222 |
| CP000011\_BMAA1900 | 2078641 | 2081118 | False | COG5351 | 1e-21 | 49.0 | 100 | 281 |
| CP000011\_BMAA1901 | 2081150 | 2083477 | False | COG3501 | 4e-131 | 94.0 | 10 | 531 |
| CP000011\_BMAA1902 | 2083549 | 2084586 | False | COG3515 | 2e-19 | 95.0 | 8 | 336 |
| CP000011\_BMAA1904 | 2085733 | 2087622 | False | COG3519 | 0.0 | 99.0 | 1 | 619 |
| CP000011\_BMAA1905 | 2087653 | 2088234 | False | COG3518 | 1e-18 | 92.0 | 6 | 151 |
| CP000011\_BMAA1906 | 2088221 | 2089186 | False | COG4455 | 1e-49 | 95.0 | 14 | 273 |
| CP000011\_BMAA1907 | 2089183 | 2089929 | False | - | - | - | - | - |
| CP000011\_BMAA1909 | 2093394 | 2093975 | True | COG3516 | 2e-56 | 95.0 | 2 | 163 |
| CP000011\_BMAA1910 | 2094010 | 2095509 | True | COG3517 | 0.0 | 100.0 | 1 | 495 |
| CP000011\_BMAA1911 | 2095626 | 2096111 | True | COG3157 | 4e-37 | 94.0 | 1 | 153 |
| CP000011\_BMAA1912 | 2096218 | 2096721 | True | COG3521 | 5e-29 | 93.0 | 8 | 155 |
| CP000011\_BMAA1913 | 2096743 | 2098089 | True | COG3522 | 8e-128 | 100.0 | 1 | 446 |
| CP000011\_BMAA1914 | 2098163 | 2099443 | True | COG3455 | 5e-48 | 95.0 | 13 | 262 |
| CP000011\_BMAA1914 | 2098163 | 2099443 | True | COG1360 | 8e-27 | 67.0 | 79 | 242 |
| CP000011\_BMAA1916 | 2104079 | 2104315 | True | - | - | - | - | - |
| CP000011\_BMAA1917 | 2104304 | 2104654 | False | - | - | - | - | - |
