# Supplementary material for: Dissecting the bacterial type VI secretion system by a genome wide in silico analysis: what can be learned from available microbial genomic resources?
Source: BMC Genomics. 2009 Mar 12;10:104. doi: 10.1186/1471-2164-10-104 (PMC2660368; doi:10.1186/1471-2164-10-104)
Supplement: Additional file 7 — Detailed description of all identified T6SS gene clusters. Archive containing the detailed description of each identified T6SS locus as an HTML file. [file 1471-2164-10-104-S7.tgz › LociHTML/HTML/CP000020A.html]

Locus CP000020A on Vibrio fischeri (strain ATCC 700601 / ES114) chromosome I, complete sequence.

import namespace="svg" implementation="#AdobeSVG"?


# Locus CP000020A

# List of CDS in T6SS locus CP000020A

|  |  |  |  |  |  |  |  |  |
| --- | --- | --- | --- | --- | --- | --- | --- | --- |
| Name | from | to | direct | COG | e-value | COG cover | COG hit start | COG hit end |
| CP000020\_VF0987 | 1087244 | 1089196 | True | COG0840 | 2e-43 | 97.0 | 10 | 408 |
| CP000020\_VF0988 | 1089670 | 1090419 | True | COG3137 | 9e-16 | 97.0 | 8 | 262 |
| CP000020\_VF0989 | 1090606 | 1092501 | True | COG2199 | 1e-37 | 98.0 | 3 | 181 |
| CP000020\_VF0989 | 1090606 | 1092501 | True | COG2202 | 3e-08 | 57.0 | 100 | 232 |
| CP000020\_VF0990 | 1092502 | 1093461 | False | COG0515 | 8e-19 | 64.0 | 2 | 248 |
| CP000020\_VF0991 | 1093458 | 1094129 | False | COG0631 | 2e-31 | 95.0 | 4 | 253 |
| CP000020\_VF0992 | 1094126 | 1097200 | False | COG3523 | 5e-150 | 82.0 | 208 | 1187 |
| CP000020\_VF0993 | 1097627 | 1098985 | False | COG3523 | 2e-83 | 36.0 | 5 | 442 |
| CP000020\_VF0994 | 1098996 | 1099829 | False | COG3455 | 2e-59 | 98.0 | 1 | 259 |
| CP000020\_VF0995 | 1099831 | 1101153 | False | COG3522 | 7e-115 | 99.0 | 5 | 446 |
| CP000020\_VF0996 | 1101165 | 1101656 | False | COG3521 | 3e-28 | 91.0 | 13 | 158 |
| CP000020\_VF0997 | 1101693 | 1103003 | False | COG3456 | 2e-41 | 98.0 | 1 | 425 |
| CP000020\_VF0998 | 1103138 | 1104706 | False | COG2204 | 2e-89 | 76.0 | 107 | 460 |
| CP000020\_VF0999 | 1104712 | 1107294 | False | COG0542 | 0.0 | 96.0 | 2 | 760 |
| CP000020\_VF1000 | 1107291 | 1108331 | False | COG3520 | 6e-65 | 94.0 | 13 | 328 |
| CP000020\_VF1001 | 1108295 | 1110046 | False | COG3519 | 7e-108 | 99.0 | 3 | 621 |
| CP000020\_VF1002 | 1110043 | 1110468 | False | COG3518 | 4e-13 | 89.0 | 14 | 154 |
| CP000020\_VF1003 | 1110558 | 1112033 | False | COG3517 | 0.0 | 99.0 | 1 | 493 |
| CP000020\_VF1004 | 1112037 | 1112537 | False | COG3516 | 2e-37 | 97.0 | 5 | 168 |
| CP000020\_VF1005 | 1112553 | 1114103 | False | - | - | - | - | - |
| CP000020\_VF1006 | 1114326 | 1114532 | False | - | - | - | - | - |
| CP000020\_VF1007 | 1114525 | 1115076 | False | COG3923 | 2e-21 | 94.0 | 10 | 174 |
| CP000020\_VF1008 | 1115087 | 1115857 | False | COG0730 | 1e-22 | 98.0 | 2 | 256 |
| CP000020\_VF1009 | 1115926 | 1118010 | False | COG1199 | 2e-119 | 97.0 | 2 | 639 |
