# Supplementary material for: Dissecting the bacterial type VI secretion system by a genome wide in silico analysis: what can be learned from available microbial genomic resources?
Source: BMC Genomics. 2009 Mar 12;10:104. doi: 10.1186/1471-2164-10-104 (PMC2660368; doi:10.1186/1471-2164-10-104)
Supplement: Additional file 7 — Detailed description of all identified T6SS gene clusters. Archive containing the detailed description of each identified T6SS locus as an HTML file. [file 1471-2164-10-104-S7.tgz › LociHTML/HTML/CP000026A.html]

Locus CP000026A on Salmonella paratyphi-a (strain SARB42 / ATCC 9150) chromosome, complete sequence.

import namespace="svg" implementation="#AdobeSVG"?


# Locus CP000026A

# List of CDS in T6SS locus CP000026A

|  |  |  |  |  |  |  |  |  |
| --- | --- | --- | --- | --- | --- | --- | --- | --- |
| Name | from | to | direct | COG | e-value | COG cover | COG hit start | COG hit end |
| CP000026\_SPA2470 | 2569117 | 2571960 | False | COG3209 | 1e-35 | 83.0 | 2 | 664 |
| CP000026\_SPA2471 | 2574363 | 2576609 | False | COG3501 | 3e-161 | 97.0 | 3 | 538 |
| CP000026\_SPA2472 | 2576641 | 2577102 | False | - | - | - | - | - |
| CP000026\_SPA2473 | 2577104 | 2577847 | False | - | - | - | - | - |
| CP000026\_SPA2474 | 2577932 | 2578393 | False | - | - | - | - | - |
| CP000026\_SPA2475 | 2578378 | 2578848 | False | - | - | - | - | - |
| CP000026\_SPA2476 | 2578952 | 2579425 | False | - | - | - | - | - |
| CP000026\_SPA2478 | 2579922 | 2580689 | False | - | - | - | - | - |
| CP000026\_SPA2479 | 2580689 | 2584549 | False | COG3523 | 0.0 | 99.0 | 2 | 1188 |
| CP000026\_SPA2480 | 2584583 | 2585014 | False | - | - | - | - | - |
| CP000026\_SPA2481 | 2585216 | 2585989 | False | - | - | - | - | - |
| CP000026\_SPA2482 | 2585994 | 2587298 | False | COG3455 | 6e-72 | 99.0 | 1 | 261 |
| CP000026\_SPA2482 | 2585994 | 2587298 | False | COG1360 | 6e-32 | 58.0 | 101 | 242 |
| CP000026\_SPA2483 | 2587295 | 2588638 | False | COG3522 | 1e-153 | 100.0 | 1 | 446 |
| CP000026\_SPA2484 | 2588642 | 2589178 | False | COG3521 | 9e-43 | 96.0 | 4 | 157 |
| CP000026\_SPA2485 | 2589246 | 2589731 | False | COG3157 | 2e-42 | 100.0 | 1 | 162 |
| CP000026\_SPA2486 | 2589874 | 2590257 | False | - | - | - | - | - |
| CP000026\_SPA2487 | 2590242 | 2590727 | False | - | - | - | - | - |
| CP000026\_SPA2488 | 2590838 | 2590954 | False | - | - | - | - | - |
| CP000026\_SPA2489 | 2591021 | 2591506 | False | COG3157 | 2e-43 | 100.0 | 1 | 162 |
| CP000026\_SPA2490 | 2591758 | 2592090 | False | - | - | - | - | - |
| CP000026\_SPA2491 | 2592217 | 2592342 | False | - | - | - | - | - |
| CP000026\_SPA2492 | 2592670 | 2594178 | False | COG3517 | 0.0 | 100.0 | 1 | 495 |
| CP000026\_SPA2493 | 2594202 | 2594744 | False | COG3516 | 5e-57 | 99.0 | 2 | 169 |
| CP000026\_SPA2494 | 2594806 | 2595096 | False | - | - | - | - | - |
| CP000026\_SPA2495 | 2595099 | 2595197 | False | - | - | - | - | - |
| CP000026\_SPA2496 | 2595182 | 2597845 | False | COG0542 | 0.0 | 99.0 | 1 | 781 |
| CP000026\_SPA2497 | 2598133 | 2598204 | True | - | - | - | - | - |
| CP000026\_SPA2499 | 2599102 | 2599926 | True | COG4455 | 2e-109 | 100.0 | 1 | 273 |
| CP000026\_SPA2500 | 2599923 | 2600417 | True | COG3518 | 1e-35 | 98.0 | 1 | 154 |
| CP000026\_SPA2501 | 2600433 | 2602316 | True | COG3519 | 0.0 | 100.0 | 1 | 621 |
| CP000026\_SPA2502 | 2602313 | 2603308 | True | COG3520 | 1e-96 | 97.0 | 11 | 335 |
| CP000026\_SPA2503 | 2603319 | 2604362 | True | COG3515 | 5e-42 | 97.0 | 3 | 340 |
| CP000026\_SPA2505 | 2604893 | 2605624 | False | COG0847 | 3e-51 | 95.0 | 8 | 240 |
| CP000026\_SPA2506 | 2605688 | 2606155 | True | COG0328 | 1e-54 | 99.0 | 2 | 154 |
| CP000026\_SPA2507 | 2606152 | 2606874 | False | COG2226 | 1e-07 | 42.0 | 81 | 181 |
| CP000026\_SPA2508 | 2606909 | 2607664 | True | COG0491 | 2e-25 | 88.0 | 19 | 241 |
| CP000026\_SPA2509 | 2607736 | 2609103 | True | COG1388 | 1e-07 | 95.0 | 1 | 119 |
| CP000026\_SPA2509 | 2607736 | 2609103 | True | COG1388 | 9e-09 | 93.0 | 1 | 116 |
| CP000026\_SPA2509 | 2607736 | 2609103 | True | COG0741 | 4e-13 | 95.0 | 1 | 284 |
| CP000026\_SPA2510 | 2609159 | 2609929 | False | COG2226 | 1e-22 | 46.0 | 54 | 164 |
