# Supplementary material for: Dissecting the bacterial type VI secretion system by a genome wide in silico analysis: what can be learned from available microbial genomic resources?
Source: BMC Genomics. 2009 Mar 12;10:104. doi: 10.1186/1471-2164-10-104 (PMC2660368; doi:10.1186/1471-2164-10-104)
Supplement: Additional file 7 — Detailed description of all identified T6SS gene clusters. Archive containing the detailed description of each identified T6SS locus as an HTML file. [file 1471-2164-10-104-S7.tgz › LociHTML/HTML/CP000038A.html]

Locus CP000038A on Shigella sonnei (strain Ss046) chromosome, complete sequence.

import namespace="svg" implementation="#AdobeSVG"?


# Locus CP000038A

# List of CDS in T6SS locus CP000038A

|  |  |  |  |  |  |  |  |  |
| --- | --- | --- | --- | --- | --- | --- | --- | --- |
| Name | from | to | direct | COG | e-value | COG cover | COG hit start | COG hit end |
| CP000038\_SSON\_0226 | 250465 | 251220 | False | COG0491 | 2e-27 | 92.0 | 19 | 252 |
| CP000038\_SSON\_0227 | 251236 | 251976 | True | COG2226 | 3e-07 | 26.0 | 106 | 168 |
| CP000038\_SSON\_0228 | 251973 | 252440 | False | COG0328 | 1e-55 | 99.0 | 2 | 154 |
| CP000038\_SSON\_0229 | 252505 | 253236 | True | COG0847 | 4e-51 | 95.0 | 8 | 240 |
| CP000038\_SSON\_0231 | 253775 | 254560 | True | - | - | - | - | - |
| CP000038\_SSON\_0232 | 255186 | 255524 | False | - | - | - | - | - |
| CP000038\_SSON\_0233 | 256165 | 256647 | False | COG3157 | 1e-34 | 98.0 | 1 | 159 |
| CP000038\_SSON\_0234 | 256671 | 258023 | False | COG3515 | 1e-55 | 96.0 | 12 | 346 |
| CP000038\_SSON\_0235 | 258034 | 258720 | False | COG3523 | 1e-75 | 19.0 | 957 | 1188 |
| CP000038\_SSON\_0236 | 258757 | 259932 | False | COG3523 | 9e-106 | 34.0 | 539 | 943 |
| CP000038\_SSON\_0237 | 259945 | 260784 | False | COG2801 | 1e-12 | 98.0 | 3 | 230 |
| CP000038\_SSON\_0238 | 260808 | 261173 | False | COG2963 | 2e-09 | 99.0 | 1 | 115 |
| CP000038\_SSON\_0239 | 261215 | 262894 | False | COG3523 | 8e-146 | 45.0 | 2 | 541 |
| CP000038\_SSON\_0240 | 262913 | 264325 | False | COG3515 | 1e-34 | 80.0 | 7 | 284 |
| CP000038\_SSON\_0241 | 264330 | 265022 | False | - | - | - | - | - |
| CP000038\_SSON\_0242 | 265070 | 267529 | False | COG0542 | 2e-110 | 42.0 | 92 | 423 |
| CP000038\_SSON\_0242 | 265070 | 267529 | False | COG0542 | 1e-97 | 51.0 | 380 | 786 |
| CP000038\_SSON\_0243 | 267548 | 267847 | False | - | - | - | - | - |
| CP000038\_SSON\_0244 | 267856 | 268617 | False | COG3455 | 2e-82 | 97.0 | 6 | 260 |
| CP000038\_SSON\_0245 | 268622 | 269410 | False | COG3522 | 1e-97 | 58.0 | 184 | 446 |
| CP000038\_SSON\_0246 | 269403 | 269912 | False | COG3522 | 5e-51 | 37.0 | 13 | 178 |
| CP000038\_SSON\_0247 | 269954 | 270478 | False | COG3521 | 1e-39 | 100.0 | 1 | 159 |
| CP000038\_SSON\_0248 | 270475 | 271755 | False | COG3456 | 4e-119 | 100.0 | 1 | 430 |
| CP000038\_SSON\_0249 | 271780 | 272862 | False | COG3520 | 3e-103 | 99.0 | 1 | 332 |
| CP000038\_SSON\_0250 | 272826 | 274676 | False | COG3519 | 0.0 | 99.0 | 2 | 621 |
| CP000038\_SSON\_0251 | 275100 | 275648 | False | COG3517 | 1e-83 | 36.0 | 311 | 493 |
| CP000038\_SSON\_0252 | 275685 | 276575 | False | COG3517 | 2e-118 | 60.0 | 1 | 297 |
| CP000038\_SSON\_0253 | 276626 | 276850 | False | - | - | - | - | - |
| CP000038\_SSON\_0254 | 276885 | 277385 | False | COG3516 | 2e-49 | 98.0 | 2 | 167 |
| CP000038\_SSON\_0255 | 278082 | 278600 | True | COG3157 | 2e-51 | 98.0 | 1 | 160 |
| CP000038\_SSON\_0256 | 278810 | 280951 | True | COG3501 | 2e-173 | 98.0 | 8 | 550 |
| CP000038\_SSON\_0257 | 281027 | 285235 | True | COG3209 | 4e-105 | 82.0 | 1 | 660 |
| CP000038\_SSON\_0257 | 281027 | 285235 | True | COG3209 | 8e-08 | 53.0 | 223 | 645 |
| CP000038\_SSON\_0258 | 285247 | 285696 | True | - | - | - | - | - |
