# Supplementary material for: Dissecting the bacterial type VI secretion system by a genome wide in silico analysis: what can be learned from available microbial genomic resources?
Source: BMC Genomics. 2009 Mar 12;10:104. doi: 10.1186/1471-2164-10-104 (PMC2660368; doi:10.1186/1471-2164-10-104)
Supplement: Additional file 7 — Detailed description of all identified T6SS gene clusters. Archive containing the detailed description of each identified T6SS locus as an HTML file. [file 1471-2164-10-104-S7.tgz › LociHTML/HTML/CP000058A.html]

Locus CP000058A on Pseudomonas syringae (pathovar phaseolicola, strain 1448A / Race 6) chromosome, complete sequence.

import namespace="svg" implementation="#AdobeSVG"?


# Locus CP000058A

# List of CDS in T6SS locus CP000058A

|  |  |  |  |  |  |  |  |  |
| --- | --- | --- | --- | --- | --- | --- | --- | --- |
| Name | from | to | direct | COG | e-value | COG cover | COG hit start | COG hit end |
| CP000058\_PSPPH\_0116 | 133108 | 134130 | False | COG0790 | 1e-06 | 61.0 | 18 | 196 |
| CP000058\_PSPPH\_0117 | 134132 | 136063 | False | COG1502 | 1e-07 | 29.0 | 165 | 293 |
| CP000058\_PSPPH\_0117 | 134132 | 136063 | False | COG1502 | 3e-11 | 13.0 | 352 | 410 |
| CP000058\_PSPPH\_0118 | 136114 | 136368 | False | COG4104 | 4e-14 | 82.0 | 11 | 91 |
| CP000058\_PSPPH\_0119 | 136714 | 137973 | False | COG3328 | 1e-95 | 97.0 | 10 | 378 |
| CP000058\_PSPPH\_0121 | 138816 | 139919 | False | COG3515 | 2e-17 | 95.0 | 2 | 331 |
| CP000058\_PSPPH\_0122 | 140006 | 140524 | False | COG3157 | 2e-24 | 98.0 | 1 | 160 |
| CP000058\_PSPPH\_0123 | 140680 | 143172 | False | COG2885 | 2e-20 | 92.0 | 13 | 188 |
| CP000058\_PSPPH\_0123 | 140680 | 143172 | False | COG3523 | 1e-84 | 49.0 | 17 | 602 |
| CP000058\_PSPPH\_0124 | 143169 | 144092 | False | COG3913 | 3e-07 | 92.0 | 8 | 216 |
| CP000058\_PSPPH\_0125 | 144124 | 148044 | False | COG3523 | 4e-77 | 47.0 | 5 | 565 |
| CP000058\_PSPPH\_0125 | 144124 | 148044 | False | COG3523 | 1e-47 | 51.0 | 552 | 1166 |
| CP000058\_PSPPH\_0126 | 148075 | 148788 | False | COG3455 | 3e-28 | 83.0 | 39 | 258 |
| CP000058\_PSPPH\_0127 | 148785 | 150128 | False | COG3522 | 4e-91 | 99.0 | 1 | 444 |
| CP000058\_PSPPH\_0128 | 150125 | 150880 | False | COG3521 | 4e-16 | 84.0 | 5 | 139 |
| CP000058\_PSPPH\_0129 | 150906 | 153509 | False | COG0542 | 0.0 | 98.0 | 1 | 777 |
| CP000058\_PSPPH\_0130 | 153506 | 154576 | False | COG3520 | 2e-71 | 96.0 | 1 | 324 |
| CP000058\_PSPPH\_0131 | 154540 | 156372 | False | COG3519 | 2e-148 | 100.0 | 1 | 621 |
| CP000058\_PSPPH\_0132 | 156372 | 156854 | False | COG3518 | 1e-17 | 98.0 | 1 | 154 |
| CP000058\_PSPPH\_0133 | 156880 | 158382 | False | COG3517 | 0.0 | 99.0 | 1 | 494 |
| CP000058\_PSPPH\_0134 | 158397 | 158930 | False | COG3516 | 9e-52 | 99.0 | 2 | 169 |
| CP000058\_PSPPH\_0135 | 158999 | 159883 | False | - | - | - | - | - |
| CP000058\_PSPPH\_0136 | 160913 | 161734 | False | - | - | - | - | - |
| CP000058\_PSPPH\_0138 | 163896 | 164447 | False | - | - | - | - | - |
