# Supplementary material for: Dissecting the bacterial type VI secretion system by a genome wide in silico analysis: what can be learned from available microbial genomic resources?
Source: BMC Genomics. 2009 Mar 12;10:104. doi: 10.1186/1471-2164-10-104 (PMC2660368; doi:10.1186/1471-2164-10-104)
Supplement: Additional file 7 — Detailed description of all identified T6SS gene clusters. Archive containing the detailed description of each identified T6SS locus as an HTML file. [file 1471-2164-10-104-S7.tgz › LociHTML/HTML/CP000075C.html]

Locus CP000075C on Pseudomonas syringae (pathovar syringae, strain B728a) chromosome, complete sequence.

import namespace="svg" implementation="#AdobeSVG"?


# Locus CP000075C

# List of CDS in T6SS locus CP000075C

|  |  |  |  |  |  |  |  |  |
| --- | --- | --- | --- | --- | --- | --- | --- | --- |
| Name | from | to | direct | COG | e-value | COG cover | COG hit start | COG hit end |
| CP000075\_Psyr\_4949 | 5866174 | 5866995 | True | - | - | - | - | - |
| CP000075\_Psyr\_4950 | 5867626 | 5869797 | False | COG0501 | 4e-09 | 51.0 | 143 | 299 |
| CP000075\_Psyr\_4951 | 5869794 | 5870114 | False | - | - | - | - | - |
| CP000075\_Psyr\_4952 | 5870498 | 5871247 | True | - | - | - | - | - |
| CP000075\_Psyr\_4953 | 5871317 | 5871850 | True | COG3516 | 3e-52 | 99.0 | 2 | 169 |
| CP000075\_Psyr\_4954 | 5871865 | 5873367 | True | COG3517 | 0.0 | 99.0 | 1 | 494 |
| CP000075\_Psyr\_4955 | 5873395 | 5873877 | True | COG3518 | 6e-18 | 98.0 | 1 | 154 |
| CP000075\_Psyr\_4956 | 5873877 | 5875709 | True | COG3519 | 7e-149 | 100.0 | 1 | 621 |
| CP000075\_Psyr\_4957 | 5875673 | 5876743 | True | COG3520 | 1e-71 | 96.0 | 1 | 324 |
| CP000075\_Psyr\_4958 | 5876740 | 5879343 | True | COG0542 | 0.0 | 98.0 | 1 | 777 |
| CP000075\_Psyr\_4959 | 5879369 | 5880124 | True | COG3521 | 7e-16 | 84.0 | 5 | 139 |
| CP000075\_Psyr\_4960 | 5880121 | 5881464 | True | COG3522 | 2e-91 | 99.0 | 1 | 444 |
| CP000075\_Psyr\_4961 | 5881461 | 5882174 | True | COG3455 | 3e-28 | 83.0 | 39 | 258 |
| CP000075\_Psyr\_4962 | 5882205 | 5886137 | True | COG3523 | 6e-75 | 47.0 | 5 | 565 |
| CP000075\_Psyr\_4962 | 5882205 | 5886137 | True | COG3523 | 1e-48 | 51.0 | 552 | 1166 |
| CP000075\_Psyr\_4963 | 5886169 | 5887092 | True | COG3913 | 7e-07 | 59.0 | 8 | 142 |
| CP000075\_Psyr\_4964 | 5887089 | 5889581 | True | COG2885 | 3e-20 | 92.0 | 13 | 188 |
| CP000075\_Psyr\_4964 | 5887089 | 5889581 | True | COG3523 | 2e-86 | 49.0 | 17 | 602 |
| CP000075\_Psyr\_4965 | 5889737 | 5890255 | True | COG3157 | 3e-24 | 98.0 | 1 | 160 |
| CP000075\_Psyr\_4966 | 5890348 | 5891442 | True | COG3515 | 2e-17 | 95.0 | 2 | 331 |
| CP000075\_Psyr\_4967 | 5891620 | 5892045 | True | - | - | - | - | - |
| CP000075\_Psyr\_4968 | 5892198 | 5892530 | True | - | - | - | - | - |
| CP000075\_Psyr\_4969 | 5892565 | 5892873 | True | COG4104 | 1e-15 | 82.0 | 11 | 91 |
| CP000075\_Psyr\_4970 | 5892924 | 5894978 | True | COG1502 | 1e-08 | 44.0 | 97 | 293 |
| CP000075\_Psyr\_4970 | 5892924 | 5894978 | True | COG1502 | 4e-11 | 17.0 | 352 | 429 |
| CP000075\_Psyr\_4971 | 5895050 | 5896006 | True | COG0790 | 3e-07 | 57.0 | 5 | 173 |
| CP000075\_Psyr\_4972 | 5896106 | 5897137 | True | COG0790 | 4e-08 | 85.0 | 19 | 268 |
| CP000075\_Psyr\_4973 | 5897201 | 5898283 | True | - | - | - | - | - |
| CP000075\_Psyr\_4974 | 5898317 | 5901193 | True | COG4253 | 2e-48 | 100.0 | 1 | 278 |
| CP000075\_Psyr\_4974 | 5898317 | 5901193 | True | COG3501 | 3e-106 | 94.0 | 6 | 527 |
| CP000075\_Psyr\_4975 | 5901334 | 5902053 | True | - | - | - | - | - |
| CP000075\_Psyr\_4976 | 5902118 | 5902672 | False | COG0317 | 9e-22 | 24.0 | 27 | 195 |
| CP000075\_Psyr\_4977 | 5903248 | 5904258 | False | COG1397 | 8e-39 | 98.0 | 4 | 311 |
| CP000075\_Psyr\_4978 | 5904412 | 5904711 | False | - | - | - | - | - |
| CP000075\_Psyr\_4979 | 5904880 | 5905437 | False | - | - | - | - | - |
| CP000075\_Psyr\_4980 | 5905568 | 5905888 | False | - | - | - | - | - |
| CP000075\_Psyr\_4981 | 5906424 | 5907503 | True | - | - | - | - | - |
| CP000075\_Psyr\_4982 | 5907500 | 5908228 | True | - | - | - | - | - |
| CP000075\_Psyr\_4983 | 5908301 | 5910145 | True | COG3501 | 5e-144 | 98.0 | 2 | 542 |
| CP000075\_Psyr\_4984 | 5910157 | 5910738 | True | - | - | - | - | - |
| CP000075\_Psyr\_4985 | 5910773 | 5911240 | True | COG5435 | 4e-22 | 99.0 | 2 | 147 |
| CP000075\_Psyr\_4986 | 5911246 | 5915475 | True | COG3209 | 1e-38 | 83.0 | 3 | 668 |
| CP000075\_Psyr\_4986 | 5911246 | 5915475 | True | COG4104 | 1e-09 | 76.0 | 24 | 98 |
