# Supplementary material for: Dissecting the bacterial type VI secretion system by a genome wide in silico analysis: what can be learned from available microbial genomic resources?
Source: BMC Genomics. 2009 Mar 12;10:104. doi: 10.1186/1471-2164-10-104 (PMC2660368; doi:10.1186/1471-2164-10-104)
Supplement: Additional file 7 — Detailed description of all identified T6SS gene clusters. Archive containing the detailed description of each identified T6SS locus as an HTML file. [file 1471-2164-10-104-S7.tgz › LociHTML/HTML/CP000076D.html]

Locus CP000076D on Pseudomonas fluorescens (strain Pf-5 / ATCC BAA-477) chromosome, complete sequence.

import namespace="svg" implementation="#AdobeSVG"?


# Locus CP000076D

# List of CDS in T6SS locus CP000076D

|  |  |  |  |  |  |  |  |  |
| --- | --- | --- | --- | --- | --- | --- | --- | --- |
| Name | from | to | direct | COG | e-value | COG cover | COG hit start | COG hit end |
| CP000076\_PFL\_6076 | 6897920 | 6899122 | False | COG4591 | 2e-12 | 99.0 | 3 | 408 |
| CP000076\_PFL\_6077 | 6899122 | 6899838 | False | COG1136 | 3e-45 | 91.0 | 1 | 207 |
| CP000076\_PFL\_6078 | 6899838 | 6902867 | False | COG0515 | 5e-40 | 91.0 | 13 | 363 |
| CP000076\_PFL\_6079 | 6902924 | 6903658 | False | COG0631 | 1e-49 | 93.0 | 6 | 250 |
| CP000076\_PFL\_6080 | 6903659 | 6904312 | False | COG3913 | 1e-41 | 92.0 | 5 | 214 |
| CP000076\_PFL\_6081 | 6904309 | 6907815 | False | COG3523 | 0.0 | 99.0 | 9 | 1187 |
| CP000076\_PFL\_6082 | 6907812 | 6909137 | False | COG3455 | 3e-67 | 100.0 | 1 | 262 |
| CP000076\_PFL\_6082 | 6907812 | 6909137 | False | COG1360 | 1e-29 | 58.0 | 103 | 244 |
| CP000076\_PFL\_6083 | 6909144 | 6910478 | False | COG3522 | 3e-149 | 100.0 | 1 | 446 |
| CP000076\_PFL\_6084 | 6910494 | 6911000 | False | COG3521 | 4e-28 | 96.0 | 7 | 159 |
| CP000076\_PFL\_6085 | 6911065 | 6912549 | False | COG3456 | 4e-76 | 100.0 | 1 | 430 |
| CP000076\_PFL\_6086 | 6912954 | 6913961 | True | COG3515 | 4e-27 | 97.0 | 6 | 343 |
| CP000076\_PFL\_6087 | 6914043 | 6914558 | True | COG3516 | 1e-56 | 98.0 | 3 | 168 |
| CP000076\_PFL\_6088 | 6914575 | 6916068 | True | COG3517 | 0.0 | 99.0 | 4 | 495 |
| CP000076\_PFL\_6089 | 6916187 | 6916675 | True | COG3157 | 3e-34 | 98.0 | 1 | 159 |
| CP000076\_PFL\_6090 | 6916803 | 6917351 | True | COG3518 | 9e-28 | 96.0 | 2 | 153 |
| CP000076\_PFL\_6091 | 6917348 | 6919207 | True | COG3519 | 0.0 | 100.0 | 1 | 621 |
| CP000076\_PFL\_6092 | 6919171 | 6920223 | True | COG3520 | 1e-89 | 99.0 | 1 | 334 |
| CP000076\_PFL\_6093 | 6920216 | 6922891 | True | COG0542 | 0.0 | 99.0 | 1 | 785 |
| CP000076\_PFL\_6094 | 6923022 | 6924959 | True | COG3501 | 1e-170 | 98.0 | 4 | 547 |
| CP000076\_PFL\_6095 | 6924978 | 6925403 | True | COG5435 | 1e-08 | 95.0 | 2 | 141 |
| CP000076\_PFL\_6096 | 6925413 | 6929873 | True | COG3209 | 7e-38 | 82.0 | 8 | 661 |
| CP000076\_PFL\_6097 | 6929889 | 6930515 | True | - | - | - | - | - |
