# Supplementary material for: Dissecting the bacterial type VI secretion system by a genome wide in silico analysis: what can be learned from available microbial genomic resources?
Source: BMC Genomics. 2009 Mar 12;10:104. doi: 10.1186/1471-2164-10-104 (PMC2660368; doi:10.1186/1471-2164-10-104)
Supplement: Additional file 7 — Detailed description of all identified T6SS gene clusters. Archive containing the detailed description of each identified T6SS locus as an HTML file. [file 1471-2164-10-104-S7.tgz › LociHTML/HTML/CP000085A.html]

Locus CP000085A on Burkholderia thailandensis (strain E264 / ATCC 700388 / DSM 13276 / CIP 106301) chromosome II, complete sequence.

import namespace="svg" implementation="#AdobeSVG"?


# Locus CP000085A

# List of CDS in T6SS locus CP000085A

|  |  |  |  |  |  |  |  |  |
| --- | --- | --- | --- | --- | --- | --- | --- | --- |
| Name | from | to | direct | COG | e-value | COG cover | COG hit start | COG hit end |
| CP000085\_BTH\_II0115 | 132907 | 133461 | True | COG3539 | 2e-13 | 100.0 | 1 | 184 |
| CP000085\_BTH\_II0116 | 133543 | 134268 | True | COG3121 | 3e-58 | 96.0 | 9 | 234 |
| CP000085\_BTH\_II0117 | 134305 | 137124 | True | COG3188 | 0.0 | 96.0 | 16 | 821 |
| CP000085\_BTH\_II0118 | 137144 | 137689 | True | COG3539 | 4e-14 | 95.0 | 7 | 182 |
| CP000085\_BTH\_II0119 | 137720 | 138409 | True | COG3455 | 1e-07 | 68.0 | 68 | 247 |
| CP000085\_BTH\_II0120 | 138412 | 140088 | True | COG2885 | 3e-25 | 65.0 | 66 | 190 |
| CP000085\_BTH\_II0121 | 140432 | 140971 | True | COG3516 | 2e-59 | 99.0 | 2 | 169 |
| CP000085\_BTH\_II0122 | 141005 | 142504 | True | COG3517 | 0.0 | 100.0 | 1 | 495 |
| CP000085\_BTH\_II0123 | 142705 | 143187 | True | COG3157 | 8e-36 | 99.0 | 1 | 161 |
| CP000085\_BTH\_II0124 | 143315 | 143857 | True | COG3521 | 2e-26 | 90.0 | 6 | 149 |
| CP000085\_BTH\_II0125 | 143770 | 145212 | True | COG3522 | 4e-135 | 99.0 | 1 | 445 |
| CP000085\_BTH\_II0126 | 145209 | 146510 | True | COG3455 | 7e-49 | 88.0 | 30 | 260 |
| CP000085\_BTH\_II0126 | 145209 | 146510 | True | COG1360 | 1e-26 | 65.0 | 81 | 241 |
| CP000085\_BTH\_II0127 | 146525 | 150433 | True | COG3523 | 0.0 | 99.0 | 2 | 1187 |
| CP000085\_BTH\_II0128 | 150603 | 151172 | True | - | - | - | - | - |
| CP000085\_BTH\_II0129 | 151273 | 153891 | True | COG3501 | 7e-134 | 95.0 | 24 | 548 |
| CP000085\_BTH\_II0129 | 151273 | 153891 | True | COG3889 | 2e-13 | 19.0 | 678 | 850 |
| CP000085\_BTH\_II0130 | 153966 | 154235 | True | COG4104 | 1e-06 | 89.0 | 10 | 97 |
| CP000085\_BTH\_II0131 | 154248 | 157700 | True | COG3209 | 2e-21 | 79.0 | 20 | 651 |
| CP000085\_BTH\_II0132 | 157593 | 158975 | False | - | - | - | - | - |
| CP000085\_BTH\_II0133 | 158984 | 160117 | False | - | - | - | - | - |
| CP000085\_BTH\_II0134 | 160068 | 161138 | False | COG3515 | 9e-21 | 97.0 | 2 | 339 |
| CP000085\_BTH\_II0135 | 161157 | 162206 | False | COG3520 | 4e-82 | 97.0 | 9 | 334 |
| CP000085\_BTH\_II0136 | 162203 | 164083 | False | COG3519 | 0.0 | 100.0 | 1 | 621 |
| CP000085\_BTH\_II0137 | 164085 | 164603 | False | COG3518 | 1e-20 | 95.0 | 8 | 157 |
| CP000085\_BTH\_II0138 | 164590 | 165444 | False | COG4455 | 5e-60 | 95.0 | 12 | 273 |
| CP000085\_BTH\_II0139 | 165428 | 166483 | False | - | - | - | - | - |
| CP000085\_BTH\_II0140 | 166857 | 169613 | True | COG0542 | 0.0 | 98.0 | 1 | 775 |
| CP000085\_BTH\_II0141 | 170006 | 172864 | False | COG2204 | 1e-17 | 33.0 | 1 | 157 |
| CP000085\_BTH\_II0141 | 170006 | 172864 | False | COG0642 | 3e-41 | 82.0 | 57 | 333 |
