# Supplementary material for: Dissecting the bacterial type VI secretion system by a genome wide in silico analysis: what can be learned from available microbial genomic resources?
Source: BMC Genomics. 2009 Mar 12;10:104. doi: 10.1186/1471-2164-10-104 (PMC2660368; doi:10.1186/1471-2164-10-104)
Supplement: Additional file 7 — Detailed description of all identified T6SS gene clusters. Archive containing the detailed description of each identified T6SS locus as an HTML file. [file 1471-2164-10-104-S7.tgz › LociHTML/HTML/CP000085B.html]

Locus CP000085B on Burkholderia thailandensis (strain E264 / ATCC 700388 / DSM 13276 / CIP 106301) chromosome II, complete sequence.

import namespace="svg" implementation="#AdobeSVG"?


# Locus CP000085B

# List of CDS in T6SS locus CP000085B

|  |  |  |  |  |  |  |  |  |
| --- | --- | --- | --- | --- | --- | --- | --- | --- |
| Name | from | to | direct | COG | e-value | COG cover | COG hit start | COG hit end |
| CP000085\_BTH\_II0245 | 297421 | 298755 | True | COG3048 | 2e-123 | 99.0 | 1 | 441 |
| CP000085\_BTH\_II0246 | 299015 | 299911 | False | COG0583 | 2e-32 | 99.0 | 1 | 296 |
| CP000085\_BTH\_II0247 | 300115 | 300819 | True | COG1028 | 2e-30 | 98.0 | 6 | 251 |
| CP000085\_BTH\_II0248 | 301071 | 303068 | True | - | - | - | - | - |
| CP000085\_BTH\_II0249 | 303044 | 304426 | False | COG3456 | 1e-28 | 93.0 | 23 | 425 |
| CP000085\_BTH\_II0250 | 304544 | 304942 | False | - | - | - | - | - |
| CP000085\_BTH\_II0251 | 305598 | 306116 | True | COG3521 | 3e-26 | 90.0 | 1 | 144 |
| CP000085\_BTH\_II0252 | 306113 | 307519 | True | COG3522 | 2e-127 | 99.0 | 3 | 446 |
| CP000085\_BTH\_II0253 | 307535 | 308848 | True | COG3455 | 5e-59 | 97.0 | 4 | 258 |
| CP000085\_BTH\_II0253 | 307535 | 308848 | True | COG1360 | 1e-27 | 56.0 | 103 | 240 |
| CP000085\_BTH\_II0254 | 308851 | 312480 | True | COG3523 | 0.0 | 99.0 | 7 | 1185 |
| CP000085\_BTH\_II0255 | 312486 | 313352 | True | COG3913 | 2e-12 | 63.0 | 2 | 146 |
| CP000085\_BTH\_II0256 | 313510 | 316143 | True | COG0515 | 2e-29 | 72.0 | 1 | 277 |
| CP000085\_BTH\_II0257 | 316196 | 317260 | True | COG3515 | 1e-21 | 97.0 | 6 | 341 |
| CP000085\_BTH\_II0258 | 317322 | 317903 | True | COG3516 | 9e-54 | 97.0 | 2 | 166 |
| CP000085\_BTH\_II0259 | 317896 | 319404 | True | COG3517 | 0.0 | 100.0 | 1 | 495 |
| CP000085\_BTH\_II0260 | 319462 | 319953 | True | COG3157 | 5e-18 | 98.0 | 1 | 159 |
| CP000085\_BTH\_II0261 | 319972 | 320532 | True | COG3518 | 7e-19 | 94.0 | 1 | 149 |
| CP000085\_BTH\_II0262 | 320537 | 322408 | True | COG3519 | 1e-164 | 100.0 | 1 | 621 |
| CP000085\_BTH\_II0263 | 322405 | 323688 | True | COG3520 | 3e-62 | 94.0 | 3 | 318 |
| CP000085\_BTH\_II0264 | 323701 | 326358 | True | COG0542 | 0.0 | 100.0 | 1 | 786 |
| CP000085\_BTH\_II0265 | 326364 | 328559 | True | COG3501 | 4e-143 | 96.0 | 23 | 550 |
| CP000085\_BTH\_II0266 | 328575 | 329840 | True | - | - | - | - | - |
| CP000085\_BTH\_II0267 | 329863 | 334482 | True | COG3209 | 4e-27 | 64.0 | 4 | 514 |
