# Supplementary material for: Dissecting the bacterial type VI secretion system by a genome wide in silico analysis: what can be learned from available microbial genomic resources?
Source: BMC Genomics. 2009 Mar 12;10:104. doi: 10.1186/1471-2164-10-104 (PMC2660368; doi:10.1186/1471-2164-10-104)
Supplement: Additional file 7 — Detailed description of all identified T6SS gene clusters. Archive containing the detailed description of each identified T6SS locus as an HTML file. [file 1471-2164-10-104-S7.tgz › LociHTML/HTML/CP000085C.html]

Locus CP000085C on Burkholderia thailandensis (strain E264 / ATCC 700388 / DSM 13276 / CIP 106301) chromosome II, complete sequence.

import namespace="svg" implementation="#AdobeSVG"?


# Locus CP000085C

# List of CDS in T6SS locus CP000085C

|  |  |  |  |  |  |  |  |  |
| --- | --- | --- | --- | --- | --- | --- | --- | --- |
| Name | from | to | direct | COG | e-value | COG cover | COG hit start | COG hit end |
| CP000085\_BTH\_II0849 | 991240 | 992778 | True | - | - | - | - | - |
| CP000085\_BTH\_II0850 | 992960 | 993415 | True | - | - | - | - | - |
| CP000085\_BTH\_II0851 | 993650 | 994321 | True | COG2197 | 3e-44 | 99.0 | 2 | 211 |
| CP000085\_BTH\_II0852 | 994546 | 994989 | True | - | - | - | - | - |
| CP000085\_BTH\_II0853 | 995098 | 996285 | True | COG2207 | 3e-13 | 99.0 | 2 | 127 |
| CP000085\_BTH\_II0854 | 996276 | 997595 | False | - | - | - | - | - |
| CP000085\_BTH\_II0855 | 997685 | 1001575 | False | COG3523 | 6e-44 | 88.0 | 5 | 1060 |
| CP000085\_BTH\_II0856 | 1001572 | 1002252 | False | COG3455 | 6e-21 | 82.0 | 41 | 256 |
| CP000085\_BTH\_II0857 | 1002249 | 1003643 | False | COG3522 | 3e-43 | 98.0 | 5 | 445 |
| CP000085\_BTH\_II0858 | 1003676 | 1004482 | False | - | - | - | - | - |
| CP000085\_BTH\_II0859 | 1004492 | 1004851 | False | - | - | - | - | - |
| CP000085\_BTH\_II0860 | 1004913 | 1005707 | False | - | - | - | - | - |
| CP000085\_BTH\_II0861 | 1005686 | 1006750 | False | COG1357 | 2e-17 | 94.0 | 12 | 236 |
| CP000085\_BTH\_II0862 | 1006768 | 1009386 | False | COG1357 | 3e-21 | 92.0 | 17 | 237 |
| CP000085\_BTH\_II0863 | 1009425 | 1012463 | False | COG3501 | 8e-80 | 82.0 | 9 | 463 |
| CP000085\_BTH\_II0864 | 1012489 | 1015365 | False | COG0542 | 2e-123 | 54.0 | 1 | 427 |
| CP000085\_BTH\_II0864 | 1012489 | 1015365 | False | COG0542 | 6e-99 | 45.0 | 420 | 775 |
| CP000085\_BTH\_II0865 | 1015352 | 1016407 | False | COG3520 | 2e-42 | 99.0 | 4 | 335 |
| CP000085\_BTH\_II0866 | 1016371 | 1018113 | False | COG3519 | 1e-96 | 99.0 | 4 | 621 |
| CP000085\_BTH\_II0867 | 1018142 | 1018618 | False | COG3518 | 6e-10 | 88.0 | 2 | 140 |
| CP000085\_BTH\_II0868 | 1018597 | 1019085 | False | COG3157 | 5e-35 | 97.0 | 1 | 158 |
| CP000085\_BTH\_II0869 | 1019268 | 1020794 | False | COG3517 | 0.0 | 97.0 | 10 | 492 |
| CP000085\_BTH\_II0870 | 1020791 | 1021285 | False | COG3516 | 1e-37 | 94.0 | 8 | 166 |
| CP000085\_BTH\_II0871 | 1021668 | 1023368 | True | COG0642 | 2e-25 | 72.0 | 95 | 336 |
| CP000085\_BTH\_II0872 | 1023365 | 1024099 | True | COG0745 | 3e-51 | 99.0 | 3 | 229 |
| CP000085\_BTH\_II0873 | 1024090 | 1025886 | True | COG3515 | 3e-07 | 30.0 | 19 | 124 |
| CP000085\_BTH\_II0874 | 1026177 | 1026500 | False | - | - | - | - | - |
