# Supplementary material for: Dissecting the bacterial type VI secretion system by a genome wide in silico analysis: what can be learned from available microbial genomic resources?
Source: BMC Genomics. 2009 Mar 12;10:104. doi: 10.1186/1471-2164-10-104 (PMC2660368; doi:10.1186/1471-2164-10-104)
Supplement: Additional file 7 — Detailed description of all identified T6SS gene clusters. Archive containing the detailed description of each identified T6SS locus as an HTML file. [file 1471-2164-10-104-S7.tgz › LociHTML/HTML/CP000085E.html]

Locus CP000085E on Burkholderia thailandensis (strain E264 / ATCC 700388 / DSM 13276 / CIP 106301) chromosome II, complete sequence.

import namespace="svg" implementation="#AdobeSVG"?


# Locus CP000085E

# List of CDS in T6SS locus CP000085E

|  |  |  |  |  |  |  |  |  |
| --- | --- | --- | --- | --- | --- | --- | --- | --- |
| Name | from | to | direct | COG | e-value | COG cover | COG hit start | COG hit end |
| CP000085\_BTH\_II1879 | 2276153 | 2277724 | False | COG2814 | 4e-15 | 50.0 | 1 | 198 |
| CP000085\_BTH\_II1880 | 2277721 | 2279148 | False | COG1819 | 2e-39 | 98.0 | 1 | 399 |
| CP000085\_BTH\_II1881 | 2279278 | 2280177 | False | - | - | - | - | - |
| CP000085\_BTH\_II1882 | 2280750 | 2281121 | False | - | - | - | - | - |
| CP000085\_BTH\_II1883 | 2281342 | 2281767 | False | - | - | - | - | - |
| CP000085\_BTH\_II1884 | 2281790 | 2282149 | False | - | - | - | - | - |
| CP000085\_BTH\_II1885 | 2282208 | 2285702 | False | COG3523 | 7e-140 | 98.0 | 17 | 1185 |
| CP000085\_BTH\_II1886 | 2285699 | 2287483 | False | COG3455 | 4e-26 | 98.0 | 1 | 257 |
| CP000085\_BTH\_II1886 | 2285699 | 2287483 | False | COG1360 | 3e-19 | 50.0 | 123 | 244 |
| CP000085\_BTH\_II1887 | 2287509 | 2288870 | False | COG3522 | 6e-91 | 98.0 | 7 | 446 |
| CP000085\_BTH\_II1888 | 2288867 | 2289346 | False | - | - | - | - | - |
| CP000085\_BTH\_II1889 | 2289466 | 2289855 | False | - | - | - | - | - |
| CP000085\_BTH\_II1890 | 2289895 | 2290611 | False | - | - | - | - | - |
| CP000085\_BTH\_II1891 | 2290614 | 2291684 | False | COG1357 | 7e-08 | 66.0 | 32 | 190 |
| CP000085\_BTH\_II1892 | 2291684 | 2293834 | False | COG1357 | 2e-08 | 61.0 | 58 | 204 |
| CP000085\_BTH\_II1893 | 2293904 | 2296192 | False | COG3501 | 3e-136 | 97.0 | 10 | 544 |
| CP000085\_BTH\_II1894 | 2296361 | 2298652 | False | COG3501 | 6e-137 | 96.0 | 10 | 537 |
| CP000085\_BTH\_II1895 | 2298643 | 2301486 | False | COG0542 | 0.0 | 97.0 | 2 | 766 |
| CP000085\_BTH\_II1896 | 2301489 | 2302478 | False | COG3520 | 9e-35 | 93.0 | 15 | 328 |
| CP000085\_BTH\_II1897 | 2302475 | 2304337 | False | COG3519 | 2e-88 | 96.0 | 3 | 604 |
| CP000085\_BTH\_II1898 | 2304351 | 2304782 | False | - | - | - | - | - |
| CP000085\_BTH\_II1899 | 2304861 | 2305388 | False | COG3157 | 6e-27 | 98.0 | 1 | 160 |
| CP000085\_BTH\_II1900 | 2305532 | 2307037 | False | COG3517 | 0.0 | 100.0 | 1 | 495 |
| CP000085\_BTH\_II1901 | 2307040 | 2307591 | False | COG3516 | 2e-47 | 97.0 | 5 | 169 |
| CP000085\_BTH\_II1902 | 2307625 | 2308569 | False | COG3515 | 4e-13 | 82.0 | 54 | 339 |
| CP000085\_BTH\_II1903 | 2310190 | 2312109 | False | COG0427 | 3e-163 | 100.0 | 1 | 501 |
| CP000085\_BTH\_II1904 | 2312067 | 2312762 | True | COG0558 | 3e-13 | 96.0 | 2 | 187 |
| CP000085\_BTH\_II1905 | 2312759 | 2313532 | True | - | - | - | - | - |
| CP000085\_BTH\_II1906 | 2313544 | 2314530 | True | - | - | - | - | - |
