# Supplementary material for: Dissecting the bacterial type VI secretion system by a genome wide in silico analysis: what can be learned from available microbial genomic resources?
Source: BMC Genomics. 2009 Mar 12;10:104. doi: 10.1186/1471-2164-10-104 (PMC2660368; doi:10.1186/1471-2164-10-104)
Supplement: Additional file 7 — Detailed description of all identified T6SS gene clusters. Archive containing the detailed description of each identified T6SS locus as an HTML file. [file 1471-2164-10-104-S7.tgz › LociHTML/HTML/CP000086H.html]

Locus CP000086H on Burkholderia thailandensis (strain E264 / ATCC 700388 / DSM 13276 / CIP 106301) chromosome I, complete sequence.

import namespace="svg" implementation="#AdobeSVG"?


# Locus CP000086H

# List of CDS in T6SS locus CP000086H

|  |  |  |  |  |  |  |  |  |
| --- | --- | --- | --- | --- | --- | --- | --- | --- |
| Name | from | to | direct | COG | e-value | COG cover | COG hit start | COG hit end |
| CP000086\_BTH\_I2949 | 3390116 | 3392428 | True | COG2274 | 0.0 | 98.0 | 1 | 696 |
| CP000086\_BTH\_I2950 | 3392434 | 3393873 | True | COG1538 | 3e-42 | 87.0 | 45 | 443 |
| CP000086\_BTH\_I2951 | 3394070 | 3395035 | False | - | - | - | - | - |
| CP000086\_BTH\_I2952 | 3395047 | 3396012 | True | - | - | - | - | - |
| CP000086\_BTH\_I2953 | 3396175 | 3396273 | True | - | - | - | - | - |
| CP000086\_BTH\_I2954 | 3396298 | 3400203 | True | COG3523 | 0.0 | 100.0 | 1 | 1188 |
| CP000086\_BTH\_I2955 | 3400200 | 3401189 | True | COG3913 | 8e-37 | 93.0 | 5 | 216 |
| CP000086\_BTH\_I2956 | 3401194 | 3402129 | True | COG2885 | 4e-26 | 84.0 | 27 | 186 |
| CP000086\_BTH\_I2957 | 3402329 | 3403438 | False | COG3515 | 8e-32 | 98.0 | 7 | 346 |
| CP000086\_BTH\_I2958 | 3403545 | 3406208 | False | COG0542 | 0.0 | 97.0 | 1 | 770 |
| CP000086\_BTH\_I2959 | 3406242 | 3407342 | False | COG3520 | 2e-61 | 99.0 | 1 | 332 |
| CP000086\_BTH\_I2960 | 3407306 | 3409144 | False | COG3519 | 3e-148 | 99.0 | 2 | 620 |
| CP000086\_BTH\_I2961 | 3409225 | 3409707 | False | COG3518 | 2e-34 | 98.0 | 4 | 157 |
| CP000086\_BTH\_I2962 | 3409765 | 3410268 | False | COG3157 | 3e-34 | 97.0 | 5 | 162 |
| CP000086\_BTH\_I2963 | 3410341 | 3411831 | False | COG3517 | 0.0 | 99.0 | 2 | 495 |
| CP000086\_BTH\_I2964 | 3411848 | 3412366 | False | COG3516 | 2e-47 | 99.0 | 2 | 169 |
| CP000086\_BTH\_I2965 | 3412403 | 3413092 | False | - | - | - | - | - |
| CP000086\_BTH\_I2966 | 3413202 | 3414080 | True | COG3521 | 2e-27 | 92.0 | 1 | 147 |
| CP000086\_BTH\_I2967 | 3414189 | 3415535 | True | COG3522 | 5e-111 | 100.0 | 1 | 446 |
| CP000086\_BTH\_I2968 | 3415532 | 3416317 | True | COG3455 | 4e-46 | 95.0 | 14 | 262 |
| CP000086\_BTH\_I2971 | 3416885 | 3417079 | True | - | - | - | - | - |
| CP000086\_BTH\_I2973 | 3417661 | 3418173 | False | COG2969 | 7e-42 | 98.0 | 3 | 155 |
| CP000086\_BTH\_I2974 | 3418243 | 3418854 | False | COG0625 | 1e-29 | 95.0 | 1 | 201 |
| CP000086\_BTH\_I2975 | 3418947 | 3419705 | False | COG2857 | 2e-56 | 100.0 | 1 | 250 |
| CP000086\_BTH\_I2976 | 3419728 | 3421110 | False | COG1290 | 1e-89 | 96.0 | 5 | 372 |
| CP000086\_BTH\_I2977 | 3421111 | 3421731 | False | COG0723 | 1e-29 | 96.0 | 4 | 173 |
