# Supplementary material for: Dissecting the bacterial type VI secretion system by a genome wide in silico analysis: what can be learned from available microbial genomic resources?
Source: BMC Genomics. 2009 Mar 12;10:104. doi: 10.1186/1471-2164-10-104 (PMC2660368; doi:10.1186/1471-2164-10-104)
Supplement: Additional file 7 — Detailed description of all identified T6SS gene clusters. Archive containing the detailed description of each identified T6SS locus as an HTML file. [file 1471-2164-10-104-S7.tgz › LociHTML/HTML/CP000089A.html]

Locus CP000089A on Dechloromonas aromatica (strain RCB) chromosome, complete sequence.

import namespace="svg" implementation="#AdobeSVG"?


# Locus CP000089A

# List of CDS in T6SS locus CP000089A

|  |  |  |  |  |  |  |  |  |
| --- | --- | --- | --- | --- | --- | --- | --- | --- |
| Name | from | to | direct | COG | e-value | COG cover | COG hit start | COG hit end |
| CP000089\_Daro\_2162 | 2334288 | 2335715 | True | COG1538 | 1e-65 | 98.0 | 1 | 449 |
| CP000089\_Daro\_2163 | 2335809 | 2336441 | False | - | - | - | - | - |
| CP000089\_Daro\_2165 | 2336665 | 2338053 | False | COG3673 | 6e-08 | 63.0 | 33 | 299 |
| CP000089\_Daro\_2166 | 2338053 | 2338604 | False | - | - | - | - | - |
| CP000089\_Daro\_2167 | 2338608 | 2338874 | False | - | - | - | - | - |
| CP000089\_Daro\_2168 | 2338874 | 2339767 | False | - | - | - | - | - |
| CP000089\_Daro\_2169 | 2339770 | 2342571 | False | COG4253 | 2e-31 | 79.0 | 1 | 220 |
| CP000089\_Daro\_2169 | 2339770 | 2342571 | False | COG3501 | 8e-103 | 99.0 | 3 | 549 |
| CP000089\_Daro\_2170 | 2342699 | 2343172 | False | - | - | - | - | - |
| CP000089\_Daro\_2171 | 2343212 | 2345911 | False | COG0542 | 0.0 | 99.0 | 1 | 784 |
| CP000089\_Daro\_2172 | 2345929 | 2346993 | False | COG3520 | 8e-64 | 97.0 | 1 | 327 |
| CP000089\_Daro\_2173 | 2346957 | 2348789 | False | COG3519 | 3e-139 | 99.0 | 2 | 620 |
| CP000089\_Daro\_2174 | 2348790 | 2349269 | False | COG3518 | 4e-26 | 97.0 | 3 | 155 |
| CP000089\_Daro\_2175 | 2349318 | 2349854 | False | COG3157 | 2e-26 | 97.0 | 2 | 159 |
| CP000089\_Daro\_2176 | 2349887 | 2351371 | False | COG3517 | 0.0 | 99.0 | 4 | 494 |
| CP000089\_Daro\_2177 | 2351368 | 2351880 | False | COG3516 | 9e-47 | 98.0 | 2 | 167 |
| CP000089\_Daro\_2178 | 2351961 | 2352578 | False | - | - | - | - | - |
| CP000089\_Daro\_2179 | 2352804 | 2353373 | True | COG3521 | 5e-13 | 80.0 | 7 | 134 |
| CP000089\_Daro\_2180 | 2353386 | 2354729 | True | COG3522 | 6e-90 | 99.0 | 2 | 445 |
| CP000089\_Daro\_2181 | 2354726 | 2355499 | True | COG3455 | 6e-37 | 90.0 | 21 | 258 |
| CP000089\_Daro\_2182 | 2355510 | 2358245 | True | COG4253 | 2e-44 | 98.0 | 1 | 273 |
| CP000089\_Daro\_2182 | 2355510 | 2358245 | True | COG3501 | 3e-105 | 96.0 | 5 | 535 |
| CP000089\_Daro\_2183 | 2358220 | 2358942 | True | - | - | - | - | - |
| CP000089\_Daro\_2184 | 2358939 | 2359379 | True | - | - | - | - | - |
| CP000089\_Daro\_2185 | 2359672 | 2360769 | True | COG3515 | 3e-32 | 99.0 | 4 | 346 |
| CP000089\_Daro\_2186 | 2360816 | 2361601 | False | COG2885 | 1e-24 | 85.0 | 26 | 187 |
| CP000089\_Daro\_2187 | 2361598 | 2362581 | False | COG3913 | 3e-18 | 90.0 | 11 | 216 |
| CP000089\_Daro\_2188 | 2362578 | 2366390 | False | COG3523 | 0.0 | 98.0 | 7 | 1171 |
| CP000089\_Daro\_2189 | 2366412 | 2367833 | False | - | - | - | - | - |
| CP000089\_Daro\_2190 | 2367853 | 2368119 | False | COG4104 | 6e-16 | 84.0 | 10 | 92 |
| CP000089\_Daro\_2191 | 2368163 | 2369011 | False | - | - | - | - | - |
| CP000089\_Daro\_2192 | 2369211 | 2369801 | True | - | - | - | - | - |
| CP000089\_Daro\_2193 | 2369943 | 2370602 | False | COG0666 | 1e-13 | 64.0 | 63 | 214 |
| CP000089\_Daro\_2194 | 2370608 | 2371381 | False | COG0084 | 5e-87 | 99.0 | 2 | 255 |
