# Supplementary material for: Dissecting the bacterial type VI secretion system by a genome wide in silico analysis: what can be learned from available microbial genomic resources?
Source: BMC Genomics. 2009 Mar 12;10:104. doi: 10.1186/1471-2164-10-104 (PMC2660368; doi:10.1186/1471-2164-10-104)
Supplement: Additional file 7 — Detailed description of all identified T6SS gene clusters. Archive containing the detailed description of each identified T6SS locus as an HTML file. [file 1471-2164-10-104-S7.tgz › LociHTML/HTML/CP000090B.html]

Locus CP000090B on Ralstonia eutropha (strain JMP134) chromosome 1, complete sequence.

import namespace="svg" implementation="#AdobeSVG"?


# Locus CP000090B

# List of CDS in T6SS locus CP000090B

|  |  |  |  |  |  |  |  |  |
| --- | --- | --- | --- | --- | --- | --- | --- | --- |
| Name | from | to | direct | COG | e-value | COG cover | COG hit start | COG hit end |
| CP000090\_Reut\_A1708 | 1843323 | 1844483 | True | COG0475 | 2e-46 | 96.0 | 4 | 385 |
| CP000090\_Reut\_A1709 | 1844616 | 1845830 | False | COG2814 | 1e-18 | 97.0 | 1 | 385 |
| CP000090\_Reut\_A1710 | 1845827 | 1846192 | False | COG0251 | 2e-12 | 96.0 | 3 | 128 |
| CP000090\_Reut\_A1711 | 1846305 | 1847138 | True | COG1378 | 3e-21 | 82.0 | 4 | 207 |
| CP000090\_Reut\_A1712 | 1847369 | 1847650 | False | - | - | - | - | - |
| CP000090\_Reut\_A1713 | 1847968 | 1848882 | False | - | - | - | - | - |
| CP000090\_Reut\_A1714 | 1848935 | 1850086 | False | COG3515 | 1e-19 | 99.0 | 1 | 343 |
| CP000090\_Reut\_A1715 | 1850094 | 1854242 | False | COG3523 | 2e-31 | 38.0 | 49 | 503 |
| CP000090\_Reut\_A1716 | 1854280 | 1855032 | False | COG3455 | 1e-15 | 80.0 | 34 | 245 |
| CP000090\_Reut\_A1717 | 1855058 | 1856386 | False | COG3522 | 1e-35 | 71.0 | 1 | 317 |
| CP000090\_Reut\_A1718 | 1856453 | 1856965 | False | - | - | - | - | - |
| CP000090\_Reut\_A1719 | 1856997 | 1858430 | False | COG1301 | 6e-29 | 92.0 | 12 | 395 |
| CP000090\_Reut\_A1720 | 1858427 | 1859128 | False | COG1794 | 3e-44 | 99.0 | 1 | 229 |
| CP000090\_Reut\_A1721 | 1859151 | 1860425 | False | COG1301 | 2e-38 | 93.0 | 10 | 395 |
| CP000090\_Reut\_A1722 | 1860446 | 1861489 | False | COG0834 | 2e-17 | 81.0 | 24 | 248 |
| CP000090\_Reut\_A1723 | 1861486 | 1861746 | False | - | - | - | - | - |
| CP000090\_Reut\_A1724 | 1861754 | 1862140 | False | - | - | - | - | - |
| CP000090\_Reut\_A1725 | 1862145 | 1862861 | False | COG2849 | 2e-16 | 68.0 | 72 | 229 |
| CP000090\_Reut\_A1726 | 1862870 | 1864798 | False | COG3501 | 2e-129 | 98.0 | 1 | 542 |
| CP000090\_Reut\_A1727 | 1864803 | 1867499 | False | COG0542 | 0.0 | 98.0 | 1 | 775 |
| CP000090\_Reut\_A1728 | 1867512 | 1868576 | False | COG3520 | 2e-39 | 97.0 | 1 | 325 |
| CP000090\_Reut\_A1729 | 1868540 | 1870300 | False | COG3519 | 9e-123 | 99.0 | 4 | 621 |
| CP000090\_Reut\_A1730 | 1870293 | 1870757 | False | COG3518 | 2e-13 | 94.0 | 4 | 152 |
| CP000090\_Reut\_A1731 | 1870799 | 1871281 | False | COG3157 | 1e-20 | 99.0 | 2 | 162 |
| CP000090\_Reut\_A1732 | 1871383 | 1872885 | False | COG3517 | 0.0 | 99.0 | 5 | 495 |
| CP000090\_Reut\_A1733 | 1872929 | 1873477 | False | COG3516 | 4e-45 | 98.0 | 2 | 167 |
| CP000090\_Reut\_A1734 | 1873980 | 1874753 | True | COG2197 | 2e-37 | 99.0 | 2 | 210 |
| CP000090\_Reut\_A1735 | 1874921 | 1876354 | False | COG1639 | 6e-13 | 75.0 | 38 | 256 |
| CP000090\_Reut\_A1736 | 1876363 | 1878354 | False | COG5001 | 7e-85 | 64.0 | 231 | 657 |
| CP000090\_Reut\_A1737 | 1878453 | 1878926 | False | - | - | - | - | - |
