# Supplementary material for: Dissecting the bacterial type VI secretion system by a genome wide in silico analysis: what can be learned from available microbial genomic resources?
Source: BMC Genomics. 2009 Mar 12;10:104. doi: 10.1186/1471-2164-10-104 (PMC2660368; doi:10.1186/1471-2164-10-104)
Supplement: Additional file 7 — Detailed description of all identified T6SS gene clusters. Archive containing the detailed description of each identified T6SS locus as an HTML file. [file 1471-2164-10-104-S7.tgz › LociHTML/HTML/CP000091E.html]

Locus CP000091E on Ralstonia eutropha (strain JMP134) chromosome 2, complete sequence.

import namespace="svg" implementation="#AdobeSVG"?


# Locus CP000091E

# List of CDS in T6SS locus CP000091E

|  |  |  |  |  |  |  |  |  |
| --- | --- | --- | --- | --- | --- | --- | --- | --- |
| Name | from | to | direct | COG | e-value | COG cover | COG hit start | COG hit end |
| CP000091\_Reut\_B5253 | 2003017 | 2003916 | False | - | - | - | - | - |
| CP000091\_Reut\_B5254 | 2004256 | 2005080 | False | COG0846 | 3e-49 | 96.0 | 2 | 241 |
| CP000091\_Reut\_B5255 | 2005077 | 2005436 | False | - | - | - | - | - |
| CP000091\_Reut\_B5256 | 2005379 | 2005708 | False | - | - | - | - | - |
| CP000091\_Reut\_B5257 | 2005753 | 2005989 | False | - | - | - | - | - |
| CP000091\_Reut\_B5258 | 2006216 | 2008549 | True | COG1752 | 4e-47 | 92.0 | 7 | 290 |
| CP000091\_Reut\_B5258 | 2006216 | 2008549 | True | COG4775 | 1e-18 | 51.0 | 366 | 763 |
| CP000091\_Reut\_B5259 | 2008750 | 2009184 | False | COG3518 | 2e-12 | 82.0 | 8 | 137 |
| CP000091\_Reut\_B5260 | 2009181 | 2011922 | False | COG0542 | 0.0 | 97.0 | 1 | 763 |
| CP000091\_Reut\_B5261 | 2011957 | 2012442 | False | COG3157 | 1e-32 | 96.0 | 1 | 157 |
| CP000091\_Reut\_B5262 | 2012449 | 2014176 | False | COG2885 | 4e-26 | 99.0 | 2 | 190 |
| CP000091\_Reut\_B5263 | 2014181 | 2014828 | False | COG3455 | 6e-18 | 64.0 | 65 | 234 |
| CP000091\_Reut\_B5264 | 2014825 | 2016171 | False | COG3522 | 5e-94 | 99.0 | 2 | 444 |
| CP000091\_Reut\_B5265 | 2016287 | 2017834 | False | COG3517 | 0.0 | 100.0 | 1 | 495 |
| CP000091\_Reut\_B5266 | 2017859 | 2018365 | False | COG3516 | 3e-43 | 94.0 | 4 | 162 |
| CP000091\_Reut\_B5267 | 2018664 | 2021132 | True | COG4253 | 1e-51 | 99.0 | 1 | 276 |
| CP000091\_Reut\_B5267 | 2018664 | 2021132 | True | COG3501 | 2e-56 | 98.0 | 10 | 550 |
| CP000091\_Reut\_B5268 | 2021156 | 2022934 | True | - | - | - | - | - |
| CP000091\_Reut\_B5269 | 2022931 | 2023839 | True | - | - | - | - | - |
| CP000091\_Reut\_B5270 | 2024056 | 2025384 | True | - | - | - | - | - |
| CP000091\_Reut\_B5271 | 2025768 | 2029238 | True | COG3523 | 4e-77 | 95.0 | 15 | 1153 |
| CP000091\_Reut\_B5272 | 2029244 | 2030818 | True | COG3515 | 5e-11 | 57.0 | 15 | 213 |
| CP000091\_Reut\_B5273 | 2030836 | 2032662 | True | COG3519 | 4e-134 | 99.0 | 2 | 617 |
| CP000091\_Reut\_B5274 | 2032659 | 2033708 | True | COG3520 | 2e-53 | 94.0 | 14 | 331 |
| CP000091\_Reut\_B5275 | 2033721 | 2034359 | True | COG3521 | 8e-18 | 83.0 | 1 | 132 |
| CP000091\_Reut\_B5276 | 2034417 | 2035232 | False | COG1414 | 4e-34 | 100.0 | 1 | 246 |
| CP000091\_Reut\_B5277 | 2035363 | 2036346 | True | COG3181 | 9e-59 | 98.0 | 5 | 317 |
| CP000091\_Reut\_B5278 | 2036393 | 2038744 | True | COG1018 | 4e-43 | 98.0 | 6 | 266 |
| CP000091\_Reut\_B5278 | 2036393 | 2038744 | True | COG2124 | 2e-61 | 94.0 | 24 | 411 |
| CP000091\_Reut\_B5279 | 2039056 | 2040567 | True | COG1012 | 3e-127 | 99.0 | 1 | 471 |
