# Supplementary material for: Dissecting the bacterial type VI secretion system by a genome wide in silico analysis: what can be learned from available microbial genomic resources?
Source: BMC Genomics. 2009 Mar 12;10:104. doi: 10.1186/1471-2164-10-104 (PMC2660368; doi:10.1186/1471-2164-10-104)
Supplement: Additional file 7 — Detailed description of all identified T6SS gene clusters. Archive containing the detailed description of each identified T6SS locus as an HTML file. [file 1471-2164-10-104-S7.tgz › LociHTML/HTML/CP000094C.html]

Locus CP000094C on Pseudomonas fluorescens (strain PfO-1) chromosome, complete sequence.

import namespace="svg" implementation="#AdobeSVG"?


# Locus CP000094C

# List of CDS in T6SS locus CP000094C

|  |  |  |  |  |  |  |  |  |
| --- | --- | --- | --- | --- | --- | --- | --- | --- |
| Name | from | to | direct | COG | e-value | COG cover | COG hit start | COG hit end |
| CP000094\_PflO1\_3395 | 3871434 | 3874058 | True | COG1049 | 0.0 | 99.0 | 2 | 852 |
| CP000094\_PflO1\_3396 | 3874222 | 3875502 | False | COG0814 | 7e-71 | 98.0 | 1 | 410 |
| CP000094\_PflO1\_3397 | 3875758 | 3875949 | False | - | - | - | - | - |
| CP000094\_PflO1\_3398 | 3876003 | 3876272 | True | - | - | - | - | - |
| CP000094\_PflO1\_3399 | 3876432 | 3876815 | True | - | - | - | - | - |
| CP000094\_PflO1\_3400 | 3876988 | 3877956 | True | COG1052 | 3e-66 | 98.0 | 4 | 322 |
| CP000094\_PflO1\_3401 | 3877953 | 3879041 | False | COG3515 | 9e-31 | 98.0 | 5 | 345 |
| CP000094\_PflO1\_3402 | 3879038 | 3882859 | False | COG3523 | 4e-63 | 98.0 | 3 | 1175 |
| CP000094\_PflO1\_3403 | 3882856 | 3883620 | False | COG3455 | 9e-33 | 90.0 | 25 | 260 |
| CP000094\_PflO1\_3404 | 3883639 | 3884970 | False | COG3522 | 7e-75 | 100.0 | 1 | 446 |
| CP000094\_PflO1\_3405 | 3885017 | 3885463 | False | - | - | - | - | - |
| CP000094\_PflO1\_3406 | 3885692 | 3886234 | True | COG3516 | 5e-51 | 97.0 | 1 | 165 |
| CP000094\_PflO1\_3407 | 3886264 | 3887748 | True | COG3517 | 0.0 | 99.0 | 5 | 495 |
| CP000094\_PflO1\_3408 | 3887825 | 3888322 | True | COG3157 | 6e-40 | 99.0 | 2 | 162 |
| CP000094\_PflO1\_3409 | 3888338 | 3888781 | True | COG3518 | 8e-20 | 93.0 | 9 | 155 |
| CP000094\_PflO1\_3410 | 3888789 | 3890558 | True | COG3519 | 2e-161 | 99.0 | 6 | 621 |
| CP000094\_PflO1\_3411 | 3890522 | 3891547 | True | COG3520 | 2e-67 | 92.0 | 9 | 319 |
| CP000094\_PflO1\_3412 | 3891549 | 3894089 | True | COG0542 | 0.0 | 96.0 | 1 | 761 |
| CP000094\_PflO1\_3413 | 3894106 | 3894720 | True | - | - | - | - | - |
| CP000094\_PflO1\_3414 | 3894859 | 3895743 | True | - | - | - | - | - |
| CP000094\_PflO1\_3415 | 3895804 | 3897813 | True | COG3501 | 1e-151 | 98.0 | 6 | 544 |
| CP000094\_PflO1\_3416 | 3897826 | 3898368 | True | COG2849 | 3e-19 | 63.0 | 61 | 205 |
| CP000094\_PflO1\_3417 | 3898370 | 3898792 | False | - | - | - | - | - |
| CP000094\_PflO1\_3418 | 3898956 | 3900023 | True | COG3173 | 2e-69 | 99.0 | 1 | 318 |
| CP000094\_PflO1\_3419 | 3900062 | 3900829 | True | COG1028 | 1e-47 | 100.0 | 1 | 251 |
| CP000094\_PflO1\_3420 | 3900875 | 3901477 | False | COG2860 | 4e-36 | 94.0 | 6 | 202 |
| CP000094\_PflO1\_3421 | 3902068 | 3902430 | False | - | - | - | - | - |
| CP000094\_PflO1\_3422 | 3902427 | 3902669 | False | - | - | - | - | - |
