# Supplementary material for: Dissecting the bacterial type VI secretion system by a genome wide in silico analysis: what can be learned from available microbial genomic resources?
Source: BMC Genomics. 2009 Mar 12;10:104. doi: 10.1186/1471-2164-10-104 (PMC2660368; doi:10.1186/1471-2164-10-104)
Supplement: Additional file 7 — Detailed description of all identified T6SS gene clusters. Archive containing the detailed description of each identified T6SS locus as an HTML file. [file 1471-2164-10-104-S7.tgz › LociHTML/HTML/CP000094E.html]

Locus CP000094E on Pseudomonas fluorescens (strain PfO-1) chromosome, complete sequence.

import namespace="svg" implementation="#AdobeSVG"?


# Locus CP000094E

# List of CDS in T6SS locus CP000094E

|  |  |  |  |  |  |  |  |  |
| --- | --- | --- | --- | --- | --- | --- | --- | --- |
| Name | from | to | direct | COG | e-value | COG cover | COG hit start | COG hit end |
| CP000094\_PflO1\_5570 | 6236555 | 6239005 | False | COG0596 | 1e-06 | 91.0 | 21 | 277 |
| CP000094\_PflO1\_5571 | 6239015 | 6240409 | False | COG1090 | 1e-87 | 98.0 | 1 | 294 |
| CP000094\_PflO1\_5572 | 6240486 | 6241364 | False | - | - | - | - | - |
| CP000094\_PflO1\_5573 | 6241361 | 6242368 | False | - | - | - | - | - |
| CP000094\_PflO1\_5574 | 6242365 | 6244590 | False | COG3501 | 5e-169 | 95.0 | 5 | 531 |
| CP000094\_PflO1\_5575 | 6244619 | 6245620 | False | COG0515 | 4e-32 | 73.0 | 2 | 285 |
| CP000094\_PflO1\_5576 | 6245617 | 6246345 | False | COG0631 | 7e-54 | 93.0 | 6 | 250 |
| CP000094\_PflO1\_5577 | 6246345 | 6249884 | False | COG3523 | 0.0 | 99.0 | 7 | 1187 |
| CP000094\_PflO1\_5578 | 6249898 | 6250773 | False | COG3455 | 5e-67 | 97.0 | 7 | 262 |
| CP000094\_PflO1\_5579 | 6250779 | 6252110 | False | COG3522 | 1e-148 | 99.0 | 2 | 446 |
| CP000094\_PflO1\_5580 | 6252113 | 6252613 | False | COG3521 | 3e-37 | 96.0 | 6 | 159 |
| CP000094\_PflO1\_5581 | 6252619 | 6253812 | False | COG3456 | 5e-67 | 100.0 | 1 | 430 |
| CP000094\_PflO1\_5582 | 6253831 | 6253995 | False | - | - | - | - | - |
| CP000094\_PflO1\_5583 | 6254065 | 6255591 | False | COG3604 | 8e-135 | 96.0 | 21 | 550 |
| CP000094\_PflO1\_5584 | 6255602 | 6258259 | False | COG0542 | 2e-123 | 57.0 | 2 | 453 |
| CP000094\_PflO1\_5584 | 6255602 | 6258259 | False | COG0542 | 1e-100 | 48.0 | 396 | 778 |
| CP000094\_PflO1\_5585 | 6258271 | 6259197 | False | COG3520 | 5e-78 | 88.0 | 37 | 333 |
| CP000094\_PflO1\_5586 | 6259242 | 6261029 | False | COG3519 | 5e-164 | 99.0 | 3 | 621 |
| CP000094\_PflO1\_5587 | 6261057 | 6261488 | False | - | - | - | - | - |
| CP000094\_PflO1\_5588 | 6261506 | 6261949 | False | - | - | - | - | - |
| CP000094\_PflO1\_5589 | 6261946 | 6262350 | False | - | - | - | - | - |
| CP000094\_PflO1\_5590 | 6262347 | 6262730 | False | - | - | - | - | - |
| CP000094\_PflO1\_5591 | 6262751 | 6263134 | False | - | - | - | - | - |
| CP000094\_PflO1\_5592 | 6263127 | 6264536 | False | COG4104 | 1e-15 | 96.0 | 4 | 98 |
| CP000094\_PflO1\_5593 | 6264548 | 6264955 | False | COG3518 | 8e-23 | 89.0 | 14 | 154 |
| CP000094\_PflO1\_5594 | 6264965 | 6266440 | False | COG3517 | 0.0 | 98.0 | 4 | 493 |
| CP000094\_PflO1\_5595 | 6266469 | 6266972 | False | COG3516 | 9e-44 | 97.0 | 5 | 169 |
| CP000094\_PflO1\_5596 | 6267005 | 6268567 | False | COG3515 | 2e-22 | 100.0 | 1 | 346 |
| CP000094\_PflO1\_5597 | 6269283 | 6272639 | True | COG3501 | 1e-134 | 97.0 | 13 | 549 |
| CP000094\_PflO1\_5598 | 6272632 | 6273303 | True | - | - | - | - | - |
| CP000094\_PflO1\_5599 | 6273334 | 6273786 | True | - | - | - | - | - |
| CP000094\_PflO1\_5600 | 6273921 | 6274196 | False | COG0776 | 1e-25 | 96.0 | 2 | 92 |
| CP000094\_PflO1\_5601 | 6274387 | 6275535 | False | COG1251 | 6e-46 | 38.0 | 6 | 308 |
| CP000094\_PflO1\_5602 | 6275557 | 6275724 | False | COG1773 | 2e-14 | 98.0 | 1 | 54 |
| CP000094\_PflO1\_5603 | 6275924 | 6276475 | True | COG3161 | 3e-26 | 93.0 | 8 | 169 |
| CP000094\_PflO1\_5604 | 6276475 | 6277365 | True | COG0382 | 4e-52 | 99.0 | 1 | 288 |
| CP000094\_PflO1\_5605 | 6277417 | 6277800 | False | COG4315 | 7e-32 | 88.0 | 17 | 138 |
