# Supplementary material for: Dissecting the bacterial type VI secretion system by a genome wide in silico analysis: what can be learned from available microbial genomic resources?
Source: BMC Genomics. 2009 Mar 12;10:104. doi: 10.1186/1471-2164-10-104 (PMC2660368; doi:10.1186/1471-2164-10-104)
Supplement: Additional file 7 — Detailed description of all identified T6SS gene clusters. Archive containing the detailed description of each identified T6SS locus as an HTML file. [file 1471-2164-10-104-S7.tgz › LociHTML/HTML/CP000113D.html]

Locus CP000113D on Myxococcus xanthus (strain DK 1622) chromosome, complete sequence.

import namespace="svg" implementation="#AdobeSVG"?


# Locus CP000113D

# List of CDS in T6SS locus CP000113D

|  |  |  |  |  |  |  |  |  |
| --- | --- | --- | --- | --- | --- | --- | --- | --- |
| Name | from | to | direct | COG | e-value | COG cover | COG hit start | COG hit end |
| CP000113\_MXAN\_4798 | 5996770 | 6001965 | False | - | - | - | - | - |
| CP000113\_MXAN\_4799 | 6001993 | 6003840 | False | COG2885 | 4e-22 | 54.0 | 85 | 187 |
| CP000113\_MXAN\_4800 | 6004071 | 6006335 | True | COG3501 | 1e-153 | 99.0 | 1 | 545 |
| CP000113\_MXAN\_4801 | 6006481 | 6007098 | True | COG3521 | 1e-14 | 80.0 | 3 | 130 |
| CP000113\_MXAN\_4802 | 6007095 | 6008462 | True | COG3522 | 2e-73 | 97.0 | 1 | 435 |
| CP000113\_MXAN\_4803 | 6008478 | 6009194 | True | COG3455 | 3e-24 | 81.0 | 43 | 255 |
| CP000113\_MXAN\_4804 | 6009191 | 6012886 | True | COG3523 | 2e-150 | 97.0 | 23 | 1184 |
| CP000113\_MXAN\_4805 | 6012888 | 6013820 | True | - | - | - | - | - |
| CP000113\_MXAN\_4806 | 6013836 | 6015860 | True | - | - | - | - | - |
| CP000113\_MXAN\_4807 | 6015943 | 6016437 | True | COG3516 | 2e-38 | 95.0 | 5 | 165 |
| CP000113\_MXAN\_4808 | 6016463 | 6017947 | True | COG3517 | 0.0 | 99.0 | 2 | 493 |
| CP000113\_MXAN\_4809 | 6018067 | 6018558 | True | COG3157 | 6e-19 | 95.0 | 1 | 155 |
| CP000113\_MXAN\_4810 | 6018642 | 6019037 | True | COG3518 | 1e-14 | 90.0 | 8 | 149 |
| CP000113\_MXAN\_4811 | 6019075 | 6020826 | True | COG3519 | 6e-111 | 99.0 | 5 | 621 |
| CP000113\_MXAN\_4812 | 6020862 | 6021833 | True | COG3520 | 7e-45 | 90.0 | 23 | 324 |
| CP000113\_MXAN\_4813 | 6021917 | 6024574 | True | COG0542 | 0.0 | 98.0 | 1 | 777 |
| CP000113\_MXAN\_4814 | 6024583 | 6025677 | True | - | - | - | - | - |
| CP000113\_MXAN\_4815 | 6025827 | 6026192 | True | - | - | - | - | - |
| CP000113\_MXAN\_4816 | 6026252 | 6026809 | True | COG1225 | 4e-12 | 85.0 | 2 | 135 |
| CP000113\_MXAN\_4817 | 6026828 | 6028195 | False | COG2273 | 1e-21 | 86.0 | 16 | 321 |
| CP000113\_MXAN\_4818 | 6028288 | 6029355 | False | COG1118 | 2e-94 | 100.0 | 1 | 345 |
| CP000113\_MXAN\_4819 | 6029352 | 6030179 | False | COG4208 | 1e-99 | 92.0 | 12 | 277 |
| CP000113\_MXAN\_4820 | 6030206 | 6031048 | False | COG0555 | 1e-71 | 97.0 | 5 | 272 |
| CP000113\_MXAN\_4821 | 6031061 | 6032071 | False | COG1613 | 3e-144 | 94.0 | 17 | 346 |
| CP000113\_MXAN\_4822 | 6032121 | 6032954 | True | - | - | - | - | - |
| CP000113\_MXAN\_4823 | 6032464 | 6035913 | True | COG0542 | 4e-93 | 83.0 | 133 | 785 |
| CP000113\_MXAN\_4824 | 6035918 | 6039301 | True | COG0542 | 8e-96 | 80.0 | 153 | 785 |
| CP000113\_MXAN\_4825 | 6039087 | 6041597 | True | COG0542 | 4e-90 | 81.0 | 136 | 777 |
| CP000113\_MXAN\_4826 | 6041643 | 6042224 | False | COG0605 | 6e-72 | 97.0 | 2 | 199 |
| CP000113\_MXAN\_4827 | 6042250 | 6042750 | False | COG1853 | 3e-20 | 90.0 | 5 | 164 |
| CP000113\_MXAN\_4828 | 6043015 | 6043107 | True | - | - | - | - | - |
| CP000113\_MXAN\_4829 | 6043111 | 6045372 | False | COG1529 | 3e-70 | 95.0 | 31 | 725 |
| CP000113\_MXAN\_4830 | 6045383 | 6045844 | False | COG2080 | 8e-56 | 99.0 | 2 | 156 |
| CP000113\_MXAN\_4831 | 6045841 | 6046524 | False | - | - | - | - | - |
| CP000113\_MXAN\_4832 | 6046572 | 6049226 | False | COG0542 | 0.0 | 99.0 | 2 | 786 |
| CP000113\_MXAN\_4833 | 6049391 | 6049825 | True | - | - | - | - | - |
| CP000113\_MXAN\_4834 | 6049632 | 6050570 | False | - | - | - | - | - |
| CP000113\_MXAN\_4835 | 6049900 | 6050466 | True | - | - | - | - | - |
| CP000113\_MXAN\_4836 | 6050649 | 6052112 | True | COG2814 | 1e-11 | 89.0 | 6 | 359 |
| CP000113\_MXAN\_4837 | 6052139 | 6053617 | False | COG5297 | 2e-24 | 41.0 | 172 | 399 |
| CP000113\_MXAN\_4838 | 6053772 | 6055445 | False | COG0154 | 4e-96 | 98.0 | 8 | 473 |
