# Supplementary material for: Dissecting the bacterial type VI secretion system by a genome wide in silico analysis: what can be learned from available microbial genomic resources?
Source: BMC Genomics. 2009 Mar 12;10:104. doi: 10.1186/1471-2164-10-104 (PMC2660368; doi:10.1186/1471-2164-10-104)
Supplement: Additional file 7 — Detailed description of all identified T6SS gene clusters. Archive containing the detailed description of each identified T6SS locus as an HTML file. [file 1471-2164-10-104-S7.tgz › LociHTML/HTML/CP000124B.html]

Locus CP000124B on Burkholderia pseudomallei (strain 1710b) chromosome I, complete sequence.

import namespace="svg" implementation="#AdobeSVG"?


# Locus CP000124B

# List of CDS in T6SS locus CP000124B

|  |  |  |  |  |  |  |  |  |
| --- | --- | --- | --- | --- | --- | --- | --- | --- |
| Name | from | to | direct | COG | e-value | COG cover | COG hit start | COG hit end |
| CP000124\_BURPS1710b\_3626 | 3949098 | 3951416 | True | COG2274 | 0.0 | 98.0 | 1 | 696 |
| CP000124\_BURPS1710b\_3627 | 3949158 | 3951434 | False | - | - | - | - | - |
| CP000124\_BURPS1710b\_3628 | 3951422 | 3952861 | True | COG1538 | 3e-41 | 87.0 | 45 | 443 |
| CP000124\_BURPS1710b\_3629 | 3953071 | 3954039 | False | - | - | - | - | - |
| CP000124\_BURPS1710b\_3630 | 3954051 | 3955016 | True | - | - | - | - | - |
| CP000124\_BURPS1710b\_3631 | 3955167 | 3955280 | True | - | - | - | - | - |
| CP000124\_BURPS1710b\_3632 | 3955288 | 3959181 | True | COG3523 | 0.0 | 100.0 | 1 | 1188 |
| CP000124\_BURPS1710b\_3634 | 3959016 | 3960689 | False | - | - | - | - | - |
| CP000124\_BURPS1710b\_3633 | 3959178 | 3960167 | True | COG3913 | 2e-36 | 93.0 | 5 | 216 |
| CP000124\_BURPS1710b\_3635 | 3960172 | 3961104 | True | COG2885 | 1e-25 | 84.0 | 27 | 186 |
| CP000124\_BURPS1710b\_3636 | 3961303 | 3962424 | False | COG3515 | 5e-32 | 98.0 | 7 | 346 |
| CP000124\_BURPS1710b\_3637 | 3962445 | 3965189 | True | - | - | - | - | - |
| CP000124\_BURPS1710b\_3638 | 3962514 | 3965183 | False | COG0542 | 0.0 | 99.0 | 1 | 784 |
| CP000124\_BURPS1710b\_3639 | 3965217 | 3966317 | False | COG3520 | 8e-61 | 99.0 | 1 | 332 |
| CP000124\_BURPS1710b\_3640 | 3966281 | 3968119 | False | COG3519 | 1e-145 | 99.0 | 2 | 620 |
| CP000124\_BURPS1710b\_3641 | 3968199 | 3968681 | False | COG3518 | 1e-33 | 98.0 | 4 | 157 |
| CP000124\_BURPS1710b\_3642 | 3968739 | 3969242 | False | COG3157 | 2e-34 | 97.0 | 5 | 162 |
| CP000124\_BURPS1710b\_3643 | 3969315 | 3970805 | False | COG3517 | 0.0 | 99.0 | 2 | 495 |
| CP000124\_BURPS1710b\_3644 | 3969444 | 3971018 | True | - | - | - | - | - |
| CP000124\_BURPS1710b\_3645 | 3970822 | 3971340 | False | COG3516 | 2e-47 | 99.0 | 2 | 169 |
| CP000124\_BURPS1710b\_3646 | 3971377 | 3972048 | False | - | - | - | - | - |
| CP000124\_BURPS1710b\_3647 | 3972422 | 3973036 | True | COG3521 | 9e-27 | 92.0 | 1 | 147 |
| CP000124\_BURPS1710b\_3648 | 3973145 | 3974491 | True | COG3522 | 3e-112 | 100.0 | 1 | 446 |
| CP000124\_BURPS1710b\_3649 | 3974611 | 3975273 | True | COG3455 | 2e-42 | 80.0 | 51 | 262 |
| CP000124\_BURPS1710b\_3650 | 3975375 | 3975740 | True | - | - | - | - | - |
| CP000124\_BURPS1710b\_3651 | 3976762 | 3977364 | False | COG0863 | 2e-15 | 62.0 | 81 | 268 |
| CP000124\_BURPS1710b\_3652 | 3977262 | 3977675 | False | COG4385 | 2e-32 | 56.0 | 5 | 121 |
| CP000124\_BURPS1710b\_3653 | 3977668 | 3978012 | False | COG3948 | 4e-21 | 34.0 | 202 | 306 |
