# Supplementary material for: Dissecting the bacterial type VI secretion system by a genome wide in silico analysis: what can be learned from available microbial genomic resources?
Source: BMC Genomics. 2009 Mar 12;10:104. doi: 10.1186/1471-2164-10-104 (PMC2660368; doi:10.1186/1471-2164-10-104)
Supplement: Additional file 7 — Detailed description of all identified T6SS gene clusters. Archive containing the detailed description of each identified T6SS locus as an HTML file. [file 1471-2164-10-104-S7.tgz › LociHTML/HTML/CP000125C.html]

Locus CP000125C on Burkholderia pseudomallei (strain 1710b) chromosome II, complete sequence.

import namespace="svg" implementation="#AdobeSVG"?


# Locus CP000125C

# List of CDS in T6SS locus CP000125C

|  |  |  |  |  |  |  |  |  |
| --- | --- | --- | --- | --- | --- | --- | --- | --- |
| Name | from | to | direct | COG | e-value | COG cover | COG hit start | COG hit end |
| CP000125\_BURPS1710b\_A0529 | 715106 | 715483 | True | - | - | - | - | - |
| CP000125\_BURPS1710b\_A0530 | 715726 | 717609 | False | COG3515 | 3e-08 | 29.0 | 22 | 124 |
| CP000125\_BURPS1710b\_A0531 | 717606 | 718343 | False | COG0745 | 7e-52 | 99.0 | 3 | 229 |
| CP000125\_BURPS1710b\_A0532 | 718340 | 720169 | False | COG0642 | 6e-26 | 98.0 | 1 | 330 |
| CP000125\_BURPS1710b\_A0532 | 718340 | 720169 | False | COG3447 | 2e-07 | 86.0 | 41 | 306 |
| CP000125\_BURPS1710b\_A0534 | 720164 | 722446 | True | COG3517 | 0.0 | 97.0 | 10 | 492 |
| CP000125\_BURPS1710b\_A0533 | 720429 | 720923 | True | COG3516 | 3e-38 | 94.0 | 8 | 166 |
| CP000125\_BURPS1710b\_A0535 | 722666 | 723175 | True | COG3157 | 2e-12 | 95.0 | 1 | 154 |
| CP000125\_BURPS1710b\_A0536 | 723102 | 723653 | False | - | - | - | - | - |
| CP000125\_BURPS1710b\_A0537 | 723168 | 723629 | True | COG3518 | 1e-09 | 87.0 | 4 | 140 |
| CP000125\_BURPS1710b\_A0538 | 723666 | 725408 | True | COG3519 | 6e-102 | 99.0 | 7 | 621 |
| CP000125\_BURPS1710b\_A0539 | 725396 | 726418 | True | COG3520 | 4e-44 | 95.0 | 15 | 335 |
| CP000125\_BURPS1710b\_A0540 | 726405 | 729533 | True | COG0542 | 2e-124 | 54.0 | 1 | 427 |
| CP000125\_BURPS1710b\_A0540 | 726405 | 729533 | True | COG0542 | 3e-101 | 42.0 | 425 | 760 |
| CP000125\_BURPS1710b\_A0541 | 729560 | 732583 | True | COG3501 | 5e-83 | 83.0 | 4 | 463 |
| CP000125\_BURPS1710b\_A0542 | 732609 | 735251 | True | COG1357 | 5e-21 | 93.0 | 17 | 238 |
| CP000125\_BURPS1710b\_A0542 | 732609 | 735251 | True | COG1357 | 3e-09 | 47.0 | 125 | 237 |
| CP000125\_BURPS1710b\_A0542 | 732609 | 735251 | True | COG5351 | 7e-07 | 23.0 | 207 | 291 |
| CP000125\_BURPS1710b\_A0543 | 735269 | 736333 | True | COG1357 | 5e-16 | 95.0 | 1 | 227 |
| CP000125\_BURPS1710b\_A0543 | 735269 | 736333 | True | COG1357 | 8e-09 | 54.0 | 93 | 221 |
| CP000125\_BURPS1710b\_A0544 | 736312 | 737082 | True | - | - | - | - | - |
| CP000125\_BURPS1710b\_A0545 | 737140 | 739740 | True | COG3522 | 1e-40 | 98.0 | 5 | 445 |
| CP000125\_BURPS1710b\_A0546 | 739737 | 740402 | True | COG3455 | 3e-20 | 82.0 | 41 | 256 |
| CP000125\_BURPS1710b\_A0549 | 740509 | 744408 | True | COG3523 | 2e-43 | 34.0 | 59 | 467 |
| CP000125\_BURPS1710b\_A0547 | 742130 | 742243 | True | - | - | - | - | - |
| CP000125\_BURPS1710b\_A0548 | 742256 | 742453 | True | - | - | - | - | - |
| CP000125\_BURPS1710b\_A0550 | 744424 | 746364 | True | - | - | - | - | - |
| CP000125\_BURPS1710b\_A0551 | 745242 | 745670 | False | - | - | - | - | - |
