# Supplementary material for: Dissecting the bacterial type VI secretion system by a genome wide in silico analysis: what can be learned from available microbial genomic resources?
Source: BMC Genomics. 2009 Mar 12;10:104. doi: 10.1186/1471-2164-10-104 (PMC2660368; doi:10.1186/1471-2164-10-104)
Supplement: Additional file 7 — Detailed description of all identified T6SS gene clusters. Archive containing the detailed description of each identified T6SS locus as an HTML file. [file 1471-2164-10-104-S7.tgz › LociHTML/HTML/CP000125D.html]

Locus CP000125D on Burkholderia pseudomallei (strain 1710b) chromosome II, complete sequence.

import namespace="svg" implementation="#AdobeSVG"?


# Locus CP000125D

# List of CDS in T6SS locus CP000125D

|  |  |  |  |  |  |  |  |  |
| --- | --- | --- | --- | --- | --- | --- | --- | --- |
| Name | from | to | direct | COG | e-value | COG cover | COG hit start | COG hit end |
| CP000125\_BURPS1710b\_A1187 | 1490546 | 1491700 | False | COG1609 | 6e-78 | 100.0 | 1 | 333 |
| CP000125\_BURPS1710b\_A1188 | 1492009 | 1492266 | True | - | - | - | - | - |
| CP000125\_BURPS1710b\_A1189 | 1493037 | 1493783 | True | - | - | - | - | - |
| CP000125\_BURPS1710b\_A1190 | 1494243 | 1494839 | True | COG1961 | 1e-15 | 46.0 | 40 | 142 |
| CP000125\_BURPS1710b\_A1191 | 1494515 | 1495792 | False | COG3209 | 3e-12 | 18.0 | 2 | 145 |
| CP000125\_BURPS1710b\_A1191 | 1494515 | 1495792 | False | COG3677 | 4e-08 | 83.0 | 22 | 129 |
| CP000125\_BURPS1710b\_A1192 | 1495734 | 1496444 | False | - | - | - | - | - |
| CP000125\_BURPS1710b\_A1193 | 1496460 | 1498655 | False | COG3501 | 3e-143 | 96.0 | 23 | 550 |
| CP000125\_BURPS1710b\_A1194 | 1498661 | 1501327 | False | COG0542 | 0.0 | 100.0 | 1 | 786 |
| CP000125\_BURPS1710b\_A1195 | 1501306 | 1502784 | False | COG3520 | 7e-57 | 94.0 | 3 | 318 |
| CP000125\_BURPS1710b\_A1196 | 1502781 | 1504652 | False | COG3519 | 4e-168 | 100.0 | 1 | 621 |
| CP000125\_BURPS1710b\_A1197 | 1504657 | 1505217 | False | COG3518 | 5e-18 | 94.0 | 1 | 149 |
| CP000125\_BURPS1710b\_A1198 | 1505236 | 1505727 | False | COG3157 | 3e-19 | 98.0 | 1 | 159 |
| CP000125\_BURPS1710b\_A1199 | 1505787 | 1507295 | False | COG3517 | 0.0 | 99.0 | 2 | 495 |
| CP000125\_BURPS1710b\_A1200 | 1507288 | 1507866 | False | COG3516 | 2e-53 | 98.0 | 2 | 167 |
| CP000125\_BURPS1710b\_A1201 | 1507929 | 1509008 | False | COG3515 | 5e-20 | 97.0 | 6 | 341 |
| CP000125\_BURPS1710b\_A1202 | 1509061 | 1511712 | False | COG0515 | 1e-30 | 72.0 | 1 | 277 |
| CP000125\_BURPS1710b\_A1203 | 1511923 | 1512828 | False | COG3913 | 3e-12 | 83.0 | 2 | 190 |
| CP000125\_BURPS1710b\_A1204 | 1512834 | 1516463 | False | COG3523 | 0.0 | 99.0 | 7 | 1185 |
| CP000125\_BURPS1710b\_A1206 | 1516445 | 1518211 | True | - | - | - | - | - |
| CP000125\_BURPS1710b\_A1205 | 1516466 | 1517782 | False | COG3455 | 2e-59 | 93.0 | 13 | 258 |
| CP000125\_BURPS1710b\_A1205 | 1516466 | 1517782 | False | COG1360 | 2e-27 | 56.0 | 103 | 240 |
| CP000125\_BURPS1710b\_A1207 | 1517798 | 1519162 | False | COG3522 | 6e-128 | 99.0 | 4 | 446 |
| CP000125\_BURPS1710b\_A1208 | 1519198 | 1519773 | False | COG3521 | 6e-26 | 99.0 | 1 | 158 |
| CP000125\_BURPS1710b\_A1209 | 1520224 | 1520757 | True | - | - | - | - | - |
| CP000125\_BURPS1710b\_A1210 | 1520812 | 1522248 | True | COG3456 | 4e-32 | 93.0 | 22 | 425 |
| CP000125\_BURPS1710b\_A1211 | 1522291 | 1524057 | False | - | - | - | - | - |
| CP000125\_BURPS1710b\_A1212 | 1522345 | 1524399 | True | - | - | - | - | - |
| CP000125\_BURPS1710b\_A1213 | 1523993 | 1524484 | True | - | - | - | - | - |
| CP000125\_BURPS1710b\_A1214 | 1524664 | 1525908 | True | COG0654 | 7e-36 | 87.0 | 2 | 340 |
| CP000125\_BURPS1710b\_A1215 | 1525981 | 1526535 | True | COG3161 | 9e-17 | 93.0 | 7 | 169 |
| CP000125\_BURPS1710b\_A1217 | 1526471 | 1527247 | False | - | - | - | - | - |
| CP000125\_BURPS1710b\_A1216 | 1526584 | 1526760 | True | - | - | - | - | - |
