# Supplementary material for: Dissecting the bacterial type VI secretion system by a genome wide in silico analysis: what can be learned from available microbial genomic resources?
Source: BMC Genomics. 2009 Mar 12;10:104. doi: 10.1186/1471-2164-10-104 (PMC2660368; doi:10.1186/1471-2164-10-104)
Supplement: Additional file 7 — Detailed description of all identified T6SS gene clusters. Archive containing the detailed description of each identified T6SS locus as an HTML file. [file 1471-2164-10-104-S7.tgz › LociHTML/HTML/CP000125F.html]

Locus CP000125F on Burkholderia pseudomallei (strain 1710b) chromosome II, complete sequence.

import namespace="svg" implementation="#AdobeSVG"?


# Locus CP000125F

# List of CDS in T6SS locus CP000125F

|  |  |  |  |  |  |  |  |  |
| --- | --- | --- | --- | --- | --- | --- | --- | --- |
| Name | from | to | direct | COG | e-value | COG cover | COG hit start | COG hit end |
| CP000125\_BURPS1710b\_A1598 | 1948636 | 1949181 | True | COG3539 | 3e-15 | 100.0 | 1 | 184 |
| CP000125\_BURPS1710b\_A1599 | 1949261 | 1949983 | True | COG3121 | 5e-59 | 95.0 | 10 | 234 |
| CP000125\_BURPS1710b\_A1600 | 1950099 | 1952840 | True | COG3188 | 0.0 | 96.0 | 12 | 818 |
| CP000125\_BURPS1710b\_A1601 | 1952742 | 1952966 | False | - | - | - | - | - |
| CP000125\_BURPS1710b\_A1602 | 1952842 | 1953405 | True | COG3539 | 1e-15 | 96.0 | 6 | 182 |
| CP000125\_BURPS1710b\_A1603 | 1953449 | 1954126 | True | COG3455 | 4e-09 | 68.0 | 68 | 247 |
| CP000125\_BURPS1710b\_A1604 | 1954129 | 1955805 | True | COG2885 | 1e-26 | 76.0 | 46 | 190 |
| CP000125\_BURPS1710b\_A1605 | 1956195 | 1956734 | True | COG3516 | 4e-59 | 99.0 | 2 | 169 |
| CP000125\_BURPS1710b\_A1606 | 1956768 | 1958267 | True | COG3517 | 0.0 | 100.0 | 1 | 495 |
| CP000125\_BURPS1710b\_A1607 | 1958467 | 1958949 | True | COG3157 | 1e-35 | 99.0 | 1 | 161 |
| CP000125\_BURPS1710b\_A1608 | 1959532 | 1960974 | True | COG3522 | 5e-136 | 99.0 | 1 | 445 |
| CP000125\_BURPS1710b\_A1609 | 1960971 | 1962272 | True | COG3455 | 8e-49 | 93.0 | 15 | 260 |
| CP000125\_BURPS1710b\_A1609 | 1960971 | 1962272 | True | COG1360 | 9e-28 | 56.0 | 103 | 241 |
| CP000125\_BURPS1710b\_A1610 | 1962287 | 1966195 | True | COG3523 | 0.0 | 99.0 | 2 | 1184 |
| CP000125\_BURPS1710b\_A1611 | 1966226 | 1966348 | True | - | - | - | - | - |
| CP000125\_BURPS1710b\_A1612 | 1966385 | 1966954 | True | - | - | - | - | - |
| CP000125\_BURPS1710b\_A1613 | 1967052 | 1969628 | True | COG3501 | 7e-132 | 90.0 | 24 | 523 |
| CP000125\_BURPS1710b\_A1613 | 1967052 | 1969628 | True | COG3889 | 2e-13 | 19.0 | 678 | 850 |
| CP000125\_BURPS1710b\_A1614 | 1969703 | 1969972 | True | COG4104 | 1e-06 | 89.0 | 10 | 97 |
| CP000125\_BURPS1710b\_A1615 | 1969985 | 1973437 | True | COG3209 | 2e-21 | 84.0 | 20 | 693 |
| CP000125\_BURPS1710b\_A1616 | 1973330 | 1974724 | False | - | - | - | - | - |
| CP000125\_BURPS1710b\_A1617 | 1974527 | 1974619 | True | - | - | - | - | - |
| CP000125\_BURPS1710b\_A1618 | 1974721 | 1975749 | False | - | - | - | - | - |
| CP000125\_BURPS1710b\_A1619 | 1975804 | 1976874 | False | COG3515 | 9e-21 | 97.0 | 1 | 338 |
| CP000125\_BURPS1710b\_A1620 | 1976893 | 1977942 | False | COG3520 | 9e-82 | 97.0 | 9 | 334 |
| CP000125\_BURPS1710b\_A1621 | 1977939 | 1979819 | False | COG3519 | 0.0 | 100.0 | 1 | 621 |
| CP000125\_BURPS1710b\_A1622 | 1979821 | 1980339 | False | COG3518 | 8e-21 | 95.0 | 8 | 157 |
| CP000125\_BURPS1710b\_A1623 | 1980326 | 1981180 | False | COG4455 | 9e-58 | 95.0 | 12 | 273 |
| CP000125\_BURPS1710b\_A1624 | 1981164 | 1982219 | False | - | - | - | - | - |
| CP000125\_BURPS1710b\_A1625 | 1982538 | 1985294 | True | COG0542 | 0.0 | 98.0 | 1 | 775 |
| CP000125\_BURPS1710b\_A1626 | 1985557 | 1985676 | True | - | - | - | - | - |
| CP000125\_BURPS1710b\_A1627 | 1985685 | 1988651 | False | COG2204 | 5e-18 | 34.0 | 6 | 165 |
| CP000125\_BURPS1710b\_A1627 | 1985685 | 1988651 | False | COG0642 | 8e-41 | 82.0 | 57 | 333 |
| CP000125\_BURPS1710b\_A1628 | 1989401 | 1989661 | True | - | - | - | - | - |
| CP000125\_BURPS1710b\_A1629 | 1989988 | 1990080 | True | - | - | - | - | - |
