# Supplementary material for: Dissecting the bacterial type VI secretion system by a genome wide in silico analysis: what can be learned from available microbial genomic resources?
Source: BMC Genomics. 2009 Mar 12;10:104. doi: 10.1186/1471-2164-10-104 (PMC2660368; doi:10.1186/1471-2164-10-104)
Supplement: Additional file 7 — Detailed description of all identified T6SS gene clusters. Archive containing the detailed description of each identified T6SS locus as an HTML file. [file 1471-2164-10-104-S7.tgz › LociHTML/HTML/CP000125G.html]

Locus CP000125G on Burkholderia pseudomallei (strain 1710b) chromosome II, complete sequence.

import namespace="svg" implementation="#AdobeSVG"?


# Locus CP000125G

# List of CDS in T6SS locus CP000125G

|  |  |  |  |  |  |  |  |  |
| --- | --- | --- | --- | --- | --- | --- | --- | --- |
| Name | from | to | direct | COG | e-value | COG cover | COG hit start | COG hit end |
| CP000125\_BURPS1710b\_A1682 | 2053579 | 2055447 | True | COG0367 | 4e-105 | 95.0 | 1 | 520 |
| CP000125\_BURPS1710b\_A1684 | 2055392 | 2057467 | False | - | - | - | - | - |
| CP000125\_BURPS1710b\_A1685 | 2055463 | 2057889 | True | - | - | - | - | - |
| CP000125\_BURPS1710b\_A1683 | 2055476 | 2056447 | True | COG0604 | 5e-63 | 100.0 | 1 | 326 |
| CP000125\_BURPS1710b\_A1686 | 2056502 | 2057428 | True | COG3384 | 1e-15 | 78.0 | 31 | 240 |
| CP000125\_BURPS1710b\_A1687 | 2057672 | 2059033 | False | COG0583 | 3e-29 | 97.0 | 2 | 292 |
| CP000125\_BURPS1710b\_A1688 | 2059272 | 2059475 | True | - | - | - | - | - |
| CP000125\_BURPS1710b\_A1689 | 2059856 | 2063932 | False | COG3523 | 0.0 | 99.0 | 2 | 1184 |
| CP000125\_BURPS1710b\_A1690 | 2063961 | 2065226 | False | COG3455 | 1e-47 | 94.0 | 15 | 262 |
| CP000125\_BURPS1710b\_A1690 | 2063961 | 2065226 | False | COG1360 | 1e-26 | 67.0 | 79 | 242 |
| CP000125\_BURPS1710b\_A1691 | 2065315 | 2066661 | False | COG3522 | 1e-127 | 100.0 | 1 | 446 |
| CP000125\_BURPS1710b\_A1692 | 2066683 | 2067186 | False | COG3521 | 4e-29 | 93.0 | 8 | 155 |
| CP000125\_BURPS1710b\_A1693 | 2067293 | 2067778 | False | COG3157 | 5e-37 | 94.0 | 1 | 153 |
| CP000125\_BURPS1710b\_A1694 | 2067895 | 2069487 | False | COG3517 | 0.0 | 99.0 | 3 | 495 |
| CP000125\_BURPS1710b\_A1695 | 2069429 | 2073145 | False | COG3516 | 4e-59 | 95.0 | 2 | 163 |
| CP000125\_BURPS1710b\_A1696 | 2070046 | 2072973 | False | COG0542 | 3e-122 | 57.0 | 1 | 452 |
| CP000125\_BURPS1710b\_A1696 | 2070046 | 2072973 | False | COG0542 | 4e-101 | 39.0 | 453 | 761 |
| CP000125\_BURPS1710b\_A1697 | 2073479 | 2074225 | True | - | - | - | - | - |
| CP000125\_BURPS1710b\_A1698 | 2074222 | 2075187 | True | COG4455 | 1e-52 | 95.0 | 14 | 273 |
| CP000125\_BURPS1710b\_A1699 | 2075174 | 2075755 | True | COG3518 | 6e-19 | 92.0 | 6 | 151 |
| CP000125\_BURPS1710b\_A1700 | 2075786 | 2077675 | True | COG3519 | 0.0 | 99.0 | 1 | 619 |
| CP000125\_BURPS1710b\_A1701 | 2077675 | 2078760 | True | COG3520 | 1e-73 | 98.0 | 7 | 335 |
| CP000125\_BURPS1710b\_A1702 | 2078757 | 2079860 | True | COG3515 | 2e-20 | 96.0 | 2 | 336 |
| CP000125\_BURPS1710b\_A1703 | 2079932 | 2082259 | True | COG3501 | 2e-131 | 94.0 | 10 | 531 |
| CP000125\_BURPS1710b\_A1704 | 2082180 | 2084768 | True | COG1357 | 3e-23 | 93.0 | 1 | 222 |
| CP000125\_BURPS1710b\_A1704 | 2082180 | 2084768 | True | COG5351 | 1e-21 | 49.0 | 100 | 281 |
| CP000125\_BURPS1710b\_A1705 | 2084765 | 2085847 | True | COG1357 | 4e-21 | 83.0 | 37 | 234 |
| CP000125\_BURPS1710b\_A1706 | 2086029 | 2086688 | True | - | - | - | - | - |
| CP000125\_BURPS1710b\_A1707 | 2086729 | 2087109 | True | - | - | - | - | - |
