# Supplementary material for: Dissecting the bacterial type VI secretion system by a genome wide in silico analysis: what can be learned from available microbial genomic resources?
Source: BMC Genomics. 2009 Mar 12;10:104. doi: 10.1186/1471-2164-10-104 (PMC2660368; doi:10.1186/1471-2164-10-104)
Supplement: Additional file 7 — Detailed description of all identified T6SS gene clusters. Archive containing the detailed description of each identified T6SS locus as an HTML file. [file 1471-2164-10-104-S7.tgz › LociHTML/HTML/CP000125H.html]

Locus CP000125H on Burkholderia pseudomallei (strain 1710b) chromosome II, complete sequence.

import namespace="svg" implementation="#AdobeSVG"?


# Locus CP000125H

# List of CDS in T6SS locus CP000125H

|  |  |  |  |  |  |  |  |  |
| --- | --- | --- | --- | --- | --- | --- | --- | --- |
| Name | from | to | direct | COG | e-value | COG cover | COG hit start | COG hit end |
| CP000125\_BURPS1710b\_A2069 | 2517018 | 2517869 | False | - | - | - | - | - |
| CP000125\_BURPS1710b\_A2070 | 2517788 | 2518618 | False | COG0558 | 1e-14 | 96.0 | 2 | 186 |
| CP000125\_BURPS1710b\_A2071 | 2518722 | 2520350 | True | COG0427 | 1e-160 | 98.0 | 8 | 501 |
| CP000125\_BURPS1710b\_A2072 | 2520447 | 2520575 | True | - | - | - | - | - |
| CP000125\_BURPS1710b\_A2073 | 2522107 | 2523009 | True | COG3515 | 2e-10 | 83.0 | 54 | 343 |
| CP000125\_BURPS1710b\_A2074 | 2523043 | 2523591 | True | COG3516 | 6e-47 | 97.0 | 5 | 169 |
| CP000125\_BURPS1710b\_A2075 | 2523594 | 2525099 | True | COG3517 | 0.0 | 99.0 | 1 | 494 |
| CP000125\_BURPS1710b\_A2076 | 2525243 | 2525770 | True | COG3157 | 2e-27 | 98.0 | 1 | 160 |
| CP000125\_BURPS1710b\_A2077 | 2525850 | 2526281 | True | - | - | - | - | - |
| CP000125\_BURPS1710b\_A2078 | 2526295 | 2528157 | True | COG3519 | 2e-87 | 96.0 | 3 | 604 |
| CP000125\_BURPS1710b\_A2079 | 2528154 | 2529143 | True | COG3520 | 1e-33 | 93.0 | 15 | 328 |
| CP000125\_BURPS1710b\_A2080 | 2529146 | 2532016 | True | COG0542 | 0.0 | 97.0 | 2 | 766 |
| CP000125\_BURPS1710b\_A2081 | 2532007 | 2534298 | True | COG3501 | 3e-136 | 97.0 | 10 | 544 |
| CP000125\_BURPS1710b\_A2082 | 2534464 | 2536752 | True | COG3501 | 3e-135 | 96.0 | 10 | 537 |
| CP000125\_BURPS1710b\_A2083 | 2536756 | 2538972 | True | COG1357 | 5e-08 | 61.0 | 58 | 204 |
| CP000125\_BURPS1710b\_A2084 | 2538972 | 2540042 | True | COG1357 | 8e-09 | 66.0 | 32 | 190 |
| CP000125\_BURPS1710b\_A2085 | 2540045 | 2540761 | True | - | - | - | - | - |
| CP000125\_BURPS1710b\_A2086 | 2540804 | 2541193 | True | - | - | - | - | - |
| CP000125\_BURPS1710b\_A2087 | 2541253 | 2541792 | True | COG3521 | 8e-07 | 58.0 | 14 | 106 |
| CP000125\_BURPS1710b\_A2088 | 2541789 | 2543150 | True | COG3522 | 5e-91 | 98.0 | 7 | 446 |
| CP000125\_BURPS1710b\_A2089 | 2543176 | 2544891 | True | COG3455 | 2e-25 | 98.0 | 1 | 257 |
| CP000125\_BURPS1710b\_A2089 | 2543176 | 2544891 | True | COG1360 | 1e-18 | 50.0 | 123 | 244 |
| CP000125\_BURPS1710b\_A2090 | 2545077 | 2548391 | True | COG3523 | 2e-127 | 92.0 | 84 | 1185 |
| CP000125\_BURPS1710b\_A2091 | 2548450 | 2548809 | True | - | - | - | - | - |
| CP000125\_BURPS1710b\_A2092 | 2548964 | 2549257 | True | - | - | - | - | - |
| CP000125\_BURPS1710b\_A2093 | 2549482 | 2549853 | True | - | - | - | - | - |
| CP000125\_BURPS1710b\_A2094 | 2550404 | 2551303 | True | - | - | - | - | - |
| CP000125\_BURPS1710b\_A2095 | 2551537 | 2552856 | True | COG1819 | 1e-39 | 98.0 | 1 | 399 |
| CP000125\_BURPS1710b\_A2096 | 2552853 | 2554439 | True | COG2814 | 3e-15 | 50.0 | 1 | 198 |
