# Supplementary material for: Dissecting the bacterial type VI secretion system by a genome wide in silico analysis: what can be learned from available microbial genomic resources?
Source: BMC Genomics. 2009 Mar 12;10:104. doi: 10.1186/1471-2164-10-104 (PMC2660368; doi:10.1186/1471-2164-10-104)
Supplement: Additional file 7 — Detailed description of all identified T6SS gene clusters. Archive containing the detailed description of each identified T6SS locus as an HTML file. [file 1471-2164-10-104-S7.tgz › LociHTML/HTML/CP000142A.html]

Locus CP000142A on Pelobacter carbinolicus (strain DSM 2380 / Gra Bd 1) chromosome, complete sequence.

import namespace="svg" implementation="#AdobeSVG"?


# Locus CP000142A

# List of CDS in T6SS locus CP000142A

|  |  |  |  |  |  |  |  |  |
| --- | --- | --- | --- | --- | --- | --- | --- | --- |
| Name | from | to | direct | COG | e-value | COG cover | COG hit start | COG hit end |
| CP000142\_Pcar\_2803 | 3269618 | 3271252 | False | - | - | - | - | - |
| CP000142\_Pcar\_2804 | 3271707 | 3272756 | True | COG1830 | 1e-64 | 100.0 | 1 | 265 |
| CP000142\_Pcar\_2805 | 3272805 | 3273509 | False | - | - | - | - | - |
| CP000142\_Pcar\_2806 | 3273717 | 3273974 | False | COG1278 | 4e-23 | 98.0 | 1 | 66 |
| CP000142\_Pcar\_3381 | 3274067 | 3274282 | True | - | - | - | - | - |
| CP000142\_Pcar\_2807 | 3274554 | 3275129 | True | - | - | - | - | - |
| CP000142\_Pcar\_2808 | 3275129 | 3275593 | True | COG3521 | 1e-14 | 81.0 | 7 | 135 |
| CP000142\_Pcar\_2809 | 3275715 | 3277043 | True | COG3522 | 3e-60 | 96.0 | 6 | 434 |
| CP000142\_Pcar\_2810 | 3277049 | 3277774 | True | COG3455 | 8e-32 | 79.0 | 39 | 246 |
| CP000142\_Pcar\_2811 | 3277808 | 3281395 | True | COG3523 | 2e-155 | 100.0 | 1 | 1188 |
| CP000142\_Pcar\_2812 | 3281407 | 3282420 | True | COG3913 | 6e-09 | 56.0 | 6 | 134 |
| CP000142\_Pcar\_2813 | 3282417 | 3283943 | True | COG3515 | 5e-09 | 47.0 | 8 | 170 |
| CP000142\_Pcar\_2814 | 3284003 | 3284530 | True | COG3516 | 6e-38 | 93.0 | 4 | 161 |
| CP000142\_Pcar\_2815 | 3284548 | 3286089 | True | COG3517 | 6e-170 | 94.0 | 23 | 491 |
| CP000142\_Pcar\_2816 | 3286337 | 3286822 | True | COG3157 | 1e-31 | 95.0 | 1 | 154 |
| CP000142\_Pcar\_2817 | 3286941 | 3287369 | True | COG3518 | 3e-11 | 76.0 | 8 | 127 |
| CP000142\_Pcar\_2818 | 3287362 | 3289119 | True | COG3519 | 2e-96 | 99.0 | 7 | 621 |
| CP000142\_Pcar\_2819 | 3289083 | 3290060 | True | COG3520 | 2e-42 | 92.0 | 15 | 324 |
| CP000142\_Pcar\_2820 | 3290118 | 3292817 | True | COG0542 | 0.0 | 96.0 | 1 | 760 |
| CP000142\_Pcar\_2821 | 3292979 | 3295051 | True | COG3501 | 1e-84 | 100.0 | 1 | 550 |
| CP000142\_Pcar\_2822 | 3295170 | 3297539 | True | COG3501 | 2e-40 | 83.0 | 6 | 463 |
| CP000142\_Pcar\_2823 | 3297546 | 3300362 | True | - | - | - | - | - |
| CP000142\_Pcar\_2824 | 3300437 | 3301507 | True | - | - | - | - | - |
| CP000142\_Pcar\_2825 | 3301659 | 3302084 | True | - | - | - | - | - |
| CP000142\_Pcar\_2826 | 3302087 | 3303325 | True | - | - | - | - | - |
