# Supplementary material for: Dissecting the bacterial type VI secretion system by a genome wide in silico analysis: what can be learned from available microbial genomic resources?
Source: BMC Genomics. 2009 Mar 12;10:104. doi: 10.1186/1471-2164-10-104 (PMC2660368; doi:10.1186/1471-2164-10-104)
Supplement: Additional file 7 — Detailed description of all identified T6SS gene clusters. Archive containing the detailed description of each identified T6SS locus as an HTML file. [file 1471-2164-10-104-S7.tgz › LociHTML/HTML/CP000144A.html]

Locus CP000144A on Rhodobacter sphaeroides (strain ATCC 17023 / 2.4.1 / NCIB 8253 / DSM 158) chromosome 2, complete sequence.

import namespace="svg" implementation="#AdobeSVG"?


# Locus CP000144A

# List of CDS in T6SS locus CP000144A

|  |  |  |  |  |  |  |  |  |
| --- | --- | --- | --- | --- | --- | --- | --- | --- |
| Name | from | to | direct | COG | e-value | COG cover | COG hit start | COG hit end |
| CP000144\_RHOS4\_35020 | 535219 | 536508 | True | - | - | - | - | - |
| CP000144\_RHOS4\_35030 | 536510 | 537532 | True | COG5351 | 1e-67 | 91.0 | 1 | 335 |
| CP000144\_RHOS4\_35040 | 537525 | 538616 | True | COG0304 | 1e-08 | 61.0 | 127 | 381 |
| CP000144\_RHOS4\_35050 | 538613 | 539614 | True | - | - | - | - | - |
| CP000144\_RHOS4\_35060 | 539611 | 540390 | True | - | - | - | - | - |
| CP000144\_RHOS4\_35070 | 540413 | 541099 | True | - | - | - | - | - |
| CP000144\_RHOS4\_35080 | 541096 | 541494 | False | - | - | - | - | - |
| CP000144\_RHOS4\_35090 | 541487 | 544156 | False | COG0542 | 0.0 | 100.0 | 1 | 786 |
| CP000144\_RHOS4\_35100 | 544270 | 546276 | True | COG0515 | 2e-31 | 76.0 | 2 | 294 |
| CP000144\_RHOS4\_35110 | 546374 | 547447 | True | COG3515 | 4e-16 | 99.0 | 1 | 345 |
| CP000144\_RHOS4\_35120 | 547466 | 547996 | True | COG3516 | 7e-46 | 99.0 | 2 | 169 |
| CP000144\_RHOS4\_35130 | 547993 | 549510 | True | COG3517 | 0.0 | 99.0 | 2 | 495 |
| CP000144\_RHOS4\_35140 | 549562 | 550053 | True | COG3157 | 2e-22 | 94.0 | 1 | 153 |
| CP000144\_RHOS4\_35150 | 550086 | 550793 | True | COG3518 | 5e-15 | 84.0 | 25 | 157 |
| CP000144\_RHOS4\_35160 | 550793 | 552658 | True | COG3519 | 9e-133 | 100.0 | 1 | 621 |
| CP000144\_RHOS4\_35170 | 552622 | 553620 | True | COG3520 | 2e-43 | 89.0 | 23 | 323 |
| CP000144\_RHOS4\_35180 | 553614 | 554843 | True | COG3456 | 4e-60 | 98.0 | 5 | 426 |
| CP000144\_RHOS4\_35190 | 554836 | 555288 | True | COG3521 | 9e-22 | 98.0 | 1 | 156 |
| CP000144\_RHOS4\_35200 | 555297 | 556625 | True | COG3522 | 5e-101 | 99.0 | 2 | 446 |
| CP000144\_RHOS4\_35210 | 556690 | 558051 | True | COG3455 | 2e-41 | 98.0 | 6 | 262 |
| CP000144\_RHOS4\_35210 | 556690 | 558051 | True | COG1360 | 1e-22 | 52.0 | 117 | 243 |
| CP000144\_RHOS4\_35220 | 558051 | 561572 | True | COG3523 | 0.0 | 99.0 | 1 | 1187 |
| CP000144\_RHOS4\_35230 | 561562 | 562053 | True | COG3913 | 2e-10 | 63.0 | 1 | 145 |
| CP000144\_RHOS4\_35240 | 562063 | 564366 | False | COG3501 | 2e-143 | 96.0 | 15 | 547 |
| CP000144\_RHOS4\_35250 | 564383 | 566791 | False | COG3523 | 1e-43 | 63.0 | 430 | 1187 |
| CP000144\_RHOS4\_35260 | 566791 | 567639 | False | - | - | - | - | - |
| CP000144\_RHOS4\_35270 | 567643 | 568389 | False | COG0631 | 2e-49 | 91.0 | 12 | 251 |
| CP000144\_RHOS4\_35280 | 568532 | 569296 | True | COG2885 | 2e-19 | 92.0 | 12 | 186 |
| CP000144\_RHOS4\_35290 | 569293 | 570219 | False | - | - | - | - | - |
| CP000144\_RHOS4\_35300 | 570216 | 570626 | False | - | - | - | - | - |
| CP000144\_RHOS4\_35310 | 570626 | 572137 | False | COG0438 | 5e-11 | 77.0 | 86 | 380 |
