# Supplementary material for: Dissecting the bacterial type VI secretion system by a genome wide in silico analysis: what can be learned from available microbial genomic resources?
Source: BMC Genomics. 2009 Mar 12;10:104. doi: 10.1186/1471-2164-10-104 (PMC2660368; doi:10.1186/1471-2164-10-104)
Supplement: Additional file 7 — Detailed description of all identified T6SS gene clusters. Archive containing the detailed description of each identified T6SS locus as an HTML file. [file 1471-2164-10-104-S7.tgz › LociHTML/HTML/CP000148A.html]

Locus CP000148A on Geobacter metallireducens (strain GS-15 / ATCC 53774 / DSM 7210) chromosome, complete sequence.

import namespace="svg" implementation="#AdobeSVG"?


# Locus CP000148A

# List of CDS in T6SS locus CP000148A

|  |  |  |  |  |  |  |  |  |
| --- | --- | --- | --- | --- | --- | --- | --- | --- |
| Name | from | to | direct | COG | e-value | COG cover | COG hit start | COG hit end |
| CP000148\_Gmet\_0265 | 296892 | 297494 | True | COG1051 | 6e-07 | 74.0 | 12 | 119 |
| CP000148\_Gmet\_0266 | 297518 | 297910 | False | - | - | - | - | - |
| CP000148\_Gmet\_0267 | 298034 | 298846 | True | COG0564 | 6e-48 | 72.0 | 78 | 286 |
| CP000148\_Gmet\_0268 | 298880 | 299368 | True | COG0225 | 3e-60 | 90.0 | 3 | 159 |
| CP000148\_Gmet\_0269 | 299386 | 299868 | True | - | - | - | - | - |
| CP000148\_Gmet\_0270 | 299885 | 300904 | True | COG2008 | 2e-98 | 100.0 | 1 | 342 |
| CP000148\_Gmet\_0271 | 300992 | 301654 | True | - | - | - | - | - |
| CP000148\_Gmet\_0272 | 301651 | 301962 | True | - | - | - | - | - |
| CP000148\_Gmet\_0273 | 302320 | 303000 | True | COG3455 | 3e-20 | 70.0 | 61 | 245 |
| CP000148\_Gmet\_0274 | 303002 | 306466 | True | COG3523 | 7e-70 | 47.0 | 15 | 581 |
| CP000148\_Gmet\_0275 | 306508 | 308880 | True | COG3515 | 4e-11 | 53.0 | 6 | 189 |
| CP000148\_Gmet\_0276 | 308902 | 309546 | True | - | - | - | - | - |
| CP000148\_Gmet\_0277 | 309543 | 309872 | True | - | - | - | - | - |
| CP000148\_Gmet\_0278 | 309957 | 310445 | True | COG3516 | 3e-41 | 95.0 | 5 | 166 |
| CP000148\_Gmet\_0279 | 310458 | 311936 | True | COG3517 | 0.0 | 98.0 | 4 | 493 |
| CP000148\_Gmet\_0280 | 312033 | 312518 | True | COG3157 | 4e-33 | 97.0 | 1 | 158 |
| CP000148\_Gmet\_0281 | 312658 | 313446 | True | - | - | - | - | - |
| CP000148\_Gmet\_0282 | 313724 | 313975 | False | - | - | - | - | - |
| CP000148\_Gmet\_0283 | 314162 | 315238 | False | COG3666 | 4e-14 | 88.0 | 14 | 156 |
| CP000148\_Gmet\_0284 | 315572 | 316501 | True | - | - | - | - | - |
| CP000148\_Gmet\_0285 | 316524 | 317402 | True | COG3409 | 3e-07 | 35.0 | 39 | 103 |
| CP000148\_Gmet\_0286 | 317492 | 319621 | True | COG3501 | 4e-141 | 99.0 | 2 | 549 |
| CP000148\_Gmet\_0287 | 319623 | 320759 | True | COG0304 | 8e-07 | 62.0 | 4 | 260 |
| CP000148\_Gmet\_0288 | 320790 | 321503 | True | - | - | - | - | - |
| CP000148\_Gmet\_0289 | 321537 | 322541 | True | - | - | - | - | - |
| CP000148\_Gmet\_0290 | 322542 | 324599 | True | - | - | - | - | - |
