# Supplementary material for: Dissecting the bacterial type VI secretion system by a genome wide in silico analysis: what can be learned from available microbial genomic resources?
Source: BMC Genomics. 2009 Mar 12;10:104. doi: 10.1186/1471-2164-10-104 (PMC2660368; doi:10.1186/1471-2164-10-104)
Supplement: Additional file 7 — Detailed description of all identified T6SS gene clusters. Archive containing the detailed description of each identified T6SS locus as an HTML file. [file 1471-2164-10-104-S7.tgz › LociHTML/HTML/CP000148D.html]

Locus CP000148D on Geobacter metallireducens (strain GS-15 / ATCC 53774 / DSM 7210) chromosome, complete sequence.

import namespace="svg" implementation="#AdobeSVG"?


# Locus CP000148D

# List of CDS in T6SS locus CP000148D

|  |  |  |  |  |  |  |  |  |
| --- | --- | --- | --- | --- | --- | --- | --- | --- |
| Name | from | to | direct | COG | e-value | COG cover | COG hit start | COG hit end |
| CP000148\_Gmet\_3306 | 3720361 | 3721503 | False | COG1960 | 8e-87 | 98.0 | 2 | 387 |
| CP000148\_Gmet\_3307 | 3721550 | 3722692 | False | COG1960 | 8e-88 | 99.0 | 2 | 391 |
| CP000148\_Gmet\_3308 | 3722946 | 3724592 | True | COG3829 | 3e-123 | 79.0 | 117 | 560 |
| CP000148\_Gmet\_3309 | 3724596 | 3725057 | False | COG2816 | 4e-12 | 44.0 | 146 | 270 |
| CP000148\_Gmet\_3310 | 3725357 | 3725911 | True | COG3521 | 5e-10 | 96.0 | 1 | 154 |
| CP000148\_Gmet\_3311 | 3725908 | 3727299 | True | COG3522 | 6e-47 | 100.0 | 1 | 446 |
| CP000148\_Gmet\_3312 | 3727303 | 3727713 | True | COG3518 | 6e-16 | 86.0 | 13 | 148 |
| CP000148\_Gmet\_3313 | 3727743 | 3729473 | True | COG3519 | 1e-109 | 99.0 | 7 | 621 |
| CP000148\_Gmet\_3314 | 3729437 | 3730426 | True | COG3520 | 1e-47 | 97.0 | 1 | 328 |
| CP000148\_Gmet\_3315 | 3730457 | 3733093 | True | COG0542 | 0.0 | 98.0 | 1 | 777 |
| CP000148\_Gmet\_3316 | 3733090 | 3733536 | True | - | - | - | - | - |
| CP000148\_Gmet\_3317 | 3733840 | 3734340 | True | COG0835 | 2e-33 | 86.0 | 18 | 160 |
| CP000148\_Gmet\_3318 | 3734343 | 3734855 | True | - | - | - | - | - |
| CP000148\_Gmet\_3319 | 3734852 | 3735802 | True | COG1941 | 8e-49 | 97.0 | 1 | 241 |
| CP000148\_Gmet\_3320 | 3735780 | 3737201 | True | COG3259 | 3e-102 | 98.0 | 1 | 435 |
| CP000148\_Gmet\_3321 | 3737201 | 3737452 | True | - | - | - | - | - |
| CP000148\_Gmet\_3322 | 3737449 | 3737907 | True | - | - | - | - | - |
| CP000148\_Gmet\_3323 | 3737925 | 3738389 | True | COG0680 | 4e-16 | 91.0 | 7 | 152 |
