# Supplementary material for: Dissecting the bacterial type VI secretion system by a genome wide in silico analysis: what can be learned from available microbial genomic resources?
Source: BMC Genomics. 2009 Mar 12;10:104. doi: 10.1186/1471-2164-10-104 (PMC2660368; doi:10.1186/1471-2164-10-104)
Supplement: Additional file 7 — Detailed description of all identified T6SS gene clusters. Archive containing the detailed description of each identified T6SS locus as an HTML file. [file 1471-2164-10-104-S7.tgz › LociHTML/HTML/CP000151C.html]

Locus CP000151C on Burkholderia sp. (strain ATCC 17760 / NCIB 9086 / R18194 / 383) / 383) chromosome 1, complete sequence.

import namespace="svg" implementation="#AdobeSVG"?


# Locus CP000151C

# List of CDS in T6SS locus CP000151C

|  |  |  |  |  |  |  |  |  |
| --- | --- | --- | --- | --- | --- | --- | --- | --- |
| Name | from | to | direct | COG | e-value | COG cover | COG hit start | COG hit end |
| CP000151\_Bcep18194\_A3544 | 407294 | 407740 | False | - | - | - | - | - |
| CP000151\_Bcep18194\_A3545 | 407888 | 408526 | True | - | - | - | - | - |
| CP000151\_Bcep18194\_A3546 | 408597 | 409253 | False | COG3279 | 5e-14 | 51.0 | 116 | 240 |
| CP000151\_Bcep18194\_A3547 | 409580 | 409783 | True | - | - | - | - | - |
| CP000151\_Bcep18194\_A3548 | 409843 | 410643 | False | COG0834 | 8e-45 | 99.0 | 1 | 273 |
| CP000151\_Bcep18194\_A3549 | 410920 | 414144 | True | COG4253 | 9e-44 | 76.0 | 1 | 214 |
| CP000151\_Bcep18194\_A3549 | 410920 | 414144 | True | COG3501 | 2e-109 | 96.0 | 1 | 532 |
| CP000151\_Bcep18194\_A3550 | 414182 | 418864 | True | COG3209 | 2e-42 | 80.0 | 19 | 660 |
| CP000151\_Bcep18194\_A3551 | 418875 | 419201 | True | - | - | - | - | - |
| CP000151\_Bcep18194\_A3553 | 419980 | 420360 | True | - | - | - | - | - |
| CP000151\_Bcep18194\_A3554 | 420524 | 420841 | True | - | - | - | - | - |
| CP000151\_Bcep18194\_A3555 | 420931 | 421713 | False | COG3455 | 8e-45 | 95.0 | 14 | 262 |
| CP000151\_Bcep18194\_A3556 | 421710 | 423056 | False | COG3522 | 5e-111 | 100.0 | 1 | 446 |
| CP000151\_Bcep18194\_A3557 | 423160 | 423771 | False | COG3521 | 1e-27 | 87.0 | 9 | 147 |
| CP000151\_Bcep18194\_A3558 | 424146 | 424784 | True | - | - | - | - | - |
| CP000151\_Bcep18194\_A3559 | 424831 | 425346 | True | COG3516 | 2e-48 | 98.0 | 2 | 168 |
| CP000151\_Bcep18194\_A3560 | 425362 | 426852 | True | COG3517 | 0.0 | 99.0 | 2 | 495 |
| CP000151\_Bcep18194\_A3561 | 426923 | 427426 | True | COG3157 | 4e-32 | 97.0 | 5 | 162 |
| CP000151\_Bcep18194\_A3562 | 427490 | 427975 | True | COG3518 | 1e-33 | 96.0 | 4 | 155 |
| CP000151\_Bcep18194\_A3563 | 428053 | 429888 | True | COG3519 | 5e-154 | 99.0 | 2 | 620 |
| CP000151\_Bcep18194\_A3564 | 429852 | 430952 | True | COG3520 | 1e-64 | 100.0 | 1 | 335 |
| CP000151\_Bcep18194\_A3565 | 430996 | 433665 | True | COG0542 | 0.0 | 100.0 | 1 | 786 |
| CP000151\_Bcep18194\_A3566 | 433708 | 434829 | True | COG3515 | 7e-31 | 98.0 | 7 | 346 |
| CP000151\_Bcep18194\_A3567 | 434898 | 437576 | True | COG4253 | 9e-55 | 100.0 | 1 | 278 |
| CP000151\_Bcep18194\_A3567 | 434898 | 437576 | True | COG3501 | 2e-114 | 96.0 | 1 | 532 |
| CP000151\_Bcep18194\_A3568 | 437578 | 439014 | True | - | - | - | - | - |
| CP000151\_Bcep18194\_A3569 | 439016 | 439762 | True | - | - | - | - | - |
| CP000151\_Bcep18194\_A3570 | 439808 | 440638 | True | - | - | - | - | - |
| CP000151\_Bcep18194\_A3571 | 440736 | 441443 | True | - | - | - | - | - |
| CP000151\_Bcep18194\_A3572 | 441518 | 441784 | True | - | - | - | - | - |
| CP000151\_Bcep18194\_A3573 | 441925 | 442887 | False | COG2885 | 1e-26 | 84.0 | 27 | 187 |
| CP000151\_Bcep18194\_A3574 | 442892 | 443881 | False | COG3913 | 4e-34 | 93.0 | 5 | 216 |
| CP000151\_Bcep18194\_A3575 | 443878 | 447825 | False | COG3523 | 7e-122 | 47.0 | 5 | 573 |
| CP000151\_Bcep18194\_A3575 | 443878 | 447825 | False | COG3523 | 4e-109 | 52.0 | 565 | 1188 |
| CP000151\_Bcep18194\_A3576 | 448127 | 448738 | False | - | - | - | - | - |
| CP000151\_Bcep18194\_A3577 | 449094 | 450047 | True | - | - | - | - | - |
| CP000151\_Bcep18194\_A3578 | 450161 | 451279 | True | COG0598 | 1e-47 | 92.0 | 23 | 320 |
| CP000151\_Bcep18194\_A3579 | 451296 | 451448 | False | COG4317 | 2e-09 | 53.0 | 1 | 50 |
| CP000151\_Bcep18194\_A3580 | 451473 | 452450 | False | COG0604 | 2e-43 | 100.0 | 1 | 326 |
| CP000151\_Bcep18194\_A3581 | 452612 | 453532 | True | COG0583 | 2e-27 | 97.0 | 5 | 295 |
