# Supplementary material for: Dissecting the bacterial type VI secretion system by a genome wide in silico analysis: what can be learned from available microbial genomic resources?
Source: BMC Genomics. 2009 Mar 12;10:104. doi: 10.1186/1471-2164-10-104 (PMC2660368; doi:10.1186/1471-2164-10-104)
Supplement: Additional file 7 — Detailed description of all identified T6SS gene clusters. Archive containing the detailed description of each identified T6SS locus as an HTML file. [file 1471-2164-10-104-S7.tgz › LociHTML/HTML/CP000152E.html]

Locus CP000152E on Burkholderia sp. (strain ATCC 17760 / NCIB 9086 / R18194 / 383) / 383) chromosome 2, complete sequence.

import namespace="svg" implementation="#AdobeSVG"?


# Locus CP000152E

# List of CDS in T6SS locus CP000152E

|  |  |  |  |  |  |  |  |  |
| --- | --- | --- | --- | --- | --- | --- | --- | --- |
| Name | from | to | direct | COG | e-value | COG cover | COG hit start | COG hit end |
| CP000152\_Bcep18194\_B0970 | 1082518 | 1084107 | True | COG0306 | 1e-39 | 65.0 | 1 | 215 |
| CP000152\_Bcep18194\_B0970 | 1082518 | 1084107 | True | COG0306 | 3e-24 | 36.0 | 207 | 326 |
| CP000152\_Bcep18194\_B0971 | 1084189 | 1085940 | True | COG4986 | 4e-142 | 100.0 | 1 | 523 |
| CP000152\_Bcep18194\_B0972 | 1085952 | 1087298 | True | COG1116 | 2e-83 | 100.0 | 1 | 248 |
| CP000152\_Bcep18194\_B0972 | 1085952 | 1087298 | True | COG4754 | 5e-28 | 96.0 | 7 | 157 |
| CP000152\_Bcep18194\_B0973 | 1087349 | 1088467 | False | COG3515 | 1e-22 | 96.0 | 5 | 338 |
| CP000152\_Bcep18194\_B0974 | 1088464 | 1089519 | False | COG3520 | 1e-77 | 95.0 | 14 | 334 |
| CP000152\_Bcep18194\_B0975 | 1089519 | 1091408 | False | COG3519 | 0.0 | 100.0 | 1 | 621 |
| CP000152\_Bcep18194\_B0976 | 1091446 | 1092018 | False | COG3518 | 2e-18 | 93.0 | 7 | 153 |
| CP000152\_Bcep18194\_B0977 | 1092011 | 1092865 | False | COG4455 | 5e-60 | 91.0 | 17 | 267 |
| CP000152\_Bcep18194\_B0978 | 1092862 | 1093512 | False | - | - | - | - | - |
| CP000152\_Bcep18194\_B0979 | 1094050 | 1096731 | True | COG0542 | 0.0 | 96.0 | 1 | 761 |
| CP000152\_Bcep18194\_B0980 | 1096768 | 1097307 | True | COG3516 | 3e-55 | 100.0 | 1 | 169 |
| CP000152\_Bcep18194\_B0981 | 1097335 | 1098828 | True | COG3517 | 0.0 | 100.0 | 1 | 495 |
| CP000152\_Bcep18194\_B0982 | 1098922 | 1099407 | True | COG3157 | 1e-36 | 100.0 | 1 | 162 |
| CP000152\_Bcep18194\_B0983 | 1099487 | 1099990 | True | COG3521 | 2e-31 | 90.0 | 1 | 144 |
| CP000152\_Bcep18194\_B0984 | 1100022 | 1101368 | True | COG3522 | 1e-121 | 99.0 | 1 | 445 |
| CP000152\_Bcep18194\_B0985 | 1101550 | 1104081 | True | COG4253 | 8e-38 | 75.0 | 3 | 212 |
| CP000152\_Bcep18194\_B0985 | 1101550 | 1104081 | True | COG3501 | 3e-108 | 95.0 | 25 | 550 |
| CP000152\_Bcep18194\_B0986 | 1104053 | 1104976 | True | - | - | - | - | - |
| CP000152\_Bcep18194\_B0987 | 1104973 | 1105494 | True | - | - | - | - | - |
| CP000152\_Bcep18194\_B0988 | 1105593 | 1106120 | True | - | - | - | - | - |
| CP000152\_Bcep18194\_B0989 | 1106133 | 1108508 | True | - | - | - | - | - |
| CP000152\_Bcep18194\_B0990 | 1108534 | 1108815 | True | COG4104 | 3e-10 | 83.0 | 10 | 91 |
| CP000152\_Bcep18194\_B0991 | 1108818 | 1110122 | True | COG3455 | 1e-44 | 91.0 | 20 | 260 |
| CP000152\_Bcep18194\_B0991 | 1108818 | 1110122 | True | COG1360 | 6e-30 | 56.0 | 104 | 242 |
| CP000152\_Bcep18194\_B0992 | 1110148 | 1114257 | True | COG3523 | 2e-31 | 16.0 | 2 | 195 |
| CP000152\_Bcep18194\_B0992 | 1110148 | 1114257 | True | COG3523 | 0.0 | 83.0 | 193 | 1188 |
| CP000152\_Bcep18194\_B0993 | 1114275 | 1115489 | True | COG3455 | 1e-28 | 88.0 | 10 | 240 |
| CP000152\_Bcep18194\_B0993 | 1114275 | 1115489 | True | COG1360 | 1e-19 | 58.0 | 100 | 242 |
| CP000152\_Bcep18194\_B0994 | 1115779 | 1116720 | True | - | - | - | - | - |
| CP000152\_Bcep18194\_B0995 | 1116699 | 1117577 | False | COG1737 | 1e-41 | 97.0 | 1 | 275 |
| CP000152\_Bcep18194\_B0996 | 1117941 | 1119692 | True | COG0405 | 1e-177 | 99.0 | 5 | 539 |
| CP000152\_Bcep18194\_B0997 | 1119870 | 1121246 | True | COG1301 | 4e-87 | 97.0 | 2 | 405 |
