# Supplementary material for: Dissecting the bacterial type VI secretion system by a genome wide in silico analysis: what can be learned from available microbial genomic resources?
Source: BMC Genomics. 2009 Mar 12;10:104. doi: 10.1186/1471-2164-10-104 (PMC2660368; doi:10.1186/1471-2164-10-104)
Supplement: Additional file 7 — Detailed description of all identified T6SS gene clusters. Archive containing the detailed description of each identified T6SS locus as an HTML file. [file 1471-2164-10-104-S7.tgz › LociHTML/HTML/CP000152F.html]

Locus CP000152F on Burkholderia sp. (strain ATCC 17760 / NCIB 9086 / R18194 / 383) / 383) chromosome 2, complete sequence.

import namespace="svg" implementation="#AdobeSVG"?


# Locus CP000152F

# List of CDS in T6SS locus CP000152F

|  |  |  |  |  |  |  |  |  |
| --- | --- | --- | --- | --- | --- | --- | --- | --- |
| Name | from | to | direct | COG | e-value | COG cover | COG hit start | COG hit end |
| CP000152\_Bcep18194\_B1578 | 1787577 | 1792247 | False | COG3209 | 4e-22 | 88.0 | 2 | 705 |
| CP000152\_Bcep18194\_B1579 | 1792244 | 1793542 | False | - | - | - | - | - |
| CP000152\_Bcep18194\_B1580 | 1793560 | 1794183 | False | - | - | - | - | - |
| CP000152\_Bcep18194\_B1581 | 1794239 | 1796512 | False | COG3501 | 3e-144 | 96.0 | 23 | 550 |
| CP000152\_Bcep18194\_B1582 | 1796509 | 1799172 | False | COG0542 | 0.0 | 99.0 | 1 | 785 |
| CP000152\_Bcep18194\_B1583 | 1799185 | 1800288 | False | COG3520 | 8e-65 | 96.0 | 12 | 335 |
| CP000152\_Bcep18194\_B1584 | 1800285 | 1802156 | False | COG3519 | 5e-166 | 100.0 | 1 | 621 |
| CP000152\_Bcep18194\_B1585 | 1802161 | 1802709 | False | COG3518 | 8e-18 | 94.0 | 2 | 149 |
| CP000152\_Bcep18194\_B1586 | 1802726 | 1803217 | False | COG3157 | 2e-20 | 98.0 | 1 | 159 |
| CP000152\_Bcep18194\_B1587 | 1803289 | 1804797 | False | COG3517 | 0.0 | 99.0 | 5 | 495 |
| CP000152\_Bcep18194\_B1588 | 1804790 | 1805344 | False | COG3516 | 1e-51 | 98.0 | 1 | 167 |
| CP000152\_Bcep18194\_B1589 | 1805418 | 1806533 | False | COG3515 | 2e-18 | 97.0 | 6 | 341 |
| CP000152\_Bcep18194\_B1590 | 1806520 | 1808829 | False | COG0515 | 3e-29 | 72.0 | 1 | 277 |
| CP000152\_Bcep18194\_B1591 | 1808910 | 1809605 | False | COG3913 | 3e-15 | 94.0 | 1 | 215 |
| CP000152\_Bcep18194\_B1592 | 1809587 | 1813213 | False | COG3523 | 0.0 | 99.0 | 1 | 1185 |
| CP000152\_Bcep18194\_B1593 | 1813216 | 1814538 | False | COG3455 | 1e-58 | 96.0 | 6 | 258 |
| CP000152\_Bcep18194\_B1593 | 1813216 | 1814538 | False | COG1360 | 3e-29 | 62.0 | 90 | 242 |
| CP000152\_Bcep18194\_B1594 | 1814554 | 1815972 | False | COG3522 | 1e-127 | 99.0 | 3 | 446 |
| CP000152\_Bcep18194\_B1595 | 1815969 | 1816484 | False | COG3521 | 3e-23 | 90.0 | 1 | 144 |
| CP000152\_Bcep18194\_B1596 | 1817013 | 1817501 | True | - | - | - | - | - |
| CP000152\_Bcep18194\_B1597 | 1817546 | 1819003 | True | COG3456 | 2e-30 | 93.0 | 22 | 425 |
| CP000152\_Bcep18194\_B1598 | 1819058 | 1820536 | False | COG3706 | 9e-47 | 42.0 | 248 | 434 |
| CP000152\_Bcep18194\_B1599 | 1820552 | 1820797 | False | - | - | - | - | - |
| CP000152\_Bcep18194\_B1600 | 1820938 | 1822383 | False | COG1012 | 5e-132 | 98.0 | 1 | 467 |
| CP000152\_Bcep18194\_B1601 | 1822536 | 1823834 | False | COG2010 | 7e-07 | 88.0 | 18 | 150 |
| CP000152\_Bcep18194\_B1602 | 1823850 | 1826072 | False | COG1529 | 4e-90 | 97.0 | 5 | 720 |
