# Supplementary material for: Dissecting the bacterial type VI secretion system by a genome wide in silico analysis: what can be learned from available microbial genomic resources?
Source: BMC Genomics. 2009 Mar 12;10:104. doi: 10.1186/1471-2164-10-104 (PMC2660368; doi:10.1186/1471-2164-10-104)
Supplement: Additional file 7 — Detailed description of all identified T6SS gene clusters. Archive containing the detailed description of each identified T6SS locus as an HTML file. [file 1471-2164-10-104-S7.tgz › LociHTML/HTML/CP000155B.html]

Locus CP000155B on Hahella chejuensis (strain KCTC 2396) chromosome, complete sequence.

import namespace="svg" implementation="#AdobeSVG"?


# Locus CP000155B

# List of CDS in T6SS locus CP000155B

|  |  |  |  |  |  |  |  |  |
| --- | --- | --- | --- | --- | --- | --- | --- | --- |
| Name | from | to | direct | COG | e-value | COG cover | COG hit start | COG hit end |
| CP000155\_HCH\_04242 | 4384561 | 4386051 | False | COG0515 | 1e-39 | 72.0 | 1 | 279 |
| CP000155\_HCH\_04243 | 4386092 | 4386241 | False | - | - | - | - | - |
| CP000155\_HCH\_04244 | 4386334 | 4388241 | False | COG4249 | 8e-08 | 23.0 | 2 | 92 |
| CP000155\_HCH\_04244 | 4386334 | 4388241 | False | COG0790 | 6e-14 | 35.0 | 51 | 155 |
| CP000155\_HCH\_04245 | 4388448 | 4389425 | False | - | - | - | - | - |
| CP000155\_HCH\_04246 | 4389751 | 4391028 | True | COG3515 | 1e-19 | 97.0 | 8 | 346 |
| CP000155\_HCH\_04247 | 4391108 | 4391641 | True | COG3516 | 8e-41 | 98.0 | 3 | 169 |
| CP000155\_HCH\_04248 | 4391641 | 4393134 | True | COG3517 | 0.0 | 99.0 | 1 | 492 |
| CP000155\_HCH\_04249 | 4393264 | 4394772 | True | COG3517 | 1e-114 | 86.0 | 64 | 493 |
| CP000155\_HCH\_04250 | 4394782 | 4395273 | True | COG3518 | 7e-18 | 94.0 | 7 | 154 |
| CP000155\_HCH\_04251 | 4395273 | 4397102 | True | COG3519 | 1e-150 | 100.0 | 1 | 621 |
| CP000155\_HCH\_04252 | 4397066 | 4398187 | True | COG3520 | 2e-50 | 100.0 | 1 | 335 |
| CP000155\_HCH\_04253 | 4398184 | 4400943 | True | COG0542 | 0.0 | 98.0 | 3 | 777 |
| CP000155\_HCH\_04254 | 4400969 | 4401670 | True | - | - | - | - | - |
| CP000155\_HCH\_04255 | 4401707 | 4402120 | True | COG3607 | 1e-10 | 93.0 | 7 | 131 |
| CP000155\_HCH\_04256 | 4402227 | 4403156 | False | COG0583 | 1e-27 | 98.0 | 2 | 293 |
| CP000155\_HCH\_04257 | 4403275 | 4403571 | True | COG1359 | 2e-11 | 77.0 | 3 | 79 |
| CP000155\_HCH\_04258 | 4403616 | 4403864 | True | - | - | - | - | - |
| CP000155\_HCH\_04259 | 4403898 | 4404284 | True | COG0251 | 9e-17 | 88.0 | 14 | 128 |
| CP000155\_HCH\_04260 | 4404317 | 4405390 | False | COG5380 | 8e-15 | 93.0 | 19 | 282 |
| CP000155\_HCH\_04261 | 4405524 | 4406504 | False | COG1075 | 1e-12 | 83.0 | 58 | 336 |
