# Supplementary material for: Dissecting the bacterial type VI secretion system by a genome wide in silico analysis: what can be learned from available microbial genomic resources?
Source: BMC Genomics. 2009 Mar 12;10:104. doi: 10.1186/1471-2164-10-104 (PMC2660368; doi:10.1186/1471-2164-10-104)
Supplement: Additional file 7 — Detailed description of all identified T6SS gene clusters. Archive containing the detailed description of each identified T6SS locus as an HTML file. [file 1471-2164-10-104-S7.tgz › LociHTML/HTML/CP000155C.html]

Locus CP000155C on Hahella chejuensis (strain KCTC 2396) chromosome, complete sequence.

import namespace="svg" implementation="#AdobeSVG"?


# Locus CP000155C

# List of CDS in T6SS locus CP000155C

|  |  |  |  |  |  |  |  |  |
| --- | --- | --- | --- | --- | --- | --- | --- | --- |
| Name | from | to | direct | COG | e-value | COG cover | COG hit start | COG hit end |
| CP000155\_HCH\_04287 | 4432871 | 4433452 | True | - | - | - | - | - |
| CP000155\_HCH\_04288 | 4433459 | 4434040 | True | - | - | - | - | - |
| CP000155\_HCH\_04289 | 4434027 | 4434629 | True | - | - | - | - | - |
| CP000155\_HCH\_04290 | 4434636 | 4435217 | True | - | - | - | - | - |
| CP000155\_HCH\_04291 | 4435221 | 4435814 | True | - | - | - | - | - |
| CP000155\_HCH\_04292 | 4435848 | 4436624 | True | - | - | - | - | - |
| CP000155\_HCH\_04293 | 4436771 | 4437598 | False | - | - | - | - | - |
| CP000155\_HCH\_04294 | 4438269 | 4438757 | True | COG3157 | 1e-14 | 96.0 | 3 | 158 |
| CP000155\_HCH\_04295 | 4438922 | 4439200 | False | - | - | - | - | - |
| CP000155\_HCH\_04296 | 4439169 | 4439894 | False | COG0631 | 7e-48 | 92.0 | 6 | 247 |
| CP000155\_HCH\_04297 | 4439965 | 4440657 | False | COG3913 | 2e-20 | 92.0 | 7 | 217 |
| CP000155\_HCH\_04298 | 4440744 | 4444202 | False | COG3523 | 0.0 | 98.0 | 13 | 1187 |
| CP000155\_HCH\_04299 | 4444288 | 4445169 | False | COG3455 | 5e-59 | 98.0 | 1 | 258 |
| CP000155\_HCH\_04300 | 4445274 | 4446611 | False | COG3522 | 4e-135 | 100.0 | 1 | 446 |
| CP000155\_HCH\_04301 | 4446626 | 4447105 | False | COG3521 | 2e-19 | 87.0 | 7 | 145 |
| CP000155\_HCH\_04302 | 4447332 | 4449218 | False | COG3456 | 7e-12 | 51.0 | 22 | 244 |
| CP000155\_HCH\_04302 | 4447332 | 4449218 | False | COG3456 | 2e-29 | 66.0 | 136 | 422 |
| CP000155\_HCH\_04303 | 4449681 | 4450439 | True | COG2204 | 1e-17 | 31.0 | 2 | 148 |
| CP000155\_HCH\_04304 | 4450662 | 4450784 | False | - | - | - | - | - |
| CP000155\_HCH\_04305 | 4450774 | 4452399 | True | COG0784 | 9e-10 | 96.0 | 5 | 129 |
| CP000155\_HCH\_04306 | 4452406 | 4453095 | True | COG0642 | 4e-18 | 62.0 | 118 | 328 |
| CP000155\_HCH\_04307 | 4453142 | 4453909 | False | COG3318 | 6e-13 | 96.0 | 8 | 215 |
