# Supplementary material for: Dissecting the bacterial type VI secretion system by a genome wide in silico analysis: what can be learned from available microbial genomic resources?
Source: BMC Genomics. 2009 Mar 12;10:104. doi: 10.1186/1471-2164-10-104 (PMC2660368; doi:10.1186/1471-2164-10-104)
Supplement: Additional file 7 — Detailed description of all identified T6SS gene clusters. Archive containing the detailed description of each identified T6SS locus as an HTML file. [file 1471-2164-10-104-S7.tgz › LociHTML/HTML/CP000243A.html]

Locus CP000243A on Escherichia coli (strain UTI89 / UPEC) chromosome, complete sequence.

import namespace="svg" implementation="#AdobeSVG"?


# Locus CP000243A

# List of CDS in T6SS locus CP000243A

|  |  |  |  |  |  |  |  |  |
| --- | --- | --- | --- | --- | --- | --- | --- | --- |
| Name | from | to | direct | COG | e-value | COG cover | COG hit start | COG hit end |
| CP000243\_UTI89\_C0230 | 237565 | 238923 | False | COG1388 | 6e-09 | 93.0 | 1 | 116 |
| CP000243\_UTI89\_C0230 | 237565 | 238923 | False | COG0741 | 2e-12 | 93.0 | 1 | 278 |
| CP000243\_UTI89\_C0231 | 238995 | 239750 | False | COG0491 | 2e-27 | 92.0 | 19 | 252 |
| CP000243\_UTI89\_C0232 | 239766 | 240506 | True | COG2226 | 1e-07 | 26.0 | 106 | 168 |
| CP000243\_UTI89\_C0233 | 240503 | 241081 | False | COG0328 | 3e-57 | 99.0 | 2 | 154 |
| CP000243\_UTI89\_C0234 | 241026 | 241766 | True | COG0847 | 7e-51 | 95.0 | 8 | 240 |
| CP000243\_UTI89\_C0236 | 242304 | 243089 | True | - | - | - | - | - |
| CP000243\_UTI89\_C0237 | 243429 | 243908 | False | COG3157 | 1e-38 | 98.0 | 1 | 159 |
| CP000243\_UTI89\_C0238 | 243926 | 245368 | False | COG3515 | 5e-42 | 96.0 | 10 | 344 |
| CP000243\_UTI89\_C0239 | 245295 | 248762 | False | COG3523 | 0.0 | 99.0 | 5 | 1188 |
| CP000243\_UTI89\_C0240 | 248842 | 250284 | False | COG3515 | 3e-25 | 76.0 | 19 | 284 |
| CP000243\_UTI89\_C0241 | 250289 | 251032 | False | - | - | - | - | - |
| CP000243\_UTI89\_C0242 | 251029 | 253788 | False | COG0542 | 9e-126 | 59.0 | 1 | 464 |
| CP000243\_UTI89\_C0242 | 251029 | 253788 | False | COG0542 | 3e-96 | 47.0 | 414 | 786 |
| CP000243\_UTI89\_C0243 | 253798 | 254562 | False | COG3455 | 5e-77 | 92.0 | 19 | 260 |
| CP000243\_UTI89\_C0244 | 254567 | 255913 | False | COG3522 | 2e-135 | 99.0 | 4 | 446 |
| CP000243\_UTI89\_C0245 | 255916 | 256440 | False | COG3521 | 1e-34 | 99.0 | 1 | 158 |
| CP000243\_UTI89\_C0246 | 256437 | 257729 | False | COG3456 | 3e-94 | 99.0 | 2 | 430 |
| CP000243\_UTI89\_C0247 | 257734 | 258783 | False | COG3520 | 9e-84 | 94.0 | 15 | 332 |
| CP000243\_UTI89\_C0248 | 258747 | 260588 | False | COG3519 | 1e-148 | 99.0 | 2 | 621 |
| CP000243\_UTI89\_C0249 | 260594 | 261019 | False | COG3518 | 4e-20 | 96.0 | 4 | 154 |
| CP000243\_UTI89\_C0250 | 261024 | 262508 | False | COG3517 | 0.0 | 99.0 | 1 | 493 |
| CP000243\_UTI89\_C0251 | 262531 | 263034 | False | COG3516 | 2e-33 | 94.0 | 8 | 167 |
| CP000243\_UTI89\_C0252 | 263740 | 264258 | True | COG3157 | 5e-52 | 98.0 | 1 | 160 |
| CP000243\_UTI89\_C0253 | 264479 | 266461 | True | COG3501 | 8e-155 | 97.0 | 11 | 545 |
| CP000243\_UTI89\_C0254 | 266568 | 267614 | True | COG5351 | 2e-43 | 87.0 | 1 | 322 |
| CP000243\_UTI89\_C0255 | 267607 | 269046 | True | - | - | - | - | - |
| CP000243\_UTI89\_C0256 | 269021 | 269311 | True | - | - | - | - | - |
| CP000243\_UTI89\_C0257 | 270562 | 271065 | True | - | - | - | - | - |
| CP000243\_UTI89\_C0258 | 271135 | 271647 | True | - | - | - | - | - |
