# Supplementary material for: Dissecting the bacterial type VI secretion system by a genome wide in silico analysis: what can be learned from available microbial genomic resources?
Source: BMC Genomics. 2009 Mar 12;10:104. doi: 10.1186/1471-2164-10-104 (PMC2660368; doi:10.1186/1471-2164-10-104)
Supplement: Additional file 7 — Detailed description of all identified T6SS gene clusters. Archive containing the detailed description of each identified T6SS locus as an HTML file. [file 1471-2164-10-104-S7.tgz › LociHTML/HTML/CP000243D.html]

Locus CP000243D on Escherichia coli (strain UTI89 / UPEC) chromosome, complete sequence.

import namespace="svg" implementation="#AdobeSVG"?


# Locus CP000243D

# List of CDS in T6SS locus CP000243D

|  |  |  |  |  |  |  |  |  |
| --- | --- | --- | --- | --- | --- | --- | --- | --- |
| Name | from | to | direct | COG | e-value | COG cover | COG hit start | COG hit end |
| CP000243\_UTI89\_C3181 | 3097390 | 3097683 | True | - | - | - | - | - |
| CP000243\_UTI89\_C3182 | 3097423 | 3097653 | False | - | - | - | - | - |
| CP000243\_UTI89\_C3183 | 3097806 | 3099047 | True | COG0520 | 2e-124 | 100.0 | 1 | 405 |
| CP000243\_UTI89\_C3184 | 3099047 | 3099490 | True | COG2166 | 3e-45 | 97.0 | 1 | 141 |
| CP000243\_UTI89\_C3185 | 3099541 | 3100347 | False | COG1179 | 2e-113 | 99.0 | 1 | 262 |
| CP000243\_UTI89\_C3186 | 3100424 | 3101854 | False | COG2821 | 4e-130 | 100.0 | 1 | 373 |
| CP000243\_UTI89\_C3190 | 3102641 | 3103162 | True | COG3516 | 3e-39 | 96.0 | 6 | 168 |
| CP000243\_UTI89\_C3191 | 3103215 | 3104759 | True | COG3517 | 0.0 | 100.0 | 1 | 495 |
| CP000243\_UTI89\_C3192 | 3104777 | 3106114 | True | COG3522 | 6e-94 | 99.0 | 2 | 446 |
| CP000243\_UTI89\_C3193 | 3106111 | 3106776 | True | COG3455 | 3e-28 | 80.0 | 43 | 254 |
| CP000243\_UTI89\_C3194 | 3106789 | 3108441 | True | COG2885 | 1e-26 | 83.0 | 33 | 190 |
| CP000243\_UTI89\_C3195 | 3108478 | 3109080 | False | - | - | - | - | - |
| CP000243\_UTI89\_C3196 | 3108499 | 3108990 | True | COG3157 | 5e-39 | 96.0 | 1 | 157 |
| CP000243\_UTI89\_C3197 | 3109182 | 3111827 | True | COG0542 | 0.0 | 99.0 | 1 | 781 |
| CP000243\_UTI89\_C3198 | 3111839 | 3114319 | True | COG4253 | 3e-43 | 98.0 | 2 | 276 |
| CP000243\_UTI89\_C3198 | 3111839 | 3114319 | True | COG3501 | 1e-86 | 98.0 | 8 | 549 |
| CP000243\_UTI89\_C3199 | 3114339 | 3116108 | True | - | - | - | - | - |
| CP000243\_UTI89\_C3200 | 3116083 | 3116916 | True | - | - | - | - | - |
| CP000243\_UTI89\_C3201 | 3116885 | 3117178 | True | - | - | - | - | - |
| CP000243\_UTI89\_C3202 | 3117642 | 3117902 | True | COG4104 | 3e-09 | 83.0 | 11 | 92 |
| CP000243\_UTI89\_C3203 | 3117829 | 3119040 | True | - | - | - | - | - |
| CP000243\_UTI89\_C3204 | 3119050 | 3122403 | True | COG3523 | 2e-97 | 96.0 | 45 | 1188 |
| CP000243\_UTI89\_C3205 | 3122369 | 3124006 | True | COG3515 | 7e-18 | 86.0 | 9 | 309 |
| CP000243\_UTI89\_C3206 | 3125577 | 3126218 | True | - | - | - | - | - |
| CP000243\_UTI89\_C3207 | 3126499 | 3126918 | True | - | - | - | - | - |
| CP000243\_UTI89\_C3208 | 3130072 | 3130608 | True | COG3521 | 2e-15 | 86.0 | 8 | 145 |
| CP000243\_UTI89\_C3209 | 3130612 | 3131040 | True | COG3518 | 2e-13 | 91.0 | 7 | 150 |
| CP000243\_UTI89\_C3210 | 3131040 | 3132416 | True | COG3515 | 6e-11 | 46.0 | 32 | 192 |
| CP000243\_UTI89\_C3211 | 3131590 | 3131742 | False | - | - | - | - | - |
| CP000243\_UTI89\_C3212 | 3132718 | 3133674 | False | COG0111 | 3e-61 | 82.0 | 48 | 313 |
| CP000243\_UTI89\_C3213 | 3133737 | 3134333 | False | COG0794 | 1e-48 | 94.0 | 4 | 193 |
| CP000243\_UTI89\_C3214 | 3134336 | 3135511 | False | COG1168 | 2e-101 | 99.0 | 4 | 388 |
| CP000243\_UTI89\_C3215 | 3135511 | 3137091 | False | COG1263 | 3e-42 | 97.0 | 1 | 384 |
| CP000243\_UTI89\_C3215 | 3135511 | 3137091 | False | COG1264 | 2e-16 | 89.0 | 2 | 80 |
