# Supplementary material for: Dissecting the bacterial type VI secretion system by a genome wide in silico analysis: what can be learned from available microbial genomic resources?
Source: BMC Genomics. 2009 Mar 12;10:104. doi: 10.1186/1471-2164-10-104 (PMC2660368; doi:10.1186/1471-2164-10-104)
Supplement: Additional file 7 — Detailed description of all identified T6SS gene clusters. Archive containing the detailed description of each identified T6SS locus as an HTML file. [file 1471-2164-10-104-S7.tgz › LociHTML/HTML/CP000247A.html]

Locus CP000247A on Escherichia coli O6:K15:H31 (strain 536 / UPEC) chromosome, complete sequence.

import namespace="svg" implementation="#AdobeSVG"?


# Locus CP000247A

# List of CDS in T6SS locus CP000247A

|  |  |  |  |  |  |  |  |  |
| --- | --- | --- | --- | --- | --- | --- | --- | --- |
| Name | from | to | direct | COG | e-value | COG cover | COG hit start | COG hit end |
| CP000247\_ECP\_0217 | 236887 | 238245 | False | COG1388 | 1e-08 | 93.0 | 1 | 116 |
| CP000247\_ECP\_0217 | 236887 | 238245 | False | COG0741 | 1e-12 | 93.0 | 1 | 278 |
| CP000247\_ECP\_0218 | 238317 | 239072 | False | COG0491 | 1e-27 | 92.0 | 19 | 252 |
| CP000247\_ECP\_0219 | 239103 | 239828 | True | COG2226 | 8e-08 | 26.0 | 106 | 168 |
| CP000247\_ECP\_0220 | 239825 | 240292 | False | COG0328 | 4e-56 | 99.0 | 2 | 154 |
| CP000247\_ECP\_0221 | 240348 | 241088 | True | COG0847 | 2e-50 | 95.0 | 8 | 240 |
| CP000247\_ECP\_0223 | 241626 | 242411 | True | - | - | - | - | - |
| CP000247\_ECP\_0224 | 242751 | 243251 | False | COG3157 | 7e-39 | 98.0 | 1 | 159 |
| CP000247\_ECP\_0225 | 243248 | 244606 | False | COG3515 | 5e-41 | 96.0 | 12 | 344 |
| CP000247\_ECP\_0226 | 244617 | 248144 | False | COG3523 | 0.0 | 99.0 | 2 | 1188 |
| CP000247\_ECP\_0227 | 248164 | 249672 | False | COG3515 | 2e-25 | 76.0 | 19 | 284 |
| CP000247\_ECP\_0228 | 249611 | 250354 | False | - | - | - | - | - |
| CP000247\_ECP\_0229 | 250351 | 253113 | False | COG0542 | 1e-124 | 59.0 | 1 | 464 |
| CP000247\_ECP\_0229 | 250351 | 253113 | False | COG0542 | 4e-96 | 47.0 | 414 | 786 |
| CP000247\_ECP\_0230 | 253123 | 253887 | False | COG3455 | 1e-76 | 92.0 | 19 | 260 |
| CP000247\_ECP\_0231 | 253892 | 255238 | False | COG3522 | 2e-134 | 99.0 | 4 | 446 |
| CP000247\_ECP\_0232 | 255241 | 255765 | False | COG3521 | 2e-35 | 99.0 | 1 | 158 |
| CP000247\_ECP\_0233 | 255762 | 257150 | False | COG3456 | 2e-93 | 99.0 | 2 | 430 |
| CP000247\_ECP\_0234 | 257059 | 258108 | False | COG3520 | 8e-85 | 94.0 | 15 | 332 |
| CP000247\_ECP\_0235 | 258072 | 259922 | False | COG3519 | 1e-148 | 99.0 | 3 | 621 |
| CP000247\_ECP\_0236 | 259919 | 260344 | False | COG3518 | 1e-19 | 96.0 | 4 | 154 |
| CP000247\_ECP\_0237 | 260349 | 261833 | False | COG3517 | 0.0 | 99.0 | 1 | 493 |
| CP000247\_ECP\_0238 | 261856 | 262359 | False | COG3516 | 2e-33 | 94.0 | 8 | 167 |
| CP000247\_ECP\_0239 | 263065 | 263583 | True | COG3157 | 5e-52 | 98.0 | 1 | 160 |
| CP000247\_ECP\_0240 | 263804 | 265786 | True | COG3501 | 4e-153 | 97.0 | 11 | 545 |
| CP000247\_ECP\_0241 | 265893 | 266939 | True | COG5351 | 2e-43 | 87.0 | 1 | 322 |
| CP000247\_ECP\_0242 | 266932 | 268371 | True | - | - | - | - | - |
| CP000247\_ECP\_0243 | 268346 | 268636 | True | - | - | - | - | - |
| CP000247\_ECP\_0244 | 268894 | 269073 | True | - | - | - | - | - |
| CP000247\_ECP\_0246 | 269350 | 269658 | True | COG5433 | 1e-12 | 84.0 | 1 | 102 |
| CP000247\_ECP\_0247 | 269887 | 270390 | True | - | - | - | - | - |
| CP000247\_ECP\_0248 | 270484 | 270972 | True | - | - | - | - | - |
