# Supplementary material for: Dissecting the bacterial type VI secretion system by a genome wide in silico analysis: what can be learned from available microbial genomic resources?
Source: BMC Genomics. 2009 Mar 12;10:104. doi: 10.1186/1471-2164-10-104 (PMC2660368; doi:10.1186/1471-2164-10-104)
Supplement: Additional file 7 — Detailed description of all identified T6SS gene clusters. Archive containing the detailed description of each identified T6SS locus as an HTML file. [file 1471-2164-10-104-S7.tgz › LociHTML/HTML/CP000247C.html]

Locus CP000247C on Escherichia coli O6:K15:H31 (strain 536 / UPEC) chromosome, complete sequence.

import namespace="svg" implementation="#AdobeSVG"?


# Locus CP000247C

# List of CDS in T6SS locus CP000247C

|  |  |  |  |  |  |  |  |  |
| --- | --- | --- | --- | --- | --- | --- | --- | --- |
| Name | from | to | direct | COG | e-value | COG cover | COG hit start | COG hit end |
| CP000247\_ECP\_2793 | 2936796 | 2938001 | True | COG0520 | 2e-124 | 100.0 | 1 | 405 |
| CP000247\_ECP\_2794 | 2938001 | 2938444 | True | COG2166 | 3e-45 | 97.0 | 1 | 141 |
| CP000247\_ECP\_2795 | 2938495 | 2939301 | False | COG1179 | 2e-113 | 99.0 | 1 | 262 |
| CP000247\_ECP\_2796 | 2939378 | 2940676 | False | COG2821 | 2e-130 | 100.0 | 1 | 373 |
| CP000247\_ECP\_2800 | 2941612 | 2942112 | True | COG3516 | 1e-39 | 96.0 | 6 | 168 |
| CP000247\_ECP\_2801 | 2942171 | 2943709 | True | COG3517 | 0.0 | 100.0 | 1 | 495 |
| CP000247\_ECP\_2802 | 2943727 | 2945064 | True | COG3522 | 2e-94 | 99.0 | 2 | 446 |
| CP000247\_ECP\_2803 | 2945061 | 2945726 | True | COG3455 | 3e-28 | 80.0 | 43 | 254 |
| CP000247\_ECP\_2804 | 2945739 | 2947391 | True | COG2885 | 9e-27 | 83.0 | 33 | 190 |
| CP000247\_ECP\_2805 | 2947449 | 2947940 | True | COG3157 | 3e-39 | 96.0 | 1 | 157 |
| CP000247\_ECP\_2806 | 2948132 | 2950768 | True | COG0542 | 0.0 | 99.0 | 1 | 781 |
| CP000247\_ECP\_2807 | 2950780 | 2953269 | True | COG4253 | 2e-54 | 92.0 | 3 | 258 |
| CP000247\_ECP\_2807 | 2950780 | 2953269 | True | COG3501 | 4e-85 | 95.0 | 13 | 539 |
| CP000247\_ECP\_2808 | 2953335 | 2954153 | True | - | - | - | - | - |
| CP000247\_ECP\_2809 | 2954167 | 2954760 | True | - | - | - | - | - |
| CP000247\_ECP\_2810 | 2954805 | 2957324 | True | COG4253 | 2e-53 | 98.0 | 3 | 277 |
| CP000247\_ECP\_2810 | 2954805 | 2957324 | True | COG3501 | 2e-86 | 95.0 | 13 | 539 |
| CP000247\_ECP\_2811 | 2957317 | 2958105 | True | - | - | - | - | - |
| CP000247\_ECP\_2812 | 2958190 | 2959110 | True | - | - | - | - | - |
| CP000247\_ECP\_2813 | 2959367 | 2960581 | True | - | - | - | - | - |
| CP000247\_ECP\_2814 | 2960533 | 2963964 | True | COG3523 | 2e-100 | 99.0 | 6 | 1188 |
| CP000247\_ECP\_2815 | 2963930 | 2965567 | True | COG3515 | 5e-18 | 88.0 | 4 | 309 |
| CP000247\_ECP\_2816 | 2965573 | 2967003 | True | - | - | - | - | - |
| CP000247\_ECP\_2817 | 2966996 | 2967400 | True | - | - | - | - | - |
| CP000247\_ECP\_2818 | 2967475 | 2967792 | True | COG3519 | 7e-27 | 16.0 | 1 | 103 |
| CP000247\_ECP\_2819 | 2967749 | 2969236 | True | COG3519 | 6e-107 | 85.0 | 92 | 621 |
| CP000247\_ECP\_2820 | 2969200 | 2970279 | True | COG3520 | 5e-59 | 95.0 | 14 | 334 |
| CP000247\_ECP\_2821 | 2970260 | 2970796 | True | COG3521 | 2e-15 | 86.0 | 8 | 145 |
| CP000247\_ECP\_2822 | 2970800 | 2971228 | True | COG3518 | 7e-14 | 90.0 | 9 | 150 |
| CP000247\_ECP\_2823 | 2971228 | 2972604 | True | COG3515 | 3e-11 | 46.0 | 32 | 192 |
| CP000247\_ECP\_2824 | 2972904 | 2973851 | False | COG0111 | 5e-61 | 82.0 | 48 | 313 |
| CP000247\_ECP\_2825 | 2973923 | 2974519 | False | COG0794 | 4e-49 | 94.0 | 4 | 193 |
| CP000247\_ECP\_2826 | 2974522 | 2975697 | False | COG1168 | 4e-102 | 99.0 | 4 | 388 |
| CP000247\_ECP\_2827 | 2975697 | 2977226 | False | COG1264 | 6e-17 | 89.0 | 2 | 80 |
| CP000247\_ECP\_2827 | 2975697 | 2977226 | False | COG1263 | 1e-39 | 95.0 | 20 | 393 |
