# Supplementary material for: Dissecting the bacterial type VI secretion system by a genome wide in silico analysis: what can be learned from available microbial genomic resources?
Source: BMC Genomics. 2009 Mar 12;10:104. doi: 10.1186/1471-2164-10-104 (PMC2660368; doi:10.1186/1471-2164-10-104)
Supplement: Additional file 7 — Detailed description of all identified T6SS gene clusters. Archive containing the detailed description of each identified T6SS locus as an HTML file. [file 1471-2164-10-104-S7.tgz › LociHTML/HTML/CP000264A.html]

Locus CP000264A on Jannaschia sp. (strain CCS1) chromosome, complete sequence.

import namespace="svg" implementation="#AdobeSVG"?


# Locus CP000264A

# List of CDS in T6SS locus CP000264A

|  |  |  |  |  |  |  |  |  |
| --- | --- | --- | --- | --- | --- | --- | --- | --- |
| Name | from | to | direct | COG | e-value | COG cover | COG hit start | COG hit end |
| CP000264\_Jann\_3014 | 3033323 | 3034330 | False | - | - | - | - | - |
| CP000264\_Jann\_3015 | 3034350 | 3035471 | False | - | - | - | - | - |
| CP000264\_Jann\_3016 | 3035468 | 3036430 | False | COG5351 | 4e-15 | 89.0 | 2 | 330 |
| CP000264\_Jann\_3017 | 3036445 | 3036804 | False | - | - | - | - | - |
| CP000264\_Jann\_3018 | 3037038 | 3037970 | False | - | - | - | - | - |
| CP000264\_Jann\_3019 | 3037974 | 3038411 | False | - | - | - | - | - |
| CP000264\_Jann\_3020 | 3038411 | 3040627 | False | COG3501 | 4e-122 | 99.0 | 2 | 548 |
| CP000264\_Jann\_3021 | 3040674 | 3041729 | False | - | - | - | - | - |
| CP000264\_Jann\_3022 | 3041821 | 3042297 | False | COG3157 | 7e-26 | 98.0 | 3 | 162 |
| CP000264\_Jann\_3023 | 3042365 | 3044959 | False | COG0542 | 0.0 | 96.0 | 1 | 761 |
| CP000264\_Jann\_3024 | 3045161 | 3046240 | True | COG3515 | 2e-24 | 96.0 | 13 | 345 |
| CP000264\_Jann\_3025 | 3046392 | 3046895 | True | COG3516 | 3e-47 | 98.0 | 1 | 167 |
| CP000264\_Jann\_3026 | 3046898 | 3048382 | True | COG3517 | 0.0 | 99.0 | 1 | 491 |
| CP000264\_Jann\_3027 | 3048384 | 3049781 | True | COG3517 | 1e-112 | 85.0 | 68 | 491 |
| CP000264\_Jann\_3028 | 3049778 | 3050575 | True | COG4455 | 3e-35 | 96.0 | 1 | 264 |
| CP000264\_Jann\_3029 | 3050568 | 3051110 | True | COG3518 | 2e-13 | 94.0 | 6 | 153 |
| CP000264\_Jann\_3030 | 3051107 | 3052885 | True | COG3519 | 3e-127 | 100.0 | 1 | 621 |
| CP000264\_Jann\_3031 | 3052882 | 3053868 | True | COG3520 | 8e-44 | 93.0 | 14 | 325 |
| CP000264\_Jann\_3032 | 3053865 | 3055115 | True | COG3456 | 2e-07 | 86.0 | 24 | 396 |
| CP000264\_Jann\_3033 | 3055112 | 3056443 | True | COG3522 | 2e-125 | 100.0 | 1 | 446 |
| CP000264\_Jann\_3034 | 3056440 | 3057903 | True | COG3455 | 1e-48 | 99.0 | 1 | 260 |
| CP000264\_Jann\_3034 | 3056440 | 3057903 | True | COG1360 | 3e-19 | 93.0 | 16 | 242 |
| CP000264\_Jann\_3035 | 3057900 | 3061373 | True | COG3523 | 0.0 | 98.0 | 13 | 1188 |
| CP000264\_Jann\_3036 | 3061370 | 3062806 | True | COG3913 | 2e-20 | 58.0 | 9 | 141 |
| CP000264\_Jann\_3036 | 3061370 | 3062806 | True | COG0631 | 1e-11 | 54.0 | 110 | 252 |
| CP000264\_Jann\_3037 | 3062843 | 3063940 | True | COG1596 | 2e-25 | 84.0 | 38 | 239 |
| CP000264\_Jann\_3038 | 3064221 | 3064841 | True | COG3707 | 2e-37 | 98.0 | 4 | 194 |
| CP000264\_Jann\_3039 | 3064838 | 3066007 | True | COG0715 | 2e-32 | 89.0 | 29 | 327 |
| CP000264\_Jann\_3040 | 3066280 | 3067638 | True | COG0715 | 4e-37 | 98.0 | 6 | 334 |
