# Supplementary material for: Dissecting the bacterial type VI secretion system by a genome wide in silico analysis: what can be learned from available microbial genomic resources?
Source: BMC Genomics. 2009 Mar 12;10:104. doi: 10.1186/1471-2164-10-104 (PMC2660368; doi:10.1186/1471-2164-10-104)
Supplement: Additional file 7 — Detailed description of all identified T6SS gene clusters. Archive containing the detailed description of each identified T6SS locus as an HTML file. [file 1471-2164-10-104-S7.tgz › LociHTML/HTML/CP000270B.html]

Locus CP000270B on Burkholderia xenovorans (strain LB400) chromosome 1, complete sequence.

import namespace="svg" implementation="#AdobeSVG"?


# Locus CP000270B

# List of CDS in T6SS locus CP000270B

|  |  |  |  |  |  |  |  |  |
| --- | --- | --- | --- | --- | --- | --- | --- | --- |
| Name | from | to | direct | COG | e-value | COG cover | COG hit start | COG hit end |
| CP000270\_Bxeno\_A2326 | 2604751 | 2605251 | True | COG5517 | 2e-08 | 85.0 | 11 | 150 |
| CP000270\_Bxeno\_A2327 | 2605278 | 2606594 | True | - | - | - | - | - |
| CP000270\_Bxeno\_A2328 | 2606806 | 2607183 | True | - | - | - | - | - |
| CP000270\_Bxeno\_A2329 | 2607167 | 2607709 | True | - | - | - | - | - |
| CP000270\_Bxeno\_A2330 | 2607706 | 2608830 | True | COG0154 | 4e-69 | 81.0 | 79 | 467 |
| CP000270\_Bxeno\_A2331 | 2608888 | 2609631 | True | COG1028 | 8e-29 | 98.0 | 3 | 250 |
| CP000270\_Bxeno\_A2332 | 2609791 | 2610888 | False | COG3515 | 2e-21 | 96.0 | 6 | 340 |
| CP000270\_Bxeno\_A2333 | 2611074 | 2611778 | False | - | - | - | - | - |
| CP000270\_Bxeno\_A2334 | 2612313 | 2615063 | True | COG0542 | 0.0 | 98.0 | 1 | 776 |
| CP000270\_Bxeno\_A2335 | 2615103 | 2615651 | True | COG3516 | 3e-56 | 96.0 | 1 | 163 |
| CP000270\_Bxeno\_A2336 | 2615685 | 2617178 | True | COG3517 | 0.0 | 100.0 | 1 | 495 |
| CP000270\_Bxeno\_A2337 | 2617345 | 2617746 | True | - | - | - | - | - |
| CP000270\_Bxeno\_A2338 | 2617743 | 2618234 | True | - | - | - | - | - |
| CP000270\_Bxeno\_A2339 | 2618277 | 2618759 | True | COG3157 | 7e-36 | 100.0 | 1 | 162 |
| CP000270\_Bxeno\_A2340 | 2618874 | 2619374 | True | COG3521 | 6e-31 | 97.0 | 1 | 155 |
| CP000270\_Bxeno\_A2341 | 2619387 | 2620733 | True | COG3522 | 2e-127 | 100.0 | 1 | 446 |
| CP000270\_Bxeno\_A2342 | 2620821 | 2622152 | True | COG3455 | 2e-46 | 98.0 | 2 | 260 |
| CP000270\_Bxeno\_A2342 | 2620821 | 2622152 | True | COG1360 | 2e-25 | 56.0 | 104 | 240 |
| CP000270\_Bxeno\_A2343 | 2622274 | 2623164 | False | COG2801 | 3e-19 | 90.0 | 20 | 230 |
| CP000270\_Bxeno\_A2344 | 2623161 | 2623448 | False | - | - | - | - | - |
| CP000270\_Bxeno\_A2345 | 2623466 | 2627563 | True | COG3523 | 1e-35 | 15.0 | 7 | 191 |
| CP000270\_Bxeno\_A2345 | 2623466 | 2627563 | True | COG3523 | 0.0 | 83.0 | 191 | 1186 |
| CP000270\_Bxeno\_A2346 | 2627759 | 2628160 | False | COG2372 | 1e-16 | 95.0 | 5 | 125 |
| CP000270\_Bxeno\_A2347 | 2628177 | 2629073 | False | - | - | - | - | - |
| CP000270\_Bxeno\_A2348 | 2629182 | 2629652 | False | - | - | - | - | - |
| CP000270\_Bxeno\_A2349 | 2629888 | 2631240 | True | COG2358 | 2e-17 | 92.0 | 7 | 304 |
| CP000270\_Bxeno\_A2350 | 2631257 | 2632111 | False | COG3109 | 8e-14 | 84.0 | 1 | 176 |
| CP000270\_Bxeno\_A2351 | 2632268 | 2633179 | True | COG0583 | 5e-35 | 98.0 | 1 | 294 |
