# Supplementary material for: Dissecting the bacterial type VI secretion system by a genome wide in silico analysis: what can be learned from available microbial genomic resources?
Source: BMC Genomics. 2009 Mar 12;10:104. doi: 10.1186/1471-2164-10-104 (PMC2660368; doi:10.1186/1471-2164-10-104)
Supplement: Additional file 7 — Detailed description of all identified T6SS gene clusters. Archive containing the detailed description of each identified T6SS locus as an HTML file. [file 1471-2164-10-104-S7.tgz › LociHTML/HTML/CP000282B.html]

Locus CP000282B on Saccharophagus degradans (strain 2-40 / ATCC 43961 / DSM 17024) chromosome, complete sequence.

import namespace="svg" implementation="#AdobeSVG"?


# Locus CP000282B

# List of CDS in T6SS locus CP000282B

|  |  |  |  |  |  |  |  |  |
| --- | --- | --- | --- | --- | --- | --- | --- | --- |
| Name | from | to | direct | COG | e-value | COG cover | COG hit start | COG hit end |
| CP000282\_Sde\_1515 | 1954421 | 1955377 | False | - | - | - | - | - |
| CP000282\_Sde\_1516 | 1955429 | 1956334 | False | - | - | - | - | - |
| CP000282\_Sde\_1517 | 1956365 | 1957363 | False | COG0332 | 4e-50 | 96.0 | 4 | 314 |
| CP000282\_Sde\_1518 | 1958049 | 1958891 | True | COG2996 | 5e-65 | 99.0 | 2 | 286 |
| CP000282\_Sde\_1519 | 1958894 | 1959325 | True | - | - | - | - | - |
| CP000282\_Sde\_1520 | 1959354 | 1960079 | False | COG0631 | 2e-44 | 90.0 | 14 | 251 |
| CP000282\_Sde\_1521 | 1960096 | 1960872 | False | COG3913 | 3e-22 | 95.0 | 2 | 217 |
| CP000282\_Sde\_1522 | 1960893 | 1964438 | False | COG3523 | 0.0 | 98.0 | 13 | 1187 |
| CP000282\_Sde\_1523 | 1964450 | 1965859 | False | COG3455 | 3e-43 | 100.0 | 1 | 262 |
| CP000282\_Sde\_1523 | 1964450 | 1965859 | False | COG1360 | 1e-23 | 55.0 | 103 | 238 |
| CP000282\_Sde\_1524 | 1965894 | 1967231 | False | COG3522 | 2e-122 | 100.0 | 1 | 446 |
| CP000282\_Sde\_1525 | 1967303 | 1967797 | False | COG3521 | 3e-19 | 98.0 | 1 | 157 |
| CP000282\_Sde\_1526 | 1967820 | 1969016 | False | COG3456 | 2e-17 | 95.0 | 1 | 412 |
| CP000282\_Sde\_1527 | 1969782 | 1971800 | True | COG0515 | 2e-28 | 91.0 | 2 | 353 |
| CP000282\_Sde\_1528 | 1971811 | 1972926 | True | COG3515 | 4e-12 | 84.0 | 52 | 343 |
| CP000282\_Sde\_1529 | 1973009 | 1973512 | True | COG3516 | 9e-50 | 97.0 | 1 | 164 |
| CP000282\_Sde\_1530 | 1973515 | 1974990 | True | COG3517 | 0.0 | 98.0 | 4 | 491 |
| CP000282\_Sde\_1531 | 1975157 | 1976710 | True | COG3517 | 6e-130 | 87.0 | 58 | 490 |
| CP000282\_Sde\_1532 | 1976725 | 1977558 | True | COG4455 | 3e-45 | 95.0 | 6 | 265 |
| CP000282\_Sde\_1533 | 1977562 | 1978059 | True | COG3518 | 2e-18 | 88.0 | 5 | 143 |
| CP000282\_Sde\_1534 | 1978052 | 1979887 | True | COG3519 | 5e-96 | 100.0 | 1 | 621 |
| CP000282\_Sde\_1535 | 1979911 | 1980900 | True | COG3520 | 4e-48 | 92.0 | 21 | 331 |
| CP000282\_Sde\_1536 | 1980897 | 1983590 | True | COG0542 | 0.0 | 98.0 | 2 | 777 |
| CP000282\_Sde\_1537 | 1983718 | 1984206 | True | COG3157 | 1e-20 | 92.0 | 3 | 152 |
| CP000282\_Sde\_1538 | 1984325 | 1986190 | True | COG3501 | 1e-109 | 100.0 | 1 | 550 |
| CP000282\_Sde\_1539 | 1986300 | 1986809 | True | COG1451 | 2e-22 | 76.0 | 51 | 220 |
| CP000282\_Sde\_1540 | 1986830 | 1987195 | False | - | - | - | - | - |
| CP000282\_Sde\_1541 | 1987316 | 1987741 | True | COG2346 | 3e-13 | 86.0 | 10 | 124 |
| CP000282\_Sde\_1542 | 1987808 | 1989745 | True | COG0550 | 2e-138 | 97.0 | 1 | 558 |
| CP000282\_Sde\_1543 | 1989943 | 1990329 | True | - | - | - | - | - |
| CP000282\_Sde\_1544 | 1990462 | 1991199 | True | COG0412 | 4e-31 | 98.0 | 3 | 235 |
