# Supplementary material for: Dissecting the bacterial type VI secretion system by a genome wide in silico analysis: what can be learned from available microbial genomic resources?
Source: BMC Genomics. 2009 Mar 12;10:104. doi: 10.1186/1471-2164-10-104 (PMC2660368; doi:10.1186/1471-2164-10-104)
Supplement: Additional file 7 — Detailed description of all identified T6SS gene clusters. Archive containing the detailed description of each identified T6SS locus as an HTML file. [file 1471-2164-10-104-S7.tgz › LociHTML/HTML/CP000285A.html]

Locus CP000285A on Chromohalobacter salexigens (strain DSM 3043 / ATCC BAA-138 / NCIMB 13768) chromosome, complete sequence.

import namespace="svg" implementation="#AdobeSVG"?


# Locus CP000285A

# List of CDS in T6SS locus CP000285A

|  |  |  |  |  |  |  |  |  |
| --- | --- | --- | --- | --- | --- | --- | --- | --- |
| Name | from | to | direct | COG | e-value | COG cover | COG hit start | COG hit end |
| CP000285\_Csal\_2251 | 2526600 | 2527553 | False | - | - | - | - | - |
| CP000285\_Csal\_2252 | 2527564 | 2530191 | False | - | - | - | - | - |
| CP000285\_Csal\_2253 | 2530188 | 2531084 | False | - | - | - | - | - |
| CP000285\_Csal\_2254 | 2531081 | 2533708 | False | COG4253 | 4e-43 | 83.0 | 1 | 231 |
| CP000285\_Csal\_2254 | 2531081 | 2533708 | False | COG3501 | 9e-127 | 95.0 | 10 | 532 |
| CP000285\_Csal\_2255 | 2533831 | 2534100 | True | COG4104 | 6e-14 | 81.0 | 11 | 90 |
| CP000285\_Csal\_2256 | 2534091 | 2535245 | False | - | - | - | - | - |
| CP000285\_Csal\_2257 | 2535248 | 2536363 | False | COG3515 | 3e-22 | 92.0 | 13 | 331 |
| CP000285\_Csal\_2258 | 2536669 | 2537199 | False | COG3157 | 5e-25 | 98.0 | 1 | 160 |
| CP000285\_Csal\_2259 | 2537272 | 2537520 | True | - | - | - | - | - |
| CP000285\_Csal\_2260 | 2537550 | 2540042 | False | COG3523 | 2e-90 | 54.0 | 7 | 657 |
| CP000285\_Csal\_2260 | 2537550 | 2540042 | False | COG2885 | 4e-22 | 64.0 | 66 | 188 |
| CP000285\_Csal\_2261 | 2540070 | 2540987 | False | COG3913 | 1e-07 | 44.0 | 8 | 108 |
| CP000285\_Csal\_2262 | 2541021 | 2545028 | False | COG3523 | 1e-74 | 47.0 | 2 | 565 |
| CP000285\_Csal\_2262 | 2541021 | 2545028 | False | COG3523 | 2e-49 | 53.0 | 553 | 1184 |
| CP000285\_Csal\_2263 | 2545043 | 2545756 | False | COG3455 | 2e-32 | 84.0 | 38 | 258 |
| CP000285\_Csal\_2264 | 2545753 | 2547099 | False | COG3522 | 9e-88 | 99.0 | 1 | 444 |
| CP000285\_Csal\_2265 | 2547192 | 2547737 | False | COG3521 | 1e-18 | 86.0 | 1 | 137 |
| CP000285\_Csal\_2266 | 2547783 | 2548430 | True | - | - | - | - | - |
| CP000285\_Csal\_2267 | 2548679 | 2549434 | True | - | - | - | - | - |
| CP000285\_Csal\_2268 | 2549491 | 2550024 | True | COG3516 | 8e-53 | 99.0 | 2 | 169 |
| CP000285\_Csal\_2269 | 2550036 | 2551535 | True | COG3517 | 0.0 | 99.0 | 1 | 494 |
| CP000285\_Csal\_2270 | 2551658 | 2552140 | True | COG3518 | 3e-16 | 98.0 | 1 | 154 |
| CP000285\_Csal\_2271 | 2552140 | 2553975 | True | COG3519 | 2e-151 | 100.0 | 1 | 621 |
| CP000285\_Csal\_2272 | 2554023 | 2555015 | True | COG3520 | 2e-61 | 87.0 | 28 | 321 |
| CP000285\_Csal\_2273 | 2555012 | 2557681 | True | COG0542 | 0.0 | 98.0 | 1 | 777 |
| CP000285\_Csal\_2274 | 2557718 | 2560333 | True | COG4253 | 3e-48 | 79.0 | 1 | 221 |
| CP000285\_Csal\_2274 | 2557718 | 2560333 | True | COG3501 | 2e-126 | 95.0 | 10 | 532 |
| CP000285\_Csal\_2275 | 2560337 | 2561224 | True | - | - | - | - | - |
| CP000285\_Csal\_2276 | 2561246 | 2563888 | True | - | - | - | - | - |
| CP000285\_Csal\_2277 | 2563890 | 2564963 | True | - | - | - | - | - |
| CP000285\_Csal\_2278 | 2565323 | 2567218 | True | - | - | - | - | - |
