# Supplementary material for: Dissecting the bacterial type VI secretion system by a genome wide in silico analysis: what can be learned from available microbial genomic resources?
Source: BMC Genomics. 2009 Mar 12;10:104. doi: 10.1186/1471-2164-10-104 (PMC2660368; doi:10.1186/1471-2164-10-104)
Supplement: Additional file 7 — Detailed description of all identified T6SS gene clusters. Archive containing the detailed description of each identified T6SS locus as an HTML file. [file 1471-2164-10-104-S7.tgz › LociHTML/HTML/CP000305A.html]

Locus CP000305A on Yersinia pestis (biovar Antiqua Nepal516, strain Nepal516) chromosome, complete sequence.

import namespace="svg" implementation="#AdobeSVG"?


# Locus CP000305A

# List of CDS in T6SS locus CP000305A

|  |  |  |  |  |  |  |  |  |
| --- | --- | --- | --- | --- | --- | --- | --- | --- |
| Name | from | to | direct | COG | e-value | COG cover | COG hit start | COG hit end |
| CP000305\_YPN\_0028 | 45430 | 45666 | True | - | - | - | - | - |
| CP000305\_YPN\_0029 | 45670 | 46779 | True | COG3839 | 9e-125 | 100.0 | 1 | 338 |
| CP000305\_YPN\_0030 | 46850 | 48121 | True | COG4580 | 4e-150 | 99.0 | 2 | 429 |
| CP000305\_YPN\_0031 | 48362 | 49273 | True | - | - | - | - | - |
| CP000305\_YPN\_0032 | 49612 | 50022 | True | - | - | - | - | - |
| CP000305\_YPN\_0033 | 50174 | 50692 | False | COG3157 | 1e-50 | 98.0 | 1 | 160 |
| CP000305\_YPN\_0034 | 51216 | 51713 | True | COG3516 | 1e-49 | 98.0 | 2 | 167 |
| CP000305\_YPN\_0035 | 51781 | 53262 | True | COG3517 | 0.0 | 99.0 | 1 | 493 |
| CP000305\_YPN\_0036 | 53269 | 53709 | True | COG3518 | 9e-35 | 100.0 | 1 | 157 |
| CP000305\_YPN\_0037 | 53709 | 54935 | True | COG3519 | 6e-95 | 59.0 | 3 | 374 |
| CP000305\_YPN\_0038 | 54825 | 55610 | False | COG2801 | 1e-17 | 92.0 | 16 | 230 |
| CP000305\_YPN\_0039 | 55664 | 56308 | False | COG2963 | 6e-12 | 95.0 | 6 | 116 |
| CP000305\_YPN\_0040 | 56921 | 59557 | False | COG2352 | 0.0 | 99.0 | 3 | 910 |
| CP000305\_YPN\_0041 | 59931 | 61094 | False | COG0624 | 3e-65 | 97.0 | 10 | 409 |
