# Supplementary material for: Dissecting the bacterial type VI secretion system by a genome wide in silico analysis: what can be learned from available microbial genomic resources?
Source: BMC Genomics. 2009 Mar 12;10:104. doi: 10.1186/1471-2164-10-104 (PMC2660368; doi:10.1186/1471-2164-10-104)
Supplement: Additional file 7 — Detailed description of all identified T6SS gene clusters. Archive containing the detailed description of each identified T6SS locus as an HTML file. [file 1471-2164-10-104-S7.tgz › LociHTML/HTML/CP000305B.html]

Locus CP000305B on Yersinia pestis (biovar Antiqua Nepal516, strain Nepal516) chromosome, complete sequence.

import namespace="svg" implementation="#AdobeSVG"?


# Locus CP000305B

# List of CDS in T6SS locus CP000305B

|  |  |  |  |  |  |  |  |  |
| --- | --- | --- | --- | --- | --- | --- | --- | --- |
| Name | from | to | direct | COG | e-value | COG cover | COG hit start | COG hit end |
| CP000305\_YPN\_0369 | 445330 | 447672 | False | COG1452 | 0.0 | 100.0 | 1 | 784 |
| CP000305\_YPN\_0370 | 447857 | 448690 | True | COG1076 | 6e-35 | 100.0 | 1 | 174 |
| CP000305\_YPN\_0371 | 448988 | 449608 | False | COG0564 | 1e-59 | 72.0 | 80 | 289 |
| CP000305\_YPN\_0372 | 449652 | 449777 | True | - | - | - | - | - |
| CP000305\_YPN\_0373 | 450831 | 451574 | False | COG5419 | 2e-48 | 100.0 | 1 | 160 |
| CP000305\_YPN\_0374 | 451919 | 452917 | True | COG3515 | 6e-54 | 98.0 | 5 | 346 |
| CP000305\_YPN\_0375 | 452928 | 453488 | True | COG3516 | 1e-57 | 99.0 | 2 | 169 |
| CP000305\_YPN\_0376 | 453488 | 454999 | True | COG3517 | 0.0 | 100.0 | 1 | 495 |
| CP000305\_YPN\_0377 | 455162 | 455680 | True | COG3157 | 7e-36 | 100.0 | 1 | 162 |
| CP000305\_YPN\_0378 | 455754 | 456197 | True | COG3518 | 8e-31 | 95.0 | 4 | 153 |
| CP000305\_YPN\_0379 | 456230 | 458074 | True | COG3519 | 0.0 | 99.0 | 1 | 617 |
| CP000305\_YPN\_0380 | 458067 | 459050 | True | COG3520 | 2e-83 | 98.0 | 4 | 334 |
| CP000305\_YPN\_0381 | 459068 | 461656 | True | COG0542 | 0.0 | 99.0 | 1 | 783 |
| CP000305\_YPN\_0382 | 461760 | 464108 | True | COG3501 | 5e-154 | 95.0 | 10 | 533 |
| CP000305\_YPN\_0383 | 464121 | 466340 | True | COG1357 | 4e-15 | 83.0 | 18 | 215 |
| CP000305\_YPN\_0383 | 464121 | 466340 | True | COG5351 | 1e-07 | 50.0 | 68 | 253 |
| CP000305\_YPN\_0384 | 466366 | 467469 | True | COG1357 | 1e-18 | 99.0 | 3 | 238 |
| CP000305\_YPN\_0385 | 467462 | 468079 | True | - | - | - | - | - |
| CP000305\_YPN\_0386 | 468085 | 468450 | True | - | - | - | - | - |
| CP000305\_YPN\_0387 | 468443 | 468934 | True | COG3521 | 1e-34 | 98.0 | 3 | 158 |
| CP000305\_YPN\_0388 | 469054 | 470409 | True | COG3522 | 2e-141 | 100.0 | 1 | 446 |
| CP000305\_YPN\_0389 | 470406 | 472016 | True | COG3455 | 1e-72 | 100.0 | 1 | 262 |
| CP000305\_YPN\_0389 | 470406 | 472016 | True | COG1360 | 2e-27 | 56.0 | 108 | 244 |
| CP000305\_YPN\_0390 | 472025 | 475519 | True | COG3523 | 0.0 | 98.0 | 12 | 1187 |
| CP000305\_YPN\_0391 | 475541 | 475894 | True | - | - | - | - | - |
| CP000305\_YPN\_0392 | 476150 | 479056 | False | COG0553 | 2e-72 | 98.0 | 8 | 861 |
| CP000305\_YPN\_0393 | 479517 | 481886 | False | COG0417 | 0.0 | 98.0 | 5 | 788 |
