# Supplementary material for: Dissecting the bacterial type VI secretion system by a genome wide in silico analysis: what can be learned from available microbial genomic resources?
Source: BMC Genomics. 2009 Mar 12;10:104. doi: 10.1186/1471-2164-10-104 (PMC2660368; doi:10.1186/1471-2164-10-104)
Supplement: Additional file 7 — Detailed description of all identified T6SS gene clusters. Archive containing the detailed description of each identified T6SS locus as an HTML file. [file 1471-2164-10-104-S7.tgz › LociHTML/HTML/CP000305C.html]

Locus CP000305C on Yersinia pestis (biovar Antiqua Nepal516, strain Nepal516) chromosome, complete sequence.

import namespace="svg" implementation="#AdobeSVG"?


# Locus CP000305C

# List of CDS in T6SS locus CP000305C

|  |  |  |  |  |  |  |  |  |
| --- | --- | --- | --- | --- | --- | --- | --- | --- |
| Name | from | to | direct | COG | e-value | COG cover | COG hit start | COG hit end |
| CP000305\_YPN\_1426 | 1622569 | 1623051 | False | COG5435 | 7e-43 | 100.0 | 1 | 147 |
| CP000305\_YPN\_1427 | 1623048 | 1624730 | False | COG2885 | 1e-27 | 55.0 | 86 | 190 |
| CP000305\_YPN\_1428 | 1624731 | 1625483 | False | - | - | - | - | - |
| CP000305\_YPN\_1429 | 1625429 | 1626766 | False | - | - | - | - | - |
| CP000305\_YPN\_1430 | 1626806 | 1627837 | False | - | - | - | - | - |
| CP000305\_YPN\_1431 | 1627844 | 1629025 | False | COG3515 | 5e-50 | 98.0 | 1 | 341 |
| CP000305\_YPN\_1432 | 1629124 | 1630149 | False | COG3520 | 1e-97 | 100.0 | 1 | 335 |
| CP000305\_YPN\_1433 | 1630149 | 1632029 | False | COG3519 | 0.0 | 100.0 | 1 | 621 |
| CP000305\_YPN\_1434 | 1632812 | 1635487 | True | COG0542 | 0.0 | 98.0 | 1 | 777 |
| CP000305\_YPN\_1435 | 1635688 | 1636233 | True | COG3539 | 1e-14 | 100.0 | 1 | 184 |
| CP000305\_YPN\_1436 | 1636390 | 1637151 | True | COG3121 | 3e-58 | 94.0 | 12 | 234 |
| CP000305\_YPN\_1437 | 1637279 | 1638829 | True | COG3188 | 1e-110 | 56.0 | 7 | 474 |
| CP000305\_YPN\_1438 | 1638851 | 1639291 | False | COG3328 | 1e-36 | 36.0 | 238 | 375 |
| CP000305\_YPN\_1439 | 1639336 | 1640058 | False | COG3328 | 6e-61 | 57.0 | 1 | 218 |
| CP000305\_YPN\_1440 | 1640083 | 1641273 | True | COG3188 | 2e-61 | 44.0 | 464 | 834 |
| CP000305\_YPN\_1441 | 1641258 | 1641866 | True | COG3539 | 4e-16 | 91.0 | 16 | 184 |
| CP000305\_YPN\_1442 | 1641971 | 1642495 | True | COG3516 | 8e-59 | 99.0 | 2 | 169 |
| CP000305\_YPN\_1443 | 1642519 | 1644021 | True | COG3517 | 0.0 | 100.0 | 1 | 495 |
| CP000305\_YPN\_1444 | 1644263 | 1644832 | True | COG3157 | 4e-42 | 100.0 | 1 | 162 |
| CP000305\_YPN\_1445 | 1645085 | 1645645 | True | COG3521 | 4e-39 | 100.0 | 1 | 159 |
| CP000305\_YPN\_1446 | 1645649 | 1646998 | True | COG3522 | 3e-158 | 99.0 | 2 | 446 |
| CP000305\_YPN\_1447 | 1646995 | 1647663 | True | COG3455 | 7e-18 | 42.0 | 40 | 151 |
| CP000305\_YPN\_1448 | 1647677 | 1648699 | True | COG4584 | 2e-58 | 100.0 | 1 | 278 |
| CP000305\_YPN\_1449 | 1648699 | 1649478 | True | COG1484 | 5e-64 | 99.0 | 2 | 254 |
| CP000305\_YPN\_1450 | 1649606 | 1649758 | True | COG3203 | 3e-07 | 14.0 | 303 | 354 |
| CP000305\_YPN\_1451 | 1650023 | 1650829 | False | COG3741 | 1e-92 | 99.0 | 3 | 272 |
| CP000305\_YPN\_1452 | 1650826 | 1652046 | False | COG1228 | 1e-101 | 98.0 | 1 | 401 |
| CP000305\_YPN\_1453 | 1652250 | 1653017 | False | COG2188 | 2e-55 | 100.0 | 1 | 236 |
