# Supplementary material for: Dissecting the bacterial type VI secretion system by a genome wide in silico analysis: what can be learned from available microbial genomic resources?
Source: BMC Genomics. 2009 Mar 12;10:104. doi: 10.1186/1471-2164-10-104 (PMC2660368; doi:10.1186/1471-2164-10-104)
Supplement: Additional file 7 — Detailed description of all identified T6SS gene clusters. Archive containing the detailed description of each identified T6SS locus as an HTML file. [file 1471-2164-10-104-S7.tgz › LociHTML/HTML/CP000305D.html]

Locus CP000305D on Yersinia pestis (biovar Antiqua Nepal516, strain Nepal516) chromosome, complete sequence.

import namespace="svg" implementation="#AdobeSVG"?


# Locus CP000305D

# List of CDS in T6SS locus CP000305D

|  |  |  |  |  |  |  |  |  |
| --- | --- | --- | --- | --- | --- | --- | --- | --- |
| Name | from | to | direct | COG | e-value | COG cover | COG hit start | COG hit end |
| CP000305\_YPN\_2214 | 2499538 | 2500005 | False | - | - | - | - | - |
| CP000305\_YPN\_2215 | 2500621 | 2501163 | True | COG1704 | 3e-55 | 99.0 | 2 | 185 |
| CP000305\_YPN\_2216 | 2501108 | 2503207 | True | COG4907 | 4e-08 | 30.0 | 412 | 594 |
| CP000305\_YPN\_2217 | 2503583 | 2503924 | True | COG2824 | 2e-46 | 100.0 | 1 | 112 |
| CP000305\_YPN\_2218 | 2503999 | 2504364 | False | - | - | - | - | - |
| CP000305\_YPN\_2219 | 2504371 | 2504850 | False | COG3518 | 4e-35 | 99.0 | 1 | 156 |
| CP000305\_YPN\_2220 | 2504979 | 2505785 | False | COG4455 | 9e-108 | 100.0 | 1 | 273 |
| CP000305\_YPN\_2221 | 2505805 | 2506653 | False | - | - | - | - | - |
| CP000305\_YPN\_2222 | 2507012 | 2509615 | False | COG3501 | 8e-150 | 97.0 | 6 | 539 |
| CP000305\_YPN\_2223 | 2509909 | 2513736 | False | COG3523 | 0.0 | 99.0 | 3 | 1185 |
| CP000305\_YPN\_2224 | 2513745 | 2514581 | False | COG1360 | 4e-32 | 59.0 | 95 | 240 |
| CP000305\_YPN\_2224 | 2513745 | 2514581 | False | COG3455 | 6e-30 | 42.0 | 151 | 262 |
| CP000305\_YPN\_2225 | 2514597 | 2515376 | False | COG1484 | 5e-64 | 99.0 | 2 | 254 |
| CP000305\_YPN\_2226 | 2515376 | 2516398 | False | COG4584 | 2e-58 | 100.0 | 1 | 278 |
| CP000305\_YPN\_2227 | 2517077 | 2517301 | True | - | - | - | - | - |
| CP000305\_YPN\_2228 | 2517311 | 2517652 | False | COG3839 | 6e-09 | 24.0 | 257 | 338 |
| CP000305\_YPN\_2229 | 2517538 | 2518428 | False | COG3839 | 9e-109 | 79.0 | 1 | 268 |
| CP000305\_YPN\_2230 | 2518453 | 2519280 | False | COG0395 | 2e-51 | 100.0 | 1 | 281 |
| CP000305\_YPN\_2231 | 2519273 | 2520133 | False | COG1175 | 1e-55 | 96.0 | 6 | 291 |
