# Supplementary material for: Dissecting the bacterial type VI secretion system by a genome wide in silico analysis: what can be learned from available microbial genomic resources?
Source: BMC Genomics. 2009 Mar 12;10:104. doi: 10.1186/1471-2164-10-104 (PMC2660368; doi:10.1186/1471-2164-10-104)
Supplement: Additional file 7 — Detailed description of all identified T6SS gene clusters. Archive containing the detailed description of each identified T6SS locus as an HTML file. [file 1471-2164-10-104-S7.tgz › LociHTML/HTML/CP000305F.html]

Locus CP000305F on Yersinia pestis (biovar Antiqua Nepal516, strain Nepal516) chromosome, complete sequence.

import namespace="svg" implementation="#AdobeSVG"?


# Locus CP000305F

# List of CDS in T6SS locus CP000305F

|  |  |  |  |  |  |  |  |  |
| --- | --- | --- | --- | --- | --- | --- | --- | --- |
| Name | from | to | direct | COG | e-value | COG cover | COG hit start | COG hit end |
| CP000305\_YPN\_2485 | 2788522 | 2789730 | True | COG3328 | 2e-112 | 98.0 | 1 | 375 |
| CP000305\_YPN\_2486 | 2789774 | 2790427 | False | - | - | - | - | - |
| CP000305\_YPN\_2487 | 2790655 | 2792418 | False | COG0488 | 8e-172 | 99.0 | 1 | 528 |
| CP000305\_YPN\_2488 | 2792933 | 2793556 | True | - | - | - | - | - |
| CP000305\_YPN\_2489 | 2793768 | 2795135 | False | COG3515 | 9e-41 | 96.0 | 13 | 346 |
| CP000305\_YPN\_2490 | 2795160 | 2795612 | False | COG3518 | 1e-27 | 98.0 | 3 | 157 |
| CP000305\_YPN\_2491 | 2795612 | 2796292 | False | COG3521 | 9e-36 | 98.0 | 1 | 157 |
| CP000305\_YPN\_2492 | 2796168 | 2797253 | False | COG3520 | 3e-85 | 97.0 | 1 | 328 |
| CP000305\_YPN\_2493 | 2797217 | 2798980 | False | COG3519 | 0.0 | 100.0 | 1 | 621 |
| CP000305\_YPN\_2494 | 2799201 | 2799656 | False | - | - | - | - | - |
| CP000305\_YPN\_2495 | 2799671 | 2800729 | False | - | - | - | - | - |
| CP000305\_YPN\_2496 | 2800747 | 2802348 | False | COG3515 | 8e-42 | 100.0 | 1 | 346 |
| CP000305\_YPN\_2497 | 2802392 | 2805814 | False | COG3523 | 0.0 | 100.0 | 1 | 1188 |
| CP000305\_YPN\_2498 | 2805811 | 2807043 | False | - | - | - | - | - |
| CP000305\_YPN\_2499 | 2807873 | 2808046 | True | - | - | - | - | - |
| CP000305\_YPN\_2500 | 2808155 | 2808475 | True | - | - | - | - | - |
| CP000305\_YPN\_2501 | 2808606 | 2808743 | True | - | - | - | - | - |
| CP000305\_YPN\_2502 | 2809208 | 2809678 | False | - | - | - | - | - |
| CP000305\_YPN\_2503 | 2809851 | 2812034 | False | - | - | - | - | - |
| CP000305\_YPN\_2504 | 2812050 | 2812310 | False | COG4253 | 2e-25 | 30.0 | 144 | 229 |
| CP000305\_YPN\_2505 | 2812464 | 2813237 | False | - | - | - | - | - |
| CP000305\_YPN\_2506 | 2813234 | 2815465 | False | - | - | - | - | - |
| CP000305\_YPN\_2507 | 2815550 | 2817898 | False | COG3501 | 1e-105 | 99.0 | 1 | 549 |
| CP000305\_YPN\_2507 | 2815550 | 2817898 | False | COG4253 | 6e-67 | 82.0 | 2 | 229 |
| CP000305\_YPN\_2508 | 2817901 | 2820543 | False | COG0542 | 0.0 | 99.0 | 1 | 784 |
| CP000305\_YPN\_2509 | 2820931 | 2821422 | False | COG3157 | 2e-40 | 98.0 | 1 | 160 |
| CP000305\_YPN\_2510 | 2821426 | 2823162 | False | COG2885 | 8e-27 | 94.0 | 12 | 190 |
| CP000305\_YPN\_2511 | 2823162 | 2823848 | False | COG3455 | 2e-48 | 91.0 | 21 | 260 |
| CP000305\_YPN\_2512 | 2823845 | 2825197 | False | COG3522 | 6e-133 | 99.0 | 2 | 446 |
| CP000305\_YPN\_2513 | 2825209 | 2826747 | False | COG3517 | 0.0 | 100.0 | 1 | 495 |
| CP000305\_YPN\_2514 | 2826796 | 2827296 | False | COG3516 | 6e-49 | 99.0 | 2 | 169 |
| CP000305\_YPN\_2515 | 2828285 | 2829133 | False | COG0331 | 2e-74 | 93.0 | 2 | 290 |
| CP000305\_YPN\_2516 | 2829232 | 2829480 | False | - | - | - | - | - |
| CP000305\_YPN\_2517 | 2829518 | 2830006 | False | - | - | - | - | - |
| CP000305\_YPN\_2518 | 2830045 | 2830833 | False | COG1028 | 4e-25 | 99.0 | 2 | 250 |
| CP000305\_YPN\_2519 | 2830836 | 2831612 | False | COG1024 | 5e-37 | 93.0 | 1 | 240 |
| CP000305\_YPN\_2520 | 2831590 | 2832309 | False | COG1024 | 6e-29 | 87.0 | 29 | 252 |
