# Supplementary material for: Dissecting the bacterial type VI secretion system by a genome wide in silico analysis: what can be learned from available microbial genomic resources?
Source: BMC Genomics. 2009 Mar 12;10:104. doi: 10.1186/1471-2164-10-104 (PMC2660368; doi:10.1186/1471-2164-10-104)
Supplement: Additional file 7 — Detailed description of all identified T6SS gene clusters. Archive containing the detailed description of each identified T6SS locus as an HTML file. [file 1471-2164-10-104-S7.tgz › LociHTML/HTML/CP000305G.html]

Locus CP000305G on Yersinia pestis (biovar Antiqua Nepal516, strain Nepal516) chromosome, complete sequence.

import namespace="svg" implementation="#AdobeSVG"?


# Locus CP000305G

# List of CDS in T6SS locus CP000305G

|  |  |  |  |  |  |  |  |  |
| --- | --- | --- | --- | --- | --- | --- | --- | --- |
| Name | from | to | direct | COG | e-value | COG cover | COG hit start | COG hit end |
| CP000305\_YPN\_3166 | 3565593 | 3566228 | False | COG4253 | 3e-62 | 81.0 | 4 | 229 |
| CP000305\_YPN\_3167 | 3567149 | 3567424 | False | COG3677 | 3e-21 | 71.0 | 26 | 117 |
| CP000305\_YPN\_3168 | 3568381 | 3570534 | False | - | - | - | - | - |
| CP000305\_YPN\_3169 | 3570618 | 3572966 | False | COG3501 | 9e-110 | 99.0 | 1 | 549 |
| CP000305\_YPN\_3169 | 3570618 | 3572966 | False | COG4253 | 6e-66 | 82.0 | 2 | 229 |
| CP000305\_YPN\_3170 | 3572963 | 3575611 | False | COG0542 | 0.0 | 100.0 | 1 | 786 |
| CP000305\_YPN\_3171 | 3576029 | 3576520 | False | COG3157 | 2e-40 | 98.0 | 1 | 160 |
| CP000305\_YPN\_3172 | 3576600 | 3578261 | False | COG2885 | 8e-17 | 88.0 | 12 | 179 |
| CP000305\_YPN\_3173 | 3578261 | 3578947 | False | COG3455 | 2e-48 | 91.0 | 21 | 260 |
| CP000305\_YPN\_3174 | 3578944 | 3580296 | False | COG3522 | 6e-133 | 99.0 | 2 | 446 |
| CP000305\_YPN\_3175 | 3580308 | 3581846 | False | COG3517 | 0.0 | 100.0 | 1 | 495 |
| CP000305\_YPN\_3176 | 3581895 | 3582395 | False | COG3516 | 6e-49 | 99.0 | 2 | 169 |
| CP000305\_YPN\_3177 | 3583795 | 3584166 | False | - | - | - | - | - |
| CP000305\_YPN\_3178 | 3584340 | 3584603 | False | COG3677 | 2e-18 | 68.0 | 26 | 114 |
| CP000305\_YPN\_3179 | 3584671 | 3585000 | False | - | - | - | - | - |
| CP000305\_YPN\_3180 | 3585279 | 3585374 | False | - | - | - | - | - |
| CP000305\_YPN\_3181 | 3585517 | 3586167 | False | - | - | - | - | - |
| CP000305\_YPN\_3182 | 3587337 | 3587987 | True | COG3916 | 3e-64 | 100.0 | 1 | 209 |
