# Supplementary material for: Dissecting the bacterial type VI secretion system by a genome wide in silico analysis: what can be learned from available microbial genomic resources?
Source: BMC Genomics. 2009 Mar 12;10:104. doi: 10.1186/1471-2164-10-104 (PMC2660368; doi:10.1186/1471-2164-10-104)
Supplement: Additional file 7 — Detailed description of all identified T6SS gene clusters. Archive containing the detailed description of each identified T6SS locus as an HTML file. [file 1471-2164-10-104-S7.tgz › LociHTML/HTML/CP000305H.html]

Locus CP000305H on Yersinia pestis (biovar Antiqua Nepal516, strain Nepal516) chromosome, complete sequence.

import namespace="svg" implementation="#AdobeSVG"?


# Locus CP000305H

# List of CDS in T6SS locus CP000305H

|  |  |  |  |  |  |  |  |  |
| --- | --- | --- | --- | --- | --- | --- | --- | --- |
| Name | from | to | direct | COG | e-value | COG cover | COG hit start | COG hit end |
| CP000305\_YPN\_3555 | 4056539 | 4061077 | False | COG3209 | 3e-63 | 99.0 | 1 | 795 |
| CP000305\_YPN\_3555 | 4056539 | 4061077 | False | COG4104 | 4e-10 | 73.0 | 25 | 96 |
| CP000305\_YPN\_3556 | 4061122 | 4061544 | False | COG5435 | 6e-45 | 97.0 | 3 | 145 |
| CP000305\_YPN\_3557 | 4061547 | 4063745 | False | COG3501 | 0.0 | 99.0 | 1 | 549 |
| CP000305\_YPN\_3558 | 4063767 | 4063943 | False | - | - | - | - | - |
| CP000305\_YPN\_3559 | 4064106 | 4064513 | True | - | - | - | - | - |
| CP000305\_YPN\_3560 | 4064595 | 4064819 | True | - | - | - | - | - |
| CP000305\_YPN\_3561 | 4064895 | 4065086 | True | - | - | - | - | - |
| CP000305\_YPN\_3562 | 4065136 | 4065621 | False | - | - | - | - | - |
| CP000305\_YPN\_3563 | 4065623 | 4066993 | False | COG3209 | 8e-32 | 59.0 | 326 | 795 |
| CP000305\_YPN\_3564 | 4067021 | 4069885 | False | COG3209 | 1e-61 | 99.0 | 2 | 794 |
| CP000305\_YPN\_3565 | 4069878 | 4070336 | False | COG5435 | 5e-48 | 100.0 | 1 | 147 |
| CP000305\_YPN\_3566 | 4070342 | 4072744 | False | COG3501 | 0.0 | 99.0 | 1 | 547 |
| CP000305\_YPN\_3567 | 4072766 | 4073389 | False | COG3515 | 3e-20 | 51.0 | 10 | 188 |
| CP000305\_YPN\_3568 | 4073296 | 4074078 | False | COG3515 | 3e-44 | 78.0 | 12 | 284 |
| CP000305\_YPN\_3569 | 4074203 | 4077736 | False | COG3523 | 0.0 | 100.0 | 1 | 1188 |
| CP000305\_YPN\_3570 | 4077768 | 4079156 | False | COG3515 | 2e-36 | 82.0 | 1 | 285 |
| CP000305\_YPN\_3571 | 4079162 | 4079848 | False | - | - | - | - | - |
| CP000305\_YPN\_3572 | 4079845 | 4080642 | False | - | - | - | - | - |
| CP000305\_YPN\_3573 | 4080639 | 4083242 | False | COG0542 | 0.0 | 99.0 | 1 | 784 |
| CP000305\_YPN\_3574 | 4083253 | 4084020 | False | COG3455 | 2e-86 | 98.0 | 4 | 260 |
| CP000305\_YPN\_3575 | 4084020 | 4085366 | False | COG3522 | 1e-167 | 100.0 | 1 | 446 |
| CP000305\_YPN\_3576 | 4085369 | 4085914 | False | COG3521 | 2e-39 | 100.0 | 1 | 159 |
| CP000305\_YPN\_3577 | 4085914 | 4087230 | False | COG3456 | 9e-124 | 100.0 | 1 | 430 |
| CP000305\_YPN\_3578 | 4087356 | 4088444 | False | COG3520 | 7e-106 | 99.0 | 1 | 332 |
| CP000305\_YPN\_3579 | 4088408 | 4089151 | False | COG3519 | 1e-59 | 39.0 | 379 | 621 |
| CP000305\_YPN\_3580 | 4089070 | 4089717 | True | COG2963 | 1e-12 | 95.0 | 6 | 116 |
| CP000305\_YPN\_3581 | 4089771 | 4090556 | True | COG2801 | 1e-17 | 92.0 | 16 | 230 |
| CP000305\_YPN\_3582 | 4090911 | 4091342 | False | - | - | - | - | - |
| CP000305\_YPN\_3583 | 4091345 | 4091809 | False | - | - | - | - | - |
| CP000305\_YPN\_3584 | 4092118 | 4092492 | False | - | - | - | - | - |
| CP000305\_YPN\_3585 | 4092891 | 4094405 | True | COG0578 | 2e-171 | 95.0 | 10 | 518 |
