# Supplementary material for: Dissecting the bacterial type VI secretion system by a genome wide in silico analysis: what can be learned from available microbial genomic resources?
Source: BMC Genomics. 2009 Mar 12;10:104. doi: 10.1186/1471-2164-10-104 (PMC2660368; doi:10.1186/1471-2164-10-104)
Supplement: Additional file 7 — Detailed description of all identified T6SS gene clusters. Archive containing the detailed description of each identified T6SS locus as an HTML file. [file 1471-2164-10-104-S7.tgz › LociHTML/HTML/CP000308A.html]

Locus CP000308A on Yersinia pestis (biovar Antiqua Antiqua, strain Antiqua) chromosome, complete sequence.

import namespace="svg" implementation="#AdobeSVG"?


# Locus CP000308A

# List of CDS in T6SS locus CP000308A

|  |  |  |  |  |  |  |  |  |
| --- | --- | --- | --- | --- | --- | --- | --- | --- |
| Name | from | to | direct | COG | e-value | COG cover | COG hit start | COG hit end |
| CP000308\_YPA\_0031 | 47393 | 47629 | True | - | - | - | - | - |
| CP000308\_YPA\_0032 | 47633 | 48742 | True | COG3839 | 9e-125 | 100.0 | 1 | 338 |
| CP000308\_YPA\_0033 | 48813 | 50084 | True | COG4580 | 4e-150 | 99.0 | 2 | 429 |
| CP000308\_YPA\_0034 | 50325 | 51236 | True | - | - | - | - | - |
| CP000308\_YPA\_0035 | 51575 | 51985 | True | - | - | - | - | - |
| CP000308\_YPA\_0036 | 52137 | 52655 | False | COG3157 | 1e-50 | 98.0 | 1 | 160 |
| CP000308\_YPA\_0037 | 53179 | 53676 | True | COG3516 | 1e-49 | 98.0 | 2 | 167 |
| CP000308\_YPA\_0038 | 53744 | 55225 | True | COG3517 | 0.0 | 99.0 | 1 | 493 |
| CP000308\_YPA\_0039 | 55232 | 55672 | True | COG3518 | 9e-35 | 100.0 | 1 | 157 |
| CP000308\_YPA\_0040 | 55672 | 56898 | True | COG3519 | 6e-95 | 59.0 | 3 | 374 |
| CP000308\_YPA\_0041 | 56788 | 57573 | False | COG2801 | 1e-17 | 92.0 | 16 | 230 |
| CP000308\_YPA\_0042 | 57627 | 58163 | False | COG2963 | 5e-12 | 95.0 | 6 | 116 |
| CP000308\_YPA\_0043 | 58229 | 58498 | False | COG0184 | 3e-28 | 100.0 | 1 | 89 |
| CP000308\_YPA\_0044 | 58620 | 59594 | False | COG0130 | 7e-74 | 98.0 | 6 | 271 |
| CP000308\_YPA\_0045 | 59594 | 60004 | False | COG0858 | 1e-29 | 99.0 | 1 | 117 |
| CP000308\_YPA\_0046 | 60070 | 62724 | False | COG0532 | 0.0 | 99.0 | 2 | 508 |
| CP000308\_YPA\_0046 | 60070 | 62724 | False | COG3064 | 2e-08 | 49.0 | 55 | 244 |
