# Supplementary material for: Dissecting the bacterial type VI secretion system by a genome wide in silico analysis: what can be learned from available microbial genomic resources?
Source: BMC Genomics. 2009 Mar 12;10:104. doi: 10.1186/1471-2164-10-104 (PMC2660368; doi:10.1186/1471-2164-10-104)
Supplement: Additional file 7 — Detailed description of all identified T6SS gene clusters. Archive containing the detailed description of each identified T6SS locus as an HTML file. [file 1471-2164-10-104-S7.tgz › LociHTML/HTML/CP000308B.html]

Locus CP000308B on Yersinia pestis (biovar Antiqua Antiqua, strain Antiqua) chromosome, complete sequence.

import namespace="svg" implementation="#AdobeSVG"?


# Locus CP000308B

# List of CDS in T6SS locus CP000308B

|  |  |  |  |  |  |  |  |  |
| --- | --- | --- | --- | --- | --- | --- | --- | --- |
| Name | from | to | direct | COG | e-value | COG cover | COG hit start | COG hit end |
| CP000308\_YPA\_0280 | 326133 | 326783 | False | COG3916 | 3e-64 | 100.0 | 1 | 209 |
| CP000308\_YPA\_0281 | 327958 | 328608 | True | - | - | - | - | - |
| CP000308\_YPA\_0282 | 329059 | 329454 | True | - | - | - | - | - |
| CP000308\_YPA\_0283 | 329522 | 329785 | True | COG3677 | 2e-18 | 68.0 | 26 | 114 |
| CP000308\_YPA\_0284 | 331730 | 332230 | True | COG3516 | 6e-49 | 99.0 | 2 | 169 |
| CP000308\_YPA\_0285 | 332279 | 333817 | True | COG3517 | 0.0 | 100.0 | 1 | 495 |
| CP000308\_YPA\_0286 | 333829 | 335181 | True | COG3522 | 6e-133 | 99.0 | 2 | 446 |
| CP000308\_YPA\_0287 | 335178 | 335864 | True | COG3455 | 2e-48 | 91.0 | 21 | 260 |
| CP000308\_YPA\_0288 | 335864 | 337600 | True | COG2885 | 8e-27 | 94.0 | 12 | 190 |
| CP000308\_YPA\_0289 | 337604 | 338095 | True | COG3157 | 2e-40 | 98.0 | 1 | 160 |
| CP000308\_YPA\_0290 | 338513 | 341161 | True | COG0542 | 0.0 | 100.0 | 1 | 786 |
| CP000308\_YPA\_0291 | 341158 | 343506 | True | COG3501 | 9e-110 | 99.0 | 1 | 549 |
| CP000308\_YPA\_0291 | 341158 | 343506 | True | COG4253 | 6e-66 | 82.0 | 2 | 229 |
| CP000308\_YPA\_0292 | 343590 | 345743 | True | - | - | - | - | - |
| CP000308\_YPA\_0293 | 346640 | 346915 | True | COG3677 | 3e-21 | 71.0 | 26 | 117 |
| CP000308\_YPA\_0295 | 346971 | 347228 | True | COG1662 | 2e-09 | 39.0 | 41 | 88 |
| CP000308\_YPA\_0296 | 347836 | 348471 | True | COG4253 | 3e-62 | 81.0 | 4 | 229 |
| CP000308\_YPA\_0297 | 348487 | 350784 | True | COG3179 | 6e-09 | 98.0 | 4 | 206 |
