# Supplementary material for: Dissecting the bacterial type VI secretion system by a genome wide in silico analysis: what can be learned from available microbial genomic resources?
Source: BMC Genomics. 2009 Mar 12;10:104. doi: 10.1186/1471-2164-10-104 (PMC2660368; doi:10.1186/1471-2164-10-104)
Supplement: Additional file 7 — Detailed description of all identified T6SS gene clusters. Archive containing the detailed description of each identified T6SS locus as an HTML file. [file 1471-2164-10-104-S7.tgz › LociHTML/HTML/CP000308C.html]

Locus CP000308C on Yersinia pestis (biovar Antiqua Antiqua, strain Antiqua) chromosome, complete sequence.

import namespace="svg" implementation="#AdobeSVG"?


# Locus CP000308C

# List of CDS in T6SS locus CP000308C

|  |  |  |  |  |  |  |  |  |
| --- | --- | --- | --- | --- | --- | --- | --- | --- |
| Name | from | to | direct | COG | e-value | COG cover | COG hit start | COG hit end |
| CP000308\_YPA\_0753 | 864762 | 865550 | True | COG1028 | 4e-25 | 99.0 | 2 | 250 |
| CP000308\_YPA\_0754 | 865616 | 866077 | True | - | - | - | - | - |
| CP000308\_YPA\_0755 | 866115 | 866363 | True | - | - | - | - | - |
| CP000308\_YPA\_0756 | 866462 | 867310 | True | COG0331 | 2e-74 | 93.0 | 2 | 290 |
| CP000308\_YPA\_0757 | 868022 | 869044 | True | COG4584 | 2e-58 | 100.0 | 1 | 278 |
| CP000308\_YPA\_0758 | 869044 | 869823 | True | COG1484 | 5e-64 | 99.0 | 2 | 254 |
| CP000308\_YPA\_0759 | 870258 | 870758 | True | COG3516 | 2e-48 | 99.0 | 2 | 169 |
| CP000308\_YPA\_0760 | 870807 | 872351 | True | COG3517 | 0.0 | 100.0 | 1 | 495 |
| CP000308\_YPA\_0761 | 872363 | 873715 | True | COG3522 | 4e-132 | 99.0 | 2 | 446 |
| CP000308\_YPA\_0762 | 873712 | 874398 | True | COG3455 | 2e-48 | 91.0 | 21 | 260 |
| CP000308\_YPA\_0763 | 874398 | 876134 | True | COG2885 | 5e-27 | 94.0 | 12 | 190 |
| CP000308\_YPA\_0764 | 876138 | 876629 | True | COG3157 | 2e-40 | 98.0 | 1 | 160 |
| CP000308\_YPA\_0765 | 877017 | 879659 | True | COG0542 | 0.0 | 99.0 | 1 | 784 |
| CP000308\_YPA\_0766 | 879662 | 882010 | True | COG3501 | 1e-105 | 99.0 | 1 | 549 |
| CP000308\_YPA\_0766 | 879662 | 882010 | True | COG4253 | 6e-67 | 82.0 | 2 | 229 |
| CP000308\_YPA\_0767 | 882095 | 884326 | True | - | - | - | - | - |
| CP000308\_YPA\_0768 | 884323 | 885096 | True | - | - | - | - | - |
| CP000308\_YPA\_0769 | 885250 | 885510 | True | COG4253 | 2e-25 | 30.0 | 144 | 229 |
| CP000308\_YPA\_0770 | 885526 | 887709 | True | - | - | - | - | - |
| CP000308\_YPA\_0771 | 887882 | 888352 | True | - | - | - | - | - |
| CP000308\_YPA\_0772 | 888817 | 888954 | False | - | - | - | - | - |
| CP000308\_YPA\_0773 | 889085 | 889405 | False | - | - | - | - | - |
| CP000308\_YPA\_0774 | 889514 | 889687 | False | - | - | - | - | - |
| CP000308\_YPA\_0775 | 890517 | 891749 | True | - | - | - | - | - |
| CP000308\_YPA\_0776 | 891746 | 895168 | True | COG3523 | 0.0 | 100.0 | 1 | 1188 |
| CP000308\_YPA\_0777 | 895212 | 896498 | True | COG3515 | 2e-42 | 100.0 | 1 | 346 |
| CP000308\_YPA\_0778 | 896550 | 896813 | True | - | - | - | - | - |
| CP000308\_YPA\_0779 | 896831 | 897889 | True | - | - | - | - | - |
| CP000308\_YPA\_0780 | 897904 | 898359 | True | - | - | - | - | - |
| CP000308\_YPA\_0781 | 898580 | 900343 | True | COG3519 | 0.0 | 100.0 | 1 | 621 |
| CP000308\_YPA\_0782 | 900307 | 901392 | True | COG3520 | 3e-85 | 97.0 | 1 | 328 |
| CP000308\_YPA\_0783 | 901268 | 901948 | True | COG3521 | 9e-36 | 98.0 | 1 | 157 |
| CP000308\_YPA\_0784 | 901948 | 902400 | True | COG3518 | 1e-27 | 98.0 | 3 | 157 |
| CP000308\_YPA\_0785 | 902425 | 903792 | True | COG3515 | 9e-41 | 96.0 | 13 | 346 |
| CP000308\_YPA\_0786 | 904004 | 904627 | False | - | - | - | - | - |
| CP000308\_YPA\_0787 | 905142 | 906905 | True | COG0488 | 8e-172 | 99.0 | 1 | 528 |
| CP000308\_YPA\_0788 | 907133 | 907786 | True | - | - | - | - | - |
| CP000308\_YPA\_0789 | 907830 | 909038 | False | COG3328 | 2e-112 | 98.0 | 1 | 375 |
