# Supplementary material for: Dissecting the bacterial type VI secretion system by a genome wide in silico analysis: what can be learned from available microbial genomic resources?
Source: BMC Genomics. 2009 Mar 12;10:104. doi: 10.1186/1471-2164-10-104 (PMC2660368; doi:10.1186/1471-2164-10-104)
Supplement: Additional file 7 — Detailed description of all identified T6SS gene clusters. Archive containing the detailed description of each identified T6SS locus as an HTML file. [file 1471-2164-10-104-S7.tgz › LociHTML/HTML/CP000308E.html]

Locus CP000308E on Yersinia pestis (biovar Antiqua Antiqua, strain Antiqua) chromosome, complete sequence.

import namespace="svg" implementation="#AdobeSVG"?


# Locus CP000308E

# List of CDS in T6SS locus CP000308E

|  |  |  |  |  |  |  |  |  |
| --- | --- | --- | --- | --- | --- | --- | --- | --- |
| Name | from | to | direct | COG | e-value | COG cover | COG hit start | COG hit end |
| CP000308\_YPA\_2111 | 2365311 | 2365778 | False | - | - | - | - | - |
| CP000308\_YPA\_2112 | 2366394 | 2366936 | True | COG1704 | 3e-55 | 99.0 | 2 | 185 |
| CP000308\_YPA\_2113 | 2366881 | 2368980 | True | COG4907 | 4e-08 | 30.0 | 412 | 594 |
| CP000308\_YPA\_2114 | 2369356 | 2369697 | True | COG2824 | 2e-46 | 100.0 | 1 | 112 |
| CP000308\_YPA\_2115 | 2369772 | 2370137 | False | - | - | - | - | - |
| CP000308\_YPA\_2116 | 2370144 | 2370623 | False | COG3518 | 3e-35 | 99.0 | 1 | 156 |
| CP000308\_YPA\_2117 | 2370720 | 2371526 | False | COG4455 | 9e-108 | 100.0 | 1 | 273 |
| CP000308\_YPA\_2118 | 2371546 | 2372394 | False | - | - | - | - | - |
| CP000308\_YPA\_2119 | 2372394 | 2372654 | False | - | - | - | - | - |
| CP000308\_YPA\_2120 | 2372745 | 2375591 | False | COG3501 | 7e-150 | 97.0 | 6 | 539 |
| CP000308\_YPA\_2121 | 2375885 | 2379712 | False | COG3523 | 0.0 | 99.0 | 3 | 1185 |
| CP000308\_YPA\_2122 | 2379721 | 2380557 | False | COG1360 | 4e-32 | 59.0 | 95 | 240 |
| CP000308\_YPA\_2122 | 2379721 | 2380557 | False | COG3455 | 6e-30 | 42.0 | 151 | 262 |
| CP000308\_YPA\_2123 | 2380573 | 2381352 | False | COG1484 | 5e-64 | 99.0 | 2 | 254 |
| CP000308\_YPA\_2124 | 2381352 | 2382374 | False | COG4584 | 2e-58 | 100.0 | 1 | 278 |
| CP000308\_YPA\_2125 | 2382388 | 2383056 | False | COG3455 | 7e-18 | 42.0 | 40 | 151 |
| CP000308\_YPA\_2126 | 2383053 | 2384402 | False | COG3522 | 3e-158 | 99.0 | 2 | 446 |
| CP000308\_YPA\_2127 | 2384406 | 2384966 | False | COG3521 | 4e-39 | 100.0 | 1 | 159 |
| CP000308\_YPA\_2128 | 2385219 | 2385788 | False | COG3157 | 4e-42 | 100.0 | 1 | 162 |
| CP000308\_YPA\_2129 | 2386030 | 2387532 | False | COG3517 | 0.0 | 100.0 | 1 | 495 |
| CP000308\_YPA\_2130 | 2387556 | 2388080 | False | COG3516 | 8e-59 | 99.0 | 2 | 169 |
| CP000308\_YPA\_2131 | 2388185 | 2388793 | False | COG3539 | 4e-16 | 91.0 | 16 | 184 |
| CP000308\_YPA\_2132 | 2388778 | 2389968 | False | COG3188 | 2e-61 | 44.0 | 464 | 834 |
| CP000308\_YPA\_2133 | 2389993 | 2391201 | True | COG3328 | 2e-112 | 98.0 | 1 | 375 |
| CP000308\_YPA\_2134 | 2391223 | 2392773 | False | COG3188 | 1e-110 | 56.0 | 7 | 474 |
| CP000308\_YPA\_2135 | 2392901 | 2393662 | False | COG3121 | 3e-58 | 94.0 | 12 | 234 |
| CP000308\_YPA\_2136 | 2393819 | 2394364 | False | COG3539 | 1e-14 | 100.0 | 1 | 184 |
| CP000308\_YPA\_2137 | 2394565 | 2397240 | False | COG0542 | 0.0 | 98.0 | 1 | 777 |
| CP000308\_YPA\_2138 | 2398023 | 2399903 | True | COG3519 | 0.0 | 100.0 | 1 | 621 |
| CP000308\_YPA\_2139 | 2399903 | 2400928 | True | COG3520 | 1e-97 | 100.0 | 1 | 335 |
| CP000308\_YPA\_2140 | 2401027 | 2402208 | True | COG3515 | 1e-49 | 98.0 | 1 | 341 |
| CP000308\_YPA\_2141 | 2402215 | 2403246 | True | - | - | - | - | - |
| CP000308\_YPA\_2142 | 2403286 | 2404623 | True | - | - | - | - | - |
| CP000308\_YPA\_2143 | 2404569 | 2405303 | True | - | - | - | - | - |
| CP000308\_YPA\_2144 | 2405304 | 2406986 | True | COG2885 | 1e-27 | 55.0 | 86 | 190 |
| CP000308\_YPA\_2145 | 2406983 | 2407465 | True | COG5435 | 7e-43 | 100.0 | 1 | 147 |
