# Supplementary material for: Dissecting the bacterial type VI secretion system by a genome wide in silico analysis: what can be learned from available microbial genomic resources?
Source: BMC Genomics. 2009 Mar 12;10:104. doi: 10.1186/1471-2164-10-104 (PMC2660368; doi:10.1186/1471-2164-10-104)
Supplement: Additional file 7 — Detailed description of all identified T6SS gene clusters. Archive containing the detailed description of each identified T6SS locus as an HTML file. [file 1471-2164-10-104-S7.tgz › LociHTML/HTML/CP000308F.html]

Locus CP000308F on Yersinia pestis (biovar Antiqua Antiqua, strain Antiqua) chromosome, complete sequence.

import namespace="svg" implementation="#AdobeSVG"?


# Locus CP000308F

# List of CDS in T6SS locus CP000308F

|  |  |  |  |  |  |  |  |  |
| --- | --- | --- | --- | --- | --- | --- | --- | --- |
| Name | from | to | direct | COG | e-value | COG cover | COG hit start | COG hit end |
| CP000308\_YPA\_3391 | 3782417 | 3783787 | False | COG0471 | 1e-54 | 98.0 | 4 | 456 |
| CP000308\_YPA\_3392 | 3784069 | 3785088 | False | COG1609 | 1e-68 | 99.0 | 1 | 332 |
| CP000308\_YPA\_3393 | 3785370 | 3786545 | True | - | - | - | - | - |
| CP000308\_YPA\_3394 | 3786435 | 3787220 | False | COG2801 | 1e-17 | 92.0 | 16 | 230 |
| CP000308\_YPA\_3395 | 3787274 | 3787921 | False | COG2963 | 1e-12 | 95.0 | 6 | 116 |
| CP000308\_YPA\_3396 | 3787840 | 3788583 | True | COG3519 | 1e-59 | 39.0 | 379 | 621 |
| CP000308\_YPA\_3397 | 3788547 | 3789635 | True | COG3520 | 7e-106 | 99.0 | 1 | 332 |
| CP000308\_YPA\_3398 | 3789761 | 3791077 | True | COG3456 | 9e-124 | 100.0 | 1 | 430 |
| CP000308\_YPA\_3399 | 3791077 | 3791622 | True | COG3521 | 2e-39 | 100.0 | 1 | 159 |
| CP000308\_YPA\_3400 | 3791625 | 3792971 | True | COG3522 | 1e-167 | 100.0 | 1 | 446 |
| CP000308\_YPA\_3401 | 3792971 | 3793738 | True | COG3455 | 2e-86 | 98.0 | 4 | 260 |
| CP000308\_YPA\_3402 | 3793749 | 3796352 | True | COG0542 | 0.0 | 99.0 | 1 | 784 |
| CP000308\_YPA\_3403 | 3796349 | 3797146 | True | - | - | - | - | - |
| CP000308\_YPA\_3404 | 3797143 | 3797829 | True | - | - | - | - | - |
| CP000308\_YPA\_3405 | 3797835 | 3799223 | True | COG3515 | 2e-36 | 82.0 | 1 | 285 |
| CP000308\_YPA\_3406 | 3799255 | 3802788 | True | COG3523 | 0.0 | 100.0 | 1 | 1188 |
| CP000308\_YPA\_3407 | 3802913 | 3803695 | True | COG3515 | 3e-44 | 78.0 | 12 | 284 |
| CP000308\_YPA\_3408 | 3803602 | 3804225 | True | COG3515 | 3e-20 | 51.0 | 10 | 188 |
| CP000308\_YPA\_3409 | 3804247 | 3806649 | True | COG3501 | 0.0 | 99.0 | 1 | 547 |
| CP000308\_YPA\_3410 | 3806655 | 3807113 | True | COG5435 | 5e-48 | 100.0 | 1 | 147 |
| CP000308\_YPA\_3411 | 3807106 | 3809970 | True | COG3209 | 1e-61 | 99.0 | 2 | 794 |
| CP000308\_YPA\_3412 | 3809998 | 3811368 | True | COG3209 | 8e-32 | 59.0 | 326 | 795 |
| CP000308\_YPA\_3413 | 3811370 | 3811855 | True | - | - | - | - | - |
| CP000308\_YPA\_3414 | 3811905 | 3812096 | False | - | - | - | - | - |
| CP000308\_YPA\_3415 | 3812172 | 3812396 | False | - | - | - | - | - |
| CP000308\_YPA\_3416 | 3812478 | 3812885 | False | - | - | - | - | - |
| CP000308\_YPA\_3417 | 3813048 | 3813224 | True | - | - | - | - | - |
| CP000308\_YPA\_3418 | 3813246 | 3815444 | True | COG3501 | 0.0 | 99.0 | 1 | 549 |
| CP000308\_YPA\_3419 | 3815447 | 3815869 | True | COG5435 | 6e-45 | 97.0 | 3 | 145 |
| CP000308\_YPA\_3420 | 3815914 | 3820452 | True | COG3209 | 3e-63 | 99.0 | 1 | 795 |
| CP000308\_YPA\_3420 | 3815914 | 3820452 | True | COG4104 | 4e-10 | 73.0 | 25 | 96 |
