# Supplementary material for: Dissecting the bacterial type VI secretion system by a genome wide in silico analysis: what can be learned from available microbial genomic resources?
Source: BMC Genomics. 2009 Mar 12;10:104. doi: 10.1186/1471-2164-10-104 (PMC2660368; doi:10.1186/1471-2164-10-104)
Supplement: Additional file 7 — Detailed description of all identified T6SS gene clusters. Archive containing the detailed description of each identified T6SS locus as an HTML file. [file 1471-2164-10-104-S7.tgz › LociHTML/HTML/CP000308G.html]

Locus CP000308G on Yersinia pestis (biovar Antiqua Antiqua, strain Antiqua) chromosome, complete sequence.

import namespace="svg" implementation="#AdobeSVG"?


# Locus CP000308G

# List of CDS in T6SS locus CP000308G

|  |  |  |  |  |  |  |  |  |
| --- | --- | --- | --- | --- | --- | --- | --- | --- |
| Name | from | to | direct | COG | e-value | COG cover | COG hit start | COG hit end |
| CP000308\_YPA\_3577 | 4000494 | 4002863 | True | COG0417 | 0.0 | 98.0 | 5 | 788 |
| CP000308\_YPA\_3578 | 4003308 | 4006214 | True | COG0553 | 2e-72 | 98.0 | 8 | 861 |
| CP000308\_YPA\_3579 | 4006470 | 4006823 | False | - | - | - | - | - |
| CP000308\_YPA\_3580 | 4006845 | 4010339 | False | COG3523 | 0.0 | 98.0 | 12 | 1187 |
| CP000308\_YPA\_3581 | 4010348 | 4011958 | False | COG3455 | 1e-72 | 100.0 | 1 | 262 |
| CP000308\_YPA\_3581 | 4010348 | 4011958 | False | COG1360 | 2e-27 | 56.0 | 108 | 244 |
| CP000308\_YPA\_3582 | 4011955 | 4013310 | False | COG3522 | 2e-141 | 100.0 | 1 | 446 |
| CP000308\_YPA\_3583 | 4013430 | 4013921 | False | COG3521 | 1e-34 | 98.0 | 3 | 158 |
| CP000308\_YPA\_3584 | 4013914 | 4014279 | False | - | - | - | - | - |
| CP000308\_YPA\_3585 | 4014285 | 4014902 | False | - | - | - | - | - |
| CP000308\_YPA\_3586 | 4014895 | 4015998 | False | COG1357 | 1e-18 | 99.0 | 3 | 238 |
| CP000308\_YPA\_3587 | 4016024 | 4017613 | False | COG1357 | 1e-13 | 83.0 | 18 | 215 |
| CP000308\_YPA\_3588 | 4017671 | 4018243 | False | - | - | - | - | - |
| CP000308\_YPA\_3589 | 4018256 | 4020595 | False | COG3501 | 2e-151 | 95.0 | 10 | 533 |
| CP000308\_YPA\_3590 | 4020699 | 4023287 | False | COG0542 | 0.0 | 99.0 | 1 | 783 |
| CP000308\_YPA\_3591 | 4023305 | 4024288 | False | COG3520 | 2e-83 | 98.0 | 4 | 334 |
| CP000308\_YPA\_3592 | 4024281 | 4026125 | False | COG3519 | 0.0 | 99.0 | 1 | 617 |
| CP000308\_YPA\_3593 | 4026158 | 4026601 | False | COG3518 | 8e-31 | 95.0 | 4 | 153 |
| CP000308\_YPA\_3594 | 4026675 | 4027193 | False | COG3157 | 7e-36 | 100.0 | 1 | 162 |
| CP000308\_YPA\_3595 | 4027356 | 4028261 | False | COG3517 | 1e-138 | 57.0 | 210 | 495 |
| CP000308\_YPA\_3596 | 4028318 | 4029340 | True | COG4584 | 2e-58 | 100.0 | 1 | 278 |
| CP000308\_YPA\_3597 | 4029340 | 4030119 | True | COG1484 | 5e-64 | 99.0 | 2 | 254 |
| CP000308\_YPA\_3598 | 4030129 | 4030419 | False | - | - | - | - | - |
| CP000308\_YPA\_3599 | 4030784 | 4031539 | False | COG2801 | 4e-13 | 88.0 | 27 | 232 |
| CP000308\_YPA\_3600 | 4031617 | 4031883 | False | - | - | - | - | - |
