# Supplementary material for: Dissecting the bacterial type VI secretion system by a genome wide in silico analysis: what can be learned from available microbial genomic resources?
Source: BMC Genomics. 2009 Mar 12;10:104. doi: 10.1186/1471-2164-10-104 (PMC2660368; doi:10.1186/1471-2164-10-104)
Supplement: Additional file 7 — Detailed description of all identified T6SS gene clusters. Archive containing the detailed description of each identified T6SS locus as an HTML file. [file 1471-2164-10-104-S7.tgz › LociHTML/HTML/CP000352A.html]

Locus CP000352A on Ralstonia metallidurans (strain CH34 / ATCC 43123 / DSM 2839) chromosome, complete sequence.

import namespace="svg" implementation="#AdobeSVG"?


# Locus CP000352A

# List of CDS in T6SS locus CP000352A

|  |  |  |  |  |  |  |  |  |
| --- | --- | --- | --- | --- | --- | --- | --- | --- |
| Name | from | to | direct | COG | e-value | COG cover | COG hit start | COG hit end |
| CP000352\_Rmet\_0614 | 668983 | 674208 | False | COG3468 | 9e-10 | 100.0 | 1 | 592 |
| CP000352\_Rmet\_0615 | 674569 | 674859 | True | COG0234 | 9e-33 | 100.0 | 1 | 96 |
| CP000352\_Rmet\_0616 | 674944 | 676587 | True | COG0459 | 1e-139 | 100.0 | 1 | 524 |
| CP000352\_Rmet\_0617 | 676887 | 677321 | False | COG3518 | 3e-11 | 63.0 | 38 | 137 |
| CP000352\_Rmet\_0618 | 677318 | 679999 | False | COG0542 | 0.0 | 97.0 | 1 | 764 |
| CP000352\_Rmet\_0619 | 680084 | 680569 | False | COG3157 | 1e-31 | 96.0 | 1 | 157 |
| CP000352\_Rmet\_0620 | 680584 | 682302 | False | COG2885 | 1e-22 | 92.0 | 16 | 190 |
| CP000352\_Rmet\_0621 | 682313 | 682969 | False | COG3455 | 4e-16 | 87.0 | 28 | 255 |
| CP000352\_Rmet\_0622 | 682966 | 683796 | False | COG3522 | 6e-57 | 60.0 | 176 | 444 |
| CP000352\_Rmet\_0623 | 683888 | 684322 | True | - | - | - | - | - |
| CP000352\_Rmet\_0624 | 684362 | 685939 | False | COG3517 | 0.0 | 99.0 | 1 | 494 |
| CP000352\_Rmet\_0625 | 685965 | 686480 | False | COG3516 | 3e-41 | 97.0 | 4 | 167 |
| CP000352\_Rmet\_0626 | 686770 | 689439 | True | COG4253 | 4e-44 | 100.0 | 1 | 278 |
| CP000352\_Rmet\_0626 | 686770 | 689439 | True | COG3501 | 9e-57 | 95.0 | 10 | 535 |
| CP000352\_Rmet\_0627 | 689436 | 691403 | True | COG1502 | 4e-10 | 33.0 | 165 | 312 |
| CP000352\_Rmet\_0627 | 689436 | 691403 | True | COG1502 | 3e-10 | 17.0 | 352 | 429 |
| CP000352\_Rmet\_0628 | 691407 | 692714 | True | COG0790 | 7e-09 | 45.0 | 109 | 241 |
| CP000352\_Rmet\_0629 | 692711 | 692911 | True | - | - | - | - | - |
| CP000352\_Rmet\_0630 | 692915 | 694213 | True | COG0790 | 5e-09 | 45.0 | 109 | 241 |
| CP000352\_Rmet\_0631 | 694310 | 694564 | True | COG4104 | 4e-14 | 81.0 | 11 | 90 |
| CP000352\_Rmet\_0632 | 694531 | 695808 | True | - | - | - | - | - |
| CP000352\_Rmet\_0633 | 695874 | 699221 | True | COG3523 | 3e-72 | 83.0 | 197 | 1188 |
| CP000352\_Rmet\_0634 | 699369 | 700940 | True | COG3515 | 2e-09 | 73.0 | 15 | 269 |
| CP000352\_Rmet\_0635 | 700962 | 702782 | True | COG3519 | 3e-132 | 99.0 | 2 | 617 |
| CP000352\_Rmet\_0636 | 702800 | 703888 | True | COG3520 | 6e-47 | 93.0 | 4 | 317 |
| CP000352\_Rmet\_0637 | 703925 | 704494 | True | COG3521 | 1e-17 | 77.0 | 10 | 132 |
| CP000352\_Rmet\_0638 | 704628 | 705416 | False | - | - | - | - | - |
| CP000352\_Rmet\_0639 | 705624 | 706595 | False | COG2064 | 1e-21 | 73.0 | 81 | 314 |
| CP000352\_Rmet\_0640 | 706647 | 707624 | False | COG4965 | 1e-51 | 99.0 | 2 | 309 |
| CP000352\_Rmet\_0641 | 707667 | 709037 | False | COG4962 | 2e-120 | 98.0 | 7 | 355 |
| CP000352\_Rmet\_0642 | 709100 | 710293 | False | COG4963 | 7e-43 | 91.0 | 31 | 366 |
