# Supplementary material for: Dissecting the bacterial type VI secretion system by a genome wide in silico analysis: what can be learned from available microbial genomic resources?
Source: BMC Genomics. 2009 Mar 12;10:104. doi: 10.1186/1471-2164-10-104 (PMC2660368; doi:10.1186/1471-2164-10-104)
Supplement: Additional file 7 — Detailed description of all identified T6SS gene clusters. Archive containing the detailed description of each identified T6SS locus as an HTML file. [file 1471-2164-10-104-S7.tgz › LociHTML/HTML/CP000378B.html]

Locus CP000378B on Burkholderia cenocepacia (strain AU 1054) chromosome 1, complete sequence.

import namespace="svg" implementation="#AdobeSVG"?


# Locus CP000378B

# List of CDS in T6SS locus CP000378B

|  |  |  |  |  |  |  |  |  |
| --- | --- | --- | --- | --- | --- | --- | --- | --- |
| Name | from | to | direct | COG | e-value | COG cover | COG hit start | COG hit end |
| CP000378\_Bcen\_2614 | 2874630 | 2875550 | False | COG0583 | 2e-28 | 97.0 | 5 | 295 |
| CP000378\_Bcen\_2615 | 2875702 | 2876691 | True | COG0604 | 2e-43 | 100.0 | 1 | 326 |
| CP000378\_Bcen\_2616 | 2876719 | 2876868 | True | COG4317 | 2e-08 | 50.0 | 4 | 50 |
| CP000378\_Bcen\_2617 | 2876912 | 2877940 | False | COG0598 | 2e-46 | 90.0 | 29 | 320 |
| CP000378\_Bcen\_2618 | 2878153 | 2879157 | False | - | - | - | - | - |
| CP000378\_Bcen\_2619 | 2879279 | 2880124 | True | - | - | - | - | - |
| CP000378\_Bcen\_2620 | 2880426 | 2884370 | True | COG3523 | 1e-121 | 47.0 | 5 | 573 |
| CP000378\_Bcen\_2620 | 2880426 | 2884370 | True | COG3523 | 4e-109 | 52.0 | 565 | 1188 |
| CP000378\_Bcen\_2621 | 2884367 | 2885356 | True | COG3913 | 1e-34 | 93.0 | 5 | 216 |
| CP000378\_Bcen\_2622 | 2885566 | 2886309 | True | COG2885 | 3e-27 | 84.0 | 27 | 187 |
| CP000378\_Bcen\_2623 | 2886577 | 2887197 | False | - | - | - | - | - |
| CP000378\_Bcen\_2624 | 2887230 | 2888099 | False | - | - | - | - | - |
| CP000378\_Bcen\_2625 | 2888276 | 2888533 | True | COG2885 | 3e-21 | 43.0 | 105 | 187 |
| CP000378\_Bcen\_2626 | 2888616 | 2890877 | False | - | - | - | - | - |
| CP000378\_Bcen\_2627 | 2890864 | 2891799 | False | - | - | - | - | - |
| CP000378\_Bcen\_2628 | 2892019 | 2892831 | False | - | - | - | - | - |
| CP000378\_Bcen\_2629 | 2892842 | 2893882 | False | - | - | - | - | - |
| CP000378\_Bcen\_2630 | 2893904 | 2896447 | False | COG3501 | 4e-103 | 96.0 | 1 | 532 |
| CP000378\_Bcen\_2631 | 2896514 | 2897635 | False | COG3515 | 1e-29 | 98.0 | 7 | 346 |
| CP000378\_Bcen\_2632 | 2897679 | 2899724 | False | COG0542 | 0.0 | 76.0 | 173 | 770 |
| CP000378\_Bcen\_2633 | 2899906 | 2900394 | True | - | - | - | - | - |
| CP000378\_Bcen\_2634 | 2900388 | 2901404 | False | COG3520 | 5e-58 | 90.0 | 32 | 335 |
| CP000378\_Bcen\_2635 | 2901452 | 2903287 | False | COG3519 | 4e-155 | 99.0 | 2 | 620 |
| CP000378\_Bcen\_2636 | 2903364 | 2903849 | False | COG3518 | 1e-33 | 96.0 | 4 | 155 |
| CP000378\_Bcen\_2637 | 2903912 | 2904415 | False | COG3157 | 4e-32 | 97.0 | 5 | 162 |
| CP000378\_Bcen\_2638 | 2904486 | 2905976 | False | COG3517 | 0.0 | 99.0 | 2 | 495 |
| CP000378\_Bcen\_2639 | 2905992 | 2906507 | False | COG3516 | 2e-48 | 98.0 | 2 | 168 |
| CP000378\_Bcen\_2640 | 2906554 | 2907189 | False | - | - | - | - | - |
| CP000378\_Bcen\_2641 | 2907562 | 2908173 | True | COG3521 | 8e-28 | 87.0 | 9 | 147 |
| CP000378\_Bcen\_2642 | 2908276 | 2909622 | True | COG3522 | 2e-111 | 100.0 | 1 | 446 |
| CP000378\_Bcen\_2643 | 2909619 | 2910401 | True | COG3455 | 3e-45 | 95.0 | 14 | 262 |
| CP000378\_Bcen\_2644 | 2910488 | 2910805 | False | - | - | - | - | - |
| CP000378\_Bcen\_2645 | 2911341 | 2911916 | False | - | - | - | - | - |
| CP000378\_Bcen\_2646 | 2911927 | 2913678 | False | COG3209 | 3e-25 | 56.0 | 214 | 665 |
| CP000378\_Bcen\_2647 | 2913671 | 2916475 | False | COG3209 | 2e-17 | 66.0 | 104 | 633 |
| CP000378\_Bcen\_2648 | 2916636 | 2919827 | False | COG4253 | 3e-46 | 93.0 | 1 | 260 |
| CP000378\_Bcen\_2648 | 2916636 | 2919827 | False | COG3501 | 2e-112 | 96.0 | 1 | 532 |
| CP000378\_Bcen\_2649 | 2920184 | 2920984 | True | COG0834 | 6e-45 | 99.0 | 1 | 273 |
| CP000378\_Bcen\_2650 | 2921094 | 2921297 | False | - | - | - | - | - |
| CP000378\_Bcen\_2651 | 2922322 | 2922966 | False | - | - | - | - | - |
| CP000378\_Bcen\_2652 | 2923030 | 2923914 | False | - | - | - | - | - |
| CP000378\_Bcen\_2653 | 2924028 | 2925644 | False | - | - | - | - | - |
