# Supplementary material for: Dissecting the bacterial type VI secretion system by a genome wide in silico analysis: what can be learned from available microbial genomic resources?
Source: BMC Genomics. 2009 Mar 12;10:104. doi: 10.1186/1471-2164-10-104 (PMC2660368; doi:10.1186/1471-2164-10-104)
Supplement: Additional file 7 — Detailed description of all identified T6SS gene clusters. Archive containing the detailed description of each identified T6SS locus as an HTML file. [file 1471-2164-10-104-S7.tgz › LociHTML/HTML/CP000438A.html]

Locus CP000438A on Pseudomonas aeruginosa (strain UCBPP-PA14) chromosome, complete sequence.

import namespace="svg" implementation="#AdobeSVG"?


# Locus CP000438A

# List of CDS in T6SS locus CP000438A

|  |  |  |  |  |  |  |  |  |
| --- | --- | --- | --- | --- | --- | --- | --- | --- |
| Name | from | to | direct | COG | e-value | COG cover | COG hit start | COG hit end |
| CP000438\_PA14\_00850 | 82730 | 83929 | False | COG4591 | 6e-10 | 99.0 | 3 | 408 |
| CP000438\_PA14\_00860 | 83929 | 84648 | False | COG1136 | 3e-51 | 92.0 | 1 | 209 |
| CP000438\_PA14\_00875 | 84645 | 87743 | False | COG0515 | 1e-47 | 96.0 | 1 | 369 |
| CP000438\_PA14\_00890 | 87751 | 88479 | False | COG0631 | 5e-56 | 95.0 | 1 | 250 |
| CP000438\_PA14\_00900 | 88489 | 89169 | False | COG3913 | 2e-77 | 98.0 | 5 | 227 |
| CP000438\_PA14\_00910 | 89166 | 92474 | False | COG3523 | 0.0 | 93.0 | 73 | 1187 |
| CP000438\_PA14\_00925 | 92696 | 94045 | False | COG3455 | 8e-80 | 100.0 | 1 | 262 |
| CP000438\_PA14\_00925 | 92696 | 94045 | False | COG1360 | 3e-32 | 58.0 | 103 | 244 |
| CP000438\_PA14\_00940 | 94052 | 95386 | False | COG3522 | 1e-163 | 100.0 | 1 | 446 |
| CP000438\_PA14\_00960 | 95402 | 95866 | False | COG3521 | 2e-48 | 96.0 | 6 | 158 |
| CP000438\_PA14\_00970 | 95911 | 96453 | False | COG3456 | 5e-49 | 40.0 | 255 | 430 |
| CP000438\_PA14\_00980 | 96450 | 97409 | False | COG3456 | 2e-47 | 58.0 | 1 | 253 |
| CP000438\_PA14\_00990 | 97777 | 98811 | True | COG3515 | 1e-43 | 99.0 | 1 | 345 |
| CP000438\_PA14\_01010 | 98900 | 99418 | True | COG3516 | 2e-58 | 99.0 | 2 | 169 |
| CP000438\_PA14\_01020 | 99431 | 100927 | True | COG3517 | 0.0 | 100.0 | 1 | 495 |
| CP000438\_PA14\_01030 | 101003 | 101491 | True | COG3157 | 5e-40 | 100.0 | 1 | 162 |
| CP000438\_PA14\_01040 | 101659 | 102504 | True | COG4455 | 3e-94 | 100.0 | 1 | 273 |
| CP000438\_PA14\_01060 | 102506 | 103015 | True | COG3518 | 3e-34 | 100.0 | 1 | 157 |
| CP000438\_PA14\_01070 | 103012 | 104871 | True | COG3519 | 0.0 | 100.0 | 1 | 621 |
| CP000438\_PA14\_01080 | 104835 | 105881 | True | COG3520 | 7e-102 | 99.0 | 2 | 335 |
| CP000438\_PA14\_01100 | 105874 | 108582 | True | COG0542 | 0.0 | 100.0 | 1 | 786 |
| CP000438\_PA14\_01110 | 108629 | 110560 | True | COG3501 | 0.0 | 98.0 | 7 | 549 |
| CP000438\_PA14\_01120 | 110675 | 110959 | False | - | - | - | - | - |
| CP000438\_PA14\_01130 | 110972 | 111205 | False | - | - | - | - | - |
| CP000438\_PA14\_01140 | 111195 | 112514 | False | - | - | - | - | - |
| CP000438\_PA14\_01150 | 112593 | 113027 | False | COG5435 | 1e-44 | 99.0 | 1 | 146 |
| CP000438\_PA14\_01160 | 113281 | 115506 | True | COG3501 | 0.0 | 99.0 | 4 | 550 |
| CP000438\_PA14\_01170 | 115534 | 115983 | True | - | - | - | - | - |
| CP000438\_PA14\_01180 | 115913 | 117112 | True | COG5351 | 1e-154 | 100.0 | 1 | 367 |
| CP000438\_PA14\_01190 | 117109 | 118146 | True | COG0304 | 2e-36 | 83.0 | 34 | 377 |
| CP000438\_PA14\_01200 | 118146 | 119237 | True | - | - | - | - | - |
| CP000438\_PA14\_01220 | 119248 | 120168 | True | - | - | - | - | - |
| CP000438\_PA14\_01230 | 120178 | 121410 | True | - | - | - | - | - |
