# Supplementary material for: Dissecting the bacterial type VI secretion system by a genome wide in silico analysis: what can be learned from available microbial genomic resources?
Source: BMC Genomics. 2009 Mar 12;10:104. doi: 10.1186/1471-2164-10-104 (PMC2660368; doi:10.1186/1471-2164-10-104)
Supplement: Additional file 7 — Detailed description of all identified T6SS gene clusters. Archive containing the detailed description of each identified T6SS locus as an HTML file. [file 1471-2164-10-104-S7.tgz › LociHTML/HTML/CP000438C.html]

Locus CP000438C on Pseudomonas aeruginosa (strain UCBPP-PA14) chromosome, complete sequence.

import namespace="svg" implementation="#AdobeSVG"?


# Locus CP000438C

# List of CDS in T6SS locus CP000438C

|  |  |  |  |  |  |  |  |  |
| --- | --- | --- | --- | --- | --- | --- | --- | --- |
| Name | from | to | direct | COG | e-value | COG cover | COG hit start | COG hit end |
| CP000438\_PA14\_33900 | 3011563 | 3013878 | True | COG1529 | 3e-99 | 99.0 | 3 | 730 |
| CP000438\_PA14\_33910 | 3014295 | 3015569 | False | COG1840 | 9e-27 | 95.0 | 1 | 286 |
| CP000438\_PA14\_33920 | 3015701 | 3016342 | False | COG2197 | 3e-39 | 98.0 | 2 | 208 |
| CP000438\_PA14\_33930 | 3016619 | 3017014 | True | - | - | - | - | - |
| CP000438\_PA14\_33940 | 3017037 | 3017573 | False | COG2849 | 1e-30 | 70.0 | 70 | 230 |
| CP000438\_PA14\_33960 | 3017584 | 3019590 | False | COG3501 | 9e-157 | 98.0 | 6 | 544 |
| CP000438\_PA14\_33970 | 3019627 | 3020694 | False | - | - | - | - | - |
| CP000438\_PA14\_33980 | 3020733 | 3021266 | False | - | - | - | - | - |
| CP000438\_PA14\_33990 | 3021340 | 3023889 | False | COG0542 | 0.0 | 96.0 | 1 | 761 |
| CP000438\_PA14\_34000 | 3023891 | 3024907 | False | COG3520 | 4e-79 | 100.0 | 1 | 335 |
| CP000438\_PA14\_34010 | 3024871 | 3026664 | False | COG3519 | 7e-180 | 100.0 | 1 | 621 |
| CP000438\_PA14\_34020 | 3026648 | 3027073 | False | COG3518 | 4e-25 | 95.0 | 8 | 157 |
| CP000438\_PA14\_34030 | 3027086 | 3027583 | False | COG3157 | 2e-39 | 99.0 | 2 | 162 |
| CP000438\_PA14\_34050 | 3027657 | 3029141 | False | COG3517 | 0.0 | 99.0 | 2 | 495 |
| CP000438\_PA14\_34070 | 3029164 | 3029709 | False | COG3516 | 3e-53 | 100.0 | 1 | 169 |
| CP000438\_PA14\_34080 | 3029917 | 3030393 | True | - | - | - | - | - |
| CP000438\_PA14\_34100 | 3030453 | 3031784 | True | COG3522 | 1e-104 | 100.0 | 1 | 446 |
| CP000438\_PA14\_34110 | 3031802 | 3032560 | True | COG3455 | 3e-51 | 95.0 | 13 | 262 |
| CP000438\_PA14\_34130 | 3032557 | 3036372 | True | COG3523 | 0.0 | 99.0 | 2 | 1187 |
| CP000438\_PA14\_34140 | 3036369 | 3037469 | True | COG3515 | 2e-46 | 100.0 | 1 | 346 |
| CP000438\_PA14\_34150 | 3037579 | 3038664 | True | COG1221 | 2e-95 | 82.0 | 66 | 399 |
| CP000438\_PA14\_34170 | 3038777 | 3039166 | True | - | - | - | - | - |
| CP000438\_PA14\_34180 | 3039326 | 3039886 | True | COG0431 | 6e-26 | 95.0 | 2 | 176 |
| CP000438\_PA14\_34190 | 3039896 | 3041041 | True | COG2141 | 1e-55 | 98.0 | 1 | 330 |
| CP000438\_PA14\_34200 | 3041071 | 3042255 | True | COG1960 | 8e-38 | 100.0 | 1 | 393 |
| CP000438\_PA14\_34210 | 3042252 | 3043382 | True | COG3829 | 3e-97 | 53.0 | 250 | 547 |
| CP000438\_PA14\_34230 | 3043554 | 3044708 | True | COG1485 | 4e-115 | 98.0 | 1 | 363 |
