# Supplementary material for: Dissecting the bacterial type VI secretion system by a genome wide in silico analysis: what can be learned from available microbial genomic resources?
Source: BMC Genomics. 2009 Mar 12;10:104. doi: 10.1186/1471-2164-10-104 (PMC2660368; doi:10.1186/1471-2164-10-104)
Supplement: Additional file 7 — Detailed description of all identified T6SS gene clusters. Archive containing the detailed description of each identified T6SS locus as an HTML file. [file 1471-2164-10-104-S7.tgz › LociHTML/HTML/CP000438D.html]

Locus CP000438D on Pseudomonas aeruginosa (strain UCBPP-PA14) chromosome, complete sequence.

import namespace="svg" implementation="#AdobeSVG"?


# Locus CP000438D

# List of CDS in T6SS locus CP000438D

|  |  |  |  |  |  |  |  |  |
| --- | --- | --- | --- | --- | --- | --- | --- | --- |
| Name | from | to | direct | COG | e-value | COG cover | COG hit start | COG hit end |
| CP000438\_PA14\_42820 | 3806993 | 3807589 | True | COG1335 | 2e-20 | 98.0 | 5 | 205 |
| CP000438\_PA14\_42830 | 3807762 | 3808082 | True | - | - | - | - | - |
| CP000438\_PA14\_42840 | 3808139 | 3808696 | True | COG2840 | 2e-47 | 96.0 | 7 | 184 |
| CP000438\_PA14\_42850 | 3808774 | 3809319 | True | COG0302 | 2e-78 | 92.0 | 14 | 193 |
| CP000438\_PA14\_42860 | 3809384 | 3809845 | False | COG2703 | 6e-34 | 100.0 | 1 | 144 |
| CP000438\_PA14\_42870 | 3810092 | 3810472 | True | COG3324 | 5e-30 | 95.0 | 7 | 127 |
| CP000438\_PA14\_42880 | 3810500 | 3811489 | False | COG0515 | 3e-37 | 73.0 | 2 | 284 |
| CP000438\_PA14\_42890 | 3811486 | 3812214 | False | COG0631 | 4e-59 | 95.0 | 2 | 250 |
| CP000438\_PA14\_42900 | 3812214 | 3815741 | False | COG3523 | 0.0 | 99.0 | 7 | 1187 |
| CP000438\_PA14\_42910 | 3815757 | 3816626 | False | COG3455 | 6e-76 | 100.0 | 1 | 262 |
| CP000438\_PA14\_42920 | 3816629 | 3817960 | False | COG3522 | 4e-153 | 99.0 | 2 | 446 |
| CP000438\_PA14\_42940 | 3817957 | 3818463 | False | COG3521 | 5e-42 | 97.0 | 5 | 159 |
| CP000438\_PA14\_42950 | 3818469 | 3819662 | False | COG3456 | 2e-90 | 100.0 | 1 | 430 |
| CP000438\_PA14\_42960 | 3819680 | 3819820 | False | - | - | - | - | - |
| CP000438\_PA14\_42970 | 3819909 | 3821420 | False | COG3604 | 1e-152 | 96.0 | 21 | 550 |
| CP000438\_PA14\_42980 | 3821431 | 3824064 | False | COG0542 | 0.0 | 97.0 | 2 | 766 |
| CP000438\_PA14\_42990 | 3824071 | 3825078 | False | COG3520 | 4e-88 | 99.0 | 1 | 333 |
| CP000438\_PA14\_43000 | 3825042 | 3826622 | False | COG3519 | 1e-149 | 88.0 | 75 | 621 |
| CP000438\_PA14\_43020 | 3827167 | 3827574 | False | COG3518 | 1e-28 | 89.0 | 14 | 154 |
| CP000438\_PA14\_43030 | 3827587 | 3829062 | False | COG3517 | 0.0 | 99.0 | 1 | 493 |
| CP000438\_PA14\_43040 | 3829090 | 3829596 | False | COG3516 | 2e-46 | 97.0 | 5 | 169 |
| CP000438\_PA14\_43050 | 3829632 | 3831188 | False | COG3515 | 2e-37 | 100.0 | 1 | 346 |
| CP000438\_PA14\_43070 | 3831886 | 3832404 | True | COG3157 | 4e-47 | 97.0 | 1 | 158 |
| CP000438\_PA14\_43080 | 3832600 | 3834642 | True | COG3501 | 1e-169 | 99.0 | 5 | 550 |
| CP000438\_PA14\_43090 | 3834632 | 3835372 | True | - | - | - | - | - |
| CP000438\_PA14\_43100 | 3835369 | 3840213 | True | COG3209 | 1e-41 | 82.0 | 9 | 662 |
