# Supplementary material for: Dissecting the bacterial type VI secretion system by a genome wide in silico analysis: what can be learned from available microbial genomic resources?
Source: BMC Genomics. 2009 Mar 12;10:104. doi: 10.1186/1471-2164-10-104 (PMC2660368; doi:10.1186/1471-2164-10-104)
Supplement: Additional file 7 — Detailed description of all identified T6SS gene clusters. Archive containing the detailed description of each identified T6SS locus as an HTML file. [file 1471-2164-10-104-S7.tgz › LociHTML/HTML/CP000440A.html]

Locus CP000440A on Burkholderia cepacia (strain ATCC 53795 / AMMD) chromosome 1, complete sequence.

import namespace="svg" implementation="#AdobeSVG"?


# Locus CP000440A

# List of CDS in T6SS locus CP000440A

|  |  |  |  |  |  |  |  |  |
| --- | --- | --- | --- | --- | --- | --- | --- | --- |
| Name | from | to | direct | COG | e-value | COG cover | COG hit start | COG hit end |
| CP000440\_Bamb\_0372 | 418105 | 418500 | True | - | - | - | - | - |
| CP000440\_Bamb\_0373 | 419903 | 420196 | True | - | - | - | - | - |
| CP000440\_Bamb\_0374 | 420594 | 420902 | True | - | - | - | - | - |
| CP000440\_Bamb\_0375 | 420910 | 421167 | True | - | - | - | - | - |
| CP000440\_Bamb\_0376 | 421479 | 421844 | True | - | - | - | - | - |
| CP000440\_Bamb\_0377 | 421936 | 422718 | False | COG3455 | 2e-44 | 95.0 | 14 | 262 |
| CP000440\_Bamb\_0378 | 422715 | 424061 | False | COG3522 | 6e-111 | 100.0 | 1 | 446 |
| CP000440\_Bamb\_0379 | 424166 | 424774 | False | COG3521 | 3e-28 | 92.0 | 1 | 147 |
| CP000440\_Bamb\_0380 | 425150 | 425788 | True | - | - | - | - | - |
| CP000440\_Bamb\_0381 | 425833 | 426348 | True | COG3516 | 3e-48 | 98.0 | 2 | 168 |
| CP000440\_Bamb\_0382 | 426364 | 427854 | True | COG3517 | 0.0 | 99.0 | 2 | 495 |
| CP000440\_Bamb\_0383 | 427925 | 428428 | True | COG3157 | 4e-32 | 97.0 | 5 | 162 |
| CP000440\_Bamb\_0384 | 428491 | 428976 | True | COG3518 | 7e-33 | 98.0 | 4 | 157 |
| CP000440\_Bamb\_0385 | 429054 | 430889 | True | COG3519 | 7e-154 | 99.0 | 2 | 620 |
| CP000440\_Bamb\_0386 | 430853 | 431953 | True | COG3520 | 1e-63 | 100.0 | 1 | 335 |
| CP000440\_Bamb\_0387 | 431997 | 434666 | True | COG0542 | 0.0 | 97.0 | 1 | 770 |
| CP000440\_Bamb\_0388 | 434706 | 435827 | True | COG3515 | 1e-31 | 98.0 | 7 | 346 |
| CP000440\_Bamb\_0389 | 435878 | 436756 | True | - | - | - | - | - |
| CP000440\_Bamb\_0390 | 437020 | 437535 | False | - | - | - | - | - |
| CP000440\_Bamb\_0391 | 437736 | 438686 | False | COG2885 | 5e-27 | 84.0 | 27 | 187 |
| CP000440\_Bamb\_0392 | 438691 | 439680 | False | COG3913 | 2e-34 | 93.0 | 5 | 216 |
| CP000440\_Bamb\_0393 | 439677 | 443618 | False | COG3523 | 9e-122 | 47.0 | 5 | 573 |
| CP000440\_Bamb\_0393 | 439677 | 443618 | False | COG3523 | 2e-109 | 52.0 | 565 | 1188 |
| CP000440\_Bamb\_0394 | 443920 | 444648 | False | - | - | - | - | - |
| CP000440\_Bamb\_0395 | 444887 | 445858 | True | - | - | - | - | - |
| CP000440\_Bamb\_0396 | 446072 | 447100 | True | COG0598 | 2e-47 | 90.0 | 29 | 320 |
| CP000440\_Bamb\_0397 | 447234 | 449402 | False | COG0308 | 2e-80 | 66.0 | 15 | 581 |
