# Supplementary material for: Dissecting the bacterial type VI secretion system by a genome wide in silico analysis: what can be learned from available microbial genomic resources?
Source: BMC Genomics. 2009 Mar 12;10:104. doi: 10.1186/1471-2164-10-104 (PMC2660368; doi:10.1186/1471-2164-10-104)
Supplement: Additional file 7 — Detailed description of all identified T6SS gene clusters. Archive containing the detailed description of each identified T6SS locus as an HTML file. [file 1471-2164-10-104-S7.tgz › LociHTML/HTML/CP000441E.html]

Locus CP000441E on Burkholderia cepacia (strain ATCC 53795 / AMMD) chromosome 2, complete sequence.

import namespace="svg" implementation="#AdobeSVG"?


# Locus CP000441E

# List of CDS in T6SS locus CP000441E

|  |  |  |  |  |  |  |  |  |
| --- | --- | --- | --- | --- | --- | --- | --- | --- |
| Name | from | to | direct | COG | e-value | COG cover | COG hit start | COG hit end |
| CP000441\_Bamb\_3462 | 259235 | 261625 | True | COG5002 | 3e-07 | 22.0 | 24 | 125 |
| CP000441\_Bamb\_3462 | 259235 | 261625 | True | COG5000 | 1e-06 | 41.0 | 86 | 384 |
| CP000441\_Bamb\_3462 | 259235 | 261625 | True | COG0643 | 2e-35 | 20.0 | 406 | 555 |
| CP000441\_Bamb\_3463 | 261628 | 262368 | True | - | - | - | - | - |
| CP000441\_Bamb\_3464 | 262365 | 262949 | True | - | - | - | - | - |
| CP000441\_Bamb\_3465 | 263027 | 263929 | True | - | - | - | - | - |
| CP000441\_Bamb\_3466 | 263965 | 264330 | True | COG4753 | 2e-17 | 25.0 | 1 | 121 |
| CP000441\_Bamb\_3467 | 264557 | 265660 | False | COG3515 | 1e-22 | 96.0 | 6 | 338 |
| CP000441\_Bamb\_3468 | 265657 | 266730 | False | COG3520 | 1e-77 | 94.0 | 19 | 334 |
| CP000441\_Bamb\_3469 | 266730 | 268619 | False | COG3519 | 0.0 | 100.0 | 1 | 621 |
| CP000441\_Bamb\_3470 | 268652 | 269209 | False | COG3518 | 4e-21 | 92.0 | 6 | 151 |
| CP000441\_Bamb\_3471 | 269202 | 270053 | False | COG4455 | 3e-59 | 95.0 | 13 | 273 |
| CP000441\_Bamb\_3472 | 270050 | 270496 | False | - | - | - | - | - |
| CP000441\_Bamb\_3473 | 271254 | 273947 | True | COG0542 | 0.0 | 96.0 | 1 | 761 |
| CP000441\_Bamb\_3474 | 273986 | 274528 | True | COG3516 | 8e-56 | 99.0 | 2 | 169 |
| CP000441\_Bamb\_3475 | 274556 | 276049 | True | COG3517 | 0.0 | 100.0 | 1 | 495 |
| CP000441\_Bamb\_3476 | 276142 | 276627 | True | COG3157 | 9e-37 | 100.0 | 1 | 162 |
| CP000441\_Bamb\_3477 | 276714 | 277220 | True | COG3521 | 1e-30 | 94.0 | 8 | 158 |
| CP000441\_Bamb\_3478 | 277252 | 278595 | True | COG3522 | 2e-124 | 99.0 | 1 | 445 |
| CP000441\_Bamb\_3479 | 278884 | 281421 | True | COG4253 | 7e-43 | 85.0 | 3 | 239 |
| CP000441\_Bamb\_3479 | 278884 | 281421 | True | COG3501 | 2e-108 | 92.0 | 20 | 525 |
| CP000441\_Bamb\_3480 | 281396 | 282280 | True | - | - | - | - | - |
| CP000441\_Bamb\_3481 | 282817 | 284832 | True | - | - | - | - | - |
| CP000441\_Bamb\_3482 | 284825 | 285754 | True | - | - | - | - | - |
| CP000441\_Bamb\_3483 | 285825 | 286103 | True | COG4104 | 8e-11 | 83.0 | 10 | 91 |
| CP000441\_Bamb\_3484 | 286100 | 287398 | True | COG3455 | 8e-47 | 91.0 | 21 | 260 |
| CP000441\_Bamb\_3484 | 286100 | 287398 | True | COG1360 | 5e-28 | 56.0 | 104 | 242 |
| CP000441\_Bamb\_3485 | 287424 | 291521 | True | COG3523 | 5e-34 | 16.0 | 2 | 195 |
| CP000441\_Bamb\_3485 | 287424 | 291521 | True | COG3523 | 0.0 | 85.0 | 176 | 1188 |
| CP000441\_Bamb\_3486 | 291542 | 292756 | True | COG3455 | 5e-26 | 89.0 | 7 | 241 |
| CP000441\_Bamb\_3486 | 291542 | 292756 | True | COG1360 | 4e-18 | 59.0 | 97 | 242 |
| CP000441\_Bamb\_3487 | 292778 | 293659 | False | COG0583 | 7e-26 | 99.0 | 1 | 296 |
| CP000441\_Bamb\_3488 | 293790 | 295199 | True | COG2814 | 4e-15 | 98.0 | 1 | 387 |
| CP000441\_Bamb\_3489 | 295318 | 296112 | True | COG1024 | 1e-47 | 100.0 | 1 | 257 |
| CP000441\_Bamb\_3490 | 296261 | 297382 | False | COG0787 | 2e-110 | 99.0 | 1 | 359 |
| CP000441\_Bamb\_3491 | 297686 | 298057 | True | COG3686 | 5e-19 | 98.0 | 1 | 123 |
