# Supplementary material for: Dissecting the bacterial type VI secretion system by a genome wide in silico analysis: what can be learned from available microbial genomic resources?
Source: BMC Genomics. 2009 Mar 12;10:104. doi: 10.1186/1471-2164-10-104 (PMC2660368; doi:10.1186/1471-2164-10-104)
Supplement: Additional file 7 — Detailed description of all identified T6SS gene clusters. Archive containing the detailed description of each identified T6SS locus as an HTML file. [file 1471-2164-10-104-S7.tgz › LociHTML/HTML/CP000447A.html]

Locus CP000447A on Shewanella frigidimarina (strain NCIMB 400) chromosome, complete sequence.

import namespace="svg" implementation="#AdobeSVG"?


# Locus CP000447A

# List of CDS in T6SS locus CP000447A

|  |  |  |  |  |  |  |  |  |
| --- | --- | --- | --- | --- | --- | --- | --- | --- |
| Name | from | to | direct | COG | e-value | COG cover | COG hit start | COG hit end |
| CP000447\_Sfri\_2355 | 2802296 | 2806780 | False | COG3209 | 1e-25 | 82.0 | 4 | 660 |
| CP000447\_Sfri\_2355 | 2802296 | 2806780 | False | COG3209 | 3e-09 | 76.0 | 45 | 656 |
| CP000447\_Sfri\_2356 | 2806787 | 2807731 | False | - | - | - | - | - |
| CP000447\_Sfri\_2357 | 2807728 | 2808828 | False | - | - | - | - | - |
| CP000447\_Sfri\_2358 | 2808850 | 2809140 | False | COG4104 | 5e-12 | 86.0 | 13 | 97 |
| CP000447\_Sfri\_2359 | 2809183 | 2809536 | False | - | - | - | - | - |
| CP000447\_Sfri\_2360 | 2809539 | 2811395 | False | COG3501 | 2e-131 | 98.0 | 6 | 549 |
| CP000447\_Sfri\_2361 | 2811466 | 2811945 | False | COG3157 | 5e-14 | 85.0 | 1 | 139 |
| CP000447\_Sfri\_2362 | 2812315 | 2814921 | False | COG0542 | 0.0 | 99.0 | 1 | 783 |
| CP000447\_Sfri\_2363 | 2814941 | 2815948 | False | COG3520 | 7e-49 | 93.0 | 12 | 324 |
| CP000447\_Sfri\_2364 | 2815945 | 2817774 | False | COG3519 | 8e-128 | 100.0 | 1 | 621 |
| CP000447\_Sfri\_2365 | 2817771 | 2818235 | False | COG3518 | 2e-16 | 88.0 | 8 | 146 |
| CP000447\_Sfri\_2366 | 2818268 | 2819056 | False | COG4455 | 3e-31 | 94.0 | 8 | 264 |
| CP000447\_Sfri\_2367 | 2819071 | 2820606 | False | COG3517 | 1e-128 | 91.0 | 41 | 492 |
| CP000447\_Sfri\_2368 | 2820658 | 2822151 | False | COG3517 | 0.0 | 99.0 | 1 | 493 |
| CP000447\_Sfri\_2369 | 2822151 | 2822651 | False | COG3516 | 7e-50 | 98.0 | 2 | 167 |
| CP000447\_Sfri\_2370 | 2822693 | 2823799 | False | COG3515 | 2e-15 | 97.0 | 10 | 345 |
| CP000447\_Sfri\_2371 | 2824086 | 2824853 | False | COG0631 | 1e-48 | 93.0 | 12 | 255 |
| CP000447\_Sfri\_2372 | 2824844 | 2825575 | False | COG3913 | 1e-21 | 93.0 | 5 | 217 |
| CP000447\_Sfri\_2373 | 2825557 | 2829105 | False | COG3523 | 0.0 | 99.0 | 9 | 1187 |
| CP000447\_Sfri\_2374 | 2829129 | 2830418 | False | COG1360 | 3e-23 | 83.0 | 35 | 238 |
| CP000447\_Sfri\_2374 | 2829129 | 2830418 | False | COG3455 | 3e-52 | 85.0 | 36 | 260 |
| CP000447\_Sfri\_2375 | 2830443 | 2831771 | False | COG3522 | 9e-119 | 99.0 | 1 | 444 |
| CP000447\_Sfri\_2376 | 2831796 | 2832266 | False | COG3521 | 4e-27 | 86.0 | 8 | 145 |
| CP000447\_Sfri\_2377 | 2832285 | 2833661 | False | COG3456 | 6e-44 | 99.0 | 1 | 427 |
| CP000447\_Sfri\_2378 | 2833694 | 2835679 | False | COG0515 | 3e-27 | 71.0 | 2 | 274 |
| CP000447\_Sfri\_2379 | 2835999 | 2836490 | True | COG2885 | 2e-12 | 64.0 | 68 | 190 |
| CP000447\_Sfri\_2380 | 2836885 | 2837727 | False | - | - | - | - | - |
| CP000447\_Sfri\_2381 | 2838049 | 2839122 | True | COG0845 | 5e-25 | 95.0 | 12 | 367 |
