# Supplementary material for: Dissecting the bacterial type VI secretion system by a genome wide in silico analysis: what can be learned from available microbial genomic resources?
Source: BMC Genomics. 2009 Mar 12;10:104. doi: 10.1186/1471-2164-10-104 (PMC2660368; doi:10.1186/1471-2164-10-104)
Supplement: Additional file 7 — Detailed description of all identified T6SS gene clusters. Archive containing the detailed description of each identified T6SS locus as an HTML file. [file 1471-2164-10-104-S7.tgz › LociHTML/HTML/CP000453A.html]

Locus CP000453A on Alkalilimnicola ehrlichei (strain MLHE-1) chromosome, complete sequence.

import namespace="svg" implementation="#AdobeSVG"?


# Locus CP000453A

# List of CDS in T6SS locus CP000453A

|  |  |  |  |  |  |  |  |  |
| --- | --- | --- | --- | --- | --- | --- | --- | --- |
| Name | from | to | direct | COG | e-value | COG cover | COG hit start | COG hit end |
| CP000453\_Mlg\_0040 | 41926 | 43257 | False | - | - | - | - | - |
| CP000453\_Mlg\_0041 | 43278 | 46577 | False | - | - | - | - | - |
| CP000453\_Mlg\_0042 | 46574 | 47473 | False | - | - | - | - | - |
| CP000453\_Mlg\_0043 | 47470 | 48432 | False | COG3501 | 1e-53 | 30.0 | 371 | 539 |
| CP000453\_Mlg\_0045 | 49079 | 49588 | True | COG3516 | 4e-40 | 96.0 | 7 | 169 |
| CP000453\_Mlg\_0046 | 49623 | 51101 | True | COG3517 | 0.0 | 98.0 | 4 | 493 |
| CP000453\_Mlg\_0047 | 51108 | 51536 | True | COG3518 | 2e-16 | 90.0 | 12 | 153 |
| CP000453\_Mlg\_0048 | 51556 | 53319 | True | COG3519 | 8e-154 | 99.0 | 7 | 621 |
| CP000453\_Mlg\_0049 | 53316 | 54302 | True | COG3520 | 2e-74 | 93.0 | 20 | 332 |
| CP000453\_Mlg\_0050 | 54307 | 55017 | True | COG3456 | 4e-12 | 43.0 | 1 | 189 |
| CP000453\_Mlg\_0051 | 55014 | 55586 | True | COG3521 | 2e-29 | 96.0 | 2 | 155 |
| CP000453\_Mlg\_0052 | 55586 | 56923 | True | COG3522 | 3e-136 | 100.0 | 1 | 446 |
| CP000453\_Mlg\_0053 | 56926 | 57741 | True | COG3455 | 2e-54 | 96.0 | 7 | 258 |
| CP000453\_Mlg\_0054 | 57738 | 59315 | True | COG3829 | 8e-99 | 56.0 | 247 | 560 |
| CP000453\_Mlg\_0055 | 59312 | 59959 | True | - | - | - | - | - |
| CP000453\_Mlg\_0056 | 59947 | 61389 | True | - | - | - | - | - |
| CP000453\_Mlg\_0057 | 61425 | 65042 | True | COG3523 | 0.0 | 99.0 | 9 | 1188 |
| CP000453\_Mlg\_0058 | 65285 | 65803 | True | COG3157 | 2e-48 | 98.0 | 1 | 160 |
| CP000453\_Mlg\_0059 | 65890 | 66189 | True | COG4104 | 5e-08 | 68.0 | 26 | 92 |
| CP000453\_Mlg\_0060 | 66332 | 66931 | True | - | - | - | - | - |
| CP000453\_Mlg\_0061 | 67016 | 67855 | True | COG0253 | 2e-89 | 100.0 | 1 | 272 |
| CP000453\_Mlg\_0062 | 67913 | 68611 | True | COG3159 | 1e-31 | 96.0 | 4 | 214 |
| CP000453\_Mlg\_0063 | 68618 | 69532 | True | COG4973 | 1e-97 | 98.0 | 4 | 299 |
| CP000453\_Mlg\_0064 | 69593 | 70225 | True | COG1309 | 3e-13 | 98.0 | 4 | 201 |
| CP000453\_Mlg\_0065 | 70272 | 71252 | True | COG0604 | 4e-53 | 100.0 | 1 | 326 |
