# Supplementary material for: Dissecting the bacterial type VI secretion system by a genome wide in silico analysis: what can be learned from available microbial genomic resources?
Source: BMC Genomics. 2009 Mar 12;10:104. doi: 10.1186/1471-2164-10-104 (PMC2660368; doi:10.1186/1471-2164-10-104)
Supplement: Additional file 7 — Detailed description of all identified T6SS gene clusters. Archive containing the detailed description of each identified T6SS locus as an HTML file. [file 1471-2164-10-104-S7.tgz › LociHTML/HTML/CP000458A.html]

Locus CP000458A on Burkholderia cenocepacia (strain HI2424) chromosome 1, complete sequence.

import namespace="svg" implementation="#AdobeSVG"?


# Locus CP000458A

# List of CDS in T6SS locus CP000458A

|  |  |  |  |  |  |  |  |  |
| --- | --- | --- | --- | --- | --- | --- | --- | --- |
| Name | from | to | direct | COG | e-value | COG cover | COG hit start | COG hit end |
| CP000458\_Bcen2424\_0454 | 499546 | 501162 | True | - | - | - | - | - |
| CP000458\_Bcen2424\_0455 | 501276 | 502160 | True | - | - | - | - | - |
| CP000458\_Bcen2424\_0456 | 502224 | 503174 | True | - | - | - | - | - |
| CP000458\_Bcen2424\_0457 | 503892 | 504095 | True | - | - | - | - | - |
| CP000458\_Bcen2424\_0458 | 504205 | 505005 | False | COG0834 | 6e-45 | 99.0 | 1 | 273 |
| CP000458\_Bcen2424\_0459 | 505362 | 508553 | True | COG4253 | 3e-46 | 93.0 | 1 | 260 |
| CP000458\_Bcen2424\_0459 | 505362 | 508553 | True | COG3501 | 2e-112 | 96.0 | 1 | 532 |
| CP000458\_Bcen2424\_0460 | 508605 | 513260 | True | COG3209 | 8e-42 | 83.0 | 1 | 665 |
| CP000458\_Bcen2424\_0461 | 513271 | 513846 | True | - | - | - | - | - |
| CP000458\_Bcen2424\_0462 | 514382 | 514699 | True | - | - | - | - | - |
| CP000458\_Bcen2424\_0463 | 514786 | 515568 | False | COG3455 | 3e-45 | 95.0 | 14 | 262 |
| CP000458\_Bcen2424\_0464 | 515565 | 516911 | False | COG3522 | 2e-111 | 100.0 | 1 | 446 |
| CP000458\_Bcen2424\_0465 | 517014 | 517625 | False | COG3521 | 8e-28 | 87.0 | 9 | 147 |
| CP000458\_Bcen2424\_0466 | 517998 | 518633 | True | - | - | - | - | - |
| CP000458\_Bcen2424\_0467 | 518680 | 519195 | True | COG3516 | 2e-48 | 98.0 | 2 | 168 |
| CP000458\_Bcen2424\_0468 | 519211 | 520701 | True | COG3517 | 0.0 | 99.0 | 2 | 495 |
| CP000458\_Bcen2424\_0469 | 520772 | 521275 | True | COG3157 | 4e-32 | 97.0 | 5 | 162 |
| CP000458\_Bcen2424\_0470 | 521338 | 521823 | True | COG3518 | 1e-33 | 96.0 | 4 | 155 |
| CP000458\_Bcen2424\_0471 | 521900 | 523735 | True | COG3519 | 4e-155 | 99.0 | 2 | 620 |
| CP000458\_Bcen2424\_0472 | 523699 | 524799 | True | COG3520 | 4e-63 | 100.0 | 1 | 335 |
| CP000458\_Bcen2424\_0473 | 524841 | 527510 | True | COG0542 | 0.0 | 97.0 | 1 | 770 |
| CP000458\_Bcen2424\_0474 | 527554 | 528675 | True | COG3515 | 1e-29 | 98.0 | 7 | 346 |
| CP000458\_Bcen2424\_0475 | 528742 | 531303 | True | COG4253 | 1e-51 | 79.0 | 1 | 221 |
| CP000458\_Bcen2424\_0475 | 528742 | 531303 | True | COG3501 | 4e-110 | 96.0 | 1 | 532 |
| CP000458\_Bcen2424\_0476 | 531303 | 532343 | True | - | - | - | - | - |
| CP000458\_Bcen2424\_0477 | 532330 | 533166 | True | - | - | - | - | - |
| CP000458\_Bcen2424\_0478 | 533386 | 534321 | True | - | - | - | - | - |
| CP000458\_Bcen2424\_0479 | 534308 | 536569 | True | - | - | - | - | - |
| CP000458\_Bcen2424\_0480 | 536652 | 536909 | False | COG2885 | 3e-21 | 43.0 | 105 | 187 |
| CP000458\_Bcen2424\_0481 | 537086 | 537955 | True | - | - | - | - | - |
| CP000458\_Bcen2424\_0482 | 537988 | 538608 | True | - | - | - | - | - |
| CP000458\_Bcen2424\_0483 | 538876 | 539826 | False | COG2885 | 3e-26 | 84.0 | 27 | 187 |
| CP000458\_Bcen2424\_0484 | 539831 | 540820 | False | COG3913 | 1e-34 | 93.0 | 5 | 216 |
| CP000458\_Bcen2424\_0485 | 540817 | 544761 | False | COG3523 | 1e-121 | 47.0 | 5 | 573 |
| CP000458\_Bcen2424\_0485 | 540817 | 544761 | False | COG3523 | 4e-109 | 52.0 | 565 | 1188 |
| CP000458\_Bcen2424\_0486 | 545063 | 545908 | False | - | - | - | - | - |
| CP000458\_Bcen2424\_0487 | 546030 | 547034 | True | - | - | - | - | - |
| CP000458\_Bcen2424\_0488 | 547247 | 548275 | True | COG0598 | 2e-46 | 90.0 | 29 | 320 |
| CP000458\_Bcen2424\_0489 | 548319 | 548471 | False | COG4317 | 6e-09 | 53.0 | 1 | 50 |
| CP000458\_Bcen2424\_0490 | 548496 | 549473 | False | COG0604 | 2e-43 | 100.0 | 1 | 326 |
| CP000458\_Bcen2424\_0491 | 549637 | 550557 | True | COG0583 | 2e-28 | 97.0 | 5 | 295 |
