# Supplementary material for: Dissecting the bacterial type VI secretion system by a genome wide in silico analysis: what can be learned from available microbial genomic resources?
Source: BMC Genomics. 2009 Mar 12;10:104. doi: 10.1186/1471-2164-10-104 (PMC2660368; doi:10.1186/1471-2164-10-104)
Supplement: Additional file 7 — Detailed description of all identified T6SS gene clusters. Archive containing the detailed description of each identified T6SS locus as an HTML file. [file 1471-2164-10-104-S7.tgz › LociHTML/HTML/CP000462B.html]

Locus CP000462B on Aeromonas hydrophila (subsp. hydrophila, strain ATCC 7966 / NCIB 9240) chromosome, complete sequence.

import namespace="svg" implementation="#AdobeSVG"?


# Locus CP000462B

# List of CDS in T6SS locus CP000462B

|  |  |  |  |  |  |  |  |  |
| --- | --- | --- | --- | --- | --- | --- | --- | --- |
| Name | from | to | direct | COG | e-value | COG cover | COG hit start | COG hit end |
| CP000462\_AHA\_1821 | 1984799 | 1986379 | True | COG1757 | 1e-70 | 99.0 | 1 | 481 |
| CP000462\_AHA\_1822 | 1986466 | 1987047 | True | COG1435 | 2e-59 | 94.0 | 3 | 191 |
| CP000462\_AHA\_1823 | 1987116 | 1988453 | False | COG3930 | 1e-57 | 98.0 | 8 | 434 |
| CP000462\_AHA\_1824 | 1988606 | 1989067 | True | - | - | - | - | - |
| CP000462\_AHA\_1826 | 1990515 | 1991033 | True | COG3157 | 2e-49 | 98.0 | 1 | 160 |
| CP000462\_AHA\_1827 | 1991311 | 1993542 | True | COG3501 | 5e-156 | 96.0 | 6 | 537 |
| CP000462\_AHA\_1828 | 1993544 | 1994863 | True | - | - | - | - | - |
| CP000462\_AHA\_1829 | 1994873 | 1995862 | True | - | - | - | - | - |
| CP000462\_AHA\_1830 | 1995977 | 1996522 | True | - | - | - | - | - |
| CP000462\_AHA\_1831 | 1996643 | 1998031 | True | COG0790 | 5e-12 | 67.0 | 34 | 230 |
| CP000462\_AHA\_1831 | 1996643 | 1998031 | True | COG0790 | 2e-12 | 80.0 | 51 | 285 |
| CP000462\_AHA\_1832 | 1998704 | 1999207 | True | COG3516 | 7e-43 | 94.0 | 10 | 168 |
| CP000462\_AHA\_1833 | 1999252 | 2000724 | True | COG3517 | 0.0 | 99.0 | 1 | 493 |
| CP000462\_AHA\_1834 | 2000731 | 2001162 | True | COG3518 | 5e-19 | 92.0 | 7 | 152 |
| CP000462\_AHA\_1835 | 2001166 | 2002932 | True | COG3519 | 8e-147 | 99.0 | 3 | 621 |
| CP000462\_AHA\_1836 | 2002896 | 2003894 | True | COG3520 | 9e-82 | 97.0 | 4 | 328 |
| CP000462\_AHA\_1837 | 2003951 | 2005201 | True | COG3456 | 3e-69 | 99.0 | 1 | 428 |
| CP000462\_AHA\_1838 | 2005201 | 2005716 | True | COG3521 | 6e-34 | 96.0 | 6 | 159 |
| CP000462\_AHA\_1839 | 2005719 | 2007053 | True | COG3522 | 7e-149 | 100.0 | 1 | 446 |
| CP000462\_AHA\_1840 | 2007145 | 2007855 | True | COG3455 | 9e-64 | 89.0 | 26 | 260 |
| CP000462\_AHA\_1841 | 2007876 | 2010518 | True | COG0542 | 0.0 | 96.0 | 1 | 762 |
| CP000462\_AHA\_1842 | 2010521 | 2012059 | True | COG2204 | 2e-94 | 72.0 | 121 | 457 |
| CP000462\_AHA\_1843 | 2012059 | 2012664 | True | - | - | - | - | - |
| CP000462\_AHA\_1844 | 2012673 | 2014109 | True | COG3515 | 1e-15 | 73.0 | 32 | 285 |
| CP000462\_AHA\_1845 | 2014151 | 2017636 | True | COG3523 | 0.0 | 99.0 | 5 | 1188 |
| CP000462\_AHA\_1846 | 2017683 | 2019113 | True | COG3515 | 7e-20 | 47.0 | 19 | 184 |
| CP000462\_AHA\_1847 | 2019371 | 2019658 | True | COG4104 | 4e-08 | 91.0 | 3 | 92 |
| CP000462\_AHA\_1848 | 2019669 | 2021714 | True | COG3501 | 1e-157 | 97.0 | 6 | 539 |
| CP000462\_AHA\_1849 | 2021749 | 2024304 | True | - | - | - | - | - |
| CP000462\_AHA\_1850 | 2024307 | 2024711 | True | COG3755 | 5e-08 | 94.0 | 6 | 125 |
| CP000462\_AHA\_1854 | 2027356 | 2028219 | True | COG0190 | 2e-113 | 99.0 | 1 | 282 |
| CP000462\_AHA\_1855 | 2028327 | 2028545 | False | COG1278 | 2e-22 | 98.0 | 1 | 66 |
| CP000462\_AHA\_1856 | 2028774 | 2029091 | True | COG2127 | 2e-31 | 86.0 | 15 | 107 |
| CP000462\_AHA\_1857 | 2029151 | 2031403 | True | COG0542 | 0.0 | 99.0 | 1 | 785 |
| CP000462\_AHA\_1858 | 2031473 | 2031712 | False | COG0361 | 6e-26 | 96.0 | 1 | 72 |
| CP000462\_AHA\_1859 | 2031761 | 2032477 | False | COG2935 | 2e-57 | 91.0 | 10 | 240 |
| CP000462\_AHA\_1860 | 2032474 | 2033181 | False | COG2360 | 7e-81 | 96.0 | 3 | 215 |
| CP000462\_AHA\_1861 | 2033201 | 2033671 | True | COG3133 | 3e-15 | 98.0 | 1 | 152 |
| CP000462\_AHA\_1862 | 2033782 | 2035029 | False | COG3706 | 8e-57 | 89.0 | 45 | 435 |
| CP000462\_AHA\_1863 | 2035087 | 2036268 | False | COG0492 | 8e-102 | 98.0 | 2 | 301 |
