# Supplementary material for: Dissecting the bacterial type VI secretion system by a genome wide in silico analysis: what can be learned from available microbial genomic resources?
Source: BMC Genomics. 2009 Mar 12;10:104. doi: 10.1186/1471-2164-10-104 (PMC2660368; doi:10.1186/1471-2164-10-104)
Supplement: Additional file 7 — Detailed description of all identified T6SS gene clusters. Archive containing the detailed description of each identified T6SS locus as an HTML file. [file 1471-2164-10-104-S7.tgz › LociHTML/HTML/CP000468A.html]

Locus CP000468A on Escherichia coli O1:K1 / APEC chromosome, complete sequence.

import namespace="svg" implementation="#AdobeSVG"?


# Locus CP000468A

# List of CDS in T6SS locus CP000468A

|  |  |  |  |  |  |  |  |  |
| --- | --- | --- | --- | --- | --- | --- | --- | --- |
| Name | from | to | direct | COG | e-value | COG cover | COG hit start | COG hit end |
| CP000468\_Ecok1\_01980 | 237543 | 238901 | False | COG1388 | 6e-09 | 93.0 | 1 | 116 |
| CP000468\_Ecok1\_01980 | 237543 | 238901 | False | COG0741 | 2e-12 | 93.0 | 1 | 278 |
| CP000468\_Ecok1\_01990 | 238973 | 239728 | False | COG0491 | 2e-27 | 92.0 | 19 | 252 |
| CP000468\_Ecok1\_02000 | 239744 | 240484 | True | COG2226 | 1e-07 | 26.0 | 106 | 168 |
| CP000468\_Ecok1\_02010 | 240481 | 241059 | False | COG0328 | 3e-57 | 99.0 | 2 | 154 |
| CP000468\_Ecok1\_02020 | 241004 | 241744 | True | COG0847 | 7e-51 | 95.0 | 8 | 240 |
| CP000468\_Ecok1\_02030 | 241646 | 241960 | False | - | - | - | - | - |
| CP000468\_Ecok1\_02040 | 242282 | 243067 | True | - | - | - | - | - |
| CP000468\_Ecok1\_02050 | 243407 | 243886 | False | COG3157 | 1e-38 | 98.0 | 1 | 159 |
| CP000468\_Ecok1\_02060 | 243904 | 245403 | False | COG3515 | 4e-42 | 96.0 | 10 | 344 |
| CP000468\_Ecok1\_02070 | 245273 | 248800 | False | COG3523 | 0.0 | 99.0 | 2 | 1188 |
| CP000468\_Ecok1\_02080 | 248820 | 250262 | False | COG3515 | 3e-25 | 76.0 | 19 | 284 |
| CP000468\_Ecok1\_02090 | 250267 | 251010 | False | - | - | - | - | - |
| CP000468\_Ecok1\_02100 | 251007 | 253766 | False | COG0542 | 9e-126 | 59.0 | 1 | 464 |
| CP000468\_Ecok1\_02100 | 251007 | 253766 | False | COG0542 | 3e-96 | 47.0 | 414 | 786 |
| CP000468\_Ecok1\_02110 | 253776 | 254540 | False | COG3455 | 5e-77 | 92.0 | 19 | 260 |
| CP000468\_Ecok1\_02120 | 254545 | 255891 | False | COG3522 | 2e-135 | 99.0 | 4 | 446 |
| CP000468\_Ecok1\_02130 | 255894 | 256418 | False | COG3521 | 1e-34 | 99.0 | 1 | 158 |
| CP000468\_Ecok1\_02140 | 256415 | 257707 | False | COG3456 | 3e-94 | 99.0 | 2 | 430 |
| CP000468\_Ecok1\_02150 | 257712 | 258761 | False | COG3520 | 9e-84 | 94.0 | 15 | 332 |
| CP000468\_Ecok1\_02160 | 258725 | 260575 | False | COG3519 | 2e-148 | 99.0 | 3 | 621 |
| CP000468\_Ecok1\_02170 | 260572 | 260997 | False | COG3518 | 4e-20 | 96.0 | 4 | 154 |
| CP000468\_Ecok1\_02180 | 261002 | 262486 | False | COG3517 | 0.0 | 99.0 | 1 | 493 |
| CP000468\_Ecok1\_02190 | 262509 | 263012 | False | COG3516 | 2e-33 | 94.0 | 8 | 167 |
| CP000468\_Ecok1\_02200 | 263718 | 264236 | True | COG3157 | 5e-52 | 98.0 | 1 | 160 |
| CP000468\_Ecok1\_02210 | 264457 | 266439 | True | COG3501 | 8e-155 | 97.0 | 11 | 545 |
| CP000468\_Ecok1\_02220 | 266546 | 267592 | True | COG5351 | 2e-43 | 87.0 | 1 | 322 |
| CP000468\_Ecok1\_02230 | 267585 | 269024 | True | - | - | - | - | - |
| CP000468\_Ecok1\_02240 | 268999 | 269289 | True | - | - | - | - | - |
| CP000468\_Ecok1\_02250 | 269826 | 270311 | True | COG5433 | 4e-12 | 84.0 | 1 | 102 |
| CP000468\_Ecok1\_02260 | 270540 | 271043 | True | - | - | - | - | - |
| CP000468\_Ecok1\_02270 | 271113 | 271625 | True | - | - | - | - | - |
