# Supplementary material for: Dissecting the bacterial type VI secretion system by a genome wide in silico analysis: what can be learned from available microbial genomic resources?
Source: BMC Genomics. 2009 Mar 12;10:104. doi: 10.1186/1471-2164-10-104 (PMC2660368; doi:10.1186/1471-2164-10-104)
Supplement: Additional file 7 — Detailed description of all identified T6SS gene clusters. Archive containing the detailed description of each identified T6SS locus as an HTML file. [file 1471-2164-10-104-S7.tgz › LociHTML/HTML/CP000468D.html]

Locus CP000468D on Escherichia coli O1:K1 / APEC chromosome, complete sequence.

import namespace="svg" implementation="#AdobeSVG"?


# Locus CP000468D

# List of CDS in T6SS locus CP000468D

|  |  |  |  |  |  |  |  |  |
| --- | --- | --- | --- | --- | --- | --- | --- | --- |
| Name | from | to | direct | COG | e-value | COG cover | COG hit start | COG hit end |
| CP000468\_Ecok1\_27420 | 3112349 | 3113554 | True | COG0520 | 3e-124 | 100.0 | 1 | 405 |
| CP000468\_Ecok1\_27430 | 3113554 | 3113997 | True | COG2166 | 3e-45 | 97.0 | 1 | 141 |
| CP000468\_Ecok1\_27440 | 3114048 | 3114854 | False | COG1179 | 2e-113 | 99.0 | 1 | 262 |
| CP000468\_Ecok1\_27450 | 3114931 | 3116229 | False | COG2821 | 2e-130 | 100.0 | 1 | 373 |
| CP000468\_Ecok1\_27460 | 3117169 | 3117669 | True | COG3516 | 7e-40 | 96.0 | 6 | 168 |
| CP000468\_Ecok1\_27470 | 3117722 | 3119266 | True | COG3517 | 0.0 | 100.0 | 1 | 495 |
| CP000468\_Ecok1\_27480 | 3119284 | 3120621 | True | COG3522 | 2e-94 | 99.0 | 2 | 446 |
| CP000468\_Ecok1\_27490 | 3120618 | 3121283 | True | COG3455 | 3e-28 | 80.0 | 43 | 254 |
| CP000468\_Ecok1\_27500 | 3121335 | 3122948 | True | COG2885 | 1e-26 | 83.0 | 33 | 190 |
| CP000468\_Ecok1\_27510 | 3123006 | 3123497 | True | COG3157 | 5e-39 | 96.0 | 1 | 157 |
| CP000468\_Ecok1\_27520 | 3123689 | 3126334 | True | COG0542 | 0.0 | 99.0 | 1 | 781 |
| CP000468\_Ecok1\_27530 | 3126346 | 3128826 | True | COG4253 | 3e-43 | 98.0 | 2 | 276 |
| CP000468\_Ecok1\_27530 | 3126346 | 3128826 | True | COG3501 | 1e-86 | 98.0 | 8 | 549 |
| CP000468\_Ecok1\_27540 | 3128846 | 3130012 | True | - | - | - | - | - |
| CP000468\_Ecok1\_27550 | 3130225 | 3130605 | True | - | - | - | - | - |
| CP000468\_Ecok1\_27560 | 3130580 | 3131413 | True | - | - | - | - | - |
| CP000468\_Ecok1\_27570 | 3131382 | 3131675 | True | - | - | - | - | - |
| CP000468\_Ecok1\_27580 | 3133547 | 3136885 | True | COG3523 | 3e-97 | 96.0 | 45 | 1188 |
| CP000468\_Ecok1\_27590 | 3136943 | 3137692 | False | COG1484 | 3e-40 | 94.0 | 6 | 246 |
| CP000468\_Ecok1\_27600 | 3137704 | 3139245 | False | COG4584 | 5e-31 | 97.0 | 1 | 270 |
| CP000468\_Ecok1\_27610 | 3139452 | 3141089 | True | COG3515 | 7e-18 | 86.0 | 9 | 309 |
| CP000468\_Ecok1\_27620 | 3141089 | 3142672 | True | - | - | - | - | - |
| CP000468\_Ecok1\_27630 | 3142672 | 3143301 | True | - | - | - | - | - |
| CP000468\_Ecok1\_27640 | 3143558 | 3144001 | True | - | - | - | - | - |
| CP000468\_Ecok1\_27650 | 3144371 | 3146131 | True | COG3519 | 2e-147 | 100.0 | 1 | 621 |
| CP000468\_Ecok1\_27660 | 3146095 | 3147174 | True | COG3520 | 5e-59 | 95.0 | 14 | 334 |
| CP000468\_Ecok1\_27670 | 3147155 | 3147691 | True | COG3521 | 2e-15 | 86.0 | 8 | 145 |
| CP000468\_Ecok1\_27680 | 3147695 | 3148123 | True | COG3518 | 2e-13 | 91.0 | 7 | 150 |
| CP000468\_Ecok1\_27690 | 3148123 | 3149499 | True | COG3515 | 6e-11 | 46.0 | 32 | 192 |
| CP000468\_Ecok1\_27700 | 3149801 | 3150748 | False | COG0111 | 6e-62 | 82.0 | 48 | 313 |
| CP000468\_Ecok1\_27710 | 3150820 | 3151416 | False | COG0794 | 1e-48 | 94.0 | 4 | 193 |
| CP000468\_Ecok1\_27720 | 3151419 | 3152594 | False | COG1168 | 2e-101 | 99.0 | 4 | 388 |
| CP000468\_Ecok1\_27730 | 3152594 | 3154174 | False | COG1263 | 3e-42 | 97.0 | 1 | 384 |
| CP000468\_Ecok1\_27730 | 3152594 | 3154174 | False | COG1264 | 2e-16 | 89.0 | 2 | 80 |
