# Supplementary material for: Dissecting the bacterial type VI secretion system by a genome wide in silico analysis: what can be learned from available microbial genomic resources?
Source: BMC Genomics. 2009 Mar 12;10:104. doi: 10.1186/1471-2164-10-104 (PMC2660368; doi:10.1186/1471-2164-10-104)
Supplement: Additional file 7 — Detailed description of all identified T6SS gene clusters. Archive containing the detailed description of each identified T6SS locus as an HTML file. [file 1471-2164-10-104-S7.tgz › LociHTML/HTML/CP000473A.html]

Locus CP000473A on Solibacter usitatus (strain Ellin6076) chromosome, complete sequence.

import namespace="svg" implementation="#AdobeSVG"?


# Locus CP000473A

# List of CDS in T6SS locus CP000473A

|  |  |  |  |  |  |  |  |  |
| --- | --- | --- | --- | --- | --- | --- | --- | --- |
| Name | from | to | direct | COG | e-value | COG cover | COG hit start | COG hit end |
| CP000473\_Acid\_0220 | 293998 | 296787 | False | COG3280 | 0.0 | 99.0 | 5 | 886 |
| CP000473\_Acid\_0221 | 296820 | 297038 | False | - | - | - | - | - |
| CP000473\_Acid\_0222 | 297580 | 299172 | True | COG0515 | 1e-45 | 74.0 | 1 | 286 |
| CP000473\_Acid\_0223 | 299183 | 302560 | False | COG3523 | 4e-86 | 93.0 | 46 | 1151 |
| CP000473\_Acid\_0224 | 302569 | 303273 | False | COG3455 | 2e-26 | 70.0 | 63 | 246 |
| CP000473\_Acid\_0225 | 303278 | 304654 | False | COG3522 | 2e-88 | 100.0 | 1 | 446 |
| CP000473\_Acid\_0226 | 304752 | 306737 | True | - | - | - | - | - |
| CP000473\_Acid\_0227 | 306739 | 308094 | False | COG3522 | 5e-91 | 99.0 | 1 | 444 |
| CP000473\_Acid\_0228 | 308238 | 309704 | True | COG2890 | 3e-16 | 46.0 | 102 | 232 |
| CP000473\_Acid\_0229 | 309711 | 311270 | True | COG2890 | 5e-18 | 55.0 | 79 | 232 |
| CP000473\_Acid\_0230 | 311287 | 312777 | False | COG4797 | 8e-07 | 16.0 | 9 | 53 |
| CP000473\_Acid\_0230 | 311287 | 312777 | False | COG0220 | 2e-09 | 56.0 | 33 | 161 |
| CP000473\_Acid\_0231 | 312973 | 313491 | True | COG3516 | 1e-46 | 98.0 | 3 | 168 |
| CP000473\_Acid\_0232 | 313484 | 314971 | True | COG3517 | 0.0 | 99.0 | 1 | 492 |
| CP000473\_Acid\_0233 | 315027 | 315515 | True | COG3157 | 2e-34 | 100.0 | 1 | 162 |
| CP000473\_Acid\_0234 | 315543 | 316280 | True | COG4455 | 2e-34 | 97.0 | 6 | 271 |
| CP000473\_Acid\_0235 | 316264 | 318090 | True | COG3515 | 2e-15 | 90.0 | 1 | 312 |
| CP000473\_Acid\_0236 | 318094 | 318594 | True | COG3518 | 1e-22 | 91.0 | 10 | 153 |
| CP000473\_Acid\_0237 | 318584 | 320425 | True | COG3519 | 4e-154 | 100.0 | 1 | 621 |
| CP000473\_Acid\_0238 | 320497 | 321423 | True | COG3520 | 1e-70 | 85.0 | 36 | 321 |
| CP000473\_Acid\_0239 | 321443 | 324115 | True | COG0542 | 0.0 | 98.0 | 1 | 777 |
| CP000473\_Acid\_0240 | 324146 | 326167 | True | COG3501 | 3e-130 | 97.0 | 5 | 539 |
| CP000473\_Acid\_0241 | 326169 | 326750 | True | - | - | - | - | - |
| CP000473\_Acid\_0242 | 326766 | 327056 | True | COG4104 | 5e-07 | 95.0 | 4 | 97 |
| CP000473\_Acid\_0244 | 328358 | 329488 | False | COG0599 | 6e-20 | 95.0 | 1 | 119 |
| CP000473\_Acid\_0244 | 328358 | 329488 | False | COG0596 | 7e-17 | 98.0 | 2 | 279 |
| CP000473\_Acid\_0245 | 329491 | 330774 | False | COG0015 | 8e-86 | 97.0 | 6 | 434 |
| CP000473\_Acid\_0246 | 330782 | 331330 | False | COG3485 | 5e-36 | 87.0 | 30 | 226 |
