# Supplementary material for: Dissecting the bacterial type VI secretion system by a genome wide in silico analysis: what can be learned from available microbial genomic resources?
Source: BMC Genomics. 2009 Mar 12;10:104. doi: 10.1186/1471-2164-10-104 (PMC2660368; doi:10.1186/1471-2164-10-104)
Supplement: Additional file 7 — Detailed description of all identified T6SS gene clusters. Archive containing the detailed description of each identified T6SS locus as an HTML file. [file 1471-2164-10-104-S7.tgz › LociHTML/HTML/CP000489A.html]

Locus CP000489A on Paracoccus denitrificans (strain Pd 1222) chromosome 1, complete sequence.

import namespace="svg" implementation="#AdobeSVG"?


# Locus CP000489A

# List of CDS in T6SS locus CP000489A

|  |  |  |  |  |  |  |  |  |
| --- | --- | --- | --- | --- | --- | --- | --- | --- |
| Name | from | to | direct | COG | e-value | COG cover | COG hit start | COG hit end |
| CP000489\_Pden\_2434 | 2437625 | 2438041 | True | - | - | - | - | - |
| CP000489\_Pden\_2435 | 2438041 | 2438394 | True | - | - | - | - | - |
| CP000489\_Pden\_2436 | 2438382 | 2439323 | False | - | - | - | - | - |
| CP000489\_Pden\_2437 | 2439316 | 2440302 | False | - | - | - | - | - |
| CP000489\_Pden\_2438 | 2440299 | 2441363 | False | COG0304 | 6e-12 | 78.0 | 49 | 372 |
| CP000489\_Pden\_2439 | 2441360 | 2442460 | False | COG5351 | 3e-92 | 99.0 | 1 | 365 |
| CP000489\_Pden\_2440 | 2442469 | 2445216 | False | COG0542 | 6e-121 | 58.0 | 1 | 459 |
| CP000489\_Pden\_2440 | 2442469 | 2445216 | False | COG0542 | 2e-95 | 50.0 | 386 | 781 |
| CP000489\_Pden\_2441 | 2445389 | 2447494 | True | COG0515 | 4e-30 | 60.0 | 2 | 233 |
| CP000489\_Pden\_2442 | 2447625 | 2448755 | True | COG3515 | 1e-14 | 97.0 | 6 | 343 |
| CP000489\_Pden\_2443 | 2448813 | 2449355 | True | COG3516 | 7e-44 | 94.0 | 2 | 161 |
| CP000489\_Pden\_2444 | 2449359 | 2450873 | True | COG3517 | 0.0 | 100.0 | 1 | 495 |
| CP000489\_Pden\_2445 | 2451018 | 2451506 | True | COG3157 | 2e-23 | 94.0 | 1 | 153 |
| CP000489\_Pden\_2446 | 2451532 | 2452278 | True | COG3518 | 1e-14 | 85.0 | 16 | 149 |
| CP000489\_Pden\_2447 | 2452278 | 2454140 | True | COG3519 | 2e-128 | 99.0 | 1 | 618 |
| CP000489\_Pden\_2448 | 2454104 | 2455141 | True | COG3520 | 6e-52 | 97.0 | 1 | 328 |
| CP000489\_Pden\_2449 | 2455141 | 2456376 | True | COG3456 | 5e-56 | 98.0 | 3 | 425 |
| CP000489\_Pden\_2450 | 2456373 | 2456846 | True | COG3521 | 2e-17 | 92.0 | 2 | 148 |
| CP000489\_Pden\_2451 | 2456858 | 2458192 | True | COG3522 | 3e-105 | 100.0 | 1 | 446 |
| CP000489\_Pden\_2452 | 2458202 | 2459593 | True | COG3455 | 1e-42 | 98.0 | 3 | 260 |
| CP000489\_Pden\_2452 | 2458202 | 2459593 | True | COG1360 | 1e-24 | 48.0 | 126 | 244 |
| CP000489\_Pden\_2453 | 2459594 | 2463124 | True | COG3523 | 0.0 | 99.0 | 1 | 1187 |
| CP000489\_Pden\_2454 | 2463106 | 2463621 | True | COG3913 | 1e-12 | 61.0 | 7 | 145 |
| CP000489\_Pden\_2455 | 2463608 | 2465959 | False | COG3501 | 3e-141 | 95.0 | 23 | 547 |
| CP000489\_Pden\_2456 | 2466042 | 2468447 | False | COG3523 | 3e-38 | 67.0 | 390 | 1187 |
| CP000489\_Pden\_2457 | 2468423 | 2469370 | False | - | - | - | - | - |
| CP000489\_Pden\_2458 | 2469372 | 2470148 | False | COG0631 | 5e-47 | 93.0 | 6 | 250 |
| CP000489\_Pden\_2459 | 2470233 | 2470826 | True | COG2885 | 1e-18 | 57.0 | 78 | 186 |
| CP000489\_Pden\_2460 | 2470943 | 2472706 | True | COG2831 | 7e-53 | 93.0 | 11 | 526 |
| CP000489\_Pden\_2461 | 2472753 | 2477666 | True | - | - | - | - | - |
