# Supplementary material for: Dissecting the bacterial type VI secretion system by a genome wide in silico analysis: what can be learned from available microbial genomic resources?
Source: BMC Genomics. 2009 Mar 12;10:104. doi: 10.1186/1471-2164-10-104 (PMC2660368; doi:10.1186/1471-2164-10-104)
Supplement: Additional file 7 — Detailed description of all identified T6SS gene clusters. Archive containing the detailed description of each identified T6SS locus as an HTML file. [file 1471-2164-10-104-S7.tgz › LociHTML/HTML/CP000510A.html]

Locus CP000510A on Psychromonas ingrahamii (strain 37) chromosome, complete sequence.

import namespace="svg" implementation="#AdobeSVG"?


# Locus CP000510A

# List of CDS in T6SS locus CP000510A

|  |  |  |  |  |  |  |  |  |
| --- | --- | --- | --- | --- | --- | --- | --- | --- |
| Name | from | to | direct | COG | e-value | COG cover | COG hit start | COG hit end |
| CP000510\_Ping\_0007 | 12572 | 13027 | False | - | - | - | - | - |
| CP000510\_Ping\_0008 | 13204 | 15375 | False | COG0210 | 1e-165 | 99.0 | 1 | 649 |
| CP000510\_Ping\_0009 | 15412 | 16119 | False | COG1011 | 3e-20 | 97.0 | 4 | 226 |
| CP000510\_Ping\_0010 | 16153 | 17052 | False | COG4973 | 3e-108 | 98.0 | 5 | 299 |
| CP000510\_Ping\_0011 | 17602 | 18108 | True | COG3516 | 3e-35 | 98.0 | 2 | 168 |
| CP000510\_Ping\_0012 | 18217 | 19665 | True | COG3517 | 6e-159 | 96.0 | 15 | 493 |
| CP000510\_Ping\_0013 | 19699 | 20217 | True | COG3157 | 1e-21 | 95.0 | 1 | 155 |
| CP000510\_Ping\_0014 | 20228 | 20935 | True | - | - | - | - | - |
| CP000510\_Ping\_0015 | 20951 | 22315 | True | COG3522 | 2e-27 | 98.0 | 7 | 446 |
| CP000510\_Ping\_0016 | 22319 | 22966 | True | COG3455 | 6e-20 | 70.0 | 69 | 253 |
| CP000510\_Ping\_0017 | 22981 | 26403 | True | COG3523 | 6e-13 | 32.0 | 1 | 392 |
| CP000510\_Ping\_0018 | 26407 | 28101 | True | COG3516 | 4e-08 | 61.0 | 17 | 120 |
| CP000510\_Ping\_0018 | 26407 | 28101 | True | COG3517 | 2e-78 | 85.0 | 61 | 484 |
| CP000510\_Ping\_0019 | 28101 | 28589 | True | COG3157 | 3e-20 | 95.0 | 1 | 155 |
| CP000510\_Ping\_0020 | 28589 | 28993 | True | - | - | - | - | - |
| CP000510\_Ping\_0021 | 28993 | 30762 | True | COG3519 | 4e-63 | 99.0 | 3 | 617 |
| CP000510\_Ping\_0022 | 30759 | 31712 | True | COG3520 | 6e-28 | 93.0 | 15 | 327 |
| CP000510\_Ping\_0023 | 31751 | 34876 | True | COG3501 | 1e-76 | 90.0 | 23 | 518 |
| CP000510\_Ping\_0024 | 34893 | 37742 | True | COG1357 | 2e-18 | 81.0 | 6 | 200 |
| CP000510\_Ping\_0024 | 34893 | 37742 | True | COG5351 | 2e-20 | 68.0 | 27 | 279 |
| CP000510\_Ping\_0024 | 34893 | 37742 | True | COG1357 | 5e-23 | 87.0 | 31 | 238 |
| CP000510\_Ping\_0025 | 37743 | 38789 | True | COG1357 | 2e-14 | 85.0 | 6 | 209 |
| CP000510\_Ping\_0025 | 37743 | 38789 | True | COG1357 | 2e-18 | 83.0 | 39 | 236 |
| CP000510\_Ping\_0026 | 38873 | 39523 | True | - | - | - | - | - |
| CP000510\_Ping\_0027 | 39545 | 39958 | True | - | - | - | - | - |
| CP000510\_Ping\_0028 | 40134 | 43757 | True | COG3501 | 6e-80 | 94.0 | 24 | 545 |
| CP000510\_Ping\_0029 | 43781 | 46711 | True | COG1357 | 3e-20 | 97.0 | 2 | 232 |
| CP000510\_Ping\_0029 | 43781 | 46711 | True | COG1357 | 9e-08 | 84.0 | 16 | 216 |
| CP000510\_Ping\_0029 | 43781 | 46711 | True | COG5351 | 8e-25 | 65.0 | 71 | 309 |
| CP000510\_Ping\_0030 | 46717 | 47796 | True | COG1357 | 8e-16 | 82.0 | 39 | 235 |
| CP000510\_Ping\_0031 | 47840 | 48484 | True | - | - | - | - | - |
| CP000510\_Ping\_0032 | 48493 | 48903 | True | - | - | - | - | - |
