# Supplementary material for: Dissecting the bacterial type VI secretion system by a genome wide in silico analysis: what can be learned from available microbial genomic resources?
Source: BMC Genomics. 2009 Mar 12;10:104. doi: 10.1186/1471-2164-10-104 (PMC2660368; doi:10.1186/1471-2164-10-104)
Supplement: Additional file 7 — Detailed description of all identified T6SS gene clusters. Archive containing the detailed description of each identified T6SS locus as an HTML file. [file 1471-2164-10-104-S7.tgz › LociHTML/HTML/CP000512B.html]

Locus CP000512B on Acidovorax avenae (subsp. citrulli, strain AAC00-1) chromosome, complete sequence.

import namespace="svg" implementation="#AdobeSVG"?


# Locus CP000512B

# List of CDS in T6SS locus CP000512B

|  |  |  |  |  |  |  |  |  |
| --- | --- | --- | --- | --- | --- | --- | --- | --- |
| Name | from | to | direct | COG | e-value | COG cover | COG hit start | COG hit end |
| CP000512\_Aave\_1452 | 1598563 | 1600005 | False | COG0277 | 3e-90 | 99.0 | 1 | 457 |
| CP000512\_Aave\_1453 | 1600067 | 1600489 | False | COG3308 | 4e-25 | 93.0 | 6 | 127 |
| CP000512\_Aave\_1454 | 1600511 | 1601851 | True | COG1295 | 1e-34 | 89.0 | 13 | 282 |
| CP000512\_Aave\_1455 | 1601822 | 1603141 | False | COG2907 | 1e-19 | 95.0 | 9 | 434 |
| CP000512\_Aave\_1456 | 1603235 | 1604095 | False | COG1562 | 1e-60 | 97.0 | 7 | 288 |
| CP000512\_Aave\_1457 | 1604726 | 1607152 | False | COG0466 | 0.0 | 99.0 | 8 | 782 |
| CP000512\_Aave\_1458 | 1607287 | 1608552 | False | COG1219 | 0.0 | 99.0 | 1 | 404 |
| CP000512\_Aave\_1459 | 1608655 | 1609263 | False | COG0740 | 7e-85 | 95.0 | 3 | 193 |
| CP000512\_Aave\_1460 | 1609386 | 1610696 | False | COG0544 | 5e-94 | 99.0 | 1 | 439 |
| CP000512\_Aave\_1461 | 1610979 | 1614182 | False | COG0841 | 0.0 | 99.0 | 1 | 1008 |
| CP000512\_Aave\_1462 | 1614190 | 1615446 | False | COG0845 | 3e-22 | 95.0 | 11 | 367 |
| CP000512\_Aave\_1463 | 1615495 | 1616532 | True | COG1562 | 2e-32 | 92.0 | 20 | 285 |
| CP000512\_Aave\_1464 | 1616619 | 1617287 | False | - | - | - | - | - |
| CP000512\_Aave\_1465 | 1617808 | 1618290 | False | COG3157 | 3e-22 | 100.0 | 1 | 162 |
| CP000512\_Aave\_1466 | 1618859 | 1620844 | True | COG0515 | 4e-31 | 72.0 | 1 | 277 |
| CP000512\_Aave\_1467 | 1620951 | 1621451 | True | - | - | - | - | - |
| CP000512\_Aave\_1468 | 1621473 | 1623806 | True | COG3456 | 1e-08 | 58.0 | 22 | 271 |
| CP000512\_Aave\_1468 | 1621473 | 1623806 | True | COG3456 | 1e-24 | 69.0 | 127 | 425 |
| CP000512\_Aave\_1469 | 1623842 | 1624591 | True | COG0631 | 4e-33 | 92.0 | 8 | 249 |
| CP000512\_Aave\_1470 | 1624635 | 1625198 | True | COG3521 | 1e-22 | 79.0 | 4 | 130 |
| CP000512\_Aave\_1471 | 1625240 | 1626574 | True | COG3522 | 7e-135 | 100.0 | 1 | 446 |
| CP000512\_Aave\_1472 | 1626583 | 1627941 | True | COG3455 | 6e-58 | 97.0 | 5 | 259 |
| CP000512\_Aave\_1472 | 1626583 | 1627941 | True | COG1360 | 3e-29 | 63.0 | 90 | 244 |
| CP000512\_Aave\_1473 | 1627945 | 1631574 | True | COG3523 | 0.0 | 99.0 | 1 | 1187 |
| CP000512\_Aave\_1474 | 1631556 | 1632269 | True | COG3913 | 6e-18 | 93.0 | 2 | 214 |
| CP000512\_Aave\_1475 | 1632316 | 1633356 | True | COG3515 | 8e-23 | 100.0 | 1 | 346 |
| CP000512\_Aave\_1476 | 1633519 | 1634067 | True | COG3516 | 2e-56 | 94.0 | 3 | 162 |
| CP000512\_Aave\_1477 | 1634081 | 1635580 | True | COG3517 | 0.0 | 99.0 | 4 | 495 |
| CP000512\_Aave\_1478 | 1635633 | 1636490 | True | COG4455 | 1e-63 | 89.0 | 17 | 261 |
| CP000512\_Aave\_1479 | 1636507 | 1637028 | True | COG3518 | 2e-18 | 98.0 | 4 | 157 |
| CP000512\_Aave\_1480 | 1637101 | 1638975 | True | COG3519 | 1e-164 | 99.0 | 1 | 619 |
| CP000512\_Aave\_1481 | 1638972 | 1640138 | True | COG3520 | 7e-70 | 96.0 | 12 | 334 |
| CP000512\_Aave\_1482 | 1640211 | 1642988 | True | COG0542 | 5e-121 | 59.0 | 1 | 464 |
| CP000512\_Aave\_1482 | 1640211 | 1642988 | True | COG0542 | 1e-101 | 44.0 | 441 | 786 |
| CP000512\_Aave\_1483 | 1643375 | 1644013 | True | - | - | - | - | - |
| CP000512\_Aave\_1484 | 1644040 | 1644546 | True | COG1451 | 1e-18 | 73.0 | 56 | 220 |
| CP000512\_Aave\_1485 | 1644568 | 1646817 | False | COG1529 | 2e-152 | 100.0 | 1 | 731 |
| CP000512\_Aave\_1486 | 1646823 | 1647773 | False | COG1319 | 3e-60 | 98.0 | 1 | 280 |
