# Supplementary material for: Dissecting the bacterial type VI secretion system by a genome wide in silico analysis: what can be learned from available microbial genomic resources?
Source: BMC Genomics. 2009 Mar 12;10:104. doi: 10.1186/1471-2164-10-104 (PMC2660368; doi:10.1186/1471-2164-10-104)
Supplement: Additional file 7 — Detailed description of all identified T6SS gene clusters. Archive containing the detailed description of each identified T6SS locus as an HTML file. [file 1471-2164-10-104-S7.tgz › LociHTML/HTML/CP000514D.html]

Locus CP000514D on Marinobacter aquaeolei (strain DSM 11845 / ATCC 700491 / / VT8) / VT8) chromosome, complete sequence.

import namespace="svg" implementation="#AdobeSVG"?


# Locus CP000514D

# List of CDS in T6SS locus CP000514D

|  |  |  |  |  |  |  |  |  |
| --- | --- | --- | --- | --- | --- | --- | --- | --- |
| Name | from | to | direct | COG | e-value | COG cover | COG hit start | COG hit end |
| CP000514\_Maqu\_3716 | 4117842 | 4118897 | False | - | - | - | - | - |
| CP000514\_Maqu\_3717 | 4118887 | 4122924 | False | - | - | - | - | - |
| CP000514\_Maqu\_3718 | 4122964 | 4123506 | False | - | - | - | - | - |
| CP000514\_Maqu\_3719 | 4123522 | 4125693 | False | COG3501 | 1e-145 | 97.0 | 6 | 541 |
| CP000514\_Maqu\_3720 | 4125749 | 4129333 | False | COG3523 | 0.0 | 99.0 | 2 | 1188 |
| CP000514\_Maqu\_3721 | 4129370 | 4130818 | False | - | - | - | - | - |
| CP000514\_Maqu\_3722 | 4130830 | 4131465 | False | - | - | - | - | - |
| CP000514\_Maqu\_3723 | 4131462 | 4133027 | False | COG3829 | 2e-100 | 60.0 | 219 | 557 |
| CP000514\_Maqu\_3724 | 4133204 | 4135891 | False | COG0542 | 3e-129 | 59.0 | 1 | 469 |
| CP000514\_Maqu\_3724 | 4133204 | 4135891 | False | COG0542 | 8e-101 | 49.0 | 396 | 786 |
| CP000514\_Maqu\_3725 | 4135857 | 4136678 | False | COG3455 | 8e-62 | 96.0 | 8 | 260 |
| CP000514\_Maqu\_3726 | 4136682 | 4138016 | False | COG3522 | 2e-148 | 100.0 | 1 | 446 |
| CP000514\_Maqu\_3727 | 4138016 | 4138549 | False | COG3521 | 1e-28 | 97.0 | 5 | 159 |
| CP000514\_Maqu\_3728 | 4138546 | 4139856 | False | COG3456 | 3e-64 | 99.0 | 2 | 430 |
| CP000514\_Maqu\_3729 | 4139874 | 4140890 | False | COG3520 | 3e-78 | 94.0 | 15 | 332 |
| CP000514\_Maqu\_3730 | 4140854 | 4142626 | False | COG3519 | 6e-162 | 99.0 | 3 | 621 |
| CP000514\_Maqu\_3731 | 4142925 | 4143362 | False | COG3518 | 1e-19 | 89.0 | 14 | 153 |
| CP000514\_Maqu\_3732 | 4143366 | 4144847 | False | COG3517 | 0.0 | 99.0 | 1 | 492 |
| CP000514\_Maqu\_3733 | 4144909 | 4145406 | False | COG3516 | 6e-44 | 98.0 | 4 | 169 |
| CP000514\_Maqu\_3734 | 4145679 | 4145939 | True | COG4281 | 1e-16 | 94.0 | 1 | 82 |
| CP000514\_Maqu\_3735 | 4146064 | 4146378 | True | - | - | - | - | - |
| CP000514\_Maqu\_3736 | 4146401 | 4146847 | False | - | - | - | - | - |
| CP000514\_Maqu\_3737 | 4146964 | 4147479 | False | COG0782 | 4e-35 | 100.0 | 1 | 151 |
| CP000514\_Maqu\_3738 | 4147761 | 4148042 | True | - | - | - | - | - |
| CP000514\_Maqu\_3739 | 4148045 | 4149523 | False | COG0642 | 9e-39 | 96.0 | 5 | 328 |
| CP000514\_Maqu\_3740 | 4149510 | 4150190 | False | COG0745 | 2e-60 | 100.0 | 1 | 229 |
| CP000514\_Maqu\_3741 | 4150294 | 4151073 | False | COG3622 | 6e-100 | 99.0 | 1 | 258 |
| CP000514\_Maqu\_3742 | 4151094 | 4152005 | False | COG2084 | 2e-72 | 99.0 | 2 | 286 |
| CP000514\_Maqu\_3743 | 4152247 | 4153107 | False | COG0568 | 2e-62 | 79.0 | 63 | 335 |
| CP000514\_Maqu\_3744 | 4153300 | 4154175 | False | COG2177 | 2e-49 | 94.0 | 17 | 296 |
| CP000514\_Maqu\_3745 | 4154294 | 4154968 | False | COG2884 | 3e-86 | 96.0 | 1 | 216 |
| CP000514\_Maqu\_3746 | 4154996 | 4156213 | False | COG0552 | 7e-115 | 100.0 | 1 | 340 |
| CP000514\_Maqu\_3747 | 4156418 | 4157053 | True | COG0742 | 6e-52 | 99.0 | 1 | 186 |
| CP000514\_Maqu\_3748 | 4157091 | 4158269 | False | COG1398 | 3e-71 | 90.0 | 29 | 289 |
| CP000514\_Maqu\_3749 | 4158457 | 4159269 | True | COG0266 | 5e-97 | 100.0 | 1 | 273 |
| CP000514\_Maqu\_3750 | 4159446 | 4159778 | True | - | - | - | - | - |
| CP000514\_Maqu\_3751 | 4159865 | 4160554 | True | COG1011 | 8e-21 | 99.0 | 1 | 227 |
| CP000514\_Maqu\_3752 | 4160603 | 4161010 | True | COG1188 | 7e-23 | 95.0 | 5 | 99 |
| CP000514\_Maqu\_3753 | 4161098 | 4161952 | True | COG1281 | 6e-73 | 100.0 | 1 | 286 |
