# Supplementary material for: Dissecting the bacterial type VI secretion system by a genome wide in silico analysis: what can be learned from available microbial genomic resources?
Source: BMC Genomics. 2009 Mar 12;10:104. doi: 10.1186/1471-2164-10-104 (PMC2660368; doi:10.1186/1471-2164-10-104)
Supplement: Additional file 7 — Detailed description of all identified T6SS gene clusters. Archive containing the detailed description of each identified T6SS locus as an HTML file. [file 1471-2164-10-104-S7.tgz › LociHTML/HTML/CP000521A.html]

Locus CP000521A on Acinetobacter baumannii (strain ATCC 17978 / NCDC KC 755) chromosome, complete sequence.

import namespace="svg" implementation="#AdobeSVG"?


# Locus CP000521A

# List of CDS in T6SS locus CP000521A

|  |  |  |  |  |  |  |  |  |
| --- | --- | --- | --- | --- | --- | --- | --- | --- |
| Name | from | to | direct | COG | e-value | COG cover | COG hit start | COG hit end |
| CP000521\_A1S\_1283 | 1506250 | 1507920 | True | COG0154 | 2e-81 | 83.0 | 73 | 467 |
| CP000521\_A1S\_1284 | 1507992 | 1508939 | True | COG0715 | 3e-25 | 90.0 | 24 | 325 |
| CP000521\_A1S\_1285 | 1508991 | 1509707 | False | COG1116 | 1e-80 | 86.0 | 34 | 247 |
| CP000521\_A1S\_1286 | 1509872 | 1510297 | False | COG0600 | 8e-23 | 54.0 | 118 | 258 |
| CP000521\_A1S\_1287 | 1510374 | 1510751 | False | - | - | - | - | - |
| CP000521\_1510910..1511179 | 1510910 | 1511179 | False | - | - | - | - | - |
| CP000521\_A1S\_1288 | 1511966 | 1513246 | True | COG3501 | 1e-19 | 44.0 | 153 | 399 |
| CP000521\_A1S\_1289 | 1513051 | 1514238 | True | COG4253 | 2e-32 | 97.0 | 1 | 270 |
| CP000521\_A1S\_1289 | 1513051 | 1514238 | True | COG3501 | 6e-11 | 17.0 | 453 | 550 |
| CP000521\_A1S\_1290 | 1514426 | 1517251 | True | - | - | - | - | - |
| CP000521\_A1S\_1291 | 1517370 | 1518053 | False | COG1262 | 3e-20 | 58.0 | 65 | 248 |
| CP000521\_A1S\_1292 | 1519251 | 1519712 | True | - | - | - | - | - |
| CP000521\_1519550..1519666 | 1519550 | 1519666 | False | - | - | - | - | - |
| CP000521\_A1S\_1293 | 1519781 | 1519930 | True | COG3516 | 1e-07 | 25.0 | 18 | 60 |
| CP000521\_A1S\_1294 | 1519993 | 1520232 | True | COG3516 | 4e-16 | 45.0 | 86 | 162 |
| CP000521\_A1S\_1295 | 1520375 | 1521706 | True | COG3517 | 0.0 | 89.0 | 54 | 495 |
| CP000521\_A1S\_1296 | 1521756 | 1522259 | True | COG3157 | 3e-29 | 93.0 | 2 | 152 |
| CP000521\_A1S\_1297 | 1522474 | 1522815 | True | COG3518 | 2e-16 | 71.0 | 46 | 157 |
| CP000521\_A1S\_1298 | 1523066 | 1523623 | True | COG3519 | 2e-22 | 30.0 | 79 | 268 |
| CP000521\_A1S\_1299 | 1523946 | 1524641 | True | COG3519 | 4e-32 | 32.0 | 419 | 621 |
| CP000521\_A1S\_1300 | 1524707 | 1525603 | True | COG3520 | 1e-36 | 80.0 | 50 | 317 |
| CP000521\_A1S\_1301 | 1525747 | 1527012 | True | - | - | - | - | - |
| CP000521\_A1S\_1302 | 1527124 | 1530249 | True | COG3523 | 3e-156 | 82.0 | 40 | 1018 |
| CP000521\_A1S\_1303 | 1530300 | 1530866 | True | COG3523 | 3e-11 | 7.0 | 1053 | 1147 |
| CP000521\_A1S\_1304 | 1531036 | 1531863 | True | - | - | - | - | - |
| CP000521\_A1S\_1305 | 1532004 | 1532633 | True | COG2885 | 8e-29 | 69.0 | 57 | 188 |
| CP000521\_A1S\_1306 | 1532650 | 1532913 | True | COG4104 | 5e-10 | 89.0 | 10 | 97 |
| CP000521\_A1S\_1307 | 1533124 | 1535814 | True | COG0542 | 0.0 | 99.0 | 1 | 781 |
| CP000521\_A1S\_1308 | 1536114 | 1536935 | True | COG3515 | 3e-07 | 31.0 | 239 | 346 |
| CP000521\_A1S\_1309 | 1537150 | 1538316 | True | COG3522 | 3e-65 | 84.0 | 68 | 444 |
| CP000521\_A1S\_1310 | 1538493 | 1539140 | True | COG3455 | 2e-30 | 77.0 | 61 | 262 |
| CP000521\_1539360..1539755 | 1539360 | 1539755 | True | - | - | - | - | - |
| CP000521\_A1S\_1311 | 1539763 | 1540020 | True | - | - | - | - | - |
| CP000521\_A1S\_1312 | 1540332 | 1540715 | True | - | - | - | - | - |
| CP000521\_A1S\_1313 | 1540718 | 1541494 | False | COG0583 | 7e-22 | 72.0 | 25 | 239 |
| CP000521\_A1S\_1314 | 1541760 | 1542389 | True | COG0666 | 5e-08 | 42.0 | 72 | 171 |
| CP000521\_A1S\_1314 | 1541760 | 1542389 | True | COG0666 | 6e-11 | 58.0 | 76 | 212 |
| CP000521\_A1S\_1315 | 1542537 | 1542887 | True | COG0590 | 2e-16 | 43.0 | 51 | 116 |
| CP000521\_A1S\_1316 | 1542968 | 1544065 | True | COG2807 | 6e-64 | 91.0 | 28 | 389 |
