# Supplementary material for: Dissecting the bacterial type VI secretion system by a genome wide in silico analysis: what can be learned from available microbial genomic resources?
Source: BMC Genomics. 2009 Mar 12;10:104. doi: 10.1186/1471-2164-10-104 (PMC2660368; doi:10.1186/1471-2164-10-104)
Supplement: Additional file 7 — Detailed description of all identified T6SS gene clusters. Archive containing the detailed description of each identified T6SS locus as an HTML file. [file 1471-2164-10-104-S7.tgz › LociHTML/HTML/CP000544A.html]

Locus CP000544A on Halorhodospira halophila (strain DSM 244 / SL1) chromosome, complete sequence.

import namespace="svg" implementation="#AdobeSVG"?


# Locus CP000544A

# List of CDS in T6SS locus CP000544A

|  |  |  |  |  |  |  |  |  |
| --- | --- | --- | --- | --- | --- | --- | --- | --- |
| Name | from | to | direct | COG | e-value | COG cover | COG hit start | COG hit end |
| CP000544\_Hhal\_0150 | 173471 | 174319 | False | COG0613 | 4e-54 | 95.0 | 5 | 251 |
| CP000544\_Hhal\_0151 | 174316 | 176103 | False | COG5265 | 4e-172 | 99.0 | 1 | 496 |
| CP000544\_Hhal\_0152 | 176256 | 176696 | True | COG0346 | 9e-15 | 100.0 | 1 | 138 |
| CP000544\_Hhal\_0153 | 176702 | 178519 | True | COG1217 | 0.0 | 100.0 | 1 | 603 |
| CP000544\_Hhal\_0154 | 178516 | 180465 | False | COG3501 | 4e-16 | 80.0 | 25 | 466 |
| CP000544\_Hhal\_0155 | 180465 | 182525 | False | COG3501 | 2e-32 | 92.0 | 9 | 516 |
| CP000544\_Hhal\_0156 | 182522 | 183484 | False | COG3520 | 1e-11 | 88.0 | 29 | 323 |
| CP000544\_Hhal\_0157 | 183469 | 185166 | False | COG3519 | 2e-34 | 98.0 | 3 | 614 |
| CP000544\_Hhal\_0158 | 185144 | 185581 | False | COG3518 | 5e-08 | 64.0 | 14 | 115 |
| CP000544\_Hhal\_0159 | 185637 | 186122 | False | COG3157 | 8e-31 | 95.0 | 1 | 154 |
| CP000544\_Hhal\_0160 | 186157 | 187671 | False | COG3517 | 2e-168 | 98.0 | 2 | 491 |
| CP000544\_Hhal\_0161 | 187675 | 188277 | False | COG3516 | 5e-33 | 95.0 | 5 | 166 |
| CP000544\_Hhal\_0162 | 188287 | 189783 | False | - | - | - | - | - |
| CP000544\_Hhal\_0163 | 189780 | 193223 | False | COG3523 | 4e-94 | 99.0 | 5 | 1187 |
| CP000544\_Hhal\_0164 | 193220 | 193933 | False | COG3455 | 5e-16 | 75.0 | 41 | 238 |
| CP000544\_Hhal\_0165 | 193930 | 195282 | False | COG3522 | 8e-53 | 99.0 | 5 | 446 |
| CP000544\_Hhal\_0166 | 195284 | 195907 | False | COG3521 | 4e-15 | 66.0 | 31 | 135 |
| CP000544\_Hhal\_0167 | 196185 | 197807 | True | COG0840 | 2e-50 | 92.0 | 31 | 408 |
| CP000544\_Hhal\_0168 | 197937 | 198410 | False | COG1047 | 2e-37 | 89.0 | 1 | 155 |
| CP000544\_Hhal\_0169 | 198430 | 199803 | False | - | - | - | - | - |
| CP000544\_Hhal\_0170 | 199803 | 200174 | False | - | - | - | - | - |
| CP000544\_Hhal\_0171 | 200224 | 200898 | False | COG1011 | 3e-21 | 97.0 | 3 | 226 |
