# Supplementary material for: Dissecting the bacterial type VI secretion system by a genome wide in silico analysis: what can be learned from available microbial genomic resources?
Source: BMC Genomics. 2009 Mar 12;10:104. doi: 10.1186/1471-2164-10-104 (PMC2660368; doi:10.1186/1471-2164-10-104)
Supplement: Additional file 7 — Detailed description of all identified T6SS gene clusters. Archive containing the detailed description of each identified T6SS locus as an HTML file. [file 1471-2164-10-104-S7.tgz › LociHTML/HTML/CP000572D.html]

Locus CP000572D on Burkholderia pseudomallei (strain 1106a) chromosome I, complete sequence.

import namespace="svg" implementation="#AdobeSVG"?


# Locus CP000572D

# List of CDS in T6SS locus CP000572D

|  |  |  |  |  |  |  |  |  |
| --- | --- | --- | --- | --- | --- | --- | --- | --- |
| Name | from | to | direct | COG | e-value | COG cover | COG hit start | COG hit end |
| CP000572\_BURPS1106A\_3643 | 3541597 | 3543858 | True | COG2274 | 0.0 | 98.0 | 1 | 696 |
| CP000572\_BURPS1106A\_3644 | 3543888 | 3545303 | True | COG1538 | 1e-40 | 87.0 | 45 | 443 |
| CP000572\_BURPS1106A\_3645 | 3545288 | 3545437 | True | - | - | - | - | - |
| CP000572\_BURPS1106A\_3646 | 3545513 | 3546481 | False | - | - | - | - | - |
| CP000572\_BURPS1106A\_3647 | 3546493 | 3547458 | True | - | - | - | - | - |
| CP000572\_BURPS1106A\_3648 | 3547487 | 3547624 | True | - | - | - | - | - |
| CP000572\_BURPS1106A\_3649 | 3547609 | 3547722 | True | - | - | - | - | - |
| CP000572\_BURPS1106A\_3650 | 3547730 | 3551623 | True | COG3523 | 0.0 | 100.0 | 1 | 1188 |
| CP000572\_BURPS1106A\_3651 | 3551620 | 3552609 | True | COG3913 | 2e-36 | 93.0 | 5 | 216 |
| CP000572\_BURPS1106A\_3652 | 3552614 | 3553546 | True | COG2885 | 1e-25 | 84.0 | 27 | 186 |
| CP000572\_BURPS1106A\_3653 | 3553745 | 3554866 | False | COG3515 | 4e-32 | 98.0 | 7 | 346 |
| CP000572\_BURPS1106A\_3654 | 3554956 | 3557625 | False | COG0542 | 0.0 | 99.0 | 1 | 784 |
| CP000572\_BURPS1106A\_3655 | 3557659 | 3558759 | False | COG3520 | 8e-61 | 99.0 | 1 | 332 |
| CP000572\_BURPS1106A\_3656 | 3558723 | 3560561 | False | COG3519 | 1e-145 | 99.0 | 2 | 620 |
| CP000572\_BURPS1106A\_3657 | 3560641 | 3561123 | False | COG3518 | 1e-33 | 98.0 | 4 | 157 |
| CP000572\_BURPS1106A\_3658 | 3561181 | 3561684 | False | COG3157 | 2e-34 | 97.0 | 5 | 162 |
| CP000572\_BURPS1106A\_3659 | 3561757 | 3563247 | False | COG3517 | 0.0 | 99.0 | 2 | 495 |
| CP000572\_BURPS1106A\_3660 | 3563264 | 3563782 | False | COG3516 | 2e-47 | 99.0 | 2 | 169 |
| CP000572\_BURPS1106A\_3661 | 3563819 | 3564454 | False | - | - | - | - | - |
| CP000572\_BURPS1106A\_3662 | 3564616 | 3564753 | False | - | - | - | - | - |
| CP000572\_BURPS1106A\_3663 | 3564876 | 3565478 | True | COG3521 | 2e-26 | 89.0 | 5 | 147 |
| CP000572\_BURPS1106A\_3664 | 3565587 | 3566933 | True | COG3522 | 3e-112 | 100.0 | 1 | 446 |
| CP000572\_BURPS1106A\_3665 | 3566930 | 3567715 | True | COG3455 | 3e-47 | 95.0 | 14 | 262 |
| CP000572\_BURPS1106A\_3666 | 3567817 | 3568182 | True | - | - | - | - | - |
| CP000572\_BURPS1106A\_3667 | 3569271 | 3569687 | False | COG0863 | 4e-10 | 52.0 | 111 | 268 |
| CP000572\_BURPS1106A\_3668 | 3569772 | 3569969 | False | COG4385 | 3e-08 | 24.0 | 71 | 121 |
| CP000572\_BURPS1106A\_3669 | 3570176 | 3570502 | False | COG3948 | 3e-20 | 34.0 | 202 | 306 |
| CP000572\_BURPS1106A\_3670 | 3570789 | 3571949 | False | COG3464 | 3e-68 | 90.0 | 41 | 402 |
