# Supplementary material for: Dissecting the bacterial type VI secretion system by a genome wide in silico analysis: what can be learned from available microbial genomic resources?
Source: BMC Genomics. 2009 Mar 12;10:104. doi: 10.1186/1471-2164-10-104 (PMC2660368; doi:10.1186/1471-2164-10-104)
Supplement: Additional file 7 — Detailed description of all identified T6SS gene clusters. Archive containing the detailed description of each identified T6SS locus as an HTML file. [file 1471-2164-10-104-S7.tgz › LociHTML/HTML/CP000573E.html]

Locus CP000573E on Burkholderia pseudomallei (strain 1106a) chromosome II, complete sequence.

import namespace="svg" implementation="#AdobeSVG"?


# Locus CP000573E

# List of CDS in T6SS locus CP000573E

|  |  |  |  |  |  |  |  |  |
| --- | --- | --- | --- | --- | --- | --- | --- | --- |
| Name | from | to | direct | COG | e-value | COG cover | COG hit start | COG hit end |
| CP000573\_BURPS1106A\_A0126 | 110188 | 110697 | True | COG3539 | 2e-14 | 94.0 | 11 | 184 |
| CP000573\_BURPS1106A\_A0127 | 110762 | 111499 | True | COG3121 | 4e-59 | 97.0 | 6 | 234 |
| CP000573\_BURPS1106A\_A0128 | 111612 | 114356 | True | COG3188 | 0.0 | 96.0 | 12 | 818 |
| CP000573\_BURPS1106A\_A0129 | 114349 | 114921 | True | COG3539 | 5e-16 | 96.0 | 6 | 182 |
| CP000573\_BURPS1106A\_A0130 | 114953 | 115642 | True | COG3455 | 3e-10 | 68.0 | 68 | 247 |
| CP000573\_BURPS1106A\_A0131 | 115693 | 117321 | True | COG2885 | 2e-26 | 79.0 | 40 | 190 |
| CP000573\_BURPS1106A\_A0132 | 117284 | 117397 | True | - | - | - | - | - |
| CP000573\_BURPS1106A\_A0133 | 117711 | 118250 | True | COG3516 | 4e-59 | 99.0 | 2 | 169 |
| CP000573\_BURPS1106A\_A0134 | 118284 | 119783 | True | COG3517 | 0.0 | 100.0 | 1 | 495 |
| CP000573\_BURPS1106A\_A0135 | 119787 | 119939 | True | - | - | - | - | - |
| CP000573\_BURPS1106A\_A0136 | 119983 | 120465 | True | COG3157 | 1e-34 | 99.0 | 1 | 161 |
| CP000573\_BURPS1106A\_A0137 | 120593 | 121135 | True | COG3521 | 2e-26 | 86.0 | 6 | 143 |
| CP000573\_BURPS1106A\_A0138 | 121132 | 122490 | True | COG3522 | 4e-135 | 99.0 | 1 | 445 |
| CP000573\_BURPS1106A\_A0139 | 122487 | 123788 | True | COG3455 | 8e-49 | 93.0 | 15 | 260 |
| CP000573\_BURPS1106A\_A0139 | 122487 | 123788 | True | COG1360 | 9e-28 | 56.0 | 103 | 241 |
| CP000573\_BURPS1106A\_A0140 | 123803 | 127711 | True | COG3523 | 0.0 | 99.0 | 2 | 1184 |
| CP000573\_BURPS1106A\_A0141 | 127742 | 127864 | True | - | - | - | - | - |
| CP000573\_BURPS1106A\_A0142 | 127901 | 128470 | True | - | - | - | - | - |
| CP000573\_BURPS1106A\_A0143 | 128568 | 131258 | True | COG3501 | 1e-131 | 90.0 | 24 | 523 |
| CP000573\_BURPS1106A\_A0143 | 128568 | 131258 | True | COG3889 | 2e-12 | 17.0 | 694 | 845 |
| CP000573\_BURPS1106A\_A0144 | 131327 | 131602 | True | COG4104 | 4e-07 | 90.0 | 9 | 97 |
| CP000573\_BURPS1106A\_A0145 | 131615 | 135067 | True | COG3209 | 4e-21 | 84.0 | 20 | 693 |
| CP000573\_BURPS1106A\_A0146 | 135974 | 136150 | True | - | - | - | - | - |
| CP000573\_BURPS1106A\_A0147 | 136351 | 137379 | False | - | - | - | - | - |
| CP000573\_BURPS1106A\_A0148 | 137434 | 138504 | False | COG3515 | 8e-21 | 97.0 | 1 | 338 |
| CP000573\_BURPS1106A\_A0149 | 138523 | 139572 | False | COG3520 | 9e-82 | 97.0 | 9 | 334 |
| CP000573\_BURPS1106A\_A0150 | 139569 | 141449 | False | COG3519 | 0.0 | 100.0 | 1 | 621 |
| CP000573\_BURPS1106A\_A0151 | 141451 | 141969 | False | COG3518 | 8e-21 | 95.0 | 8 | 157 |
| CP000573\_BURPS1106A\_A0152 | 141956 | 142762 | False | COG4455 | 8e-58 | 95.0 | 12 | 273 |
| CP000573\_BURPS1106A\_A0153 | 142794 | 143849 | False | - | - | - | - | - |
| CP000573\_BURPS1106A\_A0154 | 143908 | 144036 | False | - | - | - | - | - |
| CP000573\_BURPS1106A\_A0155 | 144139 | 144261 | False | - | - | - | - | - |
| CP000573\_BURPS1106A\_A0156 | 144237 | 146924 | True | COG0542 | 0.0 | 98.0 | 1 | 776 |
| CP000573\_BURPS1106A\_A0157 | 146998 | 147135 | False | - | - | - | - | - |
| CP000573\_BURPS1106A\_A0158 | 147187 | 147306 | True | - | - | - | - | - |
| CP000573\_BURPS1106A\_A0159 | 147315 | 150413 | False | COG2204 | 7e-18 | 34.0 | 6 | 165 |
| CP000573\_BURPS1106A\_A0159 | 147315 | 150413 | False | COG0642 | 8e-41 | 82.0 | 57 | 333 |
| CP000573\_BURPS1106A\_A0160 | 151144 | 151260 | False | - | - | - | - | - |
