# Supplementary material for: Dissecting the bacterial type VI secretion system by a genome wide in silico analysis: what can be learned from available microbial genomic resources?
Source: BMC Genomics. 2009 Mar 12;10:104. doi: 10.1186/1471-2164-10-104 (PMC2660368; doi:10.1186/1471-2164-10-104)
Supplement: Additional file 7 — Detailed description of all identified T6SS gene clusters. Archive containing the detailed description of each identified T6SS locus as an HTML file. [file 1471-2164-10-104-S7.tgz › LociHTML/HTML/CP000573F.html]

Locus CP000573F on Burkholderia pseudomallei (strain 1106a) chromosome II, complete sequence.

import namespace="svg" implementation="#AdobeSVG"?


# Locus CP000573F

# List of CDS in T6SS locus CP000573F

|  |  |  |  |  |  |  |  |  |
| --- | --- | --- | --- | --- | --- | --- | --- | --- |
| Name | from | to | direct | COG | e-value | COG cover | COG hit start | COG hit end |
| CP000573\_BURPS1106A\_A0231 | 214891 | 216759 | True | COG0367 | 4e-105 | 95.0 | 1 | 520 |
| CP000573\_BURPS1106A\_A0232 | 216788 | 217759 | True | COG0604 | 5e-63 | 100.0 | 1 | 326 |
| CP000573\_BURPS1106A\_A0233 | 217814 | 218740 | True | COG3384 | 1e-15 | 78.0 | 31 | 240 |
| CP000573\_BURPS1106A\_A0234 | 218984 | 220330 | False | COG0583 | 4e-29 | 97.0 | 2 | 292 |
| CP000573\_BURPS1106A\_A0235 | 220915 | 221148 | False | - | - | - | - | - |
| CP000573\_BURPS1106A\_A0236 | 221168 | 225244 | False | COG3523 | 0.0 | 99.0 | 2 | 1184 |
| CP000573\_BURPS1106A\_A0237 | 225273 | 226538 | False | COG3455 | 6e-48 | 95.0 | 13 | 262 |
| CP000573\_BURPS1106A\_A0237 | 225273 | 226538 | False | COG1360 | 1e-26 | 67.0 | 79 | 242 |
| CP000573\_BURPS1106A\_A0238 | 226627 | 227973 | False | COG3522 | 7e-128 | 100.0 | 1 | 446 |
| CP000573\_BURPS1106A\_A0239 | 227995 | 228498 | False | COG3521 | 4e-29 | 93.0 | 8 | 155 |
| CP000573\_BURPS1106A\_A0241 | 228511 | 228627 | True | - | - | - | - | - |
| CP000573\_BURPS1106A\_A0240 | 228605 | 229090 | False | COG3157 | 4e-37 | 94.0 | 1 | 153 |
| CP000573\_BURPS1106A\_A0242 | 229207 | 230706 | False | COG3517 | 0.0 | 100.0 | 1 | 495 |
| CP000573\_BURPS1106A\_A0243 | 230741 | 231322 | False | COG3516 | 5e-57 | 95.0 | 2 | 163 |
| CP000573\_BURPS1106A\_A0244 | 231358 | 234297 | False | COG0542 | 9e-123 | 57.0 | 1 | 452 |
| CP000573\_BURPS1106A\_A0244 | 231358 | 234297 | False | COG0542 | 3e-101 | 39.0 | 453 | 761 |
| CP000573\_BURPS1106A\_A0245 | 234829 | 235551 | True | - | - | - | - | - |
| CP000573\_BURPS1106A\_A0246 | 235548 | 236513 | True | COG4455 | 1e-52 | 95.0 | 14 | 273 |
| CP000573\_BURPS1106A\_A0247 | 236500 | 237081 | True | COG3518 | 6e-19 | 92.0 | 6 | 151 |
| CP000573\_BURPS1106A\_A0248 | 237112 | 239001 | True | COG3519 | 0.0 | 99.0 | 1 | 619 |
| CP000573\_BURPS1106A\_A0249 | 239001 | 240086 | True | COG3520 | 1e-73 | 98.0 | 7 | 335 |
| CP000573\_BURPS1106A\_A0250 | 240149 | 241198 | True | COG3515 | 1e-18 | 95.0 | 8 | 336 |
| CP000573\_BURPS1106A\_A0251 | 241270 | 243597 | True | COG3501 | 2e-131 | 94.0 | 10 | 531 |
| CP000573\_BURPS1106A\_A0252 | 243629 | 246106 | True | COG1357 | 5e-23 | 93.0 | 1 | 222 |
| CP000573\_BURPS1106A\_A0252 | 243629 | 246106 | True | COG5351 | 1e-21 | 49.0 | 100 | 281 |
| CP000573\_BURPS1106A\_A0253 | 246103 | 247185 | True | COG1357 | 7e-21 | 82.0 | 37 | 233 |
| CP000573\_BURPS1106A\_A0254 | 247179 | 247370 | True | - | - | - | - | - |
| CP000573\_BURPS1106A\_A0255 | 247367 | 248026 | True | - | - | - | - | - |
| CP000573\_BURPS1106A\_A0256 | 248067 | 248447 | True | - | - | - | - | - |
| CP000573\_BURPS1106A\_A0257 | 248514 | 248690 | True | - | - | - | - | - |
