# Supplementary material for: Dissecting the bacterial type VI secretion system by a genome wide in silico analysis: what can be learned from available microbial genomic resources?
Source: BMC Genomics. 2009 Mar 12;10:104. doi: 10.1186/1471-2164-10-104 (PMC2660368; doi:10.1186/1471-2164-10-104)
Supplement: Additional file 7 — Detailed description of all identified T6SS gene clusters. Archive containing the detailed description of each identified T6SS locus as an HTML file. [file 1471-2164-10-104-S7.tgz › LociHTML/HTML/CP000573G.html]

Locus CP000573G on Burkholderia pseudomallei (strain 1106a) chromosome II, complete sequence.

import namespace="svg" implementation="#AdobeSVG"?


# Locus CP000573G

# List of CDS in T6SS locus CP000573G

|  |  |  |  |  |  |  |  |  |
| --- | --- | --- | --- | --- | --- | --- | --- | --- |
| Name | from | to | direct | COG | e-value | COG cover | COG hit start | COG hit end |
| CP000573\_BURPS1106A\_A0694 | 678103 | 678873 | False | - | - | - | - | - |
| CP000573\_BURPS1106A\_A0695 | 678873 | 679562 | False | COG0558 | 5e-13 | 96.0 | 2 | 186 |
| CP000573\_BURPS1106A\_A0696 | 679927 | 681441 | True | COG0427 | 3e-161 | 100.0 | 1 | 501 |
| CP000573\_BURPS1106A\_A0697 | 681538 | 681666 | True | - | - | - | - | - |
| CP000573\_BURPS1106A\_A0698 | 682476 | 682601 | True | - | - | - | - | - |
| CP000573\_BURPS1106A\_A0699 | 683162 | 684100 | True | COG3515 | 8e-11 | 83.0 | 54 | 343 |
| CP000573\_BURPS1106A\_A0700 | 684134 | 684682 | True | COG3516 | 6e-47 | 97.0 | 5 | 169 |
| CP000573\_BURPS1106A\_A0701 | 684685 | 686190 | True | COG3517 | 0.0 | 99.0 | 1 | 494 |
| CP000573\_BURPS1106A\_A0702 | 686334 | 686861 | True | COG3157 | 2e-27 | 98.0 | 1 | 160 |
| CP000573\_BURPS1106A\_A0703 | 686941 | 687372 | True | - | - | - | - | - |
| CP000573\_BURPS1106A\_A0704 | 687386 | 689248 | True | COG3519 | 2e-87 | 96.0 | 3 | 604 |
| CP000573\_BURPS1106A\_A0705 | 689245 | 690234 | True | COG3520 | 1e-33 | 93.0 | 15 | 328 |
| CP000573\_BURPS1106A\_A0706 | 690237 | 693107 | True | COG0542 | 0.0 | 97.0 | 2 | 766 |
| CP000573\_BURPS1106A\_A0707 | 693098 | 695389 | True | COG3501 | 1e-135 | 97.0 | 10 | 544 |
| CP000573\_BURPS1106A\_A0708 | 695412 | 695543 | False | - | - | - | - | - |
| CP000573\_BURPS1106A\_A0709 | 695555 | 697843 | True | COG3501 | 3e-135 | 96.0 | 10 | 537 |
| CP000573\_BURPS1106A\_A0710 | 697847 | 700063 | True | COG1357 | 4e-08 | 61.0 | 58 | 204 |
| CP000573\_BURPS1106A\_A0711 | 700063 | 701133 | True | COG1357 | 8e-09 | 66.0 | 32 | 190 |
| CP000573\_BURPS1106A\_A0712 | 701136 | 701852 | True | - | - | - | - | - |
| CP000573\_BURPS1106A\_A0713 | 701895 | 702284 | True | - | - | - | - | - |
| CP000573\_BURPS1106A\_A0714 | 702290 | 702883 | True | COG3521 | 3e-08 | 62.0 | 7 | 106 |
| CP000573\_BURPS1106A\_A0715 | 702880 | 704241 | True | COG3522 | 5e-91 | 98.0 | 7 | 446 |
| CP000573\_BURPS1106A\_A0716 | 704324 | 705982 | True | COG3455 | 2e-25 | 98.0 | 1 | 257 |
| CP000573\_BURPS1106A\_A0716 | 704324 | 705982 | True | COG1360 | 1e-18 | 50.0 | 123 | 244 |
| CP000573\_BURPS1106A\_A0717 | 705979 | 709482 | True | COG3523 | 2e-139 | 98.0 | 17 | 1185 |
| CP000573\_BURPS1106A\_A0718 | 709541 | 709900 | True | - | - | - | - | - |
| CP000573\_BURPS1106A\_A0719 | 709923 | 710348 | True | - | - | - | - | - |
| CP000573\_BURPS1106A\_A0720 | 710548 | 710670 | False | - | - | - | - | - |
| CP000573\_BURPS1106A\_A0721 | 710624 | 710944 | True | - | - | - | - | - |
| CP000573\_BURPS1106A\_A0722 | 710981 | 711121 | True | - | - | - | - | - |
| CP000573\_BURPS1106A\_A0723 | 711495 | 712394 | True | - | - | - | - | - |
| CP000573\_BURPS1106A\_A0724 | 712436 | 712738 | False | - | - | - | - | - |
| CP000573\_BURPS1106A\_A0725 | 712628 | 713947 | True | COG1819 | 1e-39 | 98.0 | 1 | 399 |
| CP000573\_BURPS1106A\_A0726 | 713944 | 715527 | True | COG2814 | 3e-15 | 50.0 | 1 | 198 |
