# Supplementary material for: Dissecting the bacterial type VI secretion system by a genome wide in silico analysis: what can be learned from available microbial genomic resources?
Source: BMC Genomics. 2009 Mar 12;10:104. doi: 10.1186/1471-2164-10-104 (PMC2660368; doi:10.1186/1471-2164-10-104)
Supplement: Additional file 7 — Detailed description of all identified T6SS gene clusters. Archive containing the detailed description of each identified T6SS locus as an HTML file. [file 1471-2164-10-104-S7.tgz › LociHTML/HTML/CP000573H.html]

Locus CP000573H on Burkholderia pseudomallei (strain 1106a) chromosome II, complete sequence.

import namespace="svg" implementation="#AdobeSVG"?


# Locus CP000573H

# List of CDS in T6SS locus CP000573H

|  |  |  |  |  |  |  |  |  |
| --- | --- | --- | --- | --- | --- | --- | --- | --- |
| Name | from | to | direct | COG | e-value | COG cover | COG hit start | COG hit end |
| CP000573\_BURPS1106A\_A2021 | 1987602 | 1987979 | True | - | - | - | - | - |
| CP000573\_BURPS1106A\_A2022 | 1988222 | 1990093 | False | COG3515 | 2e-08 | 29.0 | 22 | 124 |
| CP000573\_BURPS1106A\_A2023 | 1990084 | 1990821 | False | COG0745 | 7e-52 | 99.0 | 3 | 229 |
| CP000573\_BURPS1106A\_A2024 | 1990818 | 1992662 | False | COG0642 | 1e-25 | 98.0 | 1 | 330 |
| CP000573\_BURPS1106A\_A2024 | 1990818 | 1992662 | False | COG3447 | 2e-07 | 86.0 | 41 | 306 |
| CP000573\_BURPS1106A\_A2025 | 1992922 | 1993416 | True | COG3516 | 2e-38 | 94.0 | 8 | 166 |
| CP000573\_BURPS1106A\_A2026 | 1993440 | 1994939 | True | COG3517 | 0.0 | 97.0 | 10 | 492 |
| CP000573\_BURPS1106A\_A2027 | 1995159 | 1995668 | True | COG3157 | 2e-12 | 95.0 | 1 | 154 |
| CP000573\_BURPS1106A\_A2028 | 1995661 | 1996122 | True | COG3518 | 9e-10 | 87.0 | 4 | 140 |
| CP000573\_BURPS1106A\_A2029 | 1996159 | 1997901 | True | COG3519 | 4e-102 | 99.0 | 7 | 621 |
| CP000573\_BURPS1106A\_A2030 | 1997865 | 1998911 | True | COG3520 | 2e-44 | 95.0 | 15 | 335 |
| CP000573\_BURPS1106A\_A2031 | 1998898 | 2001954 | True | COG0542 | 3e-124 | 54.0 | 1 | 427 |
| CP000573\_BURPS1106A\_A2031 | 1998898 | 2001954 | True | COG0542 | 1e-100 | 43.0 | 423 | 760 |
| CP000573\_BURPS1106A\_A2032 | 2001981 | 2005004 | True | COG3501 | 4e-82 | 83.0 | 4 | 463 |
| CP000573\_BURPS1106A\_A2033 | 2005030 | 2007663 | True | COG1357 | 6e-21 | 93.0 | 17 | 238 |
| CP000573\_BURPS1106A\_A2033 | 2005030 | 2007663 | True | COG1357 | 3e-09 | 47.0 | 125 | 237 |
| CP000573\_BURPS1106A\_A2033 | 2005030 | 2007663 | True | COG5351 | 6e-07 | 23.0 | 207 | 291 |
| CP000573\_BURPS1106A\_A2034 | 2007681 | 2008745 | True | COG1357 | 7e-16 | 95.0 | 1 | 227 |
| CP000573\_BURPS1106A\_A2034 | 2007681 | 2008745 | True | COG1357 | 1e-08 | 54.0 | 93 | 221 |
| CP000573\_BURPS1106A\_A2035 | 2008859 | 2009497 | True | - | - | - | - | - |
| CP000573\_BURPS1106A\_A2036 | 2009526 | 2009918 | True | - | - | - | - | - |
| CP000573\_BURPS1106A\_A2037 | 2009928 | 2010725 | True | - | - | - | - | - |
| CP000573\_BURPS1106A\_A2038 | 2010722 | 2012155 | True | COG3522 | 1e-42 | 98.0 | 5 | 445 |
| CP000573\_BURPS1106A\_A2039 | 2012152 | 2012817 | True | COG3455 | 3e-20 | 82.0 | 41 | 256 |
| CP000573\_BURPS1106A\_A2040 | 2012829 | 2016833 | True | COG3523 | 5e-44 | 37.0 | 18 | 467 |
| CP000573\_BURPS1106A\_A2041 | 2016849 | 2016998 | True | - | - | - | - | - |
| CP000573\_BURPS1106A\_A2042 | 2017002 | 2017187 | False | - | - | - | - | - |
| CP000573\_BURPS1106A\_A2043 | 2017683 | 2017853 | False | - | - | - | - | - |
