# Supplementary material for: Dissecting the bacterial type VI secretion system by a genome wide in silico analysis: what can be learned from available microbial genomic resources?
Source: BMC Genomics. 2009 Mar 12;10:104. doi: 10.1186/1471-2164-10-104 (PMC2660368; doi:10.1186/1471-2164-10-104)
Supplement: Additional file 7 — Detailed description of all identified T6SS gene clusters. Archive containing the detailed description of each identified T6SS locus as an HTML file. [file 1471-2164-10-104-S7.tgz › LociHTML/HTML/CP000573I.html]

Locus CP000573I on Burkholderia pseudomallei (strain 1106a) chromosome II, complete sequence.

import namespace="svg" implementation="#AdobeSVG"?


# Locus CP000573I

# List of CDS in T6SS locus CP000573I

|  |  |  |  |  |  |  |  |  |
| --- | --- | --- | --- | --- | --- | --- | --- | --- |
| Name | from | to | direct | COG | e-value | COG cover | COG hit start | COG hit end |
| CP000573\_BURPS1106A\_A2822 | 2762372 | 2763430 | False | COG1609 | 8e-78 | 100.0 | 1 | 333 |
| CP000573\_BURPS1106A\_A2823 | 2763834 | 2764091 | True | - | - | - | - | - |
| CP000573\_BURPS1106A\_A2824 | 2764868 | 2765608 | True | - | - | - | - | - |
| CP000573\_BURPS1106A\_A2825 | 2766040 | 2766153 | False | - | - | - | - | - |
| CP000573\_BURPS1106A\_A2826 | 2766340 | 2767599 | False | COG3677 | 4e-08 | 83.0 | 22 | 129 |
| CP000573\_BURPS1106A\_A2826 | 2766340 | 2767599 | False | COG3209 | 3e-12 | 29.0 | 49 | 283 |
| CP000573\_BURPS1106A\_A2827 | 2767559 | 2768269 | False | - | - | - | - | - |
| CP000573\_BURPS1106A\_A2828 | 2768285 | 2770489 | False | COG3501 | 3e-143 | 96.0 | 23 | 550 |
| CP000573\_BURPS1106A\_A2829 | 2770486 | 2773152 | False | COG0542 | 0.0 | 100.0 | 1 | 786 |
| CP000573\_BURPS1106A\_A2830 | 2773165 | 2774610 | False | COG3520 | 5e-56 | 94.0 | 3 | 318 |
| CP000573\_BURPS1106A\_A2831 | 2774607 | 2776478 | False | COG3519 | 2e-168 | 100.0 | 1 | 621 |
| CP000573\_BURPS1106A\_A2832 | 2776483 | 2777019 | False | COG3518 | 1e-17 | 94.0 | 1 | 149 |
| CP000573\_BURPS1106A\_A2833 | 2777050 | 2777541 | False | COG3157 | 3e-19 | 98.0 | 1 | 159 |
| CP000573\_BURPS1106A\_A2834 | 2777601 | 2779109 | False | COG3517 | 0.0 | 99.0 | 2 | 495 |
| CP000573\_BURPS1106A\_A2835 | 2779102 | 2779680 | False | COG3516 | 2e-53 | 98.0 | 2 | 167 |
| CP000573\_BURPS1106A\_A2836 | 2779743 | 2780822 | False | COG3515 | 5e-20 | 97.0 | 6 | 341 |
| CP000573\_BURPS1106A\_A2837 | 2780875 | 2783460 | False | COG0515 | 2e-31 | 94.0 | 1 | 361 |
| CP000573\_BURPS1106A\_A2837 | 2780875 | 2783460 | False | COG3846 | 2e-07 | 35.0 | 294 | 452 |
| CP000573\_BURPS1106A\_A2839 | 2783459 | 2783686 | True | - | - | - | - | - |
| CP000573\_BURPS1106A\_A2838 | 2783671 | 2784654 | False | COG3913 | 5e-12 | 83.0 | 2 | 190 |
| CP000573\_BURPS1106A\_A2840 | 2784636 | 2788265 | False | COG3523 | 0.0 | 99.0 | 7 | 1185 |
| CP000573\_BURPS1106A\_A2841 | 2788268 | 2789584 | False | COG3455 | 2e-59 | 93.0 | 13 | 258 |
| CP000573\_BURPS1106A\_A2841 | 2788268 | 2789584 | False | COG1360 | 1e-27 | 56.0 | 103 | 240 |
| CP000573\_BURPS1106A\_A2842 | 2789600 | 2791003 | False | COG3522 | 4e-127 | 99.0 | 3 | 446 |
| CP000573\_BURPS1106A\_A2843 | 2791000 | 2791518 | False | COG3521 | 1e-25 | 99.0 | 1 | 158 |
| CP000573\_BURPS1106A\_A2844 | 2791714 | 2791974 | False | - | - | - | - | - |
| CP000573\_BURPS1106A\_A2845 | 2792200 | 2792559 | True | - | - | - | - | - |
| CP000573\_BURPS1106A\_A2846 | 2792614 | 2794050 | True | COG3456 | 1e-31 | 93.0 | 22 | 425 |
| CP000573\_BURPS1106A\_A2847 | 2794094 | 2795998 | False | - | - | - | - | - |
| CP000573\_BURPS1106A\_A2848 | 2796017 | 2796202 | True | - | - | - | - | - |
| CP000573\_BURPS1106A\_A2849 | 2796468 | 2797712 | True | COG0654 | 2e-35 | 87.0 | 2 | 340 |
| CP000573\_BURPS1106A\_A2850 | 2797917 | 2798042 | False | - | - | - | - | - |
| CP000573\_BURPS1106A\_A2851 | 2798022 | 2798339 | True | - | - | - | - | - |
| CP000573\_BURPS1106A\_A2852 | 2798388 | 2798564 | True | - | - | - | - | - |
