# Supplementary material for: Dissecting the bacterial type VI secretion system by a genome wide in silico analysis: what can be learned from available microbial genomic resources?
Source: BMC Genomics. 2009 Mar 12;10:104. doi: 10.1186/1471-2164-10-104 (PMC2660368; doi:10.1186/1471-2164-10-104)
Supplement: Additional file 7 — Detailed description of all identified T6SS gene clusters. Archive containing the detailed description of each identified T6SS locus as an HTML file. [file 1471-2164-10-104-S7.tgz › LociHTML/HTML/CP000578A.html]

Locus CP000578A on Rhodobacter sphaeroides (strain ATCC 17029 / ATH 2.4.9) chromosome 2, complete sequence.

import namespace="svg" implementation="#AdobeSVG"?


# Locus CP000578A

# List of CDS in T6SS locus CP000578A

|  |  |  |  |  |  |  |  |  |
| --- | --- | --- | --- | --- | --- | --- | --- | --- |
| Name | from | to | direct | COG | e-value | COG cover | COG hit start | COG hit end |
| CP000578\_Rsph17029\_3111 | 124979 | 126268 | True | - | - | - | - | - |
| CP000578\_Rsph17029\_3112 | 126270 | 127292 | True | COG5351 | 2e-68 | 91.0 | 1 | 335 |
| CP000578\_Rsph17029\_3113 | 127285 | 128376 | True | COG0304 | 1e-07 | 91.0 | 7 | 381 |
| CP000578\_Rsph17029\_3114 | 128373 | 129374 | True | - | - | - | - | - |
| CP000578\_Rsph17029\_3115 | 129371 | 130150 | True | - | - | - | - | - |
| CP000578\_Rsph17029\_3116 | 130173 | 130859 | True | - | - | - | - | - |
| CP000578\_Rsph17029\_3117 | 130856 | 131254 | False | - | - | - | - | - |
| CP000578\_Rsph17029\_3118 | 131247 | 133916 | False | COG0542 | 0.0 | 100.0 | 1 | 786 |
| CP000578\_Rsph17029\_3119 | 134030 | 136036 | True | COG0515 | 2e-31 | 76.0 | 2 | 294 |
| CP000578\_Rsph17029\_3120 | 136135 | 137208 | True | COG3515 | 3e-16 | 99.0 | 1 | 345 |
| CP000578\_Rsph17029\_3121 | 137227 | 137757 | True | COG3516 | 6e-46 | 99.0 | 2 | 169 |
| CP000578\_Rsph17029\_3122 | 137760 | 139271 | True | COG3517 | 0.0 | 99.0 | 2 | 495 |
| CP000578\_Rsph17029\_3123 | 139323 | 139814 | True | COG3157 | 4e-22 | 94.0 | 1 | 153 |
| CP000578\_Rsph17029\_3124 | 139847 | 140554 | True | COG3518 | 5e-15 | 84.0 | 25 | 157 |
| CP000578\_Rsph17029\_3125 | 140554 | 142419 | True | COG3519 | 6e-132 | 100.0 | 1 | 621 |
| CP000578\_Rsph17029\_3126 | 142383 | 143381 | True | COG3520 | 3e-43 | 89.0 | 23 | 323 |
| CP000578\_Rsph17029\_3127 | 143375 | 144604 | True | COG3456 | 4e-60 | 98.0 | 5 | 426 |
| CP000578\_Rsph17029\_3128 | 144597 | 145049 | True | COG3521 | 1e-21 | 98.0 | 1 | 156 |
| CP000578\_Rsph17029\_3129 | 145058 | 146386 | True | COG3522 | 4e-101 | 99.0 | 2 | 446 |
| CP000578\_Rsph17029\_3130 | 146451 | 147812 | True | COG3455 | 6e-42 | 98.0 | 6 | 262 |
| CP000578\_Rsph17029\_3130 | 146451 | 147812 | True | COG1360 | 3e-23 | 52.0 | 117 | 243 |
| CP000578\_Rsph17029\_3131 | 147812 | 151333 | True | COG3523 | 0.0 | 99.0 | 1 | 1187 |
| CP000578\_Rsph17029\_3132 | 151323 | 151814 | True | COG3913 | 4e-11 | 63.0 | 1 | 145 |
| CP000578\_Rsph17029\_3133 | 151824 | 154127 | False | COG3501 | 6e-144 | 96.0 | 15 | 547 |
| CP000578\_Rsph17029\_3134 | 154144 | 156552 | False | COG3523 | 6e-43 | 63.0 | 430 | 1187 |
| CP000578\_Rsph17029\_3135 | 156552 | 157400 | False | - | - | - | - | - |
| CP000578\_Rsph17029\_3136 | 157404 | 158159 | False | COG0631 | 3e-50 | 91.0 | 12 | 251 |
| CP000578\_Rsph17029\_3137 | 158302 | 159066 | True | COG2885 | 5e-19 | 93.0 | 9 | 186 |
| CP000578\_Rsph17029\_3138 | 159063 | 159989 | False | - | - | - | - | - |
| CP000578\_Rsph17029\_3139 | 159986 | 160396 | False | - | - | - | - | - |
| CP000578\_Rsph17029\_3140 | 160396 | 161469 | False | COG0438 | 2e-10 | 77.0 | 86 | 380 |
