# Supplementary material for: Dissecting the bacterial type VI secretion system by a genome wide in silico analysis: what can be learned from available microbial genomic resources?
Source: BMC Genomics. 2009 Mar 12;10:104. doi: 10.1186/1471-2164-10-104 (PMC2660368; doi:10.1186/1471-2164-10-104)
Supplement: Additional file 7 — Detailed description of all identified T6SS gene clusters. Archive containing the detailed description of each identified T6SS locus as an HTML file. [file 1471-2164-10-104-S7.tgz › LociHTML/HTML/CP000644A.html]

Locus CP000644A on Aeromonas salmonicida (strain A449) chromosome, complete sequence.

import namespace="svg" implementation="#AdobeSVG"?


# Locus CP000644A

# List of CDS in T6SS locus CP000644A

|  |  |  |  |  |  |  |  |  |
| --- | --- | --- | --- | --- | --- | --- | --- | --- |
| Name | from | to | direct | COG | e-value | COG cover | COG hit start | COG hit end |
| CP000644\_ASA\_2440 | 2604522 | 2605472 | True | COG0492 | 3e-96 | 98.0 | 2 | 301 |
| CP000644\_ASA\_2441 | 2605530 | 2606777 | True | COG3706 | 2e-55 | 89.0 | 45 | 433 |
| CP000644\_ASA\_2442 | 2606880 | 2607350 | False | COG3133 | 4e-15 | 98.0 | 1 | 152 |
| CP000644\_ASA\_2443 | 2607340 | 2608077 | True | COG2360 | 9e-81 | 96.0 | 3 | 215 |
| CP000644\_ASA\_2444 | 2608074 | 2608790 | True | COG2935 | 2e-57 | 91.0 | 10 | 240 |
| CP000644\_ASA\_2445 | 2608859 | 2609077 | True | COG0361 | 2e-25 | 96.0 | 1 | 72 |
| CP000644\_ASA\_2446 | 2609146 | 2611398 | False | COG0542 | 0.0 | 99.0 | 1 | 785 |
| CP000644\_ASA\_2447 | 2611458 | 2611775 | False | COG2127 | 4e-31 | 82.0 | 20 | 107 |
| CP000644\_ASA\_2448 | 2612005 | 2612223 | True | COG1278 | 4e-23 | 98.0 | 1 | 66 |
| CP000644\_ASA\_2449 | 2612334 | 2613197 | False | COG0190 | 1e-113 | 99.0 | 1 | 281 |
| CP000644\_ASA\_2453 | 2613754 | 2614005 | False | - | - | - | - | - |
| CP000644\_ASA\_2454 | 2614056 | 2614454 | False | COG1662 | 3e-25 | 100.0 | 1 | 121 |
| CP000644\_ASA\_2455 | 2614444 | 2615091 | False | COG3501 | 6e-19 | 17.0 | 6 | 101 |
| CP000644\_ASA\_2455 | 2614444 | 2615091 | False | COG3677 | 3e-17 | 66.0 | 28 | 113 |
| CP000644\_ASA\_2456 | 2615103 | 2615390 | False | COG4104 | 2e-08 | 91.0 | 3 | 92 |
| CP000644\_ASA\_2457 | 2615682 | 2617118 | False | COG3515 | 2e-18 | 47.0 | 19 | 184 |
| CP000644\_ASA\_2459 | 2622062 | 2622667 | False | - | - | - | - | - |
| CP000644\_ASA\_2460 | 2622667 | 2624205 | False | COG3829 | 6e-95 | 62.0 | 208 | 559 |
| CP000644\_ASA\_2461 | 2624208 | 2626850 | False | COG0542 | 0.0 | 99.0 | 1 | 784 |
| CP000644\_ASA\_2462 | 2626872 | 2627651 | False | COG3455 | 9e-66 | 97.0 | 6 | 260 |
| CP000644\_ASA\_2463 | 2627675 | 2629009 | False | COG3522 | 2e-149 | 100.0 | 1 | 446 |
| CP000644\_ASA\_2464 | 2629012 | 2629527 | False | COG3521 | 3e-32 | 96.0 | 6 | 159 |
| CP000644\_ASA\_2465 | 2629527 | 2630750 | False | COG3456 | 5e-72 | 99.0 | 1 | 428 |
| CP000644\_ASA\_2466 | 2630791 | 2631795 | False | COG3520 | 2e-80 | 97.0 | 4 | 328 |
| CP000644\_ASA\_2467 | 2631759 | 2633525 | False | COG3519 | 1e-148 | 99.0 | 3 | 621 |
| CP000644\_ASA\_2468 | 2633529 | 2633960 | False | COG3518 | 3e-19 | 96.0 | 1 | 152 |
| CP000644\_ASA\_2470 | 2635484 | 2635987 | False | COG3516 | 5e-44 | 94.0 | 10 | 168 |
| CP000644\_ASA\_2474 | 2638939 | 2640675 | False | COG0659 | 6e-106 | 97.0 | 15 | 553 |
