# Supplementary material for: Dissecting the bacterial type VI secretion system by a genome wide in silico analysis: what can be learned from available microbial genomic resources?
Source: BMC Genomics. 2009 Mar 12;10:104. doi: 10.1186/1471-2164-10-104 (PMC2660368; doi:10.1186/1471-2164-10-104)
Supplement: Additional file 7 — Detailed description of all identified T6SS gene clusters. Archive containing the detailed description of each identified T6SS locus as an HTML file. [file 1471-2164-10-104-S7.tgz › LociHTML/HTML/CP000668A.html]

Locus CP000668A on Yersinia pestis (strain Pestoides F) chromosome, complete sequence.

import namespace="svg" implementation="#AdobeSVG"?


# Locus CP000668A

# List of CDS in T6SS locus CP000668A

|  |  |  |  |  |  |  |  |  |
| --- | --- | --- | --- | --- | --- | --- | --- | --- |
| Name | from | to | direct | COG | e-value | COG cover | COG hit start | COG hit end |
| CP000668\_YPDSF\_0187 | 206026 | 206262 | True | - | - | - | - | - |
| CP000668\_YPDSF\_0188 | 206266 | 207375 | True | COG3839 | 9e-125 | 100.0 | 1 | 338 |
| CP000668\_YPDSF\_0189 | 207446 | 208717 | True | COG4580 | 4e-150 | 99.0 | 2 | 429 |
| CP000668\_YPDSF\_0190 | 208958 | 209869 | True | - | - | - | - | - |
| CP000668\_YPDSF\_0191 | 210208 | 210618 | True | - | - | - | - | - |
| CP000668\_YPDSF\_0192 | 210770 | 211288 | False | COG3157 | 1e-50 | 98.0 | 1 | 160 |
| CP000668\_YPDSF\_0193 | 211812 | 212309 | True | COG3516 | 1e-49 | 98.0 | 2 | 167 |
| CP000668\_YPDSF\_0194 | 212377 | 213858 | True | COG3517 | 0.0 | 99.0 | 1 | 493 |
| CP000668\_YPDSF\_0195 | 213865 | 214305 | True | COG3518 | 9e-35 | 100.0 | 1 | 157 |
| CP000668\_YPDSF\_0196 | 214305 | 215531 | True | COG3519 | 6e-95 | 59.0 | 3 | 374 |
| CP000668\_YPDSF\_0197 | 215421 | 216206 | False | COG2801 | 1e-17 | 92.0 | 16 | 230 |
| CP000668\_YPDSF\_0198 | 216260 | 216565 | False | - | - | - | - | - |
| CP000668\_YPDSF\_0199 | 216826 | 217569 | True | COG3519 | 1e-59 | 39.0 | 379 | 621 |
| CP000668\_YPDSF\_0200 | 217533 | 218621 | True | COG3520 | 7e-106 | 99.0 | 1 | 332 |
| CP000668\_YPDSF\_0201 | 218747 | 220063 | True | COG3456 | 9e-124 | 100.0 | 1 | 430 |
| CP000668\_YPDSF\_0202 | 220063 | 220608 | True | COG3521 | 2e-39 | 100.0 | 1 | 159 |
| CP000668\_YPDSF\_0203 | 220611 | 221957 | True | COG3522 | 1e-167 | 100.0 | 1 | 446 |
| CP000668\_YPDSF\_0204 | 221957 | 222724 | True | COG3455 | 2e-86 | 98.0 | 4 | 260 |
| CP000668\_YPDSF\_0205 | 222735 | 225329 | True | COG0542 | 0.0 | 99.0 | 1 | 784 |
| CP000668\_YPDSF\_0206 | 225326 | 226123 | True | - | - | - | - | - |
| CP000668\_YPDSF\_0207 | 226120 | 226806 | True | - | - | - | - | - |
| CP000668\_YPDSF\_0208 | 226812 | 228200 | True | COG3515 | 2e-36 | 82.0 | 1 | 285 |
| CP000668\_YPDSF\_0209 | 228232 | 231765 | True | COG3523 | 0.0 | 100.0 | 1 | 1188 |
| CP000668\_YPDSF\_0210 | 231890 | 232672 | True | COG3515 | 4e-44 | 78.0 | 12 | 284 |
| CP000668\_YPDSF\_0211 | 232579 | 233202 | True | COG3515 | 3e-20 | 51.0 | 10 | 188 |
| CP000668\_YPDSF\_0212 | 233224 | 235614 | True | COG3501 | 0.0 | 99.0 | 2 | 547 |
| CP000668\_YPDSF\_0213 | 235620 | 236078 | True | COG5435 | 5e-48 | 100.0 | 1 | 147 |
| CP000668\_YPDSF\_0214 | 236071 | 238935 | True | COG3209 | 1e-61 | 99.0 | 2 | 794 |
| CP000668\_YPDSF\_0215 | 238963 | 240333 | True | COG3209 | 8e-32 | 59.0 | 326 | 795 |
| CP000668\_YPDSF\_0216 | 240870 | 241061 | False | - | - | - | - | - |
| CP000668\_YPDSF\_0217 | 241137 | 241361 | False | - | - | - | - | - |
| CP000668\_YPDSF\_0218 | 241443 | 241850 | False | - | - | - | - | - |
| CP000668\_YPDSF\_0219 | 242013 | 242189 | True | - | - | - | - | - |
| CP000668\_YPDSF\_0220 | 242211 | 244409 | True | COG3501 | 0.0 | 99.0 | 1 | 549 |
| CP000668\_YPDSF\_0221 | 244412 | 244834 | True | COG5435 | 6e-45 | 97.0 | 3 | 145 |
| CP000668\_YPDSF\_0222 | 244879 | 249417 | True | COG3209 | 3e-63 | 99.0 | 1 | 795 |
| CP000668\_YPDSF\_0222 | 244879 | 249417 | True | COG4104 | 4e-10 | 73.0 | 25 | 96 |
