# Supplementary material for: Dissecting the bacterial type VI secretion system by a genome wide in silico analysis: what can be learned from available microbial genomic resources?
Source: BMC Genomics. 2009 Mar 12;10:104. doi: 10.1186/1471-2164-10-104 (PMC2660368; doi:10.1186/1471-2164-10-104)
Supplement: Additional file 7 — Detailed description of all identified T6SS gene clusters. Archive containing the detailed description of each identified T6SS locus as an HTML file. [file 1471-2164-10-104-S7.tgz › LociHTML/HTML/CP000668B.html]

Locus CP000668B on Yersinia pestis (strain Pestoides F) chromosome, complete sequence.

import namespace="svg" implementation="#AdobeSVG"?


# Locus CP000668B

# List of CDS in T6SS locus CP000668B

|  |  |  |  |  |  |  |  |  |
| --- | --- | --- | --- | --- | --- | --- | --- | --- |
| Name | from | to | direct | COG | e-value | COG cover | COG hit start | COG hit end |
| CP000668\_YPDSF\_0534 | 595415 | 596065 | False | COG3916 | 3e-64 | 100.0 | 1 | 209 |
| CP000668\_YPDSF\_0535 | 597610 | 597891 | True | - | - | - | - | - |
| CP000668\_YPDSF\_0536 | 598342 | 598737 | True | - | - | - | - | - |
| CP000668\_YPDSF\_0537 | 598805 | 599068 | True | COG3677 | 2e-18 | 68.0 | 26 | 114 |
| CP000668\_YPDSF\_0538 | 601013 | 601513 | True | COG3516 | 2e-48 | 99.0 | 2 | 169 |
| CP000668\_YPDSF\_0539 | 601562 | 603106 | True | COG3517 | 0.0 | 100.0 | 1 | 495 |
| CP000668\_YPDSF\_0540 | 603118 | 604470 | True | COG3522 | 6e-133 | 99.0 | 2 | 446 |
| CP000668\_YPDSF\_0541 | 604467 | 605153 | True | COG3455 | 2e-48 | 91.0 | 21 | 260 |
| CP000668\_YPDSF\_0542 | 605153 | 606889 | True | COG2885 | 8e-27 | 94.0 | 12 | 190 |
| CP000668\_YPDSF\_0543 | 606866 | 607351 | False | - | - | - | - | - |
| CP000668\_YPDSF\_0544 | 606893 | 607384 | True | COG3157 | 2e-40 | 98.0 | 1 | 160 |
| CP000668\_YPDSF\_0545 | 607802 | 610450 | True | COG0542 | 0.0 | 100.0 | 1 | 786 |
| CP000668\_YPDSF\_0546 | 610447 | 612795 | True | COG3501 | 4e-110 | 99.0 | 1 | 549 |
| CP000668\_YPDSF\_0546 | 610447 | 612795 | True | COG4253 | 5e-68 | 82.0 | 2 | 229 |
| CP000668\_YPDSF\_0547 | 612811 | 615108 | True | COG3179 | 6e-09 | 98.0 | 4 | 206 |
| CP000668\_YPDSF\_0548 | 615095 | 615862 | True | - | - | - | - | - |
| CP000668\_YPDSF\_0549 | 615958 | 616233 | True | COG3677 | 8e-22 | 71.0 | 26 | 117 |
| CP000668\_YPDSF\_0550 | 616287 | 616655 | True | COG1662 | 1e-31 | 95.0 | 6 | 121 |
| CP000668\_YPDSF\_0551 | 617263 | 617898 | True | COG4253 | 6e-61 | 81.0 | 4 | 229 |
