# Supplementary material for: Dissecting the bacterial type VI secretion system by a genome wide in silico analysis: what can be learned from available microbial genomic resources?
Source: BMC Genomics. 2009 Mar 12;10:104. doi: 10.1186/1471-2164-10-104 (PMC2660368; doi:10.1186/1471-2164-10-104)
Supplement: Additional file 7 — Detailed description of all identified T6SS gene clusters. Archive containing the detailed description of each identified T6SS locus as an HTML file. [file 1471-2164-10-104-S7.tgz › LociHTML/HTML/CP000668D.html]

Locus CP000668D on Yersinia pestis (strain Pestoides F) chromosome, complete sequence.

import namespace="svg" implementation="#AdobeSVG"?


# Locus CP000668D

# List of CDS in T6SS locus CP000668D

|  |  |  |  |  |  |  |  |  |
| --- | --- | --- | --- | --- | --- | --- | --- | --- |
| Name | from | to | direct | COG | e-value | COG cover | COG hit start | COG hit end |
| CP000668\_YPDSF\_1481 | 1668932 | 1670206 | True | COG0303 | 5e-130 | 100.0 | 1 | 404 |
| CP000668\_YPDSF\_1482 | 1670208 | 1670978 | True | COG0476 | 7e-71 | 100.0 | 1 | 254 |
| CP000668\_YPDSF\_1483 | 1671043 | 1671696 | False | - | - | - | - | - |
| CP000668\_YPDSF\_1484 | 1671924 | 1673687 | False | COG0488 | 8e-172 | 99.0 | 1 | 528 |
| CP000668\_YPDSF\_1485 | 1674202 | 1674825 | True | - | - | - | - | - |
| CP000668\_YPDSF\_1486 | 1675037 | 1676404 | False | COG3515 | 9e-41 | 96.0 | 13 | 346 |
| CP000668\_YPDSF\_1487 | 1676429 | 1676881 | False | COG3518 | 1e-27 | 98.0 | 3 | 157 |
| CP000668\_YPDSF\_1488 | 1676881 | 1677462 | False | COG3521 | 3e-35 | 98.0 | 1 | 157 |
| CP000668\_YPDSF\_1489 | 1677437 | 1678522 | False | COG3520 | 3e-84 | 97.0 | 1 | 328 |
| CP000668\_YPDSF\_1490 | 1678486 | 1680249 | False | COG3519 | 0.0 | 100.0 | 1 | 621 |
| CP000668\_YPDSF\_1491 | 1680470 | 1680940 | False | - | - | - | - | - |
| CP000668\_YPDSF\_1492 | 1680940 | 1681998 | False | - | - | - | - | - |
| CP000668\_YPDSF\_1493 | 1682016 | 1683617 | False | COG3515 | 8e-42 | 100.0 | 1 | 346 |
| CP000668\_YPDSF\_1494 | 1683661 | 1687083 | False | COG3523 | 0.0 | 100.0 | 1 | 1188 |
| CP000668\_YPDSF\_1495 | 1687080 | 1688312 | False | - | - | - | - | - |
| CP000668\_YPDSF\_1496 | 1689142 | 1689315 | True | - | - | - | - | - |
| CP000668\_YPDSF\_1497 | 1689424 | 1689744 | True | - | - | - | - | - |
| CP000668\_YPDSF\_1498 | 1689875 | 1690012 | True | - | - | - | - | - |
| CP000668\_YPDSF\_1499 | 1691120 | 1693303 | False | - | - | - | - | - |
| CP000668\_YPDSF\_1500 | 1693319 | 1693579 | False | COG4253 | 2e-25 | 30.0 | 144 | 229 |
| CP000668\_YPDSF\_1501 | 1693733 | 1694506 | False | - | - | - | - | - |
| CP000668\_YPDSF\_1502 | 1694503 | 1696734 | False | - | - | - | - | - |
| CP000668\_YPDSF\_1503 | 1696819 | 1699167 | False | COG3501 | 1e-105 | 99.0 | 1 | 549 |
| CP000668\_YPDSF\_1503 | 1696819 | 1699167 | False | COG4253 | 6e-67 | 82.0 | 2 | 229 |
| CP000668\_YPDSF\_1504 | 1699170 | 1701812 | False | COG0542 | 0.0 | 99.0 | 1 | 784 |
| CP000668\_YPDSF\_1505 | 1702200 | 1702691 | False | COG3157 | 2e-40 | 98.0 | 1 | 160 |
| CP000668\_YPDSF\_1506 | 1702233 | 1702718 | True | - | - | - | - | - |
| CP000668\_YPDSF\_1507 | 1702695 | 1704431 | False | COG2885 | 5e-27 | 94.0 | 12 | 190 |
| CP000668\_YPDSF\_1508 | 1704431 | 1705117 | False | COG3455 | 2e-48 | 91.0 | 21 | 260 |
| CP000668\_YPDSF\_1509 | 1705114 | 1706466 | False | COG3522 | 4e-132 | 99.0 | 2 | 446 |
| CP000668\_YPDSF\_1510 | 1706478 | 1708022 | False | COG3517 | 0.0 | 100.0 | 1 | 495 |
| CP000668\_YPDSF\_1511 | 1708071 | 1708571 | False | COG3516 | 2e-48 | 99.0 | 2 | 169 |
| CP000668\_YPDSF\_1512 | 1709560 | 1710408 | False | COG0331 | 2e-74 | 93.0 | 2 | 290 |
| CP000668\_YPDSF\_1513 | 1710793 | 1711281 | False | - | - | - | - | - |
| CP000668\_YPDSF\_1514 | 1711320 | 1712108 | False | COG1028 | 4e-25 | 99.0 | 2 | 250 |
| CP000668\_YPDSF\_1515 | 1712111 | 1712887 | False | COG1024 | 5e-37 | 93.0 | 1 | 240 |
| CP000668\_YPDSF\_1516 | 1712865 | 1713584 | False | COG1024 | 6e-29 | 87.0 | 29 | 252 |
