# Supplementary material for: Dissecting the bacterial type VI secretion system by a genome wide in silico analysis: what can be learned from available microbial genomic resources?
Source: BMC Genomics. 2009 Mar 12;10:104. doi: 10.1186/1471-2164-10-104 (PMC2660368; doi:10.1186/1471-2164-10-104)
Supplement: Additional file 7 — Detailed description of all identified T6SS gene clusters. Archive containing the detailed description of each identified T6SS locus as an HTML file. [file 1471-2164-10-104-S7.tgz › LociHTML/HTML/CP000668E.html]

Locus CP000668E on Yersinia pestis (strain Pestoides F) chromosome, complete sequence.

import namespace="svg" implementation="#AdobeSVG"?


# Locus CP000668E

# List of CDS in T6SS locus CP000668E

|  |  |  |  |  |  |  |  |  |
| --- | --- | --- | --- | --- | --- | --- | --- | --- |
| Name | from | to | direct | COG | e-value | COG cover | COG hit start | COG hit end |
| CP000668\_YPDSF\_2027 | 2286377 | 2286844 | False | - | - | - | - | - |
| CP000668\_YPDSF\_2028 | 2287460 | 2288002 | True | COG1704 | 3e-55 | 99.0 | 2 | 185 |
| CP000668\_YPDSF\_2029 | 2287947 | 2290046 | True | COG4907 | 4e-08 | 30.0 | 412 | 594 |
| CP000668\_YPDSF\_2030 | 2290422 | 2290763 | True | COG2824 | 2e-46 | 100.0 | 1 | 112 |
| CP000668\_YPDSF\_2031 | 2290838 | 2291203 | False | - | - | - | - | - |
| CP000668\_YPDSF\_2032 | 2291210 | 2291689 | False | COG3518 | 4e-35 | 99.0 | 1 | 156 |
| CP000668\_YPDSF\_2033 | 2291770 | 2292576 | False | COG4455 | 9e-108 | 100.0 | 1 | 273 |
| CP000668\_YPDSF\_2034 | 2292596 | 2293444 | False | - | - | - | - | - |
| CP000668\_YPDSF\_2035 | 2293444 | 2293704 | False | - | - | - | - | - |
| CP000668\_YPDSF\_2036 | 2293803 | 2296082 | False | COG3501 | 5e-152 | 97.0 | 6 | 539 |
| CP000668\_YPDSF\_2039 | 2300211 | 2301047 | False | COG1360 | 4e-32 | 59.0 | 95 | 240 |
| CP000668\_YPDSF\_2039 | 2300211 | 2301047 | False | COG3455 | 6e-30 | 42.0 | 151 | 262 |
| CP000668\_YPDSF\_2040 | 2301063 | 2301842 | False | COG1484 | 5e-64 | 99.0 | 2 | 254 |
| CP000668\_YPDSF\_2041 | 2301842 | 2302864 | False | COG4584 | 2e-58 | 100.0 | 1 | 278 |
| CP000668\_YPDSF\_2042 | 2302878 | 2303546 | False | COG3455 | 5e-18 | 42.0 | 40 | 151 |
| CP000668\_YPDSF\_2043 | 2303543 | 2304892 | False | COG3522 | 3e-158 | 99.0 | 2 | 446 |
| CP000668\_YPDSF\_2044 | 2304896 | 2305456 | False | COG3521 | 4e-39 | 100.0 | 1 | 159 |
| CP000668\_YPDSF\_2045 | 2305709 | 2306278 | False | COG3157 | 4e-42 | 100.0 | 1 | 162 |
| CP000668\_YPDSF\_2046 | 2306520 | 2308022 | False | COG3517 | 0.0 | 100.0 | 1 | 495 |
| CP000668\_YPDSF\_2047 | 2308046 | 2308570 | False | COG3516 | 8e-59 | 99.0 | 2 | 169 |
| CP000668\_YPDSF\_2048 | 2308675 | 2309283 | False | COG3539 | 3e-15 | 91.0 | 16 | 184 |
| CP000668\_YPDSF\_2049 | 2309268 | 2310458 | False | COG3188 | 2e-61 | 44.0 | 464 | 834 |
| CP000668\_YPDSF\_2050 | 2310483 | 2311691 | True | COG3328 | 2e-112 | 98.0 | 1 | 375 |
| CP000668\_YPDSF\_2051 | 2311713 | 2313263 | False | COG3188 | 1e-110 | 56.0 | 7 | 474 |
| CP000668\_YPDSF\_2052 | 2313391 | 2314152 | False | COG3121 | 3e-58 | 94.0 | 12 | 234 |
| CP000668\_YPDSF\_2053 | 2314309 | 2314854 | False | COG3539 | 1e-14 | 100.0 | 1 | 184 |
| CP000668\_YPDSF\_2054 | 2315055 | 2317730 | False | COG0542 | 0.0 | 98.0 | 1 | 777 |
| CP000668\_YPDSF\_2055 | 2318513 | 2320393 | True | COG3519 | 0.0 | 100.0 | 1 | 621 |
| CP000668\_YPDSF\_2056 | 2320393 | 2321238 | True | COG3520 | 1e-76 | 79.0 | 1 | 266 |
| CP000668\_YPDSF\_2057 | 2321300 | 2321413 | True | - | - | - | - | - |
| CP000668\_YPDSF\_2058 | 2321512 | 2322693 | True | COG3515 | 1e-49 | 98.0 | 1 | 341 |
| CP000668\_YPDSF\_2059 | 2322700 | 2323731 | True | - | - | - | - | - |
| CP000668\_YPDSF\_2060 | 2323771 | 2325108 | True | - | - | - | - | - |
| CP000668\_YPDSF\_2061 | 2325054 | 2325806 | True | - | - | - | - | - |
| CP000668\_YPDSF\_2062 | 2325807 | 2327489 | True | COG2885 | 1e-27 | 55.0 | 86 | 190 |
| CP000668\_YPDSF\_2063 | 2327486 | 2327968 | True | COG5435 | 3e-42 | 100.0 | 1 | 147 |
