# Supplementary material for: Dissecting the bacterial type VI secretion system by a genome wide in silico analysis: what can be learned from available microbial genomic resources?
Source: BMC Genomics. 2009 Mar 12;10:104. doi: 10.1186/1471-2164-10-104 (PMC2660368; doi:10.1186/1471-2164-10-104)
Supplement: Additional file 7 — Detailed description of all identified T6SS gene clusters. Archive containing the detailed description of each identified T6SS locus as an HTML file. [file 1471-2164-10-104-S7.tgz › LociHTML/HTML/CP000668F.html]

Locus CP000668F on Yersinia pestis (strain Pestoides F) chromosome, complete sequence.

import namespace="svg" implementation="#AdobeSVG"?


# Locus CP000668F

# List of CDS in T6SS locus CP000668F

|  |  |  |  |  |  |  |  |  |
| --- | --- | --- | --- | --- | --- | --- | --- | --- |
| Name | from | to | direct | COG | e-value | COG cover | COG hit start | COG hit end |
| CP000668\_YPDSF\_3116 | 3537825 | 3540194 | True | COG0417 | 0.0 | 98.0 | 5 | 788 |
| CP000668\_YPDSF\_3117 | 3540639 | 3543545 | True | COG0553 | 2e-72 | 98.0 | 8 | 861 |
| CP000668\_YPDSF\_3118 | 3543801 | 3544154 | False | - | - | - | - | - |
| CP000668\_YPDSF\_3119 | 3544176 | 3547670 | False | COG3523 | 0.0 | 98.0 | 12 | 1187 |
| CP000668\_YPDSF\_3120 | 3547679 | 3549268 | False | COG3455 | 5e-71 | 100.0 | 1 | 262 |
| CP000668\_YPDSF\_3120 | 3547679 | 3549268 | False | COG1360 | 2e-27 | 56.0 | 108 | 244 |
| CP000668\_YPDSF\_3121 | 3549265 | 3550620 | False | COG3522 | 2e-141 | 100.0 | 1 | 446 |
| CP000668\_YPDSF\_3122 | 3550740 | 3551231 | False | COG3521 | 1e-34 | 98.0 | 3 | 158 |
| CP000668\_YPDSF\_3123 | 3551224 | 3551589 | False | - | - | - | - | - |
| CP000668\_YPDSF\_3124 | 3551595 | 3552212 | False | - | - | - | - | - |
| CP000668\_YPDSF\_3125 | 3552205 | 3553308 | False | COG1357 | 1e-18 | 99.0 | 3 | 238 |
| CP000668\_YPDSF\_3126 | 3553334 | 3555553 | False | COG1357 | 4e-15 | 83.0 | 18 | 215 |
| CP000668\_YPDSF\_3126 | 3553334 | 3555553 | False | COG5351 | 1e-07 | 50.0 | 68 | 253 |
| CP000668\_YPDSF\_3127 | 3555566 | 3557914 | False | COG3501 | 5e-154 | 95.0 | 10 | 533 |
| CP000668\_YPDSF\_3128 | 3558018 | 3560606 | False | COG0542 | 0.0 | 99.0 | 1 | 783 |
| CP000668\_YPDSF\_3129 | 3560624 | 3561607 | False | COG3520 | 2e-83 | 98.0 | 4 | 334 |
| CP000668\_YPDSF\_3130 | 3561600 | 3563444 | False | COG3519 | 0.0 | 99.0 | 1 | 617 |
| CP000668\_YPDSF\_3131 | 3563477 | 3563920 | False | COG3518 | 8e-31 | 95.0 | 4 | 153 |
| CP000668\_YPDSF\_3132 | 3563994 | 3564512 | False | COG3157 | 7e-36 | 100.0 | 1 | 162 |
| CP000668\_YPDSF\_3133 | 3564675 | 3566186 | False | COG3517 | 0.0 | 100.0 | 1 | 495 |
| CP000668\_YPDSF\_3134 | 3566186 | 3566746 | False | COG3516 | 1e-57 | 99.0 | 2 | 169 |
| CP000668\_YPDSF\_3135 | 3566757 | 3567755 | False | COG3515 | 6e-54 | 98.0 | 5 | 346 |
| CP000668\_YPDSF\_3136 | 3568181 | 3568843 | True | COG5419 | 1e-49 | 100.0 | 1 | 160 |
| CP000668\_YPDSF\_3137 | 3570066 | 3570686 | True | COG0564 | 1e-59 | 72.0 | 80 | 289 |
| CP000668\_YPDSF\_3138 | 3570984 | 3571817 | False | COG1076 | 6e-35 | 100.0 | 1 | 174 |
| CP000668\_YPDSF\_3139 | 3571966 | 3574344 | True | COG1452 | 0.0 | 100.0 | 1 | 784 |
