# Supplementary material for: Dissecting the bacterial type VI secretion system by a genome wide in silico analysis: what can be learned from available microbial genomic resources?
Source: BMC Genomics. 2009 Mar 12;10:104. doi: 10.1186/1471-2164-10-104 (PMC2660368; doi:10.1186/1471-2164-10-104)
Supplement: Additional file 7 — Detailed description of all identified T6SS gene clusters. Archive containing the detailed description of each identified T6SS locus as an HTML file. [file 1471-2164-10-104-S7.tgz › LociHTML/HTML/CR354531A.html]

Locus CR354531A on Photobacterium profundum (strain SS9) chromosome 1, complete sequence.

import namespace="svg" implementation="#AdobeSVG"?


# Locus CR354531A

# List of CDS in T6SS locus CR354531A

|  |  |  |  |  |  |  |  |  |
| --- | --- | --- | --- | --- | --- | --- | --- | --- |
| Name | from | to | direct | COG | e-value | COG cover | COG hit start | COG hit end |
| CR354531\_PBPRA0653 | 716760 | 717380 | False | - | - | - | - | - |
| CR354531\_PBPRA0654 | 717589 | 718539 | True | COG0739 | 6e-34 | 47.0 | 139 | 269 |
| CR354531\_PBPRA0655 | 718443 | 718889 | True | COG1664 | 3e-17 | 93.0 | 10 | 145 |
| CR354531\_PBPRA0656 | 719070 | 719549 | True | COG3542 | 4e-41 | 96.0 | 5 | 161 |
| CR354531\_PBPRA0657 | 719613 | 721811 | False | COG0515 | 1e-26 | 73.0 | 2 | 284 |
| CR354531\_PBPRA0658 | 721945 | 723255 | True | COG3456 | 1e-36 | 99.0 | 1 | 427 |
| CR354531\_PBPRA0659 | 723258 | 723725 | True | COG3521 | 1e-24 | 80.0 | 7 | 134 |
| CR354531\_PBPRA0660 | 723881 | 725206 | True | COG3522 | 1e-117 | 100.0 | 1 | 446 |
| CR354531\_PBPRA0661 | 725233 | 726552 | True | COG3455 | 5e-47 | 87.0 | 32 | 260 |
| CR354531\_PBPRA0661 | 725233 | 726552 | True | COG1360 | 4e-22 | 60.0 | 97 | 244 |
| CR354531\_PBPRA0662 | 726552 | 730097 | True | COG3523 | 0.0 | 99.0 | 8 | 1187 |
| CR354531\_PBPRA0663 | 730052 | 730765 | True | COG3913 | 4e-21 | 92.0 | 8 | 218 |
| CR354531\_PBPRA0664 | 730681 | 731589 | True | COG0631 | 3e-51 | 97.0 | 7 | 262 |
| CR354531\_PBPRA0665 | 731589 | 732722 | True | COG3515 | 3e-11 | 96.0 | 12 | 344 |
| CR354531\_PBPRA0666 | 732758 | 733291 | True | COG3516 | 7e-49 | 100.0 | 1 | 169 |
| CR354531\_PBPRA0667 | 733291 | 734781 | True | COG3517 | 0.0 | 99.0 | 1 | 491 |
| CR354531\_PBPRA0668 | 734842 | 736383 | True | COG3517 | 9e-121 | 88.0 | 54 | 492 |
| CR354531\_PBPRA0669 | 736388 | 737182 | True | COG4455 | 6e-32 | 91.0 | 12 | 261 |
| CR354531\_PBPRA0670 | 737337 | 737672 | True | COG3518 | 3e-11 | 68.0 | 47 | 154 |
| CR354531\_PBPRA0671 | 737674 | 739503 | True | COG3519 | 2e-115 | 100.0 | 1 | 621 |
| CR354531\_PBPRA0672 | 739467 | 740471 | True | COG3520 | 2e-43 | 88.0 | 22 | 318 |
| CR354531\_PBPRA0673 | 740336 | 743080 | True | COG0542 | 0.0 | 99.0 | 1 | 784 |
| CR354531\_PBPRA0674 | 743312 | 743791 | True | COG3157 | 6e-15 | 92.0 | 1 | 150 |
| CR354531\_PBPRA0675 | 743850 | 745700 | True | COG3501 | 2e-110 | 98.0 | 6 | 549 |
| CR354531\_PBPRA0676 | 745739 | 746029 | True | COG4104 | 1e-10 | 81.0 | 18 | 97 |
| CR354531\_PBPRA0677 | 746042 | 747007 | True | - | - | - | - | - |
| CR354531\_PBPRA0678 | 746998 | 751056 | True | COG3209 | 9e-41 | 82.0 | 8 | 660 |
