# Supplementary material for: Dissecting the bacterial type VI secretion system by a genome wide in silico analysis: what can be learned from available microbial genomic resources?
Source: BMC Genomics. 2009 Mar 12;10:104. doi: 10.1186/1471-2164-10-104 (PMC2660368; doi:10.1186/1471-2164-10-104)
Supplement: Additional file 7 — Detailed description of all identified T6SS gene clusters. Archive containing the detailed description of each identified T6SS locus as an HTML file. [file 1471-2164-10-104-S7.tgz › LociHTML/HTML/CR543861B.html]

Locus CR543861B on Acinetobacter sp. (strain ADP1) chromosome, complete sequence.

import namespace="svg" implementation="#AdobeSVG"?


# Locus CR543861B

# List of CDS in T6SS locus CR543861B

|  |  |  |  |  |  |  |  |  |
| --- | --- | --- | --- | --- | --- | --- | --- | --- |
| Name | from | to | direct | COG | e-value | COG cover | COG hit start | COG hit end |
| CR543861\_ACIAD2678 | 2633143 | 2634144 | False | COG2207 | 3e-16 | 89.0 | 10 | 123 |
| CR543861\_ACIAD2680 | 2634401 | 2635672 | False | COG0334 | 6e-144 | 99.0 | 4 | 411 |
| CR543861\_ACIAD2681 | 2635901 | 2636167 | False | COG4104 | 1e-08 | 84.0 | 10 | 92 |
| CR543861\_ACIAD2682 | 2636197 | 2636961 | False | COG2885 | 1e-27 | 89.0 | 19 | 188 |
| CR543861\_ACIAD2683 | 2636964 | 2637923 | False | COG3913 | 4e-11 | 44.0 | 1 | 101 |
| CR543861\_ACIAD2684 | 2637964 | 2641785 | False | COG3523 | 1e-178 | 93.0 | 49 | 1153 |
| CR543861\_ACIAD2685 | 2641820 | 2643250 | False | - | - | - | - | - |
| CR543861\_ACIAD2686 | 2643220 | 2644218 | False | COG3520 | 7e-41 | 92.0 | 15 | 325 |
| CR543861\_ACIAD2687 | 2644182 | 2645990 | False | COG3519 | 2e-99 | 100.0 | 1 | 621 |
| CR543861\_ACIAD2688 | 2646002 | 2646475 | False | COG3518 | 5e-23 | 98.0 | 3 | 157 |
| CR543861\_ACIAD2689 | 2646545 | 2647048 | False | COG3157 | 2e-28 | 93.0 | 2 | 152 |
| CR543861\_ACIAD2690 | 2647094 | 2648590 | False | COG3517 | 0.0 | 99.0 | 4 | 495 |
| CR543861\_ACIAD2691 | 2648568 | 2649077 | False | COG3516 | 6e-44 | 95.0 | 2 | 162 |
| CR543861\_ACIAD2693 | 2649091 | 2649747 | False | - | - | - | - | - |
| CR543861\_ACIAD2694 | 2650125 | 2652809 | True | COG0542 | 0.0 | 99.0 | 1 | 781 |
| CR543861\_ACIAD2695 | 2652825 | 2653928 | True | COG3515 | 3e-23 | 98.0 | 7 | 346 |
| CR543861\_ACIAD2696 | 2653945 | 2655312 | True | COG3522 | 6e-84 | 99.0 | 1 | 444 |
| CR543861\_ACIAD2697 | 2655324 | 2656127 | True | COG3455 | 1e-36 | 97.0 | 7 | 262 |
| CR543861\_ACIAD2698 | 2656138 | 2656719 | True | - | - | - | - | - |
| CR543861\_ACIAD2699 | 2656820 | 2657683 | True | - | - | - | - | - |
| CR543861\_ACIAD2700 | 2657701 | 2658882 | False | COG2828 | 9e-152 | 98.0 | 1 | 374 |
| CR543861\_ACIAD2701 | 2659669 | 2660025 | True | - | - | - | - | - |
| CR543861\_ACIAD2702 | 2660030 | 2660530 | True | - | - | - | - | - |
| CR543861\_ACIAD2704 | 2660893 | 2661510 | True | - | - | - | - | - |
