# Supplementary material for: Dissecting the bacterial type VI secretion system by a genome wide in silico analysis: what can be learned from available microbial genomic resources?
Source: BMC Genomics. 2009 Mar 12;10:104. doi: 10.1186/1471-2164-10-104 (PMC2660368; doi:10.1186/1471-2164-10-104)
Supplement: Additional file 7 — Detailed description of all identified T6SS gene clusters. Archive containing the detailed description of each identified T6SS locus as an HTML file. [file 1471-2164-10-104-S7.tgz › LociHTML/HTML/CT573326A.html]

Locus CT573326A on Pseudomonas entomophila (strain L48) chromosome, complete sequence.

import namespace="svg" implementation="#AdobeSVG"?


# Locus CT573326A

# List of CDS in T6SS locus CT573326A

|  |  |  |  |  |  |  |  |  |
| --- | --- | --- | --- | --- | --- | --- | --- | --- |
| Name | from | to | direct | COG | e-value | COG cover | COG hit start | COG hit end |
| CT573326\_PSEEN0519 | 532095 | 534929 | False | COG0178 | 0.0 | 99.0 | 3 | 935 |
| CT573326\_PSEEN0520 | 535059 | 536453 | True | COG2814 | 7e-21 | 96.0 | 2 | 383 |
| CT573326\_PSEEN0521 | 536463 | 537005 | True | COG0629 | 1e-34 | 99.0 | 2 | 167 |
| CT573326\_PSEEN0522 | 537545 | 539101 | True | COG3515 | 7e-22 | 95.0 | 1 | 330 |
| CT573326\_PSEEN0523 | 539135 | 539635 | True | COG3516 | 3e-42 | 96.0 | 5 | 167 |
| CT573326\_PSEEN0524 | 539663 | 541135 | True | COG3517 | 0.0 | 98.0 | 8 | 493 |
| CT573326\_PSEEN0525 | 541145 | 541561 | True | COG3518 | 1e-22 | 91.0 | 14 | 157 |
| CT573326\_PSEEN0526 | 541647 | 543434 | True | COG3519 | 2e-159 | 99.0 | 3 | 621 |
| CT573326\_PSEEN0527 | 543398 | 544405 | True | COG3520 | 4e-80 | 99.0 | 1 | 333 |
| CT573326\_PSEEN0528 | 544418 | 547036 | True | COG0542 | 0.0 | 99.0 | 2 | 781 |
| CT573326\_PSEEN0529 | 547047 | 548567 | True | COG3604 | 4e-131 | 94.0 | 30 | 549 |
| CT573326\_PSEEN0530 | 548651 | 548791 | True | - | - | - | - | - |
| CT573326\_PSEEN0531 | 548810 | 549991 | True | COG3456 | 1e-53 | 99.0 | 1 | 427 |
| CT573326\_PSEEN0532 | 549997 | 550503 | True | COG3521 | 8e-34 | 94.0 | 5 | 155 |
| CT573326\_PSEEN0533 | 550500 | 551831 | True | COG3522 | 3e-138 | 99.0 | 2 | 446 |
| CT573326\_PSEEN0534 | 551834 | 552703 | True | COG3455 | 2e-63 | 92.0 | 17 | 258 |
| CT573326\_PSEEN0535 | 552718 | 556245 | True | COG3523 | 0.0 | 99.0 | 7 | 1184 |
| CT573326\_PSEEN0536 | 556245 | 556973 | True | COG0631 | 7e-54 | 93.0 | 7 | 251 |
| CT573326\_PSEEN0537 | 556970 | 558028 | True | COG0515 | 2e-36 | 73.0 | 2 | 285 |
| CT573326\_PSEEN0539 | 558507 | 559025 | True | COG3157 | 3e-45 | 97.0 | 1 | 158 |
| CT573326\_PSEEN0540 | 559133 | 562153 | True | COG4253 | 4e-41 | 92.0 | 1 | 258 |
| CT573326\_PSEEN0540 | 559133 | 562153 | True | COG3501 | 5e-153 | 99.0 | 5 | 550 |
| CT573326\_PSEEN0541 | 562150 | 566832 | True | COG3209 | 3e-31 | 82.0 | 1 | 660 |
| CT573326\_PSEEN0541 | 562150 | 566832 | True | COG3209 | 9e-09 | 55.0 | 179 | 621 |
| CT573326\_PSEEN0542 | 566835 | 567218 | True | - | - | - | - | - |
