# Supplementary material for: Dissecting the bacterial type VI secretion system by a genome wide in silico analysis: what can be learned from available microbial genomic resources?
Source: BMC Genomics. 2009 Mar 12;10:104. doi: 10.1186/1471-2164-10-104 (PMC2660368; doi:10.1186/1471-2164-10-104)
Supplement: Additional file 7 — Detailed description of all identified T6SS gene clusters. Archive containing the detailed description of each identified T6SS locus as an HTML file. [file 1471-2164-10-104-S7.tgz › LociHTML/index.html]

List of identified T6SS loci - Boyer et al. BMC Genomics, 2009


# List of identified T6SS loci

 Locus AE003853C on Vibrio cholerae (serovar O1, strain ATCC 39315 / El Tor Inaba N16961) chromosome 2, complete sequence.   
 Locus AE004091A on Pseudomonas aeruginosa (strain LMG 12228 / ATCC 15692 / PRS 101 / 1C / PAO1) chromosome, complete sequence.   
 Locus AE004091D on Pseudomonas aeruginosa (strain LMG 12228 / ATCC 15692 / PRS 101 / 1C / PAO1) chromosome, complete sequence.   
 Locus AE004091E on Pseudomonas aeruginosa (strain LMG 12228 / ATCC 15692 / PRS 101 / 1C / PAO1) chromosome, complete sequence.   
 Locus AE005174A on Escherichia coli (strain EDL933 / ATCC 700927 / O157:H7 / EHEC) chromosome, complete sequence.   
 Locus AE005674A on Shigella flexneri (serovar 2a, strain 301) chromosome, complete sequence.   
 Locus AE006468A on Salmonella typhimurium (strain ATCC 700720 / SGSC1412 / LT2) chromosome, complete sequence.   
 Locus AE007870A on Agrobacterium tumefaciens (strain C58 / ATCC 33970, sub\_strain Cereon) chromosome linear, complete sequence.   
 Locus AE008689B on Agrobacterium tumefaciens (strain C58 / ATCC 33970, sub\_strain Dupont) chromosome linear, complete sequence.   
 Locus AE008923B on Xanthomonas axonopodis citri (strain 306) chromosome, complete sequence.   
 Locus AE008923C on Xanthomonas axonopodis citri (strain 306) chromosome, complete sequence.   
 Locus AE009952A on Yersinia pestis (biovar Mediaevalis, strain KIM5) chromosome, complete sequence.   
 Locus AE009952B on Yersinia pestis (biovar Mediaevalis, strain KIM5) chromosome, complete sequence.   
 Locus AE009952C on Yersinia pestis (biovar Mediaevalis, strain KIM5) chromosome, complete sequence.   
 Locus AE009952E on Yersinia pestis (biovar Mediaevalis, strain KIM5) chromosome, complete sequence.   
 Locus AE009952F on Yersinia pestis (biovar Mediaevalis, strain KIM5) chromosome, complete sequence.   
 Locus AE009952G on Yersinia pestis (biovar Mediaevalis, strain KIM5) chromosome, complete sequence.   
 Locus AE013598B on Xanthomonas oryzae oryzae (strain KXO85 / KACC10331) chromosome, complete sequence.   
 Locus AE013598C on Xanthomonas oryzae oryzae (strain KXO85 / KACC10331) chromosome, complete sequence.   
 Locus AE014073A on Shigella flexneri (serovar 2a, strain 2457T / ATCC 700930) chromosome, complete sequence.   
 Locus AE014075B on Escherichia coli O6 (strain UPEC / O6:H1 / ATCC 700928 / CFT073) chromosome, complete sequence.   
 Locus AE014613A on Salmonella typhi (strain ATCC 700931 / Ty2) chromosome, complete sequence.   
 Locus AE015451C on Pseudomonas putida (strain KT2440) chromosome, complete sequence.   
 Locus AE015451D on Pseudomonas putida (strain KT2440) chromosome, complete sequence.   
 Locus AE015451F on Pseudomonas putida (strain KT2440) chromosome, complete sequence.   
 Locus AE016796C on Vibrio vulnificus (strain CMCP6) chromosome 2, complete sequence.   
 Locus AE016825C on Chromobacterium violaceum (strain IFO 12614 / ATCC 12472 / DSM 30191 / NCIB 9131 / JCM 1249) chromosome, complete sequence.   
 Locus AE016853A on Pseudomonas syringae tomato (strain DC3000) chromosome, complete sequence.   
 Locus AE016853C on Pseudomonas syringae tomato (strain DC3000) chromosome, complete sequence.   
 Locus AE017042A on Yersinia pestis (biovar Mediaevalis, strain 91001) chromosome, complete sequence.   
 Locus AE017042E on Yersinia pestis (biovar Mediaevalis, strain 91001) chromosome, complete sequence.   
 Locus AE017042G on Yersinia pestis (biovar Mediaevalis, strain 91001) chromosome, complete sequence.   
 Locus AE017042H on Yersinia pestis (biovar Mediaevalis, strain 91001) chromosome, complete sequence.   
 Locus AE017042I on Yersinia pestis (biovar Mediaevalis, strain 91001) chromosome, complete sequence.   
 Locus AE017125A on Helicobacter hepaticus (strain 3B1 / ATCC 51449) chromosome, complete sequence.   
 Locus AE017180A on Geobacter sulfurreducens (strain ATCC 51573 / DSM 12127 / PCA) chromosome, complete sequence.   
 Locus AE017180C on Geobacter sulfurreducens (strain ATCC 51573 / DSM 12127 / PCA) chromosome, complete sequence.   
 Locus AE017220B on Salmonella choleraesuis (strain SC-B67) chromosome, complete sequence.   
 Locus AL513382A on Salmonella typhi (strain CT18) chromosome, complete sequence.   
 Locus AL590842A on Yersinia pestis (biovar Orientalis, strain CO-92) chromosome, complete sequence.   
 Locus AL590842B on Yersinia pestis (biovar Orientalis, strain CO-92) chromosome, complete sequence.   
 Locus AL590842C on Yersinia pestis (biovar Orientalis, strain CO-92) chromosome, complete sequence.   
 Locus AL590842E on Yersinia pestis (biovar Orientalis, strain CO-92) chromosome, complete sequence.   
 Locus AL590842F on Yersinia pestis (biovar Orientalis, strain CO-92) chromosome, complete sequence.   
 Locus AL590842G on Yersinia pestis (biovar Orientalis, strain CO-92) chromosome, complete sequence.   
 Locus AL646053C on Ralstonia solanacearum (strain GMI1000) plasmid megaplasmid Rsp, complete sequence.   
 Locus AM039952A on Xanthomonas campestris (pathovar vesicatoria, strain 85-10) chromosome, complete sequence.   
 Locus AM039952C on Xanthomonas campestris (pathovar vesicatoria, strain 85-10) chromosome, complete sequence.   
 Locus AM039952D on Xanthomonas campestris (pathovar vesicatoria, strain 85-10) chromosome, complete sequence.   
 Locus AM167904A on Bordetella avium (strain 197N) chromosome, complete sequence.   
 Locus AM236086B on Rhizobium leguminosarum bv. viciae (strain 3841) plasmid pRL12, complete sequence.   
 Locus AM260479A on Ralstonia eutropha (strain ATCC 17699 / H16 / DSM 428 / Stanier 337) chromosome 1, complete sequence.   
 Locus AM260480F on Ralstonia eutropha (strain ATCC 17699 / H16 / DSM 428 / Stanier 337) chromosome 2, complete sequence.   
 Locus AM286415A on Yersinia enterocolitica (serovar O:8 / biotype 1B, strain 8081) chromosome, complete sequence.   
 Locus AM406670A on Azoarcus sp. (strain BH72) chromosome, complete sequence.   
 Locus AM406670E on Azoarcus sp. (strain BH72) chromosome, complete sequence.   
 Locus AP008229B on Xanthomonas oryzae (pathovar oryzae, strain MAFF 311018) chromosome, complete sequence.   
 Locus AP008229C on Xanthomonas oryzae (pathovar oryzae, strain MAFF 311018) chromosome, complete sequence.   
 Locus BA000007A on Escherichia coli (strain Sakai / O157:H7 / RIMD 0509952 / EHEC) chromosome, complete sequence.   
 Locus BA000012A on Rhizobium loti (strain MAFF303099) chromosome, complete sequence.   
 Locus BA000031B on Vibrio parahaemolyticus (serovar O3:K6, strain RIMD 2210633) chromosome 1, complete sequence.   
 Locus BA000032D on Vibrio parahaemolyticus (serovar O3:K6, strain RIMD 2210633) chromosome 2, complete sequence.   
 Locus BA000038C on Vibrio vulnificus (strain YJ016) chromosome II, complete sequence.   
 Locus BA000040A on Bradyrhizobium japonicum (strain USDA 110) chromosome, complete sequence.   
 Locus BX119912C on Rhodopirellula baltica (strain 1) chromosome, complete sequence.   
 Locus BX470249B on Bordetella parapertussis (strain NCTC 13253 / ATCC BAA-587 / 12822) chromosome, complete sequence.   
 Locus BX470250A on Bordetella bronchiseptica (strain NCTC 13252 / RB50 / ATCC BAA-588) chromosome, complete sequence.   
 Locus BX470251C on Photorhabdus luminescens laumondii (strain TT01) chromosome, complete sequence.   
 Locus BX470251I on Photorhabdus luminescens laumondii (strain TT01) chromosome, complete sequence.   
 Locus BX470251M on Photorhabdus luminescens laumondii (strain TT01) chromosome, complete sequence.   
 Locus BX470251N on Photorhabdus luminescens laumondii (strain TT01) chromosome, complete sequence.   
 Locus BX571965D on Burkholderia pseudomallei (strain K96243) chromosome 1, complete sequence.   
 Locus BX571966E on Burkholderia pseudomallei (strain K96243) chromosome 2, complete sequence.   
 Locus BX571966F on Burkholderia pseudomallei (strain K96243) chromosome 2, complete sequence.   
 Locus BX571966G on Burkholderia pseudomallei (strain K96243) chromosome 2, complete sequence.   
 Locus BX571966I on Burkholderia pseudomallei (strain K96243) chromosome 2, complete sequence.   
 Locus BX571966J on Burkholderia pseudomallei (strain K96243) chromosome 2, complete sequence.   
 Locus BX936398A on Yersinia pseudotuberculosis (serovar I, strain IP32953) chromosome, complete sequence.   
 Locus BX936398B on Yersinia pseudotuberculosis (serovar I, strain IP32953) chromosome, complete sequence.   
 Locus BX936398C on Yersinia pseudotuberculosis (serovar I, strain IP32953) chromosome, complete sequence.   
 Locus BX936398D on Yersinia pseudotuberculosis (serovar I, strain IP32953) chromosome, complete sequence.   
 Locus BX936398E on Yersinia pseudotuberculosis (serovar I, strain IP32953) chromosome, complete sequence.   
 Locus BX950851C on Erwinia carotovora (subsp. atroseptica, strain ATCC BAA-672 / SCRI 1043) chromosome, complete sequence.   
 Locus CP000011C on Burkholderia mallei (strain ATCC 23344) chromosome 2, complete sequence.   
 Locus CP000011D on Burkholderia mallei (strain ATCC 23344) chromosome 2, complete sequence.   
 Locus CP000011E on Burkholderia mallei (strain ATCC 23344) chromosome 2, complete sequence.   
 Locus CP000011F on Burkholderia mallei (strain ATCC 23344) chromosome 2, complete sequence.   
 Locus CP000020A on Vibrio fischeri (strain ATCC 700601 / ES114) chromosome I, complete sequence.   
 Locus CP000026A on Salmonella paratyphi-a (strain SARB42 / ATCC 9150) chromosome, complete sequence.   
 Locus CP000038A on Shigella sonnei (strain Ss046) chromosome, complete sequence.   
 Locus CP000058A on Pseudomonas syringae (pathovar phaseolicola, strain 1448A / Race 6) chromosome, complete sequence.   
 Locus CP000075C on Pseudomonas syringae (pathovar syringae, strain B728a) chromosome, complete sequence.   
 Locus CP000076D on Pseudomonas fluorescens (strain Pf-5 / ATCC BAA-477) chromosome, complete sequence.   
 Locus CP000085A on Burkholderia thailandensis (strain E264 / ATCC 700388 / DSM 13276 / CIP 106301) chromosome II, complete sequence.   
 Locus CP000085B on Burkholderia thailandensis (strain E264 / ATCC 700388 / DSM 13276 / CIP 106301) chromosome II, complete sequence.   
 Locus CP000085C on Burkholderia thailandensis (strain E264 / ATCC 700388 / DSM 13276 / CIP 106301) chromosome II, complete sequence.   
 Locus CP000085E on Burkholderia thailandensis (strain E264 / ATCC 700388 / DSM 13276 / CIP 106301) chromosome II, complete sequence.   
 Locus CP000086H on Burkholderia thailandensis (strain E264 / ATCC 700388 / DSM 13276 / CIP 106301) chromosome I, complete sequence.   
 Locus CP000089A on Dechloromonas aromatica (strain RCB) chromosome, complete sequence.   
 Locus CP000090B on Ralstonia eutropha (strain JMP134) chromosome 1, complete sequence.   
 Locus CP000091E on Ralstonia eutropha (strain JMP134) chromosome 2, complete sequence.   
 Locus CP000094C on Pseudomonas fluorescens (strain PfO-1) chromosome, complete sequence.   
 Locus CP000094E on Pseudomonas fluorescens (strain PfO-1) chromosome, complete sequence.   
 Locus CP000113D on Myxococcus xanthus (strain DK 1622) chromosome, complete sequence.   
 Locus CP000124B on Burkholderia pseudomallei (strain 1710b) chromosome I, complete sequence.   
 Locus CP000125C on Burkholderia pseudomallei (strain 1710b) chromosome II, complete sequence.   
 Locus CP000125D on Burkholderia pseudomallei (strain 1710b) chromosome II, complete sequence.   
 Locus CP000125F on Burkholderia pseudomallei (strain 1710b) chromosome II, complete sequence.   
 Locus CP000125G on Burkholderia pseudomallei (strain 1710b) chromosome II, complete sequence.   
 Locus CP000125H on Burkholderia pseudomallei (strain 1710b) chromosome II, complete sequence.   
 Locus CP000142A on Pelobacter carbinolicus (strain DSM 2380 / Gra Bd 1) chromosome, complete sequence.   
 Locus CP000144A on Rhodobacter sphaeroides (strain ATCC 17023 / 2.4.1 / NCIB 8253 / DSM 158) chromosome 2, complete sequence.   
 Locus CP000148A on Geobacter metallireducens (strain GS-15 / ATCC 53774 / DSM 7210) chromosome, complete sequence.   
 Locus CP000148D on Geobacter metallireducens (strain GS-15 / ATCC 53774 / DSM 7210) chromosome, complete sequence.   
 Locus CP000151C on Burkholderia sp. (strain ATCC 17760 / NCIB 9086 / R18194 / 383) / 383) chromosome 1, complete sequence.   
 Locus CP000152E on Burkholderia sp. (strain ATCC 17760 / NCIB 9086 / R18194 / 383) / 383) chromosome 2, complete sequence.   
 Locus CP000152F on Burkholderia sp. (strain ATCC 17760 / NCIB 9086 / R18194 / 383) / 383) chromosome 2, complete sequence.   
 Locus CP000155B on Hahella chejuensis (strain KCTC 2396) chromosome, complete sequence.   
 Locus CP000155C on Hahella chejuensis (strain KCTC 2396) chromosome, complete sequence.   
 Locus CP000243A on Escherichia coli (strain UTI89 / UPEC) chromosome, complete sequence.   
 Locus CP000243D on Escherichia coli (strain UTI89 / UPEC) chromosome, complete sequence.   
 Locus CP000247A on Escherichia coli O6:K15:H31 (strain 536 / UPEC) chromosome, complete sequence.   
 Locus CP000247C on Escherichia coli O6:K15:H31 (strain 536 / UPEC) chromosome, complete sequence.   
 Locus CP000264A on Jannaschia sp. (strain CCS1) chromosome, complete sequence.   
 Locus CP000270B on Burkholderia xenovorans (strain LB400) chromosome 1, complete sequence.   
 Locus CP000282B on Saccharophagus degradans (strain 2-40 / ATCC 43961 / DSM 17024) chromosome, complete sequence.   
 Locus CP000285A on Chromohalobacter salexigens (strain DSM 3043 / ATCC BAA-138 / NCIMB 13768) chromosome, complete sequence.   
 Locus CP000305A on Yersinia pestis (biovar Antiqua Nepal516, strain Nepal516) chromosome, complete sequence.   
 Locus CP000305B on Yersinia pestis (biovar Antiqua Nepal516, strain Nepal516) chromosome, complete sequence.   
 Locus CP000305C on Yersinia pestis (biovar Antiqua Nepal516, strain Nepal516) chromosome, complete sequence.   
 Locus CP000305D on Yersinia pestis (biovar Antiqua Nepal516, strain Nepal516) chromosome, complete sequence.   
 Locus CP000305F on Yersinia pestis (biovar Antiqua Nepal516, strain Nepal516) chromosome, complete sequence.   
 Locus CP000305G on Yersinia pestis (biovar Antiqua Nepal516, strain Nepal516) chromosome, complete sequence.   
 Locus CP000305H on Yersinia pestis (biovar Antiqua Nepal516, strain Nepal516) chromosome, complete sequence.   
 Locus CP000308A on Yersinia pestis (biovar Antiqua Antiqua, strain Antiqua) chromosome, complete sequence.   
 Locus CP000308B on Yersinia pestis (biovar Antiqua Antiqua, strain Antiqua) chromosome, complete sequence.   
 Locus CP000308C on Yersinia pestis (biovar Antiqua Antiqua, strain Antiqua) chromosome, complete sequence.   
 Locus CP000308E on Yersinia pestis (biovar Antiqua Antiqua, strain Antiqua) chromosome, complete sequence.   
 Locus CP000308F on Yersinia pestis (biovar Antiqua Antiqua, strain Antiqua) chromosome, complete sequence.   
 Locus CP000308G on Yersinia pestis (biovar Antiqua Antiqua, strain Antiqua) chromosome, complete sequence.   
 Locus CP000352A on Ralstonia metallidurans (strain CH34 / ATCC 43123 / DSM 2839) chromosome, complete sequence.   
 Locus CP000378B on Burkholderia cenocepacia (strain AU 1054) chromosome 1, complete sequence.   
 Locus CP000438A on Pseudomonas aeruginosa (strain UCBPP-PA14) chromosome, complete sequence.   
 Locus CP000438C on Pseudomonas aeruginosa (strain UCBPP-PA14) chromosome, complete sequence.   
 Locus CP000438D on Pseudomonas aeruginosa (strain UCBPP-PA14) chromosome, complete sequence.   
 Locus CP000440A on Burkholderia cepacia (strain ATCC 53795 / AMMD) chromosome 1, complete sequence.   
 Locus CP000441E on Burkholderia cepacia (strain ATCC 53795 / AMMD) chromosome 2, complete sequence.   
 Locus CP000447A on Shewanella frigidimarina (strain NCIMB 400) chromosome, complete sequence.   
 Locus CP000453A on Alkalilimnicola ehrlichei (strain MLHE-1) chromosome, complete sequence.   
 Locus CP000458A on Burkholderia cenocepacia (strain HI2424) chromosome 1, complete sequence.   
 Locus CP000462B on Aeromonas hydrophila (subsp. hydrophila, strain ATCC 7966 / NCIB 9240) chromosome, complete sequence.   
 Locus CP000468A on Escherichia coli O1:K1 / APEC chromosome, complete sequence.   
 Locus CP000468D on Escherichia coli O1:K1 / APEC chromosome, complete sequence.   
 Locus CP000473A on Solibacter usitatus (strain Ellin6076) chromosome, complete sequence.   
 Locus CP000489A on Paracoccus denitrificans (strain Pd 1222) chromosome 1, complete sequence.   
 Locus CP000510A on Psychromonas ingrahamii (strain 37) chromosome, complete sequence.   
 Locus CP000512B on Acidovorax avenae (subsp. citrulli, strain AAC00-1) chromosome, complete sequence.   
 Locus CP000514D on Marinobacter aquaeolei (strain DSM 11845 / ATCC 700491 / / VT8) / VT8) chromosome, complete sequence.   
 Locus CP000521A on Acinetobacter baumannii (strain ATCC 17978 / NCDC KC 755) chromosome, complete sequence.   
 Locus CP000544A on Halorhodospira halophila (strain DSM 244 / SL1) chromosome, complete sequence.   
 Locus CP000572D on Burkholderia pseudomallei (strain 1106a) chromosome I, complete sequence.   
 Locus CP000573E on Burkholderia pseudomallei (strain 1106a) chromosome II, complete sequence.   
 Locus CP000573F on Burkholderia pseudomallei (strain 1106a) chromosome II, complete sequence.   
 Locus CP000573G on Burkholderia pseudomallei (strain 1106a) chromosome II, complete sequence.   
 Locus CP000573H on Burkholderia pseudomallei (strain 1106a) chromosome II, complete sequence.   
 Locus CP000573I on Burkholderia pseudomallei (strain 1106a) chromosome II, complete sequence.   
 Locus CP000578A on Rhodobacter sphaeroides (strain ATCC 17029 / ATH 2.4.9) chromosome 2, complete sequence.   
 Locus CP000644A on Aeromonas salmonicida (strain A449) chromosome, complete sequence.   
 Locus CP000668A on Yersinia pestis (strain Pestoides F) chromosome, complete sequence.   
 Locus CP000668B on Yersinia pestis (strain Pestoides F) chromosome, complete sequence.   
 Locus CP000668D on Yersinia pestis (strain Pestoides F) chromosome, complete sequence.   
 Locus CP000668E on Yersinia pestis (strain Pestoides F) chromosome, complete sequence.   
 Locus CP000668F on Yersinia pestis (strain Pestoides F) chromosome, complete sequence.   
 Locus CR354531A on Photobacterium profundum (strain SS9) chromosome 1, complete sequence.   
 Locus CR543861B on Acinetobacter sp. (strain ADP1) chromosome, complete sequence.   
 Locus CT573326A on Pseudomonas entomophila (strain L48) chromosome, complete sequence.   
