# Supplementary material for: Dissecting the bacterial type VI secretion system by a genome wide in silico analysis: what can be learned from available microbial genomic resources?
Source: BMC Genomics. 2009 Mar 12;10:104. doi: 10.1186/1471-2164-10-104 (PMC2660368; doi:10.1186/1471-2164-10-104)
Supplement: Additional file 8 — Bacterial strains not taken into account for the computations of genes frequencies and gene neighbourhood frequencies. List of bacterial strains not taken into account for the computations of genes frequencies and gene neighbourhood frequencies. [file 1471-2164-10-104-S8.doc]

| Status | Taxonomy | Strain name |
| --- | --- | --- |
| Used strain | Bacteria;Proteobacteria;Alphaproteobacteria;Rhizobiales;Rhizobiaceae;Rhizobium/Agrobacteriumgroup;Agrobacterium. | Agrobacterium tumefaciens (strain C58 / ATCC 33970, sub_strain Cereon) |
| Strain not taken into account | Bacteria;Proteobacteria;Alphaproteobacteria;Rhizobiales;Rhizobiaceae;Rhizobium/Agrobacteriumgroup;Agrobacterium. | Agrobacterium tumefaciens (strain C58 / ATCC 33970, sub_strain Dupont) |
| Used strain | Bacteria;Proteobacteria;Alphaproteobacteria;Rhodobacterales;Rhodobacteraceae;Rhodobacter. | Rhodobacter sphaeroides (strain ATCC 17023 / 2.4.1 / NCIB 8253 / DSM 158) |
| Strain not taken into account | Bacteria;Proteobacteria;Alphaproteobacteria;Rhodobacterales;Rhodobacteraceae;Rhodobacter. | Rhodobacter sphaeroides (strain ATCC 17029 / ATH 2.4.9) |
| Used strain | Bacteria;Proteobacteria;Betaproteobacteria;Burkholderiales;Burkholderiaceae;Burkholderia;Burkholderiacepaciacomplex. | Burkholderia cenocepacia (strain AU 1054) |
| Strain not taken into account | Bacteria;Proteobacteria;Betaproteobacteria;Burkholderiales;Burkholderiaceae;Burkholderia;Burkholderiacepaciacomplex. | Burkholderia cenocepacia (strain HI2424) |
| Used strain | Bacteria;Proteobacteria;Betaproteobacteria;Burkholderiales;Burkholderiaceae;Burkholderia;pseudomalleigroup. | Burkholderia pseudomallei (strain 1106a) |
| Strain not taken into account | Bacteria;Proteobacteria;Betaproteobacteria;Burkholderiales;Burkholderiaceae;Burkholderia;pseudomalleigroup. | Burkholderia pseudomallei (strain 1710b) |
| Strain not taken into account | Bacteria;Proteobacteria;Betaproteobacteria;Burkholderiales;Burkholderiaceae;Burkholderia;pseudomalleigroup. | Burkholderia pseudomallei (strain K96243) |
| Used strain | Bacteria;Proteobacteria;Betaproteobacteria;Burkholderiales;Burkholderiaceae;Cupriavidus. | Ralstonia eutropha (strain ATCC 17699 / H16 / DSM 428 / Stanier 337) |
| Strain not taken into account | Bacteria;Proteobacteria;Betaproteobacteria;Burkholderiales;Burkholderiaceae;Cupriavidus. | Ralstonia eutropha (strain JMP134) |
| Used strain | Bacteria;Proteobacteria;Gammaproteobacteria;Enterobacteriales;Enterobacteriaceae;Escherichia. | Escherichia coli O1:K1 / APEC |
| Strain not taken into account | Bacteria;Proteobacteria;Gammaproteobacteria;Enterobacteriales;Enterobacteriaceae;Escherichia. | Escherichia coli (strain EDL933 / ATCC 700927 / O157:H7 / EHEC) |
| Strain not taken into account | Bacteria;Proteobacteria;Gammaproteobacteria;Enterobacteriales;Enterobacteriaceae;Escherichia. | Escherichia coli (strain Sakai / O157:H7 / RIMD 0509952 / EHEC) |
| Strain not taken into account | Bacteria;Proteobacteria;Gammaproteobacteria;Enterobacteriales;Enterobacteriaceae;Escherichia. | Escherichia coli (strain UTI89 / UPEC) |
| Strain not taken into account | Bacteria;Proteobacteria;Gammaproteobacteria;Enterobacteriales;Enterobacteriaceae;Escherichia. | Escherichia coli O6 (strain UPEC / O6:H1 / ATCC 700928 / CFT073) |
| Strain not taken into account | Bacteria;Proteobacteria;Gammaproteobacteria;Enterobacteriales;Enterobacteriaceae;Escherichia. | Escherichia coli O6:K15:H31 (strain 536 / UPEC) |
| Used strain | Bacteria;Proteobacteria;Gammaproteobacteria;Enterobacteriales;Enterobacteriaceae;Shigella. | Shigella flexneri (serovar 2a, strain 2457T / ATCC 700930) |
| Strain not taken into account | Bacteria;Proteobacteria;Gammaproteobacteria;Enterobacteriales;Enterobacteriaceae;Shigella. | Shigella flexneri (serovar 2a, strain 301) |
| Used strain | Bacteria;Proteobacteria;Gammaproteobacteria;Enterobacteriales;Enterobacteriaceae;Yersinia. | Yersinia pestis (biovar Antiqua Antiqua, strain Antiqua) |
| Strain not taken into account | Bacteria;Proteobacteria;Gammaproteobacteria;Enterobacteriales;Enterobacteriaceae;Yersinia. | Yersinia pestis (biovar Antiqua Nepal516, strain Nepal516) |
| Strain not taken into account | Bacteria;Proteobacteria;Gammaproteobacteria;Enterobacteriales;Enterobacteriaceae;Yersinia. | Yersinia pestis (biovar Mediaevalis, strain 91001) |
| Strain not taken into account | Bacteria;Proteobacteria;Gammaproteobacteria;Enterobacteriales;Enterobacteriaceae;Yersinia. | Yersinia pestis (biovar Mediaevalis, strain KIM5) |
| Strain not taken into account | Bacteria;Proteobacteria;Gammaproteobacteria;Enterobacteriales;Enterobacteriaceae;Yersinia. | Yersinia pestis (biovar Orientalis, strain CO-92) |
| Strain not taken into account | Bacteria;Proteobacteria;Gammaproteobacteria;Enterobacteriales;Enterobacteriaceae;Yersinia. | Yersinia pestis (strain Pestoides F) |
| Used strain | Bacteria;Proteobacteria;Gammaproteobacteria;Pseudomonadales;Moraxellaceae;Acinetobacter. | Acinetobacter baumannii (strain ATCC 17978 / NCDC KC 755) |
| Strain not taken into account | Bacteria;Proteobacteria;Gammaproteobacteria;Pseudomonadales;Moraxellaceae;Acinetobacter. | Acinetobacter sp. (strain ADP1) |
| Used strain | Bacteria;Proteobacteria;Gammaproteobacteria;Pseudomonadales;Pseudomonadaceae;Pseudomonas. | Pseudomonas aeruginosa (strain LMG 12228 / ATCC 15692 / PRS 101 / 1C / |
| Strain not taken into account | Bacteria;Proteobacteria;Gammaproteobacteria;Pseudomonadales;Pseudomonadaceae;Pseudomonas. | Pseudomonas aeruginosa (strain UCBPP-PA14) |
| Used strain | Bacteria;Proteobacteria;Gammaproteobacteria;Pseudomonadales;Pseudomonadaceae;Pseudomonas. | Pseudomonas fluorescens (strain Pf-5 / ATCC BAA-477) |
| Strain not taken into account | Bacteria;Proteobacteria;Gammaproteobacteria;Pseudomonadales;Pseudomonadaceae;Pseudomonas. | Pseudomonas fluorescens (strain PfO-1) |
| Used strain | Bacteria;Proteobacteria;Gammaproteobacteria;Vibrionales;Vibrionaceae;Vibrio. | Vibrio vulnificus (strain CMCP6) |
| Strain not taken into account | Bacteria;Proteobacteria;Gammaproteobacteria;Vibrionales;Vibrionaceae;Vibrio. | Vibrio vulnificus (strain YJ016) |
| Used strain | Bacteria;Proteobacteria;Gammaproteobacteria;Xanthomonadales;Xanthomonadaceae;Xanthomonas. | Xanthomonas oryzae oryzae (strain KXO85 / KACC10331) |
| Strain not taken into account | Bacteria;Proteobacteria;Gammaproteobacteria;Xanthomonadales;Xanthomonadaceae;Xanthomonas. | Xanthomonas oryzae (pathovar oryzae, strain MAFF 311018) |
