# Supplementary material for: Dissecting the bacterial type VI secretion system by a genome wide in silico analysis: what can be learned from available microbial genomic resources?
Source: BMC Genomics. 2009 Mar 12;10:104. doi: 10.1186/1471-2164-10-104 (PMC2660368; doi:10.1186/1471-2164-10-104)
Supplement: Additional file 3 — Relationship between phylogeny and T6SS gene content. Rows represent T6SS loci and columns represent protein functional classes (based on COG assignment and TribeMCL protein clusters). The tree on the left is the consensus phylogenetic tree (super-tree, see Materials and Methods §5, manually rooted) whereas the two upper dendrograms represent a hierarchical clustering of the two sets of phylogenetic profiles. Core T6SS conserved proteins are depicted with the same color code as in Figure 4. Letters in front of bacteria names correspond to the four groups proposed by Bingle and co-workers [14], '#' marks functional T6SS loci depicted in Table 1 and included in this figure. [file 1471-2164-10-104-S3.pdf]

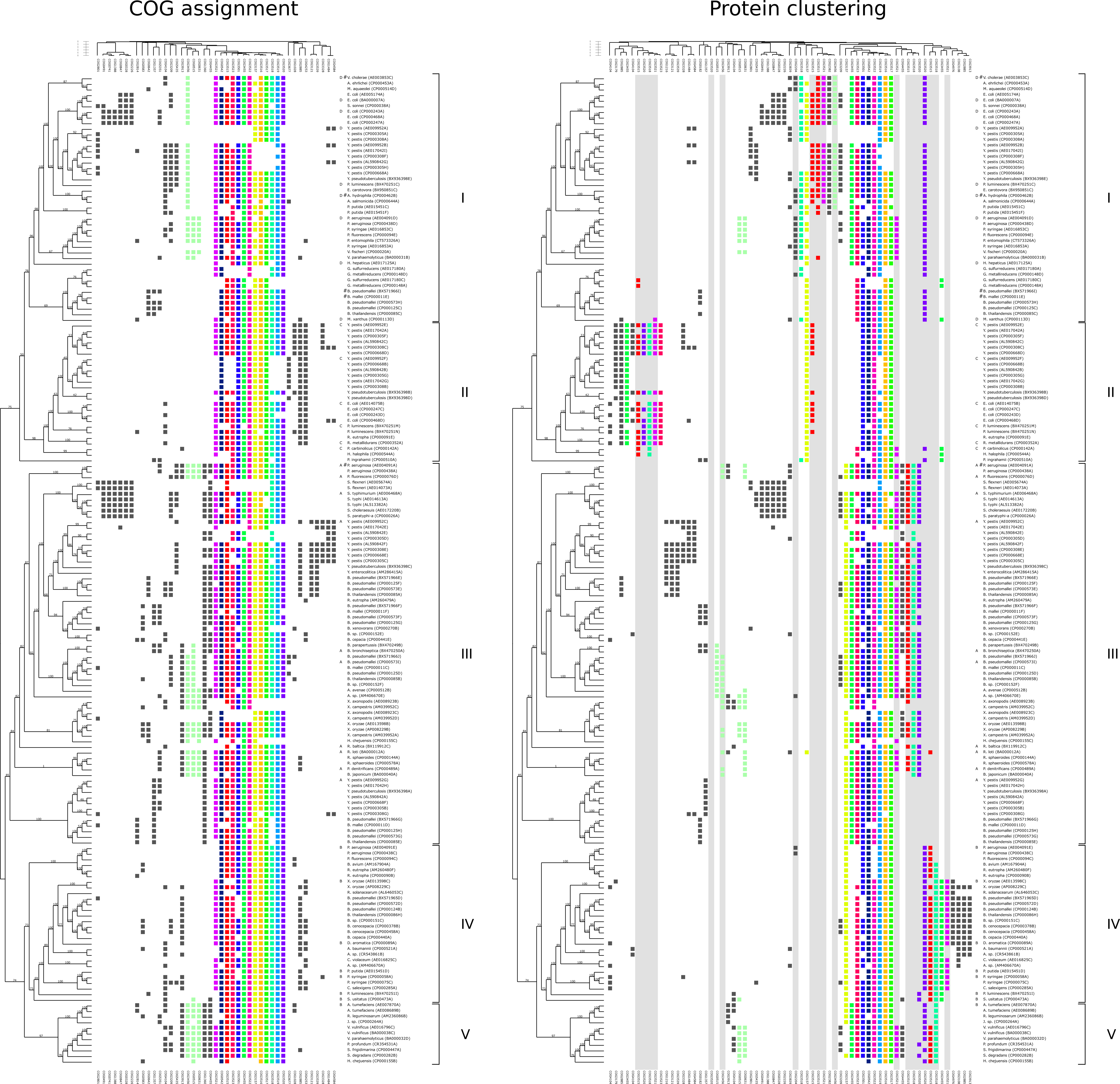

COG assignment
Protein clustering
I
II
III
IV
V
